# Identification of lncRNAs and Genes Responsible for Fatness and Fatty Acid Composition Traits between the Tibetan and Yorkshire Pigs

**DOI:** 10.1155/2019/5070975

**Published:** 2019-06-02

**Authors:** Peng Shang, Wenting Li, Gang Liu, Jian Zhang, Mengrou Li, Lvcao Wu, Kejun Wang, Yangzom Chamba

**Affiliations:** ^1^Animal Science College, Tibet Agriculture and Animal Husbandry University, Linzhi, Xizang, China; ^2^College of Animal Sciences and Veterinary Medicine, Henan Agricultural University, Zhengzhou, Henan, China; ^3^National Center for Preservation and Utilization of Animal Genetic Resources, National Animal Husbandry Service, Beijing 100193, China

## Abstract

Tibetan pigs from the Tibetan Plateau are characterized with a significant phenotypic difference relative to lowland pigs. In this study, a significant difference of the fatness and fatty acid composition traits was observed between the Tibetan and Yorkshire pigs. To uncover the involved mechanism, the expression profile of long noncoding RNAs (lncRNAs) and genes was compared between them. After serial filtered steps, 1,964 lncRNAs were obtained through our computational pipeline. In total, 63 and 715 lncRNAs and genes were identified to be differentially expressed. Evidence from *cis-* and *trans-*targeting analysis of lncRNAs demonstrated that some lncRNAs, such as *MSTRG.14097* and *MSTRG.8034*, played important roles in the fatness and fatty acid composition traits. Bioinformatics analysis revealed that many candidate genes were responsible for the two traits. Of these, *FASN*, *ACACA*, *SCD*, *ME3*, *PDHB*, *ACSS1*, *ACSS2*, and *ACLY* were identified, which functioned in regulating the level of hexadecanoic acid, hexadecenoic acid, octadecenoic acid, and monounsaturated fatty acid. And *LPGAT1*, *PDK4*, *ACAA1*, and *ADIPOQ* were associated with the content of stearic acid, octadecadienoic acid, and polyunsaturated fatty acid. Candidate genes, which were responsible for fatness trait, consisted of *FGF2*, *PLAG1*, *ADIPOQ*, *IRX3*, *MIF*, *IL-34*, *ADAM8*, *HMOX1*, *Vav1*, and *TLR8*. In addition, association analysis also revealed that 34 and 57 genes significantly correlated to the fatness and fatty acid composition trait, respectively. Working out the mechanism caused by these lncRNAs and candidate genes is proven to be complicated but is invaluable to our understanding of fatness and fatty acid composition traits.

## 1. Introduction

Tibetan pigs mainly originated from the Tibetan Plateau, which had undergone the long-term natural selection in the harsh environment. It indicated that many candidate genes related to germplasm of the Tibetan pigs, such as energy metabolism, might contribute to the adaptability to hostile environment [1]. However, the heritable adaptions to the extreme environment is quite sophisticated, which is influenced by many factors [2], for example, cardiopulmonary function, immunity, blood pressure, and fat metabolism [3–5]. Furthermore, previous publication also demonstrated that excessive caloric intake led to fat deposition, and further, the excessive fat would participate in hypoxia and inflammation (Heinonen et al. 2016).

So we took another look on this scientific question that is due to thicker back fat and higher intramuscular fat in the Tibetan pigs, which could stably deliver the energy to the body and guarantee the inflammatory response in such a harsh environment. Additionally, phenotype difference of the fatness and fatty acid composition traits was also observed between the Tibetan and Yorkshire pigs in this study. Multiple elements, including coding genes [6], miRNA [7], and DNA methylation [8], had been confirmed to be functioned in the pig fatness-related traits. But long noncoding RNAs (lncRNAs) involved in the process of fatness and fatty acid composition has not been reported in the Tibetan pigs yet. LncRNAs, groups of RNAs more than 200 nucleotides, had been demonstrated to play a crucial role in various biological processes, including metabolic activity, fat deposition, and biological development process [9]. In this study, comparative analysis of lncRNAs and mRNAs was performed between the Tibetan and Yorkshire pigs to identify the candidate genes responsible for fatness and fatty acid composition traits. These findings will help us to further decipher the molecular mechanism of adaptability to hypoxic conditions of the Tibetan pig from the sight of fat metabolism.

## 2. Materials and Methods

### 2.1. Collection of Phenotype and Sequencing Data

All phenotypes of the Tibetan and Yorkshire pig group, which included the fatness and fatty acid composition traits, were collected at days 180 [10]. Each group have three individuals. Phenotypic statistics of the fatness trait, which consisted of left leaf fat percentage, back-fat thinness at shoulder, back-fat thinness at rib, back-fat thinness at first lumbar, back-fat thinness at last lumbar, and average back-fat thinness, were adjusted to the live-weight. Meanwhile, 14 fatty acid compositions were obtained, including tetradecanoic acid, hexadecanoic acid, hexadecenoic acid, stearic acid, octadecenoic acid, octadecadienoic acid, octadecatrienoic acid, eicosenoic acid, eicosadienoic acid, eicosatrienoic acid, eicosatetraenoic acid, saturated fatty acid, monounsaturated fatty acid, and polyunsaturated fatty acid. Total RNA was extracted from fat tissue. The sequence data was collected based on the Illumina Hiseq 2000 platform [10]. All the RNA-Seq data was collected in the NCBI database (SRP090525) [10].

### 2.2. Identification Pipeline of lncRNAs

Sequencing data were assessed for quality using FastQC. High quality clean data was used in RNA-Seq analysis. Clean pair-end reads with FASTQ format were aligned to the Sscrofa 11.1 genome assembly, and novel splice junctions were determined using Hisat2 (v2.0.0-beta). The default parameters were used in the analysis, except for the “-G” option tighter with the GTF file of Ensembl gene annotation (Sscrofa 11.1.91). The alignment data with the BAM format were subjected to the StringTie (v1.3.0) to assemble transcripts. All transcripts from all samples were merged together using StringTie. Then, gffcompare (v0.10.1) was applied to match the transcript pool to Ensembl gene sets (Sscrofa 11.1.91). Transcripts, of which class code with “i,” “u,” “o,” and “x,” were subjected to lncRNA identification. To avoid incomplete assemble and false positive rate, the putative lncRNA transcripts were filtered with transcript length > 200 bp and exon number > 2. Remaining transcripts were scored with the CNCI tool [11] and CPC (Coding Potential Calculator) [12]. The transcripts, which both the CNCI and CPC scores were below 0, would be considered as the lncRNAs.

### 2.3. Differential Expression and Functional Enrichment Analysis

The number of reads that mapped to each gene was calculated with HTSeq [13]. Then, DESeq2 [14] was used to identify the differentially expressed genes with the criterion (fold change > 2 and adjusted *P* value < 0.05). Enrichment analysis (GO and KEGG pathway) was performed with the Metascape database based on human homology gene list [15]. Association analysis was performed between the expression level of each differentially expressed gene and phenotypes from the two traits. “Corrplot” and “psych” packages were used to compute and visualize the Pearson correlation coefficients under *R* environment. The Bonferroni correction was used to determine the significance level of each association event.

### 2.4. Targeting Gene Prediction and Functional Analysis

The *cis*-regulating role of lncRNAs was defined as those exerting effects on neighboring genes [16]. Differentially expressed genes (DEGs) located in 100 kb upstream or downstream of lncRNAs were identified by Bedtools [17]. The positional relationship between differentially expressed lncRNAs and DEGs was firstly investigated. Further, the expression correlation between lncRNA-DEGs pairs was computed with the Pearson method. Additionally, for transregulation relationship, the expression correlation was computed between differentially expressed lncRNA and DEGs with the Pearson correlation. The FDR correction was performed to determine the significance level.

### 2.5. Quantitative Real-Time PCR (qPCR)

Seven differentially expressed mRNAs and six differential lncRNAs were validated via qPCR. Total RNA was extracted from back-fat tissue using TRIzol reagent according to the manufacturer's instructions (Life Technologies). cDNA synthesis was performed with 1 *μ*g of total RNA, following the protocol accompanied with the FastQuant RT Kit (Tiangen). Quantitative PCR (qPCR) amplification was conducted as described previously [7].

## 3. Results and Discussion

### 3.1. Descriptive Statistics of the Fatness and Fatty Acid Composition Traits

**Figure 1:**
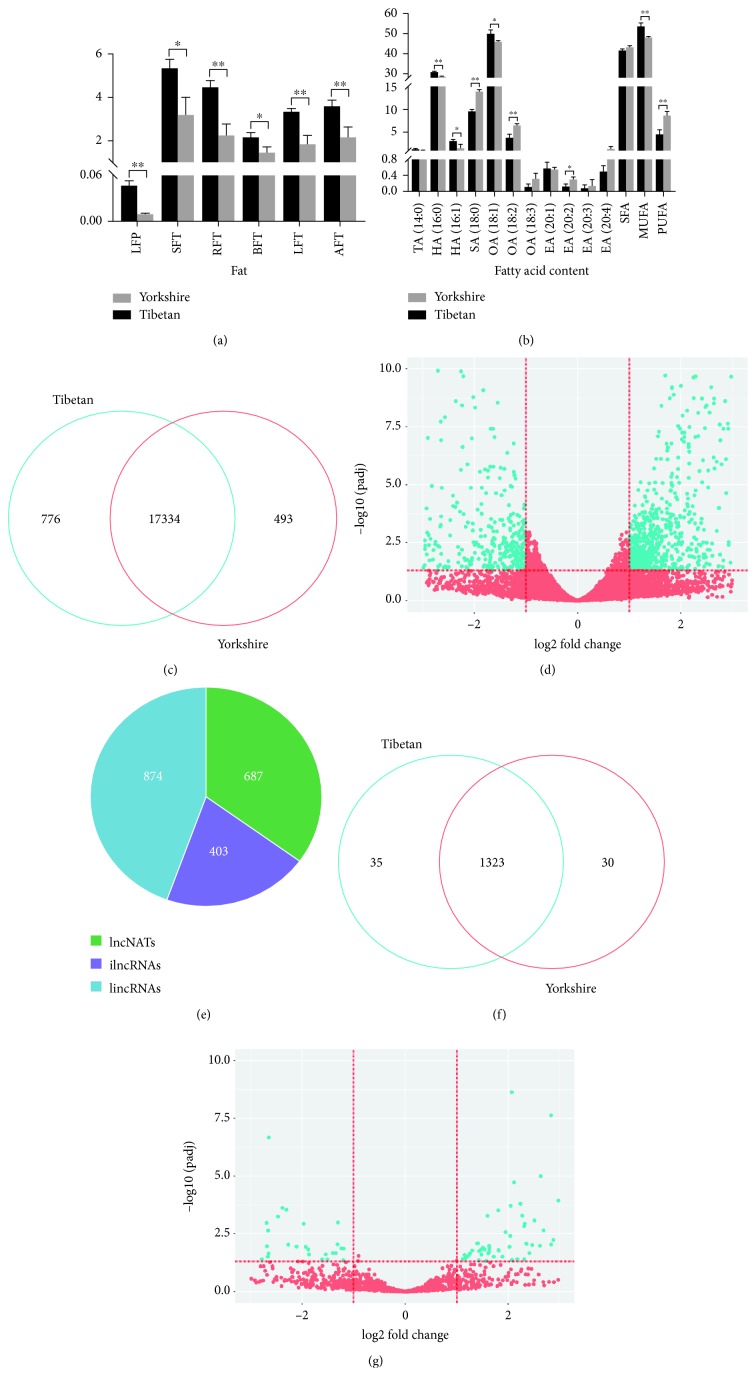
Descriptive statistics of phenotypes and RNA-Seq data. (a) Phenotypic statistics of the fatness trait. Back-fat thinness was adjusted to the body weight. (b) Phenotypic statistics of the fatty acid composition trait. (c) The number of quantified transcripts in the Tibetan and Yorkshire pigs. (d) Volcano plot of differentially expressed transcripts between the Tibetan and Yorkshire pigs. (e) Classification of identified lncRNAs. (f) The number of quantified LncRNAs in the Tibetan and Yorkshire pigs. (g) Volcano plot of differentially expressed lncRNAs between the Tibetan and Yorkshire pigs. LFP: left leaf fat percentage; SFT: back-fat thinness at shoulder; RFT: back-fat thinness at rib; BFT: back-fat thinness at first lumbar; LFT: back-fat thinness at last lumbar; AFT: average back-fat thinness; TA (14:0): tetradecanoic acid; HA (16:0): hexadecanoic acid; HA (16:1): hexadecenoic acid; SA (18:0): stearic acid; OA (18:1): octadecenoic acid; OA (18:2): octadecadienoic acid; OA (18:3): octadecatrienoic acid; EA (20:1): eicosenoic acid; EZ (20:2): eicosadienoic acid; EA (20:3): eicosatrienoic acid; EA (20:4): eicosatetraenoic acid; SFA: saturated fatty acid; MUFA: monounsaturated fatty acid; PUFA: polyunsaturated fatty acid.

Phenotypes of the fatness and fat composition traits from the Tibetan and Yorkshire pigs were collected and analyzed. Higher fat content was observed in the Tibetan group than the Yorkshire group, such as back-fat thickness at different positions and leaf fat percentage (Figure 1(a)). Evidence from the fat composition trait demonstrated that the levels of fatty acids, including hexadecenoic acid, hexadecanoic acid, octadecenoic acid, and monounsaturated fatty acid, were significantly higher in the Tibetan group compared to the Yorkshire group, while stearic acid, octadecadienoic acid, eicosadienoic acid, and polyunsaturated fatty acid were significantly abundant in the Yorkshire group relative to the Tibetan group (Figure 1(b)). In this case, RNA-Seq data was collected from back-fat tissues between three Tibetan pigs and three Yorkshire pigs.

### 3.2. Identification of Differentially Expressed lncRNAs and Genes

**Figure 2:**
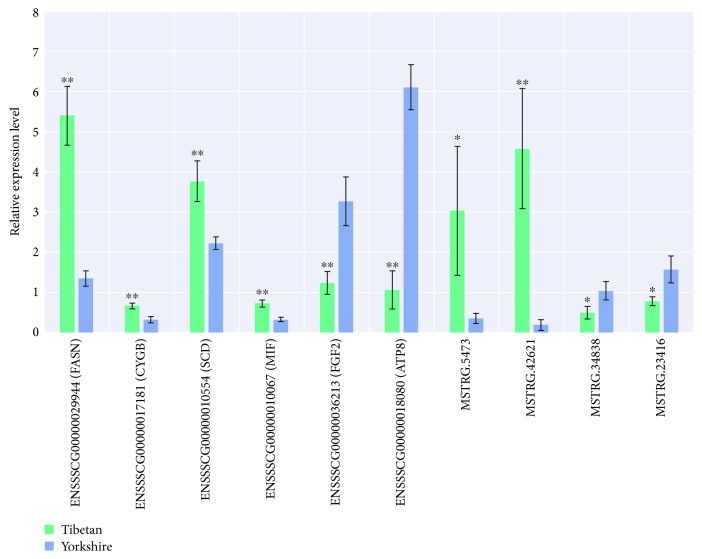
Validation of DEGs and lncRNAs with qPCR.

Clean reads were mapped to the Sscrofa 11.1 genome assembly, which mapping rates more than 83.1%. Of these, more than 84% reads were aligned on the CDS, 5′UTR, and 3′UTR region (Figure S1). A total of 17,334 genes were commonly quantified, then 776 and 493 genes were uniquely expressed in the Tibetan group and the Yorkshire group, respectively (Figure 1(c)). Specific expression is the extreme situation of differential expression, so all genes were subjected to the identification of differentially expressed genes. After statistical testing, 715 differentially expressed genes (DEGs) were identified, including 472 upregulated and 243 downregulated genes (Figure 1(d) and Table S1). After a series of filtered steps (Materials and Methods), 1,964 lncRNAs were identified based on our computational pipeline, which consist of 874 intergenic lncRNAs (lincRNAs), 687 natural antisense lncRNAs (lncNATs), and 403 intronic lncRNAs (ilncRNAs) (Figure 1(e) and Table S2). Of these, 1,323 lncRNA genes were commonly quantified, while 35 and 30 lncRNAs were uniquely expressed in the Tibetan and Yorkshire pigs (Figure 1(f)). Relative to the Yorkshire group, we found 63 lncRNAs differentially expressed in the Tibetan group, which included 44 upregulated and 19 downregulated lncRNAs (Figure 1(g) and Table S3). To validate the RNA-Seq results, six DEGs and four differentially expressed lncRNAs were randomly selected for qPCR assay (Figure 2). Figure 2 showed the result of the relative expression level between two groups, which was consistent with the RNA-Seq data. It indicated that identification and abundance estimation of genes were highly reliable in this study.

### 3.3. Candidate Genes Related to Fatty Acid Metabolism

**Figure 3:**
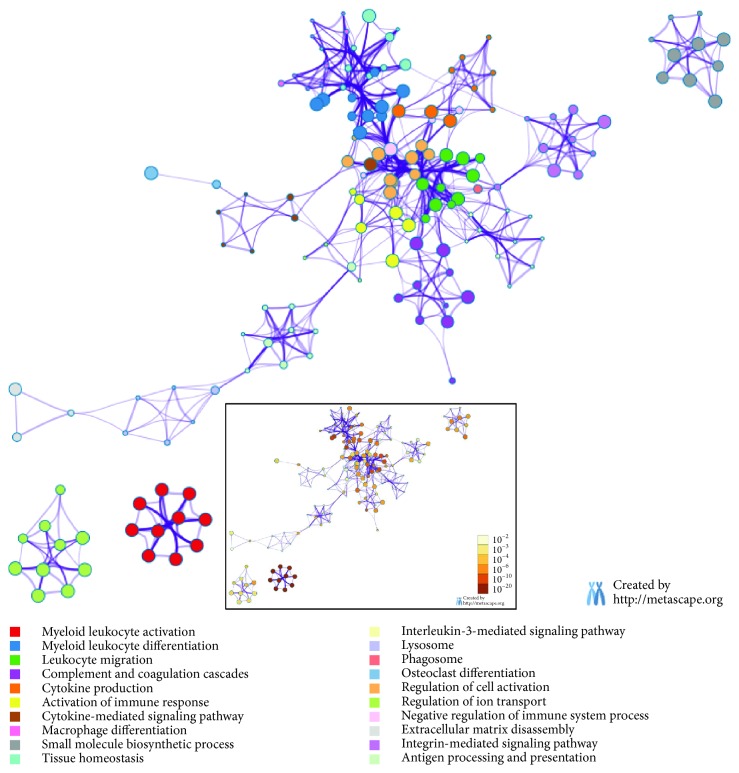
Functional enrichment analysis with upregulated DEGs. Enriched ontology clusters which is colored by cluster ID. *P* value of clusters was embedded at the bottom.

To further understand the potential genes responsible for the phenotype difference, differentially expressed genes were subjected to functional enrichment analysis. Biological process and KEGG pathway terms significantly enriched by upregulated DEGs included small molecule biosynthetic process (consists of “fatty acid biosynthetic process,” “lipid biosynthetic process,” etc.), many immune-related classes (“activation of immune response,” “macrophage differentiation,” “interleukin-3-mediated signaling pathway,” etc.), and 4 KEGG pathway classes (“complement and coagulation cascades,” “lysosome,” “phagosome,” and “osteoclast differentiation”) (Figure 3 and Table S4).

Many upregulated DEGs were enriched in fatty acid biosynthetic-related terms, which included *FASN*, *SCD*, *ME3*, *PDHB*, *ACACA*, *ACLY*, *ACSS1*, and *ACSS2*. It had been demonstrated that the polymorphism of the *FASN* gene is associated with blood monounsaturated fatty acid and saturated fatty acid [18]. *FASN* is a metabolic enzyme that catalyzed the synthesis of long-chain fatty acids through condensing acetyl-CoA and malonyl-CoA, and its main product is hexadecanoic acid [19]. Malonyl-CoA is the main substrate which was synthesized with acetyl-CoA by *ACACA* (acetyl-CoA carboxylase) [20]. Furthermore, the synthesis of monounsaturated fatty acid, including hexadecenoic acid and octadecenoic acid, was processed by *SCD* (stearoyl-CoA desaturase) [18]. Variation of *SCD* was demonstrated to correlate with the oleic acid, SFA, and MUFA content in pork [21]. *ME3* is an important paralog of *ME1*, together with *ACACA* and *PDHB* (Pyruvate Dehydrogenase E1 Beta Subunit) involved in the fatty acid synthesis process and pyruvate metabolism pathway (Table S4). *ACSS1* and *ACSS2* are both members of the acyl-CoA synthetase family, which was reported to function in the oleic acid (octadecenoic acid) synthesis process [22]. *ACLY* was demonstrated to be associated with the fatty acid content of milk in dairy cattle [23]. It suggested that many genes, such as *FASN*, *ACACA*, *SCD*, *ME3*, *PDHB*, *ACSS1*, *ACSS2*, and *ACLY*, were responsible for the higher monounsaturated fatty acid, octadecenoic acid, hexadecenoic acid, and hexadecanoic acid in the Tibetan group relative to the Yorkshire group.

**Figure 4:**
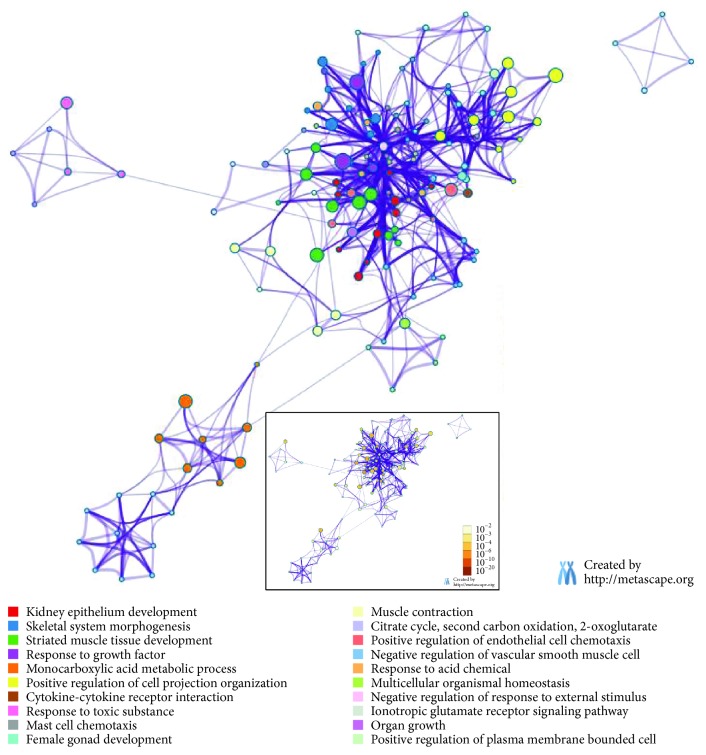
Functional enrichment analysis with downregulated DEGs. Enriched ontology clusters which is colored by cluster ID. *P* value of clusters was embedded at the bottom.

The biological process class of the “monocarboxylic acid metabolic process” was also enriched by downregulated DEGs (Figure 4 and Table S5), which consisted of “fatty acid biosynthetic process,” “fatty acid metabolic process,” “carboxylic acid biosynthetic process,” “monocarboxylic acid biosynthetic process,” etc. Some DEGs involved in fatty acid-related terms were highlighted, since higher level of stearic acid, octadecadienoic acid, eicosadienoic acid, and polyunsaturated fatty acid was observed in the Yorkshire group relative to the Tibetan group (Figure 1(b)). *LPGAT1* (lysophosphatidylglycerol specific acyltransferase) recognized various acyl-CoAs and LPGs as substrates but preferred to long-chain saturated fatty acyl-CoAs and oleoyl-CoA as acyl donors [24]. Genome-wide association analysis found that the *LPGAT1* gene was associated with stearic acid content of plasma [25]. *PDK4*-specific DNA methylation was positively associated with polyunsaturated fatty acid, i.e., eicosapentaenoic and arachidonic acid [26]. *ACAA1* was reported to be involved in the fatty acid *β*-oxidation signal pathway [27]. The *ADIPOQ* level was associated with the polyunsaturated fatty acid content in animal models [28]. *ELVO6* is the enzyme that catalyzed the transformative reaction from hexadecanoic acid into stearic acid, but no significant difference was observed between groups in this study. These findings revealed the *LPGAT1*, *PDK4*, *ACAA1*, and *ADIPOQ* genes were associated with the higher level of stearic acid, octadecadienoic acid, and polyunsaturated fatty acid in the Yorkshire pigs.

### 3.4. Candidate Genes Involved in the Immune Function Affecting Fat Metabolism

It has been surveyed that fat mass will be augmented in response to excess caloric intake leading to obesity and further to inflammatory signaling [29]. As part of the immune system, adipose tissue had been identified to be involved in inflammatory response which also inversely participated in obesity and insulin resistance [30]. GO terms, included “leukocyte migration,” “activation of immune response,” and “macrophage differentiation,” were enriched by upregulated DEGs (Figure 3 and Table S4). *MIF* (macrophage migration inhibitory factor) deficiency ameliorated high-fat diet-induced insulin resistance in mice and reduced adipose inflammation [31]. *IL-34* is associated with obesity, chronic inflammation, and insulin resistance [32]. *ADAM8* was involved in chronic inflammation in fat tissue that is contributing to the development of obesity-related insulin resistance [33]. *HMOX1* (heme oxygenase 1) could rescue adipocyte dysfunction in mice, and this gene is important to the adaption of chronic hypoxemia [34, 35]. Loss of *Vav1* in MSCs led to spontaneous adipogenic and displayed an increase in fat content [36]. The *TLR8* gene was significantly upregulated in obesity as compared with lean individuals, the increase of which has consensus with inflammatory signatures [37]. These findings revealed that many genes, involving in immune-related processes, played an important role in adipocyte metabolism.

### 3.5. Opposite Effect of *FGF2*, *PLAG1*, *ADIPOQ*, and *IRX3* on Fat and Other Tissues

Many interesting terms of biological process were also enriched by downregulated DEGs (Figure 4 and Table S5), including “skeletal system morphogenesis,” “organ growth,” “kidney epithelium development,” and “striated muscle tissue development.” These genes, such as *FGF2*, *PLAG1*, *ADIPOQ*, and *IRX3*, might participate in the development of skeletal muscle, kidney, or other organs [38–40] but would have a negative effect on fat deposition. It had been demonstrated that *FGF2* played a negative effect on adipogenesis [41]. Variants located in *PLAG1* had confirmed to be associated with the fatness and growth traits in wild boar and Yorkshire F2 intercross pig population [42]. Adiponectin (*ADIPOQ*) is secreted from adipose tissue into the bloodstream to regulate other organ and to be inversely correlated with body mass index [43, 44]. *IRX3* could promote browning of white adipocyte and correlate with human obesity [45]. It is well-known that the growth rate of the Yorkshire pigs is significantly higher than the Tibetan pigs. Furthermore, adipose tissue is a true endocrine organ secreting some mediators, which regulated adipose, skeletal muscle, and other organ using exosomes or via auto-paracrine manner [46, 47]. So, these genes might exert a negative effect on fat deposition but a positive role in skeletal muscle and other gran by cross-talking. KEGG pathway analysis also demonstrated that some downregulated genes were involved in “cytokine-cytokine receptor interaction” (Figure 4 and Table S5). It might reflect the communications between adipose and other tissues.

### 3.6. Association Analysis between Expression Level and Phenotypes

**Figure 5:**
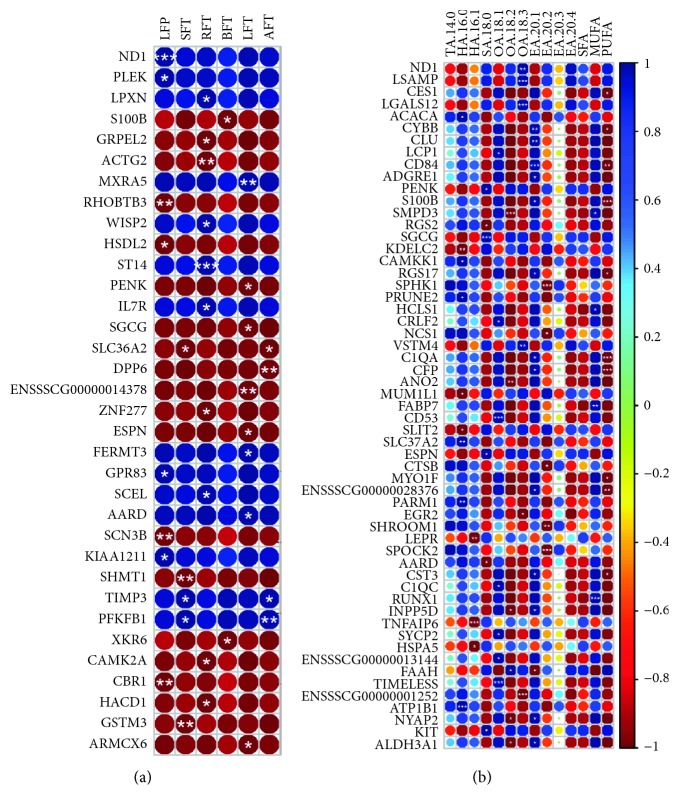
Association analysis between DEGs and phenotypes. (a) Significant correlation events on six phenotypes of the fatness trait. (b) Significant correlation events on fourteen phenotypes of the fatty acid composition trait. Note: circle color and circle size represent the correlation coefficients. Significance level was tested by Bonferroni correction. ^∗^ *P* value < 0.05; ^∗∗^ *P* value < 0.01; ^∗∗∗^ *P* value < 0.001. The abbreviation of phenotype is the same as in Figure 1.

To better identify the potential function of differentially expressed gene, association analysis between gene expression level and phenotypes was investigated. After statistical testing, 34 DEGs were significantly associated with the fatness trait, which consisted of 14 positive and 20 negative correlation events (Figure 5(a)). A total of 10 genes, consist of *LPXN*, *IL7R*, *GRPEL2*, *ACTG2*, *WISP2*, *ST14*, *ZNF277*, *SCEL*, *CAMK2A*, and *HACD1*, were related to the RFT trait. Eight genes, including *ND1*, *PLEK*, *RHOBTB3*, *HSDL2*, *GPR83*, *SCN3B*, *KIAA1211*, and *CBR1*, were associated with the LFP trait. There were also 8 genes, including *ENSSSCT00000014378*, *MXRA5*, *PENK*, *SGCG*, *ESPN*, *FERMT3*, *AARD*, and *ARMCX6*, associated with the LFT trait. Five genes were correlated to the SFT trait, including *SLC36A2*, *SHMT1*, *GSTM3*, *PFKFB1*, and *TIMP3*. Four genes were significantly correlated to the AFT trait, such as *DPP6*, *SLC36A2*, *PFKFB1*, and *TIMP3*. Only 2 genes, *S100B* and *XKR6*, were involved in BFT. Furthermore, three genes, *SLC36A2*, *TIMP3*, and *PFKFB1*, were associated with both the SFT and AFT traits. Of these genes, some had been demonstrated to be involved in fat or adipocyte-related physiological process. *TIMP3* overexpression in macrophages resulted in reduced activation of oxidative stress signals related to lipid peroxidation in fat tissue [48]. *IL-7R* deletion ameliorated diet-induced obesity and insulin resistance in mice [49]. *SLC36A2* was identified as membrane surface marker of brown and beige adipocytes [50]. A significant SNP, near the *XKR6* gene, was associated with body fat distribution [51].

There were also 57 DEGs significantly associating with the fat composition trait (Figure 5(b)). A total of 14 genes were significantly associated with the eicosenoic acid content, which included *CYBB*, *CLU*, *CD84*, *ADGRE1*, *S100B*, *RGS17*, *C1QA*, *CFP*, *ENSSSCG00000028376*, *CST3*, *INPP5D*, *FAAH*, *NYAP2*, and *ALDH3A1*. There were also 10 genes correlated to polyunsaturated fatty acid, which included *CES1*, *CYBB*, *CD84*, *S100B*, *RGS17*, *C1QA*, *CFP*, *MYO1F*, *ENSSSCG00000028376*, and *CST3*. Nine genes, such as *ACACA*, *KDELC2*, *CAMKK1*, *PRUNE2*, *MUM1L1*, *SLIT2*, *SLC37A2*, *PARM1*, and *ATP1B1*, were related to hexadecanoic acid level. Totally, 7 genes, *LCP1*, *CRLF2*, *CD53*, *C1QC*, *SYCP2*, *ENSSSCG00000013144*, and *TIMELESS*, were associated with the octadecenoic acid content. *PENK*, *RGS2*, *SGCG*, *ESPN*, *AARD*, and *KIT* were associated with the stearic acid content. *SMPD3*, *ANO2*, *INPP5D*, *FAAH*, *NYAP2*, and *ALDH3A1* were related to the octadecadienoic acid content. For octadecatrienoic acid, *ND1*, *LSAMP*, *LGALS12*, *VSTM4*, *EGR2*, and *ENSSSCG00000001252* were related. There were also 5 genes, *SPHK1*, *NCS1*, *CTSB*, *SHROOM1*, and *SPOCK2*, associated with the eicosadienoic acid level. Four genes, *SMPD3*, *HCLS1*, *FABP7*, and *RUNX1*, were correlated to monounsaturated fatty acid. There were only 3 genes, *LEPR*, *TNFAIP6*, and *HSPA5*, related to hexadecenoic acid. Some genes were associated with more than one trait. For example, *CYBB*, *CD84*, *S100B*, *RGS17*, *C1QA*, *CFP*, *ENSSSCG00000028376*, and *CST3* were associated with both the eicosenoic acid and polyunsaturated fatty acid levels. However, no gene was observed to significantly associate with the other phenotypes of fatty acid. Most genes were first reported to be associated with the fatty acid content in this study. The reason behind is that most genes might indirectly involve in fatty acid composition, and less studies about fatty acid composition were reported. SNPs of *LEPR* were reported to be associated with SFA in subcutaneous fat. In this study, the *LEPR* expression level significantly correlated with the hexadecenoic acid content. To some extent, these findings are partly similar to published result and would be regarded as important foundational evidence for further study.

### 3.7. Prediction of Regulatory Actions for Differentially Expressed LncRNAs

To find the potentially biological function of differentially expressed lncRNAs, the *cis*- and *trans*-regulating models were analyzed. For the *cis-acting* model, DEGs physically located within 100 kb near the differentially expressed lncRNAs were obtained. The 9 pairs of lncRNA-DEGs with *cis*-regulating effect were identified (Table 1). Of these, 7 pairs passed the significant threshold of FDR correction. The *cis-*regulatory effect of *MSTRG.8034* on *ACACA* is positive, but it is not significant. It was reported that *ACACA* catalyzed the first committed step of fatty acid synthesis, from carboxylation of acetyl-CoA to malonyl-CoA, leading to the biosynthesis of long-chain fatty acids [52]. A significant *cis-regulatory* effect of *MSTRG.29167* on the *RDH16* gene was also observed (Table 1). No obvious evidence was reported that *RDH16* was correlated to fatness or fat composition trait, but it also showed differentially expressed in fat tissue between lean and obese pig breeds [53].

*Trans-acting* correlations of expression between differential lncRNAs and DEGs were examined. A total of 49 significant targeting events between 21 lncRNAs and 42 DEGs were found (Table S6). Two pairs of lncRNA-DEGs, *MSTRG.14097*-*SCD* and *MSTRG.42256-NPDC1*, were identified by both the *Trans-* and *Cis-acting* models. *SCD* was discussed to be involved in the fatty acid composition trait in pigs as before. It was demonstrated that *NPDC1* might participate in antiobesity effects of the sodium alginate in high-fat diet-induced obese mice [54]. Functional enrichment analysis revealed that 42 target DEGs were involved in the unsaturated fatty acid biosynthetic process, iron ion transport, electron transport chain, antigen processing and presentation of peptide antigen, etc. (Table S7). Of these, *CBR1*, *CD74*, and *SCD* were enriched in unsaturated fatty acid biosynthetic process term. The regulatory lncRNAs of *CBR1* and *CD74* were *MSTRG.30986* and *MSTRG.4275*, respectively. The *CD74* and *CBR1* expression of adipocyte is involved in fatty acid synthesis process and metabolic complications of obesity [55, 56]. It indicated that these lncRNAs would be involved in the fatness and fatty acid composition traits.

## 4. Conclusions

In this study, the lncRNA and gene profiles from back-fat tissue were investigated in the Tibetan and Yorkshire pigs. In total, 1,964 lncRNAs were identified through our computational pipeline. Evidence from lncRNAs *cis-* and *trans-*targeting analysis demonstrated that some lncRNAs functioned as regulators of fatness and fatty acid composition. Functional analysis revealed that 12 candidate genes contributed to fat acid composition and 10 genes are involved in the pig fatness trait. Association analysis also identified 34 and 57 genes correlated to fatness and fatty acid composition trait, respectively. These findings will help us decipher the fatness and fatty acid composition trait of pig and deserve to be further explored.

## Figures and Tables

**Table 1:** The DEGs within 100 kb upstream or downstream of differentially expressed lncRNAs.

LncRNA_id	LncRNA_Location	DEG_id	Gene symbol	Location	Regulatory type	Pearson correlation	Adjust value
MSTRG.29167	5:22252178-22272031	ENSSSCG00000000419	*RDH16*	5:22251753-22293249	*Cis*	0.9456	0.0212
MSTRG.34932	7:24097492-24098545	ENSSSCG00000023611	-	7:24089073-24159027	*Cis*	0.9801	0.0057
MSTRG.37120	8:28306560-28306822	ENSSSCG00000008769	-	8:28306016-28313150	*Cis*	0.9891	0.0042
MSTRG.7083	12:1562157-1567167	ENSSSCG00000039849	-	12:1561952-1563839	*Cis*	0.8160	0.0753
MSTRG.8034	12:38825046-38833501	ENSSSCG00000017694	*ACACA*	12:38581451-38875134	*Cis*	0.6736	0.1788
MSTRG.13970	14:106696815-106700176	ENSSSCG00000010491	-	14:106693255-106713440	*Cis*	0.9418	0.0221
MSTRG.20146	18:50320447-50325416	ENSSSCG00000031144	-	18:50319797-50343287	*Cis*	0.7237	0.1376
MSTRG.14097	14:111470803-111477982	ENSSSCG00000010554	*SCD*	14:111461157-111478031	*Cis & Trans*	0.9986	0.0075
MSTRG.42256	AEMK02000682.1:839454-840881	ENSSSCG00000034566	*NPDC1*	AEMK02000682.1:836624-843893	*Cis & Trans*	0.9982	0.0075

## Data Availability

All the RNA-seq data was collected in the NCBI database (SRP090525).

## Supplementary Materials

Supplementary Materials Figure S1: summary of RNA-Seq read alignment. Table S1: the list of differentially expressed genes between the Tibetan and Yorkshire group. Table S2: the annotation of identified lncRNAs with the gtf format. Table S3: the list of differentially expressed lncRNAs between the Tibetan and Yorkshire group. Table S4: the functional enrichment results of upregulated DEGs using Metascape. Table S5: the functional enrichment results of downregulated DEGs. Table S6: the *trans*-targeting gene prediction of lncRNAs. Table S7: functional enrichment analysis of the *trans*-targeting genes of lncRNAs.
