# Supplementary material for: Identification of lncRNAs and Genes Responsible for Fatness and Fatty Acid Composition Traits between the Tibetan and Yorkshire Pigs
Source: Int J Genomics. 2019 Jun 2;2019:5070975. doi: 10.1155/2019/5070975 (PMC6589220; doi:10.1155/2019/5070975)
Supplement: Supplementary Materials — Figure S1: summary of RNA-Seq read alignment. Table S1: the list of differentially expressed genes between the Tibetan and Yorkshire group. Table S2: the annotation of identified lncRNAs with the gtf format. Table S3: the list of differentially expressed lncRNAs between the Tibetan and Yorkshire group. Table S4: the functional enrichment results of upregulated DEGs using Metascape. Table S5: the functional enrichment results of downregulated DEGs. Table S6: the trans-targeting gene prediction of lncRNAs. Table S7: functional enrichment analysis of the trans-targeting genes of lncRNAs. [file 5070975.f1.pdf]

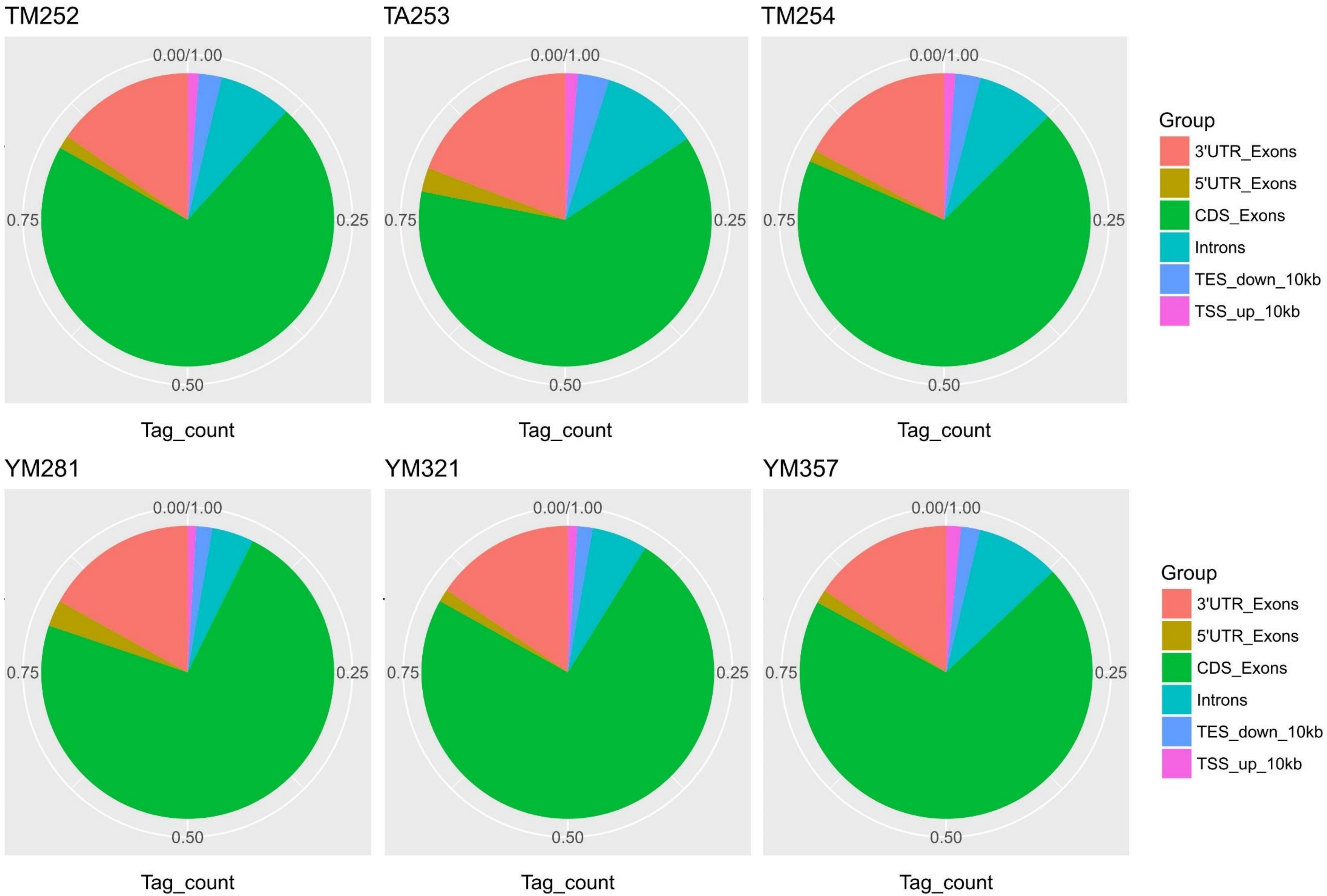

Figure S1. Summary of RNA-Seq reads alignment.

Table S1. The list of differentially expressed genes between Tibetan and Yorkshire group.

Row.names	baseMean	log2FoldChange	pvalue	padj	TA249	TA251	TA253	YA273	YA302	YA338
ENSSSCG00000035520	46420.29753	8.769719643	9.5E-245	1.4E-240	80830.84	86331.23	110722.5	176.3528	280.1581	180.7016
ENSSSCG00000018080	121773.56	-4.942325703	1.93E-76	1.45E-72	5119.065	9847.107	8049.157	259577.4	227106.4	220942.3
ENSSSCG00000038663	454.1424881	-5.554417844	9.97E-47	4.97E-43	6.731816	27.20195	22.92639	1075.055	808.6383	784.3019
ENSSSCG00000022945	662.5435487	4.229568887	6.26E-36	2.34E-32	1460.804	1416.445	897.1197	42.84277	61.54989	96.50035
ENSSSCG00000032343	151.905191	5.913989823	8.23E-28	2.46E-24	356.7862	202.0717	337.9151	6.974404	1.061205	6.622573
ENSSSCG00000036724	1373.218408	3.005722462	7.75E-25	1.93E-21	2581.171	2001.287	2744.189	213.2175	421.2984	278.1481
ENSSSCG00000017427	202.9954304	-4.578751222	5E-24	1.07E-20	20.19545	5.82899	22.92639	313.8482	548.643	306.5305
ENSSSCG00000018065	137039.0127	-2.614687632	1.31E-23	2.45E-20	39800.42	30269.95	45332.45	216037.2	302102.8	188691.3
ENSSSCG00000008348	520.4040016	3.097500677	3.77E-23	6.27E-20	1232.884	701.4218	861.2349	124.5429	120.9774	81.36304
ENSSSCG00000015810	842.3044576	3.016460347	2E-22	3E-19	1876.253	915.1514	1706.521	156.4259	202.6902	196.785
ENSSSCG00000034866	259.5243643	4.029614138	1.52E-21	2.07E-18	729.9212	287.5635	449.5566	34.87202	38.20338	17.02947
ENSSSCG00000035428	87.56714085	-5.878887223	2.44E-20	3.05E-17	4.80844	1.942997	1.993599	164.3967	212.241	140.0201
ENSSSCG00000025795	294.4686444	3.417740545	3.7E-20	4.26E-17	792.4309	383.7418	439.5886	63.76598	37.14218	50.14234
ENSSSCG00000006043	158.9556707	4.345885499	2.85E-19	3.04E-16	470.2654	295.3355	143.5391	14.94515	11.67326	17.97556
ENSSSCG00000000419	1229.616647	2.655037762	4.81E-18	4.8E-15	3061.053	1394.1	1911.862	350.7129	292.8926	367.0798
ENSSSCG00000024045	2335.574418	2.40296114	3.37E-17	3.15E-14	5096.946	2568.642	4119.773	795.0821	625.0498	807.9539
ENSSSCG00000011322	469.7290452	3.211437713	7.5E-17	6.6E-14	1255.964	642.1604	645.9262	121.5539	47.75423	105.0151
ENSSSCG00000012572	1268.660934	-3.236800615	8.32E-17	6.92E-14	351.9778	265.219	112.6384	2083.354	3316.266	1482.51
ENSSSCG00000039651	1444.957911	2.150193515	1.7E-16	1.34E-13	2391.718	1787.557	2896.7	555.9597	453.1346	584.6786
ENSSSCG00000026478	609.4242672	3.382413998	4.07E-16	3.04E-13	1585.823	1194.943	556.2142	97.64166	57.30507	164.6183
ENSSSCG00000005949	176.4899035	3.136295382	6.86E-16	4.89E-13	296.1999	233.1596	421.6462	26.90127	31.83615	49.19626
ENSSSCG00000037645	991.987344	2.341025362	2.23E-15	1.52E-12	1994.541	1441.704	1535.071	310.8592	226.0367	443.7124
ENSSSCG00000021176	148.8264304	2.956226683	7.24E-15	4.71E-12	261.5791	289.5065	240.2287	39.85374	20.1629	41.6276
ENSSSCG00000004588	182.7971564	2.930137804	9.03E-15	5.63E-12	369.2882	233.1596	366.8223	36.86471	59.42748	31.2207
ENSSSCG00000002452	13092.5564	2.024029771	2.02E-14	1.21E-11	26146.37	15470.14	21436.18	4122.869	5296.475	6083.307
ENSSSCG00000000132	71.98788083	9.612156333	2.52E-14	1.45E-11	156.7551	112.6938	162.4783	0	0	0
ENSSSCG00000035756	99.0442109	-4.942125746	2.92E-14	1.59E-11	1.923376	0.971498	15.94879	169.3784	141.1403	264.9029
ENSSSCG00000039798	501.3743265	3.164175535	2.98E-14	1.59E-11	1389.639	486.7207	830.3341	110.5941	50.93784	140.0201
ENSSSCG00000039222	370.4535373	3.101944524	3.51E-14	1.81E-11	1003.041	560.5545	427.627	71.73673	42.4482	117.3142
ENSSSCG00000029744	133.1811701	-3.369893949	7.29E-14	3.64E-11	26.92726	9.714984	33.89119	183.3272	264.2401	280.9863
ENSSSCG00000027941	93.88050364	-4.283500192	9.6E-14	4.63E-11	5.770128	3.885993	17.94239	260.0456	168.7316	106.9073
ENSSSCG00000000893	81.84932279	-3.574382408	1.14E-13	5.32E-11	10.57857	17.48697	9.967996	147.4588	113.5489	192.0546
ENSSSCG00000003914	4564.413805	2.45184368	1.17E-13	5.32E-11	9079.296	3861.706	10213.21	1296.243	1686.255	1249.774

ENSSSCG00000006247	71.81049541	-4.255005184	1.29E-13	5.52E-11	12.50194	1.942997	6.977597	161.4076	106.1205	141.9123
ENSSSCG00000015353	821.9110089	3.040380704	1.27E-13	5.52E-11	2272.469	1388.271	736.6349	177.3491	96.56966	260.1725
ENSSSCG00000006774	357.8207273	2.289624011	1.41E-13	5.84E-11	748.1932	490.6067	543.2558	110.5941	154.9359	99.3386
ENSSSCG00000013339	573.0239277	-2.707204025	2.99E-13	1.21E-10	121.1727	174.8697	160.4847	534.0401	1579.073	868.5032
ENSSSCG00000012459	415.28	-2.258034859	3.29E-13	1.29E-10	97.13048	199.1572	134.5679	650.6123	694.0281	716.184
ENSSSCG00000000133	2552.575002	1.702760405	4.99E-13	1.92E-10	3976.58	3282.693	4456.691	1200.594	1365.771	1033.121
ENSSSCG00000024752	1402.233684	2.293808946	5.66E-13	2.07E-10	2412.875	1846.818	2728.241	284.9542	675.9876	464.5262
ENSSSCG00000038569	253.4488025	-2.216534574	5.56E-13	2.07E-10	80.78179	112.6938	75.75677	475.2558	378.8502	397.3544
ENSSSCG00000038824	1302.466514	2.977764565	6.03E-13	2.15E-10	3398.605	1387.3	2148.103	233.1444	501.95	145.6966
ENSSSCG00000036352	280.7147691	2.248762022	6.63E-13	2.31E-10	565.4725	467.2907	358.8479	114.5795	80.65159	97.44644
ENSSSCG00000010772	215.8441926	4.048289113	1.5E-12	5.11E-10	671.2582	186.5277	363.8319	16.93784	6.36723	50.14234
ENSSSCG00000003558	460.7593991	2.00499046	1.6E-12	5.33E-10	897.2549	754.8542	561.1982	195.2833	159.1808	196.785
ENSSSCG00000006357	1160.672344	1.832465197	1.94E-12	6.16E-10	2372.484	1584.514	1480.247	521.0876	514.6845	491.0165
ENSSSCG00000032857	456.646398	2.593981718	1.9E-12	6.16E-10	976.1133	419.6873	954.934	168.382	90.20243	130.5593
ENSSSCG00000022236	4863.312093	1.831965783	2.15E-12	6.71E-10	10245.82	6368.172	6167.199	2274.652	1901.68	2222.346
ENSSSCG00000039556	299.6901004	3.307893487	2.56E-12	7.81E-10	345.246	467.2907	820.3661	70.74039	77.46797	17.02947
ENSSSCG00000014432	832.5386877	-1.827479893	2.75E-12	8.24E-10	390.4453	270.0765	437.595	1217.532	1345.608	1333.975
ENSSSCG00000017421	7723.306868	1.867485881	4.43E-12	1.3E-09	14126.23	8252.878	13993.07	2773.82	3338.551	3855.284
ENSSSCG00000008294	497.6292223	-2.023657069	5.72E-12	1.65E-09	162.5253	179.7272	247.2063	1010.292	715.2522	670.7721
ENSSSCG00000002825	6840.208308	2.254740593	6.44E-12	1.82E-09	13330.92	12665.42	7935.522	2908.327	1286.181	2914.878
ENSSSCG00000033909	1245.175304	1.790553478	6.71E-12	1.86E-09	2381.139	1883.735	1531.084	590.8317	462.6854	621.5758
ENSSSCG00000007748	616.037228	2.412864844	7.1E-12	1.9E-09	1613.712	650.9039	847.2797	150.4479	205.8738	228.0057
ENSSSCG00000039985	3973.305334	2.648911067	7.04E-12	1.9E-09	10209.28	5402.502	4950.107	1408.83	476.4811	1392.633
ENSSSCG00000003797	65.1064764	-4.406863655	7.28E-12	1.91E-09	2.885064	9.714984	4.983998	191.2979	123.0998	58.65708
ENSSSCG00000039270	179.8959933	4.03039774	8.48E-12	2.19E-09	609.7102	227.3306	180.4207	17.93418	4.24482	39.73544
ENSSSCG00000002375	368.2343408	2.86077176	9.83E-12	2.45E-09	412.5641	439.1173	1090.499	53.80255	92.32484	121.0985
ENSSSCG00000013056	2470.99349	-2.360631941	9.72E-12	2.45E-09	1251.156	432.3168	732.6477	3931.571	5175.497	3302.772
ENSSSCG00000018069	303196.6446	-1.549013968	1.18E-11	2.89E-09	153923.9	136499.4	172923.8	402663.2	543984.3	409185.2
ENSSSCG00000031023	129.7927773	-3.20620632	1.25E-11	3.02E-09	13.46363	42.74593	19.93599	167.3857	206.935	328.2904
ENSSSCG00000005278	263.3036247	2.645614503	1.3E-11	3.09E-09	303.8934	428.4308	629.9774	106.6088	45.63182	65.27965
ENSSSCG00000011241	650.508581	-2.238313432	1.62E-11	3.8E-09	270.2343	127.2663	285.0847	1347.056	943.4113	929.9985
ENSSSCG00000012118	501.6652066	2.504412179	1.65E-11	3.8E-09	1381.946	649.9324	527.307	154.4332	115.6714	180.7016
ENSSSCG00000017694	10414.03484	1.627397494	1.74E-11	3.95E-09	16316	13719.5	17169.87	3918.619	5273.128	6087.091
ENSSSCG00000012229	285.4310285	2.481150109	1.96E-11	4.38E-09	574.1277	558.6116	319.9727	109.5978	50.93784	99.3386
ENSSSCG00000018061	43894.79807	-1.982464399	2.12E-11	4.66E-09	14200.28	25655.33	13332.19	86535.42	56330.89	67314.67

ENSSSCG00000025133	994.1960119	2.125805949	2.91E-11	6.32E-09	2281.124	1275.577	1296.836	387.5776	255.7504	468.3105
ENSSSCG00000039926	661.0263125	2.561894696	3.59E-11	7.68E-09	1886.832	740.2817	764.5453	261.042	177.2212	136.2358
ENSSSCG00000033590	122.5412195	2.342004197	3.74E-11	7.89E-09	196.1843	221.5016	196.3695	41.84643	42.4482	36.89719
ENSSSCG00000025644	353.9653346	3.139452703	3.98E-11	8.28E-09	766.4653	811.2011	329.9407	111.5905	22.28531	82.30913
ENSSSCG00000040981	537.30696	2.038285106	5.09E-11	1.04E-08	1143.447	807.3151	641.939	270.0091	171.9152	189.2164
ENSSSCG00000009228	140.2266285	-2.572185377	5.92E-11	1.2E-08	49.04609	27.20195	44.85598	311.8555	223.9143	184.486
ENSSSCG00000017548	936.3906749	-2.650607191	9.45E-11	1.89E-08	244.2687	340.0244	187.3983	1066.088	2913.008	867.5571
ENSSSCG00000000648	90.04702912	2.91308926	1.17E-10	2.3E-08	185.6058	172.9267	118.6192	16.93784	15.91808	30.27462
ENSSSCG00000001455	1397.801629	2.756883975	1.3E-10	2.52E-08	3372.64	998.7003	2934.578	167.3857	458.4406	455.0654
ENSSSCG00000009668	1885.848882	1.951394944	1.53E-10	2.94E-08	3146.643	3573.171	2270.71	480.2376	858.5149	985.8173
ENSSSCG00000009412	2651.602804	2.170858629	1.71E-10	3.25E-08	6006.703	4724.396	2287.655	937.5592	720.5582	1232.745
ENSSSCG00000033457	128.8823028	2.47079065	1.93E-10	3.62E-08	170.2188	282.706	202.3503	43.83911	29.71374	44.46585
ENSSSCG00000011673	94.06235372	2.922304605	2.07E-10	3.83E-08	141.3681	186.5277	170.4527	25.90493	29.71374	10.4069
ENSSSCG00000001487	778.2611772	1.987867751	2.11E-10	3.85E-08	957.8412	1481.535	1289.859	195.2833	396.8907	348.1581
ENSSSCG00000008854	814.6718353	-1.682227633	2.16E-10	3.85E-08	343.3226	358.4829	459.5246	1530.384	1164.142	1032.175
ENSSSCG00000033465	131.047676	2.828320613	2.14E-10	3.85E-08	344.2843	160.2972	184.4079	27.89762	46.69302	22.70597
ENSSSCG00000029805	1710.655239	-1.636284628	2.21E-10	3.89E-08	768.3887	1062.819	666.8589	2993.016	2405.752	2367.097
ENSSSCG00000031648	75.07700452	3.428962311	2.34E-10	4.06E-08	109.6324	160.2972	142.5423	7.970748	6.36723	23.65205
ENSSSCG00000034220	83.83460918	3.933929474	2.9E-10	4.99E-08	283.698	112.6938	75.75677	18.93053	5.306025	6.622573
ENSSSCG00000038877	41.04714417	-6.380723399	2.97E-10	5.05E-08	1.923376	0.971498	0	73.72942	55.18266	114.4759
ENSSSCG00000001453	10331.50096	1.580410934	3.06E-10	5.14E-08	17205.56	11422.88	17826.76	4450.666	5152.151	5930.987
ENSSSCG00000023630	744.1633908	2.026679838	3.39E-10	5.63E-08	1686.801	1208.544	689.7853	299.8994	279.0969	300.854
ENSSSCG00000014361	778.1747247	2.072173246	3.47E-10	5.71E-08	1471.383	731.5383	1568.963	229.159	375.6666	292.3393
ENSSSCG00000030209	4625.725636	2.342691463	4.89E-10	7.96E-08	8965.817	3120.453	11097.37	1356.023	1952.617	1262.073
ENSSSCG00000026169	626.4569565	1.557743283	5.07E-10	8.16E-08	1072.282	798.5716	934.998	329.7897	284.403	338.6973
ENSSSCG00000005467	1989.371182	-1.610442086	5.67E-10	8.9E-08	738.5764	1293.064	913.0684	2857.513	3092.352	3041.653
ENSSSCG00000006140	105.0788525	3.309191448	5.68E-10	8.9E-08	205.8012	109.7793	257.1743	35.86836	9.550846	12.29906
ENSSSCG00000006381	250.47641	2.071558816	5.77E-10	8.9E-08	403.9089	500.3217	310.0047	115.5758	68.97833	104.069
ENSSSCG00000035218	266.6899748	2.282907221	5.74E-10	8.9E-08	377.9434	502.2646	447.563	62.76964	65.79471	143.8044
ENSSSCG00000003784	81.27222109	-2.899148742	6.24E-10	9.54E-08	12.50194	27.20195	17.94239	105.6124	164.4868	159.8878
ENSSSCG00000009578	219.4761117	-2.049456044	6.55E-10	9.91E-08	90.39867	59.2614	106.6576	359.68	303.5047	397.3544
ENSSSCG00000008239	926.6646484	2.022548463	7.42E-10	1.11E-07	2221.499	1159.969	1080.531	441.3802	270.6073	386.0014
ENSSSCG00000012571	85.11185593	-2.645047415	8.16E-10	1.21E-07	25.96558	15.54397	28.90719	132.5137	150.6911	157.0496
ENSSSCG00000013556	134.271167	2.771122643	8.35E-10	1.22E-07	301.0083	262.3046	139.5519	47.82449	18.04049	36.89719
ENSSSCG00000037324	541.0689002	2.061928432	9.78E-10	1.42E-07	920.3354	536.2671	1162.268	181.3345	275.9133	170.2947

ENSSSCG00000015246	50.30530524	3.99736839	9.87E-10	1.42E-07	128.8662	83.54886	71.76957	9.963435	1.061205	6.622573
ENSSSCG00000039103	183202.6447	-1.236758801	1.17E-09	1.66E-07	114717.8	105371.6	107381.2	230197.2	272734	268814
ENSSSCG00000007754	1335.895484	1.822385077	1.18E-09	1.67E-07	2515.776	1904.137	1829.127	591.828	378.8502	795.6549
ENSSSCG00000009646	201.6176686	-6.462771822	1.21E-09	1.7E-07	9.61688	2.914495	0.9968	813.0163	200.5678	182.5938
ENSSSCG00000014441	2654.555018	1.726946818	1.23E-09	1.71E-07	5196.962	3636.318	3399.087	1561.27	865.9433	1267.75
ENSSSCG00000006379	445.9212164	2.155063966	1.28E-09	1.76E-07	951.1094	803.4291	430.6174	202.2577	109.3041	178.8095
ENSSSCG00000001535	66.77383359	4.021418754	1.48E-09	2.01E-07	144.2532	85.49185	147.5263	0	14.85687	8.514737
ENSSSCG00000006243	197.09464	-2.22948432	1.72E-09	2.32E-07	39.42921	73.83387	94.69596	277.9798	334.2796	362.3494
ENSSSCG00000002001	281.7922158	2.15485588	1.76E-09	2.35E-07	570.281	274.934	535.2814	88.67457	132.6506	88.9317
ENSSSCG00000013553	322.6207115	1.750899119	1.88E-09	2.49E-07	599.1316	495.4642	397.723	168.382	136.8955	138.128
ENSSSCG00000037425	75.82730535	3.922076684	1.9E-09	2.5E-07	282.7363	59.2614	84.72797	4.981717	13.79567	9.460819
ENSSSCG00000008937	148.5625518	3.677413339	2.22E-09	2.89E-07	509.6946	179.7272	137.5583	31.88299	3.183615	29.32854
ENSSSCG00000005055	3578.416474	1.739425645	2.59E-09	3.35E-07	7208.813	5376.272	3937.358	2083.354	1211.896	1652.805
ENSSSCG00000005023	207.2839289	2.166031491	2.9E-09	3.71E-07	227.92	441.0603	347.8831	80.70382	91.26364	54.87275
ENSSSCG00000009789	3365.551382	-1.363075781	3.27E-09	4.15E-07	2038.778	1822.531	1791.249	4042.165	5789.935	4708.65
ENSSSCG00000025271	229.965954	2.640680403	5.26E-09	6.62E-07	716.4575	188.4707	284.0879	58.78426	74.28436	57.711
ENSSSCG00000015559	718.2592933	1.952586862	5.36E-09	6.69E-07	1224.229	1185.228	1015.739	198.2724	219.6695	466.4184
ENSSSCG00000026140	1131.957522	1.524678851	6.2E-09	7.61E-07	2001.273	1772.984	1265.936	531.0511	541.2146	679.2868
ENSSSCG00000027196	392.3966356	1.614442612	6.15E-09	7.61E-07	631.829	664.5049	478.4638	223.1809	171.9152	184.486
ENSSSCG00000016832	58.26341457	2.878168109	6.41E-09	7.8E-07	83.66685	121.4373	102.6704	15.9415	11.67326	14.19123
ENSSSCG00000013237	945.9656485	1.745068651	6.7E-09	8.08E-07	2089.748	987.0423	1294.843	405.5118	443.5837	455.0654
ENSSSCG00000003231	418.3636517	2.432837879	7.28E-09	8.71E-07	1246.348	325.4519	546.2462	139.4881	105.0593	147.5888
ENSSSCG00000000892	36.31718198	-6.785151295	8.3E-09	9.87E-07	0	0	1.993599	63.76598	59.42748	92.71603
ENSSSCG00000029281	558.6530184	2.70273793	8.53E-09	1.01E-06	444.2998	1874.02	587.115	189.3053	172.9764	84.20129
ENSSSCG00000000194	2080.544034	2.165512578	1.03E-08	1.2E-06	5153.686	1366.898	3687.162	724.3417	894.5959	656.5808
ENSSSCG00000006932	59.24383467	-3.190816183	1.08E-08	1.25E-06	8.655192	18.45847	7.974397	93.65629	75.34556	151.3731
ENSSSCG00000002764	89.0345639	3.468454245	1.12E-08	1.29E-06	164.4486	211.7866	113.6352	30.88665	10.61205	2.838246
ENSSSCG00000003650	131.405531	-2.133750013	1.16E-08	1.31E-06	61.54803	35.94544	48.84318	213.2175	177.2212	251.6578
ENSSSCG00000011999	159.948451	2.789950876	1.16E-08	1.31E-06	352.9395	315.737	169.4559	47.82449	60.48869	13.24515
ENSSSCG00000003470	39.08348064	4.987736927	1.27E-08	1.42E-06	43.27596	59.2614	124.6	0.996343	6.36723	0
ENSSSCG00000005481	2149.834348	-1.632089032	1.49E-08	1.65E-06	841.477	820.9161	1484.235	3403.509	2657.258	3691.612
ENSSSCG00000020785	378.9578889	-1.693768486	1.49E-08	1.65E-06	140.4064	157.3827	239.2319	656.5903	538.031	542.1049
ENSSSCG00000006003	3018.49983	-1.488551871	1.67E-08	1.84E-06	1436.762	2133.41	1188.185	4098.957	4701.138	4552.546
ENSSSCG00000012787	720.8247423	1.485434615	1.83E-08	1.98E-06	1035.738	889.8925	1260.952	333.7751	457.3794	347.2121
ENSSSCG00000013273	517.2454106	2.657438695	1.82E-08	1.98E-06	1542.548	307.965	828.3405	74.72576	172.9764	176.9173

ENSSSCG00000029458	482.6583717	-1.418111182	1.91E-08	2.06E-06	260.6174	238.9886	289.0719	648.6196	669.6204	789.0323
ENSSSCG00000017296	2867.858777	1.950944862	1.94E-08	2.08E-06	7642.534	2792.086	3236.608	1261.371	1021.94	1252.612
ENSSSCG00000039815	328.8440893	-1.79523512	2.05E-08	2.17E-06	131.7513	173.8982	135.5647	531.0511	652.6411	348.1581
ENSSSCG00000006171	146.9627305	-2.262830368	2.16E-08	2.28E-06	62.50972	45.66042	43.85918	360.6763	166.6092	202.4615
ENSSSCG00000026890	105.4481721	2.536679498	2.31E-08	2.41E-06	274.0811	136.9813	128.5872	40.85008	27.59133	24.59813
ENSSSCG00000027183	54.4375007	3.420422123	2.32E-08	2.41E-06	88.47529	77.71987	132.5743	17.93418	4.24482	5.676491
ENSSSCG00000003773	385.8210828	-1.735112025	2.36E-08	2.44E-06	175.9889	193.3282	165.4687	797.0748	410.6864	572.3795
ENSSSCG00000027030	105.3663906	2.364719804	2.49E-08	2.55E-06	221.1882	174.8697	133.5711	34.87202	22.28531	45.41193
ENSSSCG00000029771	261.0796446	-1.890241585	2.7E-08	2.75E-06	88.47529	152.5252	91.70556	390.5666	516.8069	326.3983
ENSSSCG00000008550	2849.052011	-1.233196731	2.73E-08	2.76E-06	1548.318	1967.284	1585.908	4132.833	3627.199	4232.77
ENSSSCG00000015136	397.4137883	1.835797524	3.33E-08	3.34E-06	602.0167	809.2581	451.5502	230.1553	123.0998	168.4026
ENSSSCG00000018092	190211.6984	-1.223782428	3.87E-08	3.86E-06	105253.9	110268.9	126627.4	221033.8	303305.1	274781
ENSSSCG00000033908	31.47608649	5.167395145	4.42E-08	4.38E-06	72.1266	46.63192	64.79197	0	5.306025	0
ENSSSCG00000037241	486.1033564	1.81566965	4.61E-08	4.54E-06	1125.175	586.785	559.2046	193.2906	261.0564	191.1085
ENSSSCG00000037449	2932.093559	1.930607951	4.75E-08	4.65E-06	7258.821	2167.413	4510.518	1138.821	1181.121	1335.868
ENSSSCG00000039849	19.80169608	7.749651607	4.98E-08	4.84E-06	27.88895	30.11645	60.80478	0	0	0
ENSSSCG00000016175	299.3659696	-1.734777611	5.07E-08	4.89E-06	147.1383	164.1832	103.6672	539.0218	341.708	500.4773
ENSSSCG00000017738	182.2232892	2.157787736	5.31E-08	5.1E-06	385.6369	195.2712	311.9983	72.73307	87.01882	40.68152
ENSSSCG00000001633	19.34843229	7.716740121	5.4E-08	5.15E-06	51.93115	24.28746	39.87198	0	0	0
ENSSSCG00000017673	448.2148746	-1.51021583	5.49E-08	5.2E-06	193.2993	302.136	203.3471	662.5684	642.0291	685.9094
ENSSSCG00000009492	1616.377967	-1.200752901	5.7E-08	5.37E-06	850.1322	1003.558	1086.512	2346.389	2207.307	2204.371
ENSSSCG00000006588	95.21748081	3.53507655	6.39E-08	5.98E-06	318.3187	31.08795	176.4335	13.94881	20.1629	11.35298
ENSSSCG00000035836	297.2260811	2.417399991	8.38E-08	7.79E-06	563.5492	172.9267	765.5421	86.68188	119.9162	74.74047
ENSSSCG00000028592	2076.061221	1.224097609	8.43E-08	7.79E-06	3128.371	2491.893	3102.04	1214.543	1405.036	1114.484
ENSSSCG00000021041	715.5656586	1.504905138	8.68E-08	7.97E-06	874.1744	968.5839	1331.724	344.7348	464.8078	309.3688
ENSSSCG00000032241	3832.035682	1.971163173	9E-08	8.22E-06	8313.792	6793.688	3212.685	1407.833	961.4518	2302.763
ENSSSCG00000040885	221.7559815	1.820427105	9.14E-08	8.29E-06	292.3531	433.2883	311.0015	87.67823	130.5282	75.68655
ENSSSCG00000000261	1075.907625	1.65019579	9.4E-08	8.48E-06	2112.828	1573.827	1209.118	715.3746	387.3399	456.9576
ENSSSCG00000006245	8455.803031	1.39194544	9.69E-08	8.69E-06	10619.92	11744.44	14371.86	3371.626	5804.792	4822.179
ENSSSCG00000010554	547070.9886	1.82546035	1.13E-07	1.01E-05	1149930	514409.3	895754	171724.8	363941.3	186666.7
ENSSSCG00000029752	90.87398995	3.51361332	1.19E-07	1.05E-05	330.8207	34.97394	135.5647	21.91956	10.61205	11.35298
ENSSSCG00000034871	88.37806962	-9.94239958	1.22E-07	1.07E-05	0	0	0	286.9469	219.6695	23.65205
ENSSSCG00000000363	400.6691663	1.842763663	1.25E-07	1.1E-05	403.9089	539.1816	936.9916	146.4625	165.548	211.9223
ENSSSCG00000038970	49.90760314	-2.823185098	1.29E-07	1.13E-05	10.57857	15.54397	10.9648	91.6636	64.73351	105.9612
ENSSSCG00000034156	2511.10732	-1.296688496	1.31E-07	1.13E-05	1240.577	1331.924	1786.265	3753.226	3959.356	2995.295

ENSSSCG00000009281	552.2950647	-1.27062242	1.42E-07	1.22E-05	352.9395	303.1075	314.9887	836.9285	720.5582	785.248
ENSSSCG00000031144	334.7814142	2.535750876	1.52E-07	1.3E-05	853.9789	147.6677	711.7149	96.64532	78.52918	120.1524
ENSSSCG00000025085	403.4852621	-1.854633932	1.58E-07	1.35E-05	239.4603	166.1262	118.6192	699.4331	798.0262	399.2466
ENSSSCG00000000135	62.21881657	-2.633308174	1.62E-07	1.36E-05	19.23376	10.68648	21.92959	79.70748	109.3041	132.4515
ENSSSCG00000040581	504.3706008	-2.069493702	1.62E-07	1.36E-05	112.5175	136.0098	333.9279	993.3544	576.2344	874.1797
ENSSSCG00000008019	891.0242836	1.486119408	1.93E-07	1.62E-05	1422.337	861.719	1655.684	447.3582	494.5216	464.5262
ENSSSCG00000013366	3174.307243	1.180142479	2.02E-07	1.68E-05	5280.629	3827.704	4105.818	2005.639	2006.739	1819.315
ENSSSCG00000001572	220.7248637	1.810029253	2.09E-07	1.73E-05	388.5219	375.9699	266.1455	129.5247	65.79471	98.39252
ENSSSCG00000011090	1883.634703	-1.413032618	2.13E-07	1.75E-05	769.3504	1207.572	1108.441	2348.382	3483.936	2384.126
ENSSSCG00000012050	2078.480406	2.292288874	2.16E-07	1.76E-05	4802.67	922.9234	4631.131	804.0492	512.5621	797.547
ENSSSCG00000025034	3763.569451	1.355059003	2.16E-07	1.76E-05	7106.874	4879.836	4248.36	2212.879	1788.131	2345.337
ENSSSCG00000027266	2409.829241	3.754474096	2.24E-07	1.81E-05	7465.584	2293.708	3702.114	430.4204	448.8897	118.2602
ENSSSCG00000021220	1348.891479	1.804724414	2.29E-07	1.85E-05	2346.519	981.2133	2964.482	635.6671	635.6618	529.8059
ENSSSCG00000022849	276.2239056	-1.772188042	2.88E-07	2.3E-05	82.70517	169.0407	123.6032	544.0035	384.1562	353.8346
ENSSSCG00000034192	336.7774933	1.89127632	2.88E-07	2.3E-05	429.8745	321.566	840.3021	136.4991	131.5894	160.8339
ENSSSCG00000015930	74.82512892	2.8828704	2.9E-07	2.3E-05	179.8357	151.5537	63.79518	23.91224	22.28531	7.568655
ENSSSCG00000009322	1334.647467	-1.579929772	2.93E-07	2.31E-05	681.8368	775.2557	550.2334	1842.239	1302.099	2856.221
ENSSSCG00000029521	591.6270527	1.527487237	3.04E-07	2.38E-05	915.5269	598.443	1121.4	292.925	356.5649	264.9029
ENSSSCG00000004291	493.9375151	2.125909146	3.15E-07	2.45E-05	1133.83	928.7524	348.8799	157.4223	124.161	270.5794
ENSSSCG00000015009	1131.126588	-1.473997178	3.65E-07	2.83E-05	472.1888	863.662	460.5214	1608.098	1744.621	1637.668
ENSSSCG00000039761	83.22862537	2.265407438	3.73E-07	2.88E-05	184.6441	97.14984	131.5775	34.87202	26.53013	24.59813
ENSSSCG00000012536	64.89110288	-2.414979652	3.84E-07	2.95E-05	15.38701	29.14495	16.94559	121.5539	96.56966	109.7455
ENSSSCG00000017873	666.282408	1.305276619	4.17E-07	3.18E-05	1120.366	788.8567	936.9916	417.4679	330.0348	403.977
ENSSSCG00000007133	483.7032591	1.314284583	4.48E-07	3.4E-05	562.5875	755.8257	751.5869	291.9286	253.628	286.6628
ENSSSCG00000031220	15.15976263	-7.398093373	4.58E-07	3.46E-05	0	0	0	23.91224	42.4482	24.59813
ENSSSCG00000017956	1015.106093	1.914996134	4.6E-07	3.46E-05	2680.224	1228.945	905.0941	490.201	287.5866	498.5852
ENSSSCG00000006590	110.0033736	3.014734872	5.23E-07	3.92E-05	383.7135	64.11889	139.5519	38.8574	14.85687	18.92164
ENSSSCG00000015595	289.0199611	2.143976853	5.37E-07	4E-05	454.8784	621.7589	337.9151	108.6014	43.50941	167.4565
ENSSSCG00000011714	262.4635245	1.637527773	5.4E-07	4E-05	397.1771	337.1099	457.531	175.3565	99.75328	107.8533
ENSSSCG00000016328	21.36196405	5.383268137	5.92E-07	4.34E-05	36.54414	41.77443	46.84958	0.996343	1.061205	0.946082
ENSSSCG00000040535	640.4118589	1.93597936	5.9E-07	4.34E-05	1000.155	588.728	1457.321	141.4808	367.177	287.6089
ENSSSCG00000010253	4174.522957	1.343567686	7.32E-07	5.35E-05	6048.056	4258.077	7661.402	2236.791	2021.596	2821.216
ENSSSCG00000023362	309.2545939	1.731492889	7.77E-07	5.65E-05	577.9745	269.105	579.1406	134.5064	127.3446	167.4565
ENSSSCG00000015512	122.3028487	-2.336215343	8.19E-07	5.92E-05	21.15714	56.3469	43.85918	289.9359	112.4877	210.0302
ENSSSCG00000008984	337.9031832	2.12693956	9.43E-07	6.79E-05	726.0744	336.1384	587.115	124.5429	200.5678	52.98059

ENSSSCG00000016469	29.68194272	4.869519319	9.53E-07	6.82E-05	47.12271	15.54397	109.648	1.992687	0	3.784328
ENSSSCG00000004075	40.02605215	3.054891198	9.61E-07	6.84E-05	66.35647	93.26384	54.82398	12.95247	4.24482	8.514737
ENSSSCG00000027144	120.5310836	-1.862506943	9.64E-07	6.84E-05	69.24153	37.88844	48.84318	200.265	164.4868	202.4615
ENSSSCG00000011250	10816.13635	-1.02741421	1.02E-06	7.22E-05	6787.594	7648.607	6922.773	13947.81	16355.29	13234.74
ENSSSCG00000036801	561.4843349	5.336071067	1.04E-06	7.28E-05	1966.652	452.7182	868.2125	2.98903	24.40772	53.92667
ENSSSCG00000017991	161.7680378	1.79406258	1.05E-06	7.36E-05	307.7402	186.5277	259.1679	95.64897	56.24387	65.27965
ENSSSCG00000010814	113.600338	-5.946675475	1.1E-06	7.64E-05	0	1.942997	8.971197	203.2541	96.56966	370.8641
ENSSSCG00000026850	1660.664935	1.260806025	1.11E-06	7.64E-05	1855.096	2604.587	2570.746	796.0784	944.4725	1193.009
ENSSSCG00000036213	807.4950173	-1.328441244	1.1E-06	7.64E-05	352.9395	603.3005	423.6398	1152.769	1047.409	1264.912
ENSSSCG00000008434	369.9231477	1.459125423	1.11E-06	7.65E-05	640.4842	476.0342	511.3582	180.3382	154.9359	256.3882
ENSSSCG00000003434	1891.856277	1.109635247	1.17E-06	7.97E-05	2634.063	2216.959	2905.671	1067.084	1233.12	1294.24
ENSSSCG00000017086	552.2732126	-2.413723591	1.22E-06	8.32E-05	268.3109	150.5822	104.664	243.1078	1038.92	1508.055
ENSSSCG00000010073	246.9012264	2.233924394	1.26E-06	8.52E-05	476.0355	160.2972	585.1214	82.69651	125.2222	52.0345
ENSSSCG00000035828	157.8629261	2.138355734	1.29E-06	8.69E-05	334.6674	162.2402	275.1167	88.67457	29.71374	56.76491
ENSSSCG00000000713	44.56638572	3.113033694	1.3E-06	8.7E-05	38.46752	115.6083	85.72477	5.978061	7.428436	14.19123
ENSSSCG00000013632	49.18301111	3.233164019	1.31E-06	8.78E-05	122.1344	66.06189	78.74717	11.95612	1.061205	15.13731
ENSSSCG00000032337	13.82141527	7.231423231	1.33E-06	8.86E-05	37.50583	16.51547	28.90719	0	0	0
ENSSSCG00000001552	30.65173021	-4.706676335	1.36E-06	9E-05	4.80844	1.942997	0	31.88299	22.28531	122.9906
ENSSSCG00000034761	72.33016727	-2.367874735	1.37E-06	9.06E-05	18.27207	26.23046	25.91679	156.4259	139.0179	68.1179
ENSSSCG00000012525	28.69802616	-4.380045985	1.48E-06	9.7E-05	0	0.971498	6.977597	57.78792	74.28436	32.16678
ENSSSCG00000038429	73.3908386	2.740669984	1.53E-06	0.0001	225.9967	89.37785	67.78237	13.94881	14.85687	28.38246
ENSSSCG00000002456	179.9731552	-2.92471508	1.61E-06	0.000105	10.57857	99.09283	15.94879	225.1736	449.951	279.0942
ENSSSCG00000012448	1606.521023	-1.040253014	1.62E-06	0.000105	1042.47	1079.335	1031.688	1934.899	2372.855	2177.881
ENSSSCG00000005688	318.1619935	1.582395793	1.68E-06	0.000108	534.6985	485.7492	410.6814	231.1517	119.9162	126.775
ENSSSCG00000009612	132.9990596	1.733144182	1.68E-06	0.000108	257.7324	162.2402	193.3791	58.78426	70.03954	55.81883
ENSSSCG00000033262	21597.92159	1.436372839	1.76E-06	0.000112	35772.87	17586.06	41265.51	11239.75	11008.94	12714.39
ENSSSCG00000040629	611.4534595	1.446921518	1.76E-06	0.000112	1128.06	605.2435	950.9468	399.5337	272.7297	312.207
ENSSSCG00000027275	23.37948807	-5.54038091	1.78E-06	0.000113	0	0	2.990399	72.73307	45.63182	18.92164
ENSSSCG00000038228	23.38488057	-5.546868798	1.89E-06	0.00012	0.961688	0	1.993599	11.95612	65.79471	59.60316
ENSSSCG00000010698	430.961664	-1.936090727	1.91E-06	0.00012	239.4603	68.00488	228.2671	847.8883	668.5592	533.5902
ENSSSCG00000021418	8972.601365	1.528663368	1.94E-06	0.000122	16120.78	6936.498	16921.67	3733.299	5182.926	4940.44
ENSSSCG00000017178	237.0826966	2.079385074	2.05E-06	0.000128	620.2887	193.3282	336.9183	75.7221	64.73351	131.5054
ENSSSCG00000029944	320699.5743	1.52933649	2.34E-06	0.000145	576775.2	254556.9	597771.8	120949.1	188044.5	186100
ENSSSCG00000009240	74.98865363	-2.279215814	2.36E-06	0.000146	40.39089	18.45847	17.94239	95.64897	142.2015	135.2897
ENSSSCG00000000843	2614.054872	1.305780513	2.43E-06	0.00015	4221.81	2937.811	4007.134	1479.57	1954.74	1083.264

ENSSSCG00000039009	93.24694167	2.175234901	2.46E-06	0.000151	121.1727	143.7818	193.3791	36.86471	16.97928	47.3041
ENSSSCG00000001457	2807.663318	1.631363484	2.49E-06	0.000152	4298.745	2121.752	6314.726	1067.084	1597.114	1446.559
ENSSSCG00000032251	520.1297805	1.300743668	2.57E-06	0.000157	855.9023	713.0798	650.9101	237.1297	292.8926	370.8641
ENSSSCG00000011884	1069.151798	1.256048729	2.61E-06	0.000158	1863.751	1558.283	1099.47	593.8207	712.0686	587.5169
ENSSSCG00000015830	14.37953967	7.288198736	2.65E-06	0.00016	34.62077	6.800488	44.85598	0	0	0
ENSSSCG00000027565	475.2855897	1.3090131	2.72E-06	0.000164	829.9367	617.873	584.1246	262.0383	231.3427	326.3983
ENSSSCG00000002697	636.5104425	1.403631235	2.76E-06	0.000165	1106.903	753.8827	911.0748	301.8921	268.4849	476.8253
ENSSSCG00000029163	846.7466601	1.740128576	2.78E-06	0.000166	1561.781	1682.635	665.8621	464.2961	236.6487	469.2566
ENSSSCG00000000203	45.84602464	3.283821234	2.8E-06	0.000166	126.9428	50.51791	71.76957	6.974404	16.97928	1.892164
ENSSSCG00000000171	366.1344709	1.48608138	2.94E-06	0.000174	732.8062	393.4568	492.419	197.276	222.8531	157.9957
ENSSSCG00000000773	125.1247204	-1.861325496	3.02E-06	0.000178	67.31816	40.80293	53.82718	213.2175	244.0772	131.5054
ENSSSCG00000001866	1424.988086	-1.247846627	3.03E-06	0.000178	675.105	1140.539	717.6957	2028.555	2139.389	1848.644
ENSSSCG00000016831	245.0330254	-1.85413196	3.14E-06	0.000184	105.7857	138.9243	73.76317	588.839	331.096	231.7901
ENSSSCG00000025588	160.057897	2.26755958	3.19E-06	0.000186	424.1044	98.12133	273.1231	49.81717	41.387	73.79439
ENSSSCG00000012638	80.52864668	-2.208680507	3.21E-06	0.000186	18.27207	41.77443	25.91679	171.3711	93.38605	132.4515
ENSSSCG00000017192	155.8504737	1.805137724	3.26E-06	0.000189	175.9889	193.3282	357.8511	60.77695	65.79471	81.36304
ENSSSCG00000034469	46.50514096	3.087323197	3.31E-06	0.000191	89.43698	117.5513	42.86238	8.967091	3.183615	17.02947
ENSSSCG00000029902	103.7253026	2.154667448	3.33E-06	0.000191	177.9123	135.0383	195.3727	16.93784	40.32579	56.76491
ENSSSCG00000039311	453.496771	1.530813725	3.36E-06	0.000192	877.0594	447.8607	696.7629	240.1188	168.7316	290.4471
ENSSSCG00000008769	321.2778349	5.255022734	3.39E-06	0.000193	1353.095	304.079	221.2895	35.86836	9.550846	3.784328
ENSSSCG00000040793	16397.98798	1.197912961	3.44E-06	0.000195	26101.17	17260.61	25158.23	8119.203	9498.847	12249.87
ENSSSCG00000040826	40.03477326	3.843949162	3.48E-06	0.000197	125.0194	75.77687	23.92319	6.974404	0	8.514737
ENSSSCG00000011501	4756.834881	-1.165502606	3.66E-06	0.000205	2411.913	3827.704	2560.778	6650.593	7229.99	5860.031
ENSSSCG00000013046	2634.370949	1.8551734	3.65E-06	0.000205	5741.277	1360.098	5282.041	979.4056	1270.262	1173.142
ENSSSCG00000017797	450.8843259	-1.295114084	3.67E-06	0.000205	305.8168	234.1311	243.2191	785.1187	617.6214	519.399
ENSSSCG00000012001	1855.254787	1.431289131	3.88E-06	0.000216	3063.938	2784.314	2271.706	749.2503	1519.646	742.6743
ENSSSCG00000001770	5997.560082	1.108666077	3.97E-06	0.00022	9867.88	7619.462	7097.213	3265.018	4495.265	3640.523
ENSSSCG00000025741	107.146831	1.988524819	3.98E-06	0.00022	216.3798	121.4373	175.4367	45.8318	57.30507	26.49029
ENSSSCG00000014919	748.1553677	1.111094442	4.05E-06	0.000223	1015.542	982.1848	1070.563	399.5337	543.337	477.7714
ENSSSCG00000010385	896.8914061	-1.121608841	4.19E-06	0.00023	617.4037	521.6946	555.2174	1375.95	1306.343	1004.739
ENSSSCG00000037762	42.57449436	-2.644660217	4.29E-06	0.000235	6.731816	15.54397	12.95839	56.79158	84.89641	78.5248
ENSSSCG00000003524	1874.224671	1.275562195	4.41E-06	0.00024	3069.708	2861.063	2027.49	1413.811	828.8012	1044.474
ENSSSCG00000006099	17.36070656	6.583248142	4.52E-06	0.000245	24.0422	26.23046	52.83038	0	1.061205	0
ENSSSCG00000002306	2715.675603	1.351187032	4.67E-06	0.000252	4676.689	2275.249	4753.737	1487.541	1661.847	1438.991
ENSSSCG00000002919	536.6241853	1.993994889	4.71E-06	0.000253	1586.785	490.6067	496.4062	266.0237	135.8343	244.0891

ENSSSCG00000012278	209.4396449	1.460056306	4.75E-06	0.000255	331.7823	348.7679	241.2255	121.5539	91.26364	122.0446
ENSSSCG00000037322	13.98395223	-7.282694178	4.77E-06	0.000255	0	0	0	62.76964	11.67326	9.460819
ENSSSCG00000005490	93.07015794	2.065964489	4.9E-06	0.000261	161.5636	178.7557	110.6448	44.83546	19.10169	43.51977
ENSSSCG00000015799	140.434213	1.800204325	4.95E-06	0.000263	304.8551	192.3567	157.4943	47.82449	61.54989	78.5248
ENSSSCG00000000018	8199.754446	1.131733161	5.11E-06	0.00027	11344.07	10006.43	12431.09	3771.16	6047.808	5597.967
ENSSSCG00000013901	808.8905947	1.531191638	5.17E-06	0.000273	1659.873	1058.933	887.1517	565.9231	264.2401	417.2221
ENSSSCG00000012277	533.687583	1.54925286	5.24E-06	0.000275	978.0367	418.7158	989.822	248.0895	303.5047	263.9569
ENSSSCG00000016423	21.02965885	-4.35803377	5.32E-06	0.000279	0	3.885993	1.993599	53.80255	28.65254	37.84328
ENSSSCG00000002529	718.3457396	-1.329993521	5.36E-06	0.000279	329.859	480.8917	415.6654	1061.106	1297.854	724.6987
ENSSSCG00000025881	3477.538578	1.475372057	5.36E-06	0.000279	7285.748	2906.723	5153.454	1504.479	2316.611	1698.217
ENSSSCG00000006372	577.2761178	1.296722437	5.61E-06	0.00029	873.2127	974.4128	614.0286	410.4935	289.709	301.8001
ENSSSCG00000001456	5087.945368	1.330787649	5.69E-06	0.000292	7562.714	4641.819	9639.052	2335.429	3448.917	2899.741
ENSSSCG00000002432	62.96730678	2.406678317	5.7E-06	0.000292	132.7129	77.71987	107.6544	16.93784	10.61205	32.16678
ENSSSCG00000015423	1372.625782	-1.337465662	5.7E-06	0.000292	630.8673	1091.964	612.035	1924.936	2385.589	1590.364
ENSSSCG00000027487	262.3855136	1.906898705	5.75E-06	0.000294	722.2277	292.421	228.2671	93.65629	107.1817	130.5593
ENSSSCG00000036507	13.37697256	7.184052082	5.79E-06	0.000295	5.770128	48.57492	25.91679	0	0	0
ENSSSCG00000005740	41.02731372	3.698234321	5.88E-06	0.000299	113.4792	15.54397	99.67996	3.985374	2.12241	11.35298
ENSSSCG00000015911	81.71553333	-2.858259613	5.95E-06	0.000301	5.770128	45.66042	7.974397	153.4369	115.6714	161.78
ENSSSCG00000015355	1167.410107	-1.676461486	5.98E-06	0.000302	276.9661	624.6734	767.5357	1266.353	2536.28	1532.653
ENSSSCG00000001513	299.7074865	1.650928232	6.1E-06	0.000306	586.6297	238.9886	538.2718	167.3857	141.1403	125.8289
ENSSSCG00000007436	129.6539249	2.211386528	6.35E-06	0.000318	200.9928	339.0529	99.67996	25.90493	58.36628	53.92667
ENSSSCG00000012548	535.3419344	-1.49681469	6.53E-06	0.000326	183.6824	430.3738	226.2735	740.2832	867.0045	764.4342
ENSSSCG00000008230	863.9511437	1.723481591	6.68E-06	0.000332	1949.342	567.355	1462.305	343.7385	349.1365	511.8303
ENSSSCG00000004234	84.11144528	3.165527953	6.73E-06	0.000333	53.85453	259.3901	140.5487	11.95612	36.08097	2.838246
ENSSSCG00000013614	495.5416576	-1.459723177	6.76E-06	0.000334	179.8357	226.3591	386.7583	749.2503	607.0093	824.0373
ENSSSCG00000033095	736.2640185	-1.03977868	6.8E-06	0.000335	481.8057	502.2646	461.5182	1094.981	924.3096	952.7045
ENSSSCG00000006800	564.250142	1.395410597	6.82E-06	0.000335	924.1821	997.7288	531.2942	324.808	245.1384	362.3494
ENSSSCG00000026092	1725.649985	1.3709931	6.87E-06	0.000335	3094.712	1448.504	2923.613	869.8078	1127	890.2631
ENSSSCG00000033750	46.72912113	-2.468208502	6.85E-06	0.000335	17.31038	13.60098	11.9616	53.80255	106.1205	77.57872
ENSSSCG00000014378	25.25192499	-3.332629683	7.32E-06	0.000356	3.846752	4.857492	4.983998	29.8903	53.06025	54.87275
ENSSSCG00000029668	202.9422979	1.411625372	7.56E-06	0.000366	283.698	289.5065	311.9983	129.5247	82.774	120.1524
ENSSSCG00000003894	6801.596763	1.391657292	7.63E-06	0.000368	10531.44	6637.277	12379.25	5238.774	3375.693	2647.137
ENSSSCG00000017700	17.71643183	5.696108279	7.69E-06	0.00037	38.46752	38.85993	26.91359	0.996343	1.061205	0
ENSSSCG00000007278	670.4991933	1.082301758	7.74E-06	0.000371	1022.274	886.0065	824.3533	408.5008	402.1967	479.6635
ENSSSCG00000009405	3313.21417	-1.314850844	7.92E-06	0.000379	1681.031	2539.497	1479.251	3616.727	4236.331	6326.45

ENSSSCG00000012795	525.9103977	1.766120805	7.94E-06	0.000379	1138.639	340.9959	958.9212	309.8628	202.6902	204.3537
ENSSSCG00000028997	820.8888293	-1.066062684	8.04E-06	0.000382	540.4686	538.2101	513.3518	1312.184	1023.002	998.1164
ENSSSCG00000008749	601.0373546	-1.287462582	8.56E-06	0.000405	351.0161	413.8583	283.0911	1117.897	736.4763	703.8849
ENSSSCG00000022536	510.0458027	1.12544089	8.73E-06	0.000412	765.5036	598.443	734.6413	314.8445	293.9538	352.8885
ENSSSCG00000009231	96.92535378	2.167091053	8.82E-06	0.000414	156.7551	183.6132	135.5647	40.85008	12.73446	52.0345
ENSSSCG00000025672	245.9854694	-1.6043478	8.82E-06	0.000414	96.1688	155.4397	113.6352	521.0876	341.708	247.8735
ENSSSCG00000004728	302.6523238	1.303040363	8.98E-06	0.00042	483.729	457.5757	350.8735	210.2285	160.242	153.2653
ENSSSCG00000032844	101.830679	-2.126699279	9.09E-06	0.000424	16.3487	44.68892	52.83038	143.4735	224.9755	128.6671
ENSSSCG00000025910	1857.954438	-1.010842554	9.18E-06	0.000427	1365.597	1063.791	1267.929	2799.725	2326.162	2324.523
ENSSSCG00000021084	1111.881251	1.915087832	9.22E-06	0.000427	3404.375	887.9495	980.8508	564.9267	345.9529	487.2322
ENSSSCG00000003374	31.7273778	-4.355715793	9.41E-06	0.000435	0.961688	0.971498	6.977597	7.970748	81.71279	91.76994
ENSSSCG00000033051	48.93415402	2.400001071	9.69E-06	0.000446	92.32205	73.83387	80.74077	23.91224	9.550846	13.24515
ENSSSCG00000038660	96.52278853	1.948459914	9.77E-06	0.000449	191.3759	99.09283	169.4559	53.80255	36.08097	29.32854
ENSSSCG00000039476	121.7280361	1.810681213	1.01E-05	0.000461	207.7246	118.5228	242.2223	42.84277	50.93784	68.1179
ENSSSCG00000039573	10369.03364	4.16764203	1.02E-05	0.000463	16012.1	2771.685	40151.09	1327.13	1015.573	936.6211
ENSSSCG00000040288	87.72400399	2.280688537	1.02E-05	0.000465	184.6441	159.3257	92.70236	45.8318	11.67326	32.16678
ENSSSCG00000027935	136.7912303	-2.032084771	1.03E-05	0.000469	27.88895	82.57736	50.83678	298.903	131.5894	228.9518
ENSSSCG00000012397	232.3949331	1.2472172	1.05E-05	0.000475	345.246	309.908	325.9535	149.4515	129.467	134.3436
ENSSSCG00000013513	167.1490891	1.791669567	1.07E-05	0.00048	295.2382	328.3664	154.5039	46.82814	88.08002	89.87778
ENSSSCG00000038968	1531.714675	-1.144299535	1.07E-05	0.000481	851.0939	1262.948	748.5965	2075.383	2046.003	2206.263
ENSSSCG00000000576	27995.39693	-1.037985215	1.07E-05	0.000482	17126.7	17025.51	20860.03	37926.81	44987.67	30045.67
ENSSSCG00000032381	2070.102743	1.170470025	1.08E-05	0.000484	3243.774	1980.885	3375.163	1128.857	1407.158	1284.779
ENSSSCG00000006900	934.5236253	-1.037904007	1.12E-05	0.000498	528.9284	701.4218	606.0542	1274.323	1340.302	1156.112
ENSSSCG00000027607	187.9572088	1.949330653	1.21E-05	0.000537	418.3343	154.4682	322.9631	39.85374	96.56966	95.55427
ENSSSCG00000002831	414.5635244	-1.429892811	1.24E-05	0.000542	266.3876	144.7533	262.1583	767.1845	580.4792	466.4184
ENSSSCG00000002868	1494.946702	1.035903237	1.23E-05	0.000542	2049.357	2035.289	1944.756	1156.755	786.353	997.1703
ENSSSCG00000023666	16881.87863	1.165855168	1.23E-05	0.000542	29254.55	16741.83	24067.73	10609.07	8434.458	12183.64
ENSSSCG00000032242	363.6910588	1.713507702	1.24E-05	0.000542	804.9328	314.7655	552.227	144.4698	249.3832	116.3681
ENSSSCG00000034570	1032.839044	1.274827569	1.23E-05	0.000542	1723.345	982.1848	1679.607	557.9523	509.3784	744.5665
ENSSSCG00000007522	2086.374397	1.246543666	1.24E-05	0.000543	4196.806	2269.42	2340.485	1312.184	1090.919	1308.431
ENSSSCG00000031951	37.81108595	-2.649366427	1.25E-05	0.000544	14.42532	8.743485	7.974397	49.81717	89.14123	56.76491
ENSSSCG00000004787	491.0918613	1.281509877	1.26E-05	0.000547	667.4115	532.3811	888.1485	246.0968	265.3013	347.2121
ENSSSCG00000012517	13.16231103	-7.196664206	1.27E-05	0.00055	0	0	0	41.84643	2.12241	35.00503
ENSSSCG00000017355	2637.093806	1.46827811	1.3E-05	0.00056	5821.097	1998.372	3802.791	1293.254	1288.303	1618.746
ENSSSCG00000024810	264.6006623	1.420189563	1.31E-05	0.000563	406.794	356.5399	392.739	162.404	91.26364	177.8634

ENSSSCG00000003600	6944.64389	1.143650324	1.33E-05	0.000568	10092.92	6486.694	12105.13	4263.354	4532.407	4187.358
ENSSSCG00000013041	515.7470852	1.197969184	1.33E-05	0.000568	932.8373	607.1865	615.0254	324.808	310.9331	303.6923
ENSSSCG00000037706	13976.85169	-1.234829373	1.34E-05	0.000571	6054.787	12027.15	6924.767	18426.38	22143.11	18284.92
ENSSSCG00000013604	539.0794306	1.197326078	1.35E-05	0.000574	920.3354	753.8827	578.1438	360.6763	286.5254	334.913
ENSSSCG00000028376	17.51747559	4.685303862	1.37E-05	0.000583	36.54414	40.80293	23.92319	0.996343	0	2.838246
ENSSSCG00000022429	234.1261978	-1.718448943	1.41E-05	0.000594	155.7935	113.6653	57.81438	485.2193	314.1167	278.1481
ENSSSCG00000002309	28.8258313	4.183577231	1.42E-05	0.000599	55.7779	19.42997	88.71517	7.970748	1.061205	0
ENSSSCG00000037426	266.0637816	1.814785287	1.42E-05	0.000599	541.4303	378.8844	322.9631	178.3455	49.87664	124.8828
ENSSSCG00000032434	149.4939149	1.960928116	1.46E-05	0.000613	370.2499	167.0977	176.4335	88.67457	33.95856	60.54924
ENSSSCG00000006793	438.1445403	1.784086555	1.47E-05	0.000616	880.9062	868.5195	288.0751	153.4369	155.9971	281.9324
ENSSSCG00000006132	254.7050427	-1.718522217	1.5E-05	0.000626	83.66685	175.8412	96.68956	533.0438	230.2815	408.7074
ENSSSCG00000034566	610.7084789	1.790296792	1.52E-05	0.000631	1194.416	337.1099	1310.791	248.0895	344.8917	228.9518
ENSSSCG00000003928	135.7927085	1.551973517	1.54E-05	0.00064	233.6902	147.6677	226.2735	65.75867	64.73351	76.63263
ENSSSCG00000023684	126.6462516	-2.666100817	1.55E-05	0.00064	16.3487	22.34446	64.79197	359.68	240.8936	55.81883
ENSSSCG00000000252	135.6538857	-2.170249765	1.59E-05	0.000654	42.31427	38.85993	66.78557	336.7641	252.5668	76.63263
ENSSSCG00000002893	124.8703037	1.931553176	1.63E-05	0.00067	199.0694	266.1905	128.5872	59.78061	26.53013	69.06398
ENSSSCG00000009645	257.8072484	-5.15829728	1.63E-05	0.00067	18.27207	1.942997	21.92959	937.5592	492.3992	74.74047
ENSSSCG00000009197	61.59397084	3.209432563	1.69E-05	0.00069	202.9162	115.6083	14.95199	5.978061	15.91808	14.19123
ENSSSCG00000024161	38.0222081	3.376176319	1.69E-05	0.00069	22.11882	55.37541	130.5807	1.992687	9.550846	8.514737
ENSSSCG00000040275	944.060784	1.146368595	1.7E-05	0.000692	1575.245	1047.275	1278.894	562.9341	709.9462	490.0704
ENSSSCG00000024351	507.6255186	-1.664165016	1.72E-05	0.000696	106.7474	271.048	352.8671	582.8609	775.7409	956.4888
ENSSSCG00000008799	10532.17131	1.025733707	1.73E-05	0.000701	16332.35	14163.47	11882.85	6202.238	6199.56	8412.56
ENSSSCG00000022492	303.5186834	1.716715875	1.74E-05	0.000701	658.7563	454.6612	283.0911	188.3089	76.40677	159.8878
ENSSSCG00000033541	659.4258713	1.722558225	1.75E-05	0.000704	926.1055	523.6376	1586.905	183.3272	301.3822	435.1977
ENSSSCG00000037158	2809.491244	-1.07025875	1.75E-05	0.000704	1479.076	2268.449	1690.572	3263.025	3877.643	4278.182
ENSSSCG00000033657	75.90296004	-3.027370451	1.77E-05	0.000709	2.885064	36.91694	9.967996	167.3857	50.93784	187.3242
ENSSSCG00000036946	48.32526296	2.339153768	1.8E-05	0.000718	58.66297	73.83387	109.648	10.95978	16.97928	19.86772
ENSSSCG00000003968	31.5915602	3.145011338	1.87E-05	0.000744	53.85453	88.40635	27.91039	4.981717	10.61205	3.784328
ENSSSCG00000024048	562.9205699	-1.310196488	1.9E-05	0.000754	304.8551	311.851	353.8639	510.1279	1063.327	833.4982
ENSSSCG00000022998	676.5124953	1.427759272	1.92E-05	0.000762	1157.872	537.2386	1263.942	305.8774	430.8493	363.2954
ENSSSCG00000034293	176.1026601	1.566616547	1.99E-05	0.000785	379.8667	219.5586	190.3887	85.68554	99.75328	81.36304
ENSSSCG00000035567	32.16282526	3.431587106	1.99E-05	0.000785	90.39867	26.23046	59.80798	4.981717	10.61205	0.946082
ENSSSCG00000033586	478.2177438	1.292408101	2E-05	0.000787	771.2738	447.8607	818.3725	318.8299	254.6892	258.2804
ENSSSCG00000000146	5954.674921	-1.080068508	2.09E-05	0.000817	2926.416	3937.483	4609.201	8563.572	6555.064	9136.313
ENSSSCG00000008966	221.1330205	1.405718419	2.11E-05	0.000822	252.9239	313.794	396.7262	142.4771	91.26364	129.6132

ENSSSCG00000010224	153.0114812	2.332081688	2.11E-05	0.000822	288.5064	254.5326	223.2831	40.85008	10.61205	100.2847
ENSSSCG00000014287	262.646042	1.518434229	2.15E-05	0.000835	394.2921	281.7345	492.419	148.4552	84.89641	174.0791
ENSSSCG00000038879	198.1741981	1.797379015	2.15E-05	0.000835	467.3804	135.0383	320.9695	92.65994	85.95761	87.03953
ENSSSCG00000001469	735.2565499	1.213480267	2.17E-05	0.000839	1283.853	859.776	938.9852	500.1644	322.6063	506.1538
ENSSSCG00000023611	8002.139746	1.084846572	2.18E-05	0.000841	13152.04	9207.861	10270.03	6380.584	4041.069	4961.253
ENSSSCG00000016668	98.04696537	1.804186961	2.28E-05	0.000876	99.05386	213.7296	144.5359	38.8574	46.69302	45.41193
ENSSSCG00000022029	64.47313944	2.455214536	2.28E-05	0.000876	130.7896	42.74593	153.5071	14.94515	29.71374	15.13731
ENSSSCG00000036441	172.0723344	-1.877153337	2.29E-05	0.000876	41.35258	111.7223	67.78237	180.3382	411.7476	219.491
ENSSSCG00000005040	1112.708037	-1.306985909	2.39E-05	0.000914	515.4648	852.9756	553.2238	1052.139	1695.806	2006.64
ENSSSCG00000007239	357.3459816	1.187757783	2.41E-05	0.000916	523.1583	383.7418	583.1278	204.2504	221.7919	228.0057
ENSSSCG00000025188	143.6382419	-2.032161385	2.46E-05	0.000934	60.58634	73.83387	34.88799	366.6544	99.75328	226.1136
ENSSSCG00000034360	916.5445496	1.631386348	2.56E-05	0.00097	664.5264	2099.408	1393.526	635.6671	317.3003	388.8397
ENSSSCG00000010491	40.54503735	2.703155531	2.58E-05	0.000977	78.85841	64.11889	67.78237	16.93784	12.73446	2.838246
ENSSSCG00000038757	597.2829786	-1.119642399	2.6E-05	0.000982	390.4453	374.9984	363.8319	799.0675	1017.696	637.6592
ENSSSCG00000001804	307.2728131	1.607483385	2.68E-05	0.001006	570.281	215.6726	602.067	154.4332	168.7316	132.4515
ENSSSCG00000022490	13.47642207	6.215436323	2.69E-05	0.00101	37.50583	19.42997	22.92639	0.996343	0	0
ENSSSCG00000006183	150.1083479	-1.63050377	2.7E-05	0.001012	49.04609	98.12133	72.76637	288.9396	166.6092	225.1675
ENSSSCG00000027826	1909.907376	1.507569871	2.73E-05	0.001018	2338.825	4559.242	1579.927	1031.215	718.4358	1231.799
ENSSSCG00000004570	7713.317039	-1.030930818	2.75E-05	0.001024	4092.944	6634.362	4479.617	10239.42	10359.48	10474.07
ENSSSCG00000005452	159.5458992	-1.562074005	2.76E-05	0.001026	58.66297	107.8363	75.75677	259.0493	174.0376	281.9324
ENSSSCG00000035256	201.0523499	1.425747457	2.8E-05	0.001039	292.3531	238.9886	347.8831	78.71113	109.3041	139.074
ENSSSCG00000030415	189.9256356	1.595855338	2.83E-05	0.001046	326.9739	243.8461	285.0847	77.71479	145.3851	60.54924
ENSSSCG00000016618	6955.745412	-1.017426405	2.84E-05	0.001047	3600.56	5860.078	4339.069	8844.541	9498.847	9591.378
ENSSSCG00000032135	31.70445641	3.627939618	2.86E-05	0.001053	52.89284	23.31596	99.67996	7.970748	6.36723	0
ENSSSCG00000014347	18.33585092	4.375319165	2.91E-05	0.001067	39.42921	12.62948	52.83038	0.996343	3.183615	0.946082
ENSSSCG00000032407	18.99986704	4.772953489	3.08E-05	0.001126	39.42921	9.714984	60.80478	2.98903	1.061205	0
ENSSSCG00000009474	9.283193986	6.657079543	3.12E-05	0.001133	15.38701	21.37296	18.93919	0	0	0
ENSSSCG00000010283	1547.489467	1.209564328	3.15E-05	0.001143	2776.393	1460.162	2245.79	942.5409	741.7823	1118.269
ENSSSCG00000006979	438.9563911	1.88756793	3.2E-05	0.001158	827.0517	931.6669	314.9887	177.3491	82.774	299.908
ENSSSCG00000032401	31.79508036	-2.911575653	3.22E-05	0.00116	9.61688	9.714984	2.990399	54.79889	79.59038	34.05895
ENSSSCG00000008240	337.0608019	1.493645072	3.31E-05	0.001192	511.618	348.7679	631.971	259.0493	151.7523	119.2063
ENSSSCG00000035736	679.0246277	1.449094112	3.33E-05	0.001193	762.6186	933.6099	1285.872	565.9231	245.1384	280.9863
ENSSSCG00000021601	619.2054863	-1.190686153	3.37E-05	0.001205	309.6635	510.0366	311.9983	771.1698	991.1655	821.1991
ENSSSCG00000032116	300.6565285	1.292411971	3.39E-05	0.001208	521.2349	301.1645	458.5278	148.4552	191.0169	183.5399
ENSSSCG00000006013	62.55050179	2.018036737	3.44E-05	0.001224	90.39867	107.8363	102.6704	13.94881	33.95856	26.49029

ENSSSCG00000006037	6461.808991	-1.144667025	3.48E-05	0.001232	3849.637	5124.654	3100.047	8605.419	11320.94	6770.162
ENSSSCG00000034036	29.97165239	3.073745179	3.48E-05	0.001232	61.54803	20.40147	78.74717	6.974404	7.428436	4.73041
ENSSSCG00000031022	9.386639879	-6.708479879	3.49E-05	0.001233	0	0	0	26.90127	9.550846	19.86772
ENSSSCG00000032380	111.6356138	1.98748472	3.61E-05	0.001271	136.5597	89.37785	309.0079	45.8318	44.57061	44.46585
ENSSSCG00000000362	1516.516118	1.815579794	3.71E-05	0.001301	1182.876	1483.478	4419.809	374.6251	703.579	934.7289
ENSSSCG00000009085	280.5939205	-1.230036436	3.78E-05	0.001321	134.6363	199.1572	169.4559	329.7897	401.1355	449.3889
ENSSSCG00000012927	840.3880813	1.394338032	3.83E-05	0.001331	845.3237	1358.155	1449.347	262.0383	529.5413	597.9238
ENSSSCG00000031524	10.82954852	-6.912842379	3.82E-05	0.001331	0	0	0	39.85374	22.28531	2.838246
ENSSSCG00000021576	303.0779916	1.277026774	3.92E-05	0.001357	395.2538	375.9699	516.3422	200.265	126.2834	204.3537
ENSSSCG00000014048	262.2152945	1.497703973	3.98E-05	0.001374	501.0394	297.2785	363.8319	158.4186	79.59038	173.133
ENSSSCG00000011713	331.6001327	1.556479473	4.08E-05	0.001406	583.7446	646.0464	255.1807	185.3199	117.7938	201.5154
ENSSSCG00000031306	26.39867506	4.71566713	4.19E-05	0.001438	72.1266	5.82899	74.75997	0	0	5.676491
ENSSSCG00000006425	39.49064033	-2.882451676	4.25E-05	0.001456	3.846752	17.48697	6.977597	93.65629	30.77495	84.20129
ENSSSCG00000007927	2018.844634	1.404390848	4.3E-05	0.001468	4405.493	2791.115	1594.879	1210.557	1293.609	817.4148
ENSSSCG00000017152	772.6200368	1.281907963	4.39E-05	0.001494	1293.47	652.8469	1338.702	413.4825	397.9519	539.2667
ENSSSCG00000001695	1949.681144	-1.050790983	4.4E-05	0.001495	1030.93	1471.82	1305.807	2252.733	2346.324	3290.473
ENSSSCG00000034266	530.399604	1.108042765	4.47E-05	0.001506	687.6069	571.241	915.062	351.7092	333.2184	323.56
ENSSSCG00000034702	299.1291827	1.130188674	4.47E-05	0.001506	349.0927	427.4593	455.5374	188.3089	171.9152	202.4615
ENSSSCG00000013916	106.4764561	-2.472976088	4.53E-05	0.001523	19.23376	55.37541	22.92639	266.0237	43.50941	231.7901
ENSSSCG00000037360	8255.22758	1.015629494	4.56E-05	0.00153	9797.677	13802.08	9540.369	6314.825	4462.367	5614.05
ENSSSCG00000001887	212.030332	1.438321858	4.63E-05	0.001547	415.4492	289.5065	224.2799	146.4625	101.8757	94.60819
ENSSSCG00000017605	8982.804619	-1.143690745	4.63E-05	0.001547	4840.176	8027.491	3925.397	11695.08	12721.73	12686.96
ENSSSCG00000025447	546.3569389	1.183690799	4.64E-05	0.001548	994.3854	545.9821	735.6381	307.8701	373.5442	320.7218
ENSSSCG00000026945	122.6270376	-1.790587097	4.84E-05	0.001606	31.7357	90.34935	42.86238	165.393	231.3427	174.0791
ENSSSCG00000034980	202.6129422	1.720494052	4.83E-05	0.001606	390.4453	234.1311	308.0111	156.4259	77.46797	49.19626
ENSSSCG00000031147	3975.333399	-1.128856143	4.87E-05	0.001614	2133.024	3378.871	1972.666	4713.701	4874.115	6779.623
ENSSSCG00000039745	792.1300064	1.325697015	4.99E-05	0.001649	1386.754	823.8306	1187.188	438.3911	286.5254	630.0905
ENSSSCG00000017498	7302.605801	-1.005780381	5.05E-05	0.001666	4916.149	3672.264	5977.807	9128.499	11081.1	9039.813
ENSSSCG00000007644	27.48385331	3.022802012	5.08E-05	0.001672	72.1266	41.77443	32.89439	9.963435	5.306025	2.838246
ENSSSCG00000024736	329.4621592	1.578677249	5.1E-05	0.001676	590.4764	219.5586	670.8461	152.4406	192.0781	151.3731
ENSSSCG00000004823	1232.146733	1.112249507	5.16E-05	0.001692	2035.893	1329.981	1688.579	902.6872	859.5761	576.1639
ENSSSCG00000006777	213.9661984	2.004027797	5.21E-05	0.001696	428.9128	89.37785	509.3646	95.64897	75.34556	85.14737
ENSSSCG00000024299	75.89905202	-1.876111714	5.21E-05	0.001696	25.96558	48.57492	22.92639	93.65629	133.7118	130.5593
ENSSSCG00000008903	186.3856041	1.637890944	5.24E-05	0.001701	206.7629	280.763	358.8479	143.4735	67.91713	60.54924
ENSSSCG00000030167	817.0509215	1.696889675	5.26E-05	0.001703	1667.567	389.5708	1689.575	408.5008	345.9529	401.1387

ENSSSCG00000009589	200.6697987	1.334598564	5.37E-05	0.001735	295.2382	352.6539	214.3119	96.64532	114.6101	130.5593
ENSSSCG00000005930	159.4066215	1.443366303	5.42E-05	0.00175	276.9661	166.1262	256.1775	104.6161	80.65159	71.90222
ENSSSCG00000036208	304.3734899	-1.265404391	5.45E-05	0.001753	174.0655	204.9862	157.4943	309.8628	424.482	555.3501
ENSSSCG00000011925	296.0663636	1.910917544	5.56E-05	0.001786	415.4492	755.8257	232.2543	136.4991	50.93784	185.4321
ENSSSCG00000038706	2866.763035	1.313755096	5.62E-05	0.0018	4325.672	5274.265	2666.439	2285.612	1068.634	1579.957
ENSSSCG00000031866	42165.39091	1.160524276	5.76E-05	0.001842	83723.59	45181.47	45891.66	22272.26	31609.05	24314.3
ENSSSCG00000008196	42.76890071	2.261691891	5.8E-05	0.00185	67.31816	61.2044	83.73117	19.92687	15.91808	8.514737
ENSSSCG00000015023	1933.338751	-1.110454975	5.82E-05	0.001852	1029.006	1628.231	1014.742	2321.48	2329.345	3277.228
ENSSSCG00000040795	17.58891269	-4.0874449	5.84E-05	0.001857	2.885064	0	2.990399	44.83546	16.97928	37.84328
ENSSSCG00000024399	372.6197204	-1.04246719	5.9E-05	0.001872	212.533	273.9625	244.2159	487.212	504.0724	513.7225
ENSSSCG00000014143	10.04027992	6.77057918	5.93E-05	0.001877	11.54026	43.71743	4.983998	0	0	0
ENSSSCG00000015085	567.9553634	1.07737906	5.98E-05	0.001885	822.2432	773.3127	716.6989	448.3546	279.0969	368.0259
ENSSSCG00000025858	4655.640254	-1.431332503	5.99E-05	0.001885	1836.824	1700.122	4019.096	9765.162	5533.123	5079.514
ENSSSCG00000028355	17105.39303	1.006000319	6.16E-05	0.001929	25327.98	21262.21	21926.6	11914.28	8220.095	13981.2
ENSSSCG00000040728	32.92372561	-3.643341144	6.15E-05	0.001929	0	12.62948	1.993599	28.89396	102.9369	51.08842
ENSSSCG00000004752	86.61653319	-1.625227134	6.26E-05	0.001952	40.39089	32.05945	54.82398	132.5137	128.4058	131.5054
ENSSSCG00000040977	550.3530763	1.133580102	6.26E-05	0.001952	935.7224	538.2101	794.4493	351.7092	313.0555	368.9719
ENSSSCG00000005657	327.9488819	1.45590508	6.33E-05	0.001971	533.7368	264.2476	643.9326	203.2541	191.0169	131.5054
ENSSSCG00000035420	470.7406982	1.449976181	6.36E-05	0.001974	959.7646	534.3241	573.1598	257.0566	354.4425	145.6966
ENSSSCG00000035537	316.3320834	1.277329797	6.39E-05	0.001979	481.8057	516.8371	344.8927	241.1151	176.16	137.1819
ENSSSCG00000002907	1265.142867	1.484083823	6.45E-05	0.001987	2511.929	775.2557	2304.601	571.9012	798.0262	629.1445
ENSSSCG00000003729	6336.518931	-1.024473348	6.47E-05	0.001987	3085.095	5341.298	4103.824	7708.709	8202.054	9578.133
ENSSSCG00000015821	10.14591583	6.786009412	6.45E-05	0.001987	47.12271	7.771987	5.980798	0	0	0
ENSSSCG00000017181	1570.904288	1.209015861	6.46E-05	0.001987	2393.641	1691.379	2493.993	727.3307	1367.893	751.189
ENSSSCG00000006890	358.7459123	1.957169847	6.58E-05	0.00202	563.5492	166.1262	981.8476	169.3784	183.5885	87.98562
ENSSSCG00000017106	602.5236065	1.294932245	6.64E-05	0.002033	980.9217	476.0342	1111.432	365.6581	348.0753	333.0208
ENSSSCG00000004454	18815.05903	1.015074746	6.67E-05	0.002038	24230.69	22941.93	28348.98	9549.952	16292.68	11526.12
ENSSSCG00000007857	126.6340412	-1.40028361	6.69E-05	0.002039	69.24153	80.63436	58.81118	188.3089	204.8126	157.9957
ENSSSCG00000039962	1577.703563	1.085054509	6.74E-05	0.002051	2021.468	1716.638	2695.346	787.1113	1235.243	1010.415
ENSSSCG00000015050	645.7514541	-1.003819081	6.84E-05	0.002078	423.1427	505.1791	360.8415	954.497	826.6788	804.1696
ENSSSCG00000036013	153.1598395	-5.950127409	6.9E-05	0.002088	0	12.62948	1.993599	131.5173	644.1515	128.6671
ENSSSCG00000003983	777.0083753	1.041910182	7E-05	0.002112	1307.896	994.8143	835.3181	551.9743	457.3794	514.6686
ENSSSCG00000001597	13.74816109	6.24563097	7.09E-05	0.002136	55.7779	5.82899	19.93599	0	0	0.946082
ENSSSCG00000017980	27.99195499	-2.848968964	7.16E-05	0.002151	10.57857	2.914495	6.977597	37.86105	42.4482	67.17181
ENSSSCG00000006717	660.3442561	-1.604296764	7.26E-05	0.002173	444.2998	194.2997	341.9023	1406.837	1129.122	445.6046

ENSSSCG00000013073	595.8352208	1.534118168	7.37E-05	0.002197	1051.125	360.4259	1246	287.9433	273.7909	355.7268
ENSSSCG00000013114	400.8666212	1.634230229	7.37E-05	0.002197	849.1705	480.8917	489.4286	243.1078	79.59038	263.0108
ENSSSCG00000030395	768.5429301	-1.110573766	7.52E-05	0.002234	356.7862	636.3314	466.5022	913.647	1068.634	1169.357
ENSSSCG00000008579	39.88250584	2.304310767	7.6E-05	0.002252	50.96946	60.2329	87.71837	15.9415	15.91808	8.514737
ENSSSCG00000024660	337.7579777	-1.427877333	7.61E-05	0.002252	153.8701	287.5635	107.6544	507.1388	441.4613	528.8598
ENSSSCG00000024814	522.3208042	1.098578305	7.71E-05	0.002276	697.2238	711.1368	727.6637	258.053	436.1553	303.6923
ENSSSCG00000014921	3550.819854	1.023618801	7.77E-05	0.002289	6358.681	3925.825	3996.17	2430.082	2084.207	2509.955
ENSSSCG00000030767	46.34786321	3.172781699	7.78E-05	0.002289	85.59023	34.97394	129.5839	10.95978	16.97928	0
ENSSSCG00000014051	182.1447032	1.369329518	7.89E-05	0.002316	269.2726	229.2736	289.0719	83.69285	141.1403	80.41696
ENSSSCG00000004547	1311.54907	1.065044128	8.27E-05	0.002417	2375.369	1324.152	1624.783	836.9285	910.514	797.547
ENSSSCG00000011443	2059.824789	1.002006393	8.44E-05	0.002461	2934.11	2971.813	2337.495	1547.321	1016.634	1551.574
ENSSSCG00000036203	24.47435008	-3.072247858	8.54E-05	0.002488	7.693504	2.914495	4.983998	69.74404	35.01977	26.49029
ENSSSCG00000022679	63.97930976	-2.958444619	8.65E-05	0.002514	15.38701	20.40147	7.974397	21.91956	55.18266	263.0108
ENSSSCG00000016298	439.9246015	1.149286043	8.76E-05	0.002541	634.7141	711.1368	473.4798	345.7312	231.3427	243.143
ENSSSCG00000023716	303.6225603	-1.652675231	9.01E-05	0.00261	74.04997	237.0456	128.5872	461.307	289.709	631.0366
ENSSSCG00000039332	114.1063258	1.633677608	9.12E-05	0.002636	129.8279	256.4756	131.5775	56.79158	45.63182	64.33357
ENSSSCG00000040101	226.9052991	1.283411764	9.18E-05	0.002647	336.5908	247.7321	380.7775	166.3894	105.0593	124.8828
ENSSSCG00000025618	881.6471346	1.008043509	9.22E-05	0.002649	1349.248	888.921	1294.843	594.8171	630.3558	531.698
ENSSSCG00000014570	2069.605922	-1.252520791	9.64E-05	0.002759	1080.937	1826.417	763.5485	3182.321	3309.899	2254.513
ENSSSCG00000033512	3085.847719	1.212445979	0.000101	0.002876	4855.563	5075.107	3003.357	2225.831	1137.612	2217.616
ENSSSCG00000008072	4182.120466	-1.425620919	0.000101	0.002881	1148.255	3558.598	2100.257	7806.351	4085.64	6393.621
ENSSSCG00000036383	412.34275	1.444854041	0.000102	0.002883	919.3737	377.9129	512.355	202.2577	161.3032	300.854
ENSSSCG00000033375	40.16040595	-2.753876469	0.000102	0.002888	7.693504	21.37296	1.993599	63.76598	91.26364	54.87275
ENSSSCG00000027070	76.19626798	1.618470113	0.000104	0.002931	99.05386	133.0953	112.6384	33.87568	43.50941	35.00503
ENSSSCG00000040931	67.43833561	2.034036075	0.000105	0.002971	148.0999	53.43241	123.6032	18.93053	35.01977	25.54421
ENSSSCG00000022250	59.07348705	1.967684178	0.000106	0.002991	81.74348	70.91938	129.5839	32.87933	22.28531	17.02947
ENSSSCG00000021374	2900.097484	1.384846112	0.000107	0.002997	4319.902	2151.869	6110.382	1629.022	2103.308	1086.102
ENSSSCG00000000025	110.2507852	1.425992085	0.000107	0.002998	166.372	161.2687	154.5039	74.72576	48.81543	55.81883
ENSSSCG00000033623	221.4043228	1.351093062	0.000108	0.003008	401.9856	298.25	254.1839	168.382	90.20243	115.422
ENSSSCG00000031653	29.73045548	2.929844962	0.000108	0.003009	81.74348	27.20195	48.84318	2.98903	5.306025	12.29906
ENSSSCG00000037697	7730.16491	-1.235737375	0.000108	0.003021	3619.794	4112.353	6092.439	14842.53	6430.903	11282.97
ENSSSCG00000000894	51.87695906	-2.018247394	0.000109	0.003023	12.50194	25.25896	23.92319	86.68188	62.6111	100.2847
ENSSSCG00000021181	514.7065619	1.340461516	0.000109	0.003037	434.683	649.9324	1129.374	329.7897	283.3418	261.1186
ENSSSCG00000001788	34.84733445	2.43490998	0.000112	0.00308	78.85841	37.88844	59.80798	9.963435	7.428436	15.13731
ENSSSCG00000007286	4659.739805	1.399477034	0.000112	0.003092	7658.883	3026.217	9588.215	1981.727	3300.348	2403.048

ENSSSCG00000011717	49.49144712	-1.964173571	0.000113	0.003101	22.11882	14.57248	23.92319	70.74039	70.03954	95.55427
ENSSSCG00000015299	302.5472418	-1.23149119	0.000113	0.003101	169.2571	192.3567	180.4207	354.6983	592.1524	326.3983
ENSSSCG00000010559	46.4045594	2.432187974	0.000115	0.003154	115.4026	38.85993	80.74077	21.91956	6.36723	15.13731
ENSSSCG00000007287	613.7063605	1.050993069	0.000116	0.003163	881.8679	621.7589	979.854	438.3911	415.9924	344.3738
ENSSSCG00000038954	502.4817885	1.127807816	0.000116	0.003163	672.2199	512.9511	883.1645	367.6507	313.0555	265.849
ENSSSCG00000004039	337.8714569	-1.381183566	0.000117	0.003167	163.487	265.219	133.5711	659.5794	473.2975	332.0747
ENSSSCG00000021933	9.047267303	6.620502186	0.000117	0.003167	19.23376	32.05945	2.990399	0	0	0
ENSSSCG00000031336	98.45495633	2.507824518	0.000118	0.003177	82.70517	102.9788	316.9823	13.94881	11.67326	62.44141
ENSSSCG00000007980	277.6983187	1.346381148	0.000118	0.003183	552.0089	278.82	364.8287	174.3601	183.5885	112.5837
ENSSSCG00000006648	2559.20481	1.089859603	0.000119	0.003206	4600.715	3548.883	2297.623	1638.985	1415.648	1853.374
ENSSSCG00000040252	483.4280427	-1.035437313	0.00012	0.003209	263.5025	387.6278	300.0367	618.7293	597.4585	733.2135
ENSSSCG00000001068	147.3266687	-1.650670964	0.000122	0.003269	38.46752	87.43485	87.71837	311.8555	147.5075	210.9763
ENSSSCG00000008745	881.4552119	-1.178455601	0.000122	0.003274	529.8901	560.5545	530.2974	753.2357	1202.345	1712.408
ENSSSCG00000030597	680.542125	1.577154479	0.000124	0.003292	988.6152	534.3241	1535.071	382.5959	473.2975	169.3487
ENSSSCG00000032452	2323.339829	3.329099941	0.000124	0.003292	5062.325	665.4764	6950.684	314.8445	467.9914	478.7174
ENSSSCG00000034356	7.99955486	-6.474986169	0.000125	0.003305	0	0	0	12.95247	26.53013	8.514737
ENSSSCG00000032936	875.9616047	1.542162469	0.000127	0.003352	1975.307	490.6067	1446.356	354.6983	550.7654	438.0359
ENSSSCG00000040498	16.35721388	3.962730658	0.000127	0.003352	27.88895	14.57248	49.83998	0.996343	1.061205	3.784328
ENSSSCG00000033849	312.3020592	1.35218306	0.000128	0.003371	626.0589	324.4804	395.7294	228.1627	178.2825	121.0985
ENSSSCG00000012893	625.0813163	1.345405763	0.000131	0.003435	775.1205	664.5049	1251.98	478.2449	206.935	373.7024
ENSSSCG00000027967	117.3579138	1.434915801	0.000132	0.003435	203.8778	188.4707	121.6096	60.77695	67.91713	61.49532
ENSSSCG00000036984	261.8718785	-1.636956592	0.000131	0.003435	105.7857	218.5871	57.81438	308.8665	491.338	388.8397
ENSSSCG00000039548	619.2104535	-1.454838029	0.000132	0.003435	377.9434	268.1335	346.8863	1339.086	985.8595	397.3544
ENSSSCG00000002814	40.08542863	2.610159443	0.000133	0.003456	111.5558	21.37296	73.76317	9.963435	10.61205	13.24515
ENSSSCG00000027121	393.2816498	1.031770105	0.000133	0.003469	594.3232	438.1458	552.227	290.9323	232.4039	251.6578
ENSSSCG00000007157	126.3245998	1.771121685	0.000135	0.00349	195.2227	115.6083	275.1167	43.83911	91.26364	36.89719
ENSSSCG00000015603	8177.136976	-1.290553375	0.000136	0.003527	3477.464	7668.036	3091.076	12135.46	13857.22	8833.567
ENSSSCG00000012121	46.65554564	-3.034948392	0.000137	0.003531	0	15.54397	14.95199	84.68919	36.08097	128.6671
ENSSSCG00000011806	179.3449131	2.214023189	0.000137	0.003531	277.9278	488.6637	118.6192	126.5356	43.50941	20.8138
ENSSSCG00000010394	71.11903379	-1.655990297	0.000138	0.003549	28.85064	29.14495	44.85598	112.5868	98.69207	112.5837
ENSSSCG00000004897	1796.565058	-1.055286565	0.000141	0.003613	890.5231	1416.445	1195.163	2452.998	1815.722	3008.54
ENSSSCG00000000867	47.77410384	-2.759101601	0.000143	0.003672	11.54026	23.31596	1.993599	144.4698	55.18266	50.14234
ENSSSCG00000036785	97.53061226	1.937199275	0.000144	0.003682	194.261	96.17834	173.4431	62.76964	42.4482	16.08339
ENSSSCG00000034364	167.9210837	1.413155365	0.000144	0.00369	358.7096	191.3852	182.4143	90.66726	85.95761	98.39252
ENSSSCG00000031493	396.4102097	1.539072839	0.000146	0.00373	992.462	352.6539	424.6366	189.3053	142.2015	277.202

ENSSSCG00000033246	102.8057387	2.35463856	0.000148	0.003754	214.4564	54.40391	247.2063	61.7733	10.61205	28.38246
ENSSSCG00000022295	293.0853023	1.893849738	0.000148	0.003765	578.9362	102.9788	703.7405	126.5356	125.2222	121.0985
ENSSSCG00000008767	220.7621882	2.008342852	0.000149	0.00378	661.6413	93.26384	306.0175	119.5612	63.6723	80.41696
ENSSSCG00000015999	473.9462879	-1.066307512	0.00015	0.003786	221.1882	376.9414	320.9695	673.5282	648.3963	602.6542
ENSSSCG00000013492	156.4812106	1.441898554	0.000151	0.003808	294.2765	149.6107	242.2223	70.74039	108.2429	73.79439
ENSSSCG00000005149	23.09055978	-3.076562562	0.000152	0.003826	0.961688	8.743485	4.983998	38.8574	50.93784	34.05895
ENSSSCG00000009662	107.1330261	-1.926556935	0.000156	0.003915	40.39089	43.71743	49.83998	68.7477	141.1403	298.9619
ENSSSCG00000010142	942.1067179	1.155317334	0.000156	0.003915	1706.996	1167.741	1026.704	491.1973	460.563	799.4392
ENSSSCG00000009693	58.57743844	-1.996537433	0.000158	0.003921	24.0422	15.54397	30.90079	58.78426	85.95761	136.2358
ENSSSCG00000016577	591.2143047	1.126088808	0.000157	0.003921	927.0672	514.8941	990.8188	361.6727	363.9933	388.8397
ENSSSCG00000017894	318.5084515	1.592023099	0.000158	0.003921	783.7757	223.4446	427.627	124.5429	215.4246	136.2358
ENSSSCG00000038322	1797.640545	2.92793355	0.000157	0.003921	4593.983	1027.845	3911.442	210.2285	320.4839	721.8605
ENSSSCG00000038933	431.0041573	-1.372295248	0.000159	0.003942	162.5253	391.5138	166.4655	694.4514	661.1308	509.9381
ENSSSCG00000031091	42.13952452	-2.876412215	0.000163	0.004033	0.961688	20.40147	8.971197	66.75501	40.32579	115.422
ENSSSCG00000034367	64.3352305	2.388446414	0.000166	0.004106	167.3337	27.20195	129.5839	29.8903	15.91808	16.08339
ENSSSCG00000017126	331.0485794	1.16415381	0.000169	0.004171	556.8173	380.8274	435.6014	249.0859	215.4246	148.5349
ENSSSCG00000032436	418.1336889	1.422793534	0.000171	0.004196	750.1166	280.763	796.4429	181.3345	232.4039	267.7412
ENSSSCG00000036206	838.3635065	-1.285474693	0.000171	0.004196	450.07	711.1368	302.0303	1597.139	898.8407	1070.965
ENSSSCG00000030113	73.68914141	-2.953082081	0.000173	0.004234	6.731816	39.83143	3.987198	253.0712	29.71374	108.7994
ENSSSCG00000015334	17426.76258	-1.611346359	0.000176	0.004283	3177.417	14025.52	8580.451	27138.4	14369.78	37269
ENSSSCG00000025005	225.6241469	-1.512383641	0.000176	0.004296	76.93504	181.6702	92.70236	217.2029	444.6449	340.5895
ENSSSCG00000026079	17.48082949	-3.278308623	0.000177	0.004306	1.923376	2.914495	4.983998	22.9159	37.14218	35.00503
ENSSSCG00000014443	47.75124701	-2.70604425	0.000178	0.004324	17.31038	6.800488	13.95519	15.9415	119.9162	112.5837
ENSSSCG00000007944	342.6355183	1.278903215	0.000179	0.004336	653.9478	322.5375	479.4606	256.0603	151.7523	192.0546
ENSSSCG00000038644	363.3187902	-1.303689357	0.00018	0.004347	246.1921	178.7557	203.3471	533.0438	720.5582	298.0158
ENSSSCG00000012426	143.8945282	-1.337191778	0.000182	0.004396	75.97335	103.9503	64.79197	200.265	246.1996	172.1869
ENSSSCG00000022289	1450.504172	1.300406709	0.000186	0.004493	2789.857	961.7834	2438.172	715.3746	944.4725	853.3659
ENSSSCG00000020706	183.9956227	-1.147114739	0.000187	0.004494	109.6324	134.0668	99.67996	253.0712	256.8116	250.7117
ENSSSCG00000016685	168.9148114	-1.225051937	0.000189	0.004519	99.05386	101.0358	103.6672	253.0712	180.4049	276.2559
ENSSSCG00000022500	94.36263009	-1.907329048	0.000189	0.004519	57.70128	13.60098	47.84638	109.5978	188.8945	148.5349
ENSSSCG00000012347	110.7885316	1.838988015	0.000191	0.004554	242.3454	102.9788	174.4399	31.88299	30.77495	82.30913
ENSSSCG00000021696	469.7401839	-1.38978207	0.000194	0.004628	204.8395	246.7606	326.9503	1107.934	497.7052	434.2516
ENSSSCG00000039838	146.3060403	1.446724133	0.000196	0.004668	274.0811	174.8697	193.3791	109.5978	53.06025	72.84831
ENSSSCG00000037142	470.0096511	1.449112763	0.000197	0.00468	1177.106	472.1482	414.6686	280.9689	281.2193	193.9468
ENSSSCG00000013989	34.79515658	-2.681441598	0.000202	0.004784	12.50194	11.65798	3.987198	60.77695	23.34651	96.50035

ENSSSCG00000004215	571.0311885	-1.22916548	0.000203	0.004789	284.6596	408.0293	331.9343	1031.215	457.3794	912.969
ENSSSCG00000015098	43.82589668	-2.319490251	0.000202	0.004789	20.19545	10.68648	12.95839	98.638	90.20243	30.27462
ENSSSCG00000003871	17.70161373	4.290374205	0.000204	0.004813	45.19933	42.74593	12.95839	0	5.306025	0
ENSSSCG00000001422	965.1724547	1.05654369	0.000208	0.004869	1506.965	1349.411	1054.614	796.0784	442.5225	641.4435
ENSSSCG00000017128	230.7094715	1.19373498	0.000208	0.004869	351.0161	222.4731	389.7486	139.4881	135.8343	145.6966
ENSSSCG00000037775	560.3947554	1.223583357	0.000208	0.004869	1029.968	635.3599	688.7885	472.2668	309.8719	226.1136
ENSSSCG00000037997	89.40969208	-1.853963925	0.000208	0.004869	47.12271	27.20195	41.86558	68.7477	222.8531	128.6671
ENSSSCG00000040709	210.6496699	1.682317256	0.000207	0.004869	509.6946	140.8673	312.9951	138.4917	96.56966	65.27965
ENSSSCG00000006106	20.05159932	-4.049442802	0.00021	0.004901	2.885064	0	3.987198	61.7733	5.306025	46.35801
ENSSSCG00000006495	128.9781123	1.293733456	0.00021	0.004912	166.372	206.9291	176.4335	88.67457	62.6111	72.84831
ENSSSCG00000033760	10436.75358	1.18955411	0.000212	0.004953	17201.71	7286.238	19045.85	6711.37	5717.773	6657.578
ENSSSCG00000001567	12.23138566	5.18371271	0.000214	0.00498	15.38701	27.20195	28.90719	0	0	1.892164
ENSSSCG00000028674	366.2487076	4.892349704	0.000217	0.005053	281.7746	1755.498	88.71517	15.9415	6.36723	49.19626
ENSSSCG00000031976	386.5128477	-1.060113768	0.000219	0.005075	237.5369	197.2142	316.9823	564.9267	545.4594	456.9576
ENSSSCG00000000136	266.8772166	1.502928458	0.000223	0.005171	347.1694	220.5301	616.0222	195.2833	103.9981	118.2602
ENSSSCG00000010067	671.5886702	1.275187473	0.000231	0.005338	1153.064	554.7256	1143.329	390.5666	521.0517	266.7951
ENSSSCG00000038886	142.1316403	-1.267247225	0.000231	0.005338	67.31816	92.29234	90.70876	235.1371	194.2005	173.133
ENSSSCG00000017874	707.5968757	1.094764957	0.000232	0.005342	1252.118	623.7019	1015.739	479.2412	476.4811	398.3005
ENSSSCG00000001782	64.01109257	2.150891229	0.000234	0.005362	176.9506	37.88844	98.68316	18.93053	22.28531	29.32854
ENSSSCG00000031072	373.1993995	-1.091847258	0.000234	0.005362	208.6863	303.1075	203.3471	469.2778	435.0941	619.6836
ENSSSCG00000009630	19.44369944	3.281154938	0.000235	0.005376	50.00777	37.88844	17.94239	5.978061	1.061205	3.784328
ENSSSCG00000008722	124.3355523	1.555585869	0.000238	0.005437	242.3454	101.0358	213.3151	77.71479	51.99905	59.60316
ENSSSCG00000014853	213.5488929	1.41229411	0.000238	0.005445	356.7862	174.8697	399.7166	153.4369	101.8757	94.60819
ENSSSCG00000033190	3629.287325	1.396575635	0.000239	0.005458	7288.633	1988.657	6504.117	2354.36	1991.882	1648.075
ENSSSCG00000038007	185.0166637	-1.510141472	0.000244	0.005557	44.23765	111.7223	132.5743	213.2175	314.1167	294.2315
ENSSSCG00000004919	690.9824394	1.164307073	0.000249	0.005654	1221.344	713.0798	932.0076	438.3911	558.1939	282.8785
ENSSSCG00000012967	37.03948775	2.306102809	0.000252	0.005723	94.24542	46.63192	43.85918	12.95247	16.97928	7.568655
ENSSSCG00000001596	270.5394824	1.454418139	0.000254	0.005758	495.2693	232.1881	461.5182	104.6161	221.7919	107.8533
ENSSSCG00000029267	140.9707014	1.42356072	0.000256	0.005796	222.1499	231.2166	162.4783	59.78061	112.4877	57.711
ENSSSCG00000007529	130.2644423	1.915379448	0.000261	0.005903	135.598	347.7964	134.5679	84.68919	21.2241	57.711
ENSSSCG00000029621	27.06416956	-2.957613662	0.000262	0.005911	1.923376	13.60098	2.990399	29.8903	47.75423	66.22573
ENSSSCG00000036983	142.7673971	4.932294854	0.000264	0.005933	191.3759	229.2736	408.6878	4.981717	22.28531	0
ENSSSCG00000032734	226.335276	1.259118462	0.000267	0.005984	387.5603	219.5586	350.8735	159.415	98.69207	141.9123
ENSSSCG00000005601	7201.694767	-1.094555969	0.000271	0.006071	3930.419	6031.062	3819.736	7878.088	7579.127	13971.74
ENSSSCG00000023788	215.5896594	1.05815159	0.000271	0.006071	285.6213	262.3046	325.9535	145.4661	137.9567	136.2358

ENSSSCG00000008593	436.9287905	1.002687117	0.000272	0.006074	729.9212	497.4072	521.3262	304.8811	308.8107	259.2264
ENSSSCG00000013144	1085.205388	1.209196853	0.000272	0.006074	1297.317	2193.643	1054.614	774.1589	430.8493	760.6498
ENSSSCG00000000660	755.8452902	1.279099488	0.000286	0.006345	1418.49	666.4479	1126.384	399.5337	635.6618	288.555
ENSSSCG00000037106	33.59461528	-2.240694284	0.000288	0.006359	8.655192	14.57248	11.9616	66.75501	63.6723	35.95111
ENSSSCG00000037424	739.6618961	-1.178969469	0.000288	0.006359	308.7018	595.5285	455.5374	1190.63	672.804	1214.769
ENSSSCG00000014420	178.8034787	2.201043369	0.00029	0.006396	337.5525	479.9202	63.79518	21.91956	63.6723	105.9612
ENSSSCG00000001553	20.31986272	-3.530825678	0.000291	0.006401	3.846752	4.857492	0.9968	23.91224	11.67326	76.63263
ENSSSCG00000015914	319.4202008	-1.293504892	0.000291	0.006401	112.5175	254.5326	188.3951	474.2595	325.79	561.0266
ENSSSCG00000003901	192.3099577	-1.379116545	0.000292	0.006409	108.6707	57.3184	154.5039	296.9104	270.6073	265.849
ENSSSCG00000003278	49.87041628	2.528536654	0.000293	0.00642	97.13048	133.0953	24.91999	6.974404	10.61205	26.49029
ENSSSCG00000034313	39.59484705	3.070229577	0.000297	0.00649	133.6746	7.771987	70.77277	3.985374	13.79567	7.568655
ENSSSCG00000009216	2558.062747	2.564798493	0.000297	0.006499	3083.172	6406.06	3640.312	526.0694	324.7288	1368.034
ENSSSCG00000010352	14.00931556	-4.006883741	0.000301	0.006572	0	0.971498	3.987198	33.87568	24.40772	20.8138
ENSSSCG00000006821	21.96063425	-2.79426366	0.000307	0.006675	2.885064	8.743485	4.983998	47.82449	27.59133	39.73544
ENSSSCG00000015412	127.003229	-1.758769799	0.000308	0.006687	32.69739	90.34935	50.83678	119.5612	150.6911	317.8835
ENSSSCG00000000399	46.65846103	2.125503682	0.000311	0.006731	65.39478	113.6653	48.84318	14.94515	10.61205	26.49029
ENSSSCG00000017258	202.579673	1.74939107	0.000316	0.006817	567.3959	244.8176	124.6	129.5247	75.34556	73.79439
ENSSSCG00000007849	667.2059471	1.383479896	0.000316	0.006821	829.9367	636.3314	1427.417	394.552	521.0517	193.9468
ENSSSCG00000024622	359.014655	-1.269474073	0.000325	0.006992	134.6363	323.509	173.4431	594.8171	423.4208	504.2617
ENSSSCG00000013596	139.0277823	1.463514107	0.000326	0.007011	247.1538	110.7508	254.1839	72.73307	85.95761	63.38749
ENSSSCG00000034058	4202.766496	1.121036306	0.000328	0.007054	7440.58	3085.479	6748.333	2357.349	2914.069	2670.789
ENSSSCG00000017137	238.012711	1.433419504	0.000332	0.007119	451.0317	223.4446	367.8191	197.276	80.65159	107.8533
ENSSSCG00000040162	6273.093867	-1.348151604	0.000338	0.007228	1997.426	6004.831	2612.612	10899	5831.322	10293.37
ENSSSCG00000005400	84.28700838	-1.731960661	0.000345	0.007313	34.62077	49.54642	32.89439	178.3455	63.6723	146.6427
ENSSSCG00000009180	601.0833493	-1.171431175	0.000345	0.007313	239.4603	566.3835	303.0271	852.87	841.5356	803.2235
ENSSSCG00000012824	190.6683893	1.113528428	0.000344	0.007313	283.698	220.5301	278.1071	134.5064	123.0998	104.069
ENSSSCG00000036956	165.8259346	1.192780999	0.000344	0.007313	289.4681	189.4422	213.3151	96.64532	94.44725	111.6377
ENSSSCG00000038778	58.46867967	-1.648020575	0.000345	0.007313	30.77402	30.11645	23.92319	97.64166	95.50846	72.84831
ENSSSCG00000007925	118.4776683	1.520378881	0.000354	0.007481	204.8395	102.0073	220.2927	81.70016	50.93784	51.08842
ENSSSCG00000029408	719.6180434	-1.163292339	0.000356	0.007505	368.3265	626.6164	337.9151	916.636	733.2927	1334.922
ENSSSCG00000004318	853.7348421	-1.255971377	0.000358	0.007523	252.9239	765.5407	493.4158	1115.905	1105.776	1388.848
ENSSSCG00000029449	142.3902688	2.311317918	0.000358	0.007523	187.5292	492.5497	30.90079	41.84643	63.6723	37.84328
ENSSSCG00000013885	48.64812991	1.940113068	0.00036	0.007552	90.39867	48.57492	92.70236	19.92687	13.79567	26.49029
ENSSSCG00000007146	373.125888	1.301716049	0.000361	0.007571	677.0283	370.1409	545.2494	267.02	261.0564	118.2602
ENSSSCG00000025499	398.145901	1.268948806	0.000366	0.00766	848.2088	319.623	520.3294	220.1919	269.5461	210.9763

ENSSSCG00000013601	1035.575126	1.072630927	0.000369	0.007704	1478.114	890.864	1842.086	625.7037	794.8426	581.8404
ENSSSCG00000022343	8946.041847	1.047602576	0.00038	0.0079	14501.29	7246.406	14427.68	5138.143	7066.565	5296.166
ENSSSCG00000039633	6.668177203	6.179865737	0.000381	0.007917	17.31038	8.743485	13.95519	0	0	0
ENSSSCG00000032768	491.3471818	1.239428083	0.000382	0.007921	771.2738	397.3428	902.1037	257.0566	389.4623	230.844
ENSSSCG00000017311	117.4371672	1.365236981	0.000387	0.008005	205.8012	125.3233	176.4335	49.81717	75.34556	71.90222
ENSSSCG00000004233	9801.64665	1.085225103	0.00039	0.008066	18657.71	8138.242	13174.7	5334.423	7774.388	5730.418
ENSSSCG00000001252	229.6338	1.169559074	0.000393	0.008112	344.2843	315.737	294.0559	155.4296	92.32484	175.9712
ENSSSCG00000010132	3644.632091	1.269855508	0.000394	0.008113	5413.342	2242.218	7801.951	2243.765	2249.755	1916.762
ENSSSCG00000013119	166.0253194	1.430509917	0.000394	0.008113	282.7363	251.6181	192.3823	134.5064	48.81543	86.09345
ENSSSCG00000002857	71.0949723	-1.818573364	0.000403	0.008273	54.81621	21.37296	17.94239	117.5685	123.0998	91.76994
ENSSSCG00000039894	4140.005908	1.068733319	0.000404	0.008278	7229.008	3227.318	6364.566	3116.562	2485.342	2417.239
ENSSSCG00000023527	302.6606006	1.348234895	0.000407	0.008319	561.6258	208.8721	533.2878	140.4844	199.5066	172.1869
ENSSSCG00000010447	3950.189973	-1.066195005	0.000409	0.008341	1731.038	2378.228	3551.597	5639.304	3965.723	6435.249
ENSSSCG00000016367	963.9635071	1.097982079	0.000409	0.008341	1778.161	766.5122	1397.513	650.6123	565.6223	625.3601
ENSSSCG00000012018	192.9718082	-1.207752885	0.000412	0.008393	84.62854	147.6677	117.6224	292.925	212.241	302.7462
ENSSSCG00000014431	491.5049423	1.317855941	0.000414	0.008419	932.8373	324.4804	847.2797	265.0274	343.8304	235.5744
ENSSSCG00000037376	3559.970415	1.2468747	0.000417	0.008457	4343.945	4585.472	6098.42	3563.921	1297.854	1470.211
ENSSSCG00000003064	244.9198739	1.305480041	0.000422	0.008539	388.5219	204.9862	452.547	152.4406	169.7928	101.2308
ENSSSCG00000006296	169.005258	1.246203583	0.000423	0.008547	317.357	179.7272	216.3055	115.5758	83.8352	101.2308
ENSSSCG00000020813	531.0954293	1.136468838	0.000425	0.008564	1017.466	473.1197	699.7533	398.5374	276.9745	320.7218
ENSSSCG00000024108	2368.471682	1.21599878	0.000432	0.008692	4722.85	1644.747	3566.549	1501.49	1686.255	1088.94
ENSSSCG00000040918	71.54183075	-1.989969245	0.000435	0.008745	46.16102	30.11645	9.967996	166.3894	101.8757	74.74047
ENSSSCG00000007995	254.9189153	1.480187876	0.000441	0.00884	502.0011	160.2972	463.5118	160.4113	149.6299	93.66211
ENSSSCG00000016239	17.27052386	3.397955135	0.000441	0.008842	24.0422	44.68892	25.91679	0	4.24482	4.73041
ENSSSCG00000031216	248.750824	-1.412104907	0.000445	0.0089	66.35647	211.7866	129.5839	388.574	278.0357	418.1682
ENSSSCG00000037387	363.2624114	1.224742469	0.000455	0.009062	619.3271	267.162	639.9454	219.1956	223.9143	210.0302
ENSSSCG00000012508	72.70998928	-1.833644247	0.000456	0.009067	34.62077	33.03094	27.91039	45.8318	145.3851	149.4809
ENSSSCG00000017723	363.1174593	1.340181872	0.000456	0.009067	711.6491	488.6637	361.8383	193.2906	116.7326	306.5305
ENSSSCG00000008842	1027.0112	-1.399393129	0.000458	0.009099	877.0594	330.3094	486.4382	729.3234	1753.111	1985.826
ENSSSCG00000005645	419.0805987	1.046221546	0.00046	0.009123	627.0206	406.0863	660.8781	305.8774	296.0762	218.5449
ENSSSCG00000028567	2655.659978	-1.126913252	0.000467	0.009239	1113.635	2043.061	1848.066	3434.396	2380.283	5114.519
ENSSSCG00000011482	256.4224607	-1.02221713	0.000472	0.009312	174.0655	170.0122	163.4751	417.4679	326.8512	286.6628
ENSSSCG00000015426	356.8973366	-1.500108085	0.000475	0.009353	80.78179	342.9389	135.5647	507.1388	568.8059	506.1538
ENSSSCG00000035032	6.657420071	6.17766453	0.000477	0.009386	21.15714	5.82899	12.95839	0	0	0
ENSSSCG00000002385	591.1293362	-1.169350411	0.000484	0.009479	204.8395	431.3453	455.5374	1034.205	696.1505	724.6987

ENSSSCG00000006888	27.26957871	-2.642882861	0.000491	0.009591	5.770128	2.914495	13.95519	32.87933	37.14218	70.95614
ENSSSCG00000031033	35.50906157	-2.293408706	0.000494	0.00964	5.770128	21.37296	8.971197	63.76598	40.32579	72.84831
ENSSSCG00000012943	349.6400958	1.061324776	0.000497	0.009689	477.9589	334.1954	606.0542	204.2504	248.322	227.0597
ENSSSCG00000001720	568.3375845	-1.221056001	0.000501	0.009737	208.6863	468.2622	346.8863	739.2869	556.0715	1090.832
ENSSSCG00000002680	202.3133035	1.570862404	0.000501	0.009737	378.9051	123.3803	405.6974	89.67091	144.3239	71.90222
ENSSSCG00000006811	210.8892306	1.378071159	0.000504	0.009778	293.3148	168.0692	452.547	137.4954	88.08002	125.8289
ENSSSCG00000035563	56.02108445	1.725344863	0.000504	0.009779	100.0155	55.37541	102.6704	29.8903	25.46892	22.70597
ENSSSCG00000005301	1005.062731	1.166882858	0.000507	0.009817	1696.418	786.9137	1688.579	527.0657	830.9236	500.4773
ENSSSCG00000014049	314.0481866	1.248582818	0.000511	0.009874	493.3459	249.6751	583.1278	140.4844	213.3022	204.3537
ENSSSCG00000004652	4149.473861	-1.042746036	0.000516	0.009921	2028.2	4126.925	1980.641	5526.717	5883.321	5351.039
ENSSSCG00000012978	187.054803	1.564996007	0.000517	0.009921	400.0622	109.7793	328.9439	121.5539	89.14123	72.84831
ENSSSCG00000018044	43.50385738	2.047969043	0.000518	0.009921	82.70517	79.66286	47.84638	27.89762	10.61205	12.29906
ENSSSCG00000029305	141.9976012	-1.437285942	0.000518	0.009921	50.96946	61.2044	117.6224	219.1956	252.5668	150.427
ENSSSCG00000034723	342.3895501	1.356851883	0.000515	0.009921	672.2199	233.1596	572.163	251.0786	150.6911	175.0252
ENSSSCG00000031367	15.54168987	-3.261592846	0.000519	0.009942	0.961688	4.857492	2.990399	20.92321	36.08097	27.43638
ENSSSCG00000036008	102.8402364	2.444300763	0.000521	0.009957	100.9772	139.8958	280.1007	16.93784	75.34556	3.784328
ENSSSCG00000033998	401.0618081	1.222730173	0.000523	0.009973	633.7524	559.5831	491.4222	361.6727	134.773	225.1675

Table S2. The annotation of identified lncRNAs with gtf format.

1	StringTie	transcrip	824024	825885	.	+	.	transcript_	MSTRG.49.4;	gene_id	MSTRG.49;
1	StringTie	exon	824024	824270	.	+	.	transcript_	MSTRG.49.4;	gene_id	MSTRG.49;
1	StringTie	exon	825375	825496	.	+	.	transcript_	MSTRG.49.4;	gene_id	MSTRG.49;
1	StringTie	exon	825769	825885	.	+	.	transcript_	MSTRG.49.4;	gene_id	MSTRG.49;
1	StringTie	transcrip	1698902	1710066	.	+	.	transcript_	MSTRG.67.1;	gene_id	MSTRG.67;
1	StringTie	exon	1698902	1698946	.	+	.	transcript_	MSTRG.67.1;	gene_id	MSTRG.67;
1	StringTie	exon	1707172	1710066	.	+	.	transcript_	MSTRG.67.1;	gene_id	MSTRG.67;
1	StringTie	transcrip	1698913	1709664	.	+	.	transcript_	MSTRG.67.2;	gene_id	MSTRG.67;
1	StringTie	exon	1698913	1698999	.	+	.	transcript_	MSTRG.67.2;	gene_id	MSTRG.67;
1	StringTie	exon	1707172	1709664	.	+	.	transcript_	MSTRG.67.2;	gene_id	MSTRG.67;
1	StringTie	transcrip	4321011	4330835	.	+	.	transcript_	MSTRG.86.1;	gene_id	MSTRG.86;
1	StringTie	exon	4321011	4321192	.	+	.	transcript_	MSTRG.86.1;	gene_id	MSTRG.86;
1	StringTie	exon	4322916	4330835	.	+	.	transcript_	MSTRG.86.1;	gene_id	MSTRG.86;
1	StringTie	transcrip	4885871	4888328	.	+	.	transcript_	MSTRG.93.1;	gene_id	MSTRG.93;
1	StringTie	exon	4885871	4887145	.	+	.	transcript_	MSTRG.93.1;	gene_id	MSTRG.93;
1	StringTie	exon	4888059	4888328	.	+	.	transcript_	MSTRG.93.1;	gene_id	MSTRG.93;
1	StringTie	transcrip	12122655	12126138	.	+	.	transcript_	MSTRG.224.1;	gene_id	MSTRG.224;
1	StringTie	exon	12122655	12126138	.	+	.	transcript_	MSTRG.224.1;	gene_id	MSTRG.224;
1	StringTie	transcrip	12122900	12124707	.	+	.	transcript_	MSTRG.224.2;	gene_id	MSTRG.224;
1	StringTie	exon	12122900	12123333	.	+	.	transcript_	MSTRG.224.2;	gene_id	MSTRG.224;
1	StringTie	exon	12124562	12124707	.	+	.	transcript_	MSTRG.224.2;	gene_id	MSTRG.224;
1	StringTie	transcrip	17511780	17514478	.	+	.	transcript_	MSTRG.326.1;	gene_id	MSTRG.326;
1	StringTie	exon	17511780	17512929	.	+	.	transcript_	MSTRG.326.1;	gene_id	MSTRG.326;
1	StringTie	exon	17514014	17514478	.	+	.	transcript_	MSTRG.326.1;	gene_id	MSTRG.326;
1	StringTie	transcrip	28229063	28233940	.	+	.	transcript_	MSTRG.608.1;	gene_id	MSTRG.608;
1	StringTie	exon	28229063	28230164	.	+	.	transcript_	MSTRG.608.1;	gene_id	MSTRG.608;
1	StringTie	exon	28230834	28231034	.	+	.	transcript_	MSTRG.608.1;	gene_id	MSTRG.608;
1	StringTie	exon	28233091	28233940	.	+	.	transcript_	MSTRG.608.1;	gene_id	MSTRG.608;
1	StringTie	transcrip	29922876	29927248	.	+	.	transcript_	MSTRG.628.1;	gene_id	MSTRG.628;
1	StringTie	exon	29922876	29923063	.	+	.	transcript_	MSTRG.628.1;	gene_id	MSTRG.628;
1	StringTie	exon	29926724	29927248	.	+	.	transcript_	MSTRG.628.1;	gene_id	MSTRG.628;
1	StringTie	transcrip	30390985	30448644	.	+	.	transcript_	MSTRG.638.1;	gene_id	MSTRG.638;
1	StringTie	exon	30390985	30391033	.	+	.	transcript_	MSTRG.638.1;	gene_id	MSTRG.638;
1	StringTie	exon	30444609	30448644	.	+	.	transcript_	MSTRG.638.1;	gene_id	MSTRG.638;

1	StringTie	transcrip	30776390	30787599	.	+	.	transcript_	MSTRG.635.1;	gene_id	MSTRG.635;
1	StringTie	exon	30776390	30776606	.	+	.	transcript_	MSTRG.635.1;	gene_id	MSTRG.635;
1	StringTie	exon	30780720	30787599	.	+	.	transcript_	MSTRG.635.1;	gene_id	MSTRG.635;
1	StringTie	transcrip	44257834	44261820	.	+	.	transcript_	MSTRG.851.1;	gene_id	MSTRG.851;
1	StringTie	exon	44257834	44257932	.	+	.	transcript_	MSTRG.851.1;	gene_id	MSTRG.851;
1	StringTie	exon	44259743	44261820	.	+	.	transcript_	MSTRG.851.1;	gene_id	MSTRG.851;
1	StringTie	transcrip	56160835	56178673	.	+	.	transcript_	MSTRG.1016.1	gene_id	MSTRG.1016;
1	StringTie	exon	56160835	56160882	.	+	.	transcript_	MSTRG.1016.1	gene_id	MSTRG.1016;
1	StringTie	exon	56178141	56178673	.	+	.	transcript_	MSTRG.1016.1	gene_id	MSTRG.1016;
1	StringTie	transcrip	56360451	56362017	.	+	.	transcript_	MSTRG.1020.1	gene_id	MSTRG.1020;
1	StringTie	exon	56360451	56360606	.	+	.	transcript_	MSTRG.1020.1	gene_id	MSTRG.1020;
1	StringTie	exon	56361672	56362017	.	+	.	transcript_	MSTRG.1020.1	gene_id	MSTRG.1020;
1	StringTie	transcrip	72085711	72093501	.	+	.	transcript_	MSTRG.1208.1	gene_id	MSTRG.1208;
1	StringTie	exon	72085711	72085812	.	+	.	transcript_	MSTRG.1208.1	gene_id	MSTRG.1208;
1	StringTie	exon	72093355	72093501	.	+	.	transcript_	MSTRG.1208.1	gene_id	MSTRG.1208;
1	StringTie	transcrip	76245833	76248251	.	+	.	transcript_	MSTRG.1294.1	gene_id	MSTRG.1294;
1	StringTie	exon	76245833	76248014	.	+	.	transcript_	MSTRG.1294.1	gene_id	MSTRG.1294;
1	StringTie	exon	76248035	76248251	.	+	.	transcript_	MSTRG.1294.1	gene_id	MSTRG.1294;
1	StringTie	transcrip	76623298	76660599	.	+	.	transcript_	MSTRG.1304.1	gene_id	MSTRG.1304;
1	StringTie	exon	76623298	76623338	.	+	.	transcript_	MSTRG.1304.1	gene_id	MSTRG.1304;
1	StringTie	exon	76660165	76660599	.	+	.	transcript_	MSTRG.1304.1	gene_id	MSTRG.1304;
1	StringTie	transcrip	77193637	77210343	.	+	.	transcript_	MSTRG.1333.1	gene_id	MSTRG.1333;
1	StringTie	exon	77193637	77194996	.	+	.	transcript_	MSTRG.1333.1	gene_id	MSTRG.1333;
1	StringTie	exon	77210052	77210343	.	+	.	transcript_	MSTRG.1333.1	gene_id	MSTRG.1333;
1	StringTie	transcrip	77410264	77414849	.	+	.	transcript_	MSTRG.1335.1	gene_id	MSTRG.1335;
1	StringTie	exon	77410264	77412285	.	+	.	transcript_	MSTRG.1335.1	gene_id	MSTRG.1335;
1	StringTie	exon	77413971	77414849	.	+	.	transcript_	MSTRG.1335.1	gene_id	MSTRG.1335;
1	StringTie	transcrip	77608027	77609251	.	+	.	transcript_	MSTRG.1331.1	gene_id	MSTRG.1331;
1	StringTie	exon	77608027	77608354	.	+	.	transcript_	MSTRG.1331.1	gene_id	MSTRG.1331;
1	StringTie	exon	77608718	77608804	.	+	.	transcript_	MSTRG.1331.1	gene_id	MSTRG.1331;
1	StringTie	exon	77609123	77609251	.	+	.	transcript_	MSTRG.1331.1	gene_id	MSTRG.1331;
1	StringTie	transcrip	78149331	78152555	.	+	.	transcript_	MSTRG.1356.1	gene_id	MSTRG.1356;
1	StringTie	exon	78149331	78149440	.	+	.	transcript_	MSTRG.1356.1	gene_id	MSTRG.1356;
1	StringTie	exon	78151636	78152555	.	+	.	transcript_	MSTRG.1356.1	gene_id	MSTRG.1356;
1	StringTie	transcrip	94347889	94349382	.	+	.	transcript_	MSTRG.1514.1	gene_id	MSTRG.1514;

1	StringTie	exon	94347889	94349289	.	+	.	transcript_	MSTRG.1514.1	gene_id	MSTRG.1514;
1	StringTie	exon	94349318	94349382	.	+	.	transcript_	MSTRG.1514.1	gene_id	MSTRG.1514;
1	StringTie	transcrip	100540201	100541824	.	+	.	transcript_	MSTRG.1598.1	gene_id	MSTRG.1598;
1	StringTie	exon	100540201	100541382	.	+	.	transcript_	MSTRG.1598.1	gene_id	MSTRG.1598;
1	StringTie	exon	100541673	100541824	.	+	.	transcript_	MSTRG.1598.1	gene_id	MSTRG.1598;
1	StringTie	transcrip	106898615	106901503	.	+	.	transcript_	MSTRG.1640.1	gene_id	MSTRG.1640;
1	StringTie	exon	106898615	106898706	.	+	.	transcript_	MSTRG.1640.1	gene_id	MSTRG.1640;
1	StringTie	exon	106898808	106898954	.	+	.	transcript_	MSTRG.1640.1	gene_id	MSTRG.1640;
1	StringTie	exon	106901457	106901503	.	+	.	transcript_	MSTRG.1640.1	gene_id	MSTRG.1640;
1	StringTie	transcrip	106928511	106963920	.	+	.	transcript_	MSTRG.1648.1	gene_id	MSTRG.1648;
1	StringTie	exon	106928511	106928587	.	+	.	transcript_	MSTRG.1648.1	gene_id	MSTRG.1648;
1	StringTie	exon	106963094	106963920	.	+	.	transcript_	MSTRG.1648.1	gene_id	MSTRG.1648;
1	StringTie	transcrip	106933231	106964010	.	+	.	transcript_	MSTRG.1648.2	gene_id	MSTRG.1648;
1	StringTie	exon	106933231	106933434	.	+	.	transcript_	MSTRG.1648.2	gene_id	MSTRG.1648;
1	StringTie	exon	106963094	106964010	.	+	.	transcript_	MSTRG.1648.2	gene_id	MSTRG.1648;
1	StringTie	transcrip	106952799	106963943	.	+	.	transcript_	MSTRG.1648.3	gene_id	MSTRG.1648;
1	StringTie	exon	106952799	106952831	.	+	.	transcript_	MSTRG.1648.3	gene_id	MSTRG.1648;
1	StringTie	exon	106963094	106963943	.	+	.	transcript_	MSTRG.1648.3	gene_id	MSTRG.1648;
1	StringTie	transcrip	106953206	106964010	.	+	.	transcript_	MSTRG.1648.4	gene_id	MSTRG.1648;
1	StringTie	exon	106953206	106953452	.	+	.	transcript_	MSTRG.1648.4	gene_id	MSTRG.1648;
1	StringTie	exon	106963094	106964010	.	+	.	transcript_	MSTRG.1648.4	gene_id	MSTRG.1648;
1	StringTie	transcrip	108436435	108440266	.	+	.	transcript_	MSTRG.1681.1	gene_id	MSTRG.1681;
1	StringTie	exon	108436435	108438315	.	+	.	transcript_	MSTRG.1681.1	gene_id	MSTRG.1681;
1	StringTie	exon	108438339	108440266	.	+	.	transcript_	MSTRG.1681.1	gene_id	MSTRG.1681;
1	StringTie	transcrip	109501888	109503695	.	+	.	transcript_	MSTRG.1699.1	gene_id	MSTRG.1699;
1	StringTie	exon	109501888	109501921	.	+	.	transcript_	MSTRG.1699.1	gene_id	MSTRG.1699;
1	StringTie	exon	109503405	109503695	.	+	.	transcript_	MSTRG.1699.1	gene_id	MSTRG.1699;
1	StringTie	transcrip	114266958	114297706	.	+	.	transcript_	MSTRG.1906.1	gene_id	MSTRG.1906;
1	StringTie	exon	114266958	114267022	.	+	.	transcript_	MSTRG.1906.1	gene_id	MSTRG.1906;
1	StringTie	exon	114288512	114288636	.	+	.	transcript_	MSTRG.1906.1	gene_id	MSTRG.1906;
1	StringTie	exon	114289261	114289410	.	+	.	transcript_	MSTRG.1906.1	gene_id	MSTRG.1906;
1	StringTie	exon	114297196	114297706	.	+	.	transcript_	MSTRG.1906.1	gene_id	MSTRG.1906;
1	StringTie	transcrip	115753971	115824683	.	+	.	transcript_	MSTRG.1938.1	gene_id	MSTRG.1938;
1	StringTie	exon	115753971	115754005	.	+	.	transcript_	MSTRG.1938.1	gene_id	MSTRG.1938;
1	StringTie	exon	115823226	115824683	.	+	.	transcript_	MSTRG.1938.1	gene_id	MSTRG.1938;

1	StringTie	transcrip	123403207	123547751	.	+	.	transcript_	MSTRG.2057.1	gene_id	MSTRG.2057;
1	StringTie	exon	123403207	123403379	.	+	.	transcript_	MSTRG.2057.1	gene_id	MSTRG.2057;
1	StringTie	exon	123474792	123474880	.	+	.	transcript_	MSTRG.2057.1	gene_id	MSTRG.2057;
1	StringTie	exon	123475421	123475478	.	+	.	transcript_	MSTRG.2057.1	gene_id	MSTRG.2057;
1	StringTie	exon	123499743	123499833	.	+	.	transcript_	MSTRG.2057.1	gene_id	MSTRG.2057;
1	StringTie	exon	123509580	123509661	.	+	.	transcript_	MSTRG.2057.1	gene_id	MSTRG.2057;
1	StringTie	exon	123515508	123515620	.	+	.	transcript_	MSTRG.2057.1	gene_id	MSTRG.2057;
1	StringTie	exon	123534011	123534196	.	+	.	transcript_	MSTRG.2057.1	gene_id	MSTRG.2057;
1	StringTie	exon	123538658	123538746	.	+	.	transcript_	MSTRG.2057.1	gene_id	MSTRG.2057;
1	StringTie	exon	123547663	123547751	.	+	.	transcript_	MSTRG.2057.1	gene_id	MSTRG.2057;
1	StringTie	transcrip	123461094	123547743	.	+	.	transcript_	MSTRG.2057.2	gene_id	MSTRG.2057;
1	StringTie	exon	123461094	123461592	.	+	.	transcript_	MSTRG.2057.2	gene_id	MSTRG.2057;
1	StringTie	exon	123473645	123473726	.	+	.	transcript_	MSTRG.2057.2	gene_id	MSTRG.2057;
1	StringTie	exon	123474792	123474880	.	+	.	transcript_	MSTRG.2057.2	gene_id	MSTRG.2057;
1	StringTie	exon	123475421	123475478	.	+	.	transcript_	MSTRG.2057.2	gene_id	MSTRG.2057;
1	StringTie	exon	123499743	123499833	.	+	.	transcript_	MSTRG.2057.2	gene_id	MSTRG.2057;
1	StringTie	exon	123509580	123509661	.	+	.	transcript_	MSTRG.2057.2	gene_id	MSTRG.2057;
1	StringTie	exon	123515508	123515620	.	+	.	transcript_	MSTRG.2057.2	gene_id	MSTRG.2057;
1	StringTie	exon	123534011	123534196	.	+	.	transcript_	MSTRG.2057.2	gene_id	MSTRG.2057;
1	StringTie	exon	123538658	123538746	.	+	.	transcript_	MSTRG.2057.2	gene_id	MSTRG.2057;
1	StringTie	exon	123547663	123547743	.	+	.	transcript_	MSTRG.2057.2	gene_id	MSTRG.2057;
1	StringTie	transcrip	123461095	123548837	.	+	.	transcript_	MSTRG.2057.3	gene_id	MSTRG.2057;
1	StringTie	exon	123461095	123461592	.	+	.	transcript_	MSTRG.2057.3	gene_id	MSTRG.2057;
1	StringTie	exon	123473705	123473726	.	+	.	transcript_	MSTRG.2057.3	gene_id	MSTRG.2057;
1	StringTie	exon	123474792	123474880	.	+	.	transcript_	MSTRG.2057.3	gene_id	MSTRG.2057;
1	StringTie	exon	123475421	123475478	.	+	.	transcript_	MSTRG.2057.3	gene_id	MSTRG.2057;
1	StringTie	exon	123499743	123499833	.	+	.	transcript_	MSTRG.2057.3	gene_id	MSTRG.2057;
1	StringTie	exon	123509580	123509661	.	+	.	transcript_	MSTRG.2057.3	gene_id	MSTRG.2057;
1	StringTie	exon	123515508	123515620	.	+	.	transcript_	MSTRG.2057.3	gene_id	MSTRG.2057;
1	StringTie	exon	123534011	123534196	.	+	.	transcript_	MSTRG.2057.3	gene_id	MSTRG.2057;
1	StringTie	exon	123538658	123538746	.	+	.	transcript_	MSTRG.2057.3	gene_id	MSTRG.2057;
1	StringTie	exon	123547663	123548837	.	+	.	transcript_	MSTRG.2057.3	gene_id	MSTRG.2057;
1	StringTie	transcrip	123461484	123547743	.	+	.	transcript_	MSTRG.2057.5	gene_id	MSTRG.2057;
1	StringTie	exon	123461484	123461592	.	+	.	transcript_	MSTRG.2057.5	gene_id	MSTRG.2057;
1	StringTie	exon	123473705	123473726	.	+	.	transcript_	MSTRG.2057.5	gene_id	MSTRG.2057;

1	StringTie	exon	123474792	123474880	.	+	.	transcript_	MSTRG.2057.5	gene_id	MSTRG.2057;
1	StringTie	exon	123475421	123475478	.	+	.	transcript_	MSTRG.2057.5	gene_id	MSTRG.2057;
1	StringTie	exon	123509580	123509661	.	+	.	transcript_	MSTRG.2057.5	gene_id	MSTRG.2057;
1	StringTie	exon	123515508	123515620	.	+	.	transcript_	MSTRG.2057.5	gene_id	MSTRG.2057;
1	StringTie	exon	123534011	123534196	.	+	.	transcript_	MSTRG.2057.5	gene_id	MSTRG.2057;
1	StringTie	exon	123538658	123538746	.	+	.	transcript_	MSTRG.2057.5	gene_id	MSTRG.2057;
1	StringTie	exon	123547663	123547743	.	+	.	transcript_	MSTRG.2057.5	gene_id	MSTRG.2057;
1	StringTie	transcrip	123461484	123547743	.	+	.	transcript_	MSTRG.2057.4	gene_id	MSTRG.2057;
1	StringTie	exon	123461484	123461592	.	+	.	transcript_	MSTRG.2057.4	gene_id	MSTRG.2057;
1	StringTie	exon	123473651	123473726	.	+	.	transcript_	MSTRG.2057.4	gene_id	MSTRG.2057;
1	StringTie	exon	123474792	123474880	.	+	.	transcript_	MSTRG.2057.4	gene_id	MSTRG.2057;
1	StringTie	exon	123475421	123475478	.	+	.	transcript_	MSTRG.2057.4	gene_id	MSTRG.2057;
1	StringTie	exon	123499743	123499833	.	+	.	transcript_	MSTRG.2057.4	gene_id	MSTRG.2057;
1	StringTie	exon	123509580	123509661	.	+	.	transcript_	MSTRG.2057.4	gene_id	MSTRG.2057;
1	StringTie	exon	123515508	123515620	.	+	.	transcript_	MSTRG.2057.4	gene_id	MSTRG.2057;
1	StringTie	exon	123534011	123534196	.	+	.	transcript_	MSTRG.2057.4	gene_id	MSTRG.2057;
1	StringTie	exon	123538658	123538746	.	+	.	transcript_	MSTRG.2057.4	gene_id	MSTRG.2057;
1	StringTie	exon	123547663	123547743	.	+	.	transcript_	MSTRG.2057.4	gene_id	MSTRG.2057;
1	StringTie	transcrip	123471966	123504531	.	+	.	transcript_	MSTRG.2057.6	gene_id	MSTRG.2057;
1	StringTie	exon	123471966	123473726	.	+	.	transcript_	MSTRG.2057.6	gene_id	MSTRG.2057;
1	StringTie	exon	123474792	123474880	.	+	.	transcript_	MSTRG.2057.6	gene_id	MSTRG.2057;
1	StringTie	exon	123475421	123475478	.	+	.	transcript_	MSTRG.2057.6	gene_id	MSTRG.2057;
1	StringTie	exon	123499743	123499833	.	+	.	transcript_	MSTRG.2057.6	gene_id	MSTRG.2057;
1	StringTie	exon	123504165	123504531	.	+	.	transcript_	MSTRG.2057.6	gene_id	MSTRG.2057;
1	StringTie	transcrip	123499769	123547743	.	+	.	transcript_	MSTRG.2057.7	gene_id	MSTRG.2057;
1	StringTie	exon	123499769	123499833	.	+	.	transcript_	MSTRG.2057.7	gene_id	MSTRG.2057;
1	StringTie	exon	123509580	123509661	.	+	.	transcript_	MSTRG.2057.7	gene_id	MSTRG.2057;
1	StringTie	exon	123515508	123515620	.	+	.	transcript_	MSTRG.2057.7	gene_id	MSTRG.2057;
1	StringTie	exon	123538658	123538746	.	+	.	transcript_	MSTRG.2057.7	gene_id	MSTRG.2057;
1	StringTie	exon	123547663	123547743	.	+	.	transcript_	MSTRG.2057.7	gene_id	MSTRG.2057;
1	StringTie	transcrip	127937122	127938184	.	+	.	transcript_	MSTRG.2126.1	gene_id	MSTRG.2126;
1	StringTie	exon	127937122	127937173	.	+	.	transcript_	MSTRG.2126.1	gene_id	MSTRG.2126;
1	StringTie	exon	127937414	127937489	.	+	.	transcript_	MSTRG.2126.1	gene_id	MSTRG.2126;
1	StringTie	exon	127937984	127938184	.	+	.	transcript_	MSTRG.2126.1	gene_id	MSTRG.2126;
1	StringTie	transcrip	131428583	131429197	.	+	.	transcript_	MSTRG.2312.1	gene_id	MSTRG.2312;

1	StringTie	exon	131428583	131428647	.	+	.	transcript_	MSTRG.2312.1	gene_id	MSTRG.2312;
1	StringTie	exon	131428694	131429197	.	+	.	transcript_	MSTRG.2312.1	gene_id	MSTRG.2312;
1	StringTie	transcrip	137308442	137319564	.	+	.	transcript_	MSTRG.2406.1	gene_id	MSTRG.2406;
1	StringTie	exon	137308442	137308482	.	+	.	transcript_	MSTRG.2406.1	gene_id	MSTRG.2406;
1	StringTie	exon	137316029	137316081	.	+	.	transcript_	MSTRG.2406.1	gene_id	MSTRG.2406;
1	StringTie	exon	137319328	137319564	.	+	.	transcript_	MSTRG.2406.1	gene_id	MSTRG.2406;
1	StringTie	transcrip	143506197	143508610	.	+	.	transcript_	MSTRG.2533.1	gene_id	MSTRG.2533;
1	StringTie	exon	143506197	143506218	.	+	.	transcript_	MSTRG.2533.1	gene_id	MSTRG.2533;
1	StringTie	exon	143507642	143508610	.	+	.	transcript_	MSTRG.2533.1	gene_id	MSTRG.2533;
1	StringTie	transcrip	156735451	156747020	.	+	.	transcript_	MSTRG.2656.1	gene_id	MSTRG.2656;
1	StringTie	exon	156735451	156735645	.	+	.	transcript_	MSTRG.2656.1	gene_id	MSTRG.2656;
1	StringTie	exon	156743825	156743916	.	+	.	transcript_	MSTRG.2656.1	gene_id	MSTRG.2656;
1	StringTie	exon	156746015	156747020	.	+	.	transcript_	MSTRG.2656.1	gene_id	MSTRG.2656;
1	StringTie	transcrip	156735523	156747530	.	+	.	transcript_	MSTRG.2656.2	gene_id	MSTRG.2656;
1	StringTie	exon	156735523	156735645	.	+	.	transcript_	MSTRG.2656.2	gene_id	MSTRG.2656;
1	StringTie	exon	156744486	156744657	.	+	.	transcript_	MSTRG.2656.2	gene_id	MSTRG.2656;
1	StringTie	exon	156746015	156747530	.	+	.	transcript_	MSTRG.2656.2	gene_id	MSTRG.2656;
1	StringTie	transcrip	158594579	158611713	.	+	.	transcript_	MSTRG.2665.1	gene_id	MSTRG.2665;
1	StringTie	exon	158594579	158594707	.	+	.	transcript_	MSTRG.2665.1	gene_id	MSTRG.2665;
1	StringTie	exon	158610810	158610891	.	+	.	transcript_	MSTRG.2665.1	gene_id	MSTRG.2665;
1	StringTie	exon	158611384	158611713	.	+	.	transcript_	MSTRG.2665.1	gene_id	MSTRG.2665;
1	StringTie	transcrip	163251698	163274835	.	+	.	transcript_	MSTRG.2747.1	gene_id	MSTRG.2747;
1	StringTie	exon	163251698	163252147	.	+	.	transcript_	MSTRG.2747.1	gene_id	MSTRG.2747;
1	StringTie	exon	163274795	163274835	.	+	.	transcript_	MSTRG.2747.1	gene_id	MSTRG.2747;
1	StringTie	transcrip	163894365	163952074	.	+	.	transcript_	MSTRG.2768.1	gene_id	MSTRG.2768;
1	StringTie	exon	163894365	163894606	.	+	.	transcript_	MSTRG.2768.1	gene_id	MSTRG.2768;
1	StringTie	exon	163951697	163952074	.	+	.	transcript_	MSTRG.2768.1	gene_id	MSTRG.2768;
1	StringTie	transcrip	180015227	180023529	.	+	.	transcript_	MSTRG.2984.2	gene_id	MSTRG.2984;
1	StringTie	exon	180015227	180015522	.	+	.	transcript_	MSTRG.2984.2	gene_id	MSTRG.2984;
1	StringTie	exon	180020346	180020439	.	+	.	transcript_	MSTRG.2984.2	gene_id	MSTRG.2984;
1	StringTie	exon	180022727	180023529	.	+	.	transcript_	MSTRG.2984.2	gene_id	MSTRG.2984;
1	StringTie	transcrip	180015227	180023529	.	+	.	transcript_	MSTRG.2984.1	gene_id	MSTRG.2984;
1	StringTie	exon	180015227	180015522	.	+	.	transcript_	MSTRG.2984.1	gene_id	MSTRG.2984;
1	StringTie	exon	180020346	180020439	.	+	.	transcript_	MSTRG.2984.1	gene_id	MSTRG.2984;
1	StringTie	exon	180022765	180023529	.	+	.	transcript_	MSTRG.2984.1	gene_id	MSTRG.2984;

1	StringTie	transcrip	183863604	183868043	.	+	.	transcript_	MSTRG.3076.1	gene_id	MSTRG.3076;
1	StringTie	exon	183863604	183863629	.	+	.	transcript_	MSTRG.3076.1	gene_id	MSTRG.3076;
1	StringTie	exon	183867805	183868043	.	+	.	transcript_	MSTRG.3076.1	gene_id	MSTRG.3076;
1	StringTie	transcrip	189621835	189630724	.	+	.	transcript_	MSTRG.3199.1	gene_id	MSTRG.3199;
1	StringTie	exon	189621835	189623205	.	+	.	transcript_	MSTRG.3199.1	gene_id	MSTRG.3199;
1	StringTie	exon	189627061	189630724	.	+	.	transcript_	MSTRG.3199.1	gene_id	MSTRG.3199;
1	StringTie	transcrip	189624750	189629280	.	+	.	transcript_	MSTRG.3199.2	gene_id	MSTRG.3199;
1	StringTie	exon	189624750	189625064	.	+	.	transcript_	MSTRG.3199.2	gene_id	MSTRG.3199;
1	StringTie	exon	189627061	189629280	.	+	.	transcript_	MSTRG.3199.2	gene_id	MSTRG.3199;
1	StringTie	transcrip	190167521	190175130	.	+	.	transcript_	MSTRG.3215.1	gene_id	MSTRG.3215;
1	StringTie	exon	190167521	190170132	.	+	.	transcript_	MSTRG.3215.1	gene_id	MSTRG.3215;
1	StringTie	exon	190171521	190171644	.	+	.	transcript_	MSTRG.3215.1	gene_id	MSTRG.3215;
1	StringTie	exon	190172038	190175130	.	+	.	transcript_	MSTRG.3215.1	gene_id	MSTRG.3215;
1	StringTie	transcrip	194017899	194030457	.	+	.	transcript_	MSTRG.3334.1	gene_id	MSTRG.3334;
1	StringTie	exon	194017899	194018205	.	+	.	transcript_	MSTRG.3334.1	gene_id	MSTRG.3334;
1	StringTie	exon	194028831	194030457	.	+	.	transcript_	MSTRG.3334.1	gene_id	MSTRG.3334;
1	StringTie	transcrip	201333205	201334563	.	+	.	transcript_	MSTRG.3382.1	gene_id	MSTRG.3382;
1	StringTie	exon	201333205	201333911	.	+	.	transcript_	MSTRG.3382.1	gene_id	MSTRG.3382;
1	StringTie	exon	201334495	201334563	.	+	.	transcript_	MSTRG.3382.1	gene_id	MSTRG.3382;
1	StringTie	transcrip	201353695	201410891	.	+	.	transcript_	MSTRG.3385.1	gene_id	MSTRG.3385;
1	StringTie	exon	201353695	201353845	.	+	.	transcript_	MSTRG.3385.1	gene_id	MSTRG.3385;
1	StringTie	exon	201407640	201407764	.	+	.	transcript_	MSTRG.3385.1	gene_id	MSTRG.3385;
1	StringTie	exon	201409087	201409149	.	+	.	transcript_	MSTRG.3385.1	gene_id	MSTRG.3385;
1	StringTie	exon	201410660	201410891	.	+	.	transcript_	MSTRG.3385.1	gene_id	MSTRG.3385;
1	StringTie	transcrip	201356660	201369854	.	+	.	transcript_	MSTRG.3387.1	gene_id	MSTRG.3387;
1	StringTie	exon	201356660	201356916	.	+	.	transcript_	MSTRG.3387.1	gene_id	MSTRG.3387;
1	StringTie	exon	201367581	201368800	.	+	.	transcript_	MSTRG.3387.1	gene_id	MSTRG.3387;
1	StringTie	exon	201369785	201369854	.	+	.	transcript_	MSTRG.3387.1	gene_id	MSTRG.3387;
1	StringTie	transcrip	201413086	201476397	.	+	.	transcript_	MSTRG.3401.1	gene_id	MSTRG.3401;
1	StringTie	exon	201413086	201413190	.	+	.	transcript_	MSTRG.3401.1	gene_id	MSTRG.3401;
1	StringTie	exon	201426390	201426491	.	+	.	transcript_	MSTRG.3401.1	gene_id	MSTRG.3401;
1	StringTie	exon	201475715	201475999	.	+	.	transcript_	MSTRG.3401.1	gene_id	MSTRG.3401;
1	StringTie	exon	201476161	201476397	.	+	.	transcript_	MSTRG.3401.1	gene_id	MSTRG.3401;
1	StringTie	transcrip	201443402	201456551	.	+	.	transcript_	MSTRG.3404.1	gene_id	MSTRG.3404;
1	StringTie	exon	201443402	201443727	.	+	.	transcript_	MSTRG.3404.1	gene_id	MSTRG.3404;

1	StringTie	exon	201443889	201444122	.	+	.	transcript_	MSTRG.3404.1	gene_id	MSTRG.3404;
1	StringTie	exon	201456463	201456551	.	+	.	transcript_	MSTRG.3404.1	gene_id	MSTRG.3404;
1	StringTie	transcrip	201445623	201456551	.	+	.	transcript_	MSTRG.3404.2	gene_id	MSTRG.3404;
1	StringTie	exon	201445623	201445736	.	+	.	transcript_	MSTRG.3404.2	gene_id	MSTRG.3404;
1	StringTie	exon	201456463	201456551	.	+	.	transcript_	MSTRG.3404.2	gene_id	MSTRG.3404;
1	StringTie	transcrip	201515945	201532745	.	+	.	transcript_	MSTRG.3410.1	gene_id	MSTRG.3410;
1	StringTie	exon	201515945	201515986	.	+	.	transcript_	MSTRG.3410.1	gene_id	MSTRG.3410;
1	StringTie	exon	201531010	201532745	.	+	.	transcript_	MSTRG.3410.1	gene_id	MSTRG.3410;
1	StringTie	transcrip	204383966	204413141	.	+	.	transcript_	MSTRG.3367.1	gene_id	MSTRG.3367;
1	StringTie	exon	204383966	204384122	.	+	.	transcript_	MSTRG.3367.1	gene_id	MSTRG.3367;
1	StringTie	exon	204412573	204413141	.	+	.	transcript_	MSTRG.3367.1	gene_id	MSTRG.3367;
1	StringTie	transcrip	208987016	209047433	.	+	.	transcript_	MSTRG.3505.3	gene_id	MSTRG.3505;
1	StringTie	exon	208987016	208987753	.	+	.	transcript_	MSTRG.3505.3	gene_id	MSTRG.3505;
1	StringTie	exon	209047213	209047433	.	+	.	transcript_	MSTRG.3505.3	gene_id	MSTRG.3505;
1	StringTie	transcrip	215807873	215978560	.	+	.	transcript_	MSTRG.3528.1	gene_id	MSTRG.3528;
1	StringTie	exon	215807873	215807961	.	+	.	transcript_	MSTRG.3528.1	gene_id	MSTRG.3528;
1	StringTie	exon	215811039	215811177	.	+	.	transcript_	MSTRG.3528.1	gene_id	MSTRG.3528;
1	StringTie	exon	215975867	215975975	.	+	.	transcript_	MSTRG.3528.1	gene_id	MSTRG.3528;
1	StringTie	exon	215978357	215978560	.	+	.	transcript_	MSTRG.3528.1	gene_id	MSTRG.3528;
1	StringTie	transcrip	222432517	222434362	.	+	.	transcript_	MSTRG.3698.5	gene_id	MSTRG.3698;
1	StringTie	exon	222432517	222432542	.	+	.	transcript_	MSTRG.3698.5	gene_id	MSTRG.3698;
1	StringTie	exon	222433248	222434362	.	+	.	transcript_	MSTRG.3698.5	gene_id	MSTRG.3698;
1	StringTie	transcrip	230312271	230316662	.	+	.	transcript_	MSTRG.3858.8	gene_id	MSTRG.3858;
1	StringTie	exon	230312271	230312438	.	+	.	transcript_	MSTRG.3858.8	gene_id	MSTRG.3858;
1	StringTie	exon	230316589	230316662	.	+	.	transcript_	MSTRG.3858.8	gene_id	MSTRG.3858;
1	StringTie	transcrip	235877498	235877821	.	+	.	transcript_	MSTRG.3882.1	gene_id	MSTRG.3882;
1	StringTie	exon	235877498	235877609	.	+	.	transcript_	MSTRG.3882.1	gene_id	MSTRG.3882;
1	StringTie	exon	235877722	235877821	.	+	.	transcript_	MSTRG.3882.1	gene_id	MSTRG.3882;
1	StringTie	transcrip	246728843	246753303	.	+	.	transcript_	MSTRG.4065.1	gene_id	MSTRG.4065;
1	StringTie	exon	246728843	246729077	.	+	.	transcript_	MSTRG.4065.1	gene_id	MSTRG.4065;
1	StringTie	exon	246753209	246753303	.	+	.	transcript_	MSTRG.4065.1	gene_id	MSTRG.4065;
1	StringTie	transcrip	250079982	250095437	.	+	.	transcript_	MSTRG.4136.1	gene_id	MSTRG.4136;
1	StringTie	exon	250079982	250080079	.	+	.	transcript_	MSTRG.4136.1	gene_id	MSTRG.4136;
1	StringTie	exon	250088692	250088836	.	+	.	transcript_	MSTRG.4136.1	gene_id	MSTRG.4136;
1	StringTie	exon	250089274	250089314	.	+	.	transcript_	MSTRG.4136.1	gene_id	MSTRG.4136;

1	StringTie	exon	250095025	250095437	.	+	.	transcript_	MSTRG.4136.1	gene_id	MSTRG.4136;
1	StringTie	transcrip	250123047	250123714	.	+	.	transcript_	MSTRG.4138.1	gene_id	MSTRG.4138;
1	StringTie	exon	250123047	250123482	.	+	.	transcript_	MSTRG.4138.1	gene_id	MSTRG.4138;
1	StringTie	exon	250123682	250123714	.	+	.	transcript_	MSTRG.4138.1	gene_id	MSTRG.4138;
1	StringTie	transcrip	251208475	251231319	.	+	.	transcript_	MSTRG.4144.2	gene_id	MSTRG.4144;
1	StringTie	exon	251208475	251208918	.	+	.	transcript_	MSTRG.4144.2	gene_id	MSTRG.4144;
1	StringTie	exon	251231270	251231319	.	+	.	transcript_	MSTRG.4144.2	gene_id	MSTRG.4144;
1	StringTie	transcrip	251208496	251239363	.	+	.	transcript_	MSTRG.4144.3	gene_id	MSTRG.4144;
1	StringTie	exon	251208496	251208814	.	+	.	transcript_	MSTRG.4144.3	gene_id	MSTRG.4144;
1	StringTie	exon	251239097	251239363	.	+	.	transcript_	MSTRG.4144.3	gene_id	MSTRG.4144;
1	StringTie	transcrip	251208714	251266306	.	+	.	transcript_	MSTRG.4144.4	gene_id	MSTRG.4144;
1	StringTie	exon	251208714	251208814	.	+	.	transcript_	MSTRG.4144.4	gene_id	MSTRG.4144;
1	StringTie	exon	251266174	251266306	.	+	.	transcript_	MSTRG.4144.4	gene_id	MSTRG.4144;
1	StringTie	transcrip	251799819	251809910	.	+	.	transcript_	MSTRG.4160.1	gene_id	MSTRG.4160;
1	StringTie	exon	251799819	251800103	.	+	.	transcript_	MSTRG.4160.1	gene_id	MSTRG.4160;
1	StringTie	exon	251800700	251809910	.	+	.	transcript_	MSTRG.4160.1	gene_id	MSTRG.4160;
1	StringTie	transcrip	252420661	252424771	.	+	.	transcript_	MSTRG.4187.1	gene_id	MSTRG.4187;
1	StringTie	exon	252420661	252420743	.	+	.	transcript_	MSTRG.4187.1	gene_id	MSTRG.4187;
1	StringTie	exon	252424256	252424771	.	+	.	transcript_	MSTRG.4187.1	gene_id	MSTRG.4187;
1	StringTie	transcrip	260885894	260908471	.	+	.	transcript_	MSTRG.4274.1	gene_id	MSTRG.4274;
1	StringTie	exon	260885894	260885997	.	+	.	transcript_	MSTRG.4274.1	gene_id	MSTRG.4274;
1	StringTie	exon	260907279	260908471	.	+	.	transcript_	MSTRG.4274.1	gene_id	MSTRG.4274;
1	StringTie	transcrip	261659720	261664181	.	+	.	transcript_	MSTRG.4300.4	gene_id	MSTRG.4300;
1	StringTie	exon	261659720	261659925	.	+	.	transcript_	MSTRG.4300.4	gene_id	MSTRG.4300;
1	StringTie	exon	261662504	261662627	.	+	.	transcript_	MSTRG.4300.4	gene_id	MSTRG.4300;
1	StringTie	exon	261664146	261664181	.	+	.	transcript_	MSTRG.4300.4	gene_id	MSTRG.4300;
1	StringTie	transcrip	261968018	262007870	.	+	.	transcript_	MSTRG.4302.1	gene_id	MSTRG.4302;
1	StringTie	exon	261968018	261968126	.	+	.	transcript_	MSTRG.4302.1	gene_id	MSTRG.4302;
1	StringTie	exon	262007715	262007870	.	+	.	transcript_	MSTRG.4302.1	gene_id	MSTRG.4302;
1	StringTie	transcrip	262047548	262078520	.	+	.	transcript_	MSTRG.4303.1	gene_id	MSTRG.4303;
1	StringTie	exon	262047548	262047567	.	+	.	transcript_	MSTRG.4303.1	gene_id	MSTRG.4303;
1	StringTie	exon	262077897	262078520	.	+	.	transcript_	MSTRG.4303.1	gene_id	MSTRG.4303;
1	StringTie	transcrip	263759621	263767178	.	+	.	transcript_	MSTRG.4324.1	gene_id	MSTRG.4324;
1	StringTie	exon	263759621	263760281	.	+	.	transcript_	MSTRG.4324.1	gene_id	MSTRG.4324;
1	StringTie	exon	263766681	263767178	.	+	.	transcript_	MSTRG.4324.1	gene_id	MSTRG.4324;

1	StringTie	transcrip	263759625	263767096	.	+	.	transcript_	MSTRG.4324.2	gene_id	MSTRG.4324;
1	StringTie	exon	263759625	263759997	.	+	.	transcript_	MSTRG.4324.2	gene_id	MSTRG.4324;
1	StringTie	exon	263766681	263767096	.	+	.	transcript_	MSTRG.4324.2	gene_id	MSTRG.4324;
1	StringTie	transcrip	264223414	264237738	.	+	.	transcript_	MSTRG.4329.1	gene_id	MSTRG.4329;
1	StringTie	exon	264223414	264223643	.	+	.	transcript_	MSTRG.4329.1	gene_id	MSTRG.4329;
1	StringTie	exon	264228418	264228767	.	+	.	transcript_	MSTRG.4329.1	gene_id	MSTRG.4329;
1	StringTie	exon	264230952	264231139	.	+	.	transcript_	MSTRG.4329.1	gene_id	MSTRG.4329;
1	StringTie	exon	264237266	264237738	.	+	.	transcript_	MSTRG.4329.1	gene_id	MSTRG.4329;
1	StringTie	transcrip	265461746	265493963	.	+	.	transcript_	MSTRG.4347.1	gene_id	MSTRG.4347;
1	StringTie	exon	265461746	265461958	.	+	.	transcript_	MSTRG.4347.1	gene_id	MSTRG.4347;
1	StringTie	exon	265493859	265493963	.	+	.	transcript_	MSTRG.4347.1	gene_id	MSTRG.4347;
1	StringTie	transcrip	269122224	269123090	.	+	.	transcript_	MSTRG.4476.1	gene_id	MSTRG.4476;
1	StringTie	exon	269122224	269122289	.	+	.	transcript_	MSTRG.4476.1	gene_id	MSTRG.4476;
1	StringTie	exon	269122574	269122639	.	+	.	transcript_	MSTRG.4476.1	gene_id	MSTRG.4476;
1	StringTie	exon	269122766	269123090	.	+	.	transcript_	MSTRG.4476.1	gene_id	MSTRG.4476;
1	StringTie	transcrip	269575544	269586378	.	+	.	transcript_	MSTRG.4485.1	gene_id	MSTRG.4485;
1	StringTie	exon	269575544	269575624	.	+	.	transcript_	MSTRG.4485.1	gene_id	MSTRG.4485;
1	StringTie	exon	269583521	269583665	.	+	.	transcript_	MSTRG.4485.1	gene_id	MSTRG.4485;
1	StringTie	exon	269586036	269586378	.	+	.	transcript_	MSTRG.4485.1	gene_id	MSTRG.4485;
1	StringTie	transcrip	269582356	269586366	.	+	.	transcript_	MSTRG.4485.2	gene_id	MSTRG.4485;
1	StringTie	exon	269582356	269582676	.	+	.	transcript_	MSTRG.4485.2	gene_id	MSTRG.4485;
1	StringTie	exon	269583521	269583665	.	+	.	transcript_	MSTRG.4485.2	gene_id	MSTRG.4485;
1	StringTie	exon	269586036	269586366	.	+	.	transcript_	MSTRG.4485.2	gene_id	MSTRG.4485;
1	StringTie	transcrip	270180487	270182683	.	+	.	transcript_	MSTRG.4510.1	gene_id	MSTRG.4510;
1	StringTie	exon	270180487	270181074	.	+	.	transcript_	MSTRG.4510.1	gene_id	MSTRG.4510;
1	StringTie	exon	270182034	270182683	.	+	.	transcript_	MSTRG.4510.1	gene_id	MSTRG.4510;
1	StringTie	transcrip	271275822	271277382	.	+	.	transcript_	MSTRG.4536.1	gene_id	MSTRG.4536;
1	StringTie	exon	271275822	271277091	.	+	.	transcript_	MSTRG.4536.1	gene_id	MSTRG.4536;
1	StringTie	exon	271277112	271277382	.	+	.	transcript_	MSTRG.4536.1	gene_id	MSTRG.4536;
1	StringTie	transcrip	273945827	273947258	.	+	.	transcript_	MSTRG.4619.1	gene_id	MSTRG.4619;
1	StringTie	exon	273945827	273946064	.	+	.	transcript_	MSTRG.4619.1	gene_id	MSTRG.4619;
1	StringTie	exon	273947072	273947258	.	+	.	transcript_	MSTRG.4619.1	gene_id	MSTRG.4619;
1	StringTie	transcrip	2524141	2533607	.	-	.	transcript_	MSTRG.70.1;	gene_id	MSTRG.70;
1	StringTie	exon	2524141	2526864	.	-	.	transcript_	MSTRG.70.1;	gene_id	MSTRG.70;
1	StringTie	exon	2529712	2529809	.	-	.	transcript_	MSTRG.70.1;	gene_id	MSTRG.70;

1	StringTie	exon	2533117	2533607	.	-	.	transcript_	MSTRG.70.1;	gene_id	MSTRG.70;
1	StringTie	transcrip	2536267	2537315	.	-	.	transcript_	MSTRG.69.1;	gene_id	MSTRG.69;
1	StringTie	exon	2536267	2536434	.	-	.	transcript_	MSTRG.69.1;	gene_id	MSTRG.69;
1	StringTie	exon	2537130	2537315	.	-	.	transcript_	MSTRG.69.1;	gene_id	MSTRG.69;
1	StringTie	transcrip	8033665	8036831	.	-	.	transcript_	MSTRG.151.1;	gene_id	MSTRG.151;
1	StringTie	exon	8033665	8034235	.	-	.	transcript_	MSTRG.151.1;	gene_id	MSTRG.151;
1	StringTie	exon	8036528	8036831	.	-	.	transcript_	MSTRG.151.1;	gene_id	MSTRG.151;
1	StringTie	transcrip	11888123	11892164	.	-	.	transcript_	MSTRG.222.2;	gene_id	MSTRG.222;
1	StringTie	exon	11888123	11888352	.	-	.	transcript_	MSTRG.222.2;	gene_id	MSTRG.222;
1	StringTie	exon	11892124	11892164	.	-	.	transcript_	MSTRG.222.2;	gene_id	MSTRG.222;
1	StringTie	transcrip	12005086	12006916	.	-	.	transcript_	MSTRG.223.1;	gene_id	MSTRG.223;
1	StringTie	exon	12005086	12005684	.	-	.	transcript_	MSTRG.223.1;	gene_id	MSTRG.223;
1	StringTie	exon	12005963	12006916	.	-	.	transcript_	MSTRG.223.1;	gene_id	MSTRG.223;
1	StringTie	transcrip	14147059	14151522	.	-	.	transcript_	MSTRG.272.1;	gene_id	MSTRG.272;
1	StringTie	exon	14147059	14149215	.	-	.	transcript_	MSTRG.272.1;	gene_id	MSTRG.272;
1	StringTie	exon	14149708	14149767	.	-	.	transcript_	MSTRG.272.1;	gene_id	MSTRG.272;
1	StringTie	exon	14151033	14151522	.	-	.	transcript_	MSTRG.272.1;	gene_id	MSTRG.272;
1	StringTie	transcrip	14147221	14151413	.	-	.	transcript_	MSTRG.272.3;	gene_id	MSTRG.272;
1	StringTie	exon	14147221	14147918	.	-	.	transcript_	MSTRG.272.3;	gene_id	MSTRG.272;
1	StringTie	exon	14149708	14149767	.	-	.	transcript_	MSTRG.272.3;	gene_id	MSTRG.272;
1	StringTie	exon	14151033	14151413	.	-	.	transcript_	MSTRG.272.3;	gene_id	MSTRG.272;
1	StringTie	transcrip	14147221	14151522	.	-	.	transcript_	MSTRG.272.2;	gene_id	MSTRG.272;
1	StringTie	exon	14147221	14149215	.	-	.	transcript_	MSTRG.272.2;	gene_id	MSTRG.272;
1	StringTie	exon	14149708	14149767	.	-	.	transcript_	MSTRG.272.2;	gene_id	MSTRG.272;
1	StringTie	exon	14151033	14151174	.	-	.	transcript_	MSTRG.272.2;	gene_id	MSTRG.272;
1	StringTie	exon	14151435	14151522	.	-	.	transcript_	MSTRG.272.2;	gene_id	MSTRG.272;
1	StringTie	transcrip	14148317	14151262	.	-	.	transcript_	MSTRG.272.4;	gene_id	MSTRG.272;
1	StringTie	exon	14148317	14149215	.	-	.	transcript_	MSTRG.272.4;	gene_id	MSTRG.272;
1	StringTie	exon	14149551	14149767	.	-	.	transcript_	MSTRG.272.4;	gene_id	MSTRG.272;
1	StringTie	exon	14151033	14151262	.	-	.	transcript_	MSTRG.272.4;	gene_id	MSTRG.272;
1	StringTie	transcrip	16672136	16673285	.	-	.	transcript_	MSTRG.300.1;	gene_id	MSTRG.300;
1	StringTie	exon	16672136	16672449	.	-	.	transcript_	MSTRG.300.1;	gene_id	MSTRG.300;
1	StringTie	exon	16672783	16673285	.	-	.	transcript_	MSTRG.300.1;	gene_id	MSTRG.300;
1	StringTie	transcrip	16673789	16681500	.	-	.	transcript_	MSTRG.301.1;	gene_id	MSTRG.301;
1	StringTie	exon	16673789	16674208	.	-	.	transcript_	MSTRG.301.1;	gene_id	MSTRG.301;

1	StringTie	exon	16681212	16681500	.	-	.	transcript_	MSTRG.301.1;	gene_id	MSTRG.301;
1	StringTie	transcrip	19604497	19609799	.	-	.	transcript_	MSTRG.365.1;	gene_id	MSTRG.365;
1	StringTie	exon	19604497	19604797	.	-	.	transcript_	MSTRG.365.1;	gene_id	MSTRG.365;
1	StringTie	exon	19609752	19609799	.	-	.	transcript_	MSTRG.365.1;	gene_id	MSTRG.365;
1	StringTie	transcrip	19715330	19755683	.	-	.	transcript_	MSTRG.409.1;	gene_id	MSTRG.409;
1	StringTie	exon	19715330	19716480	.	-	.	transcript_	MSTRG.409.1;	gene_id	MSTRG.409;
1	StringTie	exon	19749271	19749343	.	-	.	transcript_	MSTRG.409.1;	gene_id	MSTRG.409;
1	StringTie	exon	19755449	19755683	.	-	.	transcript_	MSTRG.409.1;	gene_id	MSTRG.409;
1	StringTie	transcrip	19730146	19755694	.	-	.	transcript_	MSTRG.409.2;	gene_id	MSTRG.409;
1	StringTie	exon	19730146	19732605	.	-	.	transcript_	MSTRG.409.2;	gene_id	MSTRG.409;
1	StringTie	exon	19749271	19749343	.	-	.	transcript_	MSTRG.409.2;	gene_id	MSTRG.409;
1	StringTie	exon	19755449	19755694	.	-	.	transcript_	MSTRG.409.2;	gene_id	MSTRG.409;
1	StringTie	transcrip	21074052	21096341	.	-	.	transcript_	MSTRG.431.1;	gene_id	MSTRG.431;
1	StringTie	exon	21074052	21074094	.	-	.	transcript_	MSTRG.431.1;	gene_id	MSTRG.431;
1	StringTie	exon	21096098	21096341	.	-	.	transcript_	MSTRG.431.1;	gene_id	MSTRG.431;
1	StringTie	transcrip	27038091	27038888	.	-	.	transcript_	MSTRG.550.1;	gene_id	MSTRG.550;
1	StringTie	exon	27038091	27038543	.	-	.	transcript_	MSTRG.550.1;	gene_id	MSTRG.550;
1	StringTie	exon	27038842	27038888	.	-	.	transcript_	MSTRG.550.1;	gene_id	MSTRG.550;
1	StringTie	transcrip	27869779	27871967	.	-	.	transcript_	MSTRG.562.1;	gene_id	MSTRG.562;
1	StringTie	exon	27869779	27871967	.	-	.	transcript_	MSTRG.562.1;	gene_id	MSTRG.562;
1	StringTie	transcrip	27869873	27871954	.	-	.	transcript_	MSTRG.562.2;	gene_id	MSTRG.562;
1	StringTie	exon	27869873	27871143	.	-	.	transcript_	MSTRG.562.2;	gene_id	MSTRG.562;
1	StringTie	exon	27871868	27871954	.	-	.	transcript_	MSTRG.562.2;	gene_id	MSTRG.562;
1	StringTie	transcrip	27934552	27941372	.	-	.	transcript_	MSTRG.561.1;	gene_id	MSTRG.561;
1	StringTie	exon	27934552	27935309	.	-	.	transcript_	MSTRG.561.1;	gene_id	MSTRG.561;
1	StringTie	exon	27940830	27941372	.	-	.	transcript_	MSTRG.561.1;	gene_id	MSTRG.561;
1	StringTie	transcrip	32586203	32590452	.	-	.	transcript_	MSTRG.680.1;	gene_id	MSTRG.680;
1	StringTie	exon	32586203	32586979	.	-	.	transcript_	MSTRG.680.1;	gene_id	MSTRG.680;
1	StringTie	exon	32587036	32590452	.	-	.	transcript_	MSTRG.680.1;	gene_id	MSTRG.680;
1	StringTie	transcrip	37199519	37203528	.	-	.	transcript_	MSTRG.738.1;	gene_id	MSTRG.738;
1	StringTie	exon	37199519	37201456	.	-	.	transcript_	MSTRG.738.1;	gene_id	MSTRG.738;
1	StringTie	exon	37201932	37203528	.	-	.	transcript_	MSTRG.738.1;	gene_id	MSTRG.738;
1	StringTie	transcrip	37201244	37203502	.	-	.	transcript_	MSTRG.738.2;	gene_id	MSTRG.738;
1	StringTie	exon	37201244	37203502	.	-	.	transcript_	MSTRG.738.2;	gene_id	MSTRG.738;
1	StringTie	transcrip	37201963	37203078	.	-	.	transcript_	MSTRG.738.3;	gene_id	MSTRG.738;

1	StringTie	exon	37201963	37202039	.	-	.	transcript_	MSTRG.738.3;	gene_id	MSTRG.738;
1	StringTie	exon	37202629	37203078	.	-	.	transcript_	MSTRG.738.3;	gene_id	MSTRG.738;
1	StringTie	transcrip	66413113	66467841	.	-	.	transcript_	MSTRG.1179.1	gene_id	MSTRG.1179;
1	StringTie	exon	66413113	66413253	.	-	.	transcript_	MSTRG.1179.1	gene_id	MSTRG.1179;
1	StringTie	exon	66467167	66467841	.	-	.	transcript_	MSTRG.1179.1	gene_id	MSTRG.1179;
1	StringTie	transcrip	107150274	107154150	.	-	.	transcript_	MSTRG.1652.4	gene_id	MSTRG.1652;
1	StringTie	exon	107150274	107150369	.	-	.	transcript_	MSTRG.1652.4	gene_id	MSTRG.1652;
1	StringTie	exon	107153928	107154150	.	-	.	transcript_	MSTRG.1652.4	gene_id	MSTRG.1652;
1	StringTie	transcrip	107329727	107413900	.	-	.	transcript_	MSTRG.1664.1	gene_id	MSTRG.1664;
1	StringTie	exon	107329727	107331699	.	-	.	transcript_	MSTRG.1664.1	gene_id	MSTRG.1664;
1	StringTie	exon	107380468	107380500	.	-	.	transcript_	MSTRG.1664.1	gene_id	MSTRG.1664;
1	StringTie	exon	107413441	107413900	.	-	.	transcript_	MSTRG.1664.1	gene_id	MSTRG.1664;
1	StringTie	transcrip	111536129	111536916	.	-	.	transcript_	MSTRG.1839.1	gene_id	MSTRG.1839;
1	StringTie	exon	111536129	111536341	.	-	.	transcript_	MSTRG.1839.1	gene_id	MSTRG.1839;
1	StringTie	exon	111536645	111536916	.	-	.	transcript_	MSTRG.1839.1	gene_id	MSTRG.1839;
1	StringTie	transcrip	115696817	115734764	.	-	.	transcript_	MSTRG.1935.1	gene_id	MSTRG.1935;
1	StringTie	exon	115696817	115702479	.	-	.	transcript_	MSTRG.1935.1	gene_id	MSTRG.1935;
1	StringTie	exon	115732322	115732439	.	-	.	transcript_	MSTRG.1935.1	gene_id	MSTRG.1935;
1	StringTie	exon	115733876	115733962	.	-	.	transcript_	MSTRG.1935.1	gene_id	MSTRG.1935;
1	StringTie	exon	115734744	115734764	.	-	.	transcript_	MSTRG.1935.1	gene_id	MSTRG.1935;
1	StringTie	transcrip	115699004	115734635	.	-	.	transcript_	MSTRG.1935.2	gene_id	MSTRG.1935;
1	StringTie	exon	115699004	115702479	.	-	.	transcript_	MSTRG.1935.2	gene_id	MSTRG.1935;
1	StringTie	exon	115732322	115732439	.	-	.	transcript_	MSTRG.1935.2	gene_id	MSTRG.1935;
1	StringTie	exon	115733876	115733962	.	-	.	transcript_	MSTRG.1935.2	gene_id	MSTRG.1935;
1	StringTie	exon	115734614	115734635	.	-	.	transcript_	MSTRG.1935.2	gene_id	MSTRG.1935;
1	StringTie	transcrip	115724386	115726541	.	-	.	transcript_	MSTRG.1936.1	gene_id	MSTRG.1936;
1	StringTie	exon	115724386	115725368	.	-	.	transcript_	MSTRG.1936.1	gene_id	MSTRG.1936;
1	StringTie	exon	115726360	115726541	.	-	.	transcript_	MSTRG.1936.1	gene_id	MSTRG.1936;
1	StringTie	transcrip	116253878	116258079	.	-	.	transcript_	MSTRG.1942.1	gene_id	MSTRG.1942;
1	StringTie	exon	116253878	116254269	.	-	.	transcript_	MSTRG.1942.1	gene_id	MSTRG.1942;
1	StringTie	exon	116257961	116258079	.	-	.	transcript_	MSTRG.1942.1	gene_id	MSTRG.1942;
1	StringTie	transcrip	129179532	129197491	.	-	.	transcript_	MSTRG.2164.1	gene_id	MSTRG.2164;
1	StringTie	exon	129179532	129179902	.	-	.	transcript_	MSTRG.2164.1	gene_id	MSTRG.2164;
1	StringTie	exon	129197383	129197491	.	-	.	transcript_	MSTRG.2164.1	gene_id	MSTRG.2164;
1	StringTie	transcrip	138326669	138419657	.	-	.	transcript_	MSTRG.2476.1	gene_id	MSTRG.2476;

1	StringTie	exon	138326669	138326686	.	-	.	transcript_	MSTRG.2476.1	gene_id	MSTRG.2476;
1	StringTie	exon	138419316	138419657	.	-	.	transcript_	MSTRG.2476.1	gene_id	MSTRG.2476;
1	StringTie	transcrip	142080325	142088686	.	-	.	transcript_	MSTRG.2517.1	gene_id	MSTRG.2517;
1	StringTie	exon	142080325	142081829	.	-	.	transcript_	MSTRG.2517.1	gene_id	MSTRG.2517;
1	StringTie	exon	142082543	142082655	.	-	.	transcript_	MSTRG.2517.1	gene_id	MSTRG.2517;
1	StringTie	exon	142083412	142083540	.	-	.	transcript_	MSTRG.2517.1	gene_id	MSTRG.2517;
1	StringTie	exon	142088477	142088686	.	-	.	transcript_	MSTRG.2517.1	gene_id	MSTRG.2517;
1	StringTie	transcrip	148795900	148799867	.	-	.	transcript_	MSTRG.2582.1	gene_id	MSTRG.2582;
1	StringTie	exon	148795900	148798629	.	-	.	transcript_	MSTRG.2582.1	gene_id	MSTRG.2582;
1	StringTie	exon	148799843	148799867	.	-	.	transcript_	MSTRG.2582.1	gene_id	MSTRG.2582;
1	StringTie	transcrip	148799901	148801066	.	-	.	transcript_	MSTRG.2583.1	gene_id	MSTRG.2583;
1	StringTie	exon	148799901	148799984	.	-	.	transcript_	MSTRG.2583.1	gene_id	MSTRG.2583;
1	StringTie	exon	148800823	148801066	.	-	.	transcript_	MSTRG.2583.1	gene_id	MSTRG.2583;
1	StringTie	transcrip	161817333	161818803	.	-	.	transcript_	MSTRG.2724.6	gene_id	MSTRG.2724;
1	StringTie	exon	161817333	161817804	.	-	.	transcript_	MSTRG.2724.6	gene_id	MSTRG.2724;
1	StringTie	exon	161818736	161818803	.	-	.	transcript_	MSTRG.2724.6	gene_id	MSTRG.2724;
1	StringTie	transcrip	162187306	162227446	.	-	.	transcript_	MSTRG.2734.1	gene_id	MSTRG.2734;
1	StringTie	exon	162187306	162187493	.	-	.	transcript_	MSTRG.2734.1	gene_id	MSTRG.2734;
1	StringTie	exon	162192951	162193054	.	-	.	transcript_	MSTRG.2734.1	gene_id	MSTRG.2734;
1	StringTie	exon	162220720	162220828	.	-	.	transcript_	MSTRG.2734.1	gene_id	MSTRG.2734;
1	StringTie	exon	162227250	162227446	.	-	.	transcript_	MSTRG.2734.1	gene_id	MSTRG.2734;
1	StringTie	transcrip	162216337	162223765	.	-	.	transcript_	MSTRG.2734.3	gene_id	MSTRG.2734;
1	StringTie	exon	162216337	162220828	.	-	.	transcript_	MSTRG.2734.3	gene_id	MSTRG.2734;
1	StringTie	exon	162223101	162223238	.	-	.	transcript_	MSTRG.2734.3	gene_id	MSTRG.2734;
1	StringTie	exon	162223719	162223765	.	-	.	transcript_	MSTRG.2734.3	gene_id	MSTRG.2734;
1	StringTie	transcrip	164282600	164285255	.	-	.	transcript_	MSTRG.2763.1	gene_id	MSTRG.2763;
1	StringTie	exon	164282600	164283843	.	-	.	transcript_	MSTRG.2763.1	gene_id	MSTRG.2763;
1	StringTie	exon	164285042	164285255	.	-	.	transcript_	MSTRG.2763.1	gene_id	MSTRG.2763;
1	StringTie	transcrip	167277992	167295948	.	-	.	transcript_	MSTRG.2852.1	gene_id	MSTRG.2852;
1	StringTie	exon	167277992	167279174	.	-	.	transcript_	MSTRG.2852.1	gene_id	MSTRG.2852;
1	StringTie	exon	167279775	167279868	.	-	.	transcript_	MSTRG.2852.1	gene_id	MSTRG.2852;
1	StringTie	exon	167295586	167295948	.	-	.	transcript_	MSTRG.2852.1	gene_id	MSTRG.2852;
1	StringTie	transcrip	184369412	184383161	.	-	.	transcript_	MSTRG.3097.1	gene_id	MSTRG.3097;
1	StringTie	exon	184369412	184369429	.	-	.	transcript_	MSTRG.3097.1	gene_id	MSTRG.3097;
1	StringTie	exon	184382710	184383161	.	-	.	transcript_	MSTRG.3097.1	gene_id	MSTRG.3097;

1	StringTie	transcrip	186524327	186529664	.	-	.	transcript_	MSTRG.3130.1	gene_id	MSTRG.3130;
1	StringTie	exon	186524327	186524372	.	-	.	transcript_	MSTRG.3130.1	gene_id	MSTRG.3130;
1	StringTie	exon	186529198	186529664	.	-	.	transcript_	MSTRG.3130.1	gene_id	MSTRG.3130;
1	StringTie	transcrip	189283455	189394043	.	-	.	transcript_	MSTRG.3175.1	gene_id	MSTRG.3175;
1	StringTie	exon	189283455	189283515	.	-	.	transcript_	MSTRG.3175.1	gene_id	MSTRG.3175;
1	StringTie	exon	189331487	189331568	.	-	.	transcript_	MSTRG.3175.1	gene_id	MSTRG.3175;
1	StringTie	exon	189356804	189356842	.	-	.	transcript_	MSTRG.3175.1	gene_id	MSTRG.3175;
1	StringTie	exon	189357898	189358058	.	-	.	transcript_	MSTRG.3175.1	gene_id	MSTRG.3175;
1	StringTie	exon	189390621	189390679	.	-	.	transcript_	MSTRG.3175.1	gene_id	MSTRG.3175;
1	StringTie	exon	189391878	189391967	.	-	.	transcript_	MSTRG.3175.1	gene_id	MSTRG.3175;
1	StringTie	exon	189394013	189394043	.	-	.	transcript_	MSTRG.3175.1	gene_id	MSTRG.3175;
1	StringTie	transcrip	194324169	194352219	.	-	.	transcript_	MSTRG.3290.1	gene_id	MSTRG.3290;
1	StringTie	exon	194324169	194325525	.	-	.	transcript_	MSTRG.3290.1	gene_id	MSTRG.3290;
1	StringTie	exon	194334064	194334208	.	-	.	transcript_	MSTRG.3290.1	gene_id	MSTRG.3290;
1	StringTie	exon	194350597	194350729	.	-	.	transcript_	MSTRG.3290.1	gene_id	MSTRG.3290;
1	StringTie	exon	194352189	194352219	.	-	.	transcript_	MSTRG.3290.1	gene_id	MSTRG.3290;
1	StringTie	transcrip	194324824	194352294	.	-	.	transcript_	MSTRG.3290.2	gene_id	MSTRG.3290;
1	StringTie	exon	194324824	194325525	.	-	.	transcript_	MSTRG.3290.2	gene_id	MSTRG.3290;
1	StringTie	exon	194350597	194350729	.	-	.	transcript_	MSTRG.3290.2	gene_id	MSTRG.3290;
1	StringTie	exon	194352189	194352294	.	-	.	transcript_	MSTRG.3290.2	gene_id	MSTRG.3290;
1	StringTie	transcrip	201568442	201585218	.	-	.	transcript_	MSTRG.3413.1	gene_id	MSTRG.3413;
1	StringTie	exon	201568442	201568616	.	-	.	transcript_	MSTRG.3413.1	gene_id	MSTRG.3413;
1	StringTie	exon	201585127	201585218	.	-	.	transcript_	MSTRG.3413.1	gene_id	MSTRG.3413;
1	StringTie	transcrip	201655009	201656982	.	-	.	transcript_	MSTRG.3429.1	gene_id	MSTRG.3429;
1	StringTie	exon	201655009	201655057	.	-	.	transcript_	MSTRG.3429.1	gene_id	MSTRG.3429;
1	StringTie	exon	201655329	201655462	.	-	.	transcript_	MSTRG.3429.1	gene_id	MSTRG.3429;
1	StringTie	exon	201656907	201656982	.	-	.	transcript_	MSTRG.3429.1	gene_id	MSTRG.3429;
1	StringTie	transcrip	201668404	201771548	.	-	.	transcript_	MSTRG.3433.3	gene_id	MSTRG.3433;
1	StringTie	exon	201668404	201668807	.	-	.	transcript_	MSTRG.3433.3	gene_id	MSTRG.3433;
1	StringTie	exon	201675162	201675292	.	-	.	transcript_	MSTRG.3433.3	gene_id	MSTRG.3433;
1	StringTie	exon	201675661	201675919	.	-	.	transcript_	MSTRG.3433.3	gene_id	MSTRG.3433;
1	StringTie	exon	201735794	201735856	.	-	.	transcript_	MSTRG.3433.3	gene_id	MSTRG.3433;
1	StringTie	exon	201771448	201771548	.	-	.	transcript_	MSTRG.3433.3	gene_id	MSTRG.3433;
1	StringTie	transcrip	201675555	201678873	.	-	.	transcript_	MSTRG.3433.4	gene_id	MSTRG.3433;
1	StringTie	exon	201675555	201675919	.	-	.	transcript_	MSTRG.3433.4	gene_id	MSTRG.3433;

1	StringTie	exon	201677438	201677500	.	-	.	transcript_	MSTRG.3433.4	gene_id MSTRG.3433;
1	StringTie	exon	201678828	201678873	.	-	.	transcript_	MSTRG.3433.4	gene_id MSTRG.3433;
1	StringTie	transcrip	201675689	201693386	.	-	.	transcript_	MSTRG.3433.5	gene_id MSTRG.3433;
1	StringTie	exon	201675689	201675919	.	-	.	transcript_	MSTRG.3433.5	gene_id MSTRG.3433;
1	StringTie	exon	201677438	201677500	.	-	.	transcript_	MSTRG.3433.5	gene_id MSTRG.3433;
1	StringTie	exon	201693337	201693386	.	-	.	transcript_	MSTRG.3433.5	gene_id MSTRG.3433;
1	StringTie	transcrip	203348141	203354890	.	-	.	transcript_	MSTRG.3339.1	gene_id MSTRG.3339;
1	StringTie	exon	203348141	203348384	.	-	.	transcript_	MSTRG.3339.1	gene_id MSTRG.3339;
1	StringTie	exon	203354747	203354890	.	-	.	transcript_	MSTRG.3339.1	gene_id MSTRG.3339;
1	StringTie	transcrip	206362461	206381007	.	-	.	transcript_	MSTRG.3451.1	gene_id MSTRG.3451;
1	StringTie	exon	206362461	206362477	.	-	.	transcript_	MSTRG.3451.1	gene_id MSTRG.3451;
1	StringTie	exon	206377273	206381007	.	-	.	transcript_	MSTRG.3451.1	gene_id MSTRG.3451;
1	StringTie	transcrip	216709163	216709966	.	-	.	transcript_	MSTRG.3569.1	gene_id MSTRG.3569;
1	StringTie	exon	216709163	216709221	.	-	.	transcript_	MSTRG.3569.1	gene_id MSTRG.3569;
1	StringTie	exon	216709774	216709966	.	-	.	transcript_	MSTRG.3569.1	gene_id MSTRG.3569;
1	StringTie	transcrip	216729402	216736972	.	-	.	transcript_	MSTRG.3570.1	gene_id MSTRG.3570;
1	StringTie	exon	216729402	216729758	.	-	.	transcript_	MSTRG.3570.1	gene_id MSTRG.3570;
1	StringTie	exon	216736798	216736972	.	-	.	transcript_	MSTRG.3570.1	gene_id MSTRG.3570;
1	StringTie	transcrip	229872022	229955692	.	-	.	transcript_	MSTRG.3832.1	gene_id MSTRG.3832;
1	StringTie	exon	229872022	229873193	.	-	.	transcript_	MSTRG.3832.1	gene_id MSTRG.3832;
1	StringTie	exon	229928721	229928855	.	-	.	transcript_	MSTRG.3832.1	gene_id MSTRG.3832;
1	StringTie	exon	229952108	229952245	.	-	.	transcript_	MSTRG.3832.1	gene_id MSTRG.3832;
1	StringTie	exon	229955621	229955692	.	-	.	transcript_	MSTRG.3832.1	gene_id MSTRG.3832;
1	StringTie	transcrip	229872046	229952745	.	-	.	transcript_	MSTRG.3832.1	gene_id MSTRG.3832;
1	StringTie	exon	229872046	229873193	.	-	.	transcript_	MSTRG.3832.1	gene_id MSTRG.3832;
1	StringTie	exon	229928721	229928855	.	-	.	transcript_	MSTRG.3832.1	gene_id MSTRG.3832;
1	StringTie	exon	229952108	229952245	.	-	.	transcript_	MSTRG.3832.1	gene_id MSTRG.3832;
1	StringTie	exon	229952570	229952745	.	-	.	transcript_	MSTRG.3832.1	gene_id MSTRG.3832;
1	StringTie	transcrip	241544999	241606079	.	-	.	transcript_	MSTRG.4017.1	gene_id MSTRG.4017;
1	StringTie	exon	241544999	241545027	.	-	.	transcript_	MSTRG.4017.1	gene_id MSTRG.4017;
1	StringTie	exon	241602720	241606079	.	-	.	transcript_	MSTRG.4017.1	gene_id MSTRG.4017;
1	StringTie	transcrip	250001985	250003402	.	-	.	transcript_	MSTRG.4124.1	gene_id MSTRG.4124;
1	StringTie	exon	250001985	250002507	.	-	.	transcript_	MSTRG.4124.1	gene_id MSTRG.4124;
1	StringTie	exon	250002786	250003402	.	-	.	transcript_	MSTRG.4124.1	gene_id MSTRG.4124;
1	StringTie	transcrip	252423032	252426375	.	-	.	transcript_	MSTRG.4188.1	gene_id MSTRG.4188;

1	StringTie	exon	252423032	252424600	.	-	.	transcript_	MSTRG.4188.1	gene_id	MSTRG.4188;
1	StringTie	exon	252425778	252426375	.	-	.	transcript_	MSTRG.4188.1	gene_id	MSTRG.4188;
1	StringTie	transcrip	267733051	267735085	.	-	.	transcript_	MSTRG.4418.1	gene_id	MSTRG.4418;
1	StringTie	exon	267733051	267733535	.	-	.	transcript_	MSTRG.4418.1	gene_id	MSTRG.4418;
1	StringTie	exon	267734864	267735085	.	-	.	transcript_	MSTRG.4418.1	gene_id	MSTRG.4418;
1	StringTie	transcrip	270038605	270048457	.	-	.	transcript_	MSTRG.4508.1	gene_id	MSTRG.4508;
1	StringTie	exon	270038605	270038723	.	-	.	transcript_	MSTRG.4508.1	gene_id	MSTRG.4508;
1	StringTie	exon	270047676	270048457	.	-	.	transcript_	MSTRG.4508.1	gene_id	MSTRG.4508;
1	StringTie	transcrip	270641281	270652483	.	-	.	transcript_	MSTRG.4520.1	gene_id	MSTRG.4520;
1	StringTie	exon	270641281	270641760	.	-	.	transcript_	MSTRG.4520.1	gene_id	MSTRG.4520;
1	StringTie	exon	270643484	270643594	.	-	.	transcript_	MSTRG.4520.1	gene_id	MSTRG.4520;
1	StringTie	exon	270652396	270652483	.	-	.	transcript_	MSTRG.4520.1	gene_id	MSTRG.4520;
1	StringTie	transcrip	271260011	271270483	.	-	.	transcript_	MSTRG.4533.3	gene_id	MSTRG.4533;
1	StringTie	exon	271260011	271261787	.	-	.	transcript_	MSTRG.4533.3	gene_id	MSTRG.4533;
1	StringTie	exon	271270408	271270483	.	-	.	transcript_	MSTRG.4533.3	gene_id	MSTRG.4533;
1	StringTie	transcrip	271260162	271270481	.	-	.	transcript_	MSTRG.4533.4	gene_id	MSTRG.4533;
1	StringTie	exon	271260162	271261787	.	-	.	transcript_	MSTRG.4533.4	gene_id	MSTRG.4533;
1	StringTie	exon	271261875	271262047	.	-	.	transcript_	MSTRG.4533.4	gene_id	MSTRG.4533;
1	StringTie	exon	271270408	271270481	.	-	.	transcript_	MSTRG.4533.4	gene_id	MSTRG.4533;
1	StringTie	transcrip	272078166	272080416	.	-	.	transcript_	MSTRG.4558.1	gene_id	MSTRG.4558;
1	StringTie	exon	272078166	272078605	.	-	.	transcript_	MSTRG.4558.1	gene_id	MSTRG.4558;
1	StringTie	exon	272078809	272078926	.	-	.	transcript_	MSTRG.4558.1	gene_id	MSTRG.4558;
1	StringTie	exon	272079703	272079843	.	-	.	transcript_	MSTRG.4558.1	gene_id	MSTRG.4558;
1	StringTie	exon	272080298	272080416	.	-	.	transcript_	MSTRG.4558.1	gene_id	MSTRG.4558;
1	StringTie	transcrip	273557907	273559878	.	-	.	transcript_	MSTRG.4604.2	gene_id	MSTRG.4604;
1	StringTie	exon	273557907	273557963	.	-	.	transcript_	MSTRG.4604.2	gene_id	MSTRG.4604;
1	StringTie	exon	273558614	273559118	.	-	.	transcript_	MSTRG.4604.2	gene_id	MSTRG.4604;
1	StringTie	exon	273559661	273559878	.	-	.	transcript_	MSTRG.4604.2	gene_id	MSTRG.4604;
1	StringTie	transcrip	273557907	273559895	.	-	.	transcript_	MSTRG.4604.1	gene_id	MSTRG.4604;
1	StringTie	exon	273557907	273557963	.	-	.	transcript_	MSTRG.4604.1	gene_id	MSTRG.4604;
1	StringTie	exon	273558614	273559118	.	-	.	transcript_	MSTRG.4604.1	gene_id	MSTRG.4604;
1	StringTie	exon	273559720	273559895	.	-	.	transcript_	MSTRG.4604.1	gene_id	MSTRG.4604;
1	StringTie	transcrip	273587934	273590538	.	-	.	transcript_	MSTRG.4607.1	gene_id	MSTRG.4607;
1	StringTie	exon	273587934	273589046	.	-	.	transcript_	MSTRG.4607.1	gene_id	MSTRG.4607;
1	StringTie	exon	273589863	273590273	.	-	.	transcript_	MSTRG.4607.1	gene_id	MSTRG.4607;

1	StringTie	exon	273590456	273590538	.	-	.	transcript_	MSTRG.4607.1	gene_id	MSTRG.4607;
10	StringTie	transcrip	1675211	1681024	.	+	.	transcript_	MSTRG.4635.2	gene_id	MSTRG.4635;
10	StringTie	exon	1675211	1675268	.	+	.	transcript_	MSTRG.4635.2	gene_id	MSTRG.4635;
10	StringTie	exon	1679829	1679872	.	+	.	transcript_	MSTRG.4635.2	gene_id	MSTRG.4635;
10	StringTie	exon	1680752	1681024	.	+	.	transcript_	MSTRG.4635.2	gene_id	MSTRG.4635;
10	StringTie	transcrip	9219962	9221106	.	+	.	transcript_	MSTRG.4678.1	gene_id	MSTRG.4678;
10	StringTie	exon	9219962	9220235	.	+	.	transcript_	MSTRG.4678.1	gene_id	MSTRG.4678;
10	StringTie	exon	9220871	9221106	.	+	.	transcript_	MSTRG.4678.1	gene_id	MSTRG.4678;
10	StringTie	transcrip	10775953	10799381	.	+	.	transcript_	MSTRG.4727.1	gene_id	MSTRG.4727;
10	StringTie	exon	10775953	10776103	.	+	.	transcript_	MSTRG.4727.1	gene_id	MSTRG.4727;
10	StringTie	exon	10799234	10799381	.	+	.	transcript_	MSTRG.4727.1	gene_id	MSTRG.4727;
10	StringTie	transcrip	11301925	11302959	.	+	.	transcript_	MSTRG.4785.1	gene_id	MSTRG.4785;
10	StringTie	exon	11301925	11302047	.	+	.	transcript_	MSTRG.4785.1	gene_id	MSTRG.4785;
10	StringTie	exon	11302131	11302264	.	+	.	transcript_	MSTRG.4785.1	gene_id	MSTRG.4785;
10	StringTie	exon	11302882	11302959	.	+	.	transcript_	MSTRG.4785.1	gene_id	MSTRG.4785;
10	StringTie	transcrip	12432018	12435457	.	+	.	transcript_	MSTRG.4832.1	gene_id	MSTRG.4832;
10	StringTie	exon	12432018	12432086	.	+	.	transcript_	MSTRG.4832.1	gene_id	MSTRG.4832;
10	StringTie	exon	12433109	12433205	.	+	.	transcript_	MSTRG.4832.1	gene_id	MSTRG.4832;
10	StringTie	exon	12434769	12435457	.	+	.	transcript_	MSTRG.4832.1	gene_id	MSTRG.4832;
10	StringTie	transcrip	12537679	12538304	.	+	.	transcript_	MSTRG.4843.1	gene_id	MSTRG.4843;
10	StringTie	exon	12537679	12538304	.	+	.	transcript_	MSTRG.4843.1	gene_id	MSTRG.4843;
10	StringTie	transcrip	12537716	12538364	.	+	.	transcript_	MSTRG.4843.2	gene_id	MSTRG.4843;
10	StringTie	exon	12537716	12537993	.	+	.	transcript_	MSTRG.4843.2	gene_id	MSTRG.4843;
10	StringTie	exon	12538090	12538364	.	+	.	transcript_	MSTRG.4843.2	gene_id	MSTRG.4843;
10	StringTie	transcrip	12774756	12790834	.	+	.	transcript_	MSTRG.4858.1	gene_id	MSTRG.4858;
10	StringTie	exon	12774756	12774865	.	+	.	transcript_	MSTRG.4858.1	gene_id	MSTRG.4858;
10	StringTie	exon	12790201	12790834	.	+	.	transcript_	MSTRG.4858.1	gene_id	MSTRG.4858;
10	StringTie	transcrip	13965290	13970367	.	+	.	transcript_	MSTRG.4895.1	gene_id	MSTRG.4895;
10	StringTie	exon	13965290	13965366	.	+	.	transcript_	MSTRG.4895.1	gene_id	MSTRG.4895;
10	StringTie	exon	13968931	13969041	.	+	.	transcript_	MSTRG.4895.1	gene_id	MSTRG.4895;
10	StringTie	exon	13970101	13970367	.	+	.	transcript_	MSTRG.4895.1	gene_id	MSTRG.4895;
10	StringTie	transcrip	14654355	14656287	.	+	.	transcript_	MSTRG.4919.1	gene_id	MSTRG.4919;
10	StringTie	exon	14654355	14654902	.	+	.	transcript_	MSTRG.4919.1	gene_id	MSTRG.4919;
10	StringTie	exon	14655645	14656287	.	+	.	transcript_	MSTRG.4919.1	gene_id	MSTRG.4919;
10	StringTie	transcrip	17392908	17393473	.	+	.	transcript_	MSTRG.4952.1	gene_id	MSTRG.4952;

10	StringTie	exon	17392908	17392948	.	+	.	transcript_	MSTRG.4952.1	gene_id	MSTRG.4952;
10	StringTie	exon	17393062	17393473	.	+	.	transcript_	MSTRG.4952.1	gene_id	MSTRG.4952;
10	StringTie	transcrip	19657446	19692131	.	+	.	transcript_	MSTRG.4981.1	gene_id	MSTRG.4981;
10	StringTie	exon	19657446	19657469	.	+	.	transcript_	MSTRG.4981.1	gene_id	MSTRG.4981;
10	StringTie	exon	19691850	19692131	.	+	.	transcript_	MSTRG.4981.1	gene_id	MSTRG.4981;
10	StringTie	transcrip	24171972	24177201	.	+	.	transcript_	MSTRG.5058.1	gene_id	MSTRG.5058;
10	StringTie	exon	24171972	24172031	.	+	.	transcript_	MSTRG.5058.1	gene_id	MSTRG.5058;
10	StringTie	exon	24176995	24177201	.	+	.	transcript_	MSTRG.5058.1	gene_id	MSTRG.5058;
10	StringTie	transcrip	24962738	24969170	.	+	.	transcript_	MSTRG.5076.1	gene_id	MSTRG.5076;
10	StringTie	exon	24962738	24962846	.	+	.	transcript_	MSTRG.5076.1	gene_id	MSTRG.5076;
10	StringTie	exon	24968885	24969170	.	+	.	transcript_	MSTRG.5076.1	gene_id	MSTRG.5076;
10	StringTie	transcrip	24962740	24969170	.	+	.	transcript_	MSTRG.5076.2	gene_id	MSTRG.5076;
10	StringTie	exon	24962740	24962887	.	+	.	transcript_	MSTRG.5076.2	gene_id	MSTRG.5076;
10	StringTie	exon	24968926	24969170	.	+	.	transcript_	MSTRG.5076.2	gene_id	MSTRG.5076;
10	StringTie	transcrip	27078284	27078762	.	+	.	transcript_	MSTRG.5131.3	gene_id	MSTRG.5131;
10	StringTie	exon	27078284	27078539	.	+	.	transcript_	MSTRG.5131.3	gene_id	MSTRG.5131;
10	StringTie	exon	27078624	27078762	.	+	.	transcript_	MSTRG.5131.3	gene_id	MSTRG.5131;
10	StringTie	transcrip	27078284	27078762	.	+	.	transcript_	MSTRG.5131.4	gene_id	MSTRG.5131;
10	StringTie	exon	27078284	27078567	.	+	.	transcript_	MSTRG.5131.4	gene_id	MSTRG.5131;
10	StringTie	exon	27078624	27078762	.	+	.	transcript_	MSTRG.5131.4	gene_id	MSTRG.5131;
10	StringTie	transcrip	27078284	27078762	.	+	.	transcript_	MSTRG.5131.2	gene_id	MSTRG.5131;
10	StringTie	exon	27078284	27078553	.	+	.	transcript_	MSTRG.5131.2	gene_id	MSTRG.5131;
10	StringTie	exon	27078582	27078762	.	+	.	transcript_	MSTRG.5131.2	gene_id	MSTRG.5131;
10	StringTie	transcrip	27078284	27078762	.	+	.	transcript_	MSTRG.5131.1	gene_id	MSTRG.5131;
10	StringTie	exon	27078284	27078525	.	+	.	transcript_	MSTRG.5131.1	gene_id	MSTRG.5131;
10	StringTie	exon	27078582	27078762	.	+	.	transcript_	MSTRG.5131.1	gene_id	MSTRG.5131;
10	StringTie	transcrip	27078373	27078762	.	+	.	transcript_	MSTRG.5131.5	gene_id	MSTRG.5131;
10	StringTie	exon	27078373	27078567	.	+	.	transcript_	MSTRG.5131.5	gene_id	MSTRG.5131;
10	StringTie	exon	27078652	27078762	.	+	.	transcript_	MSTRG.5131.5	gene_id	MSTRG.5131;
10	StringTie	transcrip	27078387	27078734	.	+	.	transcript_	MSTRG.5131.8	gene_id	MSTRG.5131;
10	StringTie	exon	27078387	27078595	.	+	.	transcript_	MSTRG.5131.8	gene_id	MSTRG.5131;
10	StringTie	exon	27078624	27078734	.	+	.	transcript_	MSTRG.5131.8	gene_id	MSTRG.5131;
10	StringTie	transcrip	27078387	27078762	.	+	.	transcript_	MSTRG.5131.7	gene_id	MSTRG.5131;
10	StringTie	exon	27078387	27078497	.	+	.	transcript_	MSTRG.5131.7	gene_id	MSTRG.5131;
10	StringTie	exon	27078582	27078762	.	+	.	transcript_	MSTRG.5131.7	gene_id	MSTRG.5131;

10	StringTie	transcrip	27078387	27078762	.	+	.	transcript_	MSTRG.5131.6	gene_id	MSTRG.5131;
10	StringTie	exon	27078387	27078511	.	+	.	transcript_	MSTRG.5131.6	gene_id	MSTRG.5131;
10	StringTie	exon	27078596	27078762	.	+	.	transcript_	MSTRG.5131.6	gene_id	MSTRG.5131;
10	StringTie	transcrip	27078405	27078762	.	+	.	transcript_	MSTRG.5131.9	gene_id	MSTRG.5131;
10	StringTie	exon	27078405	27078539	.	+	.	transcript_	MSTRG.5131.9	gene_id	MSTRG.5131;
10	StringTie	exon	27078596	27078762	.	+	.	transcript_	MSTRG.5131.9	gene_id	MSTRG.5131;
10	StringTie	transcrip	27078405	27078762	.	+	.	transcript_	MSTRG.5131.1(	gene_id	MSTRG.5131;
10	StringTie	exon	27078405	27078511	.	+	.	transcript_	MSTRG.5131.1(	gene_id	MSTRG.5131;
10	StringTie	exon	27078596	27078623	.	+	.	transcript_	MSTRG.5131.1(	gene_id	MSTRG.5131;
10	StringTie	exon	27078652	27078762	.	+	.	transcript_	MSTRG.5131.1(	gene_id	MSTRG.5131;
10	StringTie	transcrip	28700862	28770674	.	+	.	transcript_	MSTRG.5157.1	gene_id	MSTRG.5157;
10	StringTie	exon	28700862	28700882	.	+	.	transcript_	MSTRG.5157.1	gene_id	MSTRG.5157;
10	StringTie	exon	28770493	28770674	.	+	.	transcript_	MSTRG.5157.1	gene_id	MSTRG.5157;
10	StringTie	transcrip	28730942	28770679	.	+	.	transcript_	MSTRG.5157.2	gene_id	MSTRG.5157;
10	StringTie	exon	28730942	28730966	.	+	.	transcript_	MSTRG.5157.2	gene_id	MSTRG.5157;
10	StringTie	exon	28770497	28770679	.	+	.	transcript_	MSTRG.5157.2	gene_id	MSTRG.5157;
10	StringTie	transcrip	32457168	32460186	.	+	.	transcript_	MSTRG.5264.1	gene_id	MSTRG.5264;
10	StringTie	exon	32457168	32457219	.	+	.	transcript_	MSTRG.5264.1	gene_id	MSTRG.5264;
10	StringTie	exon	32460015	32460186	.	+	.	transcript_	MSTRG.5264.1	gene_id	MSTRG.5264;
10	StringTie	transcrip	39050169	39051043	.	+	.	transcript_	MSTRG.5344.1	gene_id	MSTRG.5344;
10	StringTie	exon	39050169	39050207	.	+	.	transcript_	MSTRG.5344.1	gene_id	MSTRG.5344;
10	StringTie	exon	39050743	39051043	.	+	.	transcript_	MSTRG.5344.1	gene_id	MSTRG.5344;
10	StringTie	transcrip	43072537	43073070	.	+	.	transcript_	MSTRG.5457.1	gene_id	MSTRG.5457;
10	StringTie	exon	43072537	43072876	.	+	.	transcript_	MSTRG.5457.1	gene_id	MSTRG.5457;
10	StringTie	exon	43073037	43073070	.	+	.	transcript_	MSTRG.5457.1	gene_id	MSTRG.5457;
10	StringTie	transcrip	51611422	51615888	.	+	.	transcript_	MSTRG.5657.1	gene_id	MSTRG.5657;
10	StringTie	exon	51611422	51611582	.	+	.	transcript_	MSTRG.5657.1	gene_id	MSTRG.5657;
10	StringTie	exon	51612685	51615888	.	+	.	transcript_	MSTRG.5657.1	gene_id	MSTRG.5657;
10	StringTie	transcrip	56430856	56566648	.	+	.	transcript_	MSTRG.5730.1	gene_id	MSTRG.5730;
10	StringTie	exon	56430856	56430896	.	+	.	transcript_	MSTRG.5730.1	gene_id	MSTRG.5730;
10	StringTie	exon	56437592	56437672	.	+	.	transcript_	MSTRG.5730.1	gene_id	MSTRG.5730;
10	StringTie	exon	56440798	56440959	.	+	.	transcript_	MSTRG.5730.1	gene_id	MSTRG.5730;
10	StringTie	exon	56466524	56466633	.	+	.	transcript_	MSTRG.5730.1	gene_id	MSTRG.5730;
10	StringTie	exon	56467008	56467139	.	+	.	transcript_	MSTRG.5730.1	gene_id	MSTRG.5730;
10	StringTie	exon	56475584	56475673	.	+	.	transcript_	MSTRG.5730.1	gene_id	MSTRG.5730;

10	StringTie	exon	56477531	56477609	.	+	.	transcript_	MSTRG.5730.1	gene_id	MSTRG.5730;
10	StringTie	exon	56478528	56478719	.	+	.	transcript_	MSTRG.5730.1	gene_id	MSTRG.5730;
10	StringTie	exon	56561004	56566648	.	+	.	transcript_	MSTRG.5730.1	gene_id	MSTRG.5730;
10	StringTie	transcrip	56459935	56566648	.	+	.	transcript_	MSTRG.5730.2	gene_id	MSTRG.5730;
10	StringTie	exon	56459935	56460675	.	+	.	transcript_	MSTRG.5730.2	gene_id	MSTRG.5730;
10	StringTie	exon	56466524	56466633	.	+	.	transcript_	MSTRG.5730.2	gene_id	MSTRG.5730;
10	StringTie	exon	56467008	56467139	.	+	.	transcript_	MSTRG.5730.2	gene_id	MSTRG.5730;
10	StringTie	exon	56475584	56475673	.	+	.	transcript_	MSTRG.5730.2	gene_id	MSTRG.5730;
10	StringTie	exon	56477531	56477609	.	+	.	transcript_	MSTRG.5730.2	gene_id	MSTRG.5730;
10	StringTie	exon	56478528	56478719	.	+	.	transcript_	MSTRG.5730.2	gene_id	MSTRG.5730;
10	StringTie	exon	56547773	56547924	.	+	.	transcript_	MSTRG.5730.2	gene_id	MSTRG.5730;
10	StringTie	exon	56561004	56566648	.	+	.	transcript_	MSTRG.5730.2	gene_id	MSTRG.5730;
10	StringTie	transcrip	56476510	56567237	.	+	.	transcript_	MSTRG.5730.3	gene_id	MSTRG.5730;
10	StringTie	exon	56476510	56476618	.	+	.	transcript_	MSTRG.5730.3	gene_id	MSTRG.5730;
10	StringTie	exon	56477531	56477609	.	+	.	transcript_	MSTRG.5730.3	gene_id	MSTRG.5730;
10	StringTie	exon	56478528	56478719	.	+	.	transcript_	MSTRG.5730.3	gene_id	MSTRG.5730;
10	StringTie	exon	56561004	56567237	.	+	.	transcript_	MSTRG.5730.3	gene_id	MSTRG.5730;
10	StringTie	transcrip	56766168	56771875	.	+	.	transcript_	MSTRG.5724.1	gene_id	MSTRG.5724;
10	StringTie	exon	56766168	56771875	.	+	.	transcript_	MSTRG.5724.1	gene_id	MSTRG.5724;
10	StringTie	transcrip	56766306	56769889	.	+	.	transcript_	MSTRG.5724.2	gene_id	MSTRG.5724;
10	StringTie	exon	56766306	56766438	.	+	.	transcript_	MSTRG.5724.2	gene_id	MSTRG.5724;
10	StringTie	exon	56768750	56769889	.	+	.	transcript_	MSTRG.5724.2	gene_id	MSTRG.5724;
10	StringTie	transcrip	57601382	57635626	.	+	.	transcript_	MSTRG.5732.1	gene_id	MSTRG.5732;
10	StringTie	exon	57601382	57601804	.	+	.	transcript_	MSTRG.5732.1	gene_id	MSTRG.5732;
10	StringTie	exon	57608052	57608165	.	+	.	transcript_	MSTRG.5732.1	gene_id	MSTRG.5732;
10	StringTie	exon	57635558	57635626	.	+	.	transcript_	MSTRG.5732.1	gene_id	MSTRG.5732;
10	StringTie	transcrip	58078675	58080842	.	+	.	transcript_	MSTRG.5758.1	gene_id	MSTRG.5758;
10	StringTie	exon	58078675	58078709	.	+	.	transcript_	MSTRG.5758.1	gene_id	MSTRG.5758;
10	StringTie	exon	58080542	58080842	.	+	.	transcript_	MSTRG.5758.1	gene_id	MSTRG.5758;
10	StringTie	transcrip	60121314	60129122	.	+	.	transcript_	MSTRG.5774.1	gene_id	MSTRG.5774;
10	StringTie	exon	60121314	60121382	.	+	.	transcript_	MSTRG.5774.1	gene_id	MSTRG.5774;
10	StringTie	exon	60128795	60129122	.	+	.	transcript_	MSTRG.5774.1	gene_id	MSTRG.5774;
10	StringTie	transcrip	69305031	69316011	.	+	.	transcript_	MSTRG.5883.1	gene_id	MSTRG.5883;
10	StringTie	exon	69305031	69305146	.	+	.	transcript_	MSTRG.5883.1	gene_id	MSTRG.5883;
10	StringTie	exon	69315900	69316011	.	+	.	transcript_	MSTRG.5883.1	gene_id	MSTRG.5883;

10	StringTie	transcrip	69316705	69318977	.	+	.	transcript_	MSTRG.5884.1	gene_id	MSTRG.5884;
10	StringTie	exon	69316705	69316821	.	+	.	transcript_	MSTRG.5884.1	gene_id	MSTRG.5884;
10	StringTie	exon	69318511	69318977	.	+	.	transcript_	MSTRG.5884.1	gene_id	MSTRG.5884;
10	StringTie	transcrip	995954	1017525	.	-	.	transcript_	MSTRG.4626.1	gene_id	MSTRG.4626;
10	StringTie	exon	995954	996200	.	-	.	transcript_	MSTRG.4626.1	gene_id	MSTRG.4626;
10	StringTie	exon	1017504	1017525	.	-	.	transcript_	MSTRG.4626.1	gene_id	MSTRG.4626;
10	StringTie	transcrip	8538164	8542956	.	-	.	transcript_	MSTRG.4682.1	gene_id	MSTRG.4682;
10	StringTie	exon	8538164	8538182	.	-	.	transcript_	MSTRG.4682.1	gene_id	MSTRG.4682;
10	StringTie	exon	8542751	8542956	.	-	.	transcript_	MSTRG.4682.1	gene_id	MSTRG.4682;
10	StringTie	transcrip	9243085	9253037	.	-	.	transcript_	MSTRG.4679.1	gene_id	MSTRG.4679;
10	StringTie	exon	9243085	9243117	.	-	.	transcript_	MSTRG.4679.1	gene_id	MSTRG.4679;
10	StringTie	exon	9252789	9253037	.	-	.	transcript_	MSTRG.4679.1	gene_id	MSTRG.4679;
10	StringTie	transcrip	10599145	10609927	.	-	.	transcript_	MSTRG.4726.1	gene_id	MSTRG.4726;
10	StringTie	exon	10599145	10599907	.	-	.	transcript_	MSTRG.4726.1	gene_id	MSTRG.4726;
10	StringTie	exon	10609892	10609927	.	-	.	transcript_	MSTRG.4726.1	gene_id	MSTRG.4726;
10	StringTie	transcrip	10962729	10965688	.	-	.	transcript_	MSTRG.4731.1	gene_id	MSTRG.4731;
10	StringTie	exon	10962729	10962860	.	-	.	transcript_	MSTRG.4731.1	gene_id	MSTRG.4731;
10	StringTie	exon	10965492	10965688	.	-	.	transcript_	MSTRG.4731.1	gene_id	MSTRG.4731;
10	StringTie	transcrip	11390949	11391302	.	-	.	transcript_	MSTRG.4814.1	gene_id	MSTRG.4814;
10	StringTie	exon	11390949	11391053	.	-	.	transcript_	MSTRG.4814.1	gene_id	MSTRG.4814;
10	StringTie	exon	11391102	11391197	.	-	.	transcript_	MSTRG.4814.1	gene_id	MSTRG.4814;
10	StringTie	exon	11391222	11391302	.	-	.	transcript_	MSTRG.4814.1	gene_id	MSTRG.4814;
10	StringTie	transcrip	11391016	11391258	.	-	.	transcript_	MSTRG.4814.2	gene_id	MSTRG.4814;
10	StringTie	exon	11391016	11391125	.	-	.	transcript_	MSTRG.4814.2	gene_id	MSTRG.4814;
10	StringTie	exon	11391150	11391258	.	-	.	transcript_	MSTRG.4814.2	gene_id	MSTRG.4814;
10	StringTie	transcrip	12459445	12471960	.	-	.	transcript_	MSTRG.4837.1	gene_id	MSTRG.4837;
10	StringTie	exon	12459445	12460080	.	-	.	transcript_	MSTRG.4837.1	gene_id	MSTRG.4837;
10	StringTie	exon	12471898	12471960	.	-	.	transcript_	MSTRG.4837.1	gene_id	MSTRG.4837;
10	StringTie	transcrip	12544285	12547432	.	-	.	transcript_	MSTRG.4845.1	gene_id	MSTRG.4845;
10	StringTie	exon	12544285	12544378	.	-	.	transcript_	MSTRG.4845.1	gene_id	MSTRG.4845;
10	StringTie	exon	12547118	12547432	.	-	.	transcript_	MSTRG.4845.1	gene_id	MSTRG.4845;
10	StringTie	transcrip	23601660	23607226	.	-	.	transcript_	MSTRG.5031.5	gene_id	MSTRG.5031;
10	StringTie	exon	23601660	23602492	.	-	.	transcript_	MSTRG.5031.5	gene_id	MSTRG.5031;
10	StringTie	exon	23605068	23605228	.	-	.	transcript_	MSTRG.5031.5	gene_id	MSTRG.5031;
10	StringTie	exon	23606946	23607226	.	-	.	transcript_	MSTRG.5031.5	gene_id	MSTRG.5031;

10	StringTie	transcrip	26230010	26230457	.	-	.	transcript_	MSTRG.5113.1	gene_id	MSTRG.5113;
10	StringTie	exon	26230010	26230303	.	-	.	transcript_	MSTRG.5113.1	gene_id	MSTRG.5113;
10	StringTie	exon	26230436	26230457	.	-	.	transcript_	MSTRG.5113.1	gene_id	MSTRG.5113;
10	StringTie	transcrip	26485606	26500606	.	-	.	transcript_	MSTRG.5105.1	gene_id	MSTRG.5105;
10	StringTie	exon	26485606	26485796	.	-	.	transcript_	MSTRG.5105.1	gene_id	MSTRG.5105;
10	StringTie	exon	26486715	26486828	.	-	.	transcript_	MSTRG.5105.1	gene_id	MSTRG.5105;
10	StringTie	exon	26494397	26494511	.	-	.	transcript_	MSTRG.5105.1	gene_id	MSTRG.5105;
10	StringTie	exon	26500544	26500606	.	-	.	transcript_	MSTRG.5105.1	gene_id	MSTRG.5105;
10	StringTie	transcrip	26629637	26633311	.	-	.	transcript_	MSTRG.5120.1	gene_id	MSTRG.5120;
10	StringTie	exon	26629637	26629916	.	-	.	transcript_	MSTRG.5120.1	gene_id	MSTRG.5120;
10	StringTie	exon	26630820	26631032	.	-	.	transcript_	MSTRG.5120.1	gene_id	MSTRG.5120;
10	StringTie	exon	26631917	26631970	.	-	.	transcript_	MSTRG.5120.1	gene_id	MSTRG.5120;
10	StringTie	exon	26633171	26633311	.	-	.	transcript_	MSTRG.5120.1	gene_id	MSTRG.5120;
10	StringTie	transcrip	27862058	27862352	.	-	.	transcript_	MSTRG.5161.1	gene_id	MSTRG.5161;
10	StringTie	exon	27862058	27862238	.	-	.	transcript_	MSTRG.5161.1	gene_id	MSTRG.5161;
10	StringTie	exon	27862287	27862352	.	-	.	transcript_	MSTRG.5161.1	gene_id	MSTRG.5161;
10	StringTie	transcrip	30584323	30632503	.	-	.	transcript_	MSTRG.5187.1	gene_id	MSTRG.5187;
10	StringTie	exon	30584323	30584436	.	-	.	transcript_	MSTRG.5187.1	gene_id	MSTRG.5187;
10	StringTie	exon	30588839	30588963	.	-	.	transcript_	MSTRG.5187.1	gene_id	MSTRG.5187;
10	StringTie	exon	30632462	30632503	.	-	.	transcript_	MSTRG.5187.1	gene_id	MSTRG.5187;
10	StringTie	transcrip	31740071	31742773	.	-	.	transcript_	MSTRG.5227.1	gene_id	MSTRG.5227;
10	StringTie	exon	31740071	31740209	.	-	.	transcript_	MSTRG.5227.1	gene_id	MSTRG.5227;
10	StringTie	exon	31741522	31741632	.	-	.	transcript_	MSTRG.5227.1	gene_id	MSTRG.5227;
10	StringTie	exon	31742742	31742773	.	-	.	transcript_	MSTRG.5227.1	gene_id	MSTRG.5227;
10	StringTie	transcrip	31742832	31747821	.	-	.	transcript_	MSTRG.5228.1	gene_id	MSTRG.5228;
10	StringTie	exon	31742832	31742914	.	-	.	transcript_	MSTRG.5228.1	gene_id	MSTRG.5228;
10	StringTie	exon	31744965	31745101	.	-	.	transcript_	MSTRG.5228.1	gene_id	MSTRG.5228;
10	StringTie	exon	31747626	31747821	.	-	.	transcript_	MSTRG.5228.1	gene_id	MSTRG.5228;
10	StringTie	transcrip	33865644	33866336	.	-	.	transcript_	MSTRG.5317.1	gene_id	MSTRG.5317;
10	StringTie	exon	33865644	33865867	.	-	.	transcript_	MSTRG.5317.1	gene_id	MSTRG.5317;
10	StringTie	exon	33866194	33866336	.	-	.	transcript_	MSTRG.5317.1	gene_id	MSTRG.5317;
10	StringTie	transcrip	39845691	39895175	.	-	.	transcript_	MSTRG.5355.1	gene_id	MSTRG.5355;
10	StringTie	exon	39845691	39845724	.	-	.	transcript_	MSTRG.5355.1	gene_id	MSTRG.5355;
10	StringTie	exon	39894748	39895175	.	-	.	transcript_	MSTRG.5355.1	gene_id	MSTRG.5355;
10	StringTie	transcrip	39893438	39895175	.	-	.	transcript_	MSTRG.5355.2	gene_id	MSTRG.5355;

10	StringTie	exon	39893438	39893837	.	-	.	transcript_	MSTRG.5355.2	gene_id	MSTRG.5355;
10	StringTie	exon	39893917	39895175	.	-	.	transcript_	MSTRG.5355.2	gene_id	MSTRG.5355;
10	StringTie	transcrip	40881038	40888265	.	-	.	transcript_	MSTRG.5376.1	gene_id	MSTRG.5376;
10	StringTie	exon	40881038	40882087	.	-	.	transcript_	MSTRG.5376.1	gene_id	MSTRG.5376;
10	StringTie	exon	40884706	40888265	.	-	.	transcript_	MSTRG.5376.1	gene_id	MSTRG.5376;
10	StringTie	transcrip	41784429	41787530	.	-	.	transcript_	MSTRG.5391.1	gene_id	MSTRG.5391;
10	StringTie	exon	41784429	41784999	.	-	.	transcript_	MSTRG.5391.1	gene_id	MSTRG.5391;
10	StringTie	exon	41787418	41787530	.	-	.	transcript_	MSTRG.5391.1	gene_id	MSTRG.5391;
10	StringTie	transcrip	41893847	42014204	.	-	.	transcript_	MSTRG.5446.1	gene_id	MSTRG.5446;
10	StringTie	exon	41893847	41897590	.	-	.	transcript_	MSTRG.5446.1	gene_id	MSTRG.5446;
10	StringTie	exon	41943202	41943309	.	-	.	transcript_	MSTRG.5446.1	gene_id	MSTRG.5446;
10	StringTie	exon	42014162	42014204	.	-	.	transcript_	MSTRG.5446.1	gene_id	MSTRG.5446;
10	StringTie	transcrip	41895201	42013483	.	-	.	transcript_	MSTRG.5446.2	gene_id	MSTRG.5446;
10	StringTie	exon	41895201	41897590	.	-	.	transcript_	MSTRG.5446.2	gene_id	MSTRG.5446;
10	StringTie	exon	41943202	41943309	.	-	.	transcript_	MSTRG.5446.2	gene_id	MSTRG.5446;
10	StringTie	exon	42013394	42013483	.	-	.	transcript_	MSTRG.5446.2	gene_id	MSTRG.5446;
10	StringTie	transcrip	42256119	42261528	.	-	.	transcript_	MSTRG.5437.1	gene_id	MSTRG.5437;
10	StringTie	exon	42256119	42256324	.	-	.	transcript_	MSTRG.5437.1	gene_id	MSTRG.5437;
10	StringTie	exon	42261218	42261528	.	-	.	transcript_	MSTRG.5437.1	gene_id	MSTRG.5437;
10	StringTie	transcrip	43517519	43517809	.	-	.	transcript_	MSTRG.5473.1	gene_id	MSTRG.5473;
10	StringTie	exon	43517519	43517629	.	-	.	transcript_	MSTRG.5473.1	gene_id	MSTRG.5473;
10	StringTie	exon	43517699	43517809	.	-	.	transcript_	MSTRG.5473.1	gene_id	MSTRG.5473;
10	StringTie	transcrip	44025919	44026316	.	-	.	transcript_	MSTRG.5476.1	gene_id	MSTRG.5476;
10	StringTie	exon	44025919	44026190	.	-	.	transcript_	MSTRG.5476.1	gene_id	MSTRG.5476;
10	StringTie	exon	44026230	44026316	.	-	.	transcript_	MSTRG.5476.1	gene_id	MSTRG.5476;
10	StringTie	transcrip	44886440	44888591	.	-	.	transcript_	MSTRG.5518.1	gene_id	MSTRG.5518;
10	StringTie	exon	44886440	44886642	.	-	.	transcript_	MSTRG.5518.1	gene_id	MSTRG.5518;
10	StringTie	exon	44888385	44888591	.	-	.	transcript_	MSTRG.5518.1	gene_id	MSTRG.5518;
10	StringTie	transcrip	48035808	48043352	.	-	.	transcript_	MSTRG.5593.1	gene_id	MSTRG.5593;
10	StringTie	exon	48035808	48036960	.	-	.	transcript_	MSTRG.5593.1	gene_id	MSTRG.5593;
10	StringTie	exon	48038503	48038612	.	-	.	transcript_	MSTRG.5593.1	gene_id	MSTRG.5593;
10	StringTie	exon	48039236	48039597	.	-	.	transcript_	MSTRG.5593.1	gene_id	MSTRG.5593;
10	StringTie	exon	48043188	48043352	.	-	.	transcript_	MSTRG.5593.1	gene_id	MSTRG.5593;
10	StringTie	transcrip	48335134	48413588	.	-	.	transcript_	MSTRG.5599.1	gene_id	MSTRG.5599;
10	StringTie	exon	48335134	48335149	.	-	.	transcript_	MSTRG.5599.1	gene_id	MSTRG.5599;

10	StringTie	exon	48412624	48413588	.	-	.	transcript_	MSTRG.5599.1;	gene_id	MSTRG.5599;
10	StringTie	transcrip	50361299	50363725	.	-	.	transcript_	MSTRG.5638.1;	gene_id	MSTRG.5638;
10	StringTie	exon	50361299	50362390	.	-	.	transcript_	MSTRG.5638.1;	gene_id	MSTRG.5638;
10	StringTie	exon	50362740	50363725	.	-	.	transcript_	MSTRG.5638.1;	gene_id	MSTRG.5638;
10	StringTie	transcrip	50361614	50362993	.	-	.	transcript_	MSTRG.5638.2;	gene_id	MSTRG.5638;
10	StringTie	exon	50361614	50362259	.	-	.	transcript_	MSTRG.5638.2;	gene_id	MSTRG.5638;
10	StringTie	exon	50362740	50362993	.	-	.	transcript_	MSTRG.5638.2;	gene_id	MSTRG.5638;
10	StringTie	transcrip	50531892	50539833	.	-	.	transcript_	MSTRG.5647.1;	gene_id	MSTRG.5647;
10	StringTie	exon	50531892	50532412	.	-	.	transcript_	MSTRG.5647.1;	gene_id	MSTRG.5647;
10	StringTie	exon	50537258	50537337	.	-	.	transcript_	MSTRG.5647.1;	gene_id	MSTRG.5647;
10	StringTie	exon	50539548	50539833	.	-	.	transcript_	MSTRG.5647.1;	gene_id	MSTRG.5647;
10	StringTie	transcrip	50776803	50777902	.	-	.	transcript_	MSTRG.5660.1;	gene_id	MSTRG.5660;
10	StringTie	exon	50776803	50777133	.	-	.	transcript_	MSTRG.5660.1;	gene_id	MSTRG.5660;
10	StringTie	exon	50777434	50777902	.	-	.	transcript_	MSTRG.5660.1;	gene_id	MSTRG.5660;
10	StringTie	transcrip	57091461	57114882	.	-	.	transcript_	MSTRG.5737.1;	gene_id	MSTRG.5737;
10	StringTie	exon	57091461	57092095	.	-	.	transcript_	MSTRG.5737.1;	gene_id	MSTRG.5737;
10	StringTie	exon	57114798	57114882	.	-	.	transcript_	MSTRG.5737.1;	gene_id	MSTRG.5737;
10	StringTie	transcrip	64949390	64952879	.	-	.	transcript_	MSTRG.5819.1;	gene_id	MSTRG.5819;
10	StringTie	exon	64949390	64951204	.	-	.	transcript_	MSTRG.5819.1;	gene_id	MSTRG.5819;
10	StringTie	exon	64952234	64952879	.	-	.	transcript_	MSTRG.5819.1;	gene_id	MSTRG.5819;
10	StringTie	transcrip	64974754	64989112	.	-	.	transcript_	MSTRG.5820.1;	gene_id	MSTRG.5820;
10	StringTie	exon	64974754	64975239	.	-	.	transcript_	MSTRG.5820.1;	gene_id	MSTRG.5820;
10	StringTie	exon	64989053	64989112	.	-	.	transcript_	MSTRG.5820.1;	gene_id	MSTRG.5820;
10	StringTie	transcrip	65408718	65411094	.	-	.	transcript_	MSTRG.5836.1;	gene_id	MSTRG.5836;
10	StringTie	exon	65408718	65409290	.	-	.	transcript_	MSTRG.5836.1;	gene_id	MSTRG.5836;
10	StringTie	exon	65410650	65411094	.	-	.	transcript_	MSTRG.5836.1;	gene_id	MSTRG.5836;
10	StringTie	transcrip	68531078	68538916	.	-	.	transcript_	MSTRG.5875.1;	gene_id	MSTRG.5875;
10	StringTie	exon	68531078	68531397	.	-	.	transcript_	MSTRG.5875.1;	gene_id	MSTRG.5875;
10	StringTie	exon	68538758	68538916	.	-	.	transcript_	MSTRG.5875.1;	gene_id	MSTRG.5875;
11	StringTie	transcrip	2212356	2214590	.	+	.	transcript_	MSTRG.5930.1;	gene_id	MSTRG.5930;
11	StringTie	exon	2212356	2212831	.	+	.	transcript_	MSTRG.5930.1;	gene_id	MSTRG.5930;
11	StringTie	exon	2213008	2214590	.	+	.	transcript_	MSTRG.5930.1;	gene_id	MSTRG.5930;
11	StringTie	transcrip	7142547	7144813	.	+	.	transcript_	MSTRG.6025.1;	gene_id	MSTRG.6025;
11	StringTie	exon	7142547	7142897	.	+	.	transcript_	MSTRG.6025.1;	gene_id	MSTRG.6025;
11	StringTie	exon	7144334	7144813	.	+	.	transcript_	MSTRG.6025.1;	gene_id	MSTRG.6025;

11	StringTie	transcrip	8352956	8358076	.	+	.	transcript_	MSTRG.6088.1	gene_id	MSTRG.6088;
11	StringTie	exon	8352956	8354750	.	+	.	transcript_	MSTRG.6088.1	gene_id	MSTRG.6088;
11	StringTie	exon	8355045	8358076	.	+	.	transcript_	MSTRG.6088.1	gene_id	MSTRG.6088;
11	StringTie	transcrip	9623528	9624428	.	+	.	transcript_	MSTRG.6126.1	gene_id	MSTRG.6126;
11	StringTie	exon	9623528	9624053	.	+	.	transcript_	MSTRG.6126.1	gene_id	MSTRG.6126;
11	StringTie	exon	9624128	9624428	.	+	.	transcript_	MSTRG.6126.1	gene_id	MSTRG.6126;
11	StringTie	transcrip	9837756	9838771	.	+	.	transcript_	MSTRG.6127.1	gene_id	MSTRG.6127;
11	StringTie	exon	9837756	9838207	.	+	.	transcript_	MSTRG.6127.1	gene_id	MSTRG.6127;
11	StringTie	exon	9838534	9838771	.	+	.	transcript_	MSTRG.6127.1	gene_id	MSTRG.6127;
11	StringTie	transcrip	10578304	10580178	.	+	.	transcript_	MSTRG.6107.1	gene_id	MSTRG.6107;
11	StringTie	exon	10578304	10578584	.	+	.	transcript_	MSTRG.6107.1	gene_id	MSTRG.6107;
11	StringTie	exon	10579292	10580178	.	+	.	transcript_	MSTRG.6107.1	gene_id	MSTRG.6107;
11	StringTie	transcrip	12345104	12356633	.	+	.	transcript_	MSTRG.6161.1	gene_id	MSTRG.6161;
11	StringTie	exon	12345104	12345315	.	+	.	transcript_	MSTRG.6161.1	gene_id	MSTRG.6161;
11	StringTie	exon	12347101	12347171	.	+	.	transcript_	MSTRG.6161.1	gene_id	MSTRG.6161;
11	StringTie	exon	12350616	12350675	.	+	.	transcript_	MSTRG.6161.1	gene_id	MSTRG.6161;
11	StringTie	exon	12355879	12356027	.	+	.	transcript_	MSTRG.6161.1	gene_id	MSTRG.6161;
11	StringTie	exon	12356426	12356633	.	+	.	transcript_	MSTRG.6161.1	gene_id	MSTRG.6161;
11	StringTie	transcrip	12345139	12357531	.	+	.	transcript_	MSTRG.6161.2	gene_id	MSTRG.6161;
11	StringTie	exon	12345139	12345315	.	+	.	transcript_	MSTRG.6161.2	gene_id	MSTRG.6161;
11	StringTie	exon	12347101	12347171	.	+	.	transcript_	MSTRG.6161.2	gene_id	MSTRG.6161;
11	StringTie	exon	12350616	12350675	.	+	.	transcript_	MSTRG.6161.2	gene_id	MSTRG.6161;
11	StringTie	exon	12350852	12350916	.	+	.	transcript_	MSTRG.6161.2	gene_id	MSTRG.6161;
11	StringTie	exon	12352041	12352169	.	+	.	transcript_	MSTRG.6161.2	gene_id	MSTRG.6161;
11	StringTie	exon	12353152	12353270	.	+	.	transcript_	MSTRG.6161.2	gene_id	MSTRG.6161;
11	StringTie	exon	12355879	12356027	.	+	.	transcript_	MSTRG.6161.2	gene_id	MSTRG.6161;
11	StringTie	exon	12356426	12357531	.	+	.	transcript_	MSTRG.6161.2	gene_id	MSTRG.6161;
11	StringTie	transcrip	15410728	15411879	.	+	.	transcript_	MSTRG.6231.1	gene_id	MSTRG.6231;
11	StringTie	exon	15410728	15411044	.	+	.	transcript_	MSTRG.6231.1	gene_id	MSTRG.6231;
11	StringTie	exon	15411824	15411879	.	+	.	transcript_	MSTRG.6231.1	gene_id	MSTRG.6231;
11	StringTie	transcrip	16086880	16101400	.	+	.	transcript_	MSTRG.6289.3	gene_id	MSTRG.6289;
11	StringTie	exon	16086880	16087791	.	+	.	transcript_	MSTRG.6289.3	gene_id	MSTRG.6289;
11	StringTie	exon	16099036	16101400	.	+	.	transcript_	MSTRG.6289.3	gene_id	MSTRG.6289;
11	StringTie	transcrip	17683032	17684702	.	+	.	transcript_	MSTRG.6303.1	gene_id	MSTRG.6303;
11	StringTie	exon	17683032	17683282	.	+	.	transcript_	MSTRG.6303.1	gene_id	MSTRG.6303;

11	StringTie	exon	17684669	17684702	.	+	.	transcript_	MSTRG.6303.1	gene_id	MSTRG.6303;
11	StringTie	transcrip	17690399	17833389	.	+	.	transcript_	MSTRG.6323.1	gene_id	MSTRG.6323;
11	StringTie	exon	17690399	17690489	.	+	.	transcript_	MSTRG.6323.1	gene_id	MSTRG.6323;
11	StringTie	exon	17737683	17737777	.	+	.	transcript_	MSTRG.6323.1	gene_id	MSTRG.6323;
11	StringTie	exon	17745806	17745872	.	+	.	transcript_	MSTRG.6323.1	gene_id	MSTRG.6323;
11	StringTie	exon	17756989	17757085	.	+	.	transcript_	MSTRG.6323.1	gene_id	MSTRG.6323;
11	StringTie	exon	17760988	17761081	.	+	.	transcript_	MSTRG.6323.1	gene_id	MSTRG.6323;
11	StringTie	exon	17761434	17761545	.	+	.	transcript_	MSTRG.6323.1	gene_id	MSTRG.6323;
11	StringTie	exon	17778900	17779114	.	+	.	transcript_	MSTRG.6323.1	gene_id	MSTRG.6323;
11	StringTie	exon	17781637	17781703	.	+	.	transcript_	MSTRG.6323.1	gene_id	MSTRG.6323;
11	StringTie	exon	17833023	17833389	.	+	.	transcript_	MSTRG.6323.1	gene_id	MSTRG.6323;
11	StringTie	transcrip	17737682	17760936	.	+	.	transcript_	MSTRG.6323.2	gene_id	MSTRG.6323;
11	StringTie	exon	17737682	17737777	.	+	.	transcript_	MSTRG.6323.2	gene_id	MSTRG.6323;
11	StringTie	exon	17745806	17745872	.	+	.	transcript_	MSTRG.6323.2	gene_id	MSTRG.6323;
11	StringTie	exon	17756989	17757085	.	+	.	transcript_	MSTRG.6323.2	gene_id	MSTRG.6323;
11	StringTie	exon	17760786	17760936	.	+	.	transcript_	MSTRG.6323.2	gene_id	MSTRG.6323;
11	StringTie	transcrip	17757764	17772559	.	+	.	transcript_	MSTRG.6323.4	gene_id	MSTRG.6323;
11	StringTie	exon	17757764	17758790	.	+	.	transcript_	MSTRG.6323.4	gene_id	MSTRG.6323;
11	StringTie	exon	17760988	17761081	.	+	.	transcript_	MSTRG.6323.4	gene_id	MSTRG.6323;
11	StringTie	exon	17761434	17761545	.	+	.	transcript_	MSTRG.6323.4	gene_id	MSTRG.6323;
11	StringTie	exon	17771089	17772559	.	+	.	transcript_	MSTRG.6323.4	gene_id	MSTRG.6323;
11	StringTie	transcrip	17758675	17807754	.	+	.	transcript_	MSTRG.6323.5	gene_id	MSTRG.6323;
11	StringTie	exon	17758675	17758790	.	+	.	transcript_	MSTRG.6323.5	gene_id	MSTRG.6323;
11	StringTie	exon	17760988	17761081	.	+	.	transcript_	MSTRG.6323.5	gene_id	MSTRG.6323;
11	StringTie	exon	17761434	17761545	.	+	.	transcript_	MSTRG.6323.5	gene_id	MSTRG.6323;
11	StringTie	exon	17778900	17779114	.	+	.	transcript_	MSTRG.6323.5	gene_id	MSTRG.6323;
11	StringTie	exon	17781637	17781703	.	+	.	transcript_	MSTRG.6323.5	gene_id	MSTRG.6323;
11	StringTie	exon	17807495	17807754	.	+	.	transcript_	MSTRG.6323.5	gene_id	MSTRG.6323;
11	StringTie	transcrip	17760974	17766133	.	+	.	transcript_	MSTRG.6323.6	gene_id	MSTRG.6323;
11	StringTie	exon	17760974	17761081	.	+	.	transcript_	MSTRG.6323.6	gene_id	MSTRG.6323;
11	StringTie	exon	17761434	17761545	.	+	.	transcript_	MSTRG.6323.6	gene_id	MSTRG.6323;
11	StringTie	exon	17765228	17766133	.	+	.	transcript_	MSTRG.6323.6	gene_id	MSTRG.6323;
11	StringTie	transcrip	17878829	17924326	.	+	.	transcript_	MSTRG.6312.1	gene_id	MSTRG.6312;
11	StringTie	exon	17878829	17878988	.	+	.	transcript_	MSTRG.6312.1	gene_id	MSTRG.6312;
11	StringTie	exon	17919518	17919590	.	+	.	transcript_	MSTRG.6312.1	gene_id	MSTRG.6312;

11	StringTie	exon	17924001	17924326	.	+	.	transcript_	MSTRG.6312.1	gene_id	MSTRG.6312;
11	StringTie	transcrip	22185751	22200938	.	+	.	transcript_	MSTRG.6429.1	gene_id	MSTRG.6429;
11	StringTie	exon	22185751	22185839	.	+	.	transcript_	MSTRG.6429.1	gene_id	MSTRG.6429;
11	StringTie	exon	22186573	22186660	.	+	.	transcript_	MSTRG.6429.1	gene_id	MSTRG.6429;
11	StringTie	exon	22200715	22200938	.	+	.	transcript_	MSTRG.6429.1	gene_id	MSTRG.6429;
11	StringTie	transcrip	23914858	23927125	.	+	.	transcript_	MSTRG.6486.1	gene_id	MSTRG.6486;
11	StringTie	exon	23914858	23915212	.	+	.	transcript_	MSTRG.6486.1	gene_id	MSTRG.6486;
11	StringTie	exon	23925949	23927125	.	+	.	transcript_	MSTRG.6486.1	gene_id	MSTRG.6486;
11	StringTie	transcrip	23923994	23927125	.	+	.	transcript_	MSTRG.6486.2	gene_id	MSTRG.6486;
11	StringTie	exon	23923994	23924134	.	+	.	transcript_	MSTRG.6486.2	gene_id	MSTRG.6486;
11	StringTie	exon	23925949	23927125	.	+	.	transcript_	MSTRG.6486.2	gene_id	MSTRG.6486;
11	StringTie	transcrip	52242370	52433668	.	+	.	transcript_	MSTRG.6692.1	gene_id	MSTRG.6692;
11	StringTie	exon	52242370	52242458	.	+	.	transcript_	MSTRG.6692.1	gene_id	MSTRG.6692;
11	StringTie	exon	52310948	52311047	.	+	.	transcript_	MSTRG.6692.1	gene_id	MSTRG.6692;
11	StringTie	exon	52424360	52424502	.	+	.	transcript_	MSTRG.6692.1	gene_id	MSTRG.6692;
11	StringTie	exon	52427159	52427222	.	+	.	transcript_	MSTRG.6692.1	gene_id	MSTRG.6692;
11	StringTie	exon	52433639	52433668	.	+	.	transcript_	MSTRG.6692.1	gene_id	MSTRG.6692;
11	StringTie	transcrip	62142573	62149831	.	+	.	transcript_	MSTRG.6812.1	gene_id	MSTRG.6812;
11	StringTie	exon	62142573	62142626	.	+	.	transcript_	MSTRG.6812.1	gene_id	MSTRG.6812;
11	StringTie	exon	62146081	62149831	.	+	.	transcript_	MSTRG.6812.1	gene_id	MSTRG.6812;
11	StringTie	transcrip	73878334	73884346	.	+	.	transcript_	MSTRG.6920.1	gene_id	MSTRG.6920;
11	StringTie	exon	73878334	73878784	.	+	.	transcript_	MSTRG.6920.1	gene_id	MSTRG.6920;
11	StringTie	exon	73883760	73884346	.	+	.	transcript_	MSTRG.6920.1	gene_id	MSTRG.6920;
11	StringTie	transcrip	76716846	76724724	.	+	.	transcript_	MSTRG.6956.1	gene_id	MSTRG.6956;
11	StringTie	exon	76716846	76717230	.	+	.	transcript_	MSTRG.6956.1	gene_id	MSTRG.6956;
11	StringTie	exon	76724563	76724724	.	+	.	transcript_	MSTRG.6956.1	gene_id	MSTRG.6956;
11	StringTie	transcrip	77355702	77358520	.	+	.	transcript_	MSTRG.6970.1	gene_id	MSTRG.6970;
11	StringTie	exon	77355702	77355812	.	+	.	transcript_	MSTRG.6970.1	gene_id	MSTRG.6970;
11	StringTie	exon	77357876	77357990	.	+	.	transcript_	MSTRG.6970.1	gene_id	MSTRG.6970;
11	StringTie	exon	77358163	77358520	.	+	.	transcript_	MSTRG.6970.1	gene_id	MSTRG.6970;
11	StringTie	transcrip	500080	503336	.	-	.	transcript_	MSTRG.5898.1	gene_id	MSTRG.5898;
11	StringTie	exon	500080	500736	.	-	.	transcript_	MSTRG.5898.1	gene_id	MSTRG.5898;
11	StringTie	exon	501139	503336	.	-	.	transcript_	MSTRG.5898.1	gene_id	MSTRG.5898;
11	StringTie	transcrip	5477911	5486807	.	-	.	transcript_	MSTRG.6000.1	gene_id	MSTRG.6000;
11	StringTie	exon	5477911	5480439	.	-	.	transcript_	MSTRG.6000.1	gene_id	MSTRG.6000;

11	StringTie	exon	5485954	5486096	.	-	.	transcript_	MSTRG.6000.1	gene_id	MSTRG.6000;
11	StringTie	exon	5486426	5486807	.	-	.	transcript_	MSTRG.6000.1	gene_id	MSTRG.6000;
11	StringTie	transcrip	5477952	5486807	.	-	.	transcript_	MSTRG.6000.2	gene_id	MSTRG.6000;
11	StringTie	exon	5477952	5482603	.	-	.	transcript_	MSTRG.6000.2	gene_id	MSTRG.6000;
11	StringTie	exon	5485954	5486096	.	-	.	transcript_	MSTRG.6000.2	gene_id	MSTRG.6000;
11	StringTie	exon	5486426	5486807	.	-	.	transcript_	MSTRG.6000.2	gene_id	MSTRG.6000;
11	StringTie	transcrip	7071362	7085888	.	-	.	transcript_	MSTRG.6020.1	gene_id	MSTRG.6020;
11	StringTie	exon	7071362	7071544	.	-	.	transcript_	MSTRG.6020.1	gene_id	MSTRG.6020;
11	StringTie	exon	7085655	7085888	.	-	.	transcript_	MSTRG.6020.1	gene_id	MSTRG.6020;
11	StringTie	transcrip	8407079	8496369	.	-	.	transcript_	MSTRG.6089.1	gene_id	MSTRG.6089;
11	StringTie	exon	8407079	8407103	.	-	.	transcript_	MSTRG.6089.1	gene_id	MSTRG.6089;
11	StringTie	exon	8493614	8496369	.	-	.	transcript_	MSTRG.6089.1	gene_id	MSTRG.6089;
11	StringTie	transcrip	8425852	8487729	.	-	.	transcript_	MSTRG.6090.1	gene_id	MSTRG.6090;
11	StringTie	exon	8425852	8425869	.	-	.	transcript_	MSTRG.6090.1	gene_id	MSTRG.6090;
11	StringTie	exon	8486536	8487729	.	-	.	transcript_	MSTRG.6090.1	gene_id	MSTRG.6090;
11	StringTie	transcrip	8638052	8638759	.	-	.	transcript_	MSTRG.6091.1	gene_id	MSTRG.6091;
11	StringTie	exon	8638052	8638087	.	-	.	transcript_	MSTRG.6091.1	gene_id	MSTRG.6091;
11	StringTie	exon	8638363	8638759	.	-	.	transcript_	MSTRG.6091.1	gene_id	MSTRG.6091;
11	StringTie	transcrip	14944853	15271537	.	-	.	transcript_	MSTRG.6211.1	gene_id	MSTRG.6211;
11	StringTie	exon	14944853	14944889	.	-	.	transcript_	MSTRG.6211.1	gene_id	MSTRG.6211;
11	StringTie	exon	15198209	15198284	.	-	.	transcript_	MSTRG.6211.1	gene_id	MSTRG.6211;
11	StringTie	exon	15271403	15271537	.	-	.	transcript_	MSTRG.6211.1	gene_id	MSTRG.6211;
11	StringTie	transcrip	17711787	17714725	.	-	.	transcript_	MSTRG.6326.1	gene_id	MSTRG.6326;
11	StringTie	exon	17711787	17711851	.	-	.	transcript_	MSTRG.6326.1	gene_id	MSTRG.6326;
11	StringTie	exon	17712847	17714725	.	-	.	transcript_	MSTRG.6326.1	gene_id	MSTRG.6326;
11	StringTie	transcrip	18412516	18419074	.	-	.	transcript_	MSTRG.6359.1	gene_id	MSTRG.6359;
11	StringTie	exon	18412516	18412794	.	-	.	transcript_	MSTRG.6359.1	gene_id	MSTRG.6359;
11	StringTie	exon	18418085	18419074	.	-	.	transcript_	MSTRG.6359.1	gene_id	MSTRG.6359;
11	StringTie	transcrip	18424266	18461647	.	-	.	transcript_	MSTRG.6360.1	gene_id	MSTRG.6360;
11	StringTie	exon	18424266	18426102	.	-	.	transcript_	MSTRG.6360.1	gene_id	MSTRG.6360;
11	StringTie	exon	18459324	18459444	.	-	.	transcript_	MSTRG.6360.1	gene_id	MSTRG.6360;
11	StringTie	exon	18461318	18461647	.	-	.	transcript_	MSTRG.6360.1	gene_id	MSTRG.6360;
11	StringTie	transcrip	19621240	19629345	.	-	.	transcript_	MSTRG.6404.1	gene_id	MSTRG.6404;
11	StringTie	exon	19621240	19621313	.	-	.	transcript_	MSTRG.6404.1	gene_id	MSTRG.6404;
11	StringTie	exon	19628947	19629345	.	-	.	transcript_	MSTRG.6404.1	gene_id	MSTRG.6404;

11	StringTie	transcrip	22283324	22284418	.	-	.	transcript_	MSTRG.6450.1	gene_id	MSTRG.6450;
11	StringTie	exon	22283324	22283655	.	-	.	transcript_	MSTRG.6450.1	gene_id	MSTRG.6450;
11	StringTie	exon	22284352	22284418	.	-	.	transcript_	MSTRG.6450.1	gene_id	MSTRG.6450;
11	StringTie	transcrip	22435954	22449656	.	-	.	transcript_	MSTRG.6434.1	gene_id	MSTRG.6434;
11	StringTie	exon	22435954	22436161	.	-	.	transcript_	MSTRG.6434.1	gene_id	MSTRG.6434;
11	StringTie	exon	22449504	22449656	.	-	.	transcript_	MSTRG.6434.1	gene_id	MSTRG.6434;
11	StringTie	transcrip	22477099	22489942	.	-	.	transcript_	MSTRG.6444.1	gene_id	MSTRG.6444;
11	StringTie	exon	22477099	22478223	.	-	.	transcript_	MSTRG.6444.1	gene_id	MSTRG.6444;
11	StringTie	exon	22489857	22489942	.	-	.	transcript_	MSTRG.6444.1	gene_id	MSTRG.6444;
11	StringTie	transcrip	23627015	23629086	.	-	.	transcript_	MSTRG.6491.1	gene_id	MSTRG.6491;
11	StringTie	exon	23627015	23627585	.	-	.	transcript_	MSTRG.6491.1	gene_id	MSTRG.6491;
11	StringTie	exon	23628270	23629086	.	-	.	transcript_	MSTRG.6491.1	gene_id	MSTRG.6491;
11	StringTie	transcrip	23879790	23913154	.	-	.	transcript_	MSTRG.6492.1	gene_id	MSTRG.6492;
11	StringTie	exon	23879790	23880101	.	-	.	transcript_	MSTRG.6492.1	gene_id	MSTRG.6492;
11	StringTie	exon	23912820	23913154	.	-	.	transcript_	MSTRG.6492.1	gene_id	MSTRG.6492;
11	StringTie	transcrip	23892071	23913096	.	-	.	transcript_	MSTRG.6492.2	gene_id	MSTRG.6492;
11	StringTie	exon	23892071	23893142	.	-	.	transcript_	MSTRG.6492.2	gene_id	MSTRG.6492;
11	StringTie	exon	23893586	23893673	.	-	.	transcript_	MSTRG.6492.2	gene_id	MSTRG.6492;
11	StringTie	exon	23912820	23913096	.	-	.	transcript_	MSTRG.6492.2	gene_id	MSTRG.6492;
11	StringTie	transcrip	23893496	23913141	.	-	.	transcript_	MSTRG.6492.3	gene_id	MSTRG.6492;
11	StringTie	exon	23893496	23893673	.	-	.	transcript_	MSTRG.6492.3	gene_id	MSTRG.6492;
11	StringTie	exon	23896369	23896405	.	-	.	transcript_	MSTRG.6492.3	gene_id	MSTRG.6492;
11	StringTie	exon	23912820	23913141	.	-	.	transcript_	MSTRG.6492.3	gene_id	MSTRG.6492;
11	StringTie	transcrip	29089010	29089861	.	-	.	transcript_	MSTRG.6531.1	gene_id	MSTRG.6531;
11	StringTie	exon	29089010	29089069	.	-	.	transcript_	MSTRG.6531.1	gene_id	MSTRG.6531;
11	StringTie	exon	29089669	29089861	.	-	.	transcript_	MSTRG.6531.1	gene_id	MSTRG.6531;
11	StringTie	transcrip	33256322	33258536	.	-	.	transcript_	MSTRG.6544.1	gene_id	MSTRG.6544;
11	StringTie	exon	33256322	33257768	.	-	.	transcript_	MSTRG.6544.1	gene_id	MSTRG.6544;
11	StringTie	exon	33258448	33258536	.	-	.	transcript_	MSTRG.6544.1	gene_id	MSTRG.6544;
11	StringTie	transcrip	49523776	49536106	.	-	.	transcript_	MSTRG.6726.1	gene_id	MSTRG.6726;
11	StringTie	exon	49523776	49523802	.	-	.	transcript_	MSTRG.6726.1	gene_id	MSTRG.6726;
11	StringTie	exon	49535820	49536106	.	-	.	transcript_	MSTRG.6726.1	gene_id	MSTRG.6726;
11	StringTie	transcrip	52159783	52160553	.	-	.	transcript_	MSTRG.6684.2	gene_id	MSTRG.6684;
11	StringTie	exon	52159783	52159999	.	-	.	transcript_	MSTRG.6684.2	gene_id	MSTRG.6684;
11	StringTie	exon	52160246	52160553	.	-	.	transcript_	MSTRG.6684.2	gene_id	MSTRG.6684;

11	StringTie	transcrip	52159783	52160680	.	-	.	transcript_	MSTRG.6684.1	gene_id	MSTRG.6684;
11	StringTie	exon	52159783	52159999	.	-	.	transcript_	MSTRG.6684.1	gene_id	MSTRG.6684;
11	StringTie	exon	52160296	52160680	.	-	.	transcript_	MSTRG.6684.1	gene_id	MSTRG.6684;
11	StringTie	transcrip	76285253	76290498	.	-	.	transcript_	MSTRG.6940.1	gene_id	MSTRG.6940;
11	StringTie	exon	76285253	76285579	.	-	.	transcript_	MSTRG.6940.1	gene_id	MSTRG.6940;
11	StringTie	exon	76290141	76290498	.	-	.	transcript_	MSTRG.6940.1	gene_id	MSTRG.6940;
11	StringTie	transcrip	76626163	76649334	.	-	.	transcript_	MSTRG.6954.2	gene_id	MSTRG.6954;
11	StringTie	exon	76626163	76628599	.	-	.	transcript_	MSTRG.6954.2	gene_id	MSTRG.6954;
11	StringTie	exon	76648920	76649334	.	-	.	transcript_	MSTRG.6954.2	gene_id	MSTRG.6954;
11	StringTie	transcrip	76626178	76647456	.	-	.	transcript_	MSTRG.6954.3	gene_id	MSTRG.6954;
11	StringTie	exon	76626178	76628599	.	-	.	transcript_	MSTRG.6954.3	gene_id	MSTRG.6954;
11	StringTie	exon	76647178	76647456	.	-	.	transcript_	MSTRG.6954.3	gene_id	MSTRG.6954;
12	StringTie	transcrip	205476	207729	.	+	.	transcript_	MSTRG.7015.1	gene_id	MSTRG.7015;
12	StringTie	exon	205476	205596	.	+	.	transcript_	MSTRG.7015.1	gene_id	MSTRG.7015;
12	StringTie	exon	206535	207729	.	+	.	transcript_	MSTRG.7015.1	gene_id	MSTRG.7015;
12	StringTie	transcrip	559440	560316	.	+	.	transcript_	MSTRG.7028.1	gene_id	MSTRG.7028;
12	StringTie	exon	559440	559461	.	+	.	transcript_	MSTRG.7028.1	gene_id	MSTRG.7028;
12	StringTie	exon	559923	560316	.	+	.	transcript_	MSTRG.7028.1	gene_id	MSTRG.7028;
12	StringTie	transcrip	1190077	1192134	.	+	.	transcript_	MSTRG.7074.1	gene_id	MSTRG.7074;
12	StringTie	exon	1190077	1190524	.	+	.	transcript_	MSTRG.7074.1	gene_id	MSTRG.7074;
12	StringTie	exon	1191413	1192134	.	+	.	transcript_	MSTRG.7074.1	gene_id	MSTRG.7074;
12	StringTie	transcrip	1562157	1567167	.	+	.	transcript_	MSTRG.7083.1	gene_id	MSTRG.7083;
12	StringTie	exon	1562157	1562237	.	+	.	transcript_	MSTRG.7083.1	gene_id	MSTRG.7083;
12	StringTie	exon	1566195	1566313	.	+	.	transcript_	MSTRG.7083.1	gene_id	MSTRG.7083;
12	StringTie	exon	1566965	1567167	.	+	.	transcript_	MSTRG.7083.1	gene_id	MSTRG.7083;
12	StringTie	transcrip	1562157	1567527	.	+	.	transcript_	MSTRG.7083.2	gene_id	MSTRG.7083;
12	StringTie	exon	1562157	1562237	.	+	.	transcript_	MSTRG.7083.2	gene_id	MSTRG.7083;
12	StringTie	exon	1563644	1563703	.	+	.	transcript_	MSTRG.7083.2	gene_id	MSTRG.7083;
12	StringTie	exon	1566195	1566313	.	+	.	transcript_	MSTRG.7083.2	gene_id	MSTRG.7083;
12	StringTie	exon	1566965	1567527	.	+	.	transcript_	MSTRG.7083.2	gene_id	MSTRG.7083;
12	StringTie	transcrip	1563643	1567149	.	+	.	transcript_	MSTRG.7083.3	gene_id	MSTRG.7083;
12	StringTie	exon	1563643	1563703	.	+	.	transcript_	MSTRG.7083.3	gene_id	MSTRG.7083;
12	StringTie	exon	1563966	1564209	.	+	.	transcript_	MSTRG.7083.3	gene_id	MSTRG.7083;
12	StringTie	exon	1564378	1564517	.	+	.	transcript_	MSTRG.7083.3	gene_id	MSTRG.7083;
12	StringTie	exon	1566195	1566313	.	+	.	transcript_	MSTRG.7083.3	gene_id	MSTRG.7083;

12	StringTie	exon	1566965	1567149	.	+	.	transcript_	MSTRG.7083.3	gene_id	MSTRG.7083;
12	StringTie	transcrip	1563960	1567527	.	+	.	transcript_	MSTRG.7083.4	gene_id	MSTRG.7083;
12	StringTie	exon	1563960	1564517	.	+	.	transcript_	MSTRG.7083.4	gene_id	MSTRG.7083;
12	StringTie	exon	1566195	1566313	.	+	.	transcript_	MSTRG.7083.4	gene_id	MSTRG.7083;
12	StringTie	exon	1566965	1567527	.	+	.	transcript_	MSTRG.7083.4	gene_id	MSTRG.7083;
12	StringTie	transcrip	1564021	1567527	.	+	.	transcript_	MSTRG.7083.5	gene_id	MSTRG.7083;
12	StringTie	exon	1564021	1564209	.	+	.	transcript_	MSTRG.7083.5	gene_id	MSTRG.7083;
12	StringTie	exon	1566195	1566313	.	+	.	transcript_	MSTRG.7083.5	gene_id	MSTRG.7083;
12	StringTie	exon	1566965	1567527	.	+	.	transcript_	MSTRG.7083.5	gene_id	MSTRG.7083;
12	StringTie	transcrip	3228696	3233784	.	+	.	transcript_	MSTRG.7135.1	gene_id	MSTRG.7135;
12	StringTie	exon	3228696	3229167	.	+	.	transcript_	MSTRG.7135.1	gene_id	MSTRG.7135;
12	StringTie	exon	3229435	3230295	.	+	.	transcript_	MSTRG.7135.1	gene_id	MSTRG.7135;
12	StringTie	exon	3230954	3233784	.	+	.	transcript_	MSTRG.7135.1	gene_id	MSTRG.7135;
12	StringTie	transcrip	3800153	3803313	.	+	.	transcript_	MSTRG.7158.1	gene_id	MSTRG.7158;
12	StringTie	exon	3800153	3800441	.	+	.	transcript_	MSTRG.7158.1	gene_id	MSTRG.7158;
12	StringTie	exon	3802794	3802891	.	+	.	transcript_	MSTRG.7158.1	gene_id	MSTRG.7158;
12	StringTie	exon	3803205	3803313	.	+	.	transcript_	MSTRG.7158.1	gene_id	MSTRG.7158;
12	StringTie	transcrip	4904642	4968310	.	+	.	transcript_	MSTRG.7174.1	gene_id	MSTRG.7174;
12	StringTie	exon	4904642	4904664	.	+	.	transcript_	MSTRG.7174.1	gene_id	MSTRG.7174;
12	StringTie	exon	4966705	4968310	.	+	.	transcript_	MSTRG.7174.1	gene_id	MSTRG.7174;
12	StringTie	transcrip	6714810	6715812	.	+	.	transcript_	MSTRG.7263.1	gene_id	MSTRG.7263;
12	StringTie	exon	6714810	6714909	.	+	.	transcript_	MSTRG.7263.1	gene_id	MSTRG.7263;
12	StringTie	exon	6715707	6715812	.	+	.	transcript_	MSTRG.7263.1	gene_id	MSTRG.7263;
12	StringTie	transcrip	8378751	8401632	.	+	.	transcript_	MSTRG.7274.1	gene_id	MSTRG.7274;
12	StringTie	exon	8378751	8378789	.	+	.	transcript_	MSTRG.7274.1	gene_id	MSTRG.7274;
12	StringTie	exon	8401071	8401632	.	+	.	transcript_	MSTRG.7274.1	gene_id	MSTRG.7274;
12	StringTie	transcrip	11163074	11163870	.	+	.	transcript_	MSTRG.7330.1	gene_id	MSTRG.7330;
12	StringTie	exon	11163074	11163258	.	+	.	transcript_	MSTRG.7330.1	gene_id	MSTRG.7330;
12	StringTie	exon	11163787	11163870	.	+	.	transcript_	MSTRG.7330.1	gene_id	MSTRG.7330;
12	StringTie	transcrip	12009144	12010756	.	+	.	transcript_	MSTRG.7354.1	gene_id	MSTRG.7354;
12	StringTie	exon	12009144	12009220	.	+	.	transcript_	MSTRG.7354.1	gene_id	MSTRG.7354;
12	StringTie	exon	12010405	12010756	.	+	.	transcript_	MSTRG.7354.1	gene_id	MSTRG.7354;
12	StringTie	transcrip	14089784	14091173	.	+	.	transcript_	MSTRG.7381.1	gene_id	MSTRG.7381;
12	StringTie	exon	14089784	14090224	.	+	.	transcript_	MSTRG.7381.1	gene_id	MSTRG.7381;
12	StringTie	exon	14090399	14090581	.	+	.	transcript_	MSTRG.7381.1	gene_id	MSTRG.7381;

12	StringTie	exon	14090983	14091173	.	+	.	transcript_	MSTRG.7381.1	gene_id	MSTRG.7381;
12	StringTie	transcrip	15447583	15454117	.	+	.	transcript_	MSTRG.7442.1	gene_id	MSTRG.7442;
12	StringTie	exon	15447583	15447645	.	+	.	transcript_	MSTRG.7442.1	gene_id	MSTRG.7442;
12	StringTie	exon	15449086	15454117	.	+	.	transcript_	MSTRG.7442.1	gene_id	MSTRG.7442;
12	StringTie	transcrip	15448598	15451921	.	+	.	transcript_	MSTRG.7442.2	gene_id	MSTRG.7442;
12	StringTie	exon	15448598	15449353	.	+	.	transcript_	MSTRG.7442.2	gene_id	MSTRG.7442;
12	StringTie	exon	15449498	15451921	.	+	.	transcript_	MSTRG.7442.2	gene_id	MSTRG.7442;
12	StringTie	transcrip	18254959	18265712	.	+	.	transcript_	MSTRG.7490.1	gene_id	MSTRG.7490;
12	StringTie	exon	18254959	18254984	.	+	.	transcript_	MSTRG.7490.1	gene_id	MSTRG.7490;
12	StringTie	exon	18262475	18265712	.	+	.	transcript_	MSTRG.7490.1	gene_id	MSTRG.7490;
12	StringTie	transcrip	19040404	19046983	.	+	.	transcript_	MSTRG.7531.1	gene_id	MSTRG.7531;
12	StringTie	exon	19040404	19040485	.	+	.	transcript_	MSTRG.7531.1	gene_id	MSTRG.7531;
12	StringTie	exon	19043629	19043971	.	+	.	transcript_	MSTRG.7531.1	gene_id	MSTRG.7531;
12	StringTie	exon	19044720	19044829	.	+	.	transcript_	MSTRG.7531.1	gene_id	MSTRG.7531;
12	StringTie	exon	19046739	19046983	.	+	.	transcript_	MSTRG.7531.1	gene_id	MSTRG.7531;
12	StringTie	transcrip	19281434	19281808	.	+	.	transcript_	MSTRG.7545.4	gene_id	MSTRG.7545;
12	StringTie	exon	19281434	19281543	.	+	.	transcript_	MSTRG.7545.4	gene_id	MSTRG.7545;
12	StringTie	exon	19281598	19281651	.	+	.	transcript_	MSTRG.7545.4	gene_id	MSTRG.7545;
12	StringTie	exon	19281706	19281808	.	+	.	transcript_	MSTRG.7545.4	gene_id	MSTRG.7545;
12	StringTie	transcrip	19281434	19281808	.	+	.	transcript_	MSTRG.7545.3	gene_id	MSTRG.7545;
12	StringTie	exon	19281434	19281579	.	+	.	transcript_	MSTRG.7545.3	gene_id	MSTRG.7545;
12	StringTie	exon	19281706	19281808	.	+	.	transcript_	MSTRG.7545.3	gene_id	MSTRG.7545;
12	StringTie	transcrip	19281434	19281808	.	+	.	transcript_	MSTRG.7545.2	gene_id	MSTRG.7545;
12	StringTie	exon	19281434	19281651	.	+	.	transcript_	MSTRG.7545.2	gene_id	MSTRG.7545;
12	StringTie	exon	19281706	19281808	.	+	.	transcript_	MSTRG.7545.2	gene_id	MSTRG.7545;
12	StringTie	transcrip	19281434	19281810	.	+	.	transcript_	MSTRG.7545.1	gene_id	MSTRG.7545;
12	StringTie	exon	19281434	19281597	.	+	.	transcript_	MSTRG.7545.1	gene_id	MSTRG.7545;
12	StringTie	exon	19281652	19281810	.	+	.	transcript_	MSTRG.7545.1	gene_id	MSTRG.7545;
12	StringTie	transcrip	19281437	19281805	.	+	.	transcript_	MSTRG.7545.5	gene_id	MSTRG.7545;
12	StringTie	exon	19281437	19281543	.	+	.	transcript_	MSTRG.7545.5	gene_id	MSTRG.7545;
12	StringTie	exon	19281652	19281805	.	+	.	transcript_	MSTRG.7545.5	gene_id	MSTRG.7545;
12	StringTie	transcrip	19585735	19593103	.	+	.	transcript_	MSTRG.7547.1	gene_id	MSTRG.7547;
12	StringTie	exon	19585735	19585838	.	+	.	transcript_	MSTRG.7547.1	gene_id	MSTRG.7547;
12	StringTie	exon	19592985	19593103	.	+	.	transcript_	MSTRG.7547.1	gene_id	MSTRG.7547;
12	StringTie	transcrip	20005318	20020801	.	+	.	transcript_	MSTRG.7577.1	gene_id	MSTRG.7577;

12	StringTie	exon	20005318	20005428	.	+	.	transcript_	MSTRG.7577.1	gene_id	MSTRG.7577;
12	StringTie	exon	20020691	20020801	.	+	.	transcript_	MSTRG.7577.1	gene_id	MSTRG.7577;
12	StringTie	transcrip	20499643	20502100	.	+	.	transcript_	MSTRG.7604.1	gene_id	MSTRG.7604;
12	StringTie	exon	20499643	20499717	.	+	.	transcript_	MSTRG.7604.1	gene_id	MSTRG.7604;
12	StringTie	exon	20499841	20500022	.	+	.	transcript_	MSTRG.7604.1	gene_id	MSTRG.7604;
12	StringTie	exon	20501403	20502100	.	+	.	transcript_	MSTRG.7604.1	gene_id	MSTRG.7604;
12	StringTie	transcrip	24292925	24294782	.	+	.	transcript_	MSTRG.7739.1	gene_id	MSTRG.7739;
12	StringTie	exon	24292925	24293168	.	+	.	transcript_	MSTRG.7739.1	gene_id	MSTRG.7739;
12	StringTie	exon	24294498	24294782	.	+	.	transcript_	MSTRG.7739.1	gene_id	MSTRG.7739;
12	StringTie	transcrip	24797739	24804764	.	+	.	transcript_	MSTRG.7745.1	gene_id	MSTRG.7745;
12	StringTie	exon	24797739	24797794	.	+	.	transcript_	MSTRG.7745.1	gene_id	MSTRG.7745;
12	StringTie	exon	24800373	24800499	.	+	.	transcript_	MSTRG.7745.1	gene_id	MSTRG.7745;
12	StringTie	exon	24804167	24804226	.	+	.	transcript_	MSTRG.7745.1	gene_id	MSTRG.7745;
12	StringTie	exon	24804653	24804764	.	+	.	transcript_	MSTRG.7745.1	gene_id	MSTRG.7745;
12	StringTie	transcrip	24800641	24805340	.	+	.	transcript_	MSTRG.7745.2	gene_id	MSTRG.7745;
12	StringTie	exon	24800641	24800920	.	+	.	transcript_	MSTRG.7745.2	gene_id	MSTRG.7745;
12	StringTie	exon	24804167	24804226	.	+	.	transcript_	MSTRG.7745.2	gene_id	MSTRG.7745;
12	StringTie	exon	24804653	24804718	.	+	.	transcript_	MSTRG.7745.2	gene_id	MSTRG.7745;
12	StringTie	exon	24805245	24805340	.	+	.	transcript_	MSTRG.7745.2	gene_id	MSTRG.7745;
12	StringTie	transcrip	26036268	26044566	.	+	.	transcript_	MSTRG.7789.1	gene_id	MSTRG.7789;
12	StringTie	exon	26036268	26036357	.	+	.	transcript_	MSTRG.7789.1	gene_id	MSTRG.7789;
12	StringTie	exon	26044333	26044566	.	+	.	transcript_	MSTRG.7789.1	gene_id	MSTRG.7789;
12	StringTie	transcrip	26379257	26379608	.	+	.	transcript_	MSTRG.7817.1	gene_id	MSTRG.7817;
12	StringTie	exon	26379257	26379411	.	+	.	transcript_	MSTRG.7817.1	gene_id	MSTRG.7817;
12	StringTie	exon	26379538	26379608	.	+	.	transcript_	MSTRG.7817.1	gene_id	MSTRG.7817;
12	StringTie	transcrip	26380476	26381764	.	+	.	transcript_	MSTRG.7818.1	gene_id	MSTRG.7818;
12	StringTie	exon	26380476	26380494	.	+	.	transcript_	MSTRG.7818.1	gene_id	MSTRG.7818;
12	StringTie	exon	26380515	26380670	.	+	.	transcript_	MSTRG.7818.1	gene_id	MSTRG.7818;
12	StringTie	exon	26380881	26380895	.	+	.	transcript_	MSTRG.7818.1	gene_id	MSTRG.7818;
12	StringTie	exon	26381654	26381764	.	+	.	transcript_	MSTRG.7818.1	gene_id	MSTRG.7818;
12	StringTie	transcrip	26381321	26381774	.	+	.	transcript_	MSTRG.7818.2	gene_id	MSTRG.7818;
12	StringTie	exon	26381321	26381431	.	+	.	transcript_	MSTRG.7818.2	gene_id	MSTRG.7818;
12	StringTie	exon	26381664	26381774	.	+	.	transcript_	MSTRG.7818.2	gene_id	MSTRG.7818;
12	StringTie	transcrip	27120590	27123790	.	+	.	transcript_	MSTRG.7843.1	gene_id	MSTRG.7843;
12	StringTie	exon	27120590	27120804	.	+	.	transcript_	MSTRG.7843.1	gene_id	MSTRG.7843;

12	StringTie	exon	27123490	27123790	.	+	.	transcript_	MSTRG.7843.1	gene_id	MSTRG.7843;
12	StringTie	transcrip	33212478	33214731	.	+	.	transcript_	MSTRG.7918.2	gene_id	MSTRG.7918;
12	StringTie	exon	33212478	33212828	.	+	.	transcript_	MSTRG.7918.2	gene_id	MSTRG.7918;
12	StringTie	exon	33214082	33214277	.	+	.	transcript_	MSTRG.7918.2	gene_id	MSTRG.7918;
12	StringTie	exon	33214584	33214731	.	+	.	transcript_	MSTRG.7918.2	gene_id	MSTRG.7918;
12	StringTie	transcrip	33212478	33214731	.	+	.	transcript_	MSTRG.7918.1	gene_id	MSTRG.7918;
12	StringTie	exon	33212478	33214277	.	+	.	transcript_	MSTRG.7918.1	gene_id	MSTRG.7918;
12	StringTie	exon	33214584	33214731	.	+	.	transcript_	MSTRG.7918.1	gene_id	MSTRG.7918;
12	StringTie	transcrip	33212479	33214725	.	+	.	transcript_	MSTRG.7918.3	gene_id	MSTRG.7918;
12	StringTie	exon	33212479	33212854	.	+	.	transcript_	MSTRG.7918.3	gene_id	MSTRG.7918;
12	StringTie	exon	33214082	33214277	.	+	.	transcript_	MSTRG.7918.3	gene_id	MSTRG.7918;
12	StringTie	exon	33214584	33214725	.	+	.	transcript_	MSTRG.7918.3	gene_id	MSTRG.7918;
12	StringTie	transcrip	34285370	34286966	.	+	.	transcript_	MSTRG.7947.1	gene_id	MSTRG.7947;
12	StringTie	exon	34285370	34285434	.	+	.	transcript_	MSTRG.7947.1	gene_id	MSTRG.7947;
12	StringTie	exon	34285494	34286966	.	+	.	transcript_	MSTRG.7947.1	gene_id	MSTRG.7947;
12	StringTie	transcrip	36256526	36308173	.	+	.	transcript_	MSTRG.7973.1	gene_id	MSTRG.7973;
12	StringTie	exon	36256526	36256592	.	+	.	transcript_	MSTRG.7973.1	gene_id	MSTRG.7973;
12	StringTie	exon	36257793	36257875	.	+	.	transcript_	MSTRG.7973.1	gene_id	MSTRG.7973;
12	StringTie	exon	36308045	36308173	.	+	.	transcript_	MSTRG.7973.1	gene_id	MSTRG.7973;
12	StringTie	transcrip	38825046	38833501	.	+	.	transcript_	MSTRG.8034.1	gene_id	MSTRG.8034;
12	StringTie	exon	38825046	38825303	.	+	.	transcript_	MSTRG.8034.1	gene_id	MSTRG.8034;
12	StringTie	exon	38832089	38833501	.	+	.	transcript_	MSTRG.8034.1	gene_id	MSTRG.8034;
12	StringTie	transcrip	39699364	39705587	.	+	.	transcript_	MSTRG.8066.1	gene_id	MSTRG.8066;
12	StringTie	exon	39699364	39700032	.	+	.	transcript_	MSTRG.8066.1	gene_id	MSTRG.8066;
12	StringTie	exon	39700344	39705587	.	+	.	transcript_	MSTRG.8066.1	gene_id	MSTRG.8066;
12	StringTie	transcrip	42677070	42682234	.	+	.	transcript_	MSTRG.8108.1	gene_id	MSTRG.8108;
12	StringTie	exon	42677070	42677141	.	+	.	transcript_	MSTRG.8108.1	gene_id	MSTRG.8108;
12	StringTie	exon	42677558	42677676	.	+	.	transcript_	MSTRG.8108.1	gene_id	MSTRG.8108;
12	StringTie	exon	42681357	42682234	.	+	.	transcript_	MSTRG.8108.1	gene_id	MSTRG.8108;
12	StringTie	transcrip	42980243	42982859	.	+	.	transcript_	MSTRG.8132.5	gene_id	MSTRG.8132;
12	StringTie	exon	42980243	42980617	.	+	.	transcript_	MSTRG.8132.5	gene_id	MSTRG.8132;
12	StringTie	exon	42982413	42982859	.	+	.	transcript_	MSTRG.8132.5	gene_id	MSTRG.8132;
12	StringTie	transcrip	45703286	45768906	.	+	.	transcript_	MSTRG.8231.1	gene_id	MSTRG.8231;
12	StringTie	exon	45703286	45704016	.	+	.	transcript_	MSTRG.8231.1	gene_id	MSTRG.8231;
12	StringTie	exon	45768802	45768906	.	+	.	transcript_	MSTRG.8231.1	gene_id	MSTRG.8231;

12	StringTie	transcrip	46289884	46298881	.	+	.	transcript_	MSTRG.8265.1	gene_id	MSTRG.8265;
12	StringTie	exon	46289884	46290275	.	+	.	transcript_	MSTRG.8265.1	gene_id	MSTRG.8265;
12	StringTie	exon	46290637	46290744	.	+	.	transcript_	MSTRG.8265.1	gene_id	MSTRG.8265;
12	StringTie	exon	46294301	46294524	.	+	.	transcript_	MSTRG.8265.1	gene_id	MSTRG.8265;
12	StringTie	exon	46298788	46298881	.	+	.	transcript_	MSTRG.8265.1	gene_id	MSTRG.8265;
12	StringTie	transcrip	46643270	46648045	.	+	.	transcript_	MSTRG.8277.1	gene_id	MSTRG.8277;
12	StringTie	exon	46643270	46643418	.	+	.	transcript_	MSTRG.8277.1	gene_id	MSTRG.8277;
12	StringTie	exon	46647290	46648045	.	+	.	transcript_	MSTRG.8277.1	gene_id	MSTRG.8277;
12	StringTie	transcrip	48164442	48255350	.	+	.	transcript_	MSTRG.8329.1	gene_id	MSTRG.8329;
12	StringTie	exon	48164442	48165258	.	+	.	transcript_	MSTRG.8329.1	gene_id	MSTRG.8329;
12	StringTie	exon	48255257	48255350	.	+	.	transcript_	MSTRG.8329.1	gene_id	MSTRG.8329;
12	StringTie	transcrip	48199505	48204983	.	+	.	transcript_	MSTRG.8332.1	gene_id	MSTRG.8332;
12	StringTie	exon	48199505	48199562	.	+	.	transcript_	MSTRG.8332.1	gene_id	MSTRG.8332;
12	StringTie	exon	48202091	48202147	.	+	.	transcript_	MSTRG.8332.1	gene_id	MSTRG.8332;
12	StringTie	exon	48204629	48204983	.	+	.	transcript_	MSTRG.8332.1	gene_id	MSTRG.8332;
12	StringTie	transcrip	49780951	49781493	.	+	.	transcript_	MSTRG.8362.1	gene_id	MSTRG.8362;
12	StringTie	exon	49780951	49781060	.	+	.	transcript_	MSTRG.8362.1	gene_id	MSTRG.8362;
12	StringTie	exon	49781399	49781493	.	+	.	transcript_	MSTRG.8362.1	gene_id	MSTRG.8362;
12	StringTie	transcrip	49782873	49783715	.	+	.	transcript_	MSTRG.8363.1	gene_id	MSTRG.8363;
12	StringTie	exon	49782873	49782981	.	+	.	transcript_	MSTRG.8363.1	gene_id	MSTRG.8363;
12	StringTie	exon	49783606	49783715	.	+	.	transcript_	MSTRG.8363.1	gene_id	MSTRG.8363;
12	StringTie	transcrip	49783773	49784502	.	+	.	transcript_	MSTRG.8364.1	gene_id	MSTRG.8364;
12	StringTie	exon	49783773	49783857	.	+	.	transcript_	MSTRG.8364.1	gene_id	MSTRG.8364;
12	StringTie	exon	49784359	49784502	.	+	.	transcript_	MSTRG.8364.1	gene_id	MSTRG.8364;
12	StringTie	transcrip	49956819	49960464	.	+	.	transcript_	MSTRG.8366.1	gene_id	MSTRG.8366;
12	StringTie	exon	49956819	49956889	.	+	.	transcript_	MSTRG.8366.1	gene_id	MSTRG.8366;
12	StringTie	exon	49960083	49960464	.	+	.	transcript_	MSTRG.8366.1	gene_id	MSTRG.8366;
12	StringTie	transcrip	51985733	51996435	.	+	.	transcript_	MSTRG.8453.1	gene_id	MSTRG.8453;
12	StringTie	exon	51985733	51986992	.	+	.	transcript_	MSTRG.8453.1	gene_id	MSTRG.8453;
12	StringTie	exon	51990164	51990861	.	+	.	transcript_	MSTRG.8453.1	gene_id	MSTRG.8453;
12	StringTie	exon	51991141	51991413	.	+	.	transcript_	MSTRG.8453.1	gene_id	MSTRG.8453;
12	StringTie	exon	51995949	51996435	.	+	.	transcript_	MSTRG.8453.1	gene_id	MSTRG.8453;
12	StringTie	transcrip	51986666	51996433	.	+	.	transcript_	MSTRG.8453.2	gene_id	MSTRG.8453;
12	StringTie	exon	51986666	51986992	.	+	.	transcript_	MSTRG.8453.2	gene_id	MSTRG.8453;
12	StringTie	exon	51990164	51990275	.	+	.	transcript_	MSTRG.8453.2	gene_id	MSTRG.8453;

12	StringTie	exon	51991141	51991413	.	+	.	transcript_	MSTRG.8453.2	gene_id	MSTRG.8453;
12	StringTie	exon	51995949	51996433	.	+	.	transcript_	MSTRG.8453.2	gene_id	MSTRG.8453;
12	StringTie	transcrip	51986666	51996446	.	+	.	transcript_	MSTRG.8453.3	gene_id	MSTRG.8453;
12	StringTie	exon	51986666	51986992	.	+	.	transcript_	MSTRG.8453.3	gene_id	MSTRG.8453;
12	StringTie	exon	51991141	51991413	.	+	.	transcript_	MSTRG.8453.3	gene_id	MSTRG.8453;
12	StringTie	exon	51995949	51996446	.	+	.	transcript_	MSTRG.8453.3	gene_id	MSTRG.8453;
12	StringTie	transcrip	51986691	51996415	.	+	.	transcript_	MSTRG.8453.5	gene_id	MSTRG.8453;
12	StringTie	exon	51986691	51986992	.	+	.	transcript_	MSTRG.8453.5	gene_id	MSTRG.8453;
12	StringTie	exon	51989526	51989892	.	+	.	transcript_	MSTRG.8453.5	gene_id	MSTRG.8453;
12	StringTie	exon	51990164	51990282	.	+	.	transcript_	MSTRG.8453.5	gene_id	MSTRG.8453;
12	StringTie	exon	51991141	51991413	.	+	.	transcript_	MSTRG.8453.5	gene_id	MSTRG.8453;
12	StringTie	exon	51995949	51996415	.	+	.	transcript_	MSTRG.8453.5	gene_id	MSTRG.8453;
12	StringTie	transcrip	51986691	51996415	.	+	.	transcript_	MSTRG.8453.4	gene_id	MSTRG.8453;
12	StringTie	exon	51986691	51986992	.	+	.	transcript_	MSTRG.8453.4	gene_id	MSTRG.8453;
12	StringTie	exon	51995949	51996415	.	+	.	transcript_	MSTRG.8453.4	gene_id	MSTRG.8453;
12	StringTie	transcrip	51987748	51996446	.	+	.	transcript_	MSTRG.8453.6	gene_id	MSTRG.8453;
12	StringTie	exon	51987748	51989892	.	+	.	transcript_	MSTRG.8453.6	gene_id	MSTRG.8453;
12	StringTie	exon	51990164	51990861	.	+	.	transcript_	MSTRG.8453.6	gene_id	MSTRG.8453;
12	StringTie	exon	51991141	51991413	.	+	.	transcript_	MSTRG.8453.6	gene_id	MSTRG.8453;
12	StringTie	exon	51995949	51996446	.	+	.	transcript_	MSTRG.8453.6	gene_id	MSTRG.8453;
12	StringTie	transcrip	52596338	52596603	.	+	.	transcript_	MSTRG.8494.1	gene_id	MSTRG.8494;
12	StringTie	exon	52596338	52596448	.	+	.	transcript_	MSTRG.8494.1	gene_id	MSTRG.8494;
12	StringTie	exon	52596493	52596603	.	+	.	transcript_	MSTRG.8494.1	gene_id	MSTRG.8494;
12	StringTie	transcrip	53166398	53168817	.	+	.	transcript_	MSTRG.8607.2	gene_id	MSTRG.8607;
12	StringTie	exon	53166398	53166491	.	+	.	transcript_	MSTRG.8607.2	gene_id	MSTRG.8607;
12	StringTie	exon	53168645	53168817	.	+	.	transcript_	MSTRG.8607.2	gene_id	MSTRG.8607;
12	StringTie	transcrip	53166398	53176661	.	+	.	transcript_	MSTRG.8607.1	gene_id	MSTRG.8607;
12	StringTie	exon	53166398	53166491	.	+	.	transcript_	MSTRG.8607.1	gene_id	MSTRG.8607;
12	StringTie	exon	53170426	53170460	.	+	.	transcript_	MSTRG.8607.1	gene_id	MSTRG.8607;
12	StringTie	exon	53176568	53176661	.	+	.	transcript_	MSTRG.8607.1	gene_id	MSTRG.8607;
12	StringTie	transcrip	55052095	55192166	.	+	.	transcript_	MSTRG.8729.1	gene_id	MSTRG.8729;
12	StringTie	exon	55052095	55052187	.	+	.	transcript_	MSTRG.8729.1	gene_id	MSTRG.8729;
12	StringTie	exon	55138244	55138264	.	+	.	transcript_	MSTRG.8729.1	gene_id	MSTRG.8729;
12	StringTie	exon	55192069	55192166	.	+	.	transcript_	MSTRG.8729.1	gene_id	MSTRG.8729;
12	StringTie	transcrip	55052098	55192166	.	+	.	transcript_	MSTRG.8729.2	gene_id	MSTRG.8729;

12	StringTie	exon	55052098	55052208	.	+	.	transcript_	MSTRG.8729.2	gene_id	MSTRG.8729;
12	StringTie	exon	55192069	55192166	.	+	.	transcript_	MSTRG.8729.2	gene_id	MSTRG.8729;
12	StringTie	transcrip	55065039	55262678	.	+	.	transcript_	MSTRG.8729.3	gene_id	MSTRG.8729;
12	StringTie	exon	55065039	55065130	.	+	.	transcript_	MSTRG.8729.3	gene_id	MSTRG.8729;
12	StringTie	exon	55235188	55235400	.	+	.	transcript_	MSTRG.8729.3	gene_id	MSTRG.8729;
12	StringTie	exon	55262605	55262678	.	+	.	transcript_	MSTRG.8729.3	gene_id	MSTRG.8729;
12	StringTie	transcrip	55138137	55236616	.	+	.	transcript_	MSTRG.8729.4	gene_id	MSTRG.8729;
12	StringTie	exon	55138137	55138243	.	+	.	transcript_	MSTRG.8729.4	gene_id	MSTRG.8729;
12	StringTie	exon	55228469	55228489	.	+	.	transcript_	MSTRG.8729.4	gene_id	MSTRG.8729;
12	StringTie	exon	55236506	55236616	.	+	.	transcript_	MSTRG.8729.4	gene_id	MSTRG.8729;
12	StringTie	transcrip	55146485	55236614	.	+	.	transcript_	MSTRG.8729.7	gene_id	MSTRG.8729;
12	StringTie	exon	55146485	55146523	.	+	.	transcript_	MSTRG.8729.7	gene_id	MSTRG.8729;
12	StringTie	exon	55200140	55200235	.	+	.	transcript_	MSTRG.8729.7	gene_id	MSTRG.8729;
12	StringTie	exon	55201141	55201243	.	+	.	transcript_	MSTRG.8729.7	gene_id	MSTRG.8729;
12	StringTie	exon	55236506	55236614	.	+	.	transcript_	MSTRG.8729.7	gene_id	MSTRG.8729;
12	StringTie	transcrip	55146501	55263349	.	+	.	transcript_	MSTRG.8729.8	gene_id	MSTRG.8729;
12	StringTie	exon	55146501	55146523	.	+	.	transcript_	MSTRG.8729.8	gene_id	MSTRG.8729;
12	StringTie	exon	55198894	55199052	.	+	.	transcript_	MSTRG.8729.8	gene_id	MSTRG.8729;
12	StringTie	exon	55262488	55262638	.	+	.	transcript_	MSTRG.8729.8	gene_id	MSTRG.8729;
12	StringTie	exon	55263275	55263349	.	+	.	transcript_	MSTRG.8729.8	gene_id	MSTRG.8729;
12	StringTie	transcrip	55191916	55230308	.	+	.	transcript_	MSTRG.8729.9	gene_id	MSTRG.8729;
12	StringTie	exon	55191916	55192026	.	+	.	transcript_	MSTRG.8729.9	gene_id	MSTRG.8729;
12	StringTie	exon	55228448	55228544	.	+	.	transcript_	MSTRG.8729.9	gene_id	MSTRG.8729;
12	StringTie	exon	55228635	55228737	.	+	.	transcript_	MSTRG.8729.9	gene_id	MSTRG.8729;
12	StringTie	exon	55229611	55229697	.	+	.	transcript_	MSTRG.8729.9	gene_id	MSTRG.8729;
12	StringTie	exon	55230205	55230308	.	+	.	transcript_	MSTRG.8729.9	gene_id	MSTRG.8729;
12	StringTie	transcrip	55191945	55256710	.	+	.	transcript_	MSTRG.8729.1(	gene_id	MSTRG.8729;
12	StringTie	exon	55191945	55192068	.	+	.	transcript_	MSTRG.8729.1(	gene_id	MSTRG.8729;
12	StringTie	exon	55256609	55256710	.	+	.	transcript_	MSTRG.8729.1(	gene_id	MSTRG.8729;
12	StringTie	transcrip	55193909	55229697	.	+	.	transcript_	MSTRG.8729.1]	gene_id	MSTRG.8729;
12	StringTie	exon	55193909	55194098	.	+	.	transcript_	MSTRG.8729.1]	gene_id	MSTRG.8729;
12	StringTie	exon	55194748	55194841	.	+	.	transcript_	MSTRG.8729.1]	gene_id	MSTRG.8729;
12	StringTie	exon	55229587	55229697	.	+	.	transcript_	MSTRG.8729.1]	gene_id	MSTRG.8729;
12	StringTie	transcrip	55194111	55230308	.	+	.	transcript_	MSTRG.8729.1:	gene_id	MSTRG.8729;
12	StringTie	exon	55194111	55194841	.	+	.	transcript_	MSTRG.8729.1:	gene_id	MSTRG.8729;

12	StringTie	exon	55229503	55229697	.	+	.	transcript_	MSTRG.8729.1	gene_id	MSTRG.8729;
12	StringTie	exon	55230205	55230308	.	+	.	transcript_	MSTRG.8729.1	gene_id	MSTRG.8729;
12	StringTie	transcrip	55198342	55262682	.	+	.	transcript_	MSTRG.8729.1	gene_id	MSTRG.8729;
12	StringTie	exon	55198342	55198956	.	+	.	transcript_	MSTRG.8729.1	gene_id	MSTRG.8729;
12	StringTie	exon	55235188	55235400	.	+	.	transcript_	MSTRG.8729.1	gene_id	MSTRG.8729;
12	StringTie	exon	55262605	55262682	.	+	.	transcript_	MSTRG.8729.1	gene_id	MSTRG.8729;
12	StringTie	transcrip	55198857	55234508	.	+	.	transcript_	MSTRG.8729.1	gene_id	MSTRG.8729;
12	StringTie	exon	55198857	55198956	.	+	.	transcript_	MSTRG.8729.1	gene_id	MSTRG.8729;
12	StringTie	exon	55234398	55234508	.	+	.	transcript_	MSTRG.8729.1	gene_id	MSTRG.8729;
12	StringTie	transcrip	55228075	55256710	.	+	.	transcript_	MSTRG.8729.1	gene_id	MSTRG.8729;
12	StringTie	exon	55228075	55228092	.	+	.	transcript_	MSTRG.8729.1	gene_id	MSTRG.8729;
12	StringTie	exon	55228364	55228489	.	+	.	transcript_	MSTRG.8729.1	gene_id	MSTRG.8729;
12	StringTie	exon	55256609	55256710	.	+	.	transcript_	MSTRG.8729.1	gene_id	MSTRG.8729;
12	StringTie	transcrip	55229479	55258101	.	+	.	transcript_	MSTRG.8729.1	gene_id	MSTRG.8729;
12	StringTie	exon	55229479	55229586	.	+	.	transcript_	MSTRG.8729.1	gene_id	MSTRG.8729;
12	StringTie	exon	55257920	55258101	.	+	.	transcript_	MSTRG.8729.1	gene_id	MSTRG.8729;
12	StringTie	transcrip	55234949	55263349	.	+	.	transcript_	MSTRG.8729.1	gene_id	MSTRG.8729;
12	StringTie	exon	55234949	55235187	.	+	.	transcript_	MSTRG.8729.1	gene_id	MSTRG.8729;
12	StringTie	exon	55262392	55262638	.	+	.	transcript_	MSTRG.8729.1	gene_id	MSTRG.8729;
12	StringTie	exon	55263275	55263349	.	+	.	transcript_	MSTRG.8729.1	gene_id	MSTRG.8729;
12	StringTie	transcrip	55145734	55199250	.	+	.	transcript_	MSTRG.8729.6	gene_id	MSTRG.8729;
12	StringTie	exon	55145734	55145889	.	+	.	transcript_	MSTRG.8729.6	gene_id	MSTRG.8729;
12	StringTie	exon	55199170	55199250	.	+	.	transcript_	MSTRG.8729.6	gene_id	MSTRG.8729;
12	StringTie	transcrip	55195760	55231028	.	+	.	transcript_	MSTRG.8730.1	gene_id	MSTRG.8730;
12	StringTie	exon	55195760	55195865	.	+	.	transcript_	MSTRG.8730.1	gene_id	MSTRG.8730;
12	StringTie	exon	55230919	55231028	.	+	.	transcript_	MSTRG.8730.1	gene_id	MSTRG.8730;
12	StringTie	transcrip	59159224	59167247	.	+	.	transcript_	MSTRG.8671.1	gene_id	MSTRG.8671;
12	StringTie	exon	59159224	59159339	.	+	.	transcript_	MSTRG.8671.1	gene_id	MSTRG.8671;
12	StringTie	exon	59167163	59167247	.	+	.	transcript_	MSTRG.8671.1	gene_id	MSTRG.8671;
12	StringTie	transcrip	59977665	59979208	.	+	.	transcript_	MSTRG.8705.1	gene_id	MSTRG.8705;
12	StringTie	exon	59977665	59977817	.	+	.	transcript_	MSTRG.8705.1	gene_id	MSTRG.8705;
12	StringTie	exon	59978085	59978320	.	+	.	transcript_	MSTRG.8705.1	gene_id	MSTRG.8705;
12	StringTie	exon	59978873	59979007	.	+	.	transcript_	MSTRG.8705.1	gene_id	MSTRG.8705;
12	StringTie	exon	59979105	59979208	.	+	.	transcript_	MSTRG.8705.1	gene_id	MSTRG.8705;
12	StringTie	transcrip	60012122	60021460	.	+	.	transcript_	MSTRG.8716.1	gene_id	MSTRG.8716;

12	StringTie	exon	60012122	60012144	.	+	.	transcript_	MSTRG.8716.1	gene_id	MSTRG.8716;
12	StringTie	exon	60014265	60014396	.	+	.	transcript_	MSTRG.8716.1	gene_id	MSTRG.8716;
12	StringTie	exon	60021398	60021460	.	+	.	transcript_	MSTRG.8716.1	gene_id	MSTRG.8716;
12	StringTie	transcrip	61310605	61315252	.	+	.	transcript_	MSTRG.8772.1	gene_id	MSTRG.8772;
12	StringTie	exon	61310605	61313627	.	+	.	transcript_	MSTRG.8772.1	gene_id	MSTRG.8772;
12	StringTie	exon	61314170	61315252	.	+	.	transcript_	MSTRG.8772.1	gene_id	MSTRG.8772;
12	StringTie	transcrip	61316106	61319102	.	+	.	transcript_	MSTRG.8774.1	gene_id	MSTRG.8774;
12	StringTie	exon	61316106	61316531	.	+	.	transcript_	MSTRG.8774.1	gene_id	MSTRG.8774;
12	StringTie	exon	61317831	61318087	.	+	.	transcript_	MSTRG.8774.1	gene_id	MSTRG.8774;
12	StringTie	exon	61318454	61318646	.	+	.	transcript_	MSTRG.8774.1	gene_id	MSTRG.8774;
12	StringTie	exon	61318927	61319102	.	+	.	transcript_	MSTRG.8774.1	gene_id	MSTRG.8774;
12	StringTie	transcrip	61316124	61319117	.	+	.	transcript_	MSTRG.8774.2	gene_id	MSTRG.8774;
12	StringTie	exon	61316124	61316531	.	+	.	transcript_	MSTRG.8774.2	gene_id	MSTRG.8774;
12	StringTie	exon	61317831	61318087	.	+	.	transcript_	MSTRG.8774.2	gene_id	MSTRG.8774;
12	StringTie	exon	61318454	61319117	.	+	.	transcript_	MSTRG.8774.2	gene_id	MSTRG.8774;
12	StringTie	transcrip	61316140	61319117	.	+	.	transcript_	MSTRG.8774.3	gene_id	MSTRG.8774;
12	StringTie	exon	61316140	61316531	.	+	.	transcript_	MSTRG.8774.3	gene_id	MSTRG.8774;
12	StringTie	exon	61317831	61318087	.	+	.	transcript_	MSTRG.8774.3	gene_id	MSTRG.8774;
12	StringTie	exon	61318454	61318646	.	+	.	transcript_	MSTRG.8774.3	gene_id	MSTRG.8774;
12	StringTie	exon	61318949	61319117	.	+	.	transcript_	MSTRG.8774.3	gene_id	MSTRG.8774;
12	StringTie	transcrip	790434	791809	.	-	.	transcript_	MSTRG.7033.1	gene_id	MSTRG.7033;
12	StringTie	exon	790434	790911	.	-	.	transcript_	MSTRG.7033.1	gene_id	MSTRG.7033;
12	StringTie	exon	791766	791809	.	-	.	transcript_	MSTRG.7033.1	gene_id	MSTRG.7033;
12	StringTie	transcrip	1045465	1055184	.	-	.	transcript_	MSTRG.7053.1	gene_id	MSTRG.7053;
12	StringTie	exon	1045465	1046930	.	-	.	transcript_	MSTRG.7053.1	gene_id	MSTRG.7053;
12	StringTie	exon	1049914	1050649	.	-	.	transcript_	MSTRG.7053.1	gene_id	MSTRG.7053;
12	StringTie	exon	1055027	1055184	.	-	.	transcript_	MSTRG.7053.1	gene_id	MSTRG.7053;
12	StringTie	transcrip	1431502	1432730	.	-	.	transcript_	MSTRG.7097.1	gene_id	MSTRG.7097;
12	StringTie	exon	1431502	1431786	.	-	.	transcript_	MSTRG.7097.1	gene_id	MSTRG.7097;
12	StringTie	exon	1432125	1432730	.	-	.	transcript_	MSTRG.7097.1	gene_id	MSTRG.7097;
12	StringTie	transcrip	4966510	4973244	.	-	.	transcript_	MSTRG.7180.1	gene_id	MSTRG.7180;
12	StringTie	exon	4966510	4968247	.	-	.	transcript_	MSTRG.7180.1	gene_id	MSTRG.7180;
12	StringTie	exon	4973205	4973244	.	-	.	transcript_	MSTRG.7180.1	gene_id	MSTRG.7180;
12	StringTie	transcrip	5436880	5463640	.	-	.	transcript_	MSTRG.7204.1	gene_id	MSTRG.7204;
12	StringTie	exon	5436880	5436906	.	-	.	transcript_	MSTRG.7204.1	gene_id	MSTRG.7204;

12	StringTie	exon	5462299	5463640	.	-	.	transcript_	MSTRG.7204.1	gene_id	MSTRG.7204;
12	StringTie	transcrip	5477874	5478189	.	-	.	transcript_	MSTRG.7206.1	gene_id	MSTRG.7206;
12	StringTie	exon	5477874	5477984	.	-	.	transcript_	MSTRG.7206.1	gene_id	MSTRG.7206;
12	StringTie	exon	5478096	5478189	.	-	.	transcript_	MSTRG.7206.1	gene_id	MSTRG.7206;
12	StringTie	transcrip	6151305	6161991	.	-	.	transcript_	MSTRG.7233.1	gene_id	MSTRG.7233;
12	StringTie	exon	6151305	6151882	.	-	.	transcript_	MSTRG.7233.1	gene_id	MSTRG.7233;
12	StringTie	exon	6157998	6158088	.	-	.	transcript_	MSTRG.7233.1	gene_id	MSTRG.7233;
12	StringTie	exon	6158238	6158422	.	-	.	transcript_	MSTRG.7233.1	gene_id	MSTRG.7233;
12	StringTie	exon	6160467	6160647	.	-	.	transcript_	MSTRG.7233.1	gene_id	MSTRG.7233;
12	StringTie	exon	6161925	6161991	.	-	.	transcript_	MSTRG.7233.1	gene_id	MSTRG.7233;
12	StringTie	transcrip	7686260	7709960	.	-	.	transcript_	MSTRG.7289.1	gene_id	MSTRG.7289;
12	StringTie	exon	7686260	7686533	.	-	.	transcript_	MSTRG.7289.1	gene_id	MSTRG.7289;
12	StringTie	exon	7709937	7709960	.	-	.	transcript_	MSTRG.7289.1	gene_id	MSTRG.7289;
12	StringTie	transcrip	13458226	13459034	.	-	.	transcript_	MSTRG.7359.1	gene_id	MSTRG.7359;
12	StringTie	exon	13458226	13458697	.	-	.	transcript_	MSTRG.7359.1	gene_id	MSTRG.7359;
12	StringTie	exon	13459009	13459034	.	-	.	transcript_	MSTRG.7359.1	gene_id	MSTRG.7359;
12	StringTie	transcrip	13678057	13680730	.	-	.	transcript_	MSTRG.7372.1	gene_id	MSTRG.7372;
12	StringTie	exon	13678057	13678220	.	-	.	transcript_	MSTRG.7372.1	gene_id	MSTRG.7372;
12	StringTie	exon	13680472	13680730	.	-	.	transcript_	MSTRG.7372.1	gene_id	MSTRG.7372;
12	StringTie	transcrip	20388804	20389272	.	-	.	transcript_	MSTRG.7597.1	gene_id	MSTRG.7597;
12	StringTie	exon	20388804	20388913	.	-	.	transcript_	MSTRG.7597.1	gene_id	MSTRG.7597;
12	StringTie	exon	20389179	20389272	.	-	.	transcript_	MSTRG.7597.1	gene_id	MSTRG.7597;
12	StringTie	transcrip	20932577	20933730	.	-	.	transcript_	MSTRG.7621.1	gene_id	MSTRG.7621;
12	StringTie	exon	20932577	20933661	.	-	.	transcript_	MSTRG.7621.1	gene_id	MSTRG.7621;
12	StringTie	exon	20933685	20933730	.	-	.	transcript_	MSTRG.7621.1	gene_id	MSTRG.7621;
12	StringTie	transcrip	21928113	21962619	.	-	.	transcript_	MSTRG.7636.1	gene_id	MSTRG.7636;
12	StringTie	exon	21928113	21928151	.	-	.	transcript_	MSTRG.7636.1	gene_id	MSTRG.7636;
12	StringTie	exon	21961818	21962619	.	-	.	transcript_	MSTRG.7636.1	gene_id	MSTRG.7636;
12	StringTie	transcrip	24167647	24192727	.	-	.	transcript_	MSTRG.7730.1	gene_id	MSTRG.7730;
12	StringTie	exon	24167647	24167770	.	-	.	transcript_	MSTRG.7730.1	gene_id	MSTRG.7730;
12	StringTie	exon	24174093	24174202	.	-	.	transcript_	MSTRG.7730.1	gene_id	MSTRG.7730;
12	StringTie	exon	24192665	24192727	.	-	.	transcript_	MSTRG.7730.1	gene_id	MSTRG.7730;
12	StringTie	transcrip	24173095	24191585	.	-	.	transcript_	MSTRG.7730.2	gene_id	MSTRG.7730;
12	StringTie	exon	24173095	24173277	.	-	.	transcript_	MSTRG.7730.2	gene_id	MSTRG.7730;
12	StringTie	exon	24174093	24174202	.	-	.	transcript_	MSTRG.7730.2	gene_id	MSTRG.7730;

12	StringTie	exon	24191450	24191585	.	-	.	transcript_	MSTRG.7730.2	gene_id	MSTRG.7730;
12	StringTie	transcrip	24173112	24192706	.	-	.	transcript_	MSTRG.7730.3	gene_id	MSTRG.7730;
12	StringTie	exon	24173112	24173277	.	-	.	transcript_	MSTRG.7730.3	gene_id	MSTRG.7730;
12	StringTie	exon	24174093	24174202	.	-	.	transcript_	MSTRG.7730.3	gene_id	MSTRG.7730;
12	StringTie	exon	24192665	24192706	.	-	.	transcript_	MSTRG.7730.3	gene_id	MSTRG.7730;
12	StringTie	transcrip	24739313	24739812	.	-	.	transcript_	MSTRG.7741.1	gene_id	MSTRG.7741;
12	StringTie	exon	24739313	24739435	.	-	.	transcript_	MSTRG.7741.1	gene_id	MSTRG.7741;
12	StringTie	exon	24739560	24739812	.	-	.	transcript_	MSTRG.7741.1	gene_id	MSTRG.7741;
12	StringTie	transcrip	26364171	26367678	.	-	.	transcript_	MSTRG.7811.1	gene_id	MSTRG.7811;
12	StringTie	exon	26364171	26365114	.	-	.	transcript_	MSTRG.7811.1	gene_id	MSTRG.7811;
12	StringTie	exon	26366912	26367678	.	-	.	transcript_	MSTRG.7811.1	gene_id	MSTRG.7811;
12	StringTie	transcrip	26606875	26622915	.	-	.	transcript_	MSTRG.7821.8	gene_id	MSTRG.7821;
12	StringTie	exon	26606875	26607221	.	-	.	transcript_	MSTRG.7821.8	gene_id	MSTRG.7821;
12	StringTie	exon	26607615	26607807	.	-	.	transcript_	MSTRG.7821.8	gene_id	MSTRG.7821;
12	StringTie	exon	26619942	26620170	.	-	.	transcript_	MSTRG.7821.8	gene_id	MSTRG.7821;
12	StringTie	exon	26622810	26622915	.	-	.	transcript_	MSTRG.7821.8	gene_id	MSTRG.7821;
12	StringTie	transcrip	27434269	27439484	.	-	.	transcript_	MSTRG.7889.1	gene_id	MSTRG.7889;
12	StringTie	exon	27434269	27434749	.	-	.	transcript_	MSTRG.7889.1	gene_id	MSTRG.7889;
12	StringTie	exon	27439438	27439484	.	-	.	transcript_	MSTRG.7889.1	gene_id	MSTRG.7889;
12	StringTie	transcrip	27434548	27440119	.	-	.	transcript_	MSTRG.7889.2	gene_id	MSTRG.7889;
12	StringTie	exon	27434548	27434749	.	-	.	transcript_	MSTRG.7889.2	gene_id	MSTRG.7889;
12	StringTie	exon	27440036	27440119	.	-	.	transcript_	MSTRG.7889.2	gene_id	MSTRG.7889;
12	StringTie	transcrip	33152580	33160874	.	-	.	transcript_	MSTRG.7921.1	gene_id	MSTRG.7921;
12	StringTie	exon	33152580	33152598	.	-	.	transcript_	MSTRG.7921.1	gene_id	MSTRG.7921;
12	StringTie	exon	33160672	33160874	.	-	.	transcript_	MSTRG.7921.1	gene_id	MSTRG.7921;
12	StringTie	transcrip	36424335	36434287	.	-	.	transcript_	MSTRG.7990.1	gene_id	MSTRG.7990;
12	StringTie	exon	36424335	36425024	.	-	.	transcript_	MSTRG.7990.1	gene_id	MSTRG.7990;
12	StringTie	exon	36426674	36426842	.	-	.	transcript_	MSTRG.7990.1	gene_id	MSTRG.7990;
12	StringTie	exon	36434253	36434287	.	-	.	transcript_	MSTRG.7990.1	gene_id	MSTRG.7990;
12	StringTie	transcrip	39769058	39773647	.	-	.	transcript_	MSTRG.8069.1	gene_id	MSTRG.8069;
12	StringTie	exon	39769058	39769220	.	-	.	transcript_	MSTRG.8069.1	gene_id	MSTRG.8069;
12	StringTie	exon	39772169	39772251	.	-	.	transcript_	MSTRG.8069.1	gene_id	MSTRG.8069;
12	StringTie	exon	39772487	39772598	.	-	.	transcript_	MSTRG.8069.1	gene_id	MSTRG.8069;
12	StringTie	exon	39772982	39773647	.	-	.	transcript_	MSTRG.8069.1	gene_id	MSTRG.8069;
12	StringTie	transcrip	40051483	40072824	.	-	.	transcript_	MSTRG.8078.1	gene_id	MSTRG.8078;

12	StringTie	exon	40051483	40051514	.	-	.	transcript_	MSTRG.8078.1	gene_id	MSTRG.8078;
12	StringTie	exon	40069575	40072824	.	-	.	transcript_	MSTRG.8078.1	gene_id	MSTRG.8078;
12	StringTie	transcrip	43319613	43321226	.	-	.	transcript_	MSTRG.8136.4	gene_id	MSTRG.8136;
12	StringTie	exon	43319613	43319931	.	-	.	transcript_	MSTRG.8136.4	gene_id	MSTRG.8136;
12	StringTie	exon	43320425	43320495	.	-	.	transcript_	MSTRG.8136.4	gene_id	MSTRG.8136;
12	StringTie	exon	43320873	43321226	.	-	.	transcript_	MSTRG.8136.4	gene_id	MSTRG.8136;
12	StringTie	transcrip	43319613	43322263	.	-	.	transcript_	MSTRG.8136.3	gene_id	MSTRG.8136;
12	StringTie	exon	43319613	43319931	.	-	.	transcript_	MSTRG.8136.3	gene_id	MSTRG.8136;
12	StringTie	exon	43320425	43320495	.	-	.	transcript_	MSTRG.8136.3	gene_id	MSTRG.8136;
12	StringTie	exon	43322136	43322263	.	-	.	transcript_	MSTRG.8136.3	gene_id	MSTRG.8136;
12	StringTie	transcrip	45998462	46015199	.	-	.	transcript_	MSTRG.8240.1	gene_id	MSTRG.8240;
12	StringTie	exon	45998462	45998765	.	-	.	transcript_	MSTRG.8240.1	gene_id	MSTRG.8240;
12	StringTie	exon	46015158	46015199	.	-	.	transcript_	MSTRG.8240.1	gene_id	MSTRG.8240;
12	StringTie	transcrip	47030526	47031233	.	-	.	transcript_	MSTRG.8281.1	gene_id	MSTRG.8281;
12	StringTie	exon	47030526	47030879	.	-	.	transcript_	MSTRG.8281.1	gene_id	MSTRG.8281;
12	StringTie	exon	47031141	47031233	.	-	.	transcript_	MSTRG.8281.1	gene_id	MSTRG.8281;
12	StringTie	transcrip	47554834	47570883	.	-	.	transcript_	MSTRG.8286.1	gene_id	MSTRG.8286;
12	StringTie	exon	47554834	47555128	.	-	.	transcript_	MSTRG.8286.1	gene_id	MSTRG.8286;
12	StringTie	exon	47555987	47556141	.	-	.	transcript_	MSTRG.8286.1	gene_id	MSTRG.8286;
12	StringTie	exon	47559981	47562255	.	-	.	transcript_	MSTRG.8286.1	gene_id	MSTRG.8286;
12	StringTie	exon	47563384	47563852	.	-	.	transcript_	MSTRG.8286.1	gene_id	MSTRG.8286;
12	StringTie	exon	47570750	47570883	.	-	.	transcript_	MSTRG.8286.1	gene_id	MSTRG.8286;
12	StringTie	transcrip	47559676	47562365	.	-	.	transcript_	MSTRG.8286.2	gene_id	MSTRG.8286;
12	StringTie	exon	47559676	47561420	.	-	.	transcript_	MSTRG.8286.2	gene_id	MSTRG.8286;
12	StringTie	exon	47562083	47562365	.	-	.	transcript_	MSTRG.8286.2	gene_id	MSTRG.8286;
12	StringTie	transcrip	50366377	50368577	.	-	.	transcript_	MSTRG.8379.1	gene_id	MSTRG.8379;
12	StringTie	exon	50366377	50366818	.	-	.	transcript_	MSTRG.8379.1	gene_id	MSTRG.8379;
12	StringTie	exon	50368489	50368577	.	-	.	transcript_	MSTRG.8379.1	gene_id	MSTRG.8379;
12	StringTie	transcrip	52315050	52342538	.	-	.	transcript_	MSTRG.8475.1	gene_id	MSTRG.8475;
12	StringTie	exon	52315050	52315262	.	-	.	transcript_	MSTRG.8475.1	gene_id	MSTRG.8475;
12	StringTie	exon	52318622	52318855	.	-	.	transcript_	MSTRG.8475.1	gene_id	MSTRG.8475;
12	StringTie	exon	52331935	52332213	.	-	.	transcript_	MSTRG.8475.1	gene_id	MSTRG.8475;
12	StringTie	exon	52341098	52341220	.	-	.	transcript_	MSTRG.8475.1	gene_id	MSTRG.8475;
12	StringTie	exon	52342449	52342538	.	-	.	transcript_	MSTRG.8475.1	gene_id	MSTRG.8475;
12	StringTie	transcrip	52414214	52416922	.	-	.	transcript_	MSTRG.8484.1	gene_id	MSTRG.8484;

12	StringTie	exon	52414214	52414295	.	-	.	transcript_	MSTRG.8484.1	gene_id	MSTRG.8484;
12	StringTie	exon	52416410	52416922	.	-	.	transcript_	MSTRG.8484.1	gene_id	MSTRG.8484;
12	StringTie	transcrip	52420244	52424021	.	-	.	transcript_	MSTRG.8486.2	gene_id	MSTRG.8486;
12	StringTie	exon	52420244	52420370	.	-	.	transcript_	MSTRG.8486.2	gene_id	MSTRG.8486;
12	StringTie	exon	52420881	52421068	.	-	.	transcript_	MSTRG.8486.2	gene_id	MSTRG.8486;
12	StringTie	exon	52421536	52421804	.	-	.	transcript_	MSTRG.8486.2	gene_id	MSTRG.8486;
12	StringTie	exon	52423784	52424021	.	-	.	transcript_	MSTRG.8486.2	gene_id	MSTRG.8486;
12	StringTie	transcrip	52420244	52424405	.	-	.	transcript_	MSTRG.8486.1	gene_id	MSTRG.8486;
12	StringTie	exon	52420244	52420370	.	-	.	transcript_	MSTRG.8486.1	gene_id	MSTRG.8486;
12	StringTie	exon	52420881	52421068	.	-	.	transcript_	MSTRG.8486.1	gene_id	MSTRG.8486;
12	StringTie	exon	52421536	52421808	.	-	.	transcript_	MSTRG.8486.1	gene_id	MSTRG.8486;
12	StringTie	exon	52423784	52424405	.	-	.	transcript_	MSTRG.8486.1	gene_id	MSTRG.8486;
12	StringTie	transcrip	52434621	52435463	.	-	.	transcript_	MSTRG.8467.1	gene_id	MSTRG.8467;
12	StringTie	exon	52434621	52435016	.	-	.	transcript_	MSTRG.8467.1	gene_id	MSTRG.8467;
12	StringTie	exon	52435318	52435463	.	-	.	transcript_	MSTRG.8467.1	gene_id	MSTRG.8467;
12	StringTie	transcrip	52804702	52805131	.	-	.	transcript_	MSTRG.8584.1	gene_id	MSTRG.8584;
12	StringTie	exon	52804702	52804831	.	-	.	transcript_	MSTRG.8584.1	gene_id	MSTRG.8584;
12	StringTie	exon	52804916	52805131	.	-	.	transcript_	MSTRG.8584.1	gene_id	MSTRG.8584;
12	StringTie	transcrip	52804723	52805131	.	-	.	transcript_	MSTRG.8584.3	gene_id	MSTRG.8584;
12	StringTie	exon	52804723	52804873	.	-	.	transcript_	MSTRG.8584.3	gene_id	MSTRG.8584;
12	StringTie	exon	52805021	52805131	.	-	.	transcript_	MSTRG.8584.3	gene_id	MSTRG.8584;
12	StringTie	transcrip	52804723	52805131	.	-	.	transcript_	MSTRG.8584.2	gene_id	MSTRG.8584;
12	StringTie	exon	52804723	52804831	.	-	.	transcript_	MSTRG.8584.2	gene_id	MSTRG.8584;
12	StringTie	exon	52804916	52804957	.	-	.	transcript_	MSTRG.8584.2	gene_id	MSTRG.8584;
12	StringTie	exon	52805021	52805131	.	-	.	transcript_	MSTRG.8584.2	gene_id	MSTRG.8584;
12	StringTie	transcrip	53129872	53134306	.	-	.	transcript_	MSTRG.8604.4	gene_id	MSTRG.8604;
12	StringTie	exon	53129872	53131920	.	-	.	transcript_	MSTRG.8604.4	gene_id	MSTRG.8604;
12	StringTie	exon	53134259	53134306	.	-	.	transcript_	MSTRG.8604.4	gene_id	MSTRG.8604;
12	StringTie	transcrip	53420607	53422466	.	-	.	transcript_	MSTRG.8531.1	gene_id	MSTRG.8531;
12	StringTie	exon	53420607	53421665	.	-	.	transcript_	MSTRG.8531.1	gene_id	MSTRG.8531;
12	StringTie	exon	53422397	53422466	.	-	.	transcript_	MSTRG.8531.1	gene_id	MSTRG.8531;
12	StringTie	transcrip	57933166	58011767	.	-	.	transcript_	MSTRG.8648.1	gene_id	MSTRG.8648;
12	StringTie	exon	57933166	57933183	.	-	.	transcript_	MSTRG.8648.1	gene_id	MSTRG.8648;
12	StringTie	exon	57984453	57987977	.	-	.	transcript_	MSTRG.8648.1	gene_id	MSTRG.8648;
12	StringTie	exon	57999125	57999212	.	-	.	transcript_	MSTRG.8648.1	gene_id	MSTRG.8648;

12	StringTie	exon	58011516	58011767	.	-	.	transcript_	MSTRG.8648.1	gene_id	MSTRG.8648;
12	StringTie	transcrip	57985265	57997718	.	-	.	transcript_	MSTRG.8648.2	gene_id	MSTRG.8648;
12	StringTie	exon	57985265	57987977	.	-	.	transcript_	MSTRG.8648.2	gene_id	MSTRG.8648;
12	StringTie	exon	57994440	57997718	.	-	.	transcript_	MSTRG.8648.2	gene_id	MSTRG.8648;
12	StringTie	transcrip	57985488	58011766	.	-	.	transcript_	MSTRG.8648.3	gene_id	MSTRG.8648;
12	StringTie	exon	57985488	57987977	.	-	.	transcript_	MSTRG.8648.3	gene_id	MSTRG.8648;
12	StringTie	exon	58011516	58011766	.	-	.	transcript_	MSTRG.8648.3	gene_id	MSTRG.8648;
12	StringTie	transcrip	57985584	58004399	.	-	.	transcript_	MSTRG.8648.4	gene_id	MSTRG.8648;
12	StringTie	exon	57985584	57987977	.	-	.	transcript_	MSTRG.8648.4	gene_id	MSTRG.8648;
12	StringTie	exon	57999125	57999212	.	-	.	transcript_	MSTRG.8648.4	gene_id	MSTRG.8648;
12	StringTie	exon	58004005	58004399	.	-	.	transcript_	MSTRG.8648.4	gene_id	MSTRG.8648;
12	StringTie	transcrip	57994445	58011748	.	-	.	transcript_	MSTRG.8648.5	gene_id	MSTRG.8648;
12	StringTie	exon	57994445	57994581	.	-	.	transcript_	MSTRG.8648.5	gene_id	MSTRG.8648;
12	StringTie	exon	57994878	57995096	.	-	.	transcript_	MSTRG.8648.5	gene_id	MSTRG.8648;
12	StringTie	exon	57999125	57999212	.	-	.	transcript_	MSTRG.8648.5	gene_id	MSTRG.8648;
12	StringTie	exon	58011516	58011748	.	-	.	transcript_	MSTRG.8648.5	gene_id	MSTRG.8648;
12	StringTie	transcrip	59091630	59092017	.	-	.	transcript_	MSTRG.8659.1	gene_id	MSTRG.8659;
12	StringTie	exon	59091630	59091662	.	-	.	transcript_	MSTRG.8659.1	gene_id	MSTRG.8659;
12	StringTie	exon	59091768	59092017	.	-	.	transcript_	MSTRG.8659.1	gene_id	MSTRG.8659;
12	StringTie	transcrip	59132604	59134605	.	-	.	transcript_	MSTRG.8665.2	gene_id	MSTRG.8665;
12	StringTie	exon	59132604	59132868	.	-	.	transcript_	MSTRG.8665.2	gene_id	MSTRG.8665;
12	StringTie	exon	59133101	59133181	.	-	.	transcript_	MSTRG.8665.2	gene_id	MSTRG.8665;
12	StringTie	exon	59133584	59133645	.	-	.	transcript_	MSTRG.8665.2	gene_id	MSTRG.8665;
12	StringTie	exon	59134067	59134117	.	-	.	transcript_	MSTRG.8665.2	gene_id	MSTRG.8665;
12	StringTie	exon	59134333	59134605	.	-	.	transcript_	MSTRG.8665.2	gene_id	MSTRG.8665;
12	StringTie	transcrip	59132604	59134605	.	-	.	transcript_	MSTRG.8665.1	gene_id	MSTRG.8665;
12	StringTie	exon	59132604	59132868	.	-	.	transcript_	MSTRG.8665.1	gene_id	MSTRG.8665;
12	StringTie	exon	59133101	59133204	.	-	.	transcript_	MSTRG.8665.1	gene_id	MSTRG.8665;
12	StringTie	exon	59133584	59133645	.	-	.	transcript_	MSTRG.8665.1	gene_id	MSTRG.8665;
12	StringTie	exon	59134067	59134117	.	-	.	transcript_	MSTRG.8665.1	gene_id	MSTRG.8665;
12	StringTie	exon	59134333	59134605	.	-	.	transcript_	MSTRG.8665.1	gene_id	MSTRG.8665;
12	StringTie	transcrip	60412857	60438387	.	-	.	transcript_	MSTRG.8742.1	gene_id	MSTRG.8742;
12	StringTie	exon	60412857	60412904	.	-	.	transcript_	MSTRG.8742.1	gene_id	MSTRG.8742;
12	StringTie	exon	60436960	60438387	.	-	.	transcript_	MSTRG.8742.1	gene_id	MSTRG.8742;
12	StringTie	transcrip	61326207	61332394	.	-	.	transcript_	MSTRG.8776.1	gene_id	MSTRG.8776;

12	StringTie	exon	61326207	61331314	.	-	.	transcript_	MSTRG.8776.1	gene_id	MSTRG.8776;
12	StringTie	exon	61332228	61332394	.	-	.	transcript_	MSTRG.8776.1	gene_id	MSTRG.8776;
12	StringTie	transcrip	61326337	61332375	.	-	.	transcript_	MSTRG.8776.2	gene_id	MSTRG.8776;
12	StringTie	exon	61326337	61326899	.	-	.	transcript_	MSTRG.8776.2	gene_id	MSTRG.8776;
12	StringTie	exon	61332237	61332375	.	-	.	transcript_	MSTRG.8776.2	gene_id	MSTRG.8776;
12	StringTie	transcrip	61326353	61332394	.	-	.	transcript_	MSTRG.8776.3	gene_id	MSTRG.8776;
12	StringTie	exon	61326353	61331314	.	-	.	transcript_	MSTRG.8776.3	gene_id	MSTRG.8776;
12	StringTie	exon	61332237	61332394	.	-	.	transcript_	MSTRG.8776.3	gene_id	MSTRG.8776;
12	StringTie	transcrip	61327375	61329897	.	-	.	transcript_	MSTRG.8776.4	gene_id	MSTRG.8776;
12	StringTie	exon	61327375	61327726	.	-	.	transcript_	MSTRG.8776.4	gene_id	MSTRG.8776;
12	StringTie	exon	61329661	61329897	.	-	.	transcript_	MSTRG.8776.4	gene_id	MSTRG.8776;
13	StringTie	transcrip	9844366	9884636	.	+	.	transcript_	MSTRG.8870.1	gene_id	MSTRG.8870;
13	StringTie	exon	9844366	9844612	.	+	.	transcript_	MSTRG.8870.1	gene_id	MSTRG.8870;
13	StringTie	exon	9851869	9851907	.	+	.	transcript_	MSTRG.8870.1	gene_id	MSTRG.8870;
13	StringTie	exon	9884290	9884636	.	+	.	transcript_	MSTRG.8870.1	gene_id	MSTRG.8870;
13	StringTie	transcrip	9844366	9906449	.	+	.	transcript_	MSTRG.8870.2	gene_id	MSTRG.8870;
13	StringTie	exon	9844366	9844612	.	+	.	transcript_	MSTRG.8870.2	gene_id	MSTRG.8870;
13	StringTie	exon	9851869	9851907	.	+	.	transcript_	MSTRG.8870.2	gene_id	MSTRG.8870;
13	StringTie	exon	9905238	9905304	.	+	.	transcript_	MSTRG.8870.2	gene_id	MSTRG.8870;
13	StringTie	exon	9906300	9906449	.	+	.	transcript_	MSTRG.8870.2	gene_id	MSTRG.8870;
13	StringTie	transcrip	9871287	9884636	.	+	.	transcript_	MSTRG.8870.3	gene_id	MSTRG.8870;
13	StringTie	exon	9871287	9872033	.	+	.	transcript_	MSTRG.8870.3	gene_id	MSTRG.8870;
13	StringTie	exon	9884290	9884636	.	+	.	transcript_	MSTRG.8870.3	gene_id	MSTRG.8870;
13	StringTie	transcrip	18440025	18464102	.	+	.	transcript_	MSTRG.8980.1	gene_id	MSTRG.8980;
13	StringTie	exon	18440025	18440064	.	+	.	transcript_	MSTRG.8980.1	gene_id	MSTRG.8980;
13	StringTie	exon	18462005	18464102	.	+	.	transcript_	MSTRG.8980.1	gene_id	MSTRG.8980;
13	StringTie	transcrip	18463133	18464074	.	+	.	transcript_	MSTRG.8980.2	gene_id	MSTRG.8980;
13	StringTie	exon	18463133	18463545	.	+	.	transcript_	MSTRG.8980.2	gene_id	MSTRG.8980;
13	StringTie	exon	18463587	18464074	.	+	.	transcript_	MSTRG.8980.2	gene_id	MSTRG.8980;
13	StringTie	transcrip	23090344	23092641	.	+	.	transcript_	MSTRG.9067.1	gene_id	MSTRG.9067;
13	StringTie	exon	23090344	23092113	.	+	.	transcript_	MSTRG.9067.1	gene_id	MSTRG.9067;
13	StringTie	exon	23092411	23092641	.	+	.	transcript_	MSTRG.9067.1	gene_id	MSTRG.9067;
13	StringTie	transcrip	27960044	27961986	.	+	.	transcript_	MSTRG.9196.1	gene_id	MSTRG.9196;
13	StringTie	exon	27960044	27960133	.	+	.	transcript_	MSTRG.9196.1	gene_id	MSTRG.9196;
13	StringTie	exon	27961811	27961986	.	+	.	transcript_	MSTRG.9196.1	gene_id	MSTRG.9196;

13	StringTie	transcrip	29996369	30000816	.	+	.	transcript_	MSTRG.9286.1	gene_id	MSTRG.9286;
13	StringTie	exon	29996369	29996758	.	+	.	transcript_	MSTRG.9286.1	gene_id	MSTRG.9286;
13	StringTie	exon	29997777	30000816	.	+	.	transcript_	MSTRG.9286.1	gene_id	MSTRG.9286;
13	StringTie	transcrip	30679421	30679725	.	+	.	transcript_	MSTRG.9315.1	gene_id	MSTRG.9315;
13	StringTie	exon	30679421	30679531	.	+	.	transcript_	MSTRG.9315.1	gene_id	MSTRG.9315;
13	StringTie	exon	30679616	30679725	.	+	.	transcript_	MSTRG.9315.1	gene_id	MSTRG.9315;
13	StringTie	transcrip	31951657	31957979	.	+	.	transcript_	MSTRG.9364.1	gene_id	MSTRG.9364;
13	StringTie	exon	31951657	31951678	.	+	.	transcript_	MSTRG.9364.1	gene_id	MSTRG.9364;
13	StringTie	exon	31957731	31957979	.	+	.	transcript_	MSTRG.9364.1	gene_id	MSTRG.9364;
13	StringTie	transcrip	32368390	32370365	.	+	.	transcript_	MSTRG.9382.1	gene_id	MSTRG.9382;
13	StringTie	exon	32368390	32368430	.	+	.	transcript_	MSTRG.9382.1	gene_id	MSTRG.9382;
13	StringTie	exon	32370053	32370365	.	+	.	transcript_	MSTRG.9382.1	gene_id	MSTRG.9382;
13	StringTie	transcrip	34100081	34101792	.	+	.	transcript_	MSTRG.9440.1	gene_id	MSTRG.9440;
13	StringTie	exon	34100081	34100799	.	+	.	transcript_	MSTRG.9440.1	gene_id	MSTRG.9440;
13	StringTie	exon	34101683	34101792	.	+	.	transcript_	MSTRG.9440.1	gene_id	MSTRG.9440;
13	StringTie	transcrip	37355369	37357443	.	+	.	transcript_	MSTRG.9485.1	gene_id	MSTRG.9485;
13	StringTie	exon	37355369	37355440	.	+	.	transcript_	MSTRG.9485.1	gene_id	MSTRG.9485;
13	StringTie	exon	37355575	37355673	.	+	.	transcript_	MSTRG.9485.1	gene_id	MSTRG.9485;
13	StringTie	exon	37356008	37357443	.	+	.	transcript_	MSTRG.9485.1	gene_id	MSTRG.9485;
13	StringTie	transcrip	40283029	40307104	.	+	.	transcript_	MSTRG.9569.1	gene_id	MSTRG.9569;
13	StringTie	exon	40283029	40285610	.	+	.	transcript_	MSTRG.9569.1	gene_id	MSTRG.9569;
13	StringTie	exon	40306565	40307104	.	+	.	transcript_	MSTRG.9569.1	gene_id	MSTRG.9569;
13	StringTie	transcrip	46075772	46099146	.	+	.	transcript_	MSTRG.9682.1	gene_id	MSTRG.9682;
13	StringTie	exon	46075772	46075828	.	+	.	transcript_	MSTRG.9682.1	gene_id	MSTRG.9682;
13	StringTie	exon	46083213	46083513	.	+	.	transcript_	MSTRG.9682.1	gene_id	MSTRG.9682;
13	StringTie	exon	46083588	46083648	.	+	.	transcript_	MSTRG.9682.1	gene_id	MSTRG.9682;
13	StringTie	exon	46087314	46087561	.	+	.	transcript_	MSTRG.9682.1	gene_id	MSTRG.9682;
13	StringTie	exon	46098917	46099146	.	+	.	transcript_	MSTRG.9682.1	gene_id	MSTRG.9682;
13	StringTie	transcrip	46087412	46090458	.	+	.	transcript_	MSTRG.9682.2	gene_id	MSTRG.9682;
13	StringTie	exon	46087412	46087561	.	+	.	transcript_	MSTRG.9682.2	gene_id	MSTRG.9682;
13	StringTie	exon	46090398	46090458	.	+	.	transcript_	MSTRG.9682.2	gene_id	MSTRG.9682;
13	StringTie	transcrip	46203909	46220914	.	+	.	transcript_	MSTRG.9683.1	gene_id	MSTRG.9683;
13	StringTie	exon	46203909	46204373	.	+	.	transcript_	MSTRG.9683.1	gene_id	MSTRG.9683;
13	StringTie	exon	46220630	46220914	.	+	.	transcript_	MSTRG.9683.1	gene_id	MSTRG.9683;
13	StringTie	transcrip	46203956	46232206	.	+	.	transcript_	MSTRG.9683.2	gene_id	MSTRG.9683;

13	StringTie	exon	46203956	46204373	.	+	.	transcript_	MSTRG.9683.2	gene_id	MSTRG.9683;
13	StringTie	exon	46231947	46232206	.	+	.	transcript_	MSTRG.9683.2	gene_id	MSTRG.9683;
13	StringTie	transcrip	46203962	46224622	.	+	.	transcript_	MSTRG.9683.4	gene_id	MSTRG.9683;
13	StringTie	exon	46203962	46204373	.	+	.	transcript_	MSTRG.9683.4	gene_id	MSTRG.9683;
13	StringTie	exon	46223478	46224622	.	+	.	transcript_	MSTRG.9683.4	gene_id	MSTRG.9683;
13	StringTie	transcrip	46203962	46403603	.	+	.	transcript_	MSTRG.9683.3	gene_id	MSTRG.9683;
13	StringTie	exon	46203962	46204373	.	+	.	transcript_	MSTRG.9683.3	gene_id	MSTRG.9683;
13	StringTie	exon	46363378	46363483	.	+	.	transcript_	MSTRG.9683.3	gene_id	MSTRG.9683;
13	StringTie	exon	46397675	46403603	.	+	.	transcript_	MSTRG.9683.3	gene_id	MSTRG.9683;
13	StringTie	transcrip	46203963	46277148	.	+	.	transcript_	MSTRG.9683.5	gene_id	MSTRG.9683;
13	StringTie	exon	46203963	46204373	.	+	.	transcript_	MSTRG.9683.5	gene_id	MSTRG.9683;
13	StringTie	exon	46275763	46277148	.	+	.	transcript_	MSTRG.9683.5	gene_id	MSTRG.9683;
13	StringTie	transcrip	46275380	46276990	.	+	.	transcript_	MSTRG.9683.6	gene_id	MSTRG.9683;
13	StringTie	exon	46275380	46275926	.	+	.	transcript_	MSTRG.9683.6	gene_id	MSTRG.9683;
13	StringTie	exon	46276625	46276990	.	+	.	transcript_	MSTRG.9683.6	gene_id	MSTRG.9683;
13	StringTie	transcrip	46314644	46315889	.	+	.	transcript_	MSTRG.9686.1	gene_id	MSTRG.9686;
13	StringTie	exon	46314644	46314698	.	+	.	transcript_	MSTRG.9686.1	gene_id	MSTRG.9686;
13	StringTie	exon	46315540	46315889	.	+	.	transcript_	MSTRG.9686.1	gene_id	MSTRG.9686;
13	StringTie	transcrip	47588197	47742843	.	+	.	transcript_	MSTRG.9698.1	gene_id	MSTRG.9698;
13	StringTie	exon	47588197	47588314	.	+	.	transcript_	MSTRG.9698.1	gene_id	MSTRG.9698;
13	StringTie	exon	47637664	47637775	.	+	.	transcript_	MSTRG.9698.1	gene_id	MSTRG.9698;
13	StringTie	exon	47741933	47742843	.	+	.	transcript_	MSTRG.9698.1	gene_id	MSTRG.9698;
13	StringTie	transcrip	52972171	53019403	.	+	.	transcript_	MSTRG.9820.1	gene_id	MSTRG.9820;
13	StringTie	exon	52972171	52972218	.	+	.	transcript_	MSTRG.9820.1	gene_id	MSTRG.9820;
13	StringTie	exon	53011726	53011920	.	+	.	transcript_	MSTRG.9820.1	gene_id	MSTRG.9820;
13	StringTie	exon	53014925	53015143	.	+	.	transcript_	MSTRG.9820.1	gene_id	MSTRG.9820;
13	StringTie	exon	53019380	53019403	.	+	.	transcript_	MSTRG.9820.1	gene_id	MSTRG.9820;
13	StringTie	transcrip	69591015	69594197	.	+	.	transcript_	MSTRG.10050.	gene_id	MSTRG.10050;
13	StringTie	exon	69591015	69591564	.	+	.	transcript_	MSTRG.10050.	gene_id	MSTRG.10050;
13	StringTie	exon	69594057	69594197	.	+	.	transcript_	MSTRG.10050.	gene_id	MSTRG.10050;
13	StringTie	transcrip	71671925	71700661	.	+	.	transcript_	MSTRG.10082.[illegible]	gene_id	MSTRG.10082;
13	StringTie	exon	71671925	71672884	.	+	.	transcript_	MSTRG.10082.[illegible]	gene_id	MSTRG.10082;
13	StringTie	exon	71698782	71698923	.	+	.	transcript_	MSTRG.10082.[illegible]	gene_id	MSTRG.10082;
13	StringTie	exon	71699311	71700661	.	+	.	transcript_	MSTRG.10082.[illegible]	gene_id	MSTRG.10082;
13	StringTie	transcrip	71671925	71700661	.	+	.	transcript_	MSTRG.10082.	gene_id	MSTRG.10082;

13	StringTie	exon	71671925	71672884	.	+	.	transcript_	MSTRG.10082.1	gene_id	MSTRG.10082;
13	StringTie	exon	71698782	71700661	.	+	.	transcript_	MSTRG.10082.1	gene_id	MSTRG.10082;
13	StringTie	transcrip	71671925	71701150	.	+	.	transcript_	MSTRG.10082.4	gene_id	MSTRG.10082;
13	StringTie	exon	71671925	71672884	.	+	.	transcript_	MSTRG.10082.4	gene_id	MSTRG.10082;
13	StringTie	exon	71679432	71679534	.	+	.	transcript_	MSTRG.10082.4	gene_id	MSTRG.10082;
13	StringTie	exon	71698782	71701150	.	+	.	transcript_	MSTRG.10082.4	gene_id	MSTRG.10082;
13	StringTie	transcrip	73256311	73259271	.	+	.	transcript_	MSTRG.10112.1	gene_id	MSTRG.10112;
13	StringTie	exon	73256311	73257563	.	+	.	transcript_	MSTRG.10112.1	gene_id	MSTRG.10112;
13	StringTie	exon	73259000	73259271	.	+	.	transcript_	MSTRG.10112.1	gene_id	MSTRG.10112;
13	StringTie	transcrip	75843222	75845503	.	+	.	transcript_	MSTRG.10154.1	gene_id	MSTRG.10154;
13	StringTie	exon	75843222	75843540	.	+	.	transcript_	MSTRG.10154.1	gene_id	MSTRG.10154;
13	StringTie	exon	75843672	75843760	.	+	.	transcript_	MSTRG.10154.1	gene_id	MSTRG.10154;
13	StringTie	exon	75844773	75845503	.	+	.	transcript_	MSTRG.10154.1	gene_id	MSTRG.10154;
13	StringTie	transcrip	87732417	87736253	.	+	.	transcript_	MSTRG.10349.1	gene_id	MSTRG.10349;
13	StringTie	exon	87732417	87732527	.	+	.	transcript_	MSTRG.10349.1	gene_id	MSTRG.10349;
13	StringTie	exon	87732990	87733113	.	+	.	transcript_	MSTRG.10349.1	gene_id	MSTRG.10349;
13	StringTie	exon	87733720	87736253	.	+	.	transcript_	MSTRG.10349.1	gene_id	MSTRG.10349;
13	StringTie	transcrip	87732443	87735798	.	+	.	transcript_	MSTRG.10349.2	gene_id	MSTRG.10349;
13	StringTie	exon	87732443	87732527	.	+	.	transcript_	MSTRG.10349.2	gene_id	MSTRG.10349;
13	StringTie	exon	87733720	87735798	.	+	.	transcript_	MSTRG.10349.2	gene_id	MSTRG.10349;
13	StringTie	transcrip	90857811	90859471	.	+	.	transcript_	MSTRG.10426.1	gene_id	MSTRG.10426;
13	StringTie	exon	90857811	90858100	.	+	.	transcript_	MSTRG.10426.1	gene_id	MSTRG.10426;
13	StringTie	exon	90858186	90859471	.	+	.	transcript_	MSTRG.10426.1	gene_id	MSTRG.10426;
13	StringTie	transcrip	93051963	93054117	.	+	.	transcript_	MSTRG.10470.2	gene_id	MSTRG.10470;
13	StringTie	exon	93051963	93052078	.	+	.	transcript_	MSTRG.10470.2	gene_id	MSTRG.10470;
13	StringTie	exon	93052418	93052476	.	+	.	transcript_	MSTRG.10470.2	gene_id	MSTRG.10470;
13	StringTie	exon	93053068	93054117	.	+	.	transcript_	MSTRG.10470.2	gene_id	MSTRG.10470;
13	StringTie	transcrip	93051963	93054117	.	+	.	transcript_	MSTRG.10470.1	gene_id	MSTRG.10470;
13	StringTie	exon	93051963	93052078	.	+	.	transcript_	MSTRG.10470.1	gene_id	MSTRG.10470;
13	StringTie	exon	93052418	93052668	.	+	.	transcript_	MSTRG.10470.1	gene_id	MSTRG.10470;
13	StringTie	exon	93053068	93054117	.	+	.	transcript_	MSTRG.10470.1	gene_id	MSTRG.10470;
13	StringTie	transcrip	98463259	98464777	.	+	.	transcript_	MSTRG.10577.1	gene_id	MSTRG.10577;
13	StringTie	exon	98463259	98463495	.	+	.	transcript_	MSTRG.10577.1	gene_id	MSTRG.10577;
13	StringTie	exon	98464692	98464777	.	+	.	transcript_	MSTRG.10577.1	gene_id	MSTRG.10577;
13	StringTie	transcrip	98463289	98474497	.	+	.	transcript_	MSTRG.10577.2	gene_id	MSTRG.10577;

13	StringTie	exon	98463289	98463495	.	+	.	transcript_	MSTRG.10577.2	gene_id	MSTRG.10577;
13	StringTie	exon	98472454	98472670	.	+	.	transcript_	MSTRG.10577.2	gene_id	MSTRG.10577;
13	StringTie	exon	98474241	98474497	.	+	.	transcript_	MSTRG.10577.2	gene_id	MSTRG.10577;
13	StringTie	transcrip	99821133	99837891	.	+	.	transcript_	MSTRG.10581.1	gene_id	MSTRG.10581;
13	StringTie	exon	99821133	99821363	.	+	.	transcript_	MSTRG.10581.1	gene_id	MSTRG.10581;
13	StringTie	exon	99837613	99837891	.	+	.	transcript_	MSTRG.10581.1	gene_id	MSTRG.10581;
13	StringTie	transcrip	107711431	107732570	.	+	.	transcript_	MSTRG.10624.1	gene_id	MSTRG.10624;
13	StringTie	exon	107711431	107711666	.	+	.	transcript_	MSTRG.10624.1	gene_id	MSTRG.10624;
13	StringTie	exon	107731582	107731909	.	+	.	transcript_	MSTRG.10624.1	gene_id	MSTRG.10624;
13	StringTie	exon	107732053	107732570	.	+	.	transcript_	MSTRG.10624.1	gene_id	MSTRG.10624;
13	StringTie	transcrip	110054204	110156883	.	+	.	transcript_	MSTRG.10667.1	gene_id	MSTRG.10667;
13	StringTie	exon	110054204	110054293	.	+	.	transcript_	MSTRG.10667.1	gene_id	MSTRG.10667;
13	StringTie	exon	110054885	110054977	.	+	.	transcript_	MSTRG.10667.1	gene_id	MSTRG.10667;
13	StringTie	exon	110059394	110059566	.	+	.	transcript_	MSTRG.10667.1	gene_id	MSTRG.10667;
13	StringTie	exon	110135288	110135415	.	+	.	transcript_	MSTRG.10667.1	gene_id	MSTRG.10667;
13	StringTie	exon	110156827	110156883	.	+	.	transcript_	MSTRG.10667.1	gene_id	MSTRG.10667;
13	StringTie	transcrip	110054937	110088658	.	+	.	transcript_	MSTRG.10667.2	gene_id	MSTRG.10667;
13	StringTie	exon	110054937	110054977	.	+	.	transcript_	MSTRG.10667.2	gene_id	MSTRG.10667;
13	StringTie	exon	110059394	110059566	.	+	.	transcript_	MSTRG.10667.2	gene_id	MSTRG.10667;
13	StringTie	exon	110088415	110088658	.	+	.	transcript_	MSTRG.10667.2	gene_id	MSTRG.10667;
13	StringTie	transcrip	120626650	120714264	.	+	.	transcript_	MSTRG.10770.2	gene_id	MSTRG.10770;
13	StringTie	exon	120626650	120626743	.	+	.	transcript_	MSTRG.10770.2	gene_id	MSTRG.10770;
13	StringTie	exon	120703772	120703829	.	+	.	transcript_	MSTRG.10770.2	gene_id	MSTRG.10770;
13	StringTie	exon	120712278	120712413	.	+	.	transcript_	MSTRG.10770.2	gene_id	MSTRG.10770;
13	StringTie	exon	120714194	120714264	.	+	.	transcript_	MSTRG.10770.2	gene_id	MSTRG.10770;
13	StringTie	transcrip	120717804	120719319	.	+	.	transcript_	MSTRG.10773.1	gene_id	MSTRG.10773;
13	StringTie	exon	120717804	120718075	.	+	.	transcript_	MSTRG.10773.1	gene_id	MSTRG.10773;
13	StringTie	exon	120718604	120719319	.	+	.	transcript_	MSTRG.10773.1	gene_id	MSTRG.10773;
13	StringTie	transcrip	121378454	121431080	.	+	.	transcript_	MSTRG.10822.1	gene_id	MSTRG.10822;
13	StringTie	exon	121378454	121378660	.	+	.	transcript_	MSTRG.10822.1	gene_id	MSTRG.10822;
13	StringTie	exon	121386475	121387023	.	+	.	transcript_	MSTRG.10822.1	gene_id	MSTRG.10822;
13	StringTie	exon	121393206	121393363	.	+	.	transcript_	MSTRG.10822.1	gene_id	MSTRG.10822;
13	StringTie	exon	121394753	121394913	.	+	.	transcript_	MSTRG.10822.1	gene_id	MSTRG.10822;
13	StringTie	exon	121395151	121395291	.	+	.	transcript_	MSTRG.10822.1	gene_id	MSTRG.10822;
13	StringTie	exon	121398387	121398502	.	+	.	transcript_	MSTRG.10822.1	gene_id	MSTRG.10822;

13	StringTie	exon	121428671	121431080	.	+	.	transcript_	MSTRG.10822.1	gene_id	MSTRG.10822;
13	StringTie	transcrip	125417977	125418465	.	+	.	transcript_	MSTRG.10922.1	gene_id	MSTRG.10922;
13	StringTie	exon	125417977	125418108	.	+	.	transcript_	MSTRG.10922.1	gene_id	MSTRG.10922;
13	StringTie	exon	125418380	125418465	.	+	.	transcript_	MSTRG.10922.1	gene_id	MSTRG.10922;
13	StringTie	transcrip	129626983	129631365	.	+	.	transcript_	MSTRG.10963.1	gene_id	MSTRG.10963;
13	StringTie	exon	129626983	129627069	.	+	.	transcript_	MSTRG.10963.1	gene_id	MSTRG.10963;
13	StringTie	exon	129628196	129628455	.	+	.	transcript_	MSTRG.10963.1	gene_id	MSTRG.10963;
13	StringTie	exon	129629808	129629867	.	+	.	transcript_	MSTRG.10963.1	gene_id	MSTRG.10963;
13	StringTie	exon	129631129	129631365	.	+	.	transcript_	MSTRG.10963.1	gene_id	MSTRG.10963;
13	StringTie	transcrip	129628354	129631918	.	+	.	transcript_	MSTRG.10963.2	gene_id	MSTRG.10963;
13	StringTie	exon	129628354	129628455	.	+	.	transcript_	MSTRG.10963.2	gene_id	MSTRG.10963;
13	StringTie	exon	129631129	129631285	.	+	.	transcript_	MSTRG.10963.2	gene_id	MSTRG.10963;
13	StringTie	exon	129631833	129631918	.	+	.	transcript_	MSTRG.10963.2	gene_id	MSTRG.10963;
13	StringTie	transcrip	132480175	132515188	.	+	.	transcript_	MSTRG.11007.1	gene_id	MSTRG.11007;
13	StringTie	exon	132480175	132480221	.	+	.	transcript_	MSTRG.11007.1	gene_id	MSTRG.11007;
13	StringTie	exon	132512016	132513368	.	+	.	transcript_	MSTRG.11007.1	gene_id	MSTRG.11007;
13	StringTie	exon	132515079	132515188	.	+	.	transcript_	MSTRG.11007.1	gene_id	MSTRG.11007;
13	StringTie	transcrip	135830354	135837592	.	+	.	transcript_	MSTRG.11106.1	gene_id	MSTRG.11106;
13	StringTie	exon	135830354	135830422	.	+	.	transcript_	MSTRG.11106.1	gene_id	MSTRG.11106;
13	StringTie	exon	135836787	135837592	.	+	.	transcript_	MSTRG.11106.1	gene_id	MSTRG.11106;
13	StringTie	transcrip	136146165	136147195	.	+	.	transcript_	MSTRG.11109.1	gene_id	MSTRG.11109;
13	StringTie	exon	136146165	136146241	.	+	.	transcript_	MSTRG.11109.1	gene_id	MSTRG.11109;
13	StringTie	exon	136146737	136147195	.	+	.	transcript_	MSTRG.11109.1	gene_id	MSTRG.11109;
13	StringTie	transcrip	144857906	144973584	.	+	.	transcript_	MSTRG.11281.4	gene_id	MSTRG.11281;
13	StringTie	exon	144857906	144858087	.	+	.	transcript_	MSTRG.11281.4	gene_id	MSTRG.11281;
13	StringTie	exon	144973562	144973584	.	+	.	transcript_	MSTRG.11281.4	gene_id	MSTRG.11281;
13	StringTie	transcrip	144903637	145213807	.	+	.	transcript_	MSTRG.11281.8	gene_id	MSTRG.11281;
13	StringTie	exon	144903637	144903764	.	+	.	transcript_	MSTRG.11281.8	gene_id	MSTRG.11281;
13	StringTie	exon	145041194	145041245	.	+	.	transcript_	MSTRG.11281.8	gene_id	MSTRG.11281;
13	StringTie	exon	145191075	145191148	.	+	.	transcript_	MSTRG.11281.8	gene_id	MSTRG.11281;
13	StringTie	exon	145213606	145213807	.	+	.	transcript_	MSTRG.11281.8	gene_id	MSTRG.11281;
13	StringTie	transcrip	146759583	146764963	.	+	.	transcript_	MSTRG.11309.2	gene_id	MSTRG.11309;
13	StringTie	exon	146759583	146759856	.	+	.	transcript_	MSTRG.11309.2	gene_id	MSTRG.11309;
13	StringTie	exon	146764727	146764963	.	+	.	transcript_	MSTRG.11309.2	gene_id	MSTRG.11309;
13	StringTie	transcrip	146759583	146768463	.	+	.	transcript_	MSTRG.11309.1	gene_id	MSTRG.11309;

13	StringTie	exon	146759583	146763431	.	+	.	transcript_	MSTRG.11309.1	gene_id	MSTRG.11309;
13	StringTie	exon	146768302	146768463	.	+	.	transcript_	MSTRG.11309.1	gene_id	MSTRG.11309;
13	StringTie	transcrip	147900358	147901250	.	+	.	transcript_	MSTRG.11369.1	gene_id	MSTRG.11369;
13	StringTie	exon	147900358	147900386	.	+	.	transcript_	MSTRG.11369.1	gene_id	MSTRG.11369;
13	StringTie	exon	147900561	147901250	.	+	.	transcript_	MSTRG.11369.1	gene_id	MSTRG.11369;
13	StringTie	transcrip	167811339	167876290	.	+	.	transcript_	MSTRG.11522.1	gene_id	MSTRG.11522;
13	StringTie	exon	167811339	167811414	.	+	.	transcript_	MSTRG.11522.1	gene_id	MSTRG.11522;
13	StringTie	exon	167875565	167876290	.	+	.	transcript_	MSTRG.11522.1	gene_id	MSTRG.11522;
13	StringTie	transcrip	168575027	168644921	.	+	.	transcript_	MSTRG.11511.1	gene_id	MSTRG.11511;
13	StringTie	exon	168575027	168575065	.	+	.	transcript_	MSTRG.11511.1	gene_id	MSTRG.11511;
13	StringTie	exon	168580611	168580730	.	+	.	transcript_	MSTRG.11511.1	gene_id	MSTRG.11511;
13	StringTie	exon	168592440	168592481	.	+	.	transcript_	MSTRG.11511.1	gene_id	MSTRG.11511;
13	StringTie	exon	168618153	168618204	.	+	.	transcript_	MSTRG.11511.1	gene_id	MSTRG.11511;
13	StringTie	exon	168620707	168620729	.	+	.	transcript_	MSTRG.11511.1	gene_id	MSTRG.11511;
13	StringTie	exon	168644731	168644921	.	+	.	transcript_	MSTRG.11511.1	gene_id	MSTRG.11511;
13	StringTie	transcrip	180020600	180035548	.	+	.	transcript_	MSTRG.11671.1	gene_id	MSTRG.11671;
13	StringTie	exon	180020600	180020671	.	+	.	transcript_	MSTRG.11671.1	gene_id	MSTRG.11671;
13	StringTie	exon	180023402	180023584	.	+	.	transcript_	MSTRG.11671.1	gene_id	MSTRG.11671;
13	StringTie	exon	180027489	180027631	.	+	.	transcript_	MSTRG.11671.1	gene_id	MSTRG.11671;
13	StringTie	exon	180027889	180028249	.	+	.	transcript_	MSTRG.11671.1	gene_id	MSTRG.11671;
13	StringTie	exon	180029506	180029717	.	+	.	transcript_	MSTRG.11671.1	gene_id	MSTRG.11671;
13	StringTie	exon	180034384	180035548	.	+	.	transcript_	MSTRG.11671.1	gene_id	MSTRG.11671;
13	StringTie	transcrip	180023417	180035085	.	+	.	transcript_	MSTRG.11671.2	gene_id	MSTRG.11671;
13	StringTie	exon	180023417	180023584	.	+	.	transcript_	MSTRG.11671.2	gene_id	MSTRG.11671;
13	StringTie	exon	180027489	180027631	.	+	.	transcript_	MSTRG.11671.2	gene_id	MSTRG.11671;
13	StringTie	exon	180027808	180028249	.	+	.	transcript_	MSTRG.11671.2	gene_id	MSTRG.11671;
13	StringTie	exon	180029506	180029717	.	+	.	transcript_	MSTRG.11671.2	gene_id	MSTRG.11671;
13	StringTie	exon	180034384	180035085	.	+	.	transcript_	MSTRG.11671.2	gene_id	MSTRG.11671;
13	StringTie	transcrip	180027000	180035442	.	+	.	transcript_	MSTRG.11671.3	gene_id	MSTRG.11671;
13	StringTie	exon	180027000	180027058	.	+	.	transcript_	MSTRG.11671.3	gene_id	MSTRG.11671;
13	StringTie	exon	180027489	180027631	.	+	.	transcript_	MSTRG.11671.3	gene_id	MSTRG.11671;
13	StringTie	exon	180027808	180028249	.	+	.	transcript_	MSTRG.11671.3	gene_id	MSTRG.11671;
13	StringTie	exon	180029506	180029717	.	+	.	transcript_	MSTRG.11671.3	gene_id	MSTRG.11671;
13	StringTie	exon	180034384	180035442	.	+	.	transcript_	MSTRG.11671.3	gene_id	MSTRG.11671;
13	StringTie	transcrip	180803997	180844246	.	+	.	transcript_	MSTRG.11697.1	gene_id	MSTRG.11697;

13	StringTie	exon	180803997	180804159	.	+	.	transcript_	MSTRG.11697.1	gene_id	MSTRG.11697;
13	StringTie	exon	180804274	180804376	.	+	.	transcript_	MSTRG.11697.1	gene_id	MSTRG.11697;
13	StringTie	exon	180844019	180844246	.	+	.	transcript_	MSTRG.11697.1	gene_id	MSTRG.11697;
13	StringTie	transcrip	181165625	181301470	.	+	.	transcript_	MSTRG.11741.1	gene_id	MSTRG.11741;
13	StringTie	exon	181165625	181165695	.	+	.	transcript_	MSTRG.11741.1	gene_id	MSTRG.11741;
13	StringTie	exon	181300807	181301470	.	+	.	transcript_	MSTRG.11741.1	gene_id	MSTRG.11741;
13	StringTie	transcrip	181188243	181302161	.	+	.	transcript_	MSTRG.11741.2	gene_id	MSTRG.11741;
13	StringTie	exon	181188243	181189997	.	+	.	transcript_	MSTRG.11741.2	gene_id	MSTRG.11741;
13	StringTie	exon	181246799	181246844	.	+	.	transcript_	MSTRG.11741.2	gene_id	MSTRG.11741;
13	StringTie	exon	181300807	181302161	.	+	.	transcript_	MSTRG.11741.2	gene_id	MSTRG.11741;
13	StringTie	transcrip	181189753	181246834	.	+	.	transcript_	MSTRG.11741.3	gene_id	MSTRG.11741;
13	StringTie	exon	181189753	181190113	.	+	.	transcript_	MSTRG.11741.3	gene_id	MSTRG.11741;
13	StringTie	exon	181246799	181246834	.	+	.	transcript_	MSTRG.11741.3	gene_id	MSTRG.11741;
13	StringTie	transcrip	181296806	181302170	.	+	.	transcript_	MSTRG.11741.4	gene_id	MSTRG.11741;
13	StringTie	exon	181296806	181299127	.	+	.	transcript_	MSTRG.11741.4	gene_id	MSTRG.11741;
13	StringTie	exon	181300807	181302170	.	+	.	transcript_	MSTRG.11741.4	gene_id	MSTRG.11741;
13	StringTie	transcrip	189994542	189997495	.	+	.	transcript_	MSTRG.11835.1	gene_id	MSTRG.11835;
13	StringTie	exon	189994542	189994607	.	+	.	transcript_	MSTRG.11835.1	gene_id	MSTRG.11835;
13	StringTie	exon	189997136	189997495	.	+	.	transcript_	MSTRG.11835.1	gene_id	MSTRG.11835;
13	StringTie	transcrip	190009831	190016417	.	+	.	transcript_	MSTRG.11836.1	gene_id	MSTRG.11836;
13	StringTie	exon	190009831	190011039	.	+	.	transcript_	MSTRG.11836.1	gene_id	MSTRG.11836;
13	StringTie	exon	190011986	190012071	.	+	.	transcript_	MSTRG.11836.1	gene_id	MSTRG.11836;
13	StringTie	exon	190012698	190012812	.	+	.	transcript_	MSTRG.11836.1	gene_id	MSTRG.11836;
13	StringTie	exon	190014822	190016417	.	+	.	transcript_	MSTRG.11836.1	gene_id	MSTRG.11836;
13	StringTie	transcrip	190024253	190030530	.	+	.	transcript_	MSTRG.11837.1	gene_id	MSTRG.11837;
13	StringTie	exon	190024253	190025055	.	+	.	transcript_	MSTRG.11837.1	gene_id	MSTRG.11837;
13	StringTie	exon	190030007	190030530	.	+	.	transcript_	MSTRG.11837.1	gene_id	MSTRG.11837;
13	StringTie	transcrip	196805626	196811036	.	+	.	transcript_	MSTRG.11905.1	gene_id	MSTRG.11905;
13	StringTie	exon	196805626	196807816	.	+	.	transcript_	MSTRG.11905.1	gene_id	MSTRG.11905;
13	StringTie	exon	196810899	196811036	.	+	.	transcript_	MSTRG.11905.1	gene_id	MSTRG.11905;
13	StringTie	transcrip	197445885	197455184	.	+	.	transcript_	MSTRG.11937.9	gene_id	MSTRG.11937;
13	StringTie	exon	197445885	197446039	.	+	.	transcript_	MSTRG.11937.9	gene_id	MSTRG.11937;
13	StringTie	exon	197454772	197455184	.	+	.	transcript_	MSTRG.11937.9	gene_id	MSTRG.11937;
13	StringTie	transcrip	197791226	197793959	.	+	.	transcript_	MSTRG.11918.1	gene_id	MSTRG.11918;
13	StringTie	exon	197791226	197791325	.	+	.	transcript_	MSTRG.11918.1	gene_id	MSTRG.11918;

13	StringTie	exon	197793251	197793959	.	+	.	transcript_	MSTRG.11918.1	gene_id	MSTRG.11918;
13	StringTie	transcrip	207493903	207494436	.	+	.	transcript_	MSTRG.12060.1	gene_id	MSTRG.12060;
13	StringTie	exon	207493903	207493966	.	+	.	transcript_	MSTRG.12060.1	gene_id	MSTRG.12060;
13	StringTie	exon	207494031	207494224	.	+	.	transcript_	MSTRG.12060.1	gene_id	MSTRG.12060;
13	StringTie	exon	207494327	207494436	.	+	.	transcript_	MSTRG.12060.1	gene_id	MSTRG.12060;
13	StringTie	transcrip	3257326	3258467	.	-	.	transcript_	MSTRG.8824.1	gene_id	MSTRG.8824;
13	StringTie	exon	3257326	3257508	.	-	.	transcript_	MSTRG.8824.1	gene_id	MSTRG.8824;
13	StringTie	exon	3258365	3258467	.	-	.	transcript_	MSTRG.8824.1	gene_id	MSTRG.8824;
13	StringTie	transcrip	3915559	3918648	.	-	.	transcript_	MSTRG.8858.1	gene_id	MSTRG.8858;
13	StringTie	exon	3915559	3915724	.	-	.	transcript_	MSTRG.8858.1	gene_id	MSTRG.8858;
13	StringTie	exon	3918603	3918648	.	-	.	transcript_	MSTRG.8858.1	gene_id	MSTRG.8858;
13	StringTie	transcrip	11399146	11423381	.	-	.	transcript_	MSTRG.8891.1	gene_id	MSTRG.8891;
13	StringTie	exon	11399146	11399898	.	-	.	transcript_	MSTRG.8891.1	gene_id	MSTRG.8891;
13	StringTie	exon	11401284	11401324	.	-	.	transcript_	MSTRG.8891.1	gene_id	MSTRG.8891;
13	StringTie	exon	11422487	11422652	.	-	.	transcript_	MSTRG.8891.1	gene_id	MSTRG.8891;
13	StringTie	exon	11423279	11423381	.	-	.	transcript_	MSTRG.8891.1	gene_id	MSTRG.8891;
13	StringTie	transcrip	21386550	21388006	.	-	.	transcript_	MSTRG.9013.1	gene_id	MSTRG.9013;
13	StringTie	exon	21386550	21386644	.	-	.	transcript_	MSTRG.9013.1	gene_id	MSTRG.9013;
13	StringTie	exon	21387893	21388006	.	-	.	transcript_	MSTRG.9013.1	gene_id	MSTRG.9013;
13	StringTie	transcrip	21593973	21670366	.	-	.	transcript_	MSTRG.9029.1	gene_id	MSTRG.9029;
13	StringTie	exon	21593973	21594054	.	-	.	transcript_	MSTRG.9029.1	gene_id	MSTRG.9029;
13	StringTie	exon	21670091	21670366	.	-	.	transcript_	MSTRG.9029.1	gene_id	MSTRG.9029;
13	StringTie	transcrip	24465923	24528245	.	-	.	transcript_	MSTRG.9109.1	gene_id	MSTRG.9109;
13	StringTie	exon	24465923	24466010	.	-	.	transcript_	MSTRG.9109.1	gene_id	MSTRG.9109;
13	StringTie	exon	24475450	24475580	.	-	.	transcript_	MSTRG.9109.1	gene_id	MSTRG.9109;
13	StringTie	exon	24528144	24528245	.	-	.	transcript_	MSTRG.9109.1	gene_id	MSTRG.9109;
13	StringTie	transcrip	34917268	34919484	.	-	.	transcript_	MSTRG.9510.1	gene_id	MSTRG.9510;
13	StringTie	exon	34917268	34917441	.	-	.	transcript_	MSTRG.9510.1	gene_id	MSTRG.9510;
13	StringTie	exon	34919393	34919484	.	-	.	transcript_	MSTRG.9510.1	gene_id	MSTRG.9510;
13	StringTie	transcrip	43860799	43897210	.	-	.	transcript_	MSTRG.9641.1	gene_id	MSTRG.9641;
13	StringTie	exon	43860799	43862423	.	-	.	transcript_	MSTRG.9641.1	gene_id	MSTRG.9641;
13	StringTie	exon	43866883	43866961	.	-	.	transcript_	MSTRG.9641.1	gene_id	MSTRG.9641;
13	StringTie	exon	43897065	43897210	.	-	.	transcript_	MSTRG.9641.1	gene_id	MSTRG.9641;
13	StringTie	transcrip	47142930	47143389	.	-	.	transcript_	MSTRG.9707.1	gene_id	MSTRG.9707;
13	StringTie	exon	47142930	47143241	.	-	.	transcript_	MSTRG.9707.1	gene_id	MSTRG.9707;

13	StringTie	exon	47143348	47143389	.	-	.	transcript_	MSTRG.9707.1	gene_id	MSTRG.9707;
13	StringTie	transcrip	48667789	48691314	.	-	.	transcript_	MSTRG.9771.3	gene_id	MSTRG.9771;
13	StringTie	exon	48667789	48668198	.	-	.	transcript_	MSTRG.9771.3	gene_id	MSTRG.9771;
13	StringTie	exon	48691256	48691314	.	-	.	transcript_	MSTRG.9771.3	gene_id	MSTRG.9771;
13	StringTie	transcrip	60303142	60315765	.	-	.	transcript_	MSTRG.9866.1	gene_id	MSTRG.9866;
13	StringTie	exon	60303142	60303183	.	-	.	transcript_	MSTRG.9866.1	gene_id	MSTRG.9866;
13	StringTie	exon	60315509	60315765	.	-	.	transcript_	MSTRG.9866.1	gene_id	MSTRG.9866;
13	StringTie	transcrip	60404812	60555807	.	-	.	transcript_	MSTRG.9874.2	gene_id	MSTRG.9874;
13	StringTie	exon	60404812	60404884	.	-	.	transcript_	MSTRG.9874.2	gene_id	MSTRG.9874;
13	StringTie	exon	60496301	60496447	.	-	.	transcript_	MSTRG.9874.2	gene_id	MSTRG.9874;
13	StringTie	exon	60555753	60555807	.	-	.	transcript_	MSTRG.9874.2	gene_id	MSTRG.9874;
13	StringTie	transcrip	61368416	61375154	.	-	.	transcript_	MSTRG.9918.1	gene_id	MSTRG.9918;
13	StringTie	exon	61368416	61368539	.	-	.	transcript_	MSTRG.9918.1	gene_id	MSTRG.9918;
13	StringTie	exon	61369177	61369303	.	-	.	transcript_	MSTRG.9918.1	gene_id	MSTRG.9918;
13	StringTie	exon	61374652	61375154	.	-	.	transcript_	MSTRG.9918.1	gene_id	MSTRG.9918;
13	StringTie	transcrip	61368416	61375163	.	-	.	transcript_	MSTRG.9918.2	gene_id	MSTRG.9918;
13	StringTie	exon	61368416	61368536	.	-	.	transcript_	MSTRG.9918.2	gene_id	MSTRG.9918;
13	StringTie	exon	61369177	61369303	.	-	.	transcript_	MSTRG.9918.2	gene_id	MSTRG.9918;
13	StringTie	exon	61374652	61375163	.	-	.	transcript_	MSTRG.9918.2	gene_id	MSTRG.9918;
13	StringTie	transcrip	61435760	61439071	.	-	.	transcript_	MSTRG.9924.1	gene_id	MSTRG.9924;
13	StringTie	exon	61435760	61436722	.	-	.	transcript_	MSTRG.9924.1	gene_id	MSTRG.9924;
13	StringTie	exon	61438725	61439071	.	-	.	transcript_	MSTRG.9924.1	gene_id	MSTRG.9924;
13	StringTie	transcrip	61442833	61528398	.	-	.	transcript_	MSTRG.9886.1	gene_id	MSTRG.9886;
13	StringTie	exon	61442833	61443052	.	-	.	transcript_	MSTRG.9886.1	gene_id	MSTRG.9886;
13	StringTie	exon	61453739	61453804	.	-	.	transcript_	MSTRG.9886.1	gene_id	MSTRG.9886;
13	StringTie	exon	61488180	61488274	.	-	.	transcript_	MSTRG.9886.1	gene_id	MSTRG.9886;
13	StringTie	exon	61528354	61528398	.	-	.	transcript_	MSTRG.9886.1	gene_id	MSTRG.9886;
13	StringTie	transcrip	65731642	65750529	.	-	.	transcript_	MSTRG.9944.1	gene_id	MSTRG.9944;
13	StringTie	exon	65731642	65731857	.	-	.	transcript_	MSTRG.9944.1	gene_id	MSTRG.9944;
13	StringTie	exon	65750350	65750529	.	-	.	transcript_	MSTRG.9944.1	gene_id	MSTRG.9944;
13	StringTie	transcrip	70002166	70004600	.	-	.	transcript_	MSTRG.10057.	gene_id	MSTRG.10057;
13	StringTie	exon	70002166	70003036	.	-	.	transcript_	MSTRG.10057.	gene_id	MSTRG.10057;
13	StringTie	exon	70004441	70004600	.	-	.	transcript_	MSTRG.10057.	gene_id	MSTRG.10057;
13	StringTie	transcrip	71908517	71917564	.	-	.	transcript_	MSTRG.10087.	gene_id	MSTRG.10087;
13	StringTie	exon	71908517	71908626	.	-	.	transcript_	MSTRG.10087.	gene_id	MSTRG.10087;

13	StringTie	exon	71913367	71913551	.	-	.	transcript_	MSTRG.10087.:gene_id	MSTRG.10087;
13	StringTie	exon	71916698	71916906	.	-	.	transcript_	MSTRG.10087.:gene_id	MSTRG.10087;
13	StringTie	exon	71917437	71917564	.	-	.	transcript_	MSTRG.10087.:gene_id	MSTRG.10087;
13	StringTie	transcrip	73407532	73421548	.	-	.	transcript_	MSTRG.10121.:gene_id	MSTRG.10121;
13	StringTie	exon	73407532	73408128	.	-	.	transcript_	MSTRG.10121.:gene_id	MSTRG.10121;
13	StringTie	exon	73421515	73421548	.	-	.	transcript_	MSTRG.10121.:gene_id	MSTRG.10121;
13	StringTie	transcrip	79138794	79139419	.	-	.	transcript_	MSTRG.10194.:gene_id	MSTRG.10194;
13	StringTie	exon	79138794	79139180	.	-	.	transcript_	MSTRG.10194.:gene_id	MSTRG.10194;
13	StringTie	exon	79139401	79139419	.	-	.	transcript_	MSTRG.10194.:gene_id	MSTRG.10194;
13	StringTie	transcrip	81422520	81425588	.	-	.	transcript_	MSTRG.10225.:gene_id	MSTRG.10225;
13	StringTie	exon	81422520	81422902	.	-	.	transcript_	MSTRG.10225.:gene_id	MSTRG.10225;
13	StringTie	exon	81425567	81425588	.	-	.	transcript_	MSTRG.10225.:gene_id	MSTRG.10225;
13	StringTie	transcrip	84631196	84634004	.	-	.	transcript_	MSTRG.10323.:gene_id	MSTRG.10323;
13	StringTie	exon	84631196	84631380	.	-	.	transcript_	MSTRG.10323.:gene_id	MSTRG.10323;
13	StringTie	exon	84632733	84634004	.	-	.	transcript_	MSTRG.10323.:gene_id	MSTRG.10323;
13	StringTie	transcrip	86259840	86328197	.	-	.	transcript_	MSTRG.10319.:gene_id	MSTRG.10319;
13	StringTie	exon	86259840	86261009	.	-	.	transcript_	MSTRG.10319.:gene_id	MSTRG.10319;
13	StringTie	exon	86275793	86275926	.	-	.	transcript_	MSTRG.10319.:gene_id	MSTRG.10319;
13	StringTie	exon	86328136	86328197	.	-	.	transcript_	MSTRG.10319.:gene_id	MSTRG.10319;
13	StringTie	transcrip	90244803	90273387	.	-	.	transcript_	MSTRG.10403.:gene_id	MSTRG.10403;
13	StringTie	exon	90244803	90245048	.	-	.	transcript_	MSTRG.10403.:gene_id	MSTRG.10403;
13	StringTie	exon	90248167	90248267	.	-	.	transcript_	MSTRG.10403.:gene_id	MSTRG.10403;
13	StringTie	exon	90249310	90249386	.	-	.	transcript_	MSTRG.10403.:gene_id	MSTRG.10403;
13	StringTie	exon	90251207	90251324	.	-	.	transcript_	MSTRG.10403.:gene_id	MSTRG.10403;
13	StringTie	exon	90273284	90273387	.	-	.	transcript_	MSTRG.10403.:gene_id	MSTRG.10403;
13	StringTie	transcrip	95227609	95234870	.	-	.	transcript_	MSTRG.10511.:gene_id	MSTRG.10511;
13	StringTie	exon	95227609	95231664	.	-	.	transcript_	MSTRG.10511.:gene_id	MSTRG.10511;
13	StringTie	exon	95231763	95231858	.	-	.	transcript_	MSTRG.10511.:gene_id	MSTRG.10511;
13	StringTie	exon	95234313	95234504	.	-	.	transcript_	MSTRG.10511.:gene_id	MSTRG.10511;
13	StringTie	exon	95234838	95234870	.	-	.	transcript_	MSTRG.10511.:gene_id	MSTRG.10511;
13	StringTie	transcrip	98175531	98195106	.	-	.	transcript_	MSTRG.10564.:gene_id	MSTRG.10564;
13	StringTie	exon	98175531	98175549	.	-	.	transcript_	MSTRG.10564.:gene_id	MSTRG.10564;
13	StringTie	exon	98194748	98195106	.	-	.	transcript_	MSTRG.10564.:gene_id	MSTRG.10564;
13	StringTie	transcrip	109338051	109347283	.	-	.	transcript_	MSTRG.10653.:gene_id	MSTRG.10653;
13	StringTie	exon	109338051	109338278	.	-	.	transcript_	MSTRG.10653.:gene_id	MSTRG.10653;

13	StringTie	exon	109347185	109347283	.	-	.	transcript_	MSTRG.10653.1	gene_id	MSTRG.10653;
13	StringTie	transcrip	121549010	121559799	.	-	.	transcript_	MSTRG.10804.2	gene_id	MSTRG.10804;
13	StringTie	exon	121549010	121549714	.	-	.	transcript_	MSTRG.10804.2	gene_id	MSTRG.10804;
13	StringTie	exon	121558613	121558759	.	-	.	transcript_	MSTRG.10804.2	gene_id	MSTRG.10804;
13	StringTie	exon	121558929	121559011	.	-	.	transcript_	MSTRG.10804.2	gene_id	MSTRG.10804;
13	StringTie	exon	121559716	121559799	.	-	.	transcript_	MSTRG.10804.2	gene_id	MSTRG.10804;
13	StringTie	transcrip	121549010	121559799	.	-	.	transcript_	MSTRG.10804.1	gene_id	MSTRG.10804;
13	StringTie	exon	121549010	121549714	.	-	.	transcript_	MSTRG.10804.1	gene_id	MSTRG.10804;
13	StringTie	exon	121558929	121559011	.	-	.	transcript_	MSTRG.10804.1	gene_id	MSTRG.10804;
13	StringTie	exon	121559716	121559799	.	-	.	transcript_	MSTRG.10804.1	gene_id	MSTRG.10804;
13	StringTie	transcrip	124915819	124938420	.	-	.	transcript_	MSTRG.10897.1	gene_id	MSTRG.10897;
13	StringTie	exon	124915819	124915981	.	-	.	transcript_	MSTRG.10897.1	gene_id	MSTRG.10897;
13	StringTie	exon	124932804	124932984	.	-	.	transcript_	MSTRG.10897.1	gene_id	MSTRG.10897;
13	StringTie	exon	124938368	124938420	.	-	.	transcript_	MSTRG.10897.1	gene_id	MSTRG.10897;
13	StringTie	transcrip	132153049	132154165	.	-	.	transcript_	MSTRG.10994.1	gene_id	MSTRG.10994;
13	StringTie	exon	132153049	132153448	.	-	.	transcript_	MSTRG.10994.1	gene_id	MSTRG.10994;
13	StringTie	exon	132154038	132154165	.	-	.	transcript_	MSTRG.10994.1	gene_id	MSTRG.10994;
13	StringTie	transcrip	133968404	133970512	.	-	.	transcript_	MSTRG.11041.1	gene_id	MSTRG.11041;
13	StringTie	exon	133968404	133970038	.	-	.	transcript_	MSTRG.11041.1	gene_id	MSTRG.11041;
13	StringTie	exon	133970426	133970512	.	-	.	transcript_	MSTRG.11041.1	gene_id	MSTRG.11041;
13	StringTie	transcrip	133968965	133970497	.	-	.	transcript_	MSTRG.11041.2	gene_id	MSTRG.11041;
13	StringTie	exon	133968965	133970038	.	-	.	transcript_	MSTRG.11041.2	gene_id	MSTRG.11041;
13	StringTie	exon	133970421	133970497	.	-	.	transcript_	MSTRG.11041.2	gene_id	MSTRG.11041;
13	StringTie	transcrip	133969421	133970490	.	-	.	transcript_	MSTRG.11041.3	gene_id	MSTRG.11041;
13	StringTie	exon	133969421	133970038	.	-	.	transcript_	MSTRG.11041.3	gene_id	MSTRG.11041;
13	StringTie	exon	133970393	133970490	.	-	.	transcript_	MSTRG.11041.3	gene_id	MSTRG.11041;
13	StringTie	transcrip	134473249	134515125	.	-	.	transcript_	MSTRG.11052.1	gene_id	MSTRG.11052;
13	StringTie	exon	134473249	134473270	.	-	.	transcript_	MSTRG.11052.1	gene_id	MSTRG.11052;
13	StringTie	exon	134514545	134515125	.	-	.	transcript_	MSTRG.11052.1	gene_id	MSTRG.11052;
13	StringTie	transcrip	136962454	136964198	.	-	.	transcript_	MSTRG.11099.1	gene_id	MSTRG.11099;
13	StringTie	exon	136962454	136962740	.	-	.	transcript_	MSTRG.11099.1	gene_id	MSTRG.11099;
13	StringTie	exon	136963643	136964198	.	-	.	transcript_	MSTRG.11099.1	gene_id	MSTRG.11099;
13	StringTie	transcrip	138049498	138101645	.	-	.	transcript_	MSTRG.11137.1	gene_id	MSTRG.11137;
13	StringTie	exon	138049498	138054287	.	-	.	transcript_	MSTRG.11137.1	gene_id	MSTRG.11137;
13	StringTie	exon	138101550	138101645	.	-	.	transcript_	MSTRG.11137.1	gene_id	MSTRG.11137;

13	StringTie	transcrip	146269557	146274975	.	-	.	transcript_	MSTRG.11301.1	gene_id	MSTRG.11301;
13	StringTie	exon	146269557	146270697	.	-	.	transcript_	MSTRG.11301.1	gene_id	MSTRG.11301;
13	StringTie	exon	146274865	146274975	.	-	.	transcript_	MSTRG.11301.1	gene_id	MSTRG.11301;
13	StringTie	transcrip	159185247	159187576	.	-	.	transcript_	MSTRG.11443.1	gene_id	MSTRG.11443;
13	StringTie	exon	159185247	159185338	.	-	.	transcript_	MSTRG.11443.1	gene_id	MSTRG.11443;
13	StringTie	exon	159185370	159187576	.	-	.	transcript_	MSTRG.11443.1	gene_id	MSTRG.11443;
13	StringTie	transcrip	173471225	173583018	.	-	.	transcript_	MSTRG.11618.1	gene_id	MSTRG.11618;
13	StringTie	exon	173471225	173471548	.	-	.	transcript_	MSTRG.11618.1	gene_id	MSTRG.11618;
13	StringTie	exon	173566185	173566283	.	-	.	transcript_	MSTRG.11618.1	gene_id	MSTRG.11618;
13	StringTie	exon	173582911	173583018	.	-	.	transcript_	MSTRG.11618.1	gene_id	MSTRG.11618;
13	StringTie	transcrip	180200594	180209870	.	-	.	transcript_	MSTRG.11680.1	gene_id	MSTRG.11680;
13	StringTie	exon	180200594	180201073	.	-	.	transcript_	MSTRG.11680.1	gene_id	MSTRG.11680;
13	StringTie	exon	180207610	180208616	.	-	.	transcript_	MSTRG.11680.1	gene_id	MSTRG.11680;
13	StringTie	exon	180209737	180209870	.	-	.	transcript_	MSTRG.11680.1	gene_id	MSTRG.11680;
13	StringTie	transcrip	180201684	180208618	.	-	.	transcript_	MSTRG.11680.2	gene_id	MSTRG.11680;
13	StringTie	exon	180201684	180201859	.	-	.	transcript_	MSTRG.11680.2	gene_id	MSTRG.11680;
13	StringTie	exon	180207610	180208618	.	-	.	transcript_	MSTRG.11680.2	gene_id	MSTRG.11680;
13	StringTie	transcrip	188919729	188922691	.	-	.	transcript_	MSTRG.11806.1	gene_id	MSTRG.11806;
13	StringTie	exon	188919729	188919790	.	-	.	transcript_	MSTRG.11806.1	gene_id	MSTRG.11806;
13	StringTie	exon	188920480	188920625	.	-	.	transcript_	MSTRG.11806.1	gene_id	MSTRG.11806;
13	StringTie	exon	188922624	188922691	.	-	.	transcript_	MSTRG.11806.1	gene_id	MSTRG.11806;
13	StringTie	transcrip	195290972	195326318	.	-	.	transcript_	MSTRG.11871.1	gene_id	MSTRG.11871;
13	StringTie	exon	195290972	195291321	.	-	.	transcript_	MSTRG.11871.1	gene_id	MSTRG.11871;
13	StringTie	exon	195300700	195300809	.	-	.	transcript_	MSTRG.11871.1	gene_id	MSTRG.11871;
13	StringTie	exon	195326264	195326318	.	-	.	transcript_	MSTRG.11871.1	gene_id	MSTRG.11871;
13	StringTie	transcrip	196128839	196137201	.	-	.	transcript_	MSTRG.11884.1	gene_id	MSTRG.11884;
13	StringTie	exon	196128839	196129028	.	-	.	transcript_	MSTRG.11884.1	gene_id	MSTRG.11884;
13	StringTie	exon	196137133	196137201	.	-	.	transcript_	MSTRG.11884.1	gene_id	MSTRG.11884;
13	StringTie	transcrip	197005100	197006984	.	-	.	transcript_	MSTRG.11899.1	gene_id	MSTRG.11899;
13	StringTie	exon	197005100	197006159	.	-	.	transcript_	MSTRG.11899.1	gene_id	MSTRG.11899;
13	StringTie	exon	197006923	197006984	.	-	.	transcript_	MSTRG.11899.1	gene_id	MSTRG.11899;
13	StringTie	transcrip	200346877	200354811	.	-	.	transcript_	MSTRG.11948.1	gene_id	MSTRG.11948;
13	StringTie	exon	200346877	200347229	.	-	.	transcript_	MSTRG.11948.1	gene_id	MSTRG.11948;
13	StringTie	exon	200354756	200354811	.	-	.	transcript_	MSTRG.11948.1	gene_id	MSTRG.11948;
13	StringTie	transcrip	207030885	207042636	.	-	.	transcript_	MSTRG.12040.1	gene_id	MSTRG.12040;

13	StringTie	exon	207030885	207030952	.	-	.	transcript_	MSTRG.12040.1	gene_id	MSTRG.12040;
13	StringTie	exon	207041894	207042089	.	-	.	transcript_	MSTRG.12040.1	gene_id	MSTRG.12040;
13	StringTie	exon	207042493	207042636	.	-	.	transcript_	MSTRG.12040.1	gene_id	MSTRG.12040;
13	StringTie	transcrip	207040528	207042078	.	-	.	transcript_	MSTRG.12040.2	gene_id	MSTRG.12040;
13	StringTie	exon	207040528	207040616	.	-	.	transcript_	MSTRG.12040.2	gene_id	MSTRG.12040;
13	StringTie	exon	207041894	207042078	.	-	.	transcript_	MSTRG.12040.2	gene_id	MSTRG.12040;
13	StringTie	transcrip	207037987	207038672	.	-	.	transcript_	MSTRG.12042.1	gene_id	MSTRG.12042;
13	StringTie	exon	207037987	207038579	.	-	.	transcript_	MSTRG.12042.1	gene_id	MSTRG.12042;
13	StringTie	exon	207038600	207038672	.	-	.	transcript_	MSTRG.12042.1	gene_id	MSTRG.12042;
13	StringTie	transcrip	207667580	207676635	.	-	.	transcript_	MSTRG.12063.1	gene_id	MSTRG.12063;
13	StringTie	exon	207667580	207667608	.	-	.	transcript_	MSTRG.12063.1	gene_id	MSTRG.12063;
13	StringTie	exon	207671910	207673014	.	-	.	transcript_	MSTRG.12063.1	gene_id	MSTRG.12063;
13	StringTie	exon	207676555	207676635	.	-	.	transcript_	MSTRG.12063.1	gene_id	MSTRG.12063;
13	StringTie	transcrip	207778450	207782592	.	-	.	transcript_	MSTRG.12071.1	gene_id	MSTRG.12071;
13	StringTie	exon	207778450	207778727	.	-	.	transcript_	MSTRG.12071.1	gene_id	MSTRG.12071;
13	StringTie	exon	207782023	207782592	.	-	.	transcript_	MSTRG.12071.1	gene_id	MSTRG.12071;
14	StringTie	transcrip	1374532	1379753	.	+	.	transcript_	MSTRG.12095.1	gene_id	MSTRG.12095;
14	StringTie	exon	1374532	1375043	.	+	.	transcript_	MSTRG.12095.1	gene_id	MSTRG.12095;
14	StringTie	exon	1379644	1379753	.	+	.	transcript_	MSTRG.12095.1	gene_id	MSTRG.12095;
14	StringTie	transcrip	2377076	2403858	.	+	.	transcript_	MSTRG.12110.1	gene_id	MSTRG.12110;
14	StringTie	exon	2377076	2377104	.	+	.	transcript_	MSTRG.12110.1	gene_id	MSTRG.12110;
14	StringTie	exon	2401350	2403858	.	+	.	transcript_	MSTRG.12110.1	gene_id	MSTRG.12110;
14	StringTie	transcrip	7118642	7139902	.	+	.	transcript_	MSTRG.12183.1	gene_id	MSTRG.12183;
14	StringTie	exon	7118642	7118657	.	+	.	transcript_	MSTRG.12183.1	gene_id	MSTRG.12183;
14	StringTie	exon	7139257	7139902	.	+	.	transcript_	MSTRG.12183.1	gene_id	MSTRG.12183;
14	StringTie	transcrip	7261641	7266771	.	+	.	transcript_	MSTRG.12191.1	gene_id	MSTRG.12191;
14	StringTie	exon	7261641	7262434	.	+	.	transcript_	MSTRG.12191.1	gene_id	MSTRG.12191;
14	StringTie	exon	7266382	7266771	.	+	.	transcript_	MSTRG.12191.1	gene_id	MSTRG.12191;
14	StringTie	transcrip	11379377	11380480	.	+	.	transcript_	MSTRG.12265.1	gene_id	MSTRG.12265;
14	StringTie	exon	11379377	11379670	.	+	.	transcript_	MSTRG.12265.1	gene_id	MSTRG.12265;
14	StringTie	exon	11380357	11380480	.	+	.	transcript_	MSTRG.12265.1	gene_id	MSTRG.12265;
14	StringTie	transcrip	15059860	15064032	.	+	.	transcript_	MSTRG.12355.1	gene_id	MSTRG.12355;
14	StringTie	exon	15059860	15060032	.	+	.	transcript_	MSTRG.12355.1	gene_id	MSTRG.12355;
14	StringTie	exon	15060750	15060816	.	+	.	transcript_	MSTRG.12355.1	gene_id	MSTRG.12355;
14	StringTie	exon	15061510	15061768	.	+	.	transcript_	MSTRG.12355.1	gene_id	MSTRG.12355;

14	StringTie	exon	15062804	15064032	.	+	.	transcript_	MSTRG.12355.	gene_id	MSTRG.12355;
14	StringTie	transcrip	16621007	16621894	.	+	.	transcript_	MSTRG.12369.	gene_id	MSTRG.12369;
14	StringTie	exon	16621007	16621124	.	+	.	transcript_	MSTRG.12369.	gene_id	MSTRG.12369;
14	StringTie	exon	16621790	16621894	.	+	.	transcript_	MSTRG.12369.	gene_id	MSTRG.12369;
14	StringTie	transcrip	45250215	45334000	.	+	.	transcript_	MSTRG.12833.	gene_id	MSTRG.12833;
14	StringTie	exon	45250215	45250310	.	+	.	transcript_	MSTRG.12833.	gene_id	MSTRG.12833;
14	StringTie	exon	45318222	45318415	.	+	.	transcript_	MSTRG.12833.	gene_id	MSTRG.12833;
14	StringTie	exon	45333703	45334000	.	+	.	transcript_	MSTRG.12833.	gene_id	MSTRG.12833;
14	StringTie	transcrip	48051410	48061578	.	+	.	transcript_	MSTRG.12898.	gene_id	MSTRG.12898;
14	StringTie	exon	48051410	48051658	.	+	.	transcript_	MSTRG.12898.	gene_id	MSTRG.12898;
14	StringTie	exon	48061024	48061578	.	+	.	transcript_	MSTRG.12898.	gene_id	MSTRG.12898;
14	StringTie	transcrip	50370393	50372929	.	+	.	transcript_	MSTRG.12978.	gene_id	MSTRG.12978;
14	StringTie	exon	50370393	50371858	.	+	.	transcript_	MSTRG.12978.	gene_id	MSTRG.12978;
14	StringTie	exon	50372777	50372929	.	+	.	transcript_	MSTRG.12978.	gene_id	MSTRG.12978;
14	StringTie	transcrip	50898782	50900457	.	+	.	transcript_	MSTRG.13043.	gene_id	MSTRG.13043;
14	StringTie	exon	50898782	50898892	.	+	.	transcript_	MSTRG.13043.	gene_id	MSTRG.13043;
14	StringTie	exon	50900350	50900457	.	+	.	transcript_	MSTRG.13043.	gene_id	MSTRG.13043;
14	StringTie	transcrip	55368900	55428749	.	+	.	transcript_	MSTRG.13078.	gene_id	MSTRG.13078;
14	StringTie	exon	55368900	55369136	.	+	.	transcript_	MSTRG.13078.	gene_id	MSTRG.13078;
14	StringTie	exon	55390661	55390818	.	+	.	transcript_	MSTRG.13078.	gene_id	MSTRG.13078;
14	StringTie	exon	55427914	55428749	.	+	.	transcript_	MSTRG.13078.	gene_id	MSTRG.13078;
14	StringTie	transcrip	55602782	55604369	.	+	.	transcript_	MSTRG.13093.	gene_id	MSTRG.13093;
14	StringTie	exon	55602782	55603457	.	+	.	transcript_	MSTRG.13093.	gene_id	MSTRG.13093;
14	StringTie	exon	55603740	55604369	.	+	.	transcript_	MSTRG.13093.	gene_id	MSTRG.13093;
14	StringTie	transcrip	56128636	56129310	.	+	.	transcript_	MSTRG.13097.	gene_id	MSTRG.13097;
14	StringTie	exon	56128636	56128956	.	+	.	transcript_	MSTRG.13097.	gene_id	MSTRG.13097;
14	StringTie	exon	56129263	56129310	.	+	.	transcript_	MSTRG.13097.	gene_id	MSTRG.13097;
14	StringTie	transcrip	56604933	56608193	.	+	.	transcript_	MSTRG.13114.	gene_id	MSTRG.13114;
14	StringTie	exon	56604933	56605142	.	+	.	transcript_	MSTRG.13114.	gene_id	MSTRG.13114;
14	StringTie	exon	56607677	56608193	.	+	.	transcript_	MSTRG.13114.	gene_id	MSTRG.13114;
14	StringTie	transcrip	63492581	63493643	.	+	.	transcript_	MSTRG.13298.	gene_id	MSTRG.13298;
14	StringTie	exon	63492581	63493475	.	+	.	transcript_	MSTRG.13298.	gene_id	MSTRG.13298;
14	StringTie	exon	63493605	63493643	.	+	.	transcript_	MSTRG.13298.	gene_id	MSTRG.13298;
14	StringTie	transcrip	71645354	71651747	.	+	.	transcript_	MSTRG.13339.	gene_id	MSTRG.13339;
14	StringTie	exon	71645354	71645515	.	+	.	transcript_	MSTRG.13339.	gene_id	MSTRG.13339;

14	StringTie	exon	71651499	71651747	.	+	.	transcript_	MSTRG.13339.	gene_id	MSTRG.13339;
14	StringTie	transcrip	76305059	76310589	.	+	.	transcript_	MSTRG.13469.	gene_id	MSTRG.13469;
14	StringTie	exon	76305059	76305143	.	+	.	transcript_	MSTRG.13469.	gene_id	MSTRG.13469;
14	StringTie	exon	76308020	76308187	.	+	.	transcript_	MSTRG.13469.	gene_id	MSTRG.13469;
14	StringTie	exon	76308500	76310589	.	+	.	transcript_	MSTRG.13469.	gene_id	MSTRG.13469;
14	StringTie	transcrip	76444967	76445614	.	+	.	transcript_	MSTRG.13471.	gene_id	MSTRG.13471;
14	StringTie	exon	76444967	76445026	.	+	.	transcript_	MSTRG.13471.	gene_id	MSTRG.13471;
14	StringTie	exon	76445165	76445375	.	+	.	transcript_	MSTRG.13471.	gene_id	MSTRG.13471;
14	StringTie	exon	76445504	76445614	.	+	.	transcript_	MSTRG.13471.	gene_id	MSTRG.13471;
14	StringTie	transcrip	80912236	81056170	.	+	.	transcript_	MSTRG.13605.	gene_id	MSTRG.13605;
14	StringTie	exon	80912236	80912275	.	+	.	transcript_	MSTRG.13605.	gene_id	MSTRG.13605;
14	StringTie	exon	81055909	81056170	.	+	.	transcript_	MSTRG.13605.	gene_id	MSTRG.13605;
14	StringTie	transcrip	87900581	87900821	.	+	.	transcript_	MSTRG.13670.	gene_id	MSTRG.13670;
14	StringTie	exon	87900581	87900687	.	+	.	transcript_	MSTRG.13670.	gene_id	MSTRG.13670;
14	StringTie	exon	87900726	87900821	.	+	.	transcript_	MSTRG.13670.	gene_id	MSTRG.13670;
14	StringTie	transcrip	91960423	92015488	.	+	.	transcript_	MSTRG.13754.	gene_id	MSTRG.13754;
14	StringTie	exon	91960423	91960538	.	+	.	transcript_	MSTRG.13754.	gene_id	MSTRG.13754;
14	StringTie	exon	92015318	92015488	.	+	.	transcript_	MSTRG.13754.	gene_id	MSTRG.13754;
14	StringTie	transcrip	97609822	97629276	.	+	.	transcript_	MSTRG.13814.	gene_id	MSTRG.13814;
14	StringTie	exon	97609822	97609919	.	+	.	transcript_	MSTRG.13814.	gene_id	MSTRG.13814;
14	StringTie	exon	97629068	97629276	.	+	.	transcript_	MSTRG.13814.	gene_id	MSTRG.13814;
14	StringTie	transcrip	99762683	99810990	.	+	.	transcript_	MSTRG.13827.	gene_id	MSTRG.13827;
14	StringTie	exon	99762683	99762698	.	+	.	transcript_	MSTRG.13827.	gene_id	MSTRG.13827;
14	StringTie	exon	99809558	99810990	.	+	.	transcript_	MSTRG.13827.	gene_id	MSTRG.13827;
14	StringTie	transcrip	100689751	100692229	.	+	.	transcript_	MSTRG.13832.	gene_id	MSTRG.13832;
14	StringTie	exon	100689751	100689773	.	+	.	transcript_	MSTRG.13832.	gene_id	MSTRG.13832;
14	StringTie	exon	100691958	100692229	.	+	.	transcript_	MSTRG.13832.	gene_id	MSTRG.13832;
14	StringTie	transcrip	105039641	105043635	.	+	.	transcript_	MSTRG.13959.	gene_id	MSTRG.13959;
14	StringTie	exon	105039641	105040021	.	+	.	transcript_	MSTRG.13959.	gene_id	MSTRG.13959;
14	StringTie	exon	105043593	105043635	.	+	.	transcript_	MSTRG.13959.	gene_id	MSTRG.13959;
14	StringTie	transcrip	106993579	106997533	.	+	.	transcript_	MSTRG.14011.	gene_id	MSTRG.14011;
14	StringTie	exon	106993579	106994181	.	+	.	transcript_	MSTRG.14011.	gene_id	MSTRG.14011;
14	StringTie	exon	106995338	106997533	.	+	.	transcript_	MSTRG.14011.	gene_id	MSTRG.14011;
14	StringTie	transcrip	112734970	112747428	.	+	.	transcript_	MSTRG.14135.	gene_id	MSTRG.14135;
14	StringTie	exon	112734970	112735077	.	+	.	transcript_	MSTRG.14135.	gene_id	MSTRG.14135;

14	StringTie	exon	112746646	112747428	.	+	.	transcript_	MSTRG.14135.1	gene_id	MSTRG.14135;
14	StringTie	transcrip	114064858	114065882	.	+	.	transcript_	MSTRG.14182.1	gene_id	MSTRG.14182;
14	StringTie	exon	114064858	114065031	.	+	.	transcript_	MSTRG.14182.1	gene_id	MSTRG.14182;
14	StringTie	exon	114065816	114065882	.	+	.	transcript_	MSTRG.14182.1	gene_id	MSTRG.14182;
14	StringTie	transcrip	120326287	120327058	.	+	.	transcript_	MSTRG.14222.1	gene_id	MSTRG.14222;
14	StringTie	exon	120326287	120326419	.	+	.	transcript_	MSTRG.14222.1	gene_id	MSTRG.14222;
14	StringTie	exon	120326725	120327058	.	+	.	transcript_	MSTRG.14222.1	gene_id	MSTRG.14222;
14	StringTie	transcrip	122584350	122607227	.	+	.	transcript_	MSTRG.14237.1	gene_id	MSTRG.14237;
14	StringTie	exon	122584350	122584680	.	+	.	transcript_	MSTRG.14237.1	gene_id	MSTRG.14237;
14	StringTie	exon	122606918	122607227	.	+	.	transcript_	MSTRG.14237.1	gene_id	MSTRG.14237;
14	StringTie	transcrip	123947234	123949582	.	+	.	transcript_	MSTRG.14296.1	gene_id	MSTRG.14296;
14	StringTie	exon	123947234	123947339	.	+	.	transcript_	MSTRG.14296.1	gene_id	MSTRG.14296;
14	StringTie	exon	123947505	123949582	.	+	.	transcript_	MSTRG.14296.1	gene_id	MSTRG.14296;
14	StringTie	transcrip	124134276	124135713	.	+	.	transcript_	MSTRG.14286.1	gene_id	MSTRG.14286;
14	StringTie	exon	124134276	124134444	.	+	.	transcript_	MSTRG.14286.1	gene_id	MSTRG.14286;
14	StringTie	exon	124135683	124135713	.	+	.	transcript_	MSTRG.14286.1	gene_id	MSTRG.14286;
14	StringTie	transcrip	124134317	124148926	.	+	.	transcript_	MSTRG.14286.2	gene_id	MSTRG.14286;
14	StringTie	exon	124134317	124134444	.	+	.	transcript_	MSTRG.14286.2	gene_id	MSTRG.14286;
14	StringTie	exon	124148821	124148926	.	+	.	transcript_	MSTRG.14286.2	gene_id	MSTRG.14286;
14	StringTie	transcrip	124521419	124522678	.	+	.	transcript_	MSTRG.14293.1	gene_id	MSTRG.14293;
14	StringTie	exon	124521419	124521609	.	+	.	transcript_	MSTRG.14293.1	gene_id	MSTRG.14293;
14	StringTie	exon	124522515	124522678	.	+	.	transcript_	MSTRG.14293.1	gene_id	MSTRG.14293;
14	StringTie	transcrip	125069275	125073822	.	+	.	transcript_	MSTRG.14330.1	gene_id	MSTRG.14330;
14	StringTie	exon	125069275	125069308	.	+	.	transcript_	MSTRG.14330.1	gene_id	MSTRG.14330;
14	StringTie	exon	125072896	125073822	.	+	.	transcript_	MSTRG.14330.1	gene_id	MSTRG.14330;
14	StringTie	transcrip	125107441	125122382	.	+	.	transcript_	MSTRG.14331.1	gene_id	MSTRG.14331;
14	StringTie	exon	125107441	125108221	.	+	.	transcript_	MSTRG.14331.1	gene_id	MSTRG.14331;
14	StringTie	exon	125120090	125120177	.	+	.	transcript_	MSTRG.14331.1	gene_id	MSTRG.14331;
14	StringTie	exon	125122135	125122382	.	+	.	transcript_	MSTRG.14331.1	gene_id	MSTRG.14331;
14	StringTie	transcrip	125120971	125122381	.	+	.	transcript_	MSTRG.14331.2	gene_id	MSTRG.14331;
14	StringTie	exon	125120971	125121326	.	+	.	transcript_	MSTRG.14331.2	gene_id	MSTRG.14331;
14	StringTie	exon	125122135	125122381	.	+	.	transcript_	MSTRG.14331.2	gene_id	MSTRG.14331;
14	StringTie	transcrip	130675216	130675834	.	+	.	transcript_	MSTRG.14392.1	gene_id	MSTRG.14392;
14	StringTie	exon	130675216	130675477	.	+	.	transcript_	MSTRG.14392.1	gene_id	MSTRG.14392;
14	StringTie	exon	130675797	130675834	.	+	.	transcript_	MSTRG.14392.1	gene_id	MSTRG.14392;

14	StringTie	transcrip	131586326	131601512	.	+	.	transcript_	MSTRG.14397.1	gene_id	MSTRG.14397;
14	StringTie	exon	131586326	131586402	.	+	.	transcript_	MSTRG.14397.1	gene_id	MSTRG.14397;
14	StringTie	exon	131593384	131593624	.	+	.	transcript_	MSTRG.14397.1	gene_id	MSTRG.14397;
14	StringTie	exon	131601449	131601512	.	+	.	transcript_	MSTRG.14397.1	gene_id	MSTRG.14397;
14	StringTie	transcrip	131593329	131593880	.	+	.	transcript_	MSTRG.14397.2	gene_id	MSTRG.14397;
14	StringTie	exon	131593329	131593624	.	+	.	transcript_	MSTRG.14397.2	gene_id	MSTRG.14397;
14	StringTie	exon	131593803	131593880	.	+	.	transcript_	MSTRG.14397.2	gene_id	MSTRG.14397;
14	StringTie	transcrip	138846008	138849346	.	+	.	transcript_	MSTRG.14470.1	gene_id	MSTRG.14470;
14	StringTie	exon	138846008	138846087	.	+	.	transcript_	MSTRG.14470.1	gene_id	MSTRG.14470;
14	StringTie	exon	138846697	138846809	.	+	.	transcript_	MSTRG.14470.1	gene_id	MSTRG.14470;
14	StringTie	exon	138849286	138849346	.	+	.	transcript_	MSTRG.14470.1	gene_id	MSTRG.14470;
14	StringTie	transcrip	140372113	140373753	.	+	.	transcript_	MSTRG.14472.1	gene_id	MSTRG.14472;
14	StringTie	exon	140372113	140372157	.	+	.	transcript_	MSTRG.14472.1	gene_id	MSTRG.14472;
14	StringTie	exon	140373076	140373753	.	+	.	transcript_	MSTRG.14472.1	gene_id	MSTRG.14472;
14	StringTie	transcrip	141290979	141292222	.	+	.	transcript_	MSTRG.14496.1	gene_id	MSTRG.14496;
14	StringTie	exon	141290979	141291387	.	+	.	transcript_	MSTRG.14496.1	gene_id	MSTRG.14496;
14	StringTie	exon	141292012	141292222	.	+	.	transcript_	MSTRG.14496.1	gene_id	MSTRG.14496;
14	StringTie	transcrip	925351	926987	.	-	.	transcript_	MSTRG.12093.1	gene_id	MSTRG.12093;
14	StringTie	exon	925351	925406	.	-	.	transcript_	MSTRG.12093.1	gene_id	MSTRG.12093;
14	StringTie	exon	926803	926987	.	-	.	transcript_	MSTRG.12093.1	gene_id	MSTRG.12093;
14	StringTie	transcrip	3917679	3928965	.	-	.	transcript_	MSTRG.12125.1	gene_id	MSTRG.12125;
14	StringTie	exon	3917679	3917715	.	-	.	transcript_	MSTRG.12125.1	gene_id	MSTRG.12125;
14	StringTie	exon	3919614	3919973	.	-	.	transcript_	MSTRG.12125.1	gene_id	MSTRG.12125;
14	StringTie	exon	3928879	3928965	.	-	.	transcript_	MSTRG.12125.1	gene_id	MSTRG.12125;
14	StringTie	transcrip	4126158	4127970	.	-	.	transcript_	MSTRG.12130.1	gene_id	MSTRG.12130;
14	StringTie	exon	4126158	4126268	.	-	.	transcript_	MSTRG.12130.1	gene_id	MSTRG.12130;
14	StringTie	exon	4127860	4127970	.	-	.	transcript_	MSTRG.12130.1	gene_id	MSTRG.12130;
14	StringTie	transcrip	6845435	6849745	.	-	.	transcript_	MSTRG.12163.1	gene_id	MSTRG.12163;
14	StringTie	exon	6845435	6845733	.	-	.	transcript_	MSTRG.12163.1	gene_id	MSTRG.12163;
14	StringTie	exon	6849165	6849745	.	-	.	transcript_	MSTRG.12163.1	gene_id	MSTRG.12163;
14	StringTie	transcrip	6849216	6849707	.	-	.	transcript_	MSTRG.12163.2	gene_id	MSTRG.12163;
14	StringTie	exon	6849216	6849258	.	-	.	transcript_	MSTRG.12163.2	gene_id	MSTRG.12163;
14	StringTie	exon	6849546	6849707	.	-	.	transcript_	MSTRG.12163.2	gene_id	MSTRG.12163;
14	StringTie	transcrip	7365667	7371370	.	-	.	transcript_	MSTRG.12194.2	gene_id	MSTRG.12194;
14	StringTie	exon	7365667	7366524	.	-	.	transcript_	MSTRG.12194.2	gene_id	MSTRG.12194;

14	StringTie	exon	7370890	7371370	.	-	.	transcript_	MSTRG.12194.2	gene_id	MSTRG.12194;
14	StringTie	transcrip	30319534	30320705	.	-	.	transcript_	MSTRG.12532.1	gene_id	MSTRG.12532;
14	StringTie	exon	30319534	30320466	.	-	.	transcript_	MSTRG.12532.1	gene_id	MSTRG.12532;
14	StringTie	exon	30320597	30320705	.	-	.	transcript_	MSTRG.12532.1	gene_id	MSTRG.12532;
14	StringTie	transcrip	30998214	31010872	.	-	.	transcript_	MSTRG.12543.1	gene_id	MSTRG.12543;
14	StringTie	exon	30998214	30998470	.	-	.	transcript_	MSTRG.12543.1	gene_id	MSTRG.12543;
14	StringTie	exon	31009316	31010872	.	-	.	transcript_	MSTRG.12543.1	gene_id	MSTRG.12543;
14	StringTie	transcrip	33901526	33915252	.	-	.	transcript_	MSTRG.12626.1	gene_id	MSTRG.12626;
14	StringTie	exon	33901526	33901817	.	-	.	transcript_	MSTRG.12626.1	gene_id	MSTRG.12626;
14	StringTie	exon	33915230	33915252	.	-	.	transcript_	MSTRG.12626.1	gene_id	MSTRG.12626;
14	StringTie	transcrip	37767796	37771105	.	-	.	transcript_	MSTRG.12688.1	gene_id	MSTRG.12688;
14	StringTie	exon	37767796	37768095	.	-	.	transcript_	MSTRG.12688.1	gene_id	MSTRG.12688;
14	StringTie	exon	37771052	37771105	.	-	.	transcript_	MSTRG.12688.1	gene_id	MSTRG.12688;
14	StringTie	transcrip	40927236	40943719	.	-	.	transcript_	MSTRG.12769.1	gene_id	MSTRG.12769;
14	StringTie	exon	40927236	40928010	.	-	.	transcript_	MSTRG.12769.1	gene_id	MSTRG.12769;
14	StringTie	exon	40928864	40928935	.	-	.	transcript_	MSTRG.12769.1	gene_id	MSTRG.12769;
14	StringTie	exon	40931040	40931064	.	-	.	transcript_	MSTRG.12769.1	gene_id	MSTRG.12769;
14	StringTie	exon	40932319	40932389	.	-	.	transcript_	MSTRG.12769.1	gene_id	MSTRG.12769;
14	StringTie	exon	40933436	40933613	.	-	.	transcript_	MSTRG.12769.1	gene_id	MSTRG.12769;
14	StringTie	exon	40943692	40943719	.	-	.	transcript_	MSTRG.12769.1	gene_id	MSTRG.12769;
14	StringTie	transcrip	49001079	49004417	.	-	.	transcript_	MSTRG.12945.2	gene_id	MSTRG.12945;
14	StringTie	exon	49001079	49001189	.	-	.	transcript_	MSTRG.12945.2	gene_id	MSTRG.12945;
14	StringTie	exon	49004308	49004417	.	-	.	transcript_	MSTRG.12945.2	gene_id	MSTRG.12945;
14	StringTie	transcrip	49001079	49007534	.	-	.	transcript_	MSTRG.12945.1	gene_id	MSTRG.12945;
14	StringTie	exon	49001079	49001189	.	-	.	transcript_	MSTRG.12945.1	gene_id	MSTRG.12945;
14	StringTie	exon	49007424	49007534	.	-	.	transcript_	MSTRG.12945.1	gene_id	MSTRG.12945;
14	StringTie	transcrip	50690427	50691003	.	-	.	transcript_	MSTRG.13002.1	gene_id	MSTRG.13002;
14	StringTie	exon	50690427	50690479	.	-	.	transcript_	MSTRG.13002.1	gene_id	MSTRG.13002;
14	StringTie	exon	50690594	50690763	.	-	.	transcript_	MSTRG.13002.1	gene_id	MSTRG.13002;
14	StringTie	exon	50690894	50691003	.	-	.	transcript_	MSTRG.13002.1	gene_id	MSTRG.13002;
14	StringTie	transcrip	50762070	50773772	.	-	.	transcript_	MSTRG.13038.1	gene_id	MSTRG.13038;
14	StringTie	exon	50762070	50763634	.	-	.	transcript_	MSTRG.13038.1	gene_id	MSTRG.13038;
14	StringTie	exon	50773060	50773130	.	-	.	transcript_	MSTRG.13038.1	gene_id	MSTRG.13038;
14	StringTie	exon	50773404	50773772	.	-	.	transcript_	MSTRG.13038.1	gene_id	MSTRG.13038;
14	StringTie	transcrip	50998255	51002950	.	-	.	transcript_	MSTRG.13048.6	gene_id	MSTRG.13048;

14	StringTie	exon	50998255	50998685	.	-	.	transcript_	MSTRG.13048.6	gene_id	MSTRG.13048;
14	StringTie	exon	50999626	50999848	.	-	.	transcript_	MSTRG.13048.6	gene_id	MSTRG.13048;
14	StringTie	exon	51002775	51002950	.	-	.	transcript_	MSTRG.13048.6	gene_id	MSTRG.13048;
14	StringTie	transcrip	51349877	51352953	.	-	.	transcript_	MSTRG.13007.1	gene_id	MSTRG.13007;
14	StringTie	exon	51349877	51350992	.	-	.	transcript_	MSTRG.13007.1	gene_id	MSTRG.13007;
14	StringTie	exon	51352506	51352953	.	-	.	transcript_	MSTRG.13007.1	gene_id	MSTRG.13007;
14	StringTie	transcrip	60415332	60415679	.	-	.	transcript_	MSTRG.13186.7	gene_id	MSTRG.13186;
14	StringTie	exon	60415332	60415448	.	-	.	transcript_	MSTRG.13186.7	gene_id	MSTRG.13186;
14	StringTie	exon	60415492	60415679	.	-	.	transcript_	MSTRG.13186.7	gene_id	MSTRG.13186;
14	StringTie	transcrip	60415332	60415679	.	-	.	transcript_	MSTRG.13186.6	gene_id	MSTRG.13186;
14	StringTie	exon	60415332	60415472	.	-	.	transcript_	MSTRG.13186.6	gene_id	MSTRG.13186;
14	StringTie	exon	60415590	60415679	.	-	.	transcript_	MSTRG.13186.6	gene_id	MSTRG.13186;
14	StringTie	transcrip	60911043	60938894	.	-	.	transcript_	MSTRG.13205.2	gene_id	MSTRG.13205;
14	StringTie	exon	60911043	60912810	.	-	.	transcript_	MSTRG.13205.2	gene_id	MSTRG.13205;
14	StringTie	exon	60924849	60924944	.	-	.	transcript_	MSTRG.13205.2	gene_id	MSTRG.13205;
14	StringTie	exon	60938383	60938894	.	-	.	transcript_	MSTRG.13205.2	gene_id	MSTRG.13205;
14	StringTie	transcrip	60936516	60938866	.	-	.	transcript_	MSTRG.13205.4	gene_id	MSTRG.13205;
14	StringTie	exon	60936516	60936731	.	-	.	transcript_	MSTRG.13205.4	gene_id	MSTRG.13205;
14	StringTie	exon	60938383	60938866	.	-	.	transcript_	MSTRG.13205.4	gene_id	MSTRG.13205;
14	StringTie	transcrip	61684405	61688596	.	-	.	transcript_	MSTRG.13220.1	gene_id	MSTRG.13220;
14	StringTie	exon	61684405	61685572	.	-	.	transcript_	MSTRG.13220.1	gene_id	MSTRG.13220;
14	StringTie	exon	61687782	61687993	.	-	.	transcript_	MSTRG.13220.1	gene_id	MSTRG.13220;
14	StringTie	exon	61688116	61688596	.	-	.	transcript_	MSTRG.13220.1	gene_id	MSTRG.13220;
14	StringTie	transcrip	61686164	61688565	.	-	.	transcript_	MSTRG.13220.2	gene_id	MSTRG.13220;
14	StringTie	exon	61686164	61687993	.	-	.	transcript_	MSTRG.13220.2	gene_id	MSTRG.13220;
14	StringTie	exon	61688317	61688565	.	-	.	transcript_	MSTRG.13220.2	gene_id	MSTRG.13220;
14	StringTie	transcrip	77911698	77923219	.	-	.	transcript_	MSTRG.13498.1	gene_id	MSTRG.13498;
14	StringTie	exon	77911698	77919290	.	-	.	transcript_	MSTRG.13498.1	gene_id	MSTRG.13498;
14	StringTie	exon	77922634	77923219	.	-	.	transcript_	MSTRG.13498.1	gene_id	MSTRG.13498;
14	StringTie	transcrip	77935645	77936256	.	-	.	transcript_	MSTRG.13502.1	gene_id	MSTRG.13502;
14	StringTie	exon	77935645	77935714	.	-	.	transcript_	MSTRG.13502.1	gene_id	MSTRG.13502;
14	StringTie	exon	77936041	77936256	.	-	.	transcript_	MSTRG.13502.1	gene_id	MSTRG.13502;
14	StringTie	transcrip	78543900	78564568	.	-	.	transcript_	MSTRG.13552.1	gene_id	MSTRG.13552;
14	StringTie	exon	78543900	78544004	.	-	.	transcript_	MSTRG.13552.1	gene_id	MSTRG.13552;
14	StringTie	exon	78564472	78564568	.	-	.	transcript_	MSTRG.13552.1	gene_id	MSTRG.13552;

14	StringTie	transcrip	88431289	88432647	.	-	.	transcript_	MSTRG.13683.1	gene_id	MSTRG.13683;
14	StringTie	exon	88431289	88431533	.	-	.	transcript_	MSTRG.13683.1	gene_id	MSTRG.13683;
14	StringTie	exon	88432470	88432647	.	-	.	transcript_	MSTRG.13683.1	gene_id	MSTRG.13683;
14	StringTie	transcrip	89881704	89885731	.	-	.	transcript_	MSTRG.13701.1	gene_id	MSTRG.13701;
14	StringTie	exon	89881704	89881862	.	-	.	transcript_	MSTRG.13701.1	gene_id	MSTRG.13701;
14	StringTie	exon	89885575	89885731	.	-	.	transcript_	MSTRG.13701.1	gene_id	MSTRG.13701;
14	StringTie	transcrip	99298317	99454743	.	-	.	transcript_	MSTRG.13811.8	gene_id	MSTRG.13811;
14	StringTie	exon	99298317	99298358	.	-	.	transcript_	MSTRG.13811.8	gene_id	MSTRG.13811;
14	StringTie	exon	99349892	99349974	.	-	.	transcript_	MSTRG.13811.8	gene_id	MSTRG.13811;
14	StringTie	exon	99454410	99454743	.	-	.	transcript_	MSTRG.13811.8	gene_id	MSTRG.13811;
14	StringTie	transcrip	106696815	106700176	.	-	.	transcript_	MSTRG.13970.3	gene_id	MSTRG.13970;
14	StringTie	exon	106696815	106697172	.	-	.	transcript_	MSTRG.13970.3	gene_id	MSTRG.13970;
14	StringTie	exon	106698840	106698918	.	-	.	transcript_	MSTRG.13970.3	gene_id	MSTRG.13970;
14	StringTie	exon	106699909	106700176	.	-	.	transcript_	MSTRG.13970.3	gene_id	MSTRG.13970;
14	StringTie	transcrip	107306378	107396150	.	-	.	transcript_	MSTRG.13999.1	gene_id	MSTRG.13999;
14	StringTie	exon	107306378	107306406	.	-	.	transcript_	MSTRG.13999.1	gene_id	MSTRG.13999;
14	StringTie	exon	107389015	107396150	.	-	.	transcript_	MSTRG.13999.1	gene_id	MSTRG.13999;
14	StringTie	transcrip	109065003	109119816	.	-	.	transcript_	MSTRG.14048.1	gene_id	MSTRG.14048;
14	StringTie	exon	109065003	109065097	.	-	.	transcript_	MSTRG.14048.1	gene_id	MSTRG.14048;
14	StringTie	exon	109119113	109119816	.	-	.	transcript_	MSTRG.14048.1	gene_id	MSTRG.14048;
14	StringTie	transcrip	109065061	109119773	.	-	.	transcript_	MSTRG.14048.2	gene_id	MSTRG.14048;
14	StringTie	exon	109065061	109065097	.	-	.	transcript_	MSTRG.14048.2	gene_id	MSTRG.14048;
14	StringTie	exon	109117103	109119773	.	-	.	transcript_	MSTRG.14048.2	gene_id	MSTRG.14048;
14	StringTie	transcrip	109092090	109119764	.	-	.	transcript_	MSTRG.14048.3	gene_id	MSTRG.14048;
14	StringTie	exon	109092090	109092374	.	-	.	transcript_	MSTRG.14048.3	gene_id	MSTRG.14048;
14	StringTie	exon	109119113	109119764	.	-	.	transcript_	MSTRG.14048.3	gene_id	MSTRG.14048;
14	StringTie	transcrip	109092348	109119773	.	-	.	transcript_	MSTRG.14048.4	gene_id	MSTRG.14048;
14	StringTie	exon	109092348	109092374	.	-	.	transcript_	MSTRG.14048.4	gene_id	MSTRG.14048;
14	StringTie	exon	109117103	109119773	.	-	.	transcript_	MSTRG.14048.4	gene_id	MSTRG.14048;
14	StringTie	transcrip	111470803	111477982	.	-	.	transcript_	MSTRG.14097.1	gene_id	MSTRG.14097;
14	StringTie	exon	111470803	111470938	.	-	.	transcript_	MSTRG.14097.1	gene_id	MSTRG.14097;
14	StringTie	exon	111477872	111477982	.	-	.	transcript_	MSTRG.14097.1	gene_id	MSTRG.14097;
14	StringTie	transcrip	112120679	112122120	.	-	.	transcript_	MSTRG.14115.1	gene_id	MSTRG.14115;
14	StringTie	exon	112120679	112120882	.	-	.	transcript_	MSTRG.14115.1	gene_id	MSTRG.14115;
14	StringTie	exon	112122014	112122120	.	-	.	transcript_	MSTRG.14115.1	gene_id	MSTRG.14115;

14	StringTie	transcrip	121135903	121140617	.	-	.	transcript_	MSTRG.14258.1	gene_id	MSTRG.14258;
14	StringTie	exon	121135903	121139012	.	-	.	transcript_	MSTRG.14258.1	gene_id	MSTRG.14258;
14	StringTie	exon	121139882	121140617	.	-	.	transcript_	MSTRG.14258.1	gene_id	MSTRG.14258;
14	StringTie	transcrip	121137764	121140693	.	-	.	transcript_	MSTRG.14258.2	gene_id	MSTRG.14258;
14	StringTie	exon	121137764	121139012	.	-	.	transcript_	MSTRG.14258.2	gene_id	MSTRG.14258;
14	StringTie	exon	121140021	121140693	.	-	.	transcript_	MSTRG.14258.2	gene_id	MSTRG.14258;
14	StringTie	transcrip	121269520	121285144	.	-	.	transcript_	MSTRG.14266.1	gene_id	MSTRG.14266;
14	StringTie	exon	121269520	121269646	.	-	.	transcript_	MSTRG.14266.1	gene_id	MSTRG.14266;
14	StringTie	exon	121271651	121271778	.	-	.	transcript_	MSTRG.14266.1	gene_id	MSTRG.14266;
14	StringTie	exon	121284970	121285144	.	-	.	transcript_	MSTRG.14266.1	gene_id	MSTRG.14266;
14	StringTie	transcrip	123424878	123426792	.	-	.	transcript_	MSTRG.14281.1	gene_id	MSTRG.14281;
14	StringTie	exon	123424878	123425574	.	-	.	transcript_	MSTRG.14281.1	gene_id	MSTRG.14281;
14	StringTie	exon	123426707	123426792	.	-	.	transcript_	MSTRG.14281.1	gene_id	MSTRG.14281;
14	StringTie	transcrip	124744074	124757578	.	-	.	transcript_	MSTRG.14298.1	gene_id	MSTRG.14298;
14	StringTie	exon	124744074	124744374	.	-	.	transcript_	MSTRG.14298.1	gene_id	MSTRG.14298;
14	StringTie	exon	124757437	124757578	.	-	.	transcript_	MSTRG.14298.1	gene_id	MSTRG.14298;
14	StringTie	transcrip	125122106	125124015	.	-	.	transcript_	MSTRG.14332.1	gene_id	MSTRG.14332;
14	StringTie	exon	125122106	125122353	.	-	.	transcript_	MSTRG.14332.1	gene_id	MSTRG.14332;
14	StringTie	exon	125123880	125124015	.	-	.	transcript_	MSTRG.14332.1	gene_id	MSTRG.14332;
14	StringTie	transcrip	134142790	134144193	.	-	.	transcript_	MSTRG.14426.1	gene_id	MSTRG.14426;
14	StringTie	exon	134142790	134143100	.	-	.	transcript_	MSTRG.14426.1	gene_id	MSTRG.14426;
14	StringTie	exon	134144173	134144193	.	-	.	transcript_	MSTRG.14426.1	gene_id	MSTRG.14426;
14	StringTie	transcrip	135085392	135086094	.	-	.	transcript_	MSTRG.14437.1	gene_id	MSTRG.14437;
14	StringTie	exon	135085392	135085497	.	-	.	transcript_	MSTRG.14437.1	gene_id	MSTRG.14437;
14	StringTie	exon	135085985	135086094	.	-	.	transcript_	MSTRG.14437.1	gene_id	MSTRG.14437;
14	StringTie	transcrip	138495317	138501675	.	-	.	transcript_	MSTRG.14459.1	gene_id	MSTRG.14459;
14	StringTie	exon	138495317	138501675	.	-	.	transcript_	MSTRG.14459.1	gene_id	MSTRG.14459;
14	StringTie	transcrip	138500026	138501738	.	-	.	transcript_	MSTRG.14459.2	gene_id	MSTRG.14459;
14	StringTie	exon	138500026	138501246	.	-	.	transcript_	MSTRG.14459.2	gene_id	MSTRG.14459;
14	StringTie	exon	138501378	138501738	.	-	.	transcript_	MSTRG.14459.2	gene_id	MSTRG.14459;
14	StringTie	transcrip	140633130	140637849	.	-	.	transcript_	MSTRG.14480.1	gene_id	MSTRG.14480;
14	StringTie	exon	140633130	140633156	.	-	.	transcript_	MSTRG.14480.1	gene_id	MSTRG.14480;
14	StringTie	exon	140637474	140637849	.	-	.	transcript_	MSTRG.14480.1	gene_id	MSTRG.14480;
15	StringTie	transcrip	6600701	6607813	.	+	.	transcript_	MSTRG.14564.1	gene_id	MSTRG.14564;
15	StringTie	exon	6600701	6600827	.	+	.	transcript_	MSTRG.14564.1	gene_id	MSTRG.14564;

15	StringTie	exon	6607096	6607813	.	+	.	transcript_	MSTRG.14564.[1]	gene_id	MSTRG.14564;
15	StringTie	transcrip	6685434	6840986	.	+	.	transcript_	MSTRG.14573.[1]	gene_id	MSTRG.14573;
15	StringTie	exon	6685434	6685471	.	+	.	transcript_	MSTRG.14573.[1]	gene_id	MSTRG.14573;
15	StringTie	exon	6711192	6711226	.	+	.	transcript_	MSTRG.14573.[1]	gene_id	MSTRG.14573;
15	StringTie	exon	6711253	6711395	.	+	.	transcript_	MSTRG.14573.[1]	gene_id	MSTRG.14573;
15	StringTie	exon	6838496	6840986	.	+	.	transcript_	MSTRG.14573.[1]	gene_id	MSTRG.14573;
15	StringTie	transcrip	6706210	6840865	.	+	.	transcript_	MSTRG.14573.[3]	gene_id	MSTRG.14573;
15	StringTie	exon	6706210	6706326	.	+	.	transcript_	MSTRG.14573.[3]	gene_id	MSTRG.14573;
15	StringTie	exon	6711192	6711226	.	+	.	transcript_	MSTRG.14573.[3]	gene_id	MSTRG.14573;
15	StringTie	exon	6711253	6711395	.	+	.	transcript_	MSTRG.14573.[3]	gene_id	MSTRG.14573;
15	StringTie	exon	6838496	6840865	.	+	.	transcript_	MSTRG.14573.[3]	gene_id	MSTRG.14573;
15	StringTie	transcrip	6706210	6840865	.	+	.	transcript_	MSTRG.14573.[2]	gene_id	MSTRG.14573;
15	StringTie	exon	6706210	6706326	.	+	.	transcript_	MSTRG.14573.[2]	gene_id	MSTRG.14573;
15	StringTie	exon	6838496	6840865	.	+	.	transcript_	MSTRG.14573.[2]	gene_id	MSTRG.14573;
15	StringTie	transcrip	6706235	6840986	.	+	.	transcript_	MSTRG.14573.[4]	gene_id	MSTRG.14573;
15	StringTie	exon	6706235	6706326	.	+	.	transcript_	MSTRG.14573.[4]	gene_id	MSTRG.14573;
15	StringTie	exon	6711192	6711395	.	+	.	transcript_	MSTRG.14573.[4]	gene_id	MSTRG.14573;
15	StringTie	exon	6838496	6840986	.	+	.	transcript_	MSTRG.14573.[4]	gene_id	MSTRG.14573;
15	StringTie	transcrip	6711189	6897580	.	+	.	transcript_	MSTRG.14573.[5]	gene_id	MSTRG.14573;
15	StringTie	exon	6711189	6711395	.	+	.	transcript_	MSTRG.14573.[5]	gene_id	MSTRG.14573;
15	StringTie	exon	6897502	6897580	.	+	.	transcript_	MSTRG.14573.[5]	gene_id	MSTRG.14573;
15	StringTie	transcrip	6996289	7035999	.	+	.	transcript_	MSTRG.14578.[1]	gene_id	MSTRG.14578;
15	StringTie	exon	6996289	6996472	.	+	.	transcript_	MSTRG.14578.[1]	gene_id	MSTRG.14578;
15	StringTie	exon	6996618	6996683	.	+	.	transcript_	MSTRG.14578.[1]	gene_id	MSTRG.14578;
15	StringTie	exon	7034427	7035999	.	+	.	transcript_	MSTRG.14578.[1]	gene_id	MSTRG.14578;
15	StringTie	transcrip	6996319	7019003	.	+	.	transcript_	MSTRG.14578.[2]	gene_id	MSTRG.14578;
15	StringTie	exon	6996319	6996472	.	+	.	transcript_	MSTRG.14578.[2]	gene_id	MSTRG.14578;
15	StringTie	exon	6996618	6996683	.	+	.	transcript_	MSTRG.14578.[2]	gene_id	MSTRG.14578;
15	StringTie	exon	7018572	7019003	.	+	.	transcript_	MSTRG.14578.[2]	gene_id	MSTRG.14578;
15	StringTie	transcrip	7685379	7699992	.	+	.	transcript_	MSTRG.14604.[1]	gene_id	MSTRG.14604;
15	StringTie	exon	7685379	7685453	.	+	.	transcript_	MSTRG.14604.[1]	gene_id	MSTRG.14604;
15	StringTie	exon	7698343	7699992	.	+	.	transcript_	MSTRG.14604.[1]	gene_id	MSTRG.14604;
15	StringTie	transcrip	7685989	7700005	.	+	.	transcript_	MSTRG.14604.[3]	gene_id	MSTRG.14604;
15	StringTie	exon	7685989	7686087	.	+	.	transcript_	MSTRG.14604.[3]	gene_id	MSTRG.14604;
15	StringTie	exon	7698343	7700005	.	+	.	transcript_	MSTRG.14604.[3]	gene_id	MSTRG.14604;

15	StringTie	transcrip	7686500	7699992	.	+	.	transcript_	MSTRG.14604.7	gene_id	MSTRG.14604;
15	StringTie	exon	7686500	7687086	.	+	.	transcript_	MSTRG.14604.7	gene_id	MSTRG.14604;
15	StringTie	exon	7698343	7699992	.	+	.	transcript_	MSTRG.14604.7	gene_id	MSTRG.14604;
15	StringTie	transcrip	7686500	7700204	.	+	.	transcript_	MSTRG.14604.6	gene_id	MSTRG.14604;
15	StringTie	exon	7686500	7687114	.	+	.	transcript_	MSTRG.14604.6	gene_id	MSTRG.14604;
15	StringTie	exon	7698343	7700204	.	+	.	transcript_	MSTRG.14604.6	gene_id	MSTRG.14604;
15	StringTie	transcrip	31318403	31320372	.	+	.	transcript_	MSTRG.14968.1	gene_id	MSTRG.14968;
15	StringTie	exon	31318403	31319369	.	+	.	transcript_	MSTRG.14968.1	gene_id	MSTRG.14968;
15	StringTie	exon	31320035	31320372	.	+	.	transcript_	MSTRG.14968.1	gene_id	MSTRG.14968;
15	StringTie	transcrip	31502979	31508308	.	+	.	transcript_	MSTRG.14970.3	gene_id	MSTRG.14970;
15	StringTie	exon	31502979	31503190	.	+	.	transcript_	MSTRG.14970.3	gene_id	MSTRG.14970;
15	StringTie	exon	31507359	31508308	.	+	.	transcript_	MSTRG.14970.3	gene_id	MSTRG.14970;
15	StringTie	transcrip	31502979	31508501	.	+	.	transcript_	MSTRG.14970.2	gene_id	MSTRG.14970;
15	StringTie	exon	31502979	31503190	.	+	.	transcript_	MSTRG.14970.2	gene_id	MSTRG.14970;
15	StringTie	exon	31504973	31505034	.	+	.	transcript_	MSTRG.14970.2	gene_id	MSTRG.14970;
15	StringTie	exon	31507359	31508501	.	+	.	transcript_	MSTRG.14970.2	gene_id	MSTRG.14970;
15	StringTie	transcrip	31502979	31508501	.	+	.	transcript_	MSTRG.14970.1	gene_id	MSTRG.14970;
15	StringTie	exon	31502979	31503168	.	+	.	transcript_	MSTRG.14970.1	gene_id	MSTRG.14970;
15	StringTie	exon	31504973	31505034	.	+	.	transcript_	MSTRG.14970.1	gene_id	MSTRG.14970;
15	StringTie	exon	31507359	31508501	.	+	.	transcript_	MSTRG.14970.1	gene_id	MSTRG.14970;
15	StringTie	transcrip	31502993	31508462	.	+	.	transcript_	MSTRG.14970.4	gene_id	MSTRG.14970;
15	StringTie	exon	31502993	31503362	.	+	.	transcript_	MSTRG.14970.4	gene_id	MSTRG.14970;
15	StringTie	exon	31504973	31505034	.	+	.	transcript_	MSTRG.14970.4	gene_id	MSTRG.14970;
15	StringTie	exon	31507359	31508462	.	+	.	transcript_	MSTRG.14970.4	gene_id	MSTRG.14970;
15	StringTie	transcrip	31502995	31508308	.	+	.	transcript_	MSTRG.14970.5	gene_id	MSTRG.14970;
15	StringTie	exon	31502995	31503168	.	+	.	transcript_	MSTRG.14970.5	gene_id	MSTRG.14970;
15	StringTie	exon	31507359	31508308	.	+	.	transcript_	MSTRG.14970.5	gene_id	MSTRG.14970;
15	StringTie	transcrip	34362669	34375105	.	+	.	transcript_	MSTRG.15019.1	gene_id	MSTRG.15019;
15	StringTie	exon	34362669	34362706	.	+	.	transcript_	MSTRG.15019.1	gene_id	MSTRG.15019;
15	StringTie	exon	34362801	34363092	.	+	.	transcript_	MSTRG.15019.1	gene_id	MSTRG.15019;
15	StringTie	exon	34363179	34363289	.	+	.	transcript_	MSTRG.15019.1	gene_id	MSTRG.15019;
15	StringTie	exon	34374656	34374726	.	+	.	transcript_	MSTRG.15019.1	gene_id	MSTRG.15019;
15	StringTie	exon	34374823	34375105	.	+	.	transcript_	MSTRG.15019.1	gene_id	MSTRG.15019;
15	StringTie	transcrip	47694065	47694771	.	+	.	transcript_	MSTRG.15186.1	gene_id	MSTRG.15186;
15	StringTie	exon	47694065	47694105	.	+	.	transcript_	MSTRG.15186.1	gene_id	MSTRG.15186;

15	StringTie	exon	47694331	47694771	.	+	.	transcript_	MSTRG.15186.1	gene_id	MSTRG.15186;
15	StringTie	transcrip	47809230	47811781	.	+	.	transcript_	MSTRG.15190.1	gene_id	MSTRG.15190;
15	StringTie	exon	47809230	47809300	.	+	.	transcript_	MSTRG.15190.1	gene_id	MSTRG.15190;
15	StringTie	exon	47811053	47811781	.	+	.	transcript_	MSTRG.15190.1	gene_id	MSTRG.15190;
15	StringTie	transcrip	52199842	52232611	.	+	.	transcript_	MSTRG.15223.1	gene_id	MSTRG.15223;
15	StringTie	exon	52199842	52199891	.	+	.	transcript_	MSTRG.15223.1	gene_id	MSTRG.15223;
15	StringTie	exon	52218528	52218660	.	+	.	transcript_	MSTRG.15223.1	gene_id	MSTRG.15223;
15	StringTie	exon	52232524	52232611	.	+	.	transcript_	MSTRG.15223.1	gene_id	MSTRG.15223;
15	StringTie	transcrip	54086532	54093809	.	+	.	transcript_	MSTRG.15256.1	gene_id	MSTRG.15256;
15	StringTie	exon	54086532	54086704	.	+	.	transcript_	MSTRG.15256.1	gene_id	MSTRG.15256;
15	StringTie	exon	54093677	54093809	.	+	.	transcript_	MSTRG.15256.1	gene_id	MSTRG.15256;
15	StringTie	transcrip	58992400	59045286	.	+	.	transcript_	MSTRG.15319.1	gene_id	MSTRG.15319;
15	StringTie	exon	58992400	58992447	.	+	.	transcript_	MSTRG.15319.1	gene_id	MSTRG.15319;
15	StringTie	exon	59045135	59045286	.	+	.	transcript_	MSTRG.15319.1	gene_id	MSTRG.15319;
15	StringTie	transcrip	58992410	59045295	.	+	.	transcript_	MSTRG.15319.2	gene_id	MSTRG.15319;
15	StringTie	exon	58992410	58992447	.	+	.	transcript_	MSTRG.15319.2	gene_id	MSTRG.15319;
15	StringTie	exon	59045132	59045295	.	+	.	transcript_	MSTRG.15319.2	gene_id	MSTRG.15319;
15	StringTie	transcrip	64599512	64763746	.	+	.	transcript_	MSTRG.15392.1	gene_id	MSTRG.15392;
15	StringTie	exon	64599512	64599595	.	+	.	transcript_	MSTRG.15392.1	gene_id	MSTRG.15392;
15	StringTie	exon	64763319	64763746	.	+	.	transcript_	MSTRG.15392.1	gene_id	MSTRG.15392;
15	StringTie	transcrip	65824543	65859228	.	+	.	transcript_	MSTRG.15446.1	gene_id	MSTRG.15446;
15	StringTie	exon	65824543	65824641	.	+	.	transcript_	MSTRG.15446.1	gene_id	MSTRG.15446;
15	StringTie	exon	65845468	65845618	.	+	.	transcript_	MSTRG.15446.1	gene_id	MSTRG.15446;
15	StringTie	exon	65857252	65859228	.	+	.	transcript_	MSTRG.15446.1	gene_id	MSTRG.15446;
15	StringTie	transcrip	66571061	66589276	.	+	.	transcript_	MSTRG.15476.1	gene_id	MSTRG.15476;
15	StringTie	exon	66571061	66571128	.	+	.	transcript_	MSTRG.15476.1	gene_id	MSTRG.15476;
15	StringTie	exon	66587749	66589276	.	+	.	transcript_	MSTRG.15476.1	gene_id	MSTRG.15476;
15	StringTie	transcrip	67170285	67178136	.	+	.	transcript_	MSTRG.15463.1	gene_id	MSTRG.15463;
15	StringTie	exon	67170285	67171710	.	+	.	transcript_	MSTRG.15463.1	gene_id	MSTRG.15463;
15	StringTie	exon	67176718	67178136	.	+	.	transcript_	MSTRG.15463.1	gene_id	MSTRG.15463;
15	StringTie	transcrip	67192572	67194047	.	+	.	transcript_	MSTRG.15470.1	gene_id	MSTRG.15470;
15	StringTie	exon	67192572	67192878	.	+	.	transcript_	MSTRG.15470.1	gene_id	MSTRG.15470;
15	StringTie	exon	67192940	67194047	.	+	.	transcript_	MSTRG.15470.1	gene_id	MSTRG.15470;
15	StringTie	transcrip	72445992	72460562	.	+	.	transcript_	MSTRG.15535.1	gene_id	MSTRG.15535;
15	StringTie	exon	72445992	72446242	.	+	.	transcript_	MSTRG.15535.1	gene_id	MSTRG.15535;

15	StringTie	exon	72460519	72460562	.	+	.	transcript_	MSTRG.15535.:	gene_id	MSTRG.15535;
15	StringTie	transcrip	80535703	80536904	.	+	.	transcript_	MSTRG.15669.:	gene_id	MSTRG.15669;
15	StringTie	exon	80535703	80535945	.	+	.	transcript_	MSTRG.15669.:	gene_id	MSTRG.15669;
15	StringTie	exon	80536025	80536904	.	+	.	transcript_	MSTRG.15669.:	gene_id	MSTRG.15669;
15	StringTie	transcrip	80551529	80551994	.	+	.	transcript_	MSTRG.15670.:	gene_id	MSTRG.15670;
15	StringTie	exon	80551529	80551755	.	+	.	transcript_	MSTRG.15670.:	gene_id	MSTRG.15670;
15	StringTie	exon	80551866	80551994	.	+	.	transcript_	MSTRG.15670.:	gene_id	MSTRG.15670;
15	StringTie	transcrip	81678633	81681344	.	+	.	transcript_	MSTRG.15726.:	gene_id	MSTRG.15726;
15	StringTie	exon	81678633	81678726	.	+	.	transcript_	MSTRG.15726.:	gene_id	MSTRG.15726;
15	StringTie	exon	81679047	81681344	.	+	.	transcript_	MSTRG.15726.:	gene_id	MSTRG.15726;
15	StringTie	transcrip	83007898	83009998	.	+	.	transcript_	MSTRG.15756.:	gene_id	MSTRG.15756;
15	StringTie	exon	83007898	83008177	.	+	.	transcript_	MSTRG.15756.:	gene_id	MSTRG.15756;
15	StringTie	exon	83009793	83009998	.	+	.	transcript_	MSTRG.15756.:	gene_id	MSTRG.15756;
15	StringTie	transcrip	84438509	84445238	.	+	.	transcript_	MSTRG.16070.:	gene_id	MSTRG.16070;
15	StringTie	exon	84438509	84439810	.	+	.	transcript_	MSTRG.16070.:	gene_id	MSTRG.16070;
15	StringTie	exon	84439867	84445238	.	+	.	transcript_	MSTRG.16070.:	gene_id	MSTRG.16070;
15	StringTie	transcrip	84484845	84531166	.	+	.	transcript_	MSTRG.16072.:	gene_id	MSTRG.16072;
15	StringTie	exon	84484845	84484860	.	+	.	transcript_	MSTRG.16072.:	gene_id	MSTRG.16072;
15	StringTie	exon	84530959	84531166	.	+	.	transcript_	MSTRG.16072.:	gene_id	MSTRG.16072;
15	StringTie	transcrip	91525509	91527843	.	+	.	transcript_	MSTRG.15907.:	gene_id	MSTRG.15907;
15	StringTie	exon	91525509	91526689	.	+	.	transcript_	MSTRG.15907.:	gene_id	MSTRG.15907;
15	StringTie	exon	91527172	91527843	.	+	.	transcript_	MSTRG.15907.:	gene_id	MSTRG.15907;
15	StringTie	transcrip	93285654	93322581	.	+	.	transcript_	MSTRG.15949.:	gene_id	MSTRG.15949;
15	StringTie	exon	93285654	93285882	.	+	.	transcript_	MSTRG.15949.:	gene_id	MSTRG.15949;
15	StringTie	exon	93297570	93297659	.	+	.	transcript_	MSTRG.15949.:	gene_id	MSTRG.15949;
15	StringTie	exon	93321648	93322581	.	+	.	transcript_	MSTRG.15949.:	gene_id	MSTRG.15949;
15	StringTie	transcrip	93412398	93519867	.	+	.	transcript_	MSTRG.15952.:	gene_id	MSTRG.15952;
15	StringTie	exon	93412398	93412492	.	+	.	transcript_	MSTRG.15952.:	gene_id	MSTRG.15952;
15	StringTie	exon	93512279	93512380	.	+	.	transcript_	MSTRG.15952.:	gene_id	MSTRG.15952;
15	StringTie	exon	93519638	93519867	.	+	.	transcript_	MSTRG.15952.:	gene_id	MSTRG.15952;
15	StringTie	transcrip	101602740	101623680	.	+	.	transcript_	MSTRG.16218.:	gene_id	MSTRG.16218;
15	StringTie	exon	101602740	101602763	.	+	.	transcript_	MSTRG.16218.:	gene_id	MSTRG.16218;
15	StringTie	exon	101616863	101616951	.	+	.	transcript_	MSTRG.16218.:	gene_id	MSTRG.16218;
15	StringTie	exon	101618374	101618467	.	+	.	transcript_	MSTRG.16218.:	gene_id	MSTRG.16218;
15	StringTie	exon	101623287	101623680	.	+	.	transcript_	MSTRG.16218.:	gene_id	MSTRG.16218;

15	StringTie	transcrip	106765709	106775481	.	+	.	transcript_	MSTRG.16358.1	gene_id	MSTRG.16358;
15	StringTie	exon	106765709	106773175	.	+	.	transcript_	MSTRG.16358.1	gene_id	MSTRG.16358;
15	StringTie	exon	106775389	106775481	.	+	.	transcript_	MSTRG.16358.1	gene_id	MSTRG.16358;
15	StringTie	transcrip	126567967	126568549	.	+	.	transcript_	MSTRG.16742.1	gene_id	MSTRG.16742;
15	StringTie	exon	126567967	126568296	.	+	.	transcript_	MSTRG.16742.1	gene_id	MSTRG.16742;
15	StringTie	exon	126568372	126568549	.	+	.	transcript_	MSTRG.16742.1	gene_id	MSTRG.16742;
15	StringTie	transcrip	132171104	132171347	.	+	.	transcript_	MSTRG.16808.1	gene_id	MSTRG.16808;
15	StringTie	exon	132171104	132171214	.	+	.	transcript_	MSTRG.16808.1	gene_id	MSTRG.16808;
15	StringTie	exon	132171257	132171347	.	+	.	transcript_	MSTRG.16808.1	gene_id	MSTRG.16808;
15	StringTie	transcrip	133756683	133759125	.	+	.	transcript_	MSTRG.16830.1	gene_id	MSTRG.16830;
15	StringTie	exon	133756683	133758023	.	+	.	transcript_	MSTRG.16830.1	gene_id	MSTRG.16830;
15	StringTie	exon	133758531	133759125	.	+	.	transcript_	MSTRG.16830.1	gene_id	MSTRG.16830;
15	StringTie	transcrip	136855154	136859849	.	+	.	transcript_	MSTRG.16870.1	gene_id	MSTRG.16870;
15	StringTie	exon	136855154	136855435	.	+	.	transcript_	MSTRG.16870.1	gene_id	MSTRG.16870;
15	StringTie	exon	136855881	136859849	.	+	.	transcript_	MSTRG.16870.1	gene_id	MSTRG.16870;
15	StringTie	transcrip	137025831	137026112	.	+	.	transcript_	MSTRG.16882.2	gene_id	MSTRG.16882;
15	StringTie	exon	137025831	137025941	.	+	.	transcript_	MSTRG.16882.2	gene_id	MSTRG.16882;
15	StringTie	exon	137025972	137026112	.	+	.	transcript_	MSTRG.16882.2	gene_id	MSTRG.16882;
15	StringTie	transcrip	137025831	137026112	.	+	.	transcript_	MSTRG.16882.1	gene_id	MSTRG.16882;
15	StringTie	exon	137025831	137025941	.	+	.	transcript_	MSTRG.16882.1	gene_id	MSTRG.16882;
15	StringTie	exon	137026002	137026112	.	+	.	transcript_	MSTRG.16882.1	gene_id	MSTRG.16882;
15	StringTie	transcrip	137833729	137834533	.	+	.	transcript_	MSTRG.16902.1	gene_id	MSTRG.16902;
15	StringTie	exon	137833729	137834065	.	+	.	transcript_	MSTRG.16902.1	gene_id	MSTRG.16902;
15	StringTie	exon	137834257	137834533	.	+	.	transcript_	MSTRG.16902.1	gene_id	MSTRG.16902;
15	StringTie	transcrip	138471378	138472849	.	+	.	transcript_	MSTRG.16920.1	gene_id	MSTRG.16920;
15	StringTie	exon	138471378	138471564	.	+	.	transcript_	MSTRG.16920.1	gene_id	MSTRG.16920;
15	StringTie	exon	138472522	138472849	.	+	.	transcript_	MSTRG.16920.1	gene_id	MSTRG.16920;
15	StringTie	transcrip	138682805	138684638	.	+	.	transcript_	MSTRG.16904.1	gene_id	MSTRG.16904;
15	StringTie	exon	138682805	138682927	.	+	.	transcript_	MSTRG.16904.1	gene_id	MSTRG.16904;
15	StringTie	exon	138683919	138684638	.	+	.	transcript_	MSTRG.16904.1	gene_id	MSTRG.16904;
15	StringTie	transcrip	632892	635989	.	-	.	transcript_	MSTRG.14753.1	gene_id	MSTRG.14753;
15	StringTie	exon	632892	633019	.	-	.	transcript_	MSTRG.14753.1	gene_id	MSTRG.14753;
15	StringTie	exon	635853	635989	.	-	.	transcript_	MSTRG.14753.1	gene_id	MSTRG.14753;
15	StringTie	transcrip	635564	643595	.	-	.	transcript_	MSTRG.14754.1	gene_id	MSTRG.14754;
15	StringTie	exon	635564	635852	.	-	.	transcript_	MSTRG.14754.1	gene_id	MSTRG.14754;

15	StringTie	exon	643139	643595	.	-	.	transcript_	MSTRG.14754.1	gene_id	MSTRG.14754;
15	StringTie	transcrip	2181788	2495723	.	-	.	transcript_	MSTRG.14538.2	gene_id	MSTRG.14538;
15	StringTie	exon	2181788	2181968	.	-	.	transcript_	MSTRG.14538.2	gene_id	MSTRG.14538;
15	StringTie	exon	2201659	2201857	.	-	.	transcript_	MSTRG.14538.2	gene_id	MSTRG.14538;
15	StringTie	exon	2495672	2495723	.	-	.	transcript_	MSTRG.14538.2	gene_id	MSTRG.14538;
15	StringTie	transcrip	2473719	2474470	.	-	.	transcript_	MSTRG.14540.1	gene_id	MSTRG.14540;
15	StringTie	exon	2473719	2473797	.	-	.	transcript_	MSTRG.14540.1	gene_id	MSTRG.14540;
15	StringTie	exon	2473898	2474470	.	-	.	transcript_	MSTRG.14540.1	gene_id	MSTRG.14540;
15	StringTie	transcrip	3787964	3788720	.	-	.	transcript_	MSTRG.14586.1	gene_id	MSTRG.14586;
15	StringTie	exon	3787964	3788390	.	-	.	transcript_	MSTRG.14586.1	gene_id	MSTRG.14586;
15	StringTie	exon	3788684	3788720	.	-	.	transcript_	MSTRG.14586.1	gene_id	MSTRG.14586;
15	StringTie	transcrip	6849546	7073402	.	-	.	transcript_	MSTRG.14575.1	gene_id	MSTRG.14575;
15	StringTie	exon	6849546	6849600	.	-	.	transcript_	MSTRG.14575.1	gene_id	MSTRG.14575;
15	StringTie	exon	7027549	7027732	.	-	.	transcript_	MSTRG.14575.1	gene_id	MSTRG.14575;
15	StringTie	exon	7073375	7073402	.	-	.	transcript_	MSTRG.14575.1	gene_id	MSTRG.14575;
15	StringTie	transcrip	7489953	7495344	.	-	.	transcript_	MSTRG.14583.1	gene_id	MSTRG.14583;
15	StringTie	exon	7489953	7490614	.	-	.	transcript_	MSTRG.14583.1	gene_id	MSTRG.14583;
15	StringTie	exon	7491382	7491611	.	-	.	transcript_	MSTRG.14583.1	gene_id	MSTRG.14583;
15	StringTie	exon	7493812	7493970	.	-	.	transcript_	MSTRG.14583.1	gene_id	MSTRG.14583;
15	StringTie	exon	7495113	7495344	.	-	.	transcript_	MSTRG.14583.1	gene_id	MSTRG.14583;
15	StringTie	transcrip	20043297	20043654	.	-	.	transcript_	MSTRG.14812.1	gene_id	MSTRG.14812;
15	StringTie	exon	20043297	20043495	.	-	.	transcript_	MSTRG.14812.1	gene_id	MSTRG.14812;
15	StringTie	exon	20043603	20043654	.	-	.	transcript_	MSTRG.14812.1	gene_id	MSTRG.14812;
15	StringTie	transcrip	44898868	44917584	.	-	.	transcript_	MSTRG.15102.1	gene_id	MSTRG.15102;
15	StringTie	exon	44898868	44901404	.	-	.	transcript_	MSTRG.15102.1	gene_id	MSTRG.15102;
15	StringTie	exon	44908934	44909093	.	-	.	transcript_	MSTRG.15102.1	gene_id	MSTRG.15102;
15	StringTie	exon	44917481	44917584	.	-	.	transcript_	MSTRG.15102.1	gene_id	MSTRG.15102;
15	StringTie	transcrip	46197637	46197900	.	-	.	transcript_	MSTRG.15134.1	gene_id	MSTRG.15134;
15	StringTie	exon	46197637	46197747	.	-	.	transcript_	MSTRG.15134.1	gene_id	MSTRG.15134;
15	StringTie	exon	46197790	46197900	.	-	.	transcript_	MSTRG.15134.1	gene_id	MSTRG.15134;
15	StringTie	transcrip	70676207	70724074	.	-	.	transcript_	MSTRG.15505.1	gene_id	MSTRG.15505;
15	StringTie	exon	70676207	70676465	.	-	.	transcript_	MSTRG.15505.1	gene_id	MSTRG.15505;
15	StringTie	exon	70723997	70724074	.	-	.	transcript_	MSTRG.15505.1	gene_id	MSTRG.15505;
15	StringTie	transcrip	70696118	70720135	.	-	.	transcript_	MSTRG.15507.1	gene_id	MSTRG.15507;
15	StringTie	exon	70696118	70696302	.	-	.	transcript_	MSTRG.15507.1	gene_id	MSTRG.15507;

15	StringTie	exon	70720015	70720135	.	-	.	transcript_	MSTRG.15507.	gene_id	MSTRG.15507;
15	StringTie	transcrip	75786166	75790314	.	-	.	transcript_	MSTRG.15561.	gene_id	MSTRG.15561;
15	StringTie	exon	75786166	75786338	.	-	.	transcript_	MSTRG.15561.	gene_id	MSTRG.15561;
15	StringTie	exon	75790201	75790314	.	-	.	transcript_	MSTRG.15561.	gene_id	MSTRG.15561;
15	StringTie	transcrip	78619031	78641445	.	-	.	transcript_	MSTRG.15633.	gene_id	MSTRG.15633;
15	StringTie	exon	78619031	78619580	.	-	.	transcript_	MSTRG.15633.	gene_id	MSTRG.15633;
15	StringTie	exon	78640800	78640884	.	-	.	transcript_	MSTRG.15633.	gene_id	MSTRG.15633;
15	StringTie	exon	78641288	78641445	.	-	.	transcript_	MSTRG.15633.	gene_id	MSTRG.15633;
15	StringTie	transcrip	78680635	78708958	.	-	.	transcript_	MSTRG.15647.	gene_id	MSTRG.15647;
15	StringTie	exon	78680635	78681113	.	-	.	transcript_	MSTRG.15647.	gene_id	MSTRG.15647;
15	StringTie	exon	78708059	78708094	.	-	.	transcript_	MSTRG.15647.	gene_id	MSTRG.15647;
15	StringTie	exon	78708903	78708958	.	-	.	transcript_	MSTRG.15647.	gene_id	MSTRG.15647;
15	StringTie	transcrip	78721317	78774255	.	-	.	transcript_	MSTRG.15651.	gene_id	MSTRG.15651;
15	StringTie	exon	78721317	78722235	.	-	.	transcript_	MSTRG.15651.	gene_id	MSTRG.15651;
15	StringTie	exon	78723276	78723477	.	-	.	transcript_	MSTRG.15651.	gene_id	MSTRG.15651;
15	StringTie	exon	78733684	78733766	.	-	.	transcript_	MSTRG.15651.	gene_id	MSTRG.15651;
15	StringTie	exon	78771975	78772183	.	-	.	transcript_	MSTRG.15651.	gene_id	MSTRG.15651;
15	StringTie	exon	78773788	78774255	.	-	.	transcript_	MSTRG.15651.	gene_id	MSTRG.15651;
15	StringTie	transcrip	79253739	79256433	.	-	.	transcript_	MSTRG.15655.	gene_id	MSTRG.15655;
15	StringTie	exon	79253739	79254526	.	-	.	transcript_	MSTRG.15655.	gene_id	MSTRG.15655;
15	StringTie	exon	79256409	79256433	.	-	.	transcript_	MSTRG.15655.	gene_id	MSTRG.15655;
15	StringTie	transcrip	81935537	81938117	.	-	.	transcript_	MSTRG.15722.	gene_id	MSTRG.15722;
15	StringTie	exon	81935537	81936330	.	-	.	transcript_	MSTRG.15722.	gene_id	MSTRG.15722;
15	StringTie	exon	81938009	81938117	.	-	.	transcript_	MSTRG.15722.	gene_id	MSTRG.15722;
15	StringTie	transcrip	81975462	81987753	.	-	.	transcript_	MSTRG.15732.	gene_id	MSTRG.15732;
15	StringTie	exon	81975462	81976055	.	-	.	transcript_	MSTRG.15732.	gene_id	MSTRG.15732;
15	StringTie	exon	81976985	81977168	.	-	.	transcript_	MSTRG.15732.	gene_id	MSTRG.15732;
15	StringTie	exon	81987597	81987753	.	-	.	transcript_	MSTRG.15732.	gene_id	MSTRG.15732;
15	StringTie	transcrip	83137991	83146145	.	-	.	transcript_	MSTRG.15776.	gene_id	MSTRG.15776;
15	StringTie	exon	83137991	83138205	.	-	.	transcript_	MSTRG.15776.	gene_id	MSTRG.15776;
15	StringTie	exon	83143710	83143791	.	-	.	transcript_	MSTRG.15776.	gene_id	MSTRG.15776;
15	StringTie	exon	83146056	83146145	.	-	.	transcript_	MSTRG.15776.	gene_id	MSTRG.15776;
15	StringTie	transcrip	88582164	88587653	.	-	.	transcript_	MSTRG.15896.	gene_id	MSTRG.15896;
15	StringTie	exon	88582164	88582284	.	-	.	transcript_	MSTRG.15896.	gene_id	MSTRG.15896;
15	StringTie	exon	88587423	88587653	.	-	.	transcript_	MSTRG.15896.	gene_id	MSTRG.15896;

15	StringTie	transcrip	91884490	91886091	.	-	.	transcript_	MSTRG.15931.5	gene_id	MSTRG.15931;
15	StringTie	exon	91884490	91884589	.	-	.	transcript_	MSTRG.15931.5	gene_id	MSTRG.15931;
15	StringTie	exon	91884902	91885052	.	-	.	transcript_	MSTRG.15931.5	gene_id	MSTRG.15931;
15	StringTie	exon	91886041	91886091	.	-	.	transcript_	MSTRG.15931.5	gene_id	MSTRG.15931;
15	StringTie	transcrip	93314056	93321330	.	-	.	transcript_	MSTRG.15950.1	gene_id	MSTRG.15950;
15	StringTie	exon	93314056	93314699	.	-	.	transcript_	MSTRG.15950.1	gene_id	MSTRG.15950;
15	StringTie	exon	93320538	93321330	.	-	.	transcript_	MSTRG.15950.1	gene_id	MSTRG.15950;
15	StringTie	transcrip	93321715	93341671	.	-	.	transcript_	MSTRG.15951.1	gene_id	MSTRG.15951;
15	StringTie	exon	93321715	93322972	.	-	.	transcript_	MSTRG.15951.1	gene_id	MSTRG.15951;
15	StringTie	exon	93326852	93326972	.	-	.	transcript_	MSTRG.15951.1	gene_id	MSTRG.15951;
15	StringTie	exon	93334932	93335003	.	-	.	transcript_	MSTRG.15951.1	gene_id	MSTRG.15951;
15	StringTie	exon	93341321	93341671	.	-	.	transcript_	MSTRG.15951.1	gene_id	MSTRG.15951;
15	StringTie	transcrip	93322745	93414630	.	-	.	transcript_	MSTRG.15951.2	gene_id	MSTRG.15951;
15	StringTie	exon	93322745	93322972	.	-	.	transcript_	MSTRG.15951.2	gene_id	MSTRG.15951;
15	StringTie	exon	93326852	93326972	.	-	.	transcript_	MSTRG.15951.2	gene_id	MSTRG.15951;
15	StringTie	exon	93330016	93330108	.	-	.	transcript_	MSTRG.15951.2	gene_id	MSTRG.15951;
15	StringTie	exon	93334932	93335003	.	-	.	transcript_	MSTRG.15951.2	gene_id	MSTRG.15951;
15	StringTie	exon	93414543	93414630	.	-	.	transcript_	MSTRG.15951.2	gene_id	MSTRG.15951;
15	StringTie	transcrip	94008979	94134743	.	-	.	transcript_	MSTRG.15975.1	gene_id	MSTRG.15975;
15	StringTie	exon	94008979	94009091	.	-	.	transcript_	MSTRG.15975.1	gene_id	MSTRG.15975;
15	StringTie	exon	94134312	94134743	.	-	.	transcript_	MSTRG.15975.1	gene_id	MSTRG.15975;
15	StringTie	transcrip	95524908	95527770	.	-	.	transcript_	MSTRG.16054.1	gene_id	MSTRG.16054;
15	StringTie	exon	95524908	95524996	.	-	.	transcript_	MSTRG.16054.1	gene_id	MSTRG.16054;
15	StringTie	exon	95526368	95527770	.	-	.	transcript_	MSTRG.16054.1	gene_id	MSTRG.16054;
15	StringTie	transcrip	95838609	95845076	.	-	.	transcript_	MSTRG.16041.1	gene_id	MSTRG.16041;
15	StringTie	exon	95838609	95839016	.	-	.	transcript_	MSTRG.16041.1	gene_id	MSTRG.16041;
15	StringTie	exon	95845034	95845076	.	-	.	transcript_	MSTRG.16041.1	gene_id	MSTRG.16041;
15	StringTie	transcrip	96209790	96313528	.	-	.	transcript_	MSTRG.16057.1	gene_id	MSTRG.16057;
15	StringTie	exon	96209790	96210050	.	-	.	transcript_	MSTRG.16057.1	gene_id	MSTRG.16057;
15	StringTie	exon	96232202	96232236	.	-	.	transcript_	MSTRG.16057.1	gene_id	MSTRG.16057;
15	StringTie	exon	96312086	96312182	.	-	.	transcript_	MSTRG.16057.1	gene_id	MSTRG.16057;
15	StringTie	exon	96313389	96313528	.	-	.	transcript_	MSTRG.16057.1	gene_id	MSTRG.16057;
15	StringTie	transcrip	96246473	96278337	.	-	.	transcript_	MSTRG.16057.2	gene_id	MSTRG.16057;
15	StringTie	exon	96246473	96246824	.	-	.	transcript_	MSTRG.16057.2	gene_id	MSTRG.16057;
15	StringTie	exon	96278165	96278337	.	-	.	transcript_	MSTRG.16057.2	gene_id	MSTRG.16057;

15	StringTie	transcrip	96246473	96313549	.	-	.	transcript_	MSTRG.16057.3	gene_id	MSTRG.16057;
15	StringTie	exon	96246473	96246824	.	-	.	transcript_	MSTRG.16057.3	gene_id	MSTRG.16057;
15	StringTie	exon	96312086	96312182	.	-	.	transcript_	MSTRG.16057.3	gene_id	MSTRG.16057;
15	StringTie	exon	96313389	96313549	.	-	.	transcript_	MSTRG.16057.3	gene_id	MSTRG.16057;
15	StringTie	transcrip	96278373	96313549	.	-	.	transcript_	MSTRG.16057.4	gene_id	MSTRG.16057;
15	StringTie	exon	96278373	96278763	.	-	.	transcript_	MSTRG.16057.4	gene_id	MSTRG.16057;
15	StringTie	exon	96312086	96312182	.	-	.	transcript_	MSTRG.16057.4	gene_id	MSTRG.16057;
15	StringTie	exon	96313389	96313549	.	-	.	transcript_	MSTRG.16057.4	gene_id	MSTRG.16057;
15	StringTie	transcrip	96280781	96313535	.	-	.	transcript_	MSTRG.16057.5	gene_id	MSTRG.16057;
15	StringTie	exon	96280781	96281144	.	-	.	transcript_	MSTRG.16057.5	gene_id	MSTRG.16057;
15	StringTie	exon	96312086	96312182	.	-	.	transcript_	MSTRG.16057.5	gene_id	MSTRG.16057;
15	StringTie	exon	96313389	96313535	.	-	.	transcript_	MSTRG.16057.5	gene_id	MSTRG.16057;
15	StringTie	transcrip	104182149	104183220	.	-	.	transcript_	MSTRG.16263.1	gene_id	MSTRG.16263;
15	StringTie	exon	104182149	104182987	.	-	.	transcript_	MSTRG.16263.1	gene_id	MSTRG.16263;
15	StringTie	exon	104183048	104183220	.	-	.	transcript_	MSTRG.16263.1	gene_id	MSTRG.16263;
15	StringTie	transcrip	106945685	106947672	.	-	.	transcript_	MSTRG.16352.4	gene_id	MSTRG.16352;
15	StringTie	exon	106945685	106946901	.	-	.	transcript_	MSTRG.16352.4	gene_id	MSTRG.16352;
15	StringTie	exon	106947215	106947672	.	-	.	transcript_	MSTRG.16352.4	gene_id	MSTRG.16352;
15	StringTie	transcrip	108910682	108913474	.	-	.	transcript_	MSTRG.16364.1	gene_id	MSTRG.16364;
15	StringTie	exon	108910682	108910824	.	-	.	transcript_	MSTRG.16364.1	gene_id	MSTRG.16364;
15	StringTie	exon	108910847	108913474	.	-	.	transcript_	MSTRG.16364.1	gene_id	MSTRG.16364;
15	StringTie	transcrip	110565226	110697548	.	-	.	transcript_	MSTRG.16422.1	gene_id	MSTRG.16422;
15	StringTie	exon	110565226	110565623	.	-	.	transcript_	MSTRG.16422.1	gene_id	MSTRG.16422;
15	StringTie	exon	110566363	110566413	.	-	.	transcript_	MSTRG.16422.1	gene_id	MSTRG.16422;
15	StringTie	exon	110695501	110695552	.	-	.	transcript_	MSTRG.16422.1	gene_id	MSTRG.16422;
15	StringTie	exon	110697366	110697548	.	-	.	transcript_	MSTRG.16422.1	gene_id	MSTRG.16422;
15	StringTie	transcrip	110849026	110859079	.	-	.	transcript_	MSTRG.16420.1	gene_id	MSTRG.16420;
15	StringTie	exon	110849026	110849264	.	-	.	transcript_	MSTRG.16420.1	gene_id	MSTRG.16420;
15	StringTie	exon	110858751	110859079	.	-	.	transcript_	MSTRG.16420.1	gene_id	MSTRG.16420;
15	StringTie	transcrip	120319928	120333798	.	-	.	transcript_	MSTRG.16589.1	gene_id	MSTRG.16589;
15	StringTie	exon	120319928	120320914	.	-	.	transcript_	MSTRG.16589.1	gene_id	MSTRG.16589;
15	StringTie	exon	120333710	120333798	.	-	.	transcript_	MSTRG.16589.1	gene_id	MSTRG.16589;
15	StringTie	transcrip	121272640	121272945	.	-	.	transcript_	MSTRG.16611.1	gene_id	MSTRG.16611;
15	StringTie	exon	121272640	121272741	.	-	.	transcript_	MSTRG.16611.1	gene_id	MSTRG.16611;
15	StringTie	exon	121272846	121272945	.	-	.	transcript_	MSTRG.16611.1	gene_id	MSTRG.16611;

15	StringTie	transcrip	121434646	121435254	.	-	.	transcript_	MSTRG.16664.[illegible]	gene_id	MSTRG.16664;
15	StringTie	exon	121434646	121434756	.	-	.	transcript_	MSTRG.16664.[illegible]	gene_id	MSTRG.16664;
15	StringTie	exon	121435144	121435254	.	-	.	transcript_	MSTRG.16664.[illegible]	gene_id	MSTRG.16664;
15	StringTie	transcrip	127660272	127706625	.	-	.	transcript_	MSTRG.16727.[illegible]	gene_id	MSTRG.16727;
15	StringTie	exon	127660272	127660791	.	-	.	transcript_	MSTRG.16727.[illegible]	gene_id	MSTRG.16727;
15	StringTie	exon	127706451	127706625	.	-	.	transcript_	MSTRG.16727.[illegible]	gene_id	MSTRG.16727;
15	StringTie	transcrip	127660323	127685193	.	-	.	transcript_	MSTRG.16727.[illegible]	gene_id	MSTRG.16727;
15	StringTie	exon	127660323	127660791	.	-	.	transcript_	MSTRG.16727.[illegible]	gene_id	MSTRG.16727;
15	StringTie	exon	127684189	127685193	.	-	.	transcript_	MSTRG.16727.[illegible]	gene_id	MSTRG.16727;
15	StringTie	transcrip	127660323	127706638	.	-	.	transcript_	MSTRG.16727.[illegible]	gene_id	MSTRG.16727;
15	StringTie	exon	127660323	127660791	.	-	.	transcript_	MSTRG.16727.[illegible]	gene_id	MSTRG.16727;
15	StringTie	exon	127661751	127661932	.	-	.	transcript_	MSTRG.16727.[illegible]	gene_id	MSTRG.16727;
15	StringTie	exon	127700915	127701862	.	-	.	transcript_	MSTRG.16727.[illegible]	gene_id	MSTRG.16727;
15	StringTie	exon	127705061	127705218	.	-	.	transcript_	MSTRG.16727.[illegible]	gene_id	MSTRG.16727;
15	StringTie	exon	127706157	127706638	.	-	.	transcript_	MSTRG.16727.[illegible]	gene_id	MSTRG.16727;
15	StringTie	transcrip	127661123	127706664	.	-	.	transcript_	MSTRG.16727.[illegible]	gene_id	MSTRG.16727;
15	StringTie	exon	127661123	127661932	.	-	.	transcript_	MSTRG.16727.[illegible]	gene_id	MSTRG.16727;
15	StringTie	exon	127700915	127701024	.	-	.	transcript_	MSTRG.16727.[illegible]	gene_id	MSTRG.16727;
15	StringTie	exon	127705061	127705218	.	-	.	transcript_	MSTRG.16727.[illegible]	gene_id	MSTRG.16727;
15	StringTie	exon	127705817	127706664	.	-	.	transcript_	MSTRG.16727.[illegible]	gene_id	MSTRG.16727;
15	StringTie	transcrip	127661139	127706638	.	-	.	transcript_	MSTRG.16727.[illegible]	gene_id	MSTRG.16727;
15	StringTie	exon	127661139	127661932	.	-	.	transcript_	MSTRG.16727.[illegible]	gene_id	MSTRG.16727;
15	StringTie	exon	127684189	127684299	.	-	.	transcript_	MSTRG.16727.[illegible]	gene_id	MSTRG.16727;
15	StringTie	exon	127700915	127701862	.	-	.	transcript_	MSTRG.16727.[illegible]	gene_id	MSTRG.16727;
15	StringTie	exon	127703974	127706638	.	-	.	transcript_	MSTRG.16727.[illegible]	gene_id	MSTRG.16727;
15	StringTie	transcrip	127661602	127706638	.	-	.	transcript_	MSTRG.16727.[illegible]	gene_id	MSTRG.16727;
15	StringTie	exon	127661602	127661932	.	-	.	transcript_	MSTRG.16727.[illegible]	gene_id	MSTRG.16727;
15	StringTie	exon	127700915	127701862	.	-	.	transcript_	MSTRG.16727.[illegible]	gene_id	MSTRG.16727;
15	StringTie	exon	127706451	127706638	.	-	.	transcript_	MSTRG.16727.[illegible]	gene_id	MSTRG.16727;
15	StringTie	transcrip	127661606	127706616	.	-	.	transcript_	MSTRG.16727.[illegible]	gene_id	MSTRG.16727;
15	StringTie	exon	127661606	127661932	.	-	.	transcript_	MSTRG.16727.[illegible]	gene_id	MSTRG.16727;
15	StringTie	exon	127700915	127701862	.	-	.	transcript_	MSTRG.16727.[illegible]	gene_id	MSTRG.16727;
15	StringTie	exon	127706157	127706616	.	-	.	transcript_	MSTRG.16727.[illegible]	gene_id	MSTRG.16727;
15	StringTie	transcrip	127661606	127706616	.	-	.	transcript_	MSTRG.16727.[illegible]	gene_id	MSTRG.16727;
15	StringTie	exon	127661606	127661932	.	-	.	transcript_	MSTRG.16727.[illegible]	gene_id	MSTRG.16727;

15	StringTie	exon	127700915	127701862	.	-	.	transcript_	MSTRG.16727.1	gene_id	MSTRG.16727;
15	StringTie	exon	127706182	127706616	.	-	.	transcript_	MSTRG.16727.1	gene_id	MSTRG.16727;
15	StringTie	transcrip	127704304	127706616	.	-	.	transcript_	MSTRG.16727.1	gene_id	MSTRG.16727;
15	StringTie	exon	127704304	127705218	.	-	.	transcript_	MSTRG.16727.1	gene_id	MSTRG.16727;
15	StringTie	exon	127705822	127706106	.	-	.	transcript_	MSTRG.16727.1	gene_id	MSTRG.16727;
15	StringTie	exon	127706451	127706616	.	-	.	transcript_	MSTRG.16727.1	gene_id	MSTRG.16727;
15	StringTie	transcrip	135588451	135593554	.	-	.	transcript_	MSTRG.16853.1	gene_id	MSTRG.16853;
15	StringTie	exon	135588451	135588644	.	-	.	transcript_	MSTRG.16853.1	gene_id	MSTRG.16853;
15	StringTie	exon	135593473	135593554	.	-	.	transcript_	MSTRG.16853.1	gene_id	MSTRG.16853;
15	StringTie	transcrip	136052662	136054534	.	-	.	transcript_	MSTRG.16855.1	gene_id	MSTRG.16855;
15	StringTie	exon	136052662	136053449	.	-	.	transcript_	MSTRG.16855.1	gene_id	MSTRG.16855;
15	StringTie	exon	136053759	136054534	.	-	.	transcript_	MSTRG.16855.1	gene_id	MSTRG.16855;
15	StringTie	transcrip	138219902	138224265	.	-	.	transcript_	MSTRG.16896.1	gene_id	MSTRG.16896;
15	StringTie	exon	138219902	138224265	.	-	.	transcript_	MSTRG.16896.1	gene_id	MSTRG.16896;
15	StringTie	transcrip	138219910	138221878	.	-	.	transcript_	MSTRG.16896.2	gene_id	MSTRG.16896;
15	StringTie	exon	138219910	138221057	.	-	.	transcript_	MSTRG.16896.2	gene_id	MSTRG.16896;
15	StringTie	exon	138221543	138221878	.	-	.	transcript_	MSTRG.16896.2	gene_id	MSTRG.16896;
15	StringTie	transcrip	138220050	138221861	.	-	.	transcript_	MSTRG.16896.3	gene_id	MSTRG.16896;
15	StringTie	exon	138220050	138221057	.	-	.	transcript_	MSTRG.16896.3	gene_id	MSTRG.16896;
15	StringTie	exon	138221578	138221861	.	-	.	transcript_	MSTRG.16896.3	gene_id	MSTRG.16896;
15	StringTie	transcrip	139552989	139557318	.	-	.	transcript_	MSTRG.16930.1	gene_id	MSTRG.16930;
15	StringTie	exon	139552989	139553222	.	-	.	transcript_	MSTRG.16930.1	gene_id	MSTRG.16930;
15	StringTie	exon	139557208	139557318	.	-	.	transcript_	MSTRG.16930.1	gene_id	MSTRG.16930;
15	StringTie	transcrip	140165979	140172600	.	-	.	transcript_	MSTRG.16950.1	gene_id	MSTRG.16950;
15	StringTie	exon	140165979	140166181	.	-	.	transcript_	MSTRG.16950.1	gene_id	MSTRG.16950;
15	StringTie	exon	140170601	140170679	.	-	.	transcript_	MSTRG.16950.1	gene_id	MSTRG.16950;
15	StringTie	exon	140170780	140170931	.	-	.	transcript_	MSTRG.16950.1	gene_id	MSTRG.16950;
15	StringTie	exon	140172516	140172600	.	-	.	transcript_	MSTRG.16950.1	gene_id	MSTRG.16950;
16	StringTie	transcrip	322182	339536	.	+	.	transcript_	MSTRG.16974.2	gene_id	MSTRG.16974;
16	StringTie	exon	322182	322445	.	+	.	transcript_	MSTRG.16974.2	gene_id	MSTRG.16974;
16	StringTie	exon	339225	339536	.	+	.	transcript_	MSTRG.16974.2	gene_id	MSTRG.16974;
16	StringTie	transcrip	322182	345236	.	+	.	transcript_	MSTRG.16974.1	gene_id	MSTRG.16974;
16	StringTie	exon	322182	322379	.	+	.	transcript_	MSTRG.16974.1	gene_id	MSTRG.16974;
16	StringTie	exon	339225	339483	.	+	.	transcript_	MSTRG.16974.1	gene_id	MSTRG.16974;
16	StringTie	exon	345150	345236	.	+	.	transcript_	MSTRG.16974.1	gene_id	MSTRG.16974;

16	StringTie	transcrip	322210	345407	.	+	.	transcript_	MSTRG.16974.:	gene_id	MSTRG.16974;
16	StringTie	exon	322210	322379	.	+	.	transcript_	MSTRG.16974.:	gene_id	MSTRG.16974;
16	StringTie	exon	339225	339469	.	+	.	transcript_	MSTRG.16974.:	gene_id	MSTRG.16974;
16	StringTie	exon	345150	345407	.	+	.	transcript_	MSTRG.16974.:	gene_id	MSTRG.16974;
16	StringTie	transcrip	6765575	6770482	.	+	.	transcript_	MSTRG.16998.	gene_id	MSTRG.16998;
16	StringTie	exon	6765575	6765801	.	+	.	transcript_	MSTRG.16998.	gene_id	MSTRG.16998;
16	StringTie	exon	6768129	6770482	.	+	.	transcript_	MSTRG.16998.	gene_id	MSTRG.16998;
16	StringTie	transcrip	18146807	18147086	.	+	.	transcript_	MSTRG.17036.	gene_id	MSTRG.17036;
16	StringTie	exon	18146807	18146995	.	+	.	transcript_	MSTRG.17036.	gene_id	MSTRG.17036;
16	StringTie	exon	18147045	18147086	.	+	.	transcript_	MSTRG.17036.	gene_id	MSTRG.17036;
16	StringTie	transcrip	19454145	19454932	.	+	.	transcript_	MSTRG.17065.	gene_id	MSTRG.17065;
16	StringTie	exon	19454145	19454766	.	+	.	transcript_	MSTRG.17065.	gene_id	MSTRG.17065;
16	StringTie	exon	19454790	19454932	.	+	.	transcript_	MSTRG.17065.	gene_id	MSTRG.17065;
16	StringTie	transcrip	20502233	20502772	.	+	.	transcript_	MSTRG.17082.	gene_id	MSTRG.17082;
16	StringTie	exon	20502233	20502328	.	+	.	transcript_	MSTRG.17082.	gene_id	MSTRG.17082;
16	StringTie	exon	20502604	20502772	.	+	.	transcript_	MSTRG.17082.	gene_id	MSTRG.17082;
16	StringTie	transcrip	21907643	21930231	.	+	.	transcript_	MSTRG.17108.	gene_id	MSTRG.17108;
16	StringTie	exon	21907643	21908734	.	+	.	transcript_	MSTRG.17108.	gene_id	MSTRG.17108;
16	StringTie	exon	21930164	21930231	.	+	.	transcript_	MSTRG.17108.	gene_id	MSTRG.17108;
16	StringTie	transcrip	22329010	22330930	.	+	.	transcript_	MSTRG.17113.	gene_id	MSTRG.17113;
16	StringTie	exon	22329010	22329518	.	+	.	transcript_	MSTRG.17113.	gene_id	MSTRG.17113;
16	StringTie	exon	22330132	22330930	.	+	.	transcript_	MSTRG.17113.	gene_id	MSTRG.17113;
16	StringTie	transcrip	22942993	22944310	.	+	.	transcript_	MSTRG.17124.	gene_id	MSTRG.17124;
16	StringTie	exon	22942993	22944138	.	+	.	transcript_	MSTRG.17124.	gene_id	MSTRG.17124;
16	StringTie	exon	22944188	22944310	.	+	.	transcript_	MSTRG.17124.	gene_id	MSTRG.17124;
16	StringTie	transcrip	23396431	23402509	.	+	.	transcript_	MSTRG.17134.	gene_id	MSTRG.17134;
16	StringTie	exon	23396431	23396676	.	+	.	transcript_	MSTRG.17134.	gene_id	MSTRG.17134;
16	StringTie	exon	23402369	23402509	.	+	.	transcript_	MSTRG.17134.	gene_id	MSTRG.17134;
16	StringTie	transcrip	26668887	26670462	.	+	.	transcript_	MSTRG.17203.	gene_id	MSTRG.17203;
16	StringTie	exon	26668887	26668985	.	+	.	transcript_	MSTRG.17203.	gene_id	MSTRG.17203;
16	StringTie	exon	26669957	26670462	.	+	.	transcript_	MSTRG.17203.	gene_id	MSTRG.17203;
16	StringTie	transcrip	27719061	27724818	.	+	.	transcript_	MSTRG.17240.	gene_id	MSTRG.17240;
16	StringTie	exon	27719061	27719349	.	+	.	transcript_	MSTRG.17240.	gene_id	MSTRG.17240;
16	StringTie	exon	27719943	27720183	.	+	.	transcript_	MSTRG.17240.	gene_id	MSTRG.17240;
16	StringTie	exon	27723739	27724818	.	+	.	transcript_	MSTRG.17240.	gene_id	MSTRG.17240;

16	StringTie	transcrip	28065926	28069320	.	+	.	transcript_	MSTRG.17261.1	gene_id	MSTRG.17261;
16	StringTie	exon	28065926	28066092	.	+	.	transcript_	MSTRG.17261.1	gene_id	MSTRG.17261;
16	StringTie	exon	28068396	28069320	.	+	.	transcript_	MSTRG.17261.1	gene_id	MSTRG.17261;
16	StringTie	transcrip	33292477	33294199	.	+	.	transcript_	MSTRG.17337.1	gene_id	MSTRG.17337;
16	StringTie	exon	33292477	33293166	.	+	.	transcript_	MSTRG.17337.1	gene_id	MSTRG.17337;
16	StringTie	exon	33293798	33294199	.	+	.	transcript_	MSTRG.17337.1	gene_id	MSTRG.17337;
16	StringTie	transcrip	47455467	47459703	.	+	.	transcript_	MSTRG.17686.1	gene_id	MSTRG.17686;
16	StringTie	exon	47455467	47455885	.	+	.	transcript_	MSTRG.17686.1	gene_id	MSTRG.17686;
16	StringTie	exon	47459577	47459703	.	+	.	transcript_	MSTRG.17686.1	gene_id	MSTRG.17686;
16	StringTie	transcrip	50425063	50430343	.	+	.	transcript_	MSTRG.17744.2	gene_id	MSTRG.17744;
16	StringTie	exon	50425063	50425449	.	+	.	transcript_	MSTRG.17744.2	gene_id	MSTRG.17744;
16	StringTie	exon	50426818	50427024	.	+	.	transcript_	MSTRG.17744.2	gene_id	MSTRG.17744;
16	StringTie	exon	50429078	50429298	.	+	.	transcript_	MSTRG.17744.2	gene_id	MSTRG.17744;
16	StringTie	exon	50429782	50430343	.	+	.	transcript_	MSTRG.17744.2	gene_id	MSTRG.17744;
16	StringTie	transcrip	63611176	63659621	.	+	.	transcript_	MSTRG.17865.1	gene_id	MSTRG.17865;
16	StringTie	exon	63611176	63611202	.	+	.	transcript_	MSTRG.17865.1	gene_id	MSTRG.17865;
16	StringTie	exon	63655707	63655800	.	+	.	transcript_	MSTRG.17865.1	gene_id	MSTRG.17865;
16	StringTie	exon	63658469	63659621	.	+	.	transcript_	MSTRG.17865.1	gene_id	MSTRG.17865;
16	StringTie	transcrip	63624475	63658999	.	+	.	transcript_	MSTRG.17865.2	gene_id	MSTRG.17865;
16	StringTie	exon	63624475	63624575	.	+	.	transcript_	MSTRG.17865.2	gene_id	MSTRG.17865;
16	StringTie	exon	63655707	63655800	.	+	.	transcript_	MSTRG.17865.2	gene_id	MSTRG.17865;
16	StringTie	exon	63658469	63658999	.	+	.	transcript_	MSTRG.17865.2	gene_id	MSTRG.17865;
16	StringTie	transcrip	69021212	69024430	.	+	.	transcript_	MSTRG.17941.1	gene_id	MSTRG.17941;
16	StringTie	exon	69021212	69023809	.	+	.	transcript_	MSTRG.17941.1	gene_id	MSTRG.17941;
16	StringTie	exon	69024116	69024430	.	+	.	transcript_	MSTRG.17941.1	gene_id	MSTRG.17941;
16	StringTie	transcrip	71276476	71281483	.	+	.	transcript_	MSTRG.17952.3	gene_id	MSTRG.17952;
16	StringTie	exon	71276476	71276851	.	+	.	transcript_	MSTRG.17952.3	gene_id	MSTRG.17952;
16	StringTie	exon	71281199	71281483	.	+	.	transcript_	MSTRG.17952.3	gene_id	MSTRG.17952;
16	StringTie	transcrip	71298464	71299113	.	+	.	transcript_	MSTRG.17953.1	gene_id	MSTRG.17953;
16	StringTie	exon	71298464	71298514	.	+	.	transcript_	MSTRG.17953.1	gene_id	MSTRG.17953;
16	StringTie	exon	71298813	71299113	.	+	.	transcript_	MSTRG.17953.1	gene_id	MSTRG.17953;
16	StringTie	transcrip	71589801	71593273	.	+	.	transcript_	MSTRG.17956.1	gene_id	MSTRG.17956;
16	StringTie	exon	71589801	71589856	.	+	.	transcript_	MSTRG.17956.1	gene_id	MSTRG.17956;
16	StringTie	exon	71591609	71591757	.	+	.	transcript_	MSTRG.17956.1	gene_id	MSTRG.17956;
16	StringTie	exon	71592707	71592904	.	+	.	transcript_	MSTRG.17956.1	gene_id	MSTRG.17956;

16	StringTie	exon	71593184	71593273	.	+	.	transcript_	MSTRG.17956.1	gene_id	MSTRG.17956;
16	StringTie	transcrip	75289042	75312167	.	+	.	transcript_	MSTRG.18010.1	gene_id	MSTRG.18010;
16	StringTie	exon	75289042	75289070	.	+	.	transcript_	MSTRG.18010.1	gene_id	MSTRG.18010;
16	StringTie	exon	75311512	75312167	.	+	.	transcript_	MSTRG.18010.1	gene_id	MSTRG.18010;
16	StringTie	transcrip	5721706	5725318	.	-	.	transcript_	MSTRG.16994.1	gene_id	MSTRG.16994;
16	StringTie	exon	5721706	5723493	.	-	.	transcript_	MSTRG.16994.1	gene_id	MSTRG.16994;
16	StringTie	exon	5724690	5725318	.	-	.	transcript_	MSTRG.16994.1	gene_id	MSTRG.16994;
16	StringTie	transcrip	18916096	18917448	.	-	.	transcript_	MSTRG.17049.2	gene_id	MSTRG.17049;
16	StringTie	exon	18916096	18917128	.	-	.	transcript_	MSTRG.17049.2	gene_id	MSTRG.17049;
16	StringTie	exon	18917214	18917448	.	-	.	transcript_	MSTRG.17049.2	gene_id	MSTRG.17049;
16	StringTie	transcrip	18916096	18980869	.	-	.	transcript_	MSTRG.17049.1	gene_id	MSTRG.17049;
16	StringTie	exon	18916096	18917128	.	-	.	transcript_	MSTRG.17049.1	gene_id	MSTRG.17049;
16	StringTie	exon	18980790	18980869	.	-	.	transcript_	MSTRG.17049.1	gene_id	MSTRG.17049;
16	StringTie	transcrip	21915039	21936691	.	-	.	transcript_	MSTRG.17109.1	gene_id	MSTRG.17109;
16	StringTie	exon	21915039	21915711	.	-	.	transcript_	MSTRG.17109.1	gene_id	MSTRG.17109;
16	StringTie	exon	21936494	21936691	.	-	.	transcript_	MSTRG.17109.1	gene_id	MSTRG.17109;
16	StringTie	transcrip	25816400	25816766	.	-	.	transcript_	MSTRG.17167.1	gene_id	MSTRG.17167;
16	StringTie	exon	25816400	25816510	.	-	.	transcript_	MSTRG.17167.1	gene_id	MSTRG.17167;
16	StringTie	exon	25816659	25816766	.	-	.	transcript_	MSTRG.17167.1	gene_id	MSTRG.17167;
16	StringTie	transcrip	27375702	27422645	.	-	.	transcript_	MSTRG.17228.1	gene_id	MSTRG.17228;
16	StringTie	exon	27375702	27375726	.	-	.	transcript_	MSTRG.17228.1	gene_id	MSTRG.17228;
16	StringTie	exon	27421472	27422645	.	-	.	transcript_	MSTRG.17228.1	gene_id	MSTRG.17228;
16	StringTie	transcrip	33731978	33746852	.	-	.	transcript_	MSTRG.17341.1	gene_id	MSTRG.17341;
16	StringTie	exon	33731978	33732136	.	-	.	transcript_	MSTRG.17341.1	gene_id	MSTRG.17341;
16	StringTie	exon	33744155	33744265	.	-	.	transcript_	MSTRG.17341.1	gene_id	MSTRG.17341;
16	StringTie	exon	33746741	33746852	.	-	.	transcript_	MSTRG.17341.1	gene_id	MSTRG.17341;
16	StringTie	transcrip	35169467	35211998	.	-	.	transcript_	MSTRG.17405.1	gene_id	MSTRG.17405;
16	StringTie	exon	35169467	35169570	.	-	.	transcript_	MSTRG.17405.1	gene_id	MSTRG.17405;
16	StringTie	exon	35192088	35192220	.	-	.	transcript_	MSTRG.17405.1	gene_id	MSTRG.17405;
16	StringTie	exon	35211711	35211998	.	-	.	transcript_	MSTRG.17405.1	gene_id	MSTRG.17405;
16	StringTie	transcrip	45454422	45461119	.	-	.	transcript_	MSTRG.17655.1	gene_id	MSTRG.17655;
16	StringTie	exon	45454422	45459226	.	-	.	transcript_	MSTRG.17655.1	gene_id	MSTRG.17655;
16	StringTie	exon	45461081	45461119	.	-	.	transcript_	MSTRG.17655.1	gene_id	MSTRG.17655;
16	StringTie	transcrip	46680663	46682596	.	-	.	transcript_	MSTRG.17642.1	gene_id	MSTRG.17642;
16	StringTie	exon	46680663	46681757	.	-	.	transcript_	MSTRG.17642.1	gene_id	MSTRG.17642;

16	StringTie	exon	46682502	46682596	.	-	.	transcript_	MSTRG.17642.1	gene_id	MSTRG.17642;
16	StringTie	transcrip	47255018	47284421	.	-	.	transcript_	MSTRG.17667.1	gene_id	MSTRG.17667;
16	StringTie	exon	47255018	47255262	.	-	.	transcript_	MSTRG.17667.1	gene_id	MSTRG.17667;
16	StringTie	exon	47284400	47284421	.	-	.	transcript_	MSTRG.17667.1	gene_id	MSTRG.17667;
16	StringTie	transcrip	50556645	50557525	.	-	.	transcript_	MSTRG.17735.1	gene_id	MSTRG.17735;
16	StringTie	exon	50556645	50556896	.	-	.	transcript_	MSTRG.17735.1	gene_id	MSTRG.17735;
16	StringTie	exon	50557468	50557525	.	-	.	transcript_	MSTRG.17735.1	gene_id	MSTRG.17735;
16	StringTie	transcrip	50717517	50733805	.	-	.	transcript_	MSTRG.17741.3	gene_id	MSTRG.17741;
16	StringTie	exon	50717517	50717622	.	-	.	transcript_	MSTRG.17741.3	gene_id	MSTRG.17741;
16	StringTie	exon	50733651	50733805	.	-	.	transcript_	MSTRG.17741.3	gene_id	MSTRG.17741;
16	StringTie	transcrip	52133305	52135531	.	-	.	transcript_	MSTRG.17781.1	gene_id	MSTRG.17781;
16	StringTie	exon	52133305	52133410	.	-	.	transcript_	MSTRG.17781.1	gene_id	MSTRG.17781;
16	StringTie	exon	52134167	52134293	.	-	.	transcript_	MSTRG.17781.1	gene_id	MSTRG.17781;
16	StringTie	exon	52135413	52135531	.	-	.	transcript_	MSTRG.17781.1	gene_id	MSTRG.17781;
16	StringTie	transcrip	52133348	52139117	.	-	.	transcript_	MSTRG.17781.2	gene_id	MSTRG.17781;
16	StringTie	exon	52133348	52133410	.	-	.	transcript_	MSTRG.17781.2	gene_id	MSTRG.17781;
16	StringTie	exon	52134167	52134293	.	-	.	transcript_	MSTRG.17781.2	gene_id	MSTRG.17781;
16	StringTie	exon	52137691	52137877	.	-	.	transcript_	MSTRG.17781.2	gene_id	MSTRG.17781;
16	StringTie	exon	52139032	52139117	.	-	.	transcript_	MSTRG.17781.2	gene_id	MSTRG.17781;
16	StringTie	transcrip	55217647	55262714	.	-	.	transcript_	MSTRG.17834.4	gene_id	MSTRG.17834;
16	StringTie	exon	55217647	55217757	.	-	.	transcript_	MSTRG.17834.4	gene_id	MSTRG.17834;
16	StringTie	exon	55262288	55262714	.	-	.	transcript_	MSTRG.17834.4	gene_id	MSTRG.17834;
16	StringTie	transcrip	55217647	55262714	.	-	.	transcript_	MSTRG.17834.1	gene_id	MSTRG.17834;
16	StringTie	exon	55217647	55217757	.	-	.	transcript_	MSTRG.17834.1	gene_id	MSTRG.17834;
16	StringTie	exon	55245584	55245776	.	-	.	transcript_	MSTRG.17834.1	gene_id	MSTRG.17834;
16	StringTie	exon	55262288	55262714	.	-	.	transcript_	MSTRG.17834.1	gene_id	MSTRG.17834;
16	StringTie	transcrip	55217647	55264125	.	-	.	transcript_	MSTRG.17834.3	gene_id	MSTRG.17834;
16	StringTie	exon	55217647	55217757	.	-	.	transcript_	MSTRG.17834.3	gene_id	MSTRG.17834;
16	StringTie	exon	55245584	55245776	.	-	.	transcript_	MSTRG.17834.3	gene_id	MSTRG.17834;
16	StringTie	exon	55264107	55264125	.	-	.	transcript_	MSTRG.17834.3	gene_id	MSTRG.17834;
16	StringTie	transcrip	55217647	55302063	.	-	.	transcript_	MSTRG.17834.2	gene_id	MSTRG.17834;
16	StringTie	exon	55217647	55217757	.	-	.	transcript_	MSTRG.17834.2	gene_id	MSTRG.17834;
16	StringTie	exon	55245584	55245776	.	-	.	transcript_	MSTRG.17834.2	gene_id	MSTRG.17834;
16	StringTie	exon	55302036	55302063	.	-	.	transcript_	MSTRG.17834.2	gene_id	MSTRG.17834;
16	StringTie	transcrip	55246327	55262714	.	-	.	transcript_	MSTRG.17834.5	gene_id	MSTRG.17834;

16	StringTie	exon	55246327	55246464	.	-	.	transcript_	MSTRG.17834.[illegible]	gene_id	MSTRG.17834;
16	StringTie	exon	55262288	55262714	.	-	.	transcript_	MSTRG.17834.[illegible]	gene_id	MSTRG.17834;
16	StringTie	transcrip	63134186	63176835	.	-	.	transcript_	MSTRG.17851.[illegible]	gene_id	MSTRG.17851;
16	StringTie	exon	63134186	63134294	.	-	.	transcript_	MSTRG.17851.[illegible]	gene_id	MSTRG.17851;
16	StringTie	exon	63136631	63136698	.	-	.	transcript_	MSTRG.17851.[illegible]	gene_id	MSTRG.17851;
16	StringTie	exon	63175663	63176835	.	-	.	transcript_	MSTRG.17851.[illegible]	gene_id	MSTRG.17851;
16	StringTie	transcrip	64650453	64676590	.	-	.	transcript_	MSTRG.17888.[illegible]	gene_id	MSTRG.17888;
16	StringTie	exon	64650453	64651325	.	-	.	transcript_	MSTRG.17888.[illegible]	gene_id	MSTRG.17888;
16	StringTie	exon	64674195	64676590	.	-	.	transcript_	MSTRG.17888.[illegible]	gene_id	MSTRG.17888;
17	StringTie	transcrip	11700	14463	.	+	.	transcript_	MSTRG.18048.[illegible]	gene_id	MSTRG.18048;
17	StringTie	exon	11700	11837	.	+	.	transcript_	MSTRG.18048.[illegible]	gene_id	MSTRG.18048;
17	StringTie	exon	14244	14463	.	+	.	transcript_	MSTRG.18048.[illegible]	gene_id	MSTRG.18048;
17	StringTie	transcrip	5333903	5341180	.	+	.	transcript_	MSTRG.18098.[illegible]	gene_id	MSTRG.18098;
17	StringTie	exon	5333903	5333924	.	+	.	transcript_	MSTRG.18098.[illegible]	gene_id	MSTRG.18098;
17	StringTie	exon	5340833	5341180	.	+	.	transcript_	MSTRG.18098.[illegible]	gene_id	MSTRG.18098;
17	StringTie	transcrip	5697534	5704938	.	+	.	transcript_	MSTRG.18115.[illegible]	gene_id	MSTRG.18115;
17	StringTie	exon	5697534	5702537	.	+	.	transcript_	MSTRG.18115.[illegible]	gene_id	MSTRG.18115;
17	StringTie	exon	5702831	5704938	.	+	.	transcript_	MSTRG.18115.[illegible]	gene_id	MSTRG.18115;
17	StringTie	transcrip	10761508	10762300	.	+	.	transcript_	MSTRG.18140.[illegible]	gene_id	MSTRG.18140;
17	StringTie	exon	10761508	10761664	.	+	.	transcript_	MSTRG.18140.[illegible]	gene_id	MSTRG.18140;
17	StringTie	exon	10762189	10762300	.	+	.	transcript_	MSTRG.18140.[illegible]	gene_id	MSTRG.18140;
17	StringTie	transcrip	10868854	10871064	.	+	.	transcript_	MSTRG.18141.[illegible]	gene_id	MSTRG.18141;
17	StringTie	exon	10868854	10868954	.	+	.	transcript_	MSTRG.18141.[illegible]	gene_id	MSTRG.18141;
17	StringTie	exon	10869325	10869441	.	+	.	transcript_	MSTRG.18141.[illegible]	gene_id	MSTRG.18141;
17	StringTie	exon	10870409	10871064	.	+	.	transcript_	MSTRG.18141.[illegible]	gene_id	MSTRG.18141;
17	StringTie	transcrip	18360130	18361462	.	+	.	transcript_	MSTRG.18243.[illegible]	gene_id	MSTRG.18243;
17	StringTie	exon	18360130	18360390	.	+	.	transcript_	MSTRG.18243.[illegible]	gene_id	MSTRG.18243;
17	StringTie	exon	18360596	18361462	.	+	.	transcript_	MSTRG.18243.[illegible]	gene_id	MSTRG.18243;
17	StringTie	transcrip	21045768	21073161	.	+	.	transcript_	MSTRG.18257.[illegible]	gene_id	MSTRG.18257;
17	StringTie	exon	21045768	21045905	.	+	.	transcript_	MSTRG.18257.[illegible]	gene_id	MSTRG.18257;
17	StringTie	exon	21073050	21073161	.	+	.	transcript_	MSTRG.18257.[illegible]	gene_id	MSTRG.18257;
17	StringTie	transcrip	26352954	26353501	.	+	.	transcript_	MSTRG.18316.[illegible]	gene_id	MSTRG.18316;
17	StringTie	exon	26352954	26353372	.	+	.	transcript_	MSTRG.18316.[illegible]	gene_id	MSTRG.18316;
17	StringTie	exon	26353397	26353501	.	+	.	transcript_	MSTRG.18316.[illegible]	gene_id	MSTRG.18316;
17	StringTie	transcrip	27099733	27123696	.	+	.	transcript_	MSTRG.18342.[illegible]	gene_id	MSTRG.18342;

17	StringTie	exon	27099733	27100020	.	+	.	transcript_	MSTRG.18342.2	gene_id	MSTRG.18342;
17	StringTie	exon	27123203	27123696	.	+	.	transcript_	MSTRG.18342.2	gene_id	MSTRG.18342;
17	StringTie	transcrip	27099758	27306022	.	+	.	transcript_	MSTRG.18342.3	gene_id	MSTRG.18342;
17	StringTie	exon	27099758	27100020	.	+	.	transcript_	MSTRG.18342.3	gene_id	MSTRG.18342;
17	StringTie	exon	27218192	27218429	.	+	.	transcript_	MSTRG.18342.3	gene_id	MSTRG.18342;
17	StringTie	exon	27305815	27306022	.	+	.	transcript_	MSTRG.18342.3	gene_id	MSTRG.18342;
17	StringTie	transcrip	34164816	34166190	.	+	.	transcript_	MSTRG.18505.1	gene_id	MSTRG.18505;
17	StringTie	exon	34164816	34164976	.	+	.	transcript_	MSTRG.18505.1	gene_id	MSTRG.18505;
17	StringTie	exon	34166023	34166190	.	+	.	transcript_	MSTRG.18505.1	gene_id	MSTRG.18505;
17	StringTie	transcrip	40470992	40490951	.	+	.	transcript_	MSTRG.18694.1	gene_id	MSTRG.18694;
17	StringTie	exon	40470992	40471337	.	+	.	transcript_	MSTRG.18694.1	gene_id	MSTRG.18694;
17	StringTie	exon	40490888	40490951	.	+	.	transcript_	MSTRG.18694.1	gene_id	MSTRG.18694;
17	StringTie	transcrip	43073091	43077004	.	+	.	transcript_	MSTRG.18750.1	gene_id	MSTRG.18750;
17	StringTie	exon	43073091	43073872	.	+	.	transcript_	MSTRG.18750.1	gene_id	MSTRG.18750;
17	StringTie	exon	43076442	43077004	.	+	.	transcript_	MSTRG.18750.1	gene_id	MSTRG.18750;
17	StringTie	transcrip	46677450	46678455	.	+	.	transcript_	MSTRG.18817.1	gene_id	MSTRG.18817;
17	StringTie	exon	46677450	46677759	.	+	.	transcript_	MSTRG.18817.1	gene_id	MSTRG.18817;
17	StringTie	exon	46678042	46678455	.	+	.	transcript_	MSTRG.18817.1	gene_id	MSTRG.18817;
17	StringTie	transcrip	51451495	51452415	.	+	.	transcript_	MSTRG.18924.5	gene_id	MSTRG.18924;
17	StringTie	exon	51451495	51452415	.	+	.	transcript_	MSTRG.18924.5	gene_id	MSTRG.18924;
17	StringTie	transcrip	51473358	51476011	.	+	.	transcript_	MSTRG.18924.6	gene_id	MSTRG.18924;
17	StringTie	exon	51473358	51473434	.	+	.	transcript_	MSTRG.18924.6	gene_id	MSTRG.18924;
17	StringTie	exon	51473509	51476011	.	+	.	transcript_	MSTRG.18924.6	gene_id	MSTRG.18924;
17	StringTie	transcrip	51644232	51647726	.	+	.	transcript_	MSTRG.18939.1	gene_id	MSTRG.18939;
17	StringTie	exon	51644232	51644271	.	+	.	transcript_	MSTRG.18939.1	gene_id	MSTRG.18939;
17	StringTie	exon	51647478	51647726	.	+	.	transcript_	MSTRG.18939.1	gene_id	MSTRG.18939;
17	StringTie	transcrip	51698163	51712277	.	+	.	transcript_	MSTRG.18943.1	gene_id	MSTRG.18943;
17	StringTie	exon	51698163	51698590	.	+	.	transcript_	MSTRG.18943.1	gene_id	MSTRG.18943;
17	StringTie	exon	51712145	51712277	.	+	.	transcript_	MSTRG.18943.1	gene_id	MSTRG.18943;
17	StringTie	transcrip	57984759	57985906	.	+	.	transcript_	MSTRG.18997.1	gene_id	MSTRG.18997;
17	StringTie	exon	57984759	57985129	.	+	.	transcript_	MSTRG.18997.1	gene_id	MSTRG.18997;
17	StringTie	exon	57985872	57985906	.	+	.	transcript_	MSTRG.18997.1	gene_id	MSTRG.18997;
17	StringTie	transcrip	61936379	61937190	.	+	.	transcript_	MSTRG.19052.1	gene_id	MSTRG.19052;
17	StringTie	exon	61936379	61936436	.	+	.	transcript_	MSTRG.19052.1	gene_id	MSTRG.19052;
17	StringTie	exon	61936974	61937190	.	+	.	transcript_	MSTRG.19052.1	gene_id	MSTRG.19052;

17	StringTie	transcrip	62423739	62424100	.	+	.	transcript_	MSTRG.19074.:	gene_id	MSTRG.19074;
17	StringTie	exon	62423739	62423819	.	+	.	transcript_	MSTRG.19074.:	gene_id	MSTRG.19074;
17	StringTie	exon	62423846	62423948	.	+	.	transcript_	MSTRG.19074.:	gene_id	MSTRG.19074;
17	StringTie	exon	62424000	62424100	.	+	.	transcript_	MSTRG.19074.:	gene_id	MSTRG.19074;
17	StringTie	transcrip	62517874	62518942	.	+	.	transcript_	MSTRG.19077.:	gene_id	MSTRG.19077;
17	StringTie	exon	62517874	62517969	.	+	.	transcript_	MSTRG.19077.:	gene_id	MSTRG.19077;
17	StringTie	exon	62518832	62518942	.	+	.	transcript_	MSTRG.19077.:	gene_id	MSTRG.19077;
17	StringTie	transcrip	62793419	62795100	.	+	.	transcript_	MSTRG.19097.:	gene_id	MSTRG.19097;
17	StringTie	exon	62793419	62793519	.	+	.	transcript_	MSTRG.19097.:	gene_id	MSTRG.19097;
17	StringTie	exon	62794814	62795100	.	+	.	transcript_	MSTRG.19097.:	gene_id	MSTRG.19097;
17	StringTie	transcrip	1486367	1491859	.	-	.	transcript_	MSTRG.18080.:	gene_id	MSTRG.18080;
17	StringTie	exon	1486367	1486552	.	-	.	transcript_	MSTRG.18080.:	gene_id	MSTRG.18080;
17	StringTie	exon	1488844	1491859	.	-	.	transcript_	MSTRG.18080.:	gene_id	MSTRG.18080;
17	StringTie	transcrip	15051967	15070278	.	-	.	transcript_	MSTRG.18206.:	gene_id	MSTRG.18206;
17	StringTie	exon	15051967	15052085	.	-	.	transcript_	MSTRG.18206.:	gene_id	MSTRG.18206;
17	StringTie	exon	15068055	15068120	.	-	.	transcript_	MSTRG.18206.:	gene_id	MSTRG.18206;
17	StringTie	exon	15070185	15070278	.	-	.	transcript_	MSTRG.18206.:	gene_id	MSTRG.18206;
17	StringTie	transcrip	32954227	32954973	.	-	.	transcript_	MSTRG.18461.:	gene_id	MSTRG.18461;
17	StringTie	exon	32954227	32954282	.	-	.	transcript_	MSTRG.18461.:	gene_id	MSTRG.18461;
17	StringTie	exon	32954693	32954973	.	-	.	transcript_	MSTRG.18461.:	gene_id	MSTRG.18461;
17	StringTie	transcrip	33897285	33907472	.	-	.	transcript_	MSTRG.18486.:	gene_id	MSTRG.18486;
17	StringTie	exon	33897285	33898353	.	-	.	transcript_	MSTRG.18486.:	gene_id	MSTRG.18486;
17	StringTie	exon	33902944	33903265	.	-	.	transcript_	MSTRG.18486.:	gene_id	MSTRG.18486;
17	StringTie	exon	33907032	33907472	.	-	.	transcript_	MSTRG.18486.:	gene_id	MSTRG.18486;
17	StringTie	transcrip	33962968	33971796	.	-	.	transcript_	MSTRG.18495.:	gene_id	MSTRG.18495;
17	StringTie	exon	33962968	33970654	.	-	.	transcript_	MSTRG.18495.:	gene_id	MSTRG.18495;
17	StringTie	exon	33971659	33971796	.	-	.	transcript_	MSTRG.18495.:	gene_id	MSTRG.18495;
17	StringTie	transcrip	35216410	35218138	.	-	.	transcript_	MSTRG.18531.:	gene_id	MSTRG.18531;
17	StringTie	exon	35216410	35217230	.	-	.	transcript_	MSTRG.18531.:	gene_id	MSTRG.18531;
17	StringTie	exon	35217518	35218138	.	-	.	transcript_	MSTRG.18531.:	gene_id	MSTRG.18531;
17	StringTie	transcrip	37285558	37287055	.	-	.	transcript_	MSTRG.18605.:	gene_id	MSTRG.18605;
17	StringTie	exon	37285558	37285668	.	-	.	transcript_	MSTRG.18605.:	gene_id	MSTRG.18605;
17	StringTie	exon	37286387	37287055	.	-	.	transcript_	MSTRG.18605.:	gene_id	MSTRG.18605;
17	StringTie	transcrip	37907527	37912089	.	-	.	transcript_	MSTRG.18622.:	gene_id	MSTRG.18622;
17	StringTie	exon	37907527	37907622	.	-	.	transcript_	MSTRG.18622.:	gene_id	MSTRG.18622;

17	StringTie	exon	37910977	37912089	.	-	.	transcript_	MSTRG.18622.:	gene_id	MSTRG.18622;
17	StringTie	transcrip	38517315	38632458	.	-	.	transcript_	MSTRG.18633.:	gene_id	MSTRG.18633;
17	StringTie	exon	38517315	38518099	.	-	.	transcript_	MSTRG.18633.:	gene_id	MSTRG.18633;
17	StringTie	exon	38518203	38518421	.	-	.	transcript_	MSTRG.18633.:	gene_id	MSTRG.18633;
17	StringTie	exon	38525792	38525874	.	-	.	transcript_	MSTRG.18633.:	gene_id	MSTRG.18633;
17	StringTie	exon	38596259	38596369	.	-	.	transcript_	MSTRG.18633.:	gene_id	MSTRG.18633;
17	StringTie	exon	38632320	38632458	.	-	.	transcript_	MSTRG.18633.:	gene_id	MSTRG.18633;
17	StringTie	transcrip	45962707	45966898	.	-	.	transcript_	MSTRG.18789.:	gene_id	MSTRG.18789;
17	StringTie	exon	45962707	45962970	.	-	.	transcript_	MSTRG.18789.:	gene_id	MSTRG.18789;
17	StringTie	exon	45966825	45966898	.	-	.	transcript_	MSTRG.18789.:	gene_id	MSTRG.18789;
17	StringTie	transcrip	47080562	47081260	.	-	.	transcript_	MSTRG.18828.:	gene_id	MSTRG.18828;
17	StringTie	exon	47080562	47080794	.	-	.	transcript_	MSTRG.18828.:	gene_id	MSTRG.18828;
17	StringTie	exon	47081211	47081260	.	-	.	transcript_	MSTRG.18828.:	gene_id	MSTRG.18828;
17	StringTie	transcrip	50574081	50578835	.	-	.	transcript_	MSTRG.18867.:	gene_id	MSTRG.18867;
17	StringTie	exon	50574081	50574338	.	-	.	transcript_	MSTRG.18867.:	gene_id	MSTRG.18867;
17	StringTie	exon	50575089	50575181	.	-	.	transcript_	MSTRG.18867.:	gene_id	MSTRG.18867;
17	StringTie	exon	50578355	50578835	.	-	.	transcript_	MSTRG.18867.:	gene_id	MSTRG.18867;
17	StringTie	transcrip	51472046	51473365	.	-	.	transcript_	MSTRG.18927.:	gene_id	MSTRG.18927;
17	StringTie	exon	51472046	51473199	.	-	.	transcript_	MSTRG.18927.:	gene_id	MSTRG.18927;
17	StringTie	exon	51473276	51473365	.	-	.	transcript_	MSTRG.18927.:	gene_id	MSTRG.18927;
17	StringTie	transcrip	54865781	54866298	.	-	.	transcript_	MSTRG.18972.:	gene_id	MSTRG.18972;
17	StringTie	exon	54865781	54865941	.	-	.	transcript_	MSTRG.18972.:	gene_id	MSTRG.18972;
17	StringTie	exon	54866164	54866298	.	-	.	transcript_	MSTRG.18972.:	gene_id	MSTRG.18972;
18	StringTie	transcrip	6155909	6156491	.	+	.	transcript_	MSTRG.19197.:	gene_id	MSTRG.19197;
18	StringTie	exon	6155909	6156264	.	+	.	transcript_	MSTRG.19197.:	gene_id	MSTRG.19197;
18	StringTie	exon	6156473	6156491	.	+	.	transcript_	MSTRG.19197.:	gene_id	MSTRG.19197;
18	StringTie	transcrip	13777579	13784379	.	+	.	transcript_	MSTRG.19384.:	gene_id	MSTRG.19384;
18	StringTie	exon	13777579	13777617	.	+	.	transcript_	MSTRG.19384.:	gene_id	MSTRG.19384;
18	StringTie	exon	13783661	13784379	.	+	.	transcript_	MSTRG.19384.:	gene_id	MSTRG.19384;
18	StringTie	transcrip	14279634	14281980	.	+	.	transcript_	MSTRG.19399.:	gene_id	MSTRG.19399;
18	StringTie	exon	14279634	14280898	.	+	.	transcript_	MSTRG.19399.:	gene_id	MSTRG.19399;
18	StringTie	exon	14281183	14281980	.	+	.	transcript_	MSTRG.19399.:	gene_id	MSTRG.19399;
18	StringTie	transcrip	14785344	14786536	.	+	.	transcript_	MSTRG.19400.:	gene_id	MSTRG.19400;
18	StringTie	exon	14785344	14785561	.	+	.	transcript_	MSTRG.19400.:	gene_id	MSTRG.19400;
18	StringTie	exon	14786282	14786536	.	+	.	transcript_	MSTRG.19400.:	gene_id	MSTRG.19400;

18	StringTie	transcrip	15131912	15140257	.	+	.	transcript_	MSTRG.19425.[illegible]	gene_id	MSTRG.19425;
18	StringTie	exon	15131912	15131991	.	+	.	transcript_	MSTRG.19425.[illegible]	gene_id	MSTRG.19425;
18	StringTie	exon	15139889	15140257	.	+	.	transcript_	MSTRG.19425.[illegible]	gene_id	MSTRG.19425;
18	StringTie	transcrip	17859078	17947544	.	+	.	transcript_	MSTRG.19467.[illegible]	gene_id	MSTRG.19467;
18	StringTie	exon	17859078	17859173	.	+	.	transcript_	MSTRG.19467.[illegible]	gene_id	MSTRG.19467;
18	StringTie	exon	17911857	17912006	.	+	.	transcript_	MSTRG.19467.[illegible]	gene_id	MSTRG.19467;
18	StringTie	exon	17947099	17947544	.	+	.	transcript_	MSTRG.19467.[illegible]	gene_id	MSTRG.19467;
18	StringTie	transcrip	17872880	17947954	.	+	.	transcript_	MSTRG.19467.[illegible]	gene_id	MSTRG.19467;
18	StringTie	exon	17872880	17872965	.	+	.	transcript_	MSTRG.19467.[illegible]	gene_id	MSTRG.19467;
18	StringTie	exon	17889683	17889795	.	+	.	transcript_	MSTRG.19467.[illegible]	gene_id	MSTRG.19467;
18	StringTie	exon	17911857	17912006	.	+	.	transcript_	MSTRG.19467.[illegible]	gene_id	MSTRG.19467;
18	StringTie	exon	17947099	17947954	.	+	.	transcript_	MSTRG.19467.[illegible]	gene_id	MSTRG.19467;
18	StringTie	transcrip	18029682	18032794	.	+	.	transcript_	MSTRG.19467.[illegible]	gene_id	MSTRG.19467;
18	StringTie	exon	18029682	18030833	.	+	.	transcript_	MSTRG.19467.[illegible]	gene_id	MSTRG.19467;
18	StringTie	exon	18031451	18032794	.	+	.	transcript_	MSTRG.19467.[illegible]	gene_id	MSTRG.19467;
18	StringTie	transcrip	18677663	18681859	.	+	.	transcript_	MSTRG.19490.[illegible]	gene_id	MSTRG.19490;
18	StringTie	exon	18677663	18677686	.	+	.	transcript_	MSTRG.19490.[illegible]	gene_id	MSTRG.19490;
18	StringTie	exon	18681287	18681859	.	+	.	transcript_	MSTRG.19490.[illegible]	gene_id	MSTRG.19490;
18	StringTie	transcrip	21924956	21944322	.	+	.	transcript_	MSTRG.19562.[illegible]	gene_id	MSTRG.19562;
18	StringTie	exon	21924956	21924976	.	+	.	transcript_	MSTRG.19562.[illegible]	gene_id	MSTRG.19562;
18	StringTie	exon	21931936	21932019	.	+	.	transcript_	MSTRG.19562.[illegible]	gene_id	MSTRG.19562;
18	StringTie	exon	21938845	21938926	.	+	.	transcript_	MSTRG.19562.[illegible]	gene_id	MSTRG.19562;
18	StringTie	exon	21944203	21944322	.	+	.	transcript_	MSTRG.19562.[illegible]	gene_id	MSTRG.19562;
18	StringTie	transcrip	21938772	22018357	.	+	.	transcript_	MSTRG.19562.[illegible]	gene_id	MSTRG.19562;
18	StringTie	exon	21938772	21938926	.	+	.	transcript_	MSTRG.19562.[illegible]	gene_id	MSTRG.19562;
18	StringTie	exon	21947846	21947905	.	+	.	transcript_	MSTRG.19562.[illegible]	gene_id	MSTRG.19562;
18	StringTie	exon	22014983	22015012	.	+	.	transcript_	MSTRG.19562.[illegible]	gene_id	MSTRG.19562;
18	StringTie	exon	22016439	22016548	.	+	.	transcript_	MSTRG.19562.[illegible]	gene_id	MSTRG.19562;
18	StringTie	exon	22018241	22018357	.	+	.	transcript_	MSTRG.19562.[illegible]	gene_id	MSTRG.19562;
18	StringTie	transcrip	23798285	23803301	.	+	.	transcript_	MSTRG.19589.[illegible]	gene_id	MSTRG.19589;
18	StringTie	exon	23798285	23798527	.	+	.	transcript_	MSTRG.19589.[illegible]	gene_id	MSTRG.19589;
18	StringTie	exon	23798593	23803301	.	+	.	transcript_	MSTRG.19589.[illegible]	gene_id	MSTRG.19589;
18	StringTie	transcrip	25644202	25669940	.	+	.	transcript_	MSTRG.19600.[illegible]	gene_id	MSTRG.19600;
18	StringTie	exon	25644202	25644226	.	+	.	transcript_	MSTRG.19600.[illegible]	gene_id	MSTRG.19600;
18	StringTie	exon	25669612	25669940	.	+	.	transcript_	MSTRG.19600.[illegible]	gene_id	MSTRG.19600;

18	StringTie	transcrip	32644890	32651939	.	+	.	transcript_	MSTRG.19750.1	gene_id	MSTRG.19750;
18	StringTie	exon	32644890	32645024	.	+	.	transcript_	MSTRG.19750.1	gene_id	MSTRG.19750;
18	StringTie	exon	32645736	32645889	.	+	.	transcript_	MSTRG.19750.1	gene_id	MSTRG.19750;
18	StringTie	exon	32649056	32649135	.	+	.	transcript_	MSTRG.19750.1	gene_id	MSTRG.19750;
18	StringTie	exon	32651214	32651939	.	+	.	transcript_	MSTRG.19750.1	gene_id	MSTRG.19750;
18	StringTie	transcrip	33212737	33214468	.	+	.	transcript_	MSTRG.19765.1	gene_id	MSTRG.19765;
18	StringTie	exon	33212737	33214025	.	+	.	transcript_	MSTRG.19765.1	gene_id	MSTRG.19765;
18	StringTie	exon	33214079	33214468	.	+	.	transcript_	MSTRG.19765.1	gene_id	MSTRG.19765;
18	StringTie	transcrip	39028826	39029342	.	+	.	transcript_	MSTRG.19927.1	gene_id	MSTRG.19927;
18	StringTie	exon	39028826	39028930	.	+	.	transcript_	MSTRG.19927.1	gene_id	MSTRG.19927;
18	StringTie	exon	39029070	39029342	.	+	.	transcript_	MSTRG.19927.1	gene_id	MSTRG.19927;
18	StringTie	transcrip	39031476	39040047	.	+	.	transcript_	MSTRG.19928.1	gene_id	MSTRG.19928;
18	StringTie	exon	39031476	39031659	.	+	.	transcript_	MSTRG.19928.1	gene_id	MSTRG.19928;
18	StringTie	exon	39039670	39040047	.	+	.	transcript_	MSTRG.19928.1	gene_id	MSTRG.19928;
18	StringTie	transcrip	39054178	39060757	.	+	.	transcript_	MSTRG.19934.1	gene_id	MSTRG.19934;
18	StringTie	exon	39054178	39054215	.	+	.	transcript_	MSTRG.19934.1	gene_id	MSTRG.19934;
18	StringTie	exon	39055616	39055818	.	+	.	transcript_	MSTRG.19934.1	gene_id	MSTRG.19934;
18	StringTie	exon	39060718	39060757	.	+	.	transcript_	MSTRG.19934.1	gene_id	MSTRG.19934;
18	StringTie	transcrip	39070672	39073096	.	+	.	transcript_	MSTRG.19938.1	gene_id	MSTRG.19938;
18	StringTie	exon	39070672	39070960	.	+	.	transcript_	MSTRG.19938.1	gene_id	MSTRG.19938;
18	StringTie	exon	39072494	39072806	.	+	.	transcript_	MSTRG.19938.1	gene_id	MSTRG.19938;
18	StringTie	exon	39073053	39073096	.	+	.	transcript_	MSTRG.19938.1	gene_id	MSTRG.19938;
18	StringTie	transcrip	39082439	39084247	.	+	.	transcript_	MSTRG.19940.1	gene_id	MSTRG.19940;
18	StringTie	exon	39082439	39082574	.	+	.	transcript_	MSTRG.19940.1	gene_id	MSTRG.19940;
18	StringTie	exon	39083668	39084247	.	+	.	transcript_	MSTRG.19940.1	gene_id	MSTRG.19940;
18	StringTie	transcrip	41795351	41799200	.	+	.	transcript_	MSTRG.19965.2	gene_id	MSTRG.19965;
18	StringTie	exon	41795351	41795407	.	+	.	transcript_	MSTRG.19965.2	gene_id	MSTRG.19965;
18	StringTie	exon	41798854	41799200	.	+	.	transcript_	MSTRG.19965.2	gene_id	MSTRG.19965;
18	StringTie	transcrip	42227776	42235420	.	+	.	transcript_	MSTRG.19979.1	gene_id	MSTRG.19979;
18	StringTie	exon	42227776	42227865	.	+	.	transcript_	MSTRG.19979.1	gene_id	MSTRG.19979;
18	StringTie	exon	42234838	42235420	.	+	.	transcript_	MSTRG.19979.1	gene_id	MSTRG.19979;
18	StringTie	transcrip	42323043	42324069	.	+	.	transcript_	MSTRG.19990.1	gene_id	MSTRG.19990;
18	StringTie	exon	42323043	42323153	.	+	.	transcript_	MSTRG.19990.1	gene_id	MSTRG.19990;
18	StringTie	exon	42323973	42324069	.	+	.	transcript_	MSTRG.19990.1	gene_id	MSTRG.19990;
18	StringTie	transcrip	42352237	42355703	.	+	.	transcript_	MSTRG.19991.2	gene_id	MSTRG.19991;

18	StringTie	exon	42352237	42353399	.	+	.	transcript_	MSTRG.19991.:	gene_id	MSTRG.19991;
18	StringTie	exon	42355518	42355703	.	+	.	transcript_	MSTRG.19991.:	gene_id	MSTRG.19991;
18	StringTie	transcrip	43071690	43244511	.	+	.	transcript_	MSTRG.20010.	gene_id	MSTRG.20010;
18	StringTie	exon	43071690	43072319	.	+	.	transcript_	MSTRG.20010.	gene_id	MSTRG.20010;
18	StringTie	exon	43073096	43073239	.	+	.	transcript_	MSTRG.20010.	gene_id	MSTRG.20010;
18	StringTie	exon	43244453	43244511	.	+	.	transcript_	MSTRG.20010.	gene_id	MSTRG.20010;
18	StringTie	transcrip	46515648	46518229	.	+	.	transcript_	MSTRG.20078.	gene_id	MSTRG.20078;
18	StringTie	exon	46515648	46515855	.	+	.	transcript_	MSTRG.20078.	gene_id	MSTRG.20078;
18	StringTie	exon	46518153	46518229	.	+	.	transcript_	MSTRG.20078.	gene_id	MSTRG.20078;
18	StringTie	transcrip	49251182	49251940	.	+	.	transcript_	MSTRG.20120.	gene_id	MSTRG.20120;
18	StringTie	exon	49251182	49251221	.	+	.	transcript_	MSTRG.20120.	gene_id	MSTRG.20120;
18	StringTie	exon	49251582	49251940	.	+	.	transcript_	MSTRG.20120.	gene_id	MSTRG.20120;
18	StringTie	transcrip	50320447	50325416	.	+	.	transcript_	MSTRG.20146.:	gene_id	MSTRG.20146;
18	StringTie	exon	50320447	50320497	.	+	.	transcript_	MSTRG.20146.:	gene_id	MSTRG.20146;
18	StringTie	exon	50325217	50325416	.	+	.	transcript_	MSTRG.20146.:	gene_id	MSTRG.20146;
18	StringTie	transcrip	50320447	50325437	.	+	.	transcript_	MSTRG.20146.	gene_id	MSTRG.20146;
18	StringTie	exon	50320447	50320566	.	+	.	transcript_	MSTRG.20146.	gene_id	MSTRG.20146;
18	StringTie	exon	50325286	50325437	.	+	.	transcript_	MSTRG.20146.	gene_id	MSTRG.20146;
18	StringTie	transcrip	13633005	13636017	.	-	.	transcript_	MSTRG.19382.	gene_id	MSTRG.19382;
18	StringTie	exon	13633005	13633226	.	-	.	transcript_	MSTRG.19382.	gene_id	MSTRG.19382;
18	StringTie	exon	13633509	13633602	.	-	.	transcript_	MSTRG.19382.	gene_id	MSTRG.19382;
18	StringTie	exon	13635933	13636017	.	-	.	transcript_	MSTRG.19382.	gene_id	MSTRG.19382;
18	StringTie	transcrip	17687165	17692425	.	-	.	transcript_	MSTRG.19466.(	gene_id	MSTRG.19466;
18	StringTie	exon	17687165	17688699	.	-	.	transcript_	MSTRG.19466.(	gene_id	MSTRG.19466;
18	StringTie	exon	17692399	17692425	.	-	.	transcript_	MSTRG.19466.(	gene_id	MSTRG.19466;
18	StringTie	transcrip	19120667	19136439	.	-	.	transcript_	MSTRG.19505.	gene_id	MSTRG.19505;
18	StringTie	exon	19120667	19120746	.	-	.	transcript_	MSTRG.19505.	gene_id	MSTRG.19505;
18	StringTie	exon	19135906	19136439	.	-	.	transcript_	MSTRG.19505.	gene_id	MSTRG.19505;
18	StringTie	transcrip	23710348	23711465	.	-	.	transcript_	MSTRG.19580.	gene_id	MSTRG.19580;
18	StringTie	exon	23710348	23710629	.	-	.	transcript_	MSTRG.19580.	gene_id	MSTRG.19580;
18	StringTie	exon	23711435	23711465	.	-	.	transcript_	MSTRG.19580.	gene_id	MSTRG.19580;
18	StringTie	transcrip	31806970	31816996	.	-	.	transcript_	MSTRG.19730.	gene_id	MSTRG.19730;
18	StringTie	exon	31806970	31807173	.	-	.	transcript_	MSTRG.19730.	gene_id	MSTRG.19730;
18	StringTie	exon	31816946	31816996	.	-	.	transcript_	MSTRG.19730.	gene_id	MSTRG.19730;
18	StringTie	transcrip	37168498	37190394	.	-	.	transcript_	MSTRG.19870.	gene_id	MSTRG.19870;

18	StringTie	exon	37168498	37168527	.	-	.	transcript_	MSTRG.19870.1	gene_id	MSTRG.19870;
18	StringTie	exon	37189906	37190394	.	-	.	transcript_	MSTRG.19870.1	gene_id	MSTRG.19870;
18	StringTie	transcrip	38199925	38284410	.	-	.	transcript_	MSTRG.19901.1	gene_id	MSTRG.19901;
18	StringTie	exon	38199925	38201856	.	-	.	transcript_	MSTRG.19901.1	gene_id	MSTRG.19901;
18	StringTie	exon	38254377	38254563	.	-	.	transcript_	MSTRG.19901.1	gene_id	MSTRG.19901;
18	StringTie	exon	38282788	38282849	.	-	.	transcript_	MSTRG.19901.1	gene_id	MSTRG.19901;
18	StringTie	exon	38284132	38284410	.	-	.	transcript_	MSTRG.19901.1	gene_id	MSTRG.19901;
18	StringTie	transcrip	38200970	38225537	.	-	.	transcript_	MSTRG.19901.2	gene_id	MSTRG.19901;
18	StringTie	exon	38200970	38201856	.	-	.	transcript_	MSTRG.19901.2	gene_id	MSTRG.19901;
18	StringTie	exon	38225388	38225537	.	-	.	transcript_	MSTRG.19901.2	gene_id	MSTRG.19901;
18	StringTie	transcrip	38237686	38284508	.	-	.	transcript_	MSTRG.19901.3	gene_id	MSTRG.19901;
18	StringTie	exon	38237686	38237794	.	-	.	transcript_	MSTRG.19901.3	gene_id	MSTRG.19901;
18	StringTie	exon	38254377	38254563	.	-	.	transcript_	MSTRG.19901.3	gene_id	MSTRG.19901;
18	StringTie	exon	38282788	38282849	.	-	.	transcript_	MSTRG.19901.3	gene_id	MSTRG.19901;
18	StringTie	exon	38284132	38284508	.	-	.	transcript_	MSTRG.19901.3	gene_id	MSTRG.19901;
18	StringTie	transcrip	43666642	43728019	.	-	.	transcript_	MSTRG.20020.1	gene_id	MSTRG.20020;
18	StringTie	exon	43666642	43667927	.	-	.	transcript_	MSTRG.20020.1	gene_id	MSTRG.20020;
18	StringTie	exon	43669691	43669867	.	-	.	transcript_	MSTRG.20020.1	gene_id	MSTRG.20020;
18	StringTie	exon	43674347	43674576	.	-	.	transcript_	MSTRG.20020.1	gene_id	MSTRG.20020;
18	StringTie	exon	43727908	43728019	.	-	.	transcript_	MSTRG.20020.1	gene_id	MSTRG.20020;
18	StringTie	transcrip	43667088	43728061	.	-	.	transcript_	MSTRG.20020.2	gene_id	MSTRG.20020;
18	StringTie	exon	43667088	43667927	.	-	.	transcript_	MSTRG.20020.2	gene_id	MSTRG.20020;
18	StringTie	exon	43674347	43674576	.	-	.	transcript_	MSTRG.20020.2	gene_id	MSTRG.20020;
18	StringTie	exon	43727908	43728061	.	-	.	transcript_	MSTRG.20020.2	gene_id	MSTRG.20020;
18	StringTie	transcrip	43667095	43727189	.	-	.	transcript_	MSTRG.20020.3	gene_id	MSTRG.20020;
18	StringTie	exon	43667095	43667927	.	-	.	transcript_	MSTRG.20020.3	gene_id	MSTRG.20020;
18	StringTie	exon	43669691	43669867	.	-	.	transcript_	MSTRG.20020.3	gene_id	MSTRG.20020;
18	StringTie	exon	43674347	43674576	.	-	.	transcript_	MSTRG.20020.3	gene_id	MSTRG.20020;
18	StringTie	exon	43691359	43691481	.	-	.	transcript_	MSTRG.20020.3	gene_id	MSTRG.20020;
18	StringTie	exon	43727152	43727189	.	-	.	transcript_	MSTRG.20020.3	gene_id	MSTRG.20020;
18	StringTie	transcrip	43667095	43728019	.	-	.	transcript_	MSTRG.20020.4	gene_id	MSTRG.20020;
18	StringTie	exon	43667095	43667927	.	-	.	transcript_	MSTRG.20020.4	gene_id	MSTRG.20020;
18	StringTie	exon	43674347	43674576	.	-	.	transcript_	MSTRG.20020.4	gene_id	MSTRG.20020;
18	StringTie	exon	43691359	43691481	.	-	.	transcript_	MSTRG.20020.4	gene_id	MSTRG.20020;
18	StringTie	exon	43727908	43728019	.	-	.	transcript_	MSTRG.20020.4	gene_id	MSTRG.20020;

18	StringTie	transcrip	43667138	43728019	.	-	.	transcript_	MSTRG.20020.5	gene_id	MSTRG.20020;
18	StringTie	exon	43667138	43667927	.	-	.	transcript_	MSTRG.20020.5	gene_id	MSTRG.20020;
18	StringTie	exon	43691359	43691481	.	-	.	transcript_	MSTRG.20020.5	gene_id	MSTRG.20020;
18	StringTie	exon	43727908	43728019	.	-	.	transcript_	MSTRG.20020.5	gene_id	MSTRG.20020;
18	StringTie	transcrip	43679080	43728017	.	-	.	transcript_	MSTRG.20020.6	gene_id	MSTRG.20020;
18	StringTie	exon	43679080	43680122	.	-	.	transcript_	MSTRG.20020.6	gene_id	MSTRG.20020;
18	StringTie	exon	43691359	43691481	.	-	.	transcript_	MSTRG.20020.6	gene_id	MSTRG.20020;
18	StringTie	exon	43727908	43728017	.	-	.	transcript_	MSTRG.20020.6	gene_id	MSTRG.20020;
18	StringTie	transcrip	43882424	43899957	.	-	.	transcript_	MSTRG.20023.1	gene_id	MSTRG.20023;
18	StringTie	exon	43882424	43882470	.	-	.	transcript_	MSTRG.20023.1	gene_id	MSTRG.20023;
18	StringTie	exon	43899470	43899957	.	-	.	transcript_	MSTRG.20023.1	gene_id	MSTRG.20023;
18	StringTie	transcrip	46788927	46795351	.	-	.	transcript_	MSTRG.20084.1	gene_id	MSTRG.20084;
18	StringTie	exon	46788927	46789938	.	-	.	transcript_	MSTRG.20084.1	gene_id	MSTRG.20084;
18	StringTie	exon	46794969	46795351	.	-	.	transcript_	MSTRG.20084.1	gene_id	MSTRG.20084;
18	StringTie	transcrip	48694517	48710183	.	-	.	transcript_	MSTRG.20106.1	gene_id	MSTRG.20106;
18	StringTie	exon	48694517	48696499	.	-	.	transcript_	MSTRG.20106.1	gene_id	MSTRG.20106;
18	StringTie	exon	48710075	48710183	.	-	.	transcript_	MSTRG.20106.1	gene_id	MSTRG.20106;
18	StringTie	transcrip	48694752	48710176	.	-	.	transcript_	MSTRG.20106.2	gene_id	MSTRG.20106;
18	StringTie	exon	48694752	48696499	.	-	.	transcript_	MSTRG.20106.2	gene_id	MSTRG.20106;
18	StringTie	exon	48701620	48701886	.	-	.	transcript_	MSTRG.20106.2	gene_id	MSTRG.20106;
18	StringTie	exon	48710075	48710176	.	-	.	transcript_	MSTRG.20106.2	gene_id	MSTRG.20106;
18	StringTie	transcrip	48710827	48712722	.	-	.	transcript_	MSTRG.20107.1	gene_id	MSTRG.20107;
18	StringTie	exon	48710827	48710937	.	-	.	transcript_	MSTRG.20107.1	gene_id	MSTRG.20107;
18	StringTie	exon	48712615	48712722	.	-	.	transcript_	MSTRG.20107.1	gene_id	MSTRG.20107;
18	StringTie	transcrip	48741583	48803915	.	-	.	transcript_	MSTRG.20111.1	gene_id	MSTRG.20111;
18	StringTie	exon	48741583	48741655	.	-	.	transcript_	MSTRG.20111.1	gene_id	MSTRG.20111;
18	StringTie	exon	48802735	48803915	.	-	.	transcript_	MSTRG.20111.1	gene_id	MSTRG.20111;
18	StringTie	transcrip	48819205	48825482	.	-	.	transcript_	MSTRG.20101.1	gene_id	MSTRG.20101;
18	StringTie	exon	48819205	48819586	.	-	.	transcript_	MSTRG.20101.1	gene_id	MSTRG.20101;
18	StringTie	exon	48825320	48825482	.	-	.	transcript_	MSTRG.20101.1	gene_id	MSTRG.20101;
18	StringTie	transcrip	48819904	48824519	.	-	.	transcript_	MSTRG.20102.1	gene_id	MSTRG.20102;
18	StringTie	exon	48819904	48820143	.	-	.	transcript_	MSTRG.20102.1	gene_id	MSTRG.20102;
18	StringTie	exon	48824194	48824519	.	-	.	transcript_	MSTRG.20102.1	gene_id	MSTRG.20102;
18	StringTie	transcrip	50356834	50366019	.	-	.	transcript_	MSTRG.20147.1	gene_id	MSTRG.20147;
18	StringTie	exon	50356834	50357166	.	-	.	transcript_	MSTRG.20147.1	gene_id	MSTRG.20147;

18	StringTie	exon	50365934	50366019	.	-	.	transcript_	MSTRG.20147.1	gene_id	MSTRG.20147;
18	StringTie	transcrip	50480853	50485285	.	-	.	transcript_	MSTRG.20158.1	gene_id	MSTRG.20158;
18	StringTie	exon	50480853	50481275	.	-	.	transcript_	MSTRG.20158.1	gene_id	MSTRG.20158;
18	StringTie	exon	50484926	50485285	.	-	.	transcript_	MSTRG.20158.1	gene_id	MSTRG.20158;
18	StringTie	transcrip	50481212	50485181	.	-	.	transcript_	MSTRG.20158.2	gene_id	MSTRG.20158;
18	StringTie	exon	50481212	50481275	.	-	.	transcript_	MSTRG.20158.2	gene_id	MSTRG.20158;
18	StringTie	exon	50484751	50484854	.	-	.	transcript_	MSTRG.20158.2	gene_id	MSTRG.20158;
18	StringTie	exon	50484926	50485181	.	-	.	transcript_	MSTRG.20158.2	gene_id	MSTRG.20158;
2	StringTie	transcrip	129324	135096	.	+	.	transcript_	MSTRG.20264.1	gene_id	MSTRG.20264;
2	StringTie	exon	129324	129629	.	+	.	transcript_	MSTRG.20264.1	gene_id	MSTRG.20264;
2	StringTie	exon	134992	135096	.	+	.	transcript_	MSTRG.20264.1	gene_id	MSTRG.20264;
2	StringTie	transcrip	540300	545389	.	+	.	transcript_	MSTRG.20301.1	gene_id	MSTRG.20301;
2	StringTie	exon	540300	540943	.	+	.	transcript_	MSTRG.20301.1	gene_id	MSTRG.20301;
2	StringTie	exon	545198	545389	.	+	.	transcript_	MSTRG.20301.1	gene_id	MSTRG.20301;
2	StringTie	transcrip	1480383	1486764	.	+	.	transcript_	MSTRG.20317.1	gene_id	MSTRG.20317;
2	StringTie	exon	1480383	1480451	.	+	.	transcript_	MSTRG.20317.1	gene_id	MSTRG.20317;
2	StringTie	exon	1485033	1485088	.	+	.	transcript_	MSTRG.20317.1	gene_id	MSTRG.20317;
2	StringTie	exon	1485734	1486764	.	+	.	transcript_	MSTRG.20317.1	gene_id	MSTRG.20317;
2	StringTie	transcrip	3099313	3138365	.	+	.	transcript_	MSTRG.20368.1	gene_id	MSTRG.20368;
2	StringTie	exon	3099313	3099708	.	+	.	transcript_	MSTRG.20368.1	gene_id	MSTRG.20368;
2	StringTie	exon	3138293	3138365	.	+	.	transcript_	MSTRG.20368.1	gene_id	MSTRG.20368;
2	StringTie	transcrip	4989179	4996961	.	+	.	transcript_	MSTRG.20417.1	gene_id	MSTRG.20417;
2	StringTie	exon	4989179	4989268	.	+	.	transcript_	MSTRG.20417.1	gene_id	MSTRG.20417;
2	StringTie	exon	4995857	4996961	.	+	.	transcript_	MSTRG.20417.1	gene_id	MSTRG.20417;
2	StringTie	transcrip	6166995	6172015	.	+	.	transcript_	MSTRG.20469.1	gene_id	MSTRG.20469;
2	StringTie	exon	6166995	6167152	.	+	.	transcript_	MSTRG.20469.1	gene_id	MSTRG.20469;
2	StringTie	exon	6171715	6172015	.	+	.	transcript_	MSTRG.20469.1	gene_id	MSTRG.20469;
2	StringTie	transcrip	6672014	6672430	.	+	.	transcript_	MSTRG.20503.2	gene_id	MSTRG.20503;
2	StringTie	exon	6672014	6672124	.	+	.	transcript_	MSTRG.20503.2	gene_id	MSTRG.20503;
2	StringTie	exon	6672245	6672430	.	+	.	transcript_	MSTRG.20503.2	gene_id	MSTRG.20503;
2	StringTie	transcrip	6672014	6672430	.	+	.	transcript_	MSTRG.20503.1	gene_id	MSTRG.20503;
2	StringTie	exon	6672014	6672124	.	+	.	transcript_	MSTRG.20503.1	gene_id	MSTRG.20503;
2	StringTie	exon	6672185	6672430	.	+	.	transcript_	MSTRG.20503.1	gene_id	MSTRG.20503;
2	StringTie	transcrip	6750281	6757176	.	+	.	transcript_	MSTRG.20512.2	gene_id	MSTRG.20512;
2	StringTie	exon	6750281	6750449	.	+	.	transcript_	MSTRG.20512.2	gene_id	MSTRG.20512;

2	StringTie	exon	6750507	6757176	.	+	.	transcript_	MSTRG.20512.2	gene_id	MSTRG.20512;
2	StringTie	transcrip	6750281	6757220	.	+	.	transcript_	MSTRG.20512.1	gene_id	MSTRG.20512;
2	StringTie	exon	6750281	6757220	.	+	.	transcript_	MSTRG.20512.1	gene_id	MSTRG.20512;
2	StringTie	transcrip	9320975	9337433	.	+	.	transcript_	MSTRG.20637.1	gene_id	MSTRG.20637;
2	StringTie	exon	9320975	9321045	.	+	.	transcript_	MSTRG.20637.1	gene_id	MSTRG.20637;
2	StringTie	exon	9322964	9323105	.	+	.	transcript_	MSTRG.20637.1	gene_id	MSTRG.20637;
2	StringTie	exon	9335498	9335575	.	+	.	transcript_	MSTRG.20637.1	gene_id	MSTRG.20637;
2	StringTie	exon	9336937	9337433	.	+	.	transcript_	MSTRG.20637.1	gene_id	MSTRG.20637;
2	StringTie	transcrip	11923790	11925690	.	+	.	transcript_	MSTRG.20700.1	gene_id	MSTRG.20700;
2	StringTie	exon	11923790	11924006	.	+	.	transcript_	MSTRG.20700.1	gene_id	MSTRG.20700;
2	StringTie	exon	11924115	11925690	.	+	.	transcript_	MSTRG.20700.1	gene_id	MSTRG.20700;
2	StringTie	transcrip	12279707	12286761	.	+	.	transcript_	MSTRG.20710.1	gene_id	MSTRG.20710;
2	StringTie	exon	12279707	12280206	.	+	.	transcript_	MSTRG.20710.1	gene_id	MSTRG.20710;
2	StringTie	exon	12285713	12286761	.	+	.	transcript_	MSTRG.20710.1	gene_id	MSTRG.20710;
2	StringTie	transcrip	12279715	12286698	.	+	.	transcript_	MSTRG.20710.2	gene_id	MSTRG.20710;
2	StringTie	exon	12279715	12280206	.	+	.	transcript_	MSTRG.20710.2	gene_id	MSTRG.20710;
2	StringTie	exon	12281990	12282152	.	+	.	transcript_	MSTRG.20710.2	gene_id	MSTRG.20710;
2	StringTie	exon	12285713	12286698	.	+	.	transcript_	MSTRG.20710.2	gene_id	MSTRG.20710;
2	StringTie	transcrip	16313623	16315841	.	+	.	transcript_	MSTRG.20819.1	gene_id	MSTRG.20819;
2	StringTie	exon	16313623	16313869	.	+	.	transcript_	MSTRG.20819.1	gene_id	MSTRG.20819;
2	StringTie	exon	16315757	16315841	.	+	.	transcript_	MSTRG.20819.1	gene_id	MSTRG.20819;
2	StringTie	transcrip	16608938	16611170	.	+	.	transcript_	MSTRG.20839.1	gene_id	MSTRG.20839;
2	StringTie	exon	16608938	16609435	.	+	.	transcript_	MSTRG.20839.1	gene_id	MSTRG.20839;
2	StringTie	exon	16609457	16611170	.	+	.	transcript_	MSTRG.20839.1	gene_id	MSTRG.20839;
2	StringTie	transcrip	17821540	17822075	.	+	.	transcript_	MSTRG.20846.1	gene_id	MSTRG.20846;
2	StringTie	exon	17821540	17821708	.	+	.	transcript_	MSTRG.20846.1	gene_id	MSTRG.20846;
2	StringTie	exon	17821854	17822075	.	+	.	transcript_	MSTRG.20846.1	gene_id	MSTRG.20846;
2	StringTie	transcrip	18266399	18267580	.	+	.	transcript_	MSTRG.20868.1	gene_id	MSTRG.20868;
2	StringTie	exon	18266399	18266784	.	+	.	transcript_	MSTRG.20868.1	gene_id	MSTRG.20868;
2	StringTie	exon	18267481	18267580	.	+	.	transcript_	MSTRG.20868.1	gene_id	MSTRG.20868;
2	StringTie	transcrip	24747879	24769486	.	+	.	transcript_	MSTRG.20914.2	gene_id	MSTRG.20914;
2	StringTie	exon	24747879	24748841	.	+	.	transcript_	MSTRG.20914.2	gene_id	MSTRG.20914;
2	StringTie	exon	24768838	24769486	.	+	.	transcript_	MSTRG.20914.2	gene_id	MSTRG.20914;
2	StringTie	transcrip	24748167	24760578	.	+	.	transcript_	MSTRG.20914.3	gene_id	MSTRG.20914;
2	StringTie	exon	24748167	24748841	.	+	.	transcript_	MSTRG.20914.3	gene_id	MSTRG.20914;

2	StringTie	exon	24756292	24756435	.	+	.	transcript_	MSTRG.20914.[illegible]	gene_id	MSTRG.20914;
2	StringTie	exon	24758311	24760578	.	+	.	transcript_	MSTRG.20914.[illegible]	gene_id	MSTRG.20914;
2	StringTie	transcrip	24764310	24767302	.	+	.	transcript_	MSTRG.20915.[illegible]	gene_id	MSTRG.20915;
2	StringTie	exon	24764310	24764412	.	+	.	transcript_	MSTRG.20915.[illegible]	gene_id	MSTRG.20915;
2	StringTie	exon	24764975	24765150	.	+	.	transcript_	MSTRG.20915.[illegible]	gene_id	MSTRG.20915;
2	StringTie	exon	24766494	24767302	.	+	.	transcript_	MSTRG.20915.[illegible]	gene_id	MSTRG.20915;
2	StringTie	transcrip	26493695	26493960	.	+	.	transcript_	MSTRG.20954.[illegible]	gene_id	MSTRG.20954;
2	StringTie	exon	26493695	26493805	.	+	.	transcript_	MSTRG.20954.[illegible]	gene_id	MSTRG.20954;
2	StringTie	exon	26493852	26493960	.	+	.	transcript_	MSTRG.20954.[illegible]	gene_id	MSTRG.20954;
2	StringTie	transcrip	46669543	46770338	.	+	.	transcript_	MSTRG.21202.[illegible]	gene_id	MSTRG.21202;
2	StringTie	exon	46669543	46669760	.	+	.	transcript_	MSTRG.21202.[illegible]	gene_id	MSTRG.21202;
2	StringTie	exon	46673601	46673716	.	+	.	transcript_	MSTRG.21202.[illegible]	gene_id	MSTRG.21202;
2	StringTie	exon	46767468	46767571	.	+	.	transcript_	MSTRG.21202.[illegible]	gene_id	MSTRG.21202;
2	StringTie	exon	46769546	46770338	.	+	.	transcript_	MSTRG.21202.[illegible]	gene_id	MSTRG.21202;
2	StringTie	transcrip	46669543	46770342	.	+	.	transcript_	MSTRG.21202.[illegible]	gene_id	MSTRG.21202;
2	StringTie	exon	46669543	46669760	.	+	.	transcript_	MSTRG.21202.[illegible]	gene_id	MSTRG.21202;
2	StringTie	exon	46673601	46673716	.	+	.	transcript_	MSTRG.21202.[illegible]	gene_id	MSTRG.21202;
2	StringTie	exon	46714542	46714706	.	+	.	transcript_	MSTRG.21202.[illegible]	gene_id	MSTRG.21202;
2	StringTie	exon	46767468	46767571	.	+	.	transcript_	MSTRG.21202.[illegible]	gene_id	MSTRG.21202;
2	StringTie	exon	46769546	46770342	.	+	.	transcript_	MSTRG.21202.[illegible]	gene_id	MSTRG.21202;
2	StringTie	transcrip	46672823	46770342	.	+	.	transcript_	MSTRG.21202.[illegible]	gene_id	MSTRG.21202;
2	StringTie	exon	46672823	46673716	.	+	.	transcript_	MSTRG.21202.[illegible]	gene_id	MSTRG.21202;
2	StringTie	exon	46767471	46767571	.	+	.	transcript_	MSTRG.21202.[illegible]	gene_id	MSTRG.21202;
2	StringTie	exon	46769546	46770342	.	+	.	transcript_	MSTRG.21202.[illegible]	gene_id	MSTRG.21202;
2	StringTie	transcrip	46745449	46770338	.	+	.	transcript_	MSTRG.21202.[illegible]	gene_id	MSTRG.21202;
2	StringTie	exon	46745449	46746591	.	+	.	transcript_	MSTRG.21202.[illegible]	gene_id	MSTRG.21202;
2	StringTie	exon	46767468	46767571	.	+	.	transcript_	MSTRG.21202.[illegible]	gene_id	MSTRG.21202;
2	StringTie	exon	46769546	46770338	.	+	.	transcript_	MSTRG.21202.[illegible]	gene_id	MSTRG.21202;
2	StringTie	transcrip	49204850	49207540	.	+	.	transcript_	MSTRG.21233.[illegible]	gene_id	MSTRG.21233;
2	StringTie	exon	49204850	49204922	.	+	.	transcript_	MSTRG.21233.[illegible]	gene_id	MSTRG.21233;
2	StringTie	exon	49205804	49205961	.	+	.	transcript_	MSTRG.21233.[illegible]	gene_id	MSTRG.21233;
2	StringTie	exon	49207103	49207540	.	+	.	transcript_	MSTRG.21233.[illegible]	gene_id	MSTRG.21233;
2	StringTie	transcrip	49204880	49207198	.	+	.	transcript_	MSTRG.21233.[illegible]	gene_id	MSTRG.21233;
2	StringTie	exon	49204880	49204916	.	+	.	transcript_	MSTRG.21233.[illegible]	gene_id	MSTRG.21233;
2	StringTie	exon	49205804	49205961	.	+	.	transcript_	MSTRG.21233.[illegible]	gene_id	MSTRG.21233;

2	StringTie	exon	49207103	49207198	.	+	.	transcript_	MSTRG.21233.3	gene_id	MSTRG.21233;
2	StringTie	transcrip	51391906	51398450	.	+	.	transcript_	MSTRG.21346.1	gene_id	MSTRG.21346;
2	StringTie	exon	51391906	51391942	.	+	.	transcript_	MSTRG.21346.1	gene_id	MSTRG.21346;
2	StringTie	exon	51397179	51398450	.	+	.	transcript_	MSTRG.21346.1	gene_id	MSTRG.21346;
2	StringTie	transcrip	51420610	51424646	.	+	.	transcript_	MSTRG.21348.1	gene_id	MSTRG.21348;
2	StringTie	exon	51420610	51421836	.	+	.	transcript_	MSTRG.21348.1	gene_id	MSTRG.21348;
2	StringTie	exon	51424283	51424646	.	+	.	transcript_	MSTRG.21348.1	gene_id	MSTRG.21348;
2	StringTie	transcrip	59744486	59771021	.	+	.	transcript_	MSTRG.21406.1	gene_id	MSTRG.21406;
2	StringTie	exon	59744486	59744680	.	+	.	transcript_	MSTRG.21406.1	gene_id	MSTRG.21406;
2	StringTie	exon	59751526	59751594	.	+	.	transcript_	MSTRG.21406.1	gene_id	MSTRG.21406;
2	StringTie	exon	59754907	59755018	.	+	.	transcript_	MSTRG.21406.1	gene_id	MSTRG.21406;
2	StringTie	exon	59769965	59771021	.	+	.	transcript_	MSTRG.21406.1	gene_id	MSTRG.21406;
2	StringTie	transcrip	60314957	60316521	.	+	.	transcript_	MSTRG.21432.1	gene_id	MSTRG.21432;
2	StringTie	exon	60314957	60314981	.	+	.	transcript_	MSTRG.21432.1	gene_id	MSTRG.21432;
2	StringTie	exon	60315430	60316521	.	+	.	transcript_	MSTRG.21432.1	gene_id	MSTRG.21432;
2	StringTie	transcrip	61894131	62007405	.	+	.	transcript_	MSTRG.21488.1	gene_id	MSTRG.21488;
2	StringTie	exon	61894131	61894240	.	+	.	transcript_	MSTRG.21488.1	gene_id	MSTRG.21488;
2	StringTie	exon	62007301	62007405	.	+	.	transcript_	MSTRG.21488.1	gene_id	MSTRG.21488;
2	StringTie	transcrip	61938287	62007410	.	+	.	transcript_	MSTRG.21488.3	gene_id	MSTRG.21488;
2	StringTie	exon	61938287	61938969	.	+	.	transcript_	MSTRG.21488.3	gene_id	MSTRG.21488;
2	StringTie	exon	62007301	62007410	.	+	.	transcript_	MSTRG.21488.3	gene_id	MSTRG.21488;
2	StringTie	transcrip	61938287	62052378	.	+	.	transcript_	MSTRG.21488.2	gene_id	MSTRG.21488;
2	StringTie	exon	61938287	61938969	.	+	.	transcript_	MSTRG.21488.2	gene_id	MSTRG.21488;
2	StringTie	exon	62052269	62052378	.	+	.	transcript_	MSTRG.21488.2	gene_id	MSTRG.21488;
2	StringTie	transcrip	64734068	64758062	.	+	.	transcript_	MSTRG.21518.3	gene_id	MSTRG.21518;
2	StringTie	exon	64734068	64734325	.	+	.	transcript_	MSTRG.21518.3	gene_id	MSTRG.21518;
2	StringTie	exon	64735639	64736139	.	+	.	transcript_	MSTRG.21518.3	gene_id	MSTRG.21518;
2	StringTie	exon	64753575	64753661	.	+	.	transcript_	MSTRG.21518.3	gene_id	MSTRG.21518;
2	StringTie	exon	64756451	64758062	.	+	.	transcript_	MSTRG.21518.3	gene_id	MSTRG.21518;
2	StringTie	transcrip	69289557	69290636	.	+	.	transcript_	MSTRG.21671.1	gene_id	MSTRG.21671;
2	StringTie	exon	69289557	69289849	.	+	.	transcript_	MSTRG.21671.1	gene_id	MSTRG.21671;
2	StringTie	exon	69290397	69290636	.	+	.	transcript_	MSTRG.21671.1	gene_id	MSTRG.21671;
2	StringTie	transcrip	72704633	72741833	.	+	.	transcript_	MSTRG.21838.1	gene_id	MSTRG.21838;
2	StringTie	exon	72704633	72704733	.	+	.	transcript_	MSTRG.21838.1	gene_id	MSTRG.21838;
2	StringTie	exon	72722395	72722515	.	+	.	transcript_	MSTRG.21838.1	gene_id	MSTRG.21838;

2	StringTie	exon	72741641	72741833	.	+	.	transcript_	MSTRG.21838.1	gene_id	MSTRG.21838;
2	StringTie	transcrip	74780665	74810457	.	+	.	transcript_	MSTRG.21925.1	gene_id	MSTRG.21925;
2	StringTie	exon	74780665	74780816	.	+	.	transcript_	MSTRG.21925.1	gene_id	MSTRG.21925;
2	StringTie	exon	74810368	74810457	.	+	.	transcript_	MSTRG.21925.1	gene_id	MSTRG.21925;
2	StringTie	transcrip	74780681	74824221	.	+	.	transcript_	MSTRG.21925.2	gene_id	MSTRG.21925;
2	StringTie	exon	74780681	74780816	.	+	.	transcript_	MSTRG.21925.2	gene_id	MSTRG.21925;
2	StringTie	exon	74812588	74812674	.	+	.	transcript_	MSTRG.21925.2	gene_id	MSTRG.21925;
2	StringTie	exon	74819022	74819252	.	+	.	transcript_	MSTRG.21925.2	gene_id	MSTRG.21925;
2	StringTie	exon	74823460	74824221	.	+	.	transcript_	MSTRG.21925.2	gene_id	MSTRG.21925;
2	StringTie	transcrip	74787937	74824163	.	+	.	transcript_	MSTRG.21925.3	gene_id	MSTRG.21925;
2	StringTie	exon	74787937	74788103	.	+	.	transcript_	MSTRG.21925.3	gene_id	MSTRG.21925;
2	StringTie	exon	74788293	74788461	.	+	.	transcript_	MSTRG.21925.3	gene_id	MSTRG.21925;
2	StringTie	exon	74812588	74812674	.	+	.	transcript_	MSTRG.21925.3	gene_id	MSTRG.21925;
2	StringTie	exon	74819022	74819252	.	+	.	transcript_	MSTRG.21925.3	gene_id	MSTRG.21925;
2	StringTie	exon	74823460	74824163	.	+	.	transcript_	MSTRG.21925.3	gene_id	MSTRG.21925;
2	StringTie	transcrip	76615906	76616405	.	+	.	transcript_	MSTRG.21997.1	gene_id	MSTRG.21997;
2	StringTie	exon	76615906	76616016	.	+	.	transcript_	MSTRG.21997.1	gene_id	MSTRG.21997;
2	StringTie	exon	76616299	76616405	.	+	.	transcript_	MSTRG.21997.1	gene_id	MSTRG.21997;
2	StringTie	transcrip	77005427	77021250	.	+	.	transcript_	MSTRG.22018.1	gene_id	MSTRG.22018;
2	StringTie	exon	77005427	77005459	.	+	.	transcript_	MSTRG.22018.1	gene_id	MSTRG.22018;
2	StringTie	exon	77014673	77014797	.	+	.	transcript_	MSTRG.22018.1	gene_id	MSTRG.22018;
2	StringTie	exon	77015957	77016142	.	+	.	transcript_	MSTRG.22018.1	gene_id	MSTRG.22018;
2	StringTie	exon	77019276	77021250	.	+	.	transcript_	MSTRG.22018.1	gene_id	MSTRG.22018;
2	StringTie	transcrip	77012789	77021038	.	+	.	transcript_	MSTRG.22018.2	gene_id	MSTRG.22018;
2	StringTie	exon	77012789	77014797	.	+	.	transcript_	MSTRG.22018.2	gene_id	MSTRG.22018;
2	StringTie	exon	77015957	77016142	.	+	.	transcript_	MSTRG.22018.2	gene_id	MSTRG.22018;
2	StringTie	exon	77018957	77021038	.	+	.	transcript_	MSTRG.22018.2	gene_id	MSTRG.22018;
2	StringTie	transcrip	77018911	77021045	.	+	.	transcript_	MSTRG.22018.3	gene_id	MSTRG.22018;
2	StringTie	exon	77018911	77019046	.	+	.	transcript_	MSTRG.22018.3	gene_id	MSTRG.22018;
2	StringTie	exon	77019276	77021045	.	+	.	transcript_	MSTRG.22018.3	gene_id	MSTRG.22018;
2	StringTie	transcrip	80265840	80297933	.	+	.	transcript_	MSTRG.22154.3	gene_id	MSTRG.22154;
2	StringTie	exon	80265840	80266026	.	+	.	transcript_	MSTRG.22154.3	gene_id	MSTRG.22154;
2	StringTie	exon	80281888	80281992	.	+	.	transcript_	MSTRG.22154.3	gene_id	MSTRG.22154;
2	StringTie	exon	80286589	80286720	.	+	.	transcript_	MSTRG.22154.3	gene_id	MSTRG.22154;
2	StringTie	exon	80297290	80297933	.	+	.	transcript_	MSTRG.22154.3	gene_id	MSTRG.22154;

2	StringTie	transcrip	95793109	95794071	.	+	.	transcript_	MSTRG.22452.1	gene_id	MSTRG.22452;
2	StringTie	exon	95793109	95793314	.	+	.	transcript_	MSTRG.22452.1	gene_id	MSTRG.22452;
2	StringTie	exon	95793425	95793536	.	+	.	transcript_	MSTRG.22452.1	gene_id	MSTRG.22452;
2	StringTie	exon	95793980	95794071	.	+	.	transcript_	MSTRG.22452.1	gene_id	MSTRG.22452;
2	StringTie	transcrip	96203037	96210876	.	+	.	transcript_	MSTRG.22467.1	gene_id	MSTRG.22467;
2	StringTie	exon	96203037	96205845	.	+	.	transcript_	MSTRG.22467.1	gene_id	MSTRG.22467;
2	StringTie	exon	96206326	96210876	.	+	.	transcript_	MSTRG.22467.1	gene_id	MSTRG.22467;
2	StringTie	transcrip	100959689	100981432	.	+	.	transcript_	MSTRG.22508.1	gene_id	MSTRG.22508;
2	StringTie	exon	100959689	100959738	.	+	.	transcript_	MSTRG.22508.1	gene_id	MSTRG.22508;
2	StringTie	exon	100981219	100981432	.	+	.	transcript_	MSTRG.22508.1	gene_id	MSTRG.22508;
2	StringTie	transcrip	117056799	117090706	.	+	.	transcript_	MSTRG.22758.2	gene_id	MSTRG.22758;
2	StringTie	exon	117056799	117056884	.	+	.	transcript_	MSTRG.22758.2	gene_id	MSTRG.22758;
2	StringTie	exon	117074046	117074154	.	+	.	transcript_	MSTRG.22758.2	gene_id	MSTRG.22758;
2	StringTie	exon	117090419	117090706	.	+	.	transcript_	MSTRG.22758.2	gene_id	MSTRG.22758;
2	StringTie	transcrip	117056799	117090706	.	+	.	transcript_	MSTRG.22758.1	gene_id	MSTRG.22758;
2	StringTie	exon	117056799	117056884	.	+	.	transcript_	MSTRG.22758.1	gene_id	MSTRG.22758;
2	StringTie	exon	117074050	117074154	.	+	.	transcript_	MSTRG.22758.1	gene_id	MSTRG.22758;
2	StringTie	exon	117090419	117090706	.	+	.	transcript_	MSTRG.22758.1	gene_id	MSTRG.22758;
2	StringTie	transcrip	129913717	129952978	.	+	.	transcript_	MSTRG.22920.1	gene_id	MSTRG.22920;
2	StringTie	exon	129913717	129913741	.	+	.	transcript_	MSTRG.22920.1	gene_id	MSTRG.22920;
2	StringTie	exon	129952256	129952978	.	+	.	transcript_	MSTRG.22920.1	gene_id	MSTRG.22920;
2	StringTie	transcrip	130521216	130529527	.	+	.	transcript_	MSTRG.22904.1	gene_id	MSTRG.22904;
2	StringTie	exon	130521216	130521404	.	+	.	transcript_	MSTRG.22904.1	gene_id	MSTRG.22904;
2	StringTie	exon	130528929	130529527	.	+	.	transcript_	MSTRG.22904.1	gene_id	MSTRG.22904;
2	StringTie	transcrip	131845760	131848399	.	+	.	transcript_	MSTRG.22973.1	gene_id	MSTRG.22973;
2	StringTie	exon	131845760	131846206	.	+	.	transcript_	MSTRG.22973.1	gene_id	MSTRG.22973;
2	StringTie	exon	131847629	131848399	.	+	.	transcript_	MSTRG.22973.1	gene_id	MSTRG.22973;
2	StringTie	transcrip	132112523	132144162	.	+	.	transcript_	MSTRG.22978.1	gene_id	MSTRG.22978;
2	StringTie	exon	132112523	132112582	.	+	.	transcript_	MSTRG.22978.1	gene_id	MSTRG.22978;
2	StringTie	exon	132125958	132126089	.	+	.	transcript_	MSTRG.22978.1	gene_id	MSTRG.22978;
2	StringTie	exon	132144039	132144162	.	+	.	transcript_	MSTRG.22978.1	gene_id	MSTRG.22978;
2	StringTie	transcrip	140504635	140508576	.	+	.	transcript_	MSTRG.23135.1	gene_id	MSTRG.23135;
2	StringTie	exon	140504635	140505247	.	+	.	transcript_	MSTRG.23135.1	gene_id	MSTRG.23135;
2	StringTie	exon	140508476	140508576	.	+	.	transcript_	MSTRG.23135.1	gene_id	MSTRG.23135;
2	StringTie	transcrip	141380167	141395787	.	+	.	transcript_	MSTRG.23161.1	gene_id	MSTRG.23161;

2	StringTie	exon	141380167	141380453	.	+	.	transcript_	MSTRG.23161.:	gene_id	MSTRG.23161;
2	StringTie	exon	141380562	141380648	.	+	.	transcript_	MSTRG.23161.:	gene_id	MSTRG.23161;
2	StringTie	exon	141392285	141392428	.	+	.	transcript_	MSTRG.23161.:	gene_id	MSTRG.23161;
2	StringTie	exon	141393580	141393718	.	+	.	transcript_	MSTRG.23161.:	gene_id	MSTRG.23161;
2	StringTie	exon	141395566	141395787	.	+	.	transcript_	MSTRG.23161.:	gene_id	MSTRG.23161;
2	StringTie	transcrip	143225259	143225546	.	+	.	transcript_	MSTRG.23250.:	gene_id	MSTRG.23250;
2	StringTie	exon	143225259	143225399	.	+	.	transcript_	MSTRG.23250.:	gene_id	MSTRG.23250;
2	StringTie	exon	143225436	143225546	.	+	.	transcript_	MSTRG.23250.:	gene_id	MSTRG.23250;
2	StringTie	transcrip	149052598	149069142	.	+	.	transcript_	MSTRG.23363.:	gene_id	MSTRG.23363;
2	StringTie	exon	149052598	149052683	.	+	.	transcript_	MSTRG.23363.:	gene_id	MSTRG.23363;
2	StringTie	exon	149053151	149053233	.	+	.	transcript_	MSTRG.23363.:	gene_id	MSTRG.23363;
2	StringTie	exon	149061133	149061498	.	+	.	transcript_	MSTRG.23363.:	gene_id	MSTRG.23363;
2	StringTie	exon	149065822	149065928	.	+	.	transcript_	MSTRG.23363.:	gene_id	MSTRG.23363;
2	StringTie	exon	149068625	149069142	.	+	.	transcript_	MSTRG.23363.:	gene_id	MSTRG.23363;
2	StringTie	transcrip	151402387	151402761	.	+	.	transcript_	MSTRG.23445.:	gene_id	MSTRG.23445;
2	StringTie	exon	151402387	151402497	.	+	.	transcript_	MSTRG.23445.:	gene_id	MSTRG.23445;
2	StringTie	exon	151402651	151402761	.	+	.	transcript_	MSTRG.23445.:	gene_id	MSTRG.23445;
2	StringTie	transcrip	354841	359216	.	-	.	transcript_	MSTRG.20274.:	gene_id	MSTRG.20274;
2	StringTie	exon	354841	355059	.	-	.	transcript_	MSTRG.20274.:	gene_id	MSTRG.20274;
2	StringTie	exon	357502	359216	.	-	.	transcript_	MSTRG.20274.:	gene_id	MSTRG.20274;
2	StringTie	transcrip	470512	471750	.	-	.	transcript_	MSTRG.20279.:	gene_id	MSTRG.20279;
2	StringTie	exon	470512	470622	.	-	.	transcript_	MSTRG.20279.:	gene_id	MSTRG.20279;
2	StringTie	exon	471021	471750	.	-	.	transcript_	MSTRG.20279.:	gene_id	MSTRG.20279;
2	StringTie	transcrip	1661787	1666743	.	-	.	transcript_	MSTRG.20323.:	gene_id	MSTRG.20323;
2	StringTie	exon	1661787	1661858	.	-	.	transcript_	MSTRG.20323.:	gene_id	MSTRG.20323;
2	StringTie	exon	1666472	1666743	.	-	.	transcript_	MSTRG.20323.:	gene_id	MSTRG.20323;
2	StringTie	transcrip	2375366	2376489	.	-	.	transcript_	MSTRG.20355.:	gene_id	MSTRG.20355;
2	StringTie	exon	2375366	2375456	.	-	.	transcript_	MSTRG.20355.:	gene_id	MSTRG.20355;
2	StringTie	exon	2376295	2376489	.	-	.	transcript_	MSTRG.20355.:	gene_id	MSTRG.20355;
2	StringTie	transcrip	4672578	4681905	.	-	.	transcript_	MSTRG.20381.:	gene_id	MSTRG.20381;
2	StringTie	exon	4672578	4673941	.	-	.	transcript_	MSTRG.20381.:	gene_id	MSTRG.20381;
2	StringTie	exon	4681607	4681905	.	-	.	transcript_	MSTRG.20381.:	gene_id	MSTRG.20381;
2	StringTie	transcrip	4904328	4905175	.	-	.	transcript_	MSTRG.20405.:	gene_id	MSTRG.20405;
2	StringTie	exon	4904328	4904520	.	-	.	transcript_	MSTRG.20405.:	gene_id	MSTRG.20405;
2	StringTie	exon	4904800	4904966	.	-	.	transcript_	MSTRG.20405.:	gene_id	MSTRG.20405;

2	StringTie	exon	4905142	4905175	.	-	.	transcript_	MSTRG.20405.1	gene_id	MSTRG.20405;
2	StringTie	transcrip	4908723	4909015	.	-	.	transcript_	MSTRG.20406.1	gene_id	MSTRG.20406;
2	StringTie	exon	4908723	4908830	.	-	.	transcript_	MSTRG.20406.1	gene_id	MSTRG.20406;
2	StringTie	exon	4908921	4909015	.	-	.	transcript_	MSTRG.20406.1	gene_id	MSTRG.20406;
2	StringTie	transcrip	4909126	4917248	.	-	.	transcript_	MSTRG.20407.1	gene_id	MSTRG.20407;
2	StringTie	exon	4909126	4909211	.	-	.	transcript_	MSTRG.20407.1	gene_id	MSTRG.20407;
2	StringTie	exon	4909359	4909468	.	-	.	transcript_	MSTRG.20407.1	gene_id	MSTRG.20407;
2	StringTie	exon	4909721	4909806	.	-	.	transcript_	MSTRG.20407.1	gene_id	MSTRG.20407;
2	StringTie	exon	4915182	4915275	.	-	.	transcript_	MSTRG.20407.1	gene_id	MSTRG.20407;
2	StringTie	exon	4916811	4917248	.	-	.	transcript_	MSTRG.20407.1	gene_id	MSTRG.20407;
2	StringTie	transcrip	4930260	4935063	.	-	.	transcript_	MSTRG.20410.1	gene_id	MSTRG.20410;
2	StringTie	exon	4930260	4931530	.	-	.	transcript_	MSTRG.20410.1	gene_id	MSTRG.20410;
2	StringTie	exon	4932634	4932821	.	-	.	transcript_	MSTRG.20410.1	gene_id	MSTRG.20410;
2	StringTie	exon	4935009	4935063	.	-	.	transcript_	MSTRG.20410.1	gene_id	MSTRG.20410;
2	StringTie	transcrip	4930269	4935059	.	-	.	transcript_	MSTRG.20410.2	gene_id	MSTRG.20410;
2	StringTie	exon	4930269	4931838	.	-	.	transcript_	MSTRG.20410.2	gene_id	MSTRG.20410;
2	StringTie	exon	4932265	4932330	.	-	.	transcript_	MSTRG.20410.2	gene_id	MSTRG.20410;
2	StringTie	exon	4932634	4932821	.	-	.	transcript_	MSTRG.20410.2	gene_id	MSTRG.20410;
2	StringTie	exon	4935009	4935059	.	-	.	transcript_	MSTRG.20410.2	gene_id	MSTRG.20410;
2	StringTie	transcrip	5266053	5266511	.	-	.	transcript_	MSTRG.20440.1	gene_id	MSTRG.20440;
2	StringTie	exon	5266053	5266270	.	-	.	transcript_	MSTRG.20440.1	gene_id	MSTRG.20440;
2	StringTie	exon	5266455	5266511	.	-	.	transcript_	MSTRG.20440.1	gene_id	MSTRG.20440;
2	StringTie	transcrip	6750308	6757154	.	-	.	transcript_	MSTRG.20511.5	gene_id	MSTRG.20511;
2	StringTie	exon	6750308	6755700	.	-	.	transcript_	MSTRG.20511.5	gene_id	MSTRG.20511;
2	StringTie	exon	6755785	6757154	.	-	.	transcript_	MSTRG.20511.5	gene_id	MSTRG.20511;
2	StringTie	transcrip	6793275	6805625	.	-	.	transcript_	MSTRG.20518.2	gene_id	MSTRG.20518;
2	StringTie	exon	6793275	6793489	.	-	.	transcript_	MSTRG.20518.2	gene_id	MSTRG.20518;
2	StringTie	exon	6793920	6805625	.	-	.	transcript_	MSTRG.20518.2	gene_id	MSTRG.20518;
2	StringTie	transcrip	6796875	6800604	.	-	.	transcript_	MSTRG.20518.3	gene_id	MSTRG.20518;
2	StringTie	exon	6796875	6798599	.	-	.	transcript_	MSTRG.20518.3	gene_id	MSTRG.20518;
2	StringTie	exon	6799718	6800604	.	-	.	transcript_	MSTRG.20518.3	gene_id	MSTRG.20518;
2	StringTie	transcrip	6802100	6803511	.	-	.	transcript_	MSTRG.20518.4	gene_id	MSTRG.20518;
2	StringTie	exon	6802100	6802337	.	-	.	transcript_	MSTRG.20518.4	gene_id	MSTRG.20518;
2	StringTie	exon	6802441	6803511	.	-	.	transcript_	MSTRG.20518.4	gene_id	MSTRG.20518;
2	StringTie	transcrip	7385478	7391060	.	-	.	transcript_	MSTRG.20559.1	gene_id	MSTRG.20559;

2	StringTie	exon	7385478	7386331	.	-	.	transcript_	MSTRG.20559.[illegible]	gene_id	MSTRG.20559;
2	StringTie	exon	7390623	7391060	.	-	.	transcript_	MSTRG.20559.[illegible]	gene_id	MSTRG.20559;
2	StringTie	transcrip	7420845	7421114	.	-	.	transcript_	MSTRG.20562.[illegible]	gene_id	MSTRG.20562;
2	StringTie	exon	7420845	7420955	.	-	.	transcript_	MSTRG.20562.[illegible]	gene_id	MSTRG.20562;
2	StringTie	exon	7421004	7421114	.	-	.	transcript_	MSTRG.20562.[illegible]	gene_id	MSTRG.20562;
2	StringTie	transcrip	8449011	8450754	.	-	.	transcript_	MSTRG.20610.[illegible]	gene_id	MSTRG.20610;
2	StringTie	exon	8449011	8449120	.	-	.	transcript_	MSTRG.20610.[illegible]	gene_id	MSTRG.20610;
2	StringTie	exon	8450413	8450754	.	-	.	transcript_	MSTRG.20610.[illegible]	gene_id	MSTRG.20610;
2	StringTie	transcrip	9066937	9070793	.	-	.	transcript_	MSTRG.20625.[illegible]	gene_id	MSTRG.20625;
2	StringTie	exon	9066937	9067706	.	-	.	transcript_	MSTRG.20625.[illegible]	gene_id	MSTRG.20625;
2	StringTie	exon	9067976	9068042	.	-	.	transcript_	MSTRG.20625.[illegible]	gene_id	MSTRG.20625;
2	StringTie	exon	9069174	9070793	.	-	.	transcript_	MSTRG.20625.[illegible]	gene_id	MSTRG.20625;
2	StringTie	transcrip	9066937	9070947	.	-	.	transcript_	MSTRG.20625.[illegible]	gene_id	MSTRG.20625;
2	StringTie	exon	9066937	9067706	.	-	.	transcript_	MSTRG.20625.[illegible]	gene_id	MSTRG.20625;
2	StringTie	exon	9067976	9068129	.	-	.	transcript_	MSTRG.20625.[illegible]	gene_id	MSTRG.20625;
2	StringTie	exon	9069261	9070947	.	-	.	transcript_	MSTRG.20625.[illegible]	gene_id	MSTRG.20625;
2	StringTie	transcrip	9067891	9070947	.	-	.	transcript_	MSTRG.20625.[illegible]	gene_id	MSTRG.20625;
2	StringTie	exon	9067891	9068465	.	-	.	transcript_	MSTRG.20625.[illegible]	gene_id	MSTRG.20625;
2	StringTie	exon	9069597	9070947	.	-	.	transcript_	MSTRG.20625.[illegible]	gene_id	MSTRG.20625;
2	StringTie	transcrip	9067976	9070947	.	-	.	transcript_	MSTRG.20625.[illegible]	gene_id	MSTRG.20625;
2	StringTie	exon	9067976	9068700	.	-	.	transcript_	MSTRG.20625.[illegible]	gene_id	MSTRG.20625;
2	StringTie	exon	9069833	9070947	.	-	.	transcript_	MSTRG.20625.[illegible]	gene_id	MSTRG.20625;
2	StringTie	transcrip	9068566	9070654	.	-	.	transcript_	MSTRG.20625.[illegible]	gene_id	MSTRG.20625;
2	StringTie	exon	9068566	9069054	.	-	.	transcript_	MSTRG.20625.[illegible]	gene_id	MSTRG.20625;
2	StringTie	exon	9070187	9070654	.	-	.	transcript_	MSTRG.20625.[illegible]	gene_id	MSTRG.20625;
2	StringTie	transcrip	9200819	9202192	.	-	.	transcript_	MSTRG.20764.[illegible]	gene_id	MSTRG.20764;
2	StringTie	exon	9200819	9200929	.	-	.	transcript_	MSTRG.20764.[illegible]	gene_id	MSTRG.20764;
2	StringTie	exon	9202082	9202192	.	-	.	transcript_	MSTRG.20764.[illegible]	gene_id	MSTRG.20764;
2	StringTie	transcrip	9200819	9202896	.	-	.	transcript_	MSTRG.20764.[illegible]	gene_id	MSTRG.20764;
2	StringTie	exon	9200819	9200929	.	-	.	transcript_	MSTRG.20764.[illegible]	gene_id	MSTRG.20764;
2	StringTie	exon	9202688	9202896	.	-	.	transcript_	MSTRG.20764.[illegible]	gene_id	MSTRG.20764;
2	StringTie	transcrip	9202577	9204449	.	-	.	transcript_	MSTRG.20764.[illegible]	gene_id	MSTRG.20764;
2	StringTie	exon	9202577	9202687	.	-	.	transcript_	MSTRG.20764.[illegible]	gene_id	MSTRG.20764;
2	StringTie	exon	9203456	9203533	.	-	.	transcript_	MSTRG.20764.[illegible]	gene_id	MSTRG.20764;
2	StringTie	exon	9204341	9204449	.	-	.	transcript_	MSTRG.20764.[illegible]	gene_id	MSTRG.20764;

2	StringTie	transcrip	9202961	9204449	.	-	.	transcript_	MSTRG.20764.3	gene_id	MSTRG.20764;
2	StringTie	exon	9202961	9203071	.	-	.	transcript_	MSTRG.20764.3	gene_id	MSTRG.20764;
2	StringTie	exon	9203456	9203533	.	-	.	transcript_	MSTRG.20764.3	gene_id	MSTRG.20764;
2	StringTie	exon	9204341	9204449	.	-	.	transcript_	MSTRG.20764.3	gene_id	MSTRG.20764;
2	StringTie	transcrip	9207383	9210177	.	-	.	transcript_	MSTRG.20764.5	gene_id	MSTRG.20764;
2	StringTie	exon	9207383	9207430	.	-	.	transcript_	MSTRG.20764.5	gene_id	MSTRG.20764;
2	StringTie	exon	9209165	9209298	.	-	.	transcript_	MSTRG.20764.5	gene_id	MSTRG.20764;
2	StringTie	exon	9210067	9210177	.	-	.	transcript_	MSTRG.20764.5	gene_id	MSTRG.20764;
2	StringTie	transcrip	9208198	9211953	.	-	.	transcript_	MSTRG.20764.6	gene_id	MSTRG.20764;
2	StringTie	exon	9208198	9208308	.	-	.	transcript_	MSTRG.20764.6	gene_id	MSTRG.20764;
2	StringTie	exon	9211843	9211953	.	-	.	transcript_	MSTRG.20764.6	gene_id	MSTRG.20764;
2	StringTie	transcrip	9208807	9210177	.	-	.	transcript_	MSTRG.20764.7	gene_id	MSTRG.20764;
2	StringTie	exon	9208807	9208914	.	-	.	transcript_	MSTRG.20764.7	gene_id	MSTRG.20764;
2	StringTie	exon	9210067	9210177	.	-	.	transcript_	MSTRG.20764.7	gene_id	MSTRG.20764;
2	StringTie	transcrip	9535159	9535441	.	-	.	transcript_	MSTRG.20641.1	gene_id	MSTRG.20641;
2	StringTie	exon	9535159	9535268	.	-	.	transcript_	MSTRG.20641.1	gene_id	MSTRG.20641;
2	StringTie	exon	9535349	9535441	.	-	.	transcript_	MSTRG.20641.1	gene_id	MSTRG.20641;
2	StringTie	transcrip	18105753	18106747	.	-	.	transcript_	MSTRG.20857.1	gene_id	MSTRG.20857;
2	StringTie	exon	18105753	18106481	.	-	.	transcript_	MSTRG.20857.1	gene_id	MSTRG.20857;
2	StringTie	exon	18106585	18106747	.	-	.	transcript_	MSTRG.20857.1	gene_id	MSTRG.20857;
2	StringTie	transcrip	25246858	25249568	.	-	.	transcript_	MSTRG.20917.1	gene_id	MSTRG.20917;
2	StringTie	exon	25246858	25247573	.	-	.	transcript_	MSTRG.20917.1	gene_id	MSTRG.20917;
2	StringTie	exon	25248313	25248488	.	-	.	transcript_	MSTRG.20917.1	gene_id	MSTRG.20917;
2	StringTie	exon	25249473	25249568	.	-	.	transcript_	MSTRG.20917.1	gene_id	MSTRG.20917;
2	StringTie	transcrip	25253607	25271633	.	-	.	transcript_	MSTRG.20919.1	gene_id	MSTRG.20919;
2	StringTie	exon	25253607	25253705	.	-	.	transcript_	MSTRG.20919.1	gene_id	MSTRG.20919;
2	StringTie	exon	25262975	25263148	.	-	.	transcript_	MSTRG.20919.1	gene_id	MSTRG.20919;
2	StringTie	exon	25267669	25271633	.	-	.	transcript_	MSTRG.20919.1	gene_id	MSTRG.20919;
2	StringTie	transcrip	25266018	25271146	.	-	.	transcript_	MSTRG.20919.2	gene_id	MSTRG.20919;
2	StringTie	exon	25266018	25267754	.	-	.	transcript_	MSTRG.20919.2	gene_id	MSTRG.20919;
2	StringTie	exon	25268652	25268869	.	-	.	transcript_	MSTRG.20919.2	gene_id	MSTRG.20919;
2	StringTie	exon	25271067	25271146	.	-	.	transcript_	MSTRG.20919.2	gene_id	MSTRG.20919;
2	StringTie	transcrip	28489533	28497593	.	-	.	transcript_	MSTRG.20992.1	gene_id	MSTRG.20992;
2	StringTie	exon	28489533	28489584	.	-	.	transcript_	MSTRG.20992.1	gene_id	MSTRG.20992;
2	StringTie	exon	28493225	28493268	.	-	.	transcript_	MSTRG.20992.1	gene_id	MSTRG.20992;

2	StringTie	exon	28495334	28495531	.	-	.	transcript_	MSTRG.20992.1	gene_id	MSTRG.20992;
2	StringTie	exon	28497469	28497593	.	-	.	transcript_	MSTRG.20992.1	gene_id	MSTRG.20992;
2	StringTie	transcrip	40553069	40616217	.	-	.	transcript_	MSTRG.21098.1	gene_id	MSTRG.21098;
2	StringTie	exon	40553069	40553087	.	-	.	transcript_	MSTRG.21098.1	gene_id	MSTRG.21098;
2	StringTie	exon	40615385	40616217	.	-	.	transcript_	MSTRG.21098.1	gene_id	MSTRG.21098;
2	StringTie	transcrip	40569994	40613659	.	-	.	transcript_	MSTRG.21098.2	gene_id	MSTRG.21098;
2	StringTie	exon	40569994	40570024	.	-	.	transcript_	MSTRG.21098.2	gene_id	MSTRG.21098;
2	StringTie	exon	40612960	40613659	.	-	.	transcript_	MSTRG.21098.2	gene_id	MSTRG.21098;
2	StringTie	transcrip	46333571	46336648	.	-	.	transcript_	MSTRG.21191.1	gene_id	MSTRG.21191;
2	StringTie	exon	46333571	46334755	.	-	.	transcript_	MSTRG.21191.1	gene_id	MSTRG.21191;
2	StringTie	exon	46334811	46336648	.	-	.	transcript_	MSTRG.21191.1	gene_id	MSTRG.21191;
2	StringTie	transcrip	46769553	46770310	.	-	.	transcript_	MSTRG.21203.1	gene_id	MSTRG.21203;
2	StringTie	exon	46769553	46769789	.	-	.	transcript_	MSTRG.21203.1	gene_id	MSTRG.21203;
2	StringTie	exon	46769864	46770310	.	-	.	transcript_	MSTRG.21203.1	gene_id	MSTRG.21203;
2	StringTie	transcrip	48414848	48460559	.	-	.	transcript_	MSTRG.21208.1	gene_id	MSTRG.21208;
2	StringTie	exon	48414848	48414904	.	-	.	transcript_	MSTRG.21208.1	gene_id	MSTRG.21208;
2	StringTie	exon	48444554	48444674	.	-	.	transcript_	MSTRG.21208.1	gene_id	MSTRG.21208;
2	StringTie	exon	48460479	48460559	.	-	.	transcript_	MSTRG.21208.1	gene_id	MSTRG.21208;
2	StringTie	transcrip	51470458	51471304	.	-	.	transcript_	MSTRG.21349.1	gene_id	MSTRG.21349;
2	StringTie	exon	51470458	51470579	.	-	.	transcript_	MSTRG.21349.1	gene_id	MSTRG.21349;
2	StringTie	exon	51471187	51471304	.	-	.	transcript_	MSTRG.21349.1	gene_id	MSTRG.21349;
2	StringTie	transcrip	51650456	51657094	.	-	.	transcript_	MSTRG.21282.1	gene_id	MSTRG.21282;
2	StringTie	exon	51650456	51655001	.	-	.	transcript_	MSTRG.21282.1	gene_id	MSTRG.21282;
2	StringTie	exon	51656974	51657094	.	-	.	transcript_	MSTRG.21282.1	gene_id	MSTRG.21282;
2	StringTie	transcrip	51729601	51730585	.	-	.	transcript_	MSTRG.21287.1	gene_id	MSTRG.21287;
2	StringTie	exon	51729601	51729661	.	-	.	transcript_	MSTRG.21287.1	gene_id	MSTRG.21287;
2	StringTie	exon	51729765	51730585	.	-	.	transcript_	MSTRG.21287.1	gene_id	MSTRG.21287;
2	StringTie	transcrip	57848597	57870793	.	-	.	transcript_	MSTRG.21338.1	gene_id	MSTRG.21338;
2	StringTie	exon	57848597	57848613	.	-	.	transcript_	MSTRG.21338.1	gene_id	MSTRG.21338;
2	StringTie	exon	57869489	57870793	.	-	.	transcript_	MSTRG.21338.1	gene_id	MSTRG.21338;
2	StringTie	transcrip	62011611	62057651	.	-	.	transcript_	MSTRG.21489.1	gene_id	MSTRG.21489;
2	StringTie	exon	62011611	62011736	.	-	.	transcript_	MSTRG.21489.1	gene_id	MSTRG.21489;
2	StringTie	exon	62056704	62057651	.	-	.	transcript_	MSTRG.21489.1	gene_id	MSTRG.21489;
2	StringTie	transcrip	79202817	79204270	.	-	.	transcript_	MSTRG.22108.1	gene_id	MSTRG.22108;
2	StringTie	exon	79202817	79203062	.	-	.	transcript_	MSTRG.22108.1	gene_id	MSTRG.22108;

2	StringTie	exon	79203654	79203766	.	-	.	transcript_	MSTRG.22108.	gene_id	MSTRG.22108;
2	StringTie	exon	79204092	79204270	.	-	.	transcript_	MSTRG.22108.	gene_id	MSTRG.22108;
2	StringTie	transcrip	85486691	85513807	.	-	.	transcript_	MSTRG.22266.	gene_id	MSTRG.22266;
2	StringTie	exon	85486691	85492619	.	-	.	transcript_	MSTRG.22266.	gene_id	MSTRG.22266;
2	StringTie	exon	85499494	85499654	.	-	.	transcript_	MSTRG.22266.	gene_id	MSTRG.22266;
2	StringTie	exon	85513667	85513807	.	-	.	transcript_	MSTRG.22266.	gene_id	MSTRG.22266;
2	StringTie	transcrip	85490502	85513781	.	-	.	transcript_	MSTRG.22266.	gene_id	MSTRG.22266;
2	StringTie	exon	85490502	85492619	.	-	.	transcript_	MSTRG.22266.	gene_id	MSTRG.22266;
2	StringTie	exon	85499494	85499654	.	-	.	transcript_	MSTRG.22266.	gene_id	MSTRG.22266;
2	StringTie	exon	85513712	85513781	.	-	.	transcript_	MSTRG.22266.	gene_id	MSTRG.22266;
2	StringTie	transcrip	88449576	88450530	.	-	.	transcript_	MSTRG.22302.	gene_id	MSTRG.22302;
2	StringTie	exon	88449576	88449660	.	-	.	transcript_	MSTRG.22302.	gene_id	MSTRG.22302;
2	StringTie	exon	88449961	88450075	.	-	.	transcript_	MSTRG.22302.	gene_id	MSTRG.22302;
2	StringTie	exon	88450382	88450530	.	-	.	transcript_	MSTRG.22302.	gene_id	MSTRG.22302;
2	StringTie	transcrip	88449576	88450530	.	-	.	transcript_	MSTRG.22302.	gene_id	MSTRG.22302;
2	StringTie	exon	88449576	88449660	.	-	.	transcript_	MSTRG.22302.	gene_id	MSTRG.22302;
2	StringTie	exon	88449961	88450113	.	-	.	transcript_	MSTRG.22302.	gene_id	MSTRG.22302;
2	StringTie	exon	88450420	88450530	.	-	.	transcript_	MSTRG.22302.	gene_id	MSTRG.22302;
2	StringTie	transcrip	91271983	91273602	.	-	.	transcript_	MSTRG.22398.	gene_id	MSTRG.22398;
2	StringTie	exon	91271983	91272303	.	-	.	transcript_	MSTRG.22398.	gene_id	MSTRG.22398;
2	StringTie	exon	91273560	91273602	.	-	.	transcript_	MSTRG.22398.	gene_id	MSTRG.22398;
2	StringTie	transcrip	100293711	100395947	.	-	.	transcript_	MSTRG.22494.	gene_id	MSTRG.22494;
2	StringTie	exon	100293711	100294100	.	-	.	transcript_	MSTRG.22494.	gene_id	MSTRG.22494;
2	StringTie	exon	100380422	100380618	.	-	.	transcript_	MSTRG.22494.	gene_id	MSTRG.22494;
2	StringTie	exon	100395891	100395947	.	-	.	transcript_	MSTRG.22494.	gene_id	MSTRG.22494;
2	StringTie	transcrip	102296665	102335493	.	-	.	transcript_	MSTRG.22530.	gene_id	MSTRG.22530;
2	StringTie	exon	102296665	102297586	.	-	.	transcript_	MSTRG.22530.	gene_id	MSTRG.22530;
2	StringTie	exon	102330090	102330137	.	-	.	transcript_	MSTRG.22530.	gene_id	MSTRG.22530;
2	StringTie	exon	102330881	102330967	.	-	.	transcript_	MSTRG.22530.	gene_id	MSTRG.22530;
2	StringTie	exon	102335377	102335493	.	-	.	transcript_	MSTRG.22530.	gene_id	MSTRG.22530;
2	StringTie	transcrip	102297383	102335457	.	-	.	transcript_	MSTRG.22530.	gene_id	MSTRG.22530;
2	StringTie	exon	102297383	102297586	.	-	.	transcript_	MSTRG.22530.	gene_id	MSTRG.22530;
2	StringTie	exon	102301121	102301195	.	-	.	transcript_	MSTRG.22530.	gene_id	MSTRG.22530;
2	StringTie	exon	102330881	102330967	.	-	.	transcript_	MSTRG.22530.	gene_id	MSTRG.22530;
2	StringTie	exon	102335377	102335457	.	-	.	transcript_	MSTRG.22530.	gene_id	MSTRG.22530;

2	StringTie	transcrip	102297386	102335456	.	-	.	transcript_	MSTRG.22530.3	gene_id	MSTRG.22530;
2	StringTie	exon	102297386	102297586	.	-	.	transcript_	MSTRG.22530.3	gene_id	MSTRG.22530;
2	StringTie	exon	102330881	102330967	.	-	.	transcript_	MSTRG.22530.3	gene_id	MSTRG.22530;
2	StringTie	exon	102335377	102335456	.	-	.	transcript_	MSTRG.22530.3	gene_id	MSTRG.22530;
2	StringTie	transcrip	105044217	105045622	.	-	.	transcript_	MSTRG.22608.1	gene_id	MSTRG.22608;
2	StringTie	exon	105044217	105044313	.	-	.	transcript_	MSTRG.22608.1	gene_id	MSTRG.22608;
2	StringTie	exon	105045002	105045622	.	-	.	transcript_	MSTRG.22608.1	gene_id	MSTRG.22608;
2	StringTie	transcrip	117504170	117505215	.	-	.	transcript_	MSTRG.22764.1	gene_id	MSTRG.22764;
2	StringTie	exon	117504170	117504280	.	-	.	transcript_	MSTRG.22764.1	gene_id	MSTRG.22764;
2	StringTie	exon	117504638	117504712	.	-	.	transcript_	MSTRG.22764.1	gene_id	MSTRG.22764;
2	StringTie	exon	117505105	117505215	.	-	.	transcript_	MSTRG.22764.1	gene_id	MSTRG.22764;
2	StringTie	transcrip	123306626	123313386	.	-	.	transcript_	MSTRG.22847.1	gene_id	MSTRG.22847;
2	StringTie	exon	123306626	123307240	.	-	.	transcript_	MSTRG.22847.1	gene_id	MSTRG.22847;
2	StringTie	exon	123312635	123313386	.	-	.	transcript_	MSTRG.22847.1	gene_id	MSTRG.22847;
2	StringTie	transcrip	130440426	130522522	.	-	.	transcript_	MSTRG.22901.1	gene_id	MSTRG.22901;
2	StringTie	exon	130440426	130440807	.	-	.	transcript_	MSTRG.22901.1	gene_id	MSTRG.22901;
2	StringTie	exon	130491950	130492050	.	-	.	transcript_	MSTRG.22901.1	gene_id	MSTRG.22901;
2	StringTie	exon	130522134	130522231	.	-	.	transcript_	MSTRG.22901.1	gene_id	MSTRG.22901;
2	StringTie	exon	130522433	130522522	.	-	.	transcript_	MSTRG.22901.1	gene_id	MSTRG.22901;
2	StringTie	transcrip	135118782	135119887	.	-	.	transcript_	MSTRG.23023.1	gene_id	MSTRG.23023;
2	StringTie	exon	135118782	135119103	.	-	.	transcript_	MSTRG.23023.1	gene_id	MSTRG.23023;
2	StringTie	exon	135119793	135119887	.	-	.	transcript_	MSTRG.23023.1	gene_id	MSTRG.23023;
2	StringTie	transcrip	136713256	136728868	.	-	.	transcript_	MSTRG.23064.1	gene_id	MSTRG.23064;
2	StringTie	exon	136713256	136713950	.	-	.	transcript_	MSTRG.23064.1	gene_id	MSTRG.23064;
2	StringTie	exon	136714498	136714597	.	-	.	transcript_	MSTRG.23064.1	gene_id	MSTRG.23064;
2	StringTie	exon	136716175	136716422	.	-	.	transcript_	MSTRG.23064.1	gene_id	MSTRG.23064;
2	StringTie	exon	136728602	136728868	.	-	.	transcript_	MSTRG.23064.1	gene_id	MSTRG.23064;
2	StringTie	transcrip	136931527	136957532	.	-	.	transcript_	MSTRG.23096.1	gene_id	MSTRG.23096;
2	StringTie	exon	136931527	136931875	.	-	.	transcript_	MSTRG.23096.1	gene_id	MSTRG.23096;
2	StringTie	exon	136946346	136946575	.	-	.	transcript_	MSTRG.23096.1	gene_id	MSTRG.23096;
2	StringTie	exon	136957329	136957532	.	-	.	transcript_	MSTRG.23096.1	gene_id	MSTRG.23096;
2	StringTie	transcrip	137383777	137385365	.	-	.	transcript_	MSTRG.23082.1	gene_id	MSTRG.23082;
2	StringTie	exon	137383777	137384143	.	-	.	transcript_	MSTRG.23082.1	gene_id	MSTRG.23082;
2	StringTie	exon	137384946	137385365	.	-	.	transcript_	MSTRG.23082.1	gene_id	MSTRG.23082;
2	StringTie	transcrip	137557556	137560808	.	-	.	transcript_	MSTRG.23085.1	gene_id	MSTRG.23085;

2	StringTie	exon	137557556	137557877	.	-	.	transcript_	MSTRG.23085.1	gene_id	MSTRG.23085;
2	StringTie	exon	137557911	137560808	.	-	.	transcript_	MSTRG.23085.1	gene_id	MSTRG.23085;
2	StringTie	transcrip	138144615	138146329	.	-	.	transcript_	MSTRG.23086.1	gene_id	MSTRG.23086;
2	StringTie	exon	138144615	138144807	.	-	.	transcript_	MSTRG.23086.1	gene_id	MSTRG.23086;
2	StringTie	exon	138145491	138146329	.	-	.	transcript_	MSTRG.23086.1	gene_id	MSTRG.23086;
2	StringTie	transcrip	141481009	141481792	.	-	.	transcript_	MSTRG.23166.1	gene_id	MSTRG.23166;
2	StringTie	exon	141481009	141481264	.	-	.	transcript_	MSTRG.23166.1	gene_id	MSTRG.23166;
2	StringTie	exon	141481401	141481792	.	-	.	transcript_	MSTRG.23166.1	gene_id	MSTRG.23166;
2	StringTie	transcrip	141873490	141900151	.	-	.	transcript_	MSTRG.23176.1	gene_id	MSTRG.23176;
2	StringTie	exon	141873490	141873522	.	-	.	transcript_	MSTRG.23176.1	gene_id	MSTRG.23176;
2	StringTie	exon	141897823	141900151	.	-	.	transcript_	MSTRG.23176.1	gene_id	MSTRG.23176;
2	StringTie	transcrip	142858908	142874601	.	-	.	transcript_	MSTRG.23232.1	gene_id	MSTRG.23232;
2	StringTie	exon	142858908	142859159	.	-	.	transcript_	MSTRG.23232.1	gene_id	MSTRG.23232;
2	StringTie	exon	142873129	142874601	.	-	.	transcript_	MSTRG.23232.1	gene_id	MSTRG.23232;
2	StringTie	transcrip	142864170	142874691	.	-	.	transcript_	MSTRG.23232.2	gene_id	MSTRG.23232;
2	StringTie	exon	142864170	142864349	.	-	.	transcript_	MSTRG.23232.2	gene_id	MSTRG.23232;
2	StringTie	exon	142873129	142874691	.	-	.	transcript_	MSTRG.23232.2	gene_id	MSTRG.23232;
2	StringTie	transcrip	144084295	144089512	.	-	.	transcript_	MSTRG.23279.1	gene_id	MSTRG.23279;
2	StringTie	exon	144084295	144084367	.	-	.	transcript_	MSTRG.23279.1	gene_id	MSTRG.23279;
2	StringTie	exon	144089312	144089512	.	-	.	transcript_	MSTRG.23279.1	gene_id	MSTRG.23279;
2	StringTie	transcrip	149552448	149553203	.	-	.	transcript_	MSTRG.23373.1	gene_id	MSTRG.23373;
2	StringTie	exon	149552448	149552505	.	-	.	transcript_	MSTRG.23373.1	gene_id	MSTRG.23373;
2	StringTie	exon	149553005	149553203	.	-	.	transcript_	MSTRG.23373.1	gene_id	MSTRG.23373;
2	StringTie	transcrip	150392988	150499256	.	-	.	transcript_	MSTRG.23412.1	gene_id	MSTRG.23412;
2	StringTie	exon	150392988	150393296	.	-	.	transcript_	MSTRG.23412.1	gene_id	MSTRG.23412;
2	StringTie	exon	150409569	150409701	.	-	.	transcript_	MSTRG.23412.1	gene_id	MSTRG.23412;
2	StringTie	exon	150499137	150499256	.	-	.	transcript_	MSTRG.23412.1	gene_id	MSTRG.23412;
2	StringTie	transcrip	150515242	150525096	.	-	.	transcript_	MSTRG.23416.1	gene_id	MSTRG.23416;
2	StringTie	exon	150515242	150521028	.	-	.	transcript_	MSTRG.23416.1	gene_id	MSTRG.23416;
2	StringTie	exon	150524899	150525096	.	-	.	transcript_	MSTRG.23416.1	gene_id	MSTRG.23416;
2	StringTie	transcrip	150517068	150525569	.	-	.	transcript_	MSTRG.23416.2	gene_id	MSTRG.23416;
2	StringTie	exon	150517068	150517165	.	-	.	transcript_	MSTRG.23416.2	gene_id	MSTRG.23416;
2	StringTie	exon	150519165	150519286	.	-	.	transcript_	MSTRG.23416.2	gene_id	MSTRG.23416;
2	StringTie	exon	150524899	150525569	.	-	.	transcript_	MSTRG.23416.2	gene_id	MSTRG.23416;
2	StringTie	transcrip	150517752	150526916	.	-	.	transcript_	MSTRG.23416.3	gene_id	MSTRG.23416;

2	StringTie	exon	150517752	150517820	.	-	.	transcript_	MSTRG.23416.3	gene_id	MSTRG.23416;
2	StringTie	exon	150519165	150519286	.	-	.	transcript_	MSTRG.23416.3	gene_id	MSTRG.23416;
2	StringTie	exon	150526590	150526916	.	-	.	transcript_	MSTRG.23416.3	gene_id	MSTRG.23416;
2	StringTie	transcrip	150528214	150535922	.	-	.	transcript_	MSTRG.23416.5	gene_id	MSTRG.23416;
2	StringTie	exon	150528214	150529079	.	-	.	transcript_	MSTRG.23416.5	gene_id	MSTRG.23416;
2	StringTie	exon	150531160	150531253	.	-	.	transcript_	MSTRG.23416.5	gene_id	MSTRG.23416;
2	StringTie	exon	150535646	150535922	.	-	.	transcript_	MSTRG.23416.5	gene_id	MSTRG.23416;
2	StringTie	transcrip	150528289	150536048	.	-	.	transcript_	MSTRG.23416.6	gene_id	MSTRG.23416;
2	StringTie	exon	150528289	150529079	.	-	.	transcript_	MSTRG.23416.6	gene_id	MSTRG.23416;
2	StringTie	exon	150531160	150531253	.	-	.	transcript_	MSTRG.23416.6	gene_id	MSTRG.23416;
2	StringTie	exon	150532037	150532202	.	-	.	transcript_	MSTRG.23416.6	gene_id	MSTRG.23416;
2	StringTie	exon	150535646	150536048	.	-	.	transcript_	MSTRG.23416.6	gene_id	MSTRG.23416;
2	StringTie	transcrip	150531222	150531621	.	-	.	transcript_	MSTRG.23416.7	gene_id	MSTRG.23416;
2	StringTie	exon	150531222	150531253	.	-	.	transcript_	MSTRG.23416.7	gene_id	MSTRG.23416;
2	StringTie	exon	150531352	150531621	.	-	.	transcript_	MSTRG.23416.7	gene_id	MSTRG.23416;
2	StringTie	transcrip	150557834	150581699	.	-	.	transcript_	MSTRG.23416.1	gene_id	MSTRG.23416;
2	StringTie	exon	150557834	150559186	.	-	.	transcript_	MSTRG.23416.1	gene_id	MSTRG.23416;
2	StringTie	exon	150580217	150581699	.	-	.	transcript_	MSTRG.23416.1	gene_id	MSTRG.23416;
2	StringTie	transcrip	150562346	150581697	.	-	.	transcript_	MSTRG.23416.1	gene_id	MSTRG.23416;
2	StringTie	exon	150562346	150562854	.	-	.	transcript_	MSTRG.23416.1	gene_id	MSTRG.23416;
2	StringTie	exon	150580217	150581697	.	-	.	transcript_	MSTRG.23416.1	gene_id	MSTRG.23416;
2	StringTie	transcrip	150530129	150530726	.	-	.	transcript_	MSTRG.23418.1	gene_id	MSTRG.23418;
2	StringTie	exon	150530129	150530625	.	-	.	transcript_	MSTRG.23418.1	gene_id	MSTRG.23418;
2	StringTie	exon	150530678	150530726	.	-	.	transcript_	MSTRG.23418.1	gene_id	MSTRG.23418;
2	StringTie	transcrip	150816816	150825223	.	-	.	transcript_	MSTRG.23404.1	gene_id	MSTRG.23404;
2	StringTie	exon	150816816	150821163	.	-	.	transcript_	MSTRG.23404.1	gene_id	MSTRG.23404;
2	StringTie	exon	150825169	150825223	.	-	.	transcript_	MSTRG.23404.1	gene_id	MSTRG.23404;
2	StringTie	transcrip	151742556	151745517	.	-	.	transcript_	MSTRG.23441.1	gene_id	MSTRG.23441;
2	StringTie	exon	151742556	151743518	.	-	.	transcript_	MSTRG.23441.1	gene_id	MSTRG.23441;
2	StringTie	exon	151744975	151745517	.	-	.	transcript_	MSTRG.23441.1	gene_id	MSTRG.23441;
3	StringTie	transcrip	1721093	1788850	.	+	.	transcript_	MSTRG.23503.2	gene_id	MSTRG.23503;
3	StringTie	exon	1721093	1721155	.	+	.	transcript_	MSTRG.23503.2	gene_id	MSTRG.23503;
3	StringTie	exon	1761839	1761939	.	+	.	transcript_	MSTRG.23503.2	gene_id	MSTRG.23503;
3	StringTie	exon	1772361	1772422	.	+	.	transcript_	MSTRG.23503.2	gene_id	MSTRG.23503;
3	StringTie	exon	1788699	1788850	.	+	.	transcript_	MSTRG.23503.2	gene_id	MSTRG.23503;

3	StringTie	transcrip	4120688	4124151	.	+	.	transcript_	MSTRG.23543.:	gene_id	MSTRG.23543;
3	StringTie	exon	4120688	4121469	.	+	.	transcript_	MSTRG.23543.:	gene_id	MSTRG.23543;
3	StringTie	exon	4122439	4122476	.	+	.	transcript_	MSTRG.23543.:	gene_id	MSTRG.23543;
3	StringTie	exon	4123678	4124151	.	+	.	transcript_	MSTRG.23543.:	gene_id	MSTRG.23543;
3	StringTie	transcrip	5498351	5504851	.	+	.	transcript_	MSTRG.23595.:	gene_id	MSTRG.23595;
3	StringTie	exon	5498351	5500913	.	+	.	transcript_	MSTRG.23595.:	gene_id	MSTRG.23595;
3	StringTie	exon	5504022	5504851	.	+	.	transcript_	MSTRG.23595.:	gene_id	MSTRG.23595;
3	StringTie	transcrip	6422379	6425678	.	+	.	transcript_	MSTRG.23611.:	gene_id	MSTRG.23611;
3	StringTie	exon	6422379	6422987	.	+	.	transcript_	MSTRG.23611.:	gene_id	MSTRG.23611;
3	StringTie	exon	6425649	6425678	.	+	.	transcript_	MSTRG.23611.:	gene_id	MSTRG.23611;
3	StringTie	transcrip	7646107	7653415	.	+	.	transcript_	MSTRG.23649.:	gene_id	MSTRG.23649;
3	StringTie	exon	7646107	7646258	.	+	.	transcript_	MSTRG.23649.:	gene_id	MSTRG.23649;
3	StringTie	exon	7651000	7653415	.	+	.	transcript_	MSTRG.23649.:	gene_id	MSTRG.23649;
3	StringTie	transcrip	8942030	8946849	.	+	.	transcript_	MSTRG.23725.:	gene_id	MSTRG.23725;
3	StringTie	exon	8942030	8942134	.	+	.	transcript_	MSTRG.23725.:	gene_id	MSTRG.23725;
3	StringTie	exon	8945087	8945300	.	+	.	transcript_	MSTRG.23725.:	gene_id	MSTRG.23725;
3	StringTie	exon	8946748	8946849	.	+	.	transcript_	MSTRG.23725.:	gene_id	MSTRG.23725;
3	StringTie	transcrip	10536603	10635884	.	+	.	transcript_	MSTRG.23768.:	gene_id	MSTRG.23768;
3	StringTie	exon	10536603	10536637	.	+	.	transcript_	MSTRG.23768.:	gene_id	MSTRG.23768;
3	StringTie	exon	10635617	10635884	.	+	.	transcript_	MSTRG.23768.:	gene_id	MSTRG.23768;
3	StringTie	transcrip	12040281	12060327	.	+	.	transcript_	MSTRG.23836.:	gene_id	MSTRG.23836;
3	StringTie	exon	12040281	12040339	.	+	.	transcript_	MSTRG.23836.:	gene_id	MSTRG.23836;
3	StringTie	exon	12041100	12041172	.	+	.	transcript_	MSTRG.23836.:	gene_id	MSTRG.23836;
3	StringTie	exon	12042144	12042224	.	+	.	transcript_	MSTRG.23836.:	gene_id	MSTRG.23836;
3	StringTie	exon	12060253	12060327	.	+	.	transcript_	MSTRG.23836.:	gene_id	MSTRG.23836;
3	StringTie	transcrip	16376831	16388410	.	+	.	transcript_	MSTRG.23872.:	gene_id	MSTRG.23872;
3	StringTie	exon	16376831	16376980	.	+	.	transcript_	MSTRG.23872.:	gene_id	MSTRG.23872;
3	StringTie	exon	16382682	16382827	.	+	.	transcript_	MSTRG.23872.:	gene_id	MSTRG.23872;
3	StringTie	exon	16388145	16388410	.	+	.	transcript_	MSTRG.23872.:	gene_id	MSTRG.23872;
3	StringTie	transcrip	17431158	17436921	.	+	.	transcript_	MSTRG.23938.:	gene_id	MSTRG.23938;
3	StringTie	exon	17431158	17431219	.	+	.	transcript_	MSTRG.23938.:	gene_id	MSTRG.23938;
3	StringTie	exon	17432977	17433106	.	+	.	transcript_	MSTRG.23938.:	gene_id	MSTRG.23938;
3	StringTie	exon	17436858	17436921	.	+	.	transcript_	MSTRG.23938.:	gene_id	MSTRG.23938;
3	StringTie	transcrip	17909773	17911691	.	+	.	transcript_	MSTRG.23986.:	gene_id	MSTRG.23986;
3	StringTie	exon	17909773	17909842	.	+	.	transcript_	MSTRG.23986.:	gene_id	MSTRG.23986;

3	StringTie	exon	17910742	17911691	.	+	.	transcript_	MSTRG.23986.1	gene_id	MSTRG.23986;
3	StringTie	transcrip	23720311	23760058	.	+	.	transcript_	MSTRG.24094.1	gene_id	MSTRG.24094;
3	StringTie	exon	23720311	23720418	.	+	.	transcript_	MSTRG.24094.1	gene_id	MSTRG.24094;
3	StringTie	exon	23721008	23721095	.	+	.	transcript_	MSTRG.24094.1	gene_id	MSTRG.24094;
3	StringTie	exon	23754421	23754532	.	+	.	transcript_	MSTRG.24094.1	gene_id	MSTRG.24094;
3	StringTie	exon	23756048	23760058	.	+	.	transcript_	MSTRG.24094.1	gene_id	MSTRG.24094;
3	StringTie	transcrip	23720320	23792578	.	+	.	transcript_	MSTRG.24094.2	gene_id	MSTRG.24094;
3	StringTie	exon	23720320	23720418	.	+	.	transcript_	MSTRG.24094.2	gene_id	MSTRG.24094;
3	StringTie	exon	23721008	23721095	.	+	.	transcript_	MSTRG.24094.2	gene_id	MSTRG.24094;
3	StringTie	exon	23754421	23754532	.	+	.	transcript_	MSTRG.24094.2	gene_id	MSTRG.24094;
3	StringTie	exon	23775095	23775223	.	+	.	transcript_	MSTRG.24094.2	gene_id	MSTRG.24094;
3	StringTie	exon	23792546	23792578	.	+	.	transcript_	MSTRG.24094.2	gene_id	MSTRG.24094;
3	StringTie	transcrip	23720341	23777802	.	+	.	transcript_	MSTRG.24094.3	gene_id	MSTRG.24094;
3	StringTie	exon	23720341	23720418	.	+	.	transcript_	MSTRG.24094.3	gene_id	MSTRG.24094;
3	StringTie	exon	23721008	23721095	.	+	.	transcript_	MSTRG.24094.3	gene_id	MSTRG.24094;
3	StringTie	exon	23754421	23754532	.	+	.	transcript_	MSTRG.24094.3	gene_id	MSTRG.24094;
3	StringTie	exon	23775095	23775223	.	+	.	transcript_	MSTRG.24094.3	gene_id	MSTRG.24094;
3	StringTie	exon	23777558	23777802	.	+	.	transcript_	MSTRG.24094.3	gene_id	MSTRG.24094;
3	StringTie	transcrip	23720342	23731341	.	+	.	transcript_	MSTRG.24094.4	gene_id	MSTRG.24094;
3	StringTie	exon	23720342	23720418	.	+	.	transcript_	MSTRG.24094.4	gene_id	MSTRG.24094;
3	StringTie	exon	23721008	23721095	.	+	.	transcript_	MSTRG.24094.4	gene_id	MSTRG.24094;
3	StringTie	exon	23731014	23731341	.	+	.	transcript_	MSTRG.24094.4	gene_id	MSTRG.24094;
3	StringTie	transcrip	23754421	23796665	.	+	.	transcript_	MSTRG.24094.5	gene_id	MSTRG.24094;
3	StringTie	exon	23754421	23754532	.	+	.	transcript_	MSTRG.24094.5	gene_id	MSTRG.24094;
3	StringTie	exon	23775095	23775223	.	+	.	transcript_	MSTRG.24094.5	gene_id	MSTRG.24094;
3	StringTie	exon	23796128	23796665	.	+	.	transcript_	MSTRG.24094.5	gene_id	MSTRG.24094;
3	StringTie	transcrip	27379195	27385447	.	+	.	transcript_	MSTRG.24177.1	gene_id	MSTRG.24177;
3	StringTie	exon	27379195	27379310	.	+	.	transcript_	MSTRG.24177.1	gene_id	MSTRG.24177;
3	StringTie	exon	27382972	27385447	.	+	.	transcript_	MSTRG.24177.1	gene_id	MSTRG.24177;
3	StringTie	transcrip	28567903	28572792	.	+	.	transcript_	MSTRG.24188.1	gene_id	MSTRG.24188;
3	StringTie	exon	28567903	28568073	.	+	.	transcript_	MSTRG.24188.1	gene_id	MSTRG.24188;
3	StringTie	exon	28572526	28572792	.	+	.	transcript_	MSTRG.24188.1	gene_id	MSTRG.24188;
3	StringTie	transcrip	28578810	28588752	.	+	.	transcript_	MSTRG.24190.2	gene_id	MSTRG.24190;
3	StringTie	exon	28578810	28578904	.	+	.	transcript_	MSTRG.24190.2	gene_id	MSTRG.24190;
3	StringTie	exon	28587997	28588116	.	+	.	transcript_	MSTRG.24190.2	gene_id	MSTRG.24190;

3	StringTie	exon	28588643	28588752	.	+	.	transcript_	MSTRG.24190.2	gene_id	MSTRG.24190;
3	StringTie	transcrip	31133728	31137638	.	+	.	transcript_	MSTRG.24214.1	gene_id	MSTRG.24214;
3	StringTie	exon	31133728	31133834	.	+	.	transcript_	MSTRG.24214.1	gene_id	MSTRG.24214;
3	StringTie	exon	31137531	31137638	.	+	.	transcript_	MSTRG.24214.1	gene_id	MSTRG.24214;
3	StringTie	transcrip	31133747	31141503	.	+	.	transcript_	MSTRG.24214.2	gene_id	MSTRG.24214;
3	StringTie	exon	31133747	31133834	.	+	.	transcript_	MSTRG.24214.2	gene_id	MSTRG.24214;
3	StringTie	exon	31140914	31141503	.	+	.	transcript_	MSTRG.24214.2	gene_id	MSTRG.24214;
3	StringTie	transcrip	32530151	32530905	.	+	.	transcript_	MSTRG.24234.1	gene_id	MSTRG.24234;
3	StringTie	exon	32530151	32530360	.	+	.	transcript_	MSTRG.24234.1	gene_id	MSTRG.24234;
3	StringTie	exon	32530871	32530905	.	+	.	transcript_	MSTRG.24234.1	gene_id	MSTRG.24234;
3	StringTie	transcrip	37633012	37635786	.	+	.	transcript_	MSTRG.24264.1	gene_id	MSTRG.24264;
3	StringTie	exon	37633012	37633077	.	+	.	transcript_	MSTRG.24264.1	gene_id	MSTRG.24264;
3	StringTie	exon	37634671	37634885	.	+	.	transcript_	MSTRG.24264.1	gene_id	MSTRG.24264;
3	StringTie	exon	37635214	37635786	.	+	.	transcript_	MSTRG.24264.1	gene_id	MSTRG.24264;
3	StringTie	transcrip	38399026	38399951	.	+	.	transcript_	MSTRG.24308.1	gene_id	MSTRG.24308;
3	StringTie	exon	38399026	38399580	.	+	.	transcript_	MSTRG.24308.1	gene_id	MSTRG.24308;
3	StringTie	exon	38399907	38399951	.	+	.	transcript_	MSTRG.24308.1	gene_id	MSTRG.24308;
3	StringTie	transcrip	41265797	41266613	.	+	.	transcript_	MSTRG.24495.1	gene_id	MSTRG.24495;
3	StringTie	exon	41265797	41266215	.	+	.	transcript_	MSTRG.24495.1	gene_id	MSTRG.24495;
3	StringTie	exon	41266268	41266613	.	+	.	transcript_	MSTRG.24495.1	gene_id	MSTRG.24495;
3	StringTie	transcrip	41482346	41486942	.	+	.	transcript_	MSTRG.24516.1	gene_id	MSTRG.24516;
3	StringTie	exon	41482346	41482485	.	+	.	transcript_	MSTRG.24516.1	gene_id	MSTRG.24516;
3	StringTie	exon	41486832	41486942	.	+	.	transcript_	MSTRG.24516.1	gene_id	MSTRG.24516;
3	StringTie	transcrip	43915868	43950785	.	+	.	transcript_	MSTRG.24607.2	gene_id	MSTRG.24607;
3	StringTie	exon	43915868	43916244	.	+	.	transcript_	MSTRG.24607.2	gene_id	MSTRG.24607;
3	StringTie	exon	43950718	43950785	.	+	.	transcript_	MSTRG.24607.2	gene_id	MSTRG.24607;
3	StringTie	transcrip	44022714	44024477	.	+	.	transcript_	MSTRG.24612.1	gene_id	MSTRG.24612;
3	StringTie	exon	44022714	44023005	.	+	.	transcript_	MSTRG.24612.1	gene_id	MSTRG.24612;
3	StringTie	exon	44023826	44024477	.	+	.	transcript_	MSTRG.24612.1	gene_id	MSTRG.24612;
3	StringTie	transcrip	44352216	44355059	.	+	.	transcript_	MSTRG.24630.1	gene_id	MSTRG.24630;
3	StringTie	exon	44352216	44352275	.	+	.	transcript_	MSTRG.24630.1	gene_id	MSTRG.24630;
3	StringTie	exon	44354092	44355059	.	+	.	transcript_	MSTRG.24630.1	gene_id	MSTRG.24630;
3	StringTie	transcrip	52648699	52652979	.	+	.	transcript_	MSTRG.24736.1	gene_id	MSTRG.24736;
3	StringTie	exon	52648699	52648928	.	+	.	transcript_	MSTRG.24736.1	gene_id	MSTRG.24736;
3	StringTie	exon	52650163	52650301	.	+	.	transcript_	MSTRG.24736.1	gene_id	MSTRG.24736;

3	StringTie	exon	52651791	52652979	.	+	.	transcript_	MSTRG.24736.:	gene_id	MSTRG.24736;
3	StringTie	transcrip	58097049	58117254	.	+	.	transcript_	MSTRG.24857.:	gene_id	MSTRG.24857;
3	StringTie	exon	58097049	58097085	.	+	.	transcript_	MSTRG.24857.:	gene_id	MSTRG.24857;
3	StringTie	exon	58098338	58098366	.	+	.	transcript_	MSTRG.24857.:	gene_id	MSTRG.24857;
3	StringTie	exon	58112481	58112532	.	+	.	transcript_	MSTRG.24857.:	gene_id	MSTRG.24857;
3	StringTie	exon	58116765	58117254	.	+	.	transcript_	MSTRG.24857.:	gene_id	MSTRG.24857;
3	StringTie	transcrip	59426152	59450355	.	+	.	transcript_	MSTRG.24955.:	gene_id	MSTRG.24955;
3	StringTie	exon	59426152	59426179	.	+	.	transcript_	MSTRG.24955.:	gene_id	MSTRG.24955;
3	StringTie	exon	59449127	59450355	.	+	.	transcript_	MSTRG.24955.:	gene_id	MSTRG.24955;
3	StringTie	transcrip	71435798	71447325	.	+	.	transcript_	MSTRG.25069.:	gene_id	MSTRG.25069;
3	StringTie	exon	71435798	71435889	.	+	.	transcript_	MSTRG.25069.:	gene_id	MSTRG.25069;
3	StringTie	exon	71444761	71444866	.	+	.	transcript_	MSTRG.25069.:	gene_id	MSTRG.25069;
3	StringTie	exon	71447264	71447325	.	+	.	transcript_	MSTRG.25069.:	gene_id	MSTRG.25069;
3	StringTie	transcrip	72277357	72281811	.	+	.	transcript_	MSTRG.25078.:	gene_id	MSTRG.25078;
3	StringTie	exon	72277357	72278232	.	+	.	transcript_	MSTRG.25078.:	gene_id	MSTRG.25078;
3	StringTie	exon	72279670	72281811	.	+	.	transcript_	MSTRG.25078.:	gene_id	MSTRG.25078;
3	StringTie	transcrip	72596634	72636719	.	+	.	transcript_	MSTRG.25107.:	gene_id	MSTRG.25107;
3	StringTie	exon	72596634	72596655	.	+	.	transcript_	MSTRG.25107.:	gene_id	MSTRG.25107;
3	StringTie	exon	72635308	72636719	.	+	.	transcript_	MSTRG.25107.:	gene_id	MSTRG.25107;
3	StringTie	transcrip	77422247	77423790	.	+	.	transcript_	MSTRG.25178.:	gene_id	MSTRG.25178;
3	StringTie	exon	77422247	77422548	.	+	.	transcript_	MSTRG.25178.:	gene_id	MSTRG.25178;
3	StringTie	exon	77423558	77423790	.	+	.	transcript_	MSTRG.25178.:	gene_id	MSTRG.25178;
3	StringTie	transcrip	77658656	77704950	.	+	.	transcript_	MSTRG.25186.:	gene_id	MSTRG.25186;
3	StringTie	exon	77658656	77658776	.	+	.	transcript_	MSTRG.25186.:	gene_id	MSTRG.25186;
3	StringTie	exon	77670974	77671034	.	+	.	transcript_	MSTRG.25186.:	gene_id	MSTRG.25186;
3	StringTie	exon	77704124	77704272	.	+	.	transcript_	MSTRG.25186.:	gene_id	MSTRG.25186;
3	StringTie	exon	77704888	77704950	.	+	.	transcript_	MSTRG.25186.:	gene_id	MSTRG.25186;
3	StringTie	transcrip	94012811	94013906	.	+	.	transcript_	MSTRG.25446.:	gene_id	MSTRG.25446;
3	StringTie	exon	94012811	94012944	.	+	.	transcript_	MSTRG.25446.:	gene_id	MSTRG.25446;
3	StringTie	exon	94013838	94013906	.	+	.	transcript_	MSTRG.25446.:	gene_id	MSTRG.25446;
3	StringTie	transcrip	94069841	94073841	.	+	.	transcript_	MSTRG.25441.:	gene_id	MSTRG.25441;
3	StringTie	exon	94069841	94069934	.	+	.	transcript_	MSTRG.25441.:	gene_id	MSTRG.25441;
3	StringTie	exon	94073310	94073841	.	+	.	transcript_	MSTRG.25441.:	gene_id	MSTRG.25441;
3	StringTie	transcrip	109175445	109187039	.	+	.	transcript_	MSTRG.25683.:	gene_id	MSTRG.25683;
3	StringTie	exon	109175445	109175483	.	+	.	transcript_	MSTRG.25683.:	gene_id	MSTRG.25683;

3	StringTie	exon	109186167	109187039	.	+	.	transcript_	MSTRG.25683.1	gene_id	MSTRG.25683;
3	StringTie	transcrip	110649034	110652626	.	+	.	transcript_	MSTRG.25710.1	gene_id	MSTRG.25710;
3	StringTie	exon	110649034	110649359	.	+	.	transcript_	MSTRG.25710.1	gene_id	MSTRG.25710;
3	StringTie	exon	110652528	110652626	.	+	.	transcript_	MSTRG.25710.1	gene_id	MSTRG.25710;
3	StringTie	transcrip	115178006	115475934	.	+	.	transcript_	MSTRG.25853.1	gene_id	MSTRG.25853;
3	StringTie	exon	115178006	115178211	.	+	.	transcript_	MSTRG.25853.1	gene_id	MSTRG.25853;
3	StringTie	exon	115218429	115219189	.	+	.	transcript_	MSTRG.25853.1	gene_id	MSTRG.25853;
3	StringTie	exon	115323329	115323417	.	+	.	transcript_	MSTRG.25853.1	gene_id	MSTRG.25853;
3	StringTie	exon	115339722	115339925	.	+	.	transcript_	MSTRG.25853.1	gene_id	MSTRG.25853;
3	StringTie	exon	115440940	115441103	.	+	.	transcript_	MSTRG.25853.1	gene_id	MSTRG.25853;
3	StringTie	exon	115475857	115475934	.	+	.	transcript_	MSTRG.25853.1	gene_id	MSTRG.25853;
3	StringTie	transcrip	115178146	115458542	.	+	.	transcript_	MSTRG.25853.2	gene_id	MSTRG.25853;
3	StringTie	exon	115178146	115178211	.	+	.	transcript_	MSTRG.25853.2	gene_id	MSTRG.25853;
3	StringTie	exon	115218429	115219189	.	+	.	transcript_	MSTRG.25853.2	gene_id	MSTRG.25853;
3	StringTie	exon	115323329	115323417	.	+	.	transcript_	MSTRG.25853.2	gene_id	MSTRG.25853;
3	StringTie	exon	115440940	115441103	.	+	.	transcript_	MSTRG.25853.2	gene_id	MSTRG.25853;
3	StringTie	exon	115456167	115456209	.	+	.	transcript_	MSTRG.25853.2	gene_id	MSTRG.25853;
3	StringTie	exon	115456988	115458542	.	+	.	transcript_	MSTRG.25853.2	gene_id	MSTRG.25853;
3	StringTie	transcrip	115178155	115441199	.	+	.	transcript_	MSTRG.25853.3	gene_id	MSTRG.25853;
3	StringTie	exon	115178155	115178211	.	+	.	transcript_	MSTRG.25853.3	gene_id	MSTRG.25853;
3	StringTie	exon	115218429	115219189	.	+	.	transcript_	MSTRG.25853.3	gene_id	MSTRG.25853;
3	StringTie	exon	115323329	115323417	.	+	.	transcript_	MSTRG.25853.3	gene_id	MSTRG.25853;
3	StringTie	exon	115344701	115344904	.	+	.	transcript_	MSTRG.25853.3	gene_id	MSTRG.25853;
3	StringTie	exon	115440940	115441199	.	+	.	transcript_	MSTRG.25853.3	gene_id	MSTRG.25853;
3	StringTie	transcrip	115218442	115600677	.	+	.	transcript_	MSTRG.25853.4	gene_id	MSTRG.25853;
3	StringTie	exon	115218442	115219189	.	+	.	transcript_	MSTRG.25853.4	gene_id	MSTRG.25853;
3	StringTie	exon	115323329	115323417	.	+	.	transcript_	MSTRG.25853.4	gene_id	MSTRG.25853;
3	StringTie	exon	115344701	115344904	.	+	.	transcript_	MSTRG.25853.4	gene_id	MSTRG.25853;
3	StringTie	exon	115600656	115600677	.	+	.	transcript_	MSTRG.25853.4	gene_id	MSTRG.25853;
3	StringTie	transcrip	115344698	115386338	.	+	.	transcript_	MSTRG.25853.5	gene_id	MSTRG.25853;
3	StringTie	exon	115344698	115344904	.	+	.	transcript_	MSTRG.25853.5	gene_id	MSTRG.25853;
3	StringTie	exon	115386249	115386338	.	+	.	transcript_	MSTRG.25853.5	gene_id	MSTRG.25853;
3	StringTie	transcrip	115453174	115459086	.	+	.	transcript_	MSTRG.25853.6	gene_id	MSTRG.25853;
3	StringTie	exon	115453174	115453870	.	+	.	transcript_	MSTRG.25853.6	gene_id	MSTRG.25853;
3	StringTie	exon	115456167	115456209	.	+	.	transcript_	MSTRG.25853.6	gene_id	MSTRG.25853;

3	StringTie	exon	115456988	115459086	.	+	.	transcript_	MSTRG.25853.	gene_id	MSTRG.25853;
3	StringTie	transcrip	125276327	125276691	.	+	.	transcript_	MSTRG.25956.	gene_id	MSTRG.25956;
3	StringTie	exon	125276327	125276412	.	+	.	transcript_	MSTRG.25956.	gene_id	MSTRG.25956;
3	StringTie	exon	125276494	125276691	.	+	.	transcript_	MSTRG.25956.	gene_id	MSTRG.25956;
3	StringTie	transcrip	127524535	127525392	.	+	.	transcript_	MSTRG.26004.	gene_id	MSTRG.26004;
3	StringTie	exon	127524535	127524649	.	+	.	transcript_	MSTRG.26004.	gene_id	MSTRG.26004;
3	StringTie	exon	127524792	127525392	.	+	.	transcript_	MSTRG.26004.	gene_id	MSTRG.26004;
3	StringTie	transcrip	514634	534637	.	-	.	transcript_	MSTRG.23487.	gene_id	MSTRG.23487;
3	StringTie	exon	514634	515111	.	-	.	transcript_	MSTRG.23487.	gene_id	MSTRG.23487;
3	StringTie	exon	534479	534637	.	-	.	transcript_	MSTRG.23487.	gene_id	MSTRG.23487;
3	StringTie	transcrip	514653	534593	.	-	.	transcript_	MSTRG.23487.	gene_id	MSTRG.23487;
3	StringTie	exon	514653	515111	.	-	.	transcript_	MSTRG.23487.	gene_id	MSTRG.23487;
3	StringTie	exon	534418	534593	.	-	.	transcript_	MSTRG.23487.	gene_id	MSTRG.23487;
3	StringTie	transcrip	5039318	5116873	.	-	.	transcript_	MSTRG.23587.	gene_id	MSTRG.23587;
3	StringTie	exon	5039318	5039364	.	-	.	transcript_	MSTRG.23587.	gene_id	MSTRG.23587;
3	StringTie	exon	5115975	5116873	.	-	.	transcript_	MSTRG.23587.	gene_id	MSTRG.23587;
3	StringTie	transcrip	5104095	5145105	.	-	.	transcript_	MSTRG.23592.	gene_id	MSTRG.23592;
3	StringTie	exon	5104095	5104336	.	-	.	transcript_	MSTRG.23592.	gene_id	MSTRG.23592;
3	StringTie	exon	5144995	5145105	.	-	.	transcript_	MSTRG.23592.	gene_id	MSTRG.23592;
3	StringTie	transcrip	5462559	5463263	.	-	.	transcript_	MSTRG.23581.	gene_id	MSTRG.23581;
3	StringTie	exon	5462559	5462985	.	-	.	transcript_	MSTRG.23581.	gene_id	MSTRG.23581;
3	StringTie	exon	5463245	5463263	.	-	.	transcript_	MSTRG.23581.	gene_id	MSTRG.23581;
3	StringTie	transcrip	6265573	6296207	.	-	.	transcript_	MSTRG.23606.	gene_id	MSTRG.23606;
3	StringTie	exon	6265573	6265640	.	-	.	transcript_	MSTRG.23606.	gene_id	MSTRG.23606;
3	StringTie	exon	6295706	6295957	.	-	.	transcript_	MSTRG.23606.	gene_id	MSTRG.23606;
3	StringTie	exon	6296129	6296207	.	-	.	transcript_	MSTRG.23606.	gene_id	MSTRG.23606;
3	StringTie	transcrip	8484021	8494347	.	-	.	transcript_	MSTRG.23704.	gene_id	MSTRG.23704;
3	StringTie	exon	8484021	8484297	.	-	.	transcript_	MSTRG.23704.	gene_id	MSTRG.23704;
3	StringTie	exon	8484907	8484994	.	-	.	transcript_	MSTRG.23704.	gene_id	MSTRG.23704;
3	StringTie	exon	8487888	8488058	.	-	.	transcript_	MSTRG.23704.	gene_id	MSTRG.23704;
3	StringTie	exon	8491163	8491288	.	-	.	transcript_	MSTRG.23704.	gene_id	MSTRG.23704;
3	StringTie	exon	8493798	8493938	.	-	.	transcript_	MSTRG.23704.	gene_id	MSTRG.23704;
3	StringTie	exon	8494260	8494347	.	-	.	transcript_	MSTRG.23704.	gene_id	MSTRG.23704;
3	StringTie	transcrip	8484021	8494874	.	-	.	transcript_	MSTRG.23704.	gene_id	MSTRG.23704;
3	StringTie	exon	8484021	8484994	.	-	.	transcript_	MSTRG.23704.	gene_id	MSTRG.23704;

3	StringTie	exon	8487888	8488058	.	-	.	transcript_	MSTRG.23704.9	gene_id	MSTRG.23704;
3	StringTie	exon	8491163	8491288	.	-	.	transcript_	MSTRG.23704.9	gene_id	MSTRG.23704;
3	StringTie	exon	8492155	8492284	.	-	.	transcript_	MSTRG.23704.9	gene_id	MSTRG.23704;
3	StringTie	exon	8493759	8493938	.	-	.	transcript_	MSTRG.23704.9	gene_id	MSTRG.23704;
3	StringTie	exon	8494766	8494874	.	-	.	transcript_	MSTRG.23704.9	gene_id	MSTRG.23704;
3	StringTie	transcrip	8484021	8494874	.	-	.	transcript_	MSTRG.23704.1	gene_id	MSTRG.23704;
3	StringTie	exon	8484021	8484994	.	-	.	transcript_	MSTRG.23704.1	gene_id	MSTRG.23704;
3	StringTie	exon	8487888	8488058	.	-	.	transcript_	MSTRG.23704.1	gene_id	MSTRG.23704;
3	StringTie	exon	8491163	8491288	.	-	.	transcript_	MSTRG.23704.1	gene_id	MSTRG.23704;
3	StringTie	exon	8493798	8493938	.	-	.	transcript_	MSTRG.23704.1	gene_id	MSTRG.23704;
3	StringTie	exon	8494766	8494874	.	-	.	transcript_	MSTRG.23704.1	gene_id	MSTRG.23704;
3	StringTie	transcrip	8484021	8494874	.	-	.	transcript_	MSTRG.23704.1	gene_id	MSTRG.23704;
3	StringTie	exon	8484021	8484297	.	-	.	transcript_	MSTRG.23704.1	gene_id	MSTRG.23704;
3	StringTie	exon	8484907	8485275	.	-	.	transcript_	MSTRG.23704.1	gene_id	MSTRG.23704;
3	StringTie	exon	8487888	8488058	.	-	.	transcript_	MSTRG.23704.1	gene_id	MSTRG.23704;
3	StringTie	exon	8491163	8491288	.	-	.	transcript_	MSTRG.23704.1	gene_id	MSTRG.23704;
3	StringTie	exon	8492155	8492284	.	-	.	transcript_	MSTRG.23704.1	gene_id	MSTRG.23704;
3	StringTie	exon	8493798	8493938	.	-	.	transcript_	MSTRG.23704.1	gene_id	MSTRG.23704;
3	StringTie	exon	8494766	8494874	.	-	.	transcript_	MSTRG.23704.1	gene_id	MSTRG.23704;
3	StringTie	transcrip	9644597	9660698	.	-	.	transcript_	MSTRG.23733.1	gene_id	MSTRG.23733;
3	StringTie	exon	9644597	9653248	.	-	.	transcript_	MSTRG.23733.1	gene_id	MSTRG.23733;
3	StringTie	exon	9656257	9656352	.	-	.	transcript_	MSTRG.23733.1	gene_id	MSTRG.23733;
3	StringTie	exon	9660495	9660698	.	-	.	transcript_	MSTRG.23733.1	gene_id	MSTRG.23733;
3	StringTie	transcrip	10738582	10740699	.	-	.	transcript_	MSTRG.23763.1	gene_id	MSTRG.23763;
3	StringTie	exon	10738582	10738839	.	-	.	transcript_	MSTRG.23763.1	gene_id	MSTRG.23763;
3	StringTie	exon	10740556	10740699	.	-	.	transcript_	MSTRG.23763.1	gene_id	MSTRG.23763;
3	StringTie	transcrip	16789321	16812394	.	-	.	transcript_	MSTRG.23889.1	gene_id	MSTRG.23889;
3	StringTie	exon	16789321	16789848	.	-	.	transcript_	MSTRG.23889.1	gene_id	MSTRG.23889;
3	StringTie	exon	16790736	16790834	.	-	.	transcript_	MSTRG.23889.1	gene_id	MSTRG.23889;
3	StringTie	exon	16803175	16803341	.	-	.	transcript_	MSTRG.23889.1	gene_id	MSTRG.23889;
3	StringTie	exon	16812179	16812394	.	-	.	transcript_	MSTRG.23889.1	gene_id	MSTRG.23889;
3	StringTie	transcrip	16790736	16812286	.	-	.	transcript_	MSTRG.23889.2	gene_id	MSTRG.23889;
3	StringTie	exon	16790736	16790834	.	-	.	transcript_	MSTRG.23889.2	gene_id	MSTRG.23889;
3	StringTie	exon	16812179	16812286	.	-	.	transcript_	MSTRG.23889.2	gene_id	MSTRG.23889;
3	StringTie	transcrip	16802180	16802993	.	-	.	transcript_	MSTRG.23890.1	gene_id	MSTRG.23890;

3	StringTie	exon	16802180	16802474	.	-	.	transcript_	MSTRG.23890.1	gene_id	MSTRG.23890;
3	StringTie	exon	16802973	16802993	.	-	.	transcript_	MSTRG.23890.1	gene_id	MSTRG.23890;
3	StringTie	transcrip	17556266	17559085	.	-	.	transcript_	MSTRG.23951.1	gene_id	MSTRG.23951;
3	StringTie	exon	17556266	17556671	.	-	.	transcript_	MSTRG.23951.1	gene_id	MSTRG.23951;
3	StringTie	exon	17556923	17557106	.	-	.	transcript_	MSTRG.23951.1	gene_id	MSTRG.23951;
3	StringTie	exon	17559010	17559085	.	-	.	transcript_	MSTRG.23951.1	gene_id	MSTRG.23951;
3	StringTie	transcrip	17585836	17604052	.	-	.	transcript_	MSTRG.23955.1	gene_id	MSTRG.23955;
3	StringTie	exon	17585836	17589766	.	-	.	transcript_	MSTRG.23955.1	gene_id	MSTRG.23955;
3	StringTie	exon	17594310	17594509	.	-	.	transcript_	MSTRG.23955.1	gene_id	MSTRG.23955;
3	StringTie	exon	17603781	17604052	.	-	.	transcript_	MSTRG.23955.1	gene_id	MSTRG.23955;
3	StringTie	transcrip	18500840	18507347	.	-	.	transcript_	MSTRG.24036.1	gene_id	MSTRG.24036;
3	StringTie	exon	18500840	18501111	.	-	.	transcript_	MSTRG.24036.1	gene_id	MSTRG.24036;
3	StringTie	exon	18507257	18507347	.	-	.	transcript_	MSTRG.24036.1	gene_id	MSTRG.24036;
3	StringTie	transcrip	23799238	23802719	.	-	.	transcript_	MSTRG.24099.1	gene_id	MSTRG.24099;
3	StringTie	exon	23799238	23799445	.	-	.	transcript_	MSTRG.24099.1	gene_id	MSTRG.24099;
3	StringTie	exon	23802573	23802719	.	-	.	transcript_	MSTRG.24099.1	gene_id	MSTRG.24099;
3	StringTie	transcrip	28739641	28743098	.	-	.	transcript_	MSTRG.24197.1	gene_id	MSTRG.24197;
3	StringTie	exon	28739641	28739716	.	-	.	transcript_	MSTRG.24197.1	gene_id	MSTRG.24197;
3	StringTie	exon	28740044	28740133	.	-	.	transcript_	MSTRG.24197.1	gene_id	MSTRG.24197;
3	StringTie	exon	28740406	28740519	.	-	.	transcript_	MSTRG.24197.1	gene_id	MSTRG.24197;
3	StringTie	exon	28742998	28743098	.	-	.	transcript_	MSTRG.24197.1	gene_id	MSTRG.24197;
3	StringTie	transcrip	31324344	31332384	.	-	.	transcript_	MSTRG.24223.1	gene_id	MSTRG.24223;
3	StringTie	exon	31324344	31326131	.	-	.	transcript_	MSTRG.24223.1	gene_id	MSTRG.24223;
3	StringTie	exon	31330873	31330981	.	-	.	transcript_	MSTRG.24223.1	gene_id	MSTRG.24223;
3	StringTie	exon	31332362	31332384	.	-	.	transcript_	MSTRG.24223.1	gene_id	MSTRG.24223;
3	StringTie	transcrip	31324667	31326035	.	-	.	transcript_	MSTRG.24223.2	gene_id	MSTRG.24223;
3	StringTie	exon	31324667	31325572	.	-	.	transcript_	MSTRG.24223.2	gene_id	MSTRG.24223;
3	StringTie	exon	31325879	31326035	.	-	.	transcript_	MSTRG.24223.2	gene_id	MSTRG.24223;
3	StringTie	transcrip	38084956	38088744	.	-	.	transcript_	MSTRG.24302.1	gene_id	MSTRG.24302;
3	StringTie	exon	38084956	38086332	.	-	.	transcript_	MSTRG.24302.1	gene_id	MSTRG.24302;
3	StringTie	exon	38086680	38088744	.	-	.	transcript_	MSTRG.24302.1	gene_id	MSTRG.24302;
3	StringTie	transcrip	38782798	38789693	.	-	.	transcript_	MSTRG.24321.1	gene_id	MSTRG.24321;
3	StringTie	exon	38782798	38783855	.	-	.	transcript_	MSTRG.24321.1	gene_id	MSTRG.24321;
3	StringTie	exon	38789673	38789693	.	-	.	transcript_	MSTRG.24321.1	gene_id	MSTRG.24321;
3	StringTie	transcrip	39624688	39626094	.	-	.	transcript_	MSTRG.24377.1	gene_id	MSTRG.24377;

3	StringTie	exon	39624688	39624734	.	-	.	transcript_	MSTRG.24377.1	gene_id	MSTRG.24377;
3	StringTie	exon	39625634	39625756	.	-	.	transcript_	MSTRG.24377.1	gene_id	MSTRG.24377;
3	StringTie	exon	39626048	39626094	.	-	.	transcript_	MSTRG.24377.1	gene_id	MSTRG.24377;
3	StringTie	transcrip	40170167	40172364	.	-	.	transcript_	MSTRG.24429.1	gene_id	MSTRG.24429;
3	StringTie	exon	40170167	40170493	.	-	.	transcript_	MSTRG.24429.1	gene_id	MSTRG.24429;
3	StringTie	exon	40172127	40172364	.	-	.	transcript_	MSTRG.24429.1	gene_id	MSTRG.24429;
3	StringTie	transcrip	40931972	40956077	.	-	.	transcript_	MSTRG.24467.1	gene_id	MSTRG.24467;
3	StringTie	exon	40931972	40935319	.	-	.	transcript_	MSTRG.24467.1	gene_id	MSTRG.24467;
3	StringTie	exon	40946893	40947020	.	-	.	transcript_	MSTRG.24467.1	gene_id	MSTRG.24467;
3	StringTie	exon	40954688	40954758	.	-	.	transcript_	MSTRG.24467.1	gene_id	MSTRG.24467;
3	StringTie	exon	40955261	40955420	.	-	.	transcript_	MSTRG.24467.1	gene_id	MSTRG.24467;
3	StringTie	exon	40955684	40956077	.	-	.	transcript_	MSTRG.24467.1	gene_id	MSTRG.24467;
3	StringTie	transcrip	41864618	41957527	.	-	.	transcript_	MSTRG.24523.1	gene_id	MSTRG.24523;
3	StringTie	exon	41864618	41864668	.	-	.	transcript_	MSTRG.24523.1	gene_id	MSTRG.24523;
3	StringTie	exon	41956353	41957527	.	-	.	transcript_	MSTRG.24523.1	gene_id	MSTRG.24523;
3	StringTie	transcrip	41880828	41957716	.	-	.	transcript_	MSTRG.24523.2	gene_id	MSTRG.24523;
3	StringTie	exon	41880828	41880872	.	-	.	transcript_	MSTRG.24523.2	gene_id	MSTRG.24523;
3	StringTie	exon	41956346	41957716	.	-	.	transcript_	MSTRG.24523.2	gene_id	MSTRG.24523;
3	StringTie	transcrip	42857790	42861300	.	-	.	transcript_	MSTRG.24546.1	gene_id	MSTRG.24546;
3	StringTie	exon	42857790	42859467	.	-	.	transcript_	MSTRG.24546.1	gene_id	MSTRG.24546;
3	StringTie	exon	42860670	42861300	.	-	.	transcript_	MSTRG.24546.1	gene_id	MSTRG.24546;
3	StringTie	transcrip	42978277	42987096	.	-	.	transcript_	MSTRG.24544.1	gene_id	MSTRG.24544;
3	StringTie	exon	42978277	42979203	.	-	.	transcript_	MSTRG.24544.1	gene_id	MSTRG.24544;
3	StringTie	exon	42981340	42981450	.	-	.	transcript_	MSTRG.24544.1	gene_id	MSTRG.24544;
3	StringTie	exon	42987018	42987096	.	-	.	transcript_	MSTRG.24544.1	gene_id	MSTRG.24544;
3	StringTie	transcrip	47654641	47655028	.	-	.	transcript_	MSTRG.24689.1	gene_id	MSTRG.24689;
3	StringTie	exon	47654641	47654700	.	-	.	transcript_	MSTRG.24689.1	gene_id	MSTRG.24689;
3	StringTie	exon	47654879	47655028	.	-	.	transcript_	MSTRG.24689.1	gene_id	MSTRG.24689;
3	StringTie	transcrip	58206338	58206841	.	-	.	transcript_	MSTRG.24865.1	gene_id	MSTRG.24865;
3	StringTie	exon	58206338	58206514	.	-	.	transcript_	MSTRG.24865.1	gene_id	MSTRG.24865;
3	StringTie	exon	58206732	58206841	.	-	.	transcript_	MSTRG.24865.1	gene_id	MSTRG.24865;
3	StringTie	transcrip	68071222	68074095	.	-	.	transcript_	MSTRG.24980.1	gene_id	MSTRG.24980;
3	StringTie	exon	68071222	68071434	.	-	.	transcript_	MSTRG.24980.1	gene_id	MSTRG.24980;
3	StringTie	exon	68074034	68074095	.	-	.	transcript_	MSTRG.24980.1	gene_id	MSTRG.24980;
3	StringTie	transcrip	69702082	69702886	.	-	.	transcript_	MSTRG.25045.1	gene_id	MSTRG.25045;

3	StringTie	exon	69702082	69702303	.	-	.	transcript_	MSTRG.25045.1	gene_id	MSTRG.25045;
3	StringTie	exon	69702774	69702886	.	-	.	transcript_	MSTRG.25045.1	gene_id	MSTRG.25045;
3	StringTie	transcrip	72769581	72772322	.	-	.	transcript_	MSTRG.25108.1	gene_id	MSTRG.25108;
3	StringTie	exon	72769581	72769799	.	-	.	transcript_	MSTRG.25108.1	gene_id	MSTRG.25108;
3	StringTie	exon	72772197	72772322	.	-	.	transcript_	MSTRG.25108.1	gene_id	MSTRG.25108;
3	StringTie	transcrip	79991716	80033945	.	-	.	transcript_	MSTRG.25275.1	gene_id	MSTRG.25275;
3	StringTie	exon	79991716	79993340	.	-	.	transcript_	MSTRG.25275.1	gene_id	MSTRG.25275;
3	StringTie	exon	80019192	80019244	.	-	.	transcript_	MSTRG.25275.1	gene_id	MSTRG.25275;
3	StringTie	exon	80028090	80028166	.	-	.	transcript_	MSTRG.25275.1	gene_id	MSTRG.25275;
3	StringTie	exon	80033880	80033945	.	-	.	transcript_	MSTRG.25275.1	gene_id	MSTRG.25275;
3	StringTie	transcrip	80030842	80034478	.	-	.	transcript_	MSTRG.25276.1	gene_id	MSTRG.25276;
3	StringTie	exon	80030842	80031336	.	-	.	transcript_	MSTRG.25276.1	gene_id	MSTRG.25276;
3	StringTie	exon	80034161	80034478	.	-	.	transcript_	MSTRG.25276.1	gene_id	MSTRG.25276;
3	StringTie	transcrip	85265117	85335671	.	-	.	transcript_	MSTRG.25319.1	gene_id	MSTRG.25319;
3	StringTie	exon	85265117	85265366	.	-	.	transcript_	MSTRG.25319.1	gene_id	MSTRG.25319;
3	StringTie	exon	85335583	85335671	.	-	.	transcript_	MSTRG.25319.1	gene_id	MSTRG.25319;
3	StringTie	transcrip	92704343	92717698	.	-	.	transcript_	MSTRG.25424.1	gene_id	MSTRG.25424;
3	StringTie	exon	92704343	92704371	.	-	.	transcript_	MSTRG.25424.1	gene_id	MSTRG.25424;
3	StringTie	exon	92713361	92717698	.	-	.	transcript_	MSTRG.25424.1	gene_id	MSTRG.25424;
3	StringTie	transcrip	92704343	92803272	.	-	.	transcript_	MSTRG.25424.2	gene_id	MSTRG.25424;
3	StringTie	exon	92704343	92704371	.	-	.	transcript_	MSTRG.25424.2	gene_id	MSTRG.25424;
3	StringTie	exon	92802913	92803272	.	-	.	transcript_	MSTRG.25424.2	gene_id	MSTRG.25424;
3	StringTie	transcrip	93564738	93573296	.	-	.	transcript_	MSTRG.25431.1	gene_id	MSTRG.25431;
3	StringTie	exon	93564738	93564754	.	-	.	transcript_	MSTRG.25431.1	gene_id	MSTRG.25431;
3	StringTie	exon	93573090	93573296	.	-	.	transcript_	MSTRG.25431.1	gene_id	MSTRG.25431;
3	StringTie	transcrip	111347433	111351484	.	-	.	transcript_	MSTRG.25717.1	gene_id	MSTRG.25717;
3	StringTie	exon	111347433	111347485	.	-	.	transcript_	MSTRG.25717.1	gene_id	MSTRG.25717;
3	StringTie	exon	111350542	111351484	.	-	.	transcript_	MSTRG.25717.1	gene_id	MSTRG.25717;
3	StringTie	transcrip	111930026	111930362	.	-	.	transcript_	MSTRG.25763.1	gene_id	MSTRG.25763;
3	StringTie	exon	111930026	111930134	.	-	.	transcript_	MSTRG.25763.1	gene_id	MSTRG.25763;
3	StringTie	exon	111930252	111930362	.	-	.	transcript_	MSTRG.25763.1	gene_id	MSTRG.25763;
3	StringTie	transcrip	114275242	114311997	.	-	.	transcript_	MSTRG.25815.1	gene_id	MSTRG.25815;
3	StringTie	exon	114275242	114275873	.	-	.	transcript_	MSTRG.25815.1	gene_id	MSTRG.25815;
3	StringTie	exon	114280516	114280643	.	-	.	transcript_	MSTRG.25815.1	gene_id	MSTRG.25815;
3	StringTie	exon	114284033	114284074	.	-	.	transcript_	MSTRG.25815.1	gene_id	MSTRG.25815;

3	StringTie	exon	114311977	114311997	.	-	.	transcript_	MSTRG.25815.:	gene_id	MSTRG.25815;
3	StringTie	transcrip	120868281	120869536	.	-	.	transcript_	MSTRG.25925.:	gene_id	MSTRG.25925;
3	StringTie	exon	120868281	120868928	.	-	.	transcript_	MSTRG.25925.:	gene_id	MSTRG.25925;
3	StringTie	exon	120869424	120869536	.	-	.	transcript_	MSTRG.25925.:	gene_id	MSTRG.25925;
3	StringTie	transcrip	124457909	124461094	.	-	.	transcript_	MSTRG.25941.:	gene_id	MSTRG.25941;
3	StringTie	exon	124457909	124459343	.	-	.	transcript_	MSTRG.25941.:	gene_id	MSTRG.25941;
3	StringTie	exon	124460978	124461094	.	-	.	transcript_	MSTRG.25941.:	gene_id	MSTRG.25941;
3	StringTie	transcrip	127869858	127874239	.	-	.	transcript_	MSTRG.25995.:	gene_id	MSTRG.25995;
3	StringTie	exon	127869858	127870152	.	-	.	transcript_	MSTRG.25995.:	gene_id	MSTRG.25995;
3	StringTie	exon	127870991	127871133	.	-	.	transcript_	MSTRG.25995.:	gene_id	MSTRG.25995;
3	StringTie	exon	127874148	127874239	.	-	.	transcript_	MSTRG.25995.:	gene_id	MSTRG.25995;
4	StringTie	transcrip	1592968	1595232	.	+	.	transcript_	MSTRG.26148.:	gene_id	MSTRG.26148;
4	StringTie	exon	1592968	1593049	.	+	.	transcript_	MSTRG.26148.:	gene_id	MSTRG.26148;
4	StringTie	exon	1594666	1595232	.	+	.	transcript_	MSTRG.26148.:	gene_id	MSTRG.26148;
4	StringTie	transcrip	2635321	2641645	.	+	.	transcript_	MSTRG.26164.:	gene_id	MSTRG.26164;
4	StringTie	exon	2635321	2635875	.	+	.	transcript_	MSTRG.26164.:	gene_id	MSTRG.26164;
4	StringTie	exon	2636850	2637051	.	+	.	transcript_	MSTRG.26164.:	gene_id	MSTRG.26164;
4	StringTie	exon	2637394	2641645	.	+	.	transcript_	MSTRG.26164.:	gene_id	MSTRG.26164;
4	StringTie	transcrip	7372566	7373247	.	+	.	transcript_	MSTRG.26194.:	gene_id	MSTRG.26194;
4	StringTie	exon	7372566	7372627	.	+	.	transcript_	MSTRG.26194.:	gene_id	MSTRG.26194;
4	StringTie	exon	7372958	7373247	.	+	.	transcript_	MSTRG.26194.:	gene_id	MSTRG.26194;
4	StringTie	transcrip	14402984	14403905	.	+	.	transcript_	MSTRG.26238.:	gene_id	MSTRG.26238;
4	StringTie	exon	14402984	14403463	.	+	.	transcript_	MSTRG.26238.:	gene_id	MSTRG.26238;
4	StringTie	exon	14403873	14403905	.	+	.	transcript_	MSTRG.26238.:	gene_id	MSTRG.26238;
4	StringTie	transcrip	15396200	15397224	.	+	.	transcript_	MSTRG.26294.:	gene_id	MSTRG.26294;
4	StringTie	exon	15396200	15396389	.	+	.	transcript_	MSTRG.26294.:	gene_id	MSTRG.26294;
4	StringTie	exon	15396593	15396703	.	+	.	transcript_	MSTRG.26294.:	gene_id	MSTRG.26294;
4	StringTie	exon	15396916	15397224	.	+	.	transcript_	MSTRG.26294.:	gene_id	MSTRG.26294;
4	StringTie	transcrip	17192373	17281592	.	+	.	transcript_	MSTRG.26349.:	gene_id	MSTRG.26349;
4	StringTie	exon	17192373	17192550	.	+	.	transcript_	MSTRG.26349.:	gene_id	MSTRG.26349;
4	StringTie	exon	17279705	17279823	.	+	.	transcript_	MSTRG.26349.:	gene_id	MSTRG.26349;
4	StringTie	exon	17281182	17281592	.	+	.	transcript_	MSTRG.26349.:	gene_id	MSTRG.26349;
4	StringTie	transcrip	29088411	29164887	.	+	.	transcript_	MSTRG.26506.:	gene_id	MSTRG.26506;
4	StringTie	exon	29088411	29088487	.	+	.	transcript_	MSTRG.26506.:	gene_id	MSTRG.26506;
4	StringTie	exon	29164225	29164384	.	+	.	transcript_	MSTRG.26506.:	gene_id	MSTRG.26506;

4	StringTie	exon	29164774	29164887	.	+	.	transcript_	MSTRG.26506.1	gene_id	MSTRG.26506;
4	StringTie	transcrip	30277717	30491337	.	+	.	transcript_	MSTRG.26518.1	gene_id	MSTRG.26518;
4	StringTie	exon	30277717	30277840	.	+	.	transcript_	MSTRG.26518.1	gene_id	MSTRG.26518;
4	StringTie	exon	30277969	30278076	.	+	.	transcript_	MSTRG.26518.1	gene_id	MSTRG.26518;
4	StringTie	exon	30297124	30297191	.	+	.	transcript_	MSTRG.26518.1	gene_id	MSTRG.26518;
4	StringTie	exon	30399078	30399394	.	+	.	transcript_	MSTRG.26518.1	gene_id	MSTRG.26518;
4	StringTie	exon	30491085	30491337	.	+	.	transcript_	MSTRG.26518.1	gene_id	MSTRG.26518;
4	StringTie	transcrip	30277980	30522672	.	+	.	transcript_	MSTRG.26518.2	gene_id	MSTRG.26518;
4	StringTie	exon	30277980	30278076	.	+	.	transcript_	MSTRG.26518.2	gene_id	MSTRG.26518;
4	StringTie	exon	30317117	30317213	.	+	.	transcript_	MSTRG.26518.2	gene_id	MSTRG.26518;
4	StringTie	exon	30399078	30399394	.	+	.	transcript_	MSTRG.26518.2	gene_id	MSTRG.26518;
4	StringTie	exon	30494321	30494414	.	+	.	transcript_	MSTRG.26518.2	gene_id	MSTRG.26518;
4	StringTie	exon	30522417	30522672	.	+	.	transcript_	MSTRG.26518.2	gene_id	MSTRG.26518;
4	StringTie	transcrip	30875374	30952439	.	+	.	transcript_	MSTRG.26525.1	gene_id	MSTRG.26525;
4	StringTie	exon	30875374	30875545	.	+	.	transcript_	MSTRG.26525.1	gene_id	MSTRG.26525;
4	StringTie	exon	30882299	30882381	.	+	.	transcript_	MSTRG.26525.1	gene_id	MSTRG.26525;
4	StringTie	exon	30952345	30952439	.	+	.	transcript_	MSTRG.26525.1	gene_id	MSTRG.26525;
4	StringTie	transcrip	30953243	30959437	.	+	.	transcript_	MSTRG.26526.1	gene_id	MSTRG.26526;
4	StringTie	exon	30953243	30953269	.	+	.	transcript_	MSTRG.26526.1	gene_id	MSTRG.26526;
4	StringTie	exon	30959046	30959437	.	+	.	transcript_	MSTRG.26526.1	gene_id	MSTRG.26526;
4	StringTie	transcrip	36744626	36748061	.	+	.	transcript_	MSTRG.26655.1	gene_id	MSTRG.26655;
4	StringTie	exon	36744626	36745809	.	+	.	transcript_	MSTRG.26655.1	gene_id	MSTRG.26655;
4	StringTie	exon	36745996	36746141	.	+	.	transcript_	MSTRG.26655.1	gene_id	MSTRG.26655;
4	StringTie	exon	36747960	36748061	.	+	.	transcript_	MSTRG.26655.1	gene_id	MSTRG.26655;
4	StringTie	transcrip	41665422	41666932	.	+	.	transcript_	MSTRG.26726.1	gene_id	MSTRG.26726;
4	StringTie	exon	41665422	41665601	.	+	.	transcript_	MSTRG.26726.1	gene_id	MSTRG.26726;
4	StringTie	exon	41666036	41666932	.	+	.	transcript_	MSTRG.26726.1	gene_id	MSTRG.26726;
4	StringTie	transcrip	41918417	41925610	.	+	.	transcript_	MSTRG.26730.1	gene_id	MSTRG.26730;
4	StringTie	exon	41918417	41918476	.	+	.	transcript_	MSTRG.26730.1	gene_id	MSTRG.26730;
4	StringTie	exon	41921505	41925610	.	+	.	transcript_	MSTRG.26730.1	gene_id	MSTRG.26730;
4	StringTie	transcrip	42658580	42665764	.	+	.	transcript_	MSTRG.26738.1	gene_id	MSTRG.26738;
4	StringTie	exon	42658580	42659007	.	+	.	transcript_	MSTRG.26738.1	gene_id	MSTRG.26738;
4	StringTie	exon	42665739	42665764	.	+	.	transcript_	MSTRG.26738.1	gene_id	MSTRG.26738;
4	StringTie	transcrip	45793523	45795979	.	+	.	transcript_	MSTRG.26800.2	gene_id	MSTRG.26800;
4	StringTie	exon	45793523	45793655	.	+	.	transcript_	MSTRG.26800.2	gene_id	MSTRG.26800;

4	StringTie	exon	45793769	45794562	.	+	.	transcript_	MSTRG.26800.2	gene_id	MSTRG.26800;
4	StringTie	exon	45794583	45795979	.	+	.	transcript_	MSTRG.26800.2	gene_id	MSTRG.26800;
4	StringTie	transcrip	45793523	45796112	.	+	.	transcript_	MSTRG.26800.1	gene_id	MSTRG.26800;
4	StringTie	exon	45793523	45793655	.	+	.	transcript_	MSTRG.26800.1	gene_id	MSTRG.26800;
4	StringTie	exon	45793769	45796112	.	+	.	transcript_	MSTRG.26800.1	gene_id	MSTRG.26800;
4	StringTie	transcrip	45793523	45804129	.	+	.	transcript_	MSTRG.26800.3	gene_id	MSTRG.26800;
4	StringTie	exon	45793523	45793655	.	+	.	transcript_	MSTRG.26800.3	gene_id	MSTRG.26800;
4	StringTie	exon	45803972	45804129	.	+	.	transcript_	MSTRG.26800.3	gene_id	MSTRG.26800;
4	StringTie	transcrip	53903890	53954298	.	+	.	transcript_	MSTRG.26863.1	gene_id	MSTRG.26863;
4	StringTie	exon	53903890	53903906	.	+	.	transcript_	MSTRG.26863.1	gene_id	MSTRG.26863;
4	StringTie	exon	53953609	53954298	.	+	.	transcript_	MSTRG.26863.1	gene_id	MSTRG.26863;
4	StringTie	transcrip	70928896	70933668	.	+	.	transcript_	MSTRG.27113.3	gene_id	MSTRG.27113;
4	StringTie	exon	70928896	70929135	.	+	.	transcript_	MSTRG.27113.3	gene_id	MSTRG.27113;
4	StringTie	exon	70933461	70933668	.	+	.	transcript_	MSTRG.27113.3	gene_id	MSTRG.27113;
4	StringTie	transcrip	70928940	70941808	.	+	.	transcript_	MSTRG.27113.5	gene_id	MSTRG.27113;
4	StringTie	exon	70928940	70929456	.	+	.	transcript_	MSTRG.27113.5	gene_id	MSTRG.27113;
4	StringTie	exon	70933461	70941808	.	+	.	transcript_	MSTRG.27113.5	gene_id	MSTRG.27113;
4	StringTie	transcrip	72148130	72150953	.	+	.	transcript_	MSTRG.27128.1	gene_id	MSTRG.27128;
4	StringTie	exon	72148130	72148248	.	+	.	transcript_	MSTRG.27128.1	gene_id	MSTRG.27128;
4	StringTie	exon	72150670	72150953	.	+	.	transcript_	MSTRG.27128.1	gene_id	MSTRG.27128;
4	StringTie	transcrip	72326408	72332234	.	+	.	transcript_	MSTRG.27119.1	gene_id	MSTRG.27119;
4	StringTie	exon	72326408	72326456	.	+	.	transcript_	MSTRG.27119.1	gene_id	MSTRG.27119;
4	StringTie	exon	72331920	72332234	.	+	.	transcript_	MSTRG.27119.1	gene_id	MSTRG.27119;
4	StringTie	transcrip	72542203	72543586	.	+	.	transcript_	MSTRG.27122.1	gene_id	MSTRG.27122;
4	StringTie	exon	72542203	72542356	.	+	.	transcript_	MSTRG.27122.1	gene_id	MSTRG.27122;
4	StringTie	exon	72543185	72543586	.	+	.	transcript_	MSTRG.27122.1	gene_id	MSTRG.27122;
4	StringTie	transcrip	73297935	73303071	.	+	.	transcript_	MSTRG.27140.1	gene_id	MSTRG.27140;
4	StringTie	exon	73297935	73297960	.	+	.	transcript_	MSTRG.27140.1	gene_id	MSTRG.27140;
4	StringTie	exon	73302808	73303071	.	+	.	transcript_	MSTRG.27140.1	gene_id	MSTRG.27140;
4	StringTie	transcrip	74125201	74212951	.	+	.	transcript_	MSTRG.27141.1	gene_id	MSTRG.27141;
4	StringTie	exon	74125201	74125248	.	+	.	transcript_	MSTRG.27141.1	gene_id	MSTRG.27141;
4	StringTie	exon	74212762	74212951	.	+	.	transcript_	MSTRG.27141.1	gene_id	MSTRG.27141;
4	StringTie	transcrip	75959122	75978040	.	+	.	transcript_	MSTRG.27221.1	gene_id	MSTRG.27221;
4	StringTie	exon	75959122	75959139	.	+	.	transcript_	MSTRG.27221.1	gene_id	MSTRG.27221;
4	StringTie	exon	75977135	75978040	.	+	.	transcript_	MSTRG.27221.1	gene_id	MSTRG.27221;

4	StringTie	transcrip	80190932	80204507	.	+	.	transcript_	MSTRG.27284.1	gene_id	MSTRG.27284;
4	StringTie	exon	80190932	80191094	.	+	.	transcript_	MSTRG.27284.1	gene_id	MSTRG.27284;
4	StringTie	exon	80204430	80204507	.	+	.	transcript_	MSTRG.27284.1	gene_id	MSTRG.27284;
4	StringTie	transcrip	80190986	80451420	.	+	.	transcript_	MSTRG.27284.2	gene_id	MSTRG.27284;
4	StringTie	exon	80190986	80191094	.	+	.	transcript_	MSTRG.27284.2	gene_id	MSTRG.27284;
4	StringTie	exon	80295694	80295794	.	+	.	transcript_	MSTRG.27284.2	gene_id	MSTRG.27284;
4	StringTie	exon	80450832	80451420	.	+	.	transcript_	MSTRG.27284.2	gene_id	MSTRG.27284;
4	StringTie	transcrip	80191051	80451676	.	+	.	transcript_	MSTRG.27284.3	gene_id	MSTRG.27284;
4	StringTie	exon	80191051	80191094	.	+	.	transcript_	MSTRG.27284.3	gene_id	MSTRG.27284;
4	StringTie	exon	80295691	80295794	.	+	.	transcript_	MSTRG.27284.3	gene_id	MSTRG.27284;
4	StringTie	exon	80450832	80451676	.	+	.	transcript_	MSTRG.27284.3	gene_id	MSTRG.27284;
4	StringTie	transcrip	82794830	82800336	.	+	.	transcript_	MSTRG.27333.1	gene_id	MSTRG.27333;
4	StringTie	exon	82794830	82794952	.	+	.	transcript_	MSTRG.27333.1	gene_id	MSTRG.27333;
4	StringTie	exon	82799779	82800336	.	+	.	transcript_	MSTRG.27333.1	gene_id	MSTRG.27333;
4	StringTie	transcrip	87194274	87246764	.	+	.	transcript_	MSTRG.27437.1	gene_id	MSTRG.27437;
4	StringTie	exon	87194274	87194512	.	+	.	transcript_	MSTRG.27437.1	gene_id	MSTRG.27437;
4	StringTie	exon	87246424	87246764	.	+	.	transcript_	MSTRG.27437.1	gene_id	MSTRG.27437;
4	StringTie	transcrip	87722805	87756158	.	+	.	transcript_	MSTRG.27449.1	gene_id	MSTRG.27449;
4	StringTie	exon	87722805	87722819	.	+	.	transcript_	MSTRG.27449.1	gene_id	MSTRG.27449;
4	StringTie	exon	87751647	87756158	.	+	.	transcript_	MSTRG.27449.1	gene_id	MSTRG.27449;
4	StringTie	transcrip	88965860	88968544	.	+	.	transcript_	MSTRG.27473.1	gene_id	MSTRG.27473;
4	StringTie	exon	88965860	88966455	.	+	.	transcript_	MSTRG.27473.1	gene_id	MSTRG.27473;
4	StringTie	exon	88968472	88968544	.	+	.	transcript_	MSTRG.27473.1	gene_id	MSTRG.27473;
4	StringTie	transcrip	90721661	90773024	.	+	.	transcript_	MSTRG.27534.1	gene_id	MSTRG.27534;
4	StringTie	exon	90721661	90721723	.	+	.	transcript_	MSTRG.27534.1	gene_id	MSTRG.27534;
4	StringTie	exon	90722828	90722924	.	+	.	transcript_	MSTRG.27534.1	gene_id	MSTRG.27534;
4	StringTie	exon	90747558	90747687	.	+	.	transcript_	MSTRG.27534.1	gene_id	MSTRG.27534;
4	StringTie	exon	90772936	90773024	.	+	.	transcript_	MSTRG.27534.1	gene_id	MSTRG.27534;
4	StringTie	transcrip	91402638	91432984	.	+	.	transcript_	MSTRG.27569.3	gene_id	MSTRG.27569;
4	StringTie	exon	91402638	91402757	.	+	.	transcript_	MSTRG.27569.3	gene_id	MSTRG.27569;
4	StringTie	exon	91425086	91425495	.	+	.	transcript_	MSTRG.27569.3	gene_id	MSTRG.27569;
4	StringTie	exon	91432664	91432984	.	+	.	transcript_	MSTRG.27569.3	gene_id	MSTRG.27569;
4	StringTie	transcrip	91402640	91432986	.	+	.	transcript_	MSTRG.27569.5	gene_id	MSTRG.27569;
4	StringTie	exon	91402640	91402872	.	+	.	transcript_	MSTRG.27569.5	gene_id	MSTRG.27569;
4	StringTie	exon	91425086	91425472	.	+	.	transcript_	MSTRG.27569.5	gene_id	MSTRG.27569;

4	StringTie	exon	91432664	91432986	.	+	.	transcript_	MSTRG.27569.[illegible]	gene_id	MSTRG.27569;
4	StringTie	transcrip	91402641	91432984	.	+	.	transcript_	MSTRG.27569.[illegible]	gene_id	MSTRG.27569;
4	StringTie	exon	91402641	91402872	.	+	.	transcript_	MSTRG.27569.[illegible]	gene_id	MSTRG.27569;
4	StringTie	exon	91425086	91425495	.	+	.	transcript_	MSTRG.27569.[illegible]	gene_id	MSTRG.27569;
4	StringTie	exon	91432664	91432984	.	+	.	transcript_	MSTRG.27569.[illegible]	gene_id	MSTRG.27569;
4	StringTie	transcrip	92178145	92178376	.	+	.	transcript_	MSTRG.27561.[illegible]	gene_id	MSTRG.27561;
4	StringTie	exon	92178145	92178254	.	+	.	transcript_	MSTRG.27561.[illegible]	gene_id	MSTRG.27561;
4	StringTie	exon	92178277	92178376	.	+	.	transcript_	MSTRG.27561.[illegible]	gene_id	MSTRG.27561;
4	StringTie	transcrip	94243388	94262062	.	+	.	transcript_	MSTRG.27645.[illegible]	gene_id	MSTRG.27645;
4	StringTie	exon	94243388	94243415	.	+	.	transcript_	MSTRG.27645.[illegible]	gene_id	MSTRG.27645;
4	StringTie	exon	94260062	94262062	.	+	.	transcript_	MSTRG.27645.[illegible]	gene_id	MSTRG.27645;
4	StringTie	transcrip	94496982	94500288	.	+	.	transcript_	MSTRG.27656.[illegible]	gene_id	MSTRG.27656;
4	StringTie	exon	94496982	94497020	.	+	.	transcript_	MSTRG.27656.[illegible]	gene_id	MSTRG.27656;
4	StringTie	exon	94497657	94497772	.	+	.	transcript_	MSTRG.27656.[illegible]	gene_id	MSTRG.27656;
4	StringTie	exon	94500214	94500288	.	+	.	transcript_	MSTRG.27656.[illegible]	gene_id	MSTRG.27656;
4	StringTie	transcrip	94810916	94811715	.	+	.	transcript_	MSTRG.27679.[illegible]	gene_id	MSTRG.27679;
4	StringTie	exon	94810916	94811025	.	+	.	transcript_	MSTRG.27679.[illegible]	gene_id	MSTRG.27679;
4	StringTie	exon	94811522	94811715	.	+	.	transcript_	MSTRG.27679.[illegible]	gene_id	MSTRG.27679;
4	StringTie	transcrip	97319769	97322051	.	+	.	transcript_	MSTRG.27733.[illegible]	gene_id	MSTRG.27733;
4	StringTie	exon	97319769	97320150	.	+	.	transcript_	MSTRG.27733.[illegible]	gene_id	MSTRG.27733;
4	StringTie	exon	97321554	97322051	.	+	.	transcript_	MSTRG.27733.[illegible]	gene_id	MSTRG.27733;
4	StringTie	transcrip	97467400	97473561	.	+	.	transcript_	MSTRG.27749.[illegible]	gene_id	MSTRG.27749;
4	StringTie	exon	97467400	97467421	.	+	.	transcript_	MSTRG.27749.[illegible]	gene_id	MSTRG.27749;
4	StringTie	exon	97472225	97472410	.	+	.	transcript_	MSTRG.27749.[illegible]	gene_id	MSTRG.27749;
4	StringTie	exon	97473046	97473561	.	+	.	transcript_	MSTRG.27749.[illegible]	gene_id	MSTRG.27749;
4	StringTie	transcrip	98771826	98772612	.	+	.	transcript_	MSTRG.27813.[illegible]	gene_id	MSTRG.27813;
4	StringTie	exon	98771826	98772005	.	+	.	transcript_	MSTRG.27813.[illegible]	gene_id	MSTRG.27813;
4	StringTie	exon	98772543	98772612	.	+	.	transcript_	MSTRG.27813.[illegible]	gene_id	MSTRG.27813;
4	StringTie	transcrip	99157337	99160883	.	+	.	transcript_	MSTRG.27836.[illegible]	gene_id	MSTRG.27836;
4	StringTie	exon	99157337	99157544	.	+	.	transcript_	MSTRG.27836.[illegible]	gene_id	MSTRG.27836;
4	StringTie	exon	99160745	99160883	.	+	.	transcript_	MSTRG.27836.[illegible]	gene_id	MSTRG.27836;
4	StringTie	transcrip	99711671	99723961	.	+	.	transcript_	MSTRG.27880.[illegible]	gene_id	MSTRG.27880;
4	StringTie	exon	99711671	99712030	.	+	.	transcript_	MSTRG.27880.[illegible]	gene_id	MSTRG.27880;
4	StringTie	exon	99723854	99723961	.	+	.	transcript_	MSTRG.27880.[illegible]	gene_id	MSTRG.27880;
4	StringTie	transcrip	100442763	100450897	.	+	.	transcript_	MSTRG.27891.[illegible]	gene_id	MSTRG.27891;

4	StringTie	exon	100442763	100442839	.	+	.	transcript_	MSTRG.27891.2	gene_id	MSTRG.27891;
4	StringTie	exon	100446495	100446562	.	+	.	transcript_	MSTRG.27891.2	gene_id	MSTRG.27891;
4	StringTie	exon	100448730	100449083	.	+	.	transcript_	MSTRG.27891.2	gene_id	MSTRG.27891;
4	StringTie	exon	100450656	100450897	.	+	.	transcript_	MSTRG.27891.2	gene_id	MSTRG.27891;
4	StringTie	transcrip	100443753	100450871	.	+	.	transcript_	MSTRG.27891.3	gene_id	MSTRG.27891;
4	StringTie	exon	100443753	100443809	.	+	.	transcript_	MSTRG.27891.3	gene_id	MSTRG.27891;
4	StringTie	exon	100446495	100446562	.	+	.	transcript_	MSTRG.27891.3	gene_id	MSTRG.27891;
4	StringTie	exon	100448730	100449293	.	+	.	transcript_	MSTRG.27891.3	gene_id	MSTRG.27891;
4	StringTie	exon	100450681	100450871	.	+	.	transcript_	MSTRG.27891.3	gene_id	MSTRG.27891;
4	StringTie	transcrip	100906829	100933471	.	+	.	transcript_	MSTRG.27905.1	gene_id	MSTRG.27905;
4	StringTie	exon	100906829	100910678	.	+	.	transcript_	MSTRG.27905.1	gene_id	MSTRG.27905;
4	StringTie	exon	100932766	100933471	.	+	.	transcript_	MSTRG.27905.1	gene_id	MSTRG.27905;
4	StringTie	transcrip	110566229	110629231	.	+	.	transcript_	MSTRG.28190.1	gene_id	MSTRG.28190;
4	StringTie	exon	110566229	110566245	.	+	.	transcript_	MSTRG.28190.1	gene_id	MSTRG.28190;
4	StringTie	exon	110628538	110629231	.	+	.	transcript_	MSTRG.28190.1	gene_id	MSTRG.28190;
4	StringTie	transcrip	110658454	110661877	.	+	.	transcript_	MSTRG.28200.1	gene_id	MSTRG.28200;
4	StringTie	exon	110658454	110661758	.	+	.	transcript_	MSTRG.28200.1	gene_id	MSTRG.28200;
4	StringTie	exon	110661782	110661877	.	+	.	transcript_	MSTRG.28200.1	gene_id	MSTRG.28200;
4	StringTie	transcrip	110971589	110974519	.	+	.	transcript_	MSTRG.28206.1	gene_id	MSTRG.28206;
4	StringTie	exon	110971589	110971740	.	+	.	transcript_	MSTRG.28206.1	gene_id	MSTRG.28206;
4	StringTie	exon	110973988	110974519	.	+	.	transcript_	MSTRG.28206.1	gene_id	MSTRG.28206;
4	StringTie	transcrip	111627337	111629251	.	+	.	transcript_	MSTRG.28214.1	gene_id	MSTRG.28214;
4	StringTie	exon	111627337	111627837	.	+	.	transcript_	MSTRG.28214.1	gene_id	MSTRG.28214;
4	StringTie	exon	111628127	111629251	.	+	.	transcript_	MSTRG.28214.1	gene_id	MSTRG.28214;
4	StringTie	transcrip	117193027	117195167	.	+	.	transcript_	MSTRG.28270.1	gene_id	MSTRG.28270;
4	StringTie	exon	117193027	117193565	.	+	.	transcript_	MSTRG.28270.1	gene_id	MSTRG.28270;
4	StringTie	exon	117193889	117195167	.	+	.	transcript_	MSTRG.28270.1	gene_id	MSTRG.28270;
4	StringTie	transcrip	118090709	118092081	.	+	.	transcript_	MSTRG.28284.1	gene_id	MSTRG.28284;
4	StringTie	exon	118090709	118091008	.	+	.	transcript_	MSTRG.28284.1	gene_id	MSTRG.28284;
4	StringTie	exon	118091761	118092081	.	+	.	transcript_	MSTRG.28284.1	gene_id	MSTRG.28284;
4	StringTie	transcrip	118382879	118383174	.	+	.	transcript_	MSTRG.28310.1	gene_id	MSTRG.28310;
4	StringTie	exon	118382879	118382989	.	+	.	transcript_	MSTRG.28310.1	gene_id	MSTRG.28310;
4	StringTie	exon	118383064	118383174	.	+	.	transcript_	MSTRG.28310.1	gene_id	MSTRG.28310;
4	StringTie	transcrip	120737037	120764439	.	+	.	transcript_	MSTRG.28317.1	gene_id	MSTRG.28317;
4	StringTie	exon	120737037	120737063	.	+	.	transcript_	MSTRG.28317.1	gene_id	MSTRG.28317;

4	StringTie	exon	120764266	120764439	.	+	.	transcript_	MSTRG.28317.1	gene_id	MSTRG.28317;
4	StringTie	transcrip	123802453	123820199	.	+	.	transcript_	MSTRG.28384.1	gene_id	MSTRG.28384;
4	StringTie	exon	123802453	123803067	.	+	.	transcript_	MSTRG.28384.1	gene_id	MSTRG.28384;
4	StringTie	exon	123819361	123820199	.	+	.	transcript_	MSTRG.28384.1	gene_id	MSTRG.28384;
4	StringTie	transcrip	127042948	127045594	.	+	.	transcript_	MSTRG.28438.1	gene_id	MSTRG.28438;
4	StringTie	exon	127042948	127043002	.	+	.	transcript_	MSTRG.28438.1	gene_id	MSTRG.28438;
4	StringTie	exon	127043440	127045594	.	+	.	transcript_	MSTRG.28438.1	gene_id	MSTRG.28438;
4	StringTie	transcrip	153292	159099	.	-	.	transcript_	MSTRG.26044.1	gene_id	MSTRG.26044;
4	StringTie	exon	153292	153976	.	-	.	transcript_	MSTRG.26044.1	gene_id	MSTRG.26044;
4	StringTie	exon	157993	159099	.	-	.	transcript_	MSTRG.26044.1	gene_id	MSTRG.26044;
4	StringTie	transcrip	153301	159099	.	-	.	transcript_	MSTRG.26044.2	gene_id	MSTRG.26044;
4	StringTie	exon	153301	153976	.	-	.	transcript_	MSTRG.26044.2	gene_id	MSTRG.26044;
4	StringTie	exon	157993	158600	.	-	.	transcript_	MSTRG.26044.2	gene_id	MSTRG.26044;
4	StringTie	exon	158761	159099	.	-	.	transcript_	MSTRG.26044.2	gene_id	MSTRG.26044;
4	StringTie	transcrip	153361	159087	.	-	.	transcript_	MSTRG.26044.3	gene_id	MSTRG.26044;
4	StringTie	exon	153361	153976	.	-	.	transcript_	MSTRG.26044.3	gene_id	MSTRG.26044;
4	StringTie	exon	157993	158603	.	-	.	transcript_	MSTRG.26044.3	gene_id	MSTRG.26044;
4	StringTie	exon	158761	159087	.	-	.	transcript_	MSTRG.26044.3	gene_id	MSTRG.26044;
4	StringTie	transcrip	416362	418812	.	-	.	transcript_	MSTRG.26068.1	gene_id	MSTRG.26068;
4	StringTie	exon	416362	416578	.	-	.	transcript_	MSTRG.26068.1	gene_id	MSTRG.26068;
4	StringTie	exon	418541	418812	.	-	.	transcript_	MSTRG.26068.1	gene_id	MSTRG.26068;
4	StringTie	transcrip	747326	790164	.	-	.	transcript_	MSTRG.26126.1	gene_id	MSTRG.26126;
4	StringTie	exon	747326	747436	.	-	.	transcript_	MSTRG.26126.1	gene_id	MSTRG.26126;
4	StringTie	exon	790054	790164	.	-	.	transcript_	MSTRG.26126.1	gene_id	MSTRG.26126;
4	StringTie	transcrip	924566	925537	.	-	.	transcript_	MSTRG.26094.1	gene_id	MSTRG.26094;
4	StringTie	exon	924566	925537	.	-	.	transcript_	MSTRG.26094.1	gene_id	MSTRG.26094;
4	StringTie	transcrip	924719	925537	.	-	.	transcript_	MSTRG.26094.2	gene_id	MSTRG.26094;
4	StringTie	exon	924719	924872	.	-	.	transcript_	MSTRG.26094.2	gene_id	MSTRG.26094;
4	StringTie	exon	924909	925537	.	-	.	transcript_	MSTRG.26094.2	gene_id	MSTRG.26094;
4	StringTie	transcrip	2018144	2021201	.	-	.	transcript_	MSTRG.26151.1	gene_id	MSTRG.26151;
4	StringTie	exon	2018144	2018622	.	-	.	transcript_	MSTRG.26151.1	gene_id	MSTRG.26151;
4	StringTie	exon	2020863	2021201	.	-	.	transcript_	MSTRG.26151.1	gene_id	MSTRG.26151;
4	StringTie	transcrip	8475335	8507321	.	-	.	transcript_	MSTRG.26210.2	gene_id	MSTRG.26210;
4	StringTie	exon	8475335	8477437	.	-	.	transcript_	MSTRG.26210.2	gene_id	MSTRG.26210;
4	StringTie	exon	8485561	8485979	.	-	.	transcript_	MSTRG.26210.2	gene_id	MSTRG.26210;

4	StringTie	exon	8494066	8494246	.	-	.	transcript_	MSTRG.26210.2	gene_id	MSTRG.26210;
4	StringTie	exon	8507183	8507321	.	-	.	transcript_	MSTRG.26210.2	gene_id	MSTRG.26210;
4	StringTie	transcrip	8475335	8507321	.	-	.	transcript_	MSTRG.26210.1	gene_id	MSTRG.26210;
4	StringTie	exon	8475335	8477437	.	-	.	transcript_	MSTRG.26210.1	gene_id	MSTRG.26210;
4	StringTie	exon	8494066	8494246	.	-	.	transcript_	MSTRG.26210.1	gene_id	MSTRG.26210;
4	StringTie	exon	8507183	8507321	.	-	.	transcript_	MSTRG.26210.1	gene_id	MSTRG.26210;
4	StringTie	transcrip	8506148	8506893	.	-	.	transcript_	MSTRG.26210.4	gene_id	MSTRG.26210;
4	StringTie	exon	8506148	8506434	.	-	.	transcript_	MSTRG.26210.4	gene_id	MSTRG.26210;
4	StringTie	exon	8506628	8506893	.	-	.	transcript_	MSTRG.26210.4	gene_id	MSTRG.26210;
4	StringTie	transcrip	8506148	8507302	.	-	.	transcript_	MSTRG.26210.3	gene_id	MSTRG.26210;
4	StringTie	exon	8506148	8506434	.	-	.	transcript_	MSTRG.26210.3	gene_id	MSTRG.26210;
4	StringTie	exon	8507183	8507302	.	-	.	transcript_	MSTRG.26210.3	gene_id	MSTRG.26210;
4	StringTie	transcrip	14788625	14825648	.	-	.	transcript_	MSTRG.26232.1	gene_id	MSTRG.26232;
4	StringTie	exon	14788625	14789092	.	-	.	transcript_	MSTRG.26232.1	gene_id	MSTRG.26232;
4	StringTie	exon	14818698	14818889	.	-	.	transcript_	MSTRG.26232.1	gene_id	MSTRG.26232;
4	StringTie	exon	14825618	14825648	.	-	.	transcript_	MSTRG.26232.1	gene_id	MSTRG.26232;
4	StringTie	transcrip	15223157	15237666	.	-	.	transcript_	MSTRG.26295.1	gene_id	MSTRG.26295;
4	StringTie	exon	15223157	15223555	.	-	.	transcript_	MSTRG.26295.1	gene_id	MSTRG.26295;
4	StringTie	exon	15237572	15237666	.	-	.	transcript_	MSTRG.26295.1	gene_id	MSTRG.26295;
4	StringTie	transcrip	15800674	15800908	.	-	.	transcript_	MSTRG.26311.1	gene_id	MSTRG.26311;
4	StringTie	exon	15800674	15800782	.	-	.	transcript_	MSTRG.26311.1	gene_id	MSTRG.26311;
4	StringTie	exon	15800803	15800908	.	-	.	transcript_	MSTRG.26311.1	gene_id	MSTRG.26311;
4	StringTie	transcrip	16542352	16545650	.	-	.	transcript_	MSTRG.26337.1	gene_id	MSTRG.26337;
4	StringTie	exon	16542352	16544674	.	-	.	transcript_	MSTRG.26337.1	gene_id	MSTRG.26337;
4	StringTie	exon	16544696	16545650	.	-	.	transcript_	MSTRG.26337.1	gene_id	MSTRG.26337;
4	StringTie	transcrip	22530959	22539446	.	-	.	transcript_	MSTRG.26426.1	gene_id	MSTRG.26426;
4	StringTie	exon	22530959	22532255	.	-	.	transcript_	MSTRG.26426.1	gene_id	MSTRG.26426;
4	StringTie	exon	22539283	22539446	.	-	.	transcript_	MSTRG.26426.1	gene_id	MSTRG.26426;
4	StringTie	transcrip	28816403	28981234	.	-	.	transcript_	MSTRG.26499.1	gene_id	MSTRG.26499;
4	StringTie	exon	28816403	28816432	.	-	.	transcript_	MSTRG.26499.1	gene_id	MSTRG.26499;
4	StringTie	exon	28835461	28835646	.	-	.	transcript_	MSTRG.26499.1	gene_id	MSTRG.26499;
4	StringTie	exon	28981119	28981234	.	-	.	transcript_	MSTRG.26499.1	gene_id	MSTRG.26499;
4	StringTie	transcrip	34328227	34332473	.	-	.	transcript_	MSTRG.26588.1	gene_id	MSTRG.26588;
4	StringTie	exon	34328227	34329154	.	-	.	transcript_	MSTRG.26588.1	gene_id	MSTRG.26588;
4	StringTie	exon	34332246	34332473	.	-	.	transcript_	MSTRG.26588.1	gene_id	MSTRG.26588;

4	StringTie	transcrip	34328971	34350281	.	-	.	transcript_	MSTRG.26588.2	gene_id	MSTRG.26588;
4	StringTie	exon	34328971	34329154	.	-	.	transcript_	MSTRG.26588.2	gene_id	MSTRG.26588;
4	StringTie	exon	34350152	34350281	.	-	.	transcript_	MSTRG.26588.2	gene_id	MSTRG.26588;
4	StringTie	transcrip	41428411	41455712	.	-	.	transcript_	MSTRG.26709.1	gene_id	MSTRG.26709;
4	StringTie	exon	41428411	41428999	.	-	.	transcript_	MSTRG.26709.1	gene_id	MSTRG.26709;
4	StringTie	exon	41452195	41452436	.	-	.	transcript_	MSTRG.26709.1	gene_id	MSTRG.26709;
4	StringTie	exon	41455651	41455712	.	-	.	transcript_	MSTRG.26709.1	gene_id	MSTRG.26709;
4	StringTie	transcrip	42788999	42813869	.	-	.	transcript_	MSTRG.26743.1	gene_id	MSTRG.26743;
4	StringTie	exon	42788999	42789028	.	-	.	transcript_	MSTRG.26743.1	gene_id	MSTRG.26743;
4	StringTie	exon	42795487	42795962	.	-	.	transcript_	MSTRG.26743.1	gene_id	MSTRG.26743;
4	StringTie	exon	42813437	42813869	.	-	.	transcript_	MSTRG.26743.1	gene_id	MSTRG.26743;
4	StringTie	transcrip	53869552	53954299	.	-	.	transcript_	MSTRG.26862.1	gene_id	MSTRG.26862;
4	StringTie	exon	53869552	53869603	.	-	.	transcript_	MSTRG.26862.1	gene_id	MSTRG.26862;
4	StringTie	exon	53953473	53954299	.	-	.	transcript_	MSTRG.26862.1	gene_id	MSTRG.26862;
4	StringTie	transcrip	55107799	55168079	.	-	.	transcript_	MSTRG.26887.1	gene_id	MSTRG.26887;
4	StringTie	exon	55107799	55107909	.	-	.	transcript_	MSTRG.26887.1	gene_id	MSTRG.26887;
4	StringTie	exon	55167608	55168079	.	-	.	transcript_	MSTRG.26887.1	gene_id	MSTRG.26887;
4	StringTie	transcrip	55157046	55168079	.	-	.	transcript_	MSTRG.26887.2	gene_id	MSTRG.26887;
4	StringTie	exon	55157046	55158093	.	-	.	transcript_	MSTRG.26887.2	gene_id	MSTRG.26887;
4	StringTie	exon	55167608	55168079	.	-	.	transcript_	MSTRG.26887.2	gene_id	MSTRG.26887;
4	StringTie	transcrip	55228484	55230047	.	-	.	transcript_	MSTRG.26885.1	gene_id	MSTRG.26885;
4	StringTie	exon	55228484	55228866	.	-	.	transcript_	MSTRG.26885.1	gene_id	MSTRG.26885;
4	StringTie	exon	55229899	55230047	.	-	.	transcript_	MSTRG.26885.1	gene_id	MSTRG.26885;
4	StringTie	transcrip	58868973	58877968	.	-	.	transcript_	MSTRG.26923.6	gene_id	MSTRG.26923;
4	StringTie	exon	58868973	58870509	.	-	.	transcript_	MSTRG.26923.6	gene_id	MSTRG.26923;
4	StringTie	exon	58871141	58871179	.	-	.	transcript_	MSTRG.26923.6	gene_id	MSTRG.26923;
4	StringTie	exon	58877672	58877968	.	-	.	transcript_	MSTRG.26923.6	gene_id	MSTRG.26923;
4	StringTie	transcrip	62182422	62197652	.	-	.	transcript_	MSTRG.26961.1	gene_id	MSTRG.26961;
4	StringTie	exon	62182422	62182548	.	-	.	transcript_	MSTRG.26961.1	gene_id	MSTRG.26961;
4	StringTie	exon	62194786	62197652	.	-	.	transcript_	MSTRG.26961.1	gene_id	MSTRG.26961;
4	StringTie	transcrip	64367264	64429208	.	-	.	transcript_	MSTRG.26967.1	gene_id	MSTRG.26967;
4	StringTie	exon	64367264	64367321	.	-	.	transcript_	MSTRG.26967.1	gene_id	MSTRG.26967;
4	StringTie	exon	64428963	64429208	.	-	.	transcript_	MSTRG.26967.1	gene_id	MSTRG.26967;
4	StringTie	transcrip	68037755	68058951	.	-	.	transcript_	MSTRG.27020.1	gene_id	MSTRG.27020;
4	StringTie	exon	68037755	68038053	.	-	.	transcript_	MSTRG.27020.1	gene_id	MSTRG.27020;

4	StringTie	exon	68058338	68058951	.	-	.	transcript_	MSTRG.27020.1	gene_id	MSTRG.27020;
4	StringTie	transcrip	71615147	71618461	.	-	.	transcript_	MSTRG.27088.1	gene_id	MSTRG.27088;
4	StringTie	exon	71615147	71615367	.	-	.	transcript_	MSTRG.27088.1	gene_id	MSTRG.27088;
4	StringTie	exon	71616573	71616867	.	-	.	transcript_	MSTRG.27088.1	gene_id	MSTRG.27088;
4	StringTie	exon	71618295	71618461	.	-	.	transcript_	MSTRG.27088.1	gene_id	MSTRG.27088;
4	StringTie	transcrip	71615894	71618463	.	-	.	transcript_	MSTRG.27088.2	gene_id	MSTRG.27088;
4	StringTie	exon	71615894	71615982	.	-	.	transcript_	MSTRG.27088.2	gene_id	MSTRG.27088;
4	StringTie	exon	71616573	71616867	.	-	.	transcript_	MSTRG.27088.2	gene_id	MSTRG.27088;
4	StringTie	exon	71618295	71618463	.	-	.	transcript_	MSTRG.27088.2	gene_id	MSTRG.27088;
4	StringTie	transcrip	71621455	71624179	.	-	.	transcript_	MSTRG.27089.1	gene_id	MSTRG.27089;
4	StringTie	exon	71621455	71621566	.	-	.	transcript_	MSTRG.27089.1	gene_id	MSTRG.27089;
4	StringTie	exon	71622387	71622443	.	-	.	transcript_	MSTRG.27089.1	gene_id	MSTRG.27089;
4	StringTie	exon	71623111	71624179	.	-	.	transcript_	MSTRG.27089.1	gene_id	MSTRG.27089;
4	StringTie	transcrip	77592777	77594193	.	-	.	transcript_	MSTRG.27245.1	gene_id	MSTRG.27245;
4	StringTie	exon	77592777	77593290	.	-	.	transcript_	MSTRG.27245.1	gene_id	MSTRG.27245;
4	StringTie	exon	77593439	77593528	.	-	.	transcript_	MSTRG.27245.1	gene_id	MSTRG.27245;
4	StringTie	exon	77594100	77594193	.	-	.	transcript_	MSTRG.27245.1	gene_id	MSTRG.27245;
4	StringTie	transcrip	77592789	77594193	.	-	.	transcript_	MSTRG.27245.2	gene_id	MSTRG.27245;
4	StringTie	exon	77592789	77593528	.	-	.	transcript_	MSTRG.27245.2	gene_id	MSTRG.27245;
4	StringTie	exon	77594100	77594193	.	-	.	transcript_	MSTRG.27245.2	gene_id	MSTRG.27245;
4	StringTie	transcrip	79424347	79425055	.	-	.	transcript_	MSTRG.27248.1	gene_id	MSTRG.27248;
4	StringTie	exon	79424347	79424757	.	-	.	transcript_	MSTRG.27248.1	gene_id	MSTRG.27248;
4	StringTie	exon	79425011	79425055	.	-	.	transcript_	MSTRG.27248.1	gene_id	MSTRG.27248;
4	StringTie	transcrip	82304612	82308891	.	-	.	transcript_	MSTRG.27311.4	gene_id	MSTRG.27311;
4	StringTie	exon	82304612	82304749	.	-	.	transcript_	MSTRG.27311.4	gene_id	MSTRG.27311;
4	StringTie	exon	82307671	82308891	.	-	.	transcript_	MSTRG.27311.4	gene_id	MSTRG.27311;
4	StringTie	transcrip	82366322	82385965	.	-	.	transcript_	MSTRG.27311.5	gene_id	MSTRG.27311;
4	StringTie	exon	82366322	82366400	.	-	.	transcript_	MSTRG.27311.5	gene_id	MSTRG.27311;
4	StringTie	exon	82385836	82385965	.	-	.	transcript_	MSTRG.27311.5	gene_id	MSTRG.27311;
4	StringTie	transcrip	93418543	93418829	.	-	.	transcript_	MSTRG.27608.1	gene_id	MSTRG.27608;
4	StringTie	exon	93418543	93418652	.	-	.	transcript_	MSTRG.27608.1	gene_id	MSTRG.27608;
4	StringTie	exon	93418719	93418829	.	-	.	transcript_	MSTRG.27608.1	gene_id	MSTRG.27608;
4	StringTie	transcrip	94449212	94456637	.	-	.	transcript_	MSTRG.27649.1	gene_id	MSTRG.27649;
4	StringTie	exon	94449212	94449494	.	-	.	transcript_	MSTRG.27649.1	gene_id	MSTRG.27649;
4	StringTie	exon	94456589	94456637	.	-	.	transcript_	MSTRG.27649.1	gene_id	MSTRG.27649;

4	StringTie	transcrip	97292485	97319210	.	-	.	transcript_	MSTRG.27732.1	gene_id	MSTRG.27732;
4	StringTie	exon	97292485	97292608	.	-	.	transcript_	MSTRG.27732.1	gene_id	MSTRG.27732;
4	StringTie	exon	97317723	97317863	.	-	.	transcript_	MSTRG.27732.1	gene_id	MSTRG.27732;
4	StringTie	exon	97319183	97319210	.	-	.	transcript_	MSTRG.27732.1	gene_id	MSTRG.27732;
4	StringTie	transcrip	99342887	99352327	.	-	.	transcript_	MSTRG.27852.1	gene_id	MSTRG.27852;
4	StringTie	exon	99342887	99343074	.	-	.	transcript_	MSTRG.27852.1	gene_id	MSTRG.27852;
4	StringTie	exon	99352219	99352327	.	-	.	transcript_	MSTRG.27852.1	gene_id	MSTRG.27852;
4	StringTie	transcrip	99347030	99348303	.	-	.	transcript_	MSTRG.27854.1	gene_id	MSTRG.27854;
4	StringTie	exon	99347030	99347378	.	-	.	transcript_	MSTRG.27854.1	gene_id	MSTRG.27854;
4	StringTie	exon	99348219	99348303	.	-	.	transcript_	MSTRG.27854.1	gene_id	MSTRG.27854;
4	StringTie	transcrip	105066868	105075423	.	-	.	transcript_	MSTRG.28017.1	gene_id	MSTRG.28017;
4	StringTie	exon	105066868	105066956	.	-	.	transcript_	MSTRG.28017.1	gene_id	MSTRG.28017;
4	StringTie	exon	105075051	105075423	.	-	.	transcript_	MSTRG.28017.1	gene_id	MSTRG.28017;
4	StringTie	transcrip	105091592	105113363	.	-	.	transcript_	MSTRG.28020.1	gene_id	MSTRG.28020;
4	StringTie	exon	105091592	105091677	.	-	.	transcript_	MSTRG.28020.1	gene_id	MSTRG.28020;
4	StringTie	exon	105113111	105113363	.	-	.	transcript_	MSTRG.28020.1	gene_id	MSTRG.28020;
4	StringTie	transcrip	105109211	105113296	.	-	.	transcript_	MSTRG.28020.2	gene_id	MSTRG.28020;
4	StringTie	exon	105109211	105109342	.	-	.	transcript_	MSTRG.28020.2	gene_id	MSTRG.28020;
4	StringTie	exon	105113111	105113296	.	-	.	transcript_	MSTRG.28020.2	gene_id	MSTRG.28020;
4	StringTie	transcrip	108075466	108080305	.	-	.	transcript_	MSTRG.28095.1	gene_id	MSTRG.28095;
4	StringTie	exon	108075466	108075702	.	-	.	transcript_	MSTRG.28095.1	gene_id	MSTRG.28095;
4	StringTie	exon	108076073	108076318	.	-	.	transcript_	MSTRG.28095.1	gene_id	MSTRG.28095;
4	StringTie	exon	108079923	108080305	.	-	.	transcript_	MSTRG.28095.1	gene_id	MSTRG.28095;
4	StringTie	transcrip	108075548	108080147	.	-	.	transcript_	MSTRG.28095.2	gene_id	MSTRG.28095;
4	StringTie	exon	108075548	108075702	.	-	.	transcript_	MSTRG.28095.2	gene_id	MSTRG.28095;
4	StringTie	exon	108079923	108080147	.	-	.	transcript_	MSTRG.28095.2	gene_id	MSTRG.28095;
4	StringTie	transcrip	110676780	110692890	.	-	.	transcript_	MSTRG.28202.1	gene_id	MSTRG.28202;
4	StringTie	exon	110676780	110678632	.	-	.	transcript_	MSTRG.28202.1	gene_id	MSTRG.28202;
4	StringTie	exon	110686945	110687128	.	-	.	transcript_	MSTRG.28202.1	gene_id	MSTRG.28202;
4	StringTie	exon	110692716	110692890	.	-	.	transcript_	MSTRG.28202.1	gene_id	MSTRG.28202;
4	StringTie	transcrip	123074721	123085676	.	-	.	transcript_	MSTRG.28363.1	gene_id	MSTRG.28363;
4	StringTie	exon	123074721	123074903	.	-	.	transcript_	MSTRG.28363.1	gene_id	MSTRG.28363;
4	StringTie	exon	123085520	123085676	.	-	.	transcript_	MSTRG.28363.1	gene_id	MSTRG.28363;
4	StringTie	transcrip	127144146	127158094	.	-	.	transcript_	MSTRG.28469.1	gene_id	MSTRG.28469;
4	StringTie	exon	127144146	127144867	.	-	.	transcript_	MSTRG.28469.1	gene_id	MSTRG.28469;

4	StringTie	exon	127155686	127155871	.	-	.	transcript_	MSTRG.28469.1	gene_id	MSTRG.28469;
4	StringTie	exon	127157789	127158094	.	-	.	transcript_	MSTRG.28469.1	gene_id	MSTRG.28469;
4	StringTie	transcrip	127413001	127451384	.	-	.	transcript_	MSTRG.28484.4	gene_id	MSTRG.28484;
4	StringTie	exon	127413001	127414586	.	-	.	transcript_	MSTRG.28484.4	gene_id	MSTRG.28484;
4	StringTie	exon	127415381	127415598	.	-	.	transcript_	MSTRG.28484.4	gene_id	MSTRG.28484;
4	StringTie	exon	127444429	127444637	.	-	.	transcript_	MSTRG.28484.4	gene_id	MSTRG.28484;
4	StringTie	exon	127450284	127450344	.	-	.	transcript_	MSTRG.28484.4	gene_id	MSTRG.28484;
4	StringTie	exon	127451258	127451384	.	-	.	transcript_	MSTRG.28484.4	gene_id	MSTRG.28484;
4	StringTie	transcrip	130227319	130228836	.	-	.	transcript_	MSTRG.28505.1	gene_id	MSTRG.28505;
4	StringTie	exon	130227319	130228836	.	-	.	transcript_	MSTRG.28505.1	gene_id	MSTRG.28505;
4	StringTie	transcrip	130227343	130230291	.	-	.	transcript_	MSTRG.28505.2	gene_id	MSTRG.28505;
4	StringTie	exon	130227343	130227560	.	-	.	transcript_	MSTRG.28505.2	gene_id	MSTRG.28505;
4	StringTie	exon	130228781	130228833	.	-	.	transcript_	MSTRG.28505.2	gene_id	MSTRG.28505;
4	StringTie	exon	130230211	130230291	.	-	.	transcript_	MSTRG.28505.2	gene_id	MSTRG.28505;
4	StringTie	transcrip	130663283	130665611	.	-	.	transcript_	MSTRG.28524.1	gene_id	MSTRG.28524;
4	StringTie	exon	130663283	130663330	.	-	.	transcript_	MSTRG.28524.1	gene_id	MSTRG.28524;
4	StringTie	exon	130665247	130665611	.	-	.	transcript_	MSTRG.28524.1	gene_id	MSTRG.28524;
4	StringTie	transcrip	130689981	130700693	.	-	.	transcript_	MSTRG.28526.1	gene_id	MSTRG.28526;
4	StringTie	exon	130689981	130690068	.	-	.	transcript_	MSTRG.28526.1	gene_id	MSTRG.28526;
4	StringTie	exon	130700508	130700693	.	-	.	transcript_	MSTRG.28526.1	gene_id	MSTRG.28526;
4	StringTie	transcrip	130695135	130703041	.	-	.	transcript_	MSTRG.28527.1	gene_id	MSTRG.28527;
4	StringTie	exon	130695135	130697911	.	-	.	transcript_	MSTRG.28527.1	gene_id	MSTRG.28527;
4	StringTie	exon	130702966	130703041	.	-	.	transcript_	MSTRG.28527.1	gene_id	MSTRG.28527;
5	StringTie	transcrip	689159	694405	.	+	.	transcript_	MSTRG.28551.1	gene_id	MSTRG.28551;
5	StringTie	exon	689159	689389	.	+	.	transcript_	MSTRG.28551.1	gene_id	MSTRG.28551;
5	StringTie	exon	693140	693236	.	+	.	transcript_	MSTRG.28551.1	gene_id	MSTRG.28551;
5	StringTie	exon	694325	694405	.	+	.	transcript_	MSTRG.28551.1	gene_id	MSTRG.28551;
5	StringTie	transcrip	3294852	3301663	.	+	.	transcript_	MSTRG.28602.1	gene_id	MSTRG.28602;
5	StringTie	exon	3294852	3296098	.	+	.	transcript_	MSTRG.28602.1	gene_id	MSTRG.28602;
5	StringTie	exon	3296120	3301663	.	+	.	transcript_	MSTRG.28602.1	gene_id	MSTRG.28602;
5	StringTie	transcrip	3460380	3462280	.	+	.	transcript_	MSTRG.28594.1	gene_id	MSTRG.28594;
5	StringTie	exon	3460380	3461768	.	+	.	transcript_	MSTRG.28594.1	gene_id	MSTRG.28594;
5	StringTie	exon	3461979	3462280	.	+	.	transcript_	MSTRG.28594.1	gene_id	MSTRG.28594;
5	StringTie	transcrip	3460457	3462284	.	+	.	transcript_	MSTRG.28594.2	gene_id	MSTRG.28594;
5	StringTie	exon	3460457	3462284	.	+	.	transcript_	MSTRG.28594.2	gene_id	MSTRG.28594;

5	StringTie	transcrip	5074101	5077189	.	+	.	transcript_	MSTRG.28630.1	gene_id	MSTRG.28630;
5	StringTie	exon	5074101	5074203	.	+	.	transcript_	MSTRG.28630.1	gene_id	MSTRG.28630;
5	StringTie	exon	5076957	5077189	.	+	.	transcript_	MSTRG.28630.1	gene_id	MSTRG.28630;
5	StringTie	transcrip	6346531	6351275	.	+	.	transcript_	MSTRG.28659.3	gene_id	MSTRG.28659;
5	StringTie	exon	6346531	6346670	.	+	.	transcript_	MSTRG.28659.3	gene_id	MSTRG.28659;
5	StringTie	exon	6350913	6351275	.	+	.	transcript_	MSTRG.28659.3	gene_id	MSTRG.28659;
5	StringTie	transcrip	7110601	7117267	.	+	.	transcript_	MSTRG.28680.1	gene_id	MSTRG.28680;
5	StringTie	exon	7110601	7110644	.	+	.	transcript_	MSTRG.28680.1	gene_id	MSTRG.28680;
5	StringTie	exon	7111768	7111841	.	+	.	transcript_	MSTRG.28680.1	gene_id	MSTRG.28680;
5	StringTie	exon	7117058	7117267	.	+	.	transcript_	MSTRG.28680.1	gene_id	MSTRG.28680;
5	StringTie	transcrip	8046031	8049870	.	+	.	transcript_	MSTRG.28703.1	gene_id	MSTRG.28703;
5	StringTie	exon	8046031	8046380	.	+	.	transcript_	MSTRG.28703.1	gene_id	MSTRG.28703;
5	StringTie	exon	8049144	8049870	.	+	.	transcript_	MSTRG.28703.1	gene_id	MSTRG.28703;
5	StringTie	transcrip	9141884	9169820	.	+	.	transcript_	MSTRG.28734.1	gene_id	MSTRG.28734;
5	StringTie	exon	9141884	9142115	.	+	.	transcript_	MSTRG.28734.1	gene_id	MSTRG.28734;
5	StringTie	exon	9148431	9148512	.	+	.	transcript_	MSTRG.28734.1	gene_id	MSTRG.28734;
5	StringTie	exon	9169498	9169820	.	+	.	transcript_	MSTRG.28734.1	gene_id	MSTRG.28734;
5	StringTie	transcrip	9815734	9823341	.	+	.	transcript_	MSTRG.28772.1	gene_id	MSTRG.28772;
5	StringTie	exon	9815734	9816100	.	+	.	transcript_	MSTRG.28772.1	gene_id	MSTRG.28772;
5	StringTie	exon	9818659	9818746	.	+	.	transcript_	MSTRG.28772.1	gene_id	MSTRG.28772;
5	StringTie	exon	9820254	9823341	.	+	.	transcript_	MSTRG.28772.1	gene_id	MSTRG.28772;
5	StringTie	transcrip	9815768	9822149	.	+	.	transcript_	MSTRG.28772.2	gene_id	MSTRG.28772;
5	StringTie	exon	9815768	9815962	.	+	.	transcript_	MSTRG.28772.2	gene_id	MSTRG.28772;
5	StringTie	exon	9820254	9822149	.	+	.	transcript_	MSTRG.28772.2	gene_id	MSTRG.28772;
5	StringTie	transcrip	18397815	18403548	.	+	.	transcript_	MSTRG.29006.2	gene_id	MSTRG.29006;
5	StringTie	exon	18397815	18398644	.	+	.	transcript_	MSTRG.29006.2	gene_id	MSTRG.29006;
5	StringTie	exon	18400286	18403548	.	+	.	transcript_	MSTRG.29006.2	gene_id	MSTRG.29006;
5	StringTie	transcrip	19104831	19111847	.	+	.	transcript_	MSTRG.29036.1	gene_id	MSTRG.29036;
5	StringTie	exon	19104831	19104903	.	+	.	transcript_	MSTRG.29036.1	gene_id	MSTRG.29036;
5	StringTie	exon	19111678	19111847	.	+	.	transcript_	MSTRG.29036.1	gene_id	MSTRG.29036;
5	StringTie	transcrip	19344837	19350116	.	+	.	transcript_	MSTRG.29049.1	gene_id	MSTRG.29049;
5	StringTie	exon	19344837	19344975	.	+	.	transcript_	MSTRG.29049.1	gene_id	MSTRG.29049;
5	StringTie	exon	19350038	19350116	.	+	.	transcript_	MSTRG.29049.1	gene_id	MSTRG.29049;
5	StringTie	transcrip	20208617	20213203	.	+	.	transcript_	MSTRG.29066.1	gene_id	MSTRG.29066;
5	StringTie	exon	20208617	20208734	.	+	.	transcript_	MSTRG.29066.1	gene_id	MSTRG.29066;

5	StringTie	exon	20212826	20213203	.	+	.	transcript_	MSTRG.29066.1	gene_id	MSTRG.29066;
5	StringTie	transcrip	21180809	21186161	.	+	.	transcript_	MSTRG.29074.1	gene_id	MSTRG.29074;
5	StringTie	exon	21180809	21181160	.	+	.	transcript_	MSTRG.29074.1	gene_id	MSTRG.29074;
5	StringTie	exon	21186091	21186161	.	+	.	transcript_	MSTRG.29074.1	gene_id	MSTRG.29074;
5	StringTie	transcrip	21987244	21989244	.	+	.	transcript_	MSTRG.29150.1	gene_id	MSTRG.29150;
5	StringTie	exon	21987244	21987353	.	+	.	transcript_	MSTRG.29150.1	gene_id	MSTRG.29150;
5	StringTie	exon	21989134	21989244	.	+	.	transcript_	MSTRG.29150.1	gene_id	MSTRG.29150;
5	StringTie	transcrip	22252178	22272031	.	+	.	transcript_	MSTRG.29167.1	gene_id	MSTRG.29167;
5	StringTie	exon	22252178	22252286	.	+	.	transcript_	MSTRG.29167.1	gene_id	MSTRG.29167;
5	StringTie	exon	22271848	22272031	.	+	.	transcript_	MSTRG.29167.1	gene_id	MSTRG.29167;
5	StringTie	transcrip	22257623	22266951	.	+	.	transcript_	MSTRG.29168.1	gene_id	MSTRG.29168;
5	StringTie	exon	22257623	22257733	.	+	.	transcript_	MSTRG.29168.1	gene_id	MSTRG.29168;
5	StringTie	exon	22266787	22266951	.	+	.	transcript_	MSTRG.29168.1	gene_id	MSTRG.29168;
5	StringTie	transcrip	22262184	22278560	.	+	.	transcript_	MSTRG.29169.1	gene_id	MSTRG.29169;
5	StringTie	exon	22262184	22262288	.	+	.	transcript_	MSTRG.29169.1	gene_id	MSTRG.29169;
5	StringTie	exon	22278450	22278560	.	+	.	transcript_	MSTRG.29169.1	gene_id	MSTRG.29169;
5	StringTie	transcrip	39309451	39331254	.	+	.	transcript_	MSTRG.29500.1	gene_id	MSTRG.29500;
5	StringTie	exon	39309451	39312037	.	+	.	transcript_	MSTRG.29500.1	gene_id	MSTRG.29500;
5	StringTie	exon	39331160	39331254	.	+	.	transcript_	MSTRG.29500.1	gene_id	MSTRG.29500;
5	StringTie	transcrip	40424454	40426524	.	+	.	transcript_	MSTRG.29504.1	gene_id	MSTRG.29504;
5	StringTie	exon	40424454	40424680	.	+	.	transcript_	MSTRG.29504.1	gene_id	MSTRG.29504;
5	StringTie	exon	40426488	40426524	.	+	.	transcript_	MSTRG.29504.1	gene_id	MSTRG.29504;
5	StringTie	transcrip	42299877	42313915	.	+	.	transcript_	MSTRG.29540.1	gene_id	MSTRG.29540;
5	StringTie	exon	42299877	42299923	.	+	.	transcript_	MSTRG.29540.1	gene_id	MSTRG.29540;
5	StringTie	exon	42313674	42313915	.	+	.	transcript_	MSTRG.29540.1	gene_id	MSTRG.29540;
5	StringTie	transcrip	42308971	42313997	.	+	.	transcript_	MSTRG.29540.2	gene_id	MSTRG.29540;
5	StringTie	exon	42308971	42309037	.	+	.	transcript_	MSTRG.29540.2	gene_id	MSTRG.29540;
5	StringTie	exon	42313674	42313997	.	+	.	transcript_	MSTRG.29540.2	gene_id	MSTRG.29540;
5	StringTie	transcrip	42485479	42488828	.	+	.	transcript_	MSTRG.29535.1	gene_id	MSTRG.29535;
5	StringTie	exon	42485479	42485499	.	+	.	transcript_	MSTRG.29535.1	gene_id	MSTRG.29535;
5	StringTie	exon	42488534	42488828	.	+	.	transcript_	MSTRG.29535.1	gene_id	MSTRG.29535;
5	StringTie	transcrip	46023152	46024840	.	+	.	transcript_	MSTRG.29574.1	gene_id	MSTRG.29574;
5	StringTie	exon	46023152	46023281	.	+	.	transcript_	MSTRG.29574.1	gene_id	MSTRG.29574;
5	StringTie	exon	46024273	46024840	.	+	.	transcript_	MSTRG.29574.1	gene_id	MSTRG.29574;
5	StringTie	transcrip	46323950	46326714	.	+	.	transcript_	MSTRG.29585.1	gene_id	MSTRG.29585;

5	StringTie	exon	46323950	46325406	.	+	.	transcript_	MSTRG.29585.1	gene_id	MSTRG.29585;
5	StringTie	exon	46326278	46326714	.	+	.	transcript_	MSTRG.29585.1	gene_id	MSTRG.29585;
5	StringTie	transcrip	46362446	46401065	.	+	.	transcript_	MSTRG.29591.1	gene_id	MSTRG.29591;
5	StringTie	exon	46362446	46362762	.	+	.	transcript_	MSTRG.29591.1	gene_id	MSTRG.29591;
5	StringTie	exon	46398252	46401065	.	+	.	transcript_	MSTRG.29591.1	gene_id	MSTRG.29591;
5	StringTie	transcrip	49482544	49531726	.	+	.	transcript_	MSTRG.29702.2	gene_id	MSTRG.29702;
5	StringTie	exon	49482544	49482626	.	+	.	transcript_	MSTRG.29702.2	gene_id	MSTRG.29702;
5	StringTie	exon	49504551	49504611	.	+	.	transcript_	MSTRG.29702.2	gene_id	MSTRG.29702;
5	StringTie	exon	49531506	49531726	.	+	.	transcript_	MSTRG.29702.2	gene_id	MSTRG.29702;
5	StringTie	transcrip	49969118	50005799	.	+	.	transcript_	MSTRG.29711.1	gene_id	MSTRG.29711;
5	StringTie	exon	49969118	49969142	.	+	.	transcript_	MSTRG.29711.1	gene_id	MSTRG.29711;
5	StringTie	exon	50001004	50005799	.	+	.	transcript_	MSTRG.29711.1	gene_id	MSTRG.29711;
5	StringTie	transcrip	54758768	54763183	.	+	.	transcript_	MSTRG.29811.1	gene_id	MSTRG.29811;
5	StringTie	exon	54758768	54760303	.	+	.	transcript_	MSTRG.29811.1	gene_id	MSTRG.29811;
5	StringTie	exon	54762885	54763183	.	+	.	transcript_	MSTRG.29811.1	gene_id	MSTRG.29811;
5	StringTie	transcrip	57390395	57393014	.	+	.	transcript_	MSTRG.29833.1	gene_id	MSTRG.29833;
5	StringTie	exon	57390395	57390433	.	+	.	transcript_	MSTRG.29833.1	gene_id	MSTRG.29833;
5	StringTie	exon	57392852	57393014	.	+	.	transcript_	MSTRG.29833.1	gene_id	MSTRG.29833;
5	StringTie	transcrip	62023548	62025200	.	+	.	transcript_	MSTRG.29924.4	gene_id	MSTRG.29924;
5	StringTie	exon	62023548	62023981	.	+	.	transcript_	MSTRG.29924.4	gene_id	MSTRG.29924;
5	StringTie	exon	62024548	62025200	.	+	.	transcript_	MSTRG.29924.4	gene_id	MSTRG.29924;
5	StringTie	transcrip	64135109	64141527	.	+	.	transcript_	MSTRG.30008.1	gene_id	MSTRG.30008;
5	StringTie	exon	64135109	64135207	.	+	.	transcript_	MSTRG.30008.1	gene_id	MSTRG.30008;
5	StringTie	exon	64140729	64140861	.	+	.	transcript_	MSTRG.30008.1	gene_id	MSTRG.30008;
5	StringTie	exon	64141406	64141527	.	+	.	transcript_	MSTRG.30008.1	gene_id	MSTRG.30008;
5	StringTie	transcrip	64378769	64420505	.	+	.	transcript_	MSTRG.30025.1	gene_id	MSTRG.30025;
5	StringTie	exon	64378769	64378925	.	+	.	transcript_	MSTRG.30025.1	gene_id	MSTRG.30025;
5	StringTie	exon	64394687	64394861	.	+	.	transcript_	MSTRG.30025.1	gene_id	MSTRG.30025;
5	StringTie	exon	64400043	64400124	.	+	.	transcript_	MSTRG.30025.1	gene_id	MSTRG.30025;
5	StringTie	exon	64420488	64420505	.	+	.	transcript_	MSTRG.30025.1	gene_id	MSTRG.30025;
5	StringTie	transcrip	65160914	65171891	.	+	.	transcript_	MSTRG.30031.1	gene_id	MSTRG.30031;
5	StringTie	exon	65160914	65161053	.	+	.	transcript_	MSTRG.30031.1	gene_id	MSTRG.30031;
5	StringTie	exon	65171419	65171891	.	+	.	transcript_	MSTRG.30031.1	gene_id	MSTRG.30031;
5	StringTie	transcrip	69554496	69555451	.	+	.	transcript_	MSTRG.30128.2	gene_id	MSTRG.30128;
5	StringTie	exon	69554496	69554567	.	+	.	transcript_	MSTRG.30128.2	gene_id	MSTRG.30128;

5	StringTie	exon	69554903	69555451	.	+	.	transcript_	MSTRG.30128.2	gene_id	MSTRG.30128;
5	StringTie	transcrip	74881408	74884901	.	+	.	transcript_	MSTRG.30200.1	gene_id	MSTRG.30200;
5	StringTie	exon	74881408	74881907	.	+	.	transcript_	MSTRG.30200.1	gene_id	MSTRG.30200;
5	StringTie	exon	74882102	74882226	.	+	.	transcript_	MSTRG.30200.1	gene_id	MSTRG.30200;
5	StringTie	exon	74884510	74884901	.	+	.	transcript_	MSTRG.30200.1	gene_id	MSTRG.30200;
5	StringTie	transcrip	77005418	77013089	.	+	.	transcript_	MSTRG.30226.1	gene_id	MSTRG.30226;
5	StringTie	exon	77005418	77011264	.	+	.	transcript_	MSTRG.30226.1	gene_id	MSTRG.30226;
5	StringTie	exon	77011944	77012067	.	+	.	transcript_	MSTRG.30226.1	gene_id	MSTRG.30226;
5	StringTie	exon	77013018	77013089	.	+	.	transcript_	MSTRG.30226.1	gene_id	MSTRG.30226;
5	StringTie	transcrip	77009888	77012015	.	+	.	transcript_	MSTRG.30226.2	gene_id	MSTRG.30226;
5	StringTie	exon	77009888	77010031	.	+	.	transcript_	MSTRG.30226.2	gene_id	MSTRG.30226;
5	StringTie	exon	77011113	77011264	.	+	.	transcript_	MSTRG.30226.2	gene_id	MSTRG.30226;
5	StringTie	exon	77011944	77012015	.	+	.	transcript_	MSTRG.30226.2	gene_id	MSTRG.30226;
5	StringTie	transcrip	78267150	78288428	.	+	.	transcript_	MSTRG.30265.3	gene_id	MSTRG.30265;
5	StringTie	exon	78267150	78267254	.	+	.	transcript_	MSTRG.30265.3	gene_id	MSTRG.30265;
5	StringTie	exon	78268953	78269061	.	+	.	transcript_	MSTRG.30265.3	gene_id	MSTRG.30265;
5	StringTie	exon	78288166	78288428	.	+	.	transcript_	MSTRG.30265.3	gene_id	MSTRG.30265;
5	StringTie	transcrip	82554075	82555514	.	+	.	transcript_	MSTRG.30373.1	gene_id	MSTRG.30373;
5	StringTie	exon	82554075	82554151	.	+	.	transcript_	MSTRG.30373.1	gene_id	MSTRG.30373;
5	StringTie	exon	82555052	82555514	.	+	.	transcript_	MSTRG.30373.1	gene_id	MSTRG.30373;
5	StringTie	transcrip	85272530	85273301	.	+	.	transcript_	MSTRG.30407.1	gene_id	MSTRG.30407;
5	StringTie	exon	85272530	85273114	.	+	.	transcript_	MSTRG.30407.1	gene_id	MSTRG.30407;
5	StringTie	exon	85273136	85273301	.	+	.	transcript_	MSTRG.30407.1	gene_id	MSTRG.30407;
5	StringTie	transcrip	89479500	89490328	.	+	.	transcript_	MSTRG.30522.1	gene_id	MSTRG.30522;
5	StringTie	exon	89479500	89479672	.	+	.	transcript_	MSTRG.30522.1	gene_id	MSTRG.30522;
5	StringTie	exon	89489649	89490328	.	+	.	transcript_	MSTRG.30522.1	gene_id	MSTRG.30522;
5	StringTie	transcrip	89728488	89954515	.	+	.	transcript_	MSTRG.30536.1	gene_id	MSTRG.30536;
5	StringTie	exon	89728488	89728697	.	+	.	transcript_	MSTRG.30536.1	gene_id	MSTRG.30536;
5	StringTie	exon	89746204	89746331	.	+	.	transcript_	MSTRG.30536.1	gene_id	MSTRG.30536;
5	StringTie	exon	89884161	89884309	.	+	.	transcript_	MSTRG.30536.1	gene_id	MSTRG.30536;
5	StringTie	exon	89954285	89954515	.	+	.	transcript_	MSTRG.30536.1	gene_id	MSTRG.30536;
5	StringTie	transcrip	89728567	90053329	.	+	.	transcript_	MSTRG.30536.2	gene_id	MSTRG.30536;
5	StringTie	exon	89728567	89728697	.	+	.	transcript_	MSTRG.30536.2	gene_id	MSTRG.30536;
5	StringTie	exon	89746204	89746331	.	+	.	transcript_	MSTRG.30536.2	gene_id	MSTRG.30536;
5	StringTie	exon	89884161	89884309	.	+	.	transcript_	MSTRG.30536.2	gene_id	MSTRG.30536;

5	StringTie	exon	90053224	90053329	.	+	.	transcript_	MSTRG.30536.2	gene_id	MSTRG.30536;
5	StringTie	transcrip	91038633	91116091	.	+	.	transcript_	MSTRG.30571.1	gene_id	MSTRG.30571;
5	StringTie	exon	91038633	91038693	.	+	.	transcript_	MSTRG.30571.1	gene_id	MSTRG.30571;
5	StringTie	exon	91068924	91069059	.	+	.	transcript_	MSTRG.30571.1	gene_id	MSTRG.30571;
5	StringTie	exon	91094027	91094086	.	+	.	transcript_	MSTRG.30571.1	gene_id	MSTRG.30571;
5	StringTie	exon	91094706	91094886	.	+	.	transcript_	MSTRG.30571.1	gene_id	MSTRG.30571;
5	StringTie	exon	91113511	91116091	.	+	.	transcript_	MSTRG.30571.1	gene_id	MSTRG.30571;
5	StringTie	transcrip	91081632	91114796	.	+	.	transcript_	MSTRG.30571.2	gene_id	MSTRG.30571;
5	StringTie	exon	91081632	91081722	.	+	.	transcript_	MSTRG.30571.2	gene_id	MSTRG.30571;
5	StringTie	exon	91083146	91083284	.	+	.	transcript_	MSTRG.30571.2	gene_id	MSTRG.30571;
5	StringTie	exon	91094027	91094086	.	+	.	transcript_	MSTRG.30571.2	gene_id	MSTRG.30571;
5	StringTie	exon	91094706	91094886	.	+	.	transcript_	MSTRG.30571.2	gene_id	MSTRG.30571;
5	StringTie	exon	91113511	91114796	.	+	.	transcript_	MSTRG.30571.2	gene_id	MSTRG.30571;
5	StringTie	transcrip	91094019	91115078	.	+	.	transcript_	MSTRG.30571.3	gene_id	MSTRG.30571;
5	StringTie	exon	91094019	91094086	.	+	.	transcript_	MSTRG.30571.3	gene_id	MSTRG.30571;
5	StringTie	exon	91094706	91094886	.	+	.	transcript_	MSTRG.30571.3	gene_id	MSTRG.30571;
5	StringTie	exon	91113511	91113691	.	+	.	transcript_	MSTRG.30571.3	gene_id	MSTRG.30571;
5	StringTie	exon	91113783	91115078	.	+	.	transcript_	MSTRG.30571.3	gene_id	MSTRG.30571;
5	StringTie	transcrip	92812567	92900121	.	+	.	transcript_	MSTRG.30589.1	gene_id	MSTRG.30589;
5	StringTie	exon	92812567	92812761	.	+	.	transcript_	MSTRG.30589.1	gene_id	MSTRG.30589;
5	StringTie	exon	92838094	92838177	.	+	.	transcript_	MSTRG.30589.1	gene_id	MSTRG.30589;
5	StringTie	exon	92892698	92892775	.	+	.	transcript_	MSTRG.30589.1	gene_id	MSTRG.30589;
5	StringTie	exon	92900074	92900121	.	+	.	transcript_	MSTRG.30589.1	gene_id	MSTRG.30589;
5	StringTie	transcrip	94297605	94328234	.	+	.	transcript_	MSTRG.30640.1	gene_id	MSTRG.30640;
5	StringTie	exon	94297605	94297636	.	+	.	transcript_	MSTRG.30640.1	gene_id	MSTRG.30640;
5	StringTie	exon	94327314	94328234	.	+	.	transcript_	MSTRG.30640.1	gene_id	MSTRG.30640;
5	StringTie	transcrip	103351195	103358162	.	+	.	transcript_	MSTRG.30688.1	gene_id	MSTRG.30688;
5	StringTie	exon	103351195	103351249	.	+	.	transcript_	MSTRG.30688.1	gene_id	MSTRG.30688;
5	StringTie	exon	103355341	103355485	.	+	.	transcript_	MSTRG.30688.1	gene_id	MSTRG.30688;
5	StringTie	exon	103356449	103356880	.	+	.	transcript_	MSTRG.30688.1	gene_id	MSTRG.30688;
5	StringTie	exon	103358074	103358162	.	+	.	transcript_	MSTRG.30688.1	gene_id	MSTRG.30688;
5	StringTie	transcrip	104379202	104404036	.	+	.	transcript_	MSTRG.30695.1	gene_id	MSTRG.30695;
5	StringTie	exon	104379202	104379402	.	+	.	transcript_	MSTRG.30695.1	gene_id	MSTRG.30695;
5	StringTie	exon	104403982	104404036	.	+	.	transcript_	MSTRG.30695.1	gene_id	MSTRG.30695;
5	StringTie	transcrip	91248	92654	.	-	.	transcript_	MSTRG.28536.2	gene_id	MSTRG.28536;

5	StringTie	exon	91248	91582	.	-	.	transcript_	MSTRG.28536.2	gene_id	MSTRG.28536;
5	StringTie	exon	92520	92654	.	-	.	transcript_	MSTRG.28536.2	gene_id	MSTRG.28536;
5	StringTie	transcrip	4906700	4907825	.	-	.	transcript_	MSTRG.28622.1	gene_id	MSTRG.28622;
5	StringTie	exon	4906700	4907034	.	-	.	transcript_	MSTRG.28622.1	gene_id	MSTRG.28622;
5	StringTie	exon	4907625	4907825	.	-	.	transcript_	MSTRG.28622.1	gene_id	MSTRG.28622;
5	StringTie	transcrip	6283479	6290544	.	-	.	transcript_	MSTRG.28656.1	gene_id	MSTRG.28656;
5	StringTie	exon	6283479	6284154	.	-	.	transcript_	MSTRG.28656.1	gene_id	MSTRG.28656;
5	StringTie	exon	6286488	6286634	.	-	.	transcript_	MSTRG.28656.1	gene_id	MSTRG.28656;
5	StringTie	exon	6290101	6290544	.	-	.	transcript_	MSTRG.28656.1	gene_id	MSTRG.28656;
5	StringTie	transcrip	8971953	8976026	.	-	.	transcript_	MSTRG.28724.1	gene_id	MSTRG.28724;
5	StringTie	exon	8971953	8972147	.	-	.	transcript_	MSTRG.28724.1	gene_id	MSTRG.28724;
5	StringTie	exon	8975770	8976026	.	-	.	transcript_	MSTRG.28724.1	gene_id	MSTRG.28724;
5	StringTie	transcrip	9419336	9485571	.	-	.	transcript_	MSTRG.28762.1	gene_id	MSTRG.28762;
5	StringTie	exon	9419336	9419517	.	-	.	transcript_	MSTRG.28762.1	gene_id	MSTRG.28762;
5	StringTie	exon	9485502	9485571	.	-	.	transcript_	MSTRG.28762.1	gene_id	MSTRG.28762;
5	StringTie	transcrip	11908534	11913292	.	-	.	transcript_	MSTRG.28837.1	gene_id	MSTRG.28837;
5	StringTie	exon	11908534	11911565	.	-	.	transcript_	MSTRG.28837.1	gene_id	MSTRG.28837;
5	StringTie	exon	11912672	11913292	.	-	.	transcript_	MSTRG.28837.1	gene_id	MSTRG.28837;
5	StringTie	transcrip	14030040	14068160	.	-	.	transcript_	MSTRG.28874.1	gene_id	MSTRG.28874;
5	StringTie	exon	14030040	14031067	.	-	.	transcript_	MSTRG.28874.1	gene_id	MSTRG.28874;
5	StringTie	exon	14056824	14057044	.	-	.	transcript_	MSTRG.28874.1	gene_id	MSTRG.28874;
5	StringTie	exon	14059391	14059530	.	-	.	transcript_	MSTRG.28874.1	gene_id	MSTRG.28874;
5	StringTie	exon	14061205	14062357	.	-	.	transcript_	MSTRG.28874.1	gene_id	MSTRG.28874;
5	StringTie	exon	14067842	14068160	.	-	.	transcript_	MSTRG.28874.1	gene_id	MSTRG.28874;
5	StringTie	transcrip	14030047	14067882	.	-	.	transcript_	MSTRG.28874.2	gene_id	MSTRG.28874;
5	StringTie	exon	14030047	14031067	.	-	.	transcript_	MSTRG.28874.2	gene_id	MSTRG.28874;
5	StringTie	exon	14056824	14057044	.	-	.	transcript_	MSTRG.28874.2	gene_id	MSTRG.28874;
5	StringTie	exon	14059391	14059530	.	-	.	transcript_	MSTRG.28874.2	gene_id	MSTRG.28874;
5	StringTie	exon	14061205	14062357	.	-	.	transcript_	MSTRG.28874.2	gene_id	MSTRG.28874;
5	StringTie	exon	14062851	14062925	.	-	.	transcript_	MSTRG.28874.2	gene_id	MSTRG.28874;
5	StringTie	exon	14067842	14067882	.	-	.	transcript_	MSTRG.28874.2	gene_id	MSTRG.28874;
5	StringTie	transcrip	14030484	14068010	.	-	.	transcript_	MSTRG.28874.4	gene_id	MSTRG.28874;
5	StringTie	exon	14030484	14031067	.	-	.	transcript_	MSTRG.28874.4	gene_id	MSTRG.28874;
5	StringTie	exon	14067842	14068010	.	-	.	transcript_	MSTRG.28874.4	gene_id	MSTRG.28874;
5	StringTie	transcrip	14030484	14068160	.	-	.	transcript_	MSTRG.28874.3	gene_id	MSTRG.28874;

5	StringTie	exon	14030484	14031067	.	-	.	transcript_	MSTRG.28874.3	gene_id	MSTRG.28874;
5	StringTie	exon	14056824	14057321	.	-	.	transcript_	MSTRG.28874.3	gene_id	MSTRG.28874;
5	StringTie	exon	14059391	14059530	.	-	.	transcript_	MSTRG.28874.3	gene_id	MSTRG.28874;
5	StringTie	exon	14061205	14062357	.	-	.	transcript_	MSTRG.28874.3	gene_id	MSTRG.28874;
5	StringTie	exon	14067842	14068160	.	-	.	transcript_	MSTRG.28874.3	gene_id	MSTRG.28874;
5	StringTie	transcrip	14032397	14061304	.	-	.	transcript_	MSTRG.28874.5	gene_id	MSTRG.28874;
5	StringTie	exon	14032397	14032627	.	-	.	transcript_	MSTRG.28874.5	gene_id	MSTRG.28874;
5	StringTie	exon	14056824	14057044	.	-	.	transcript_	MSTRG.28874.5	gene_id	MSTRG.28874;
5	StringTie	exon	14059391	14059530	.	-	.	transcript_	MSTRG.28874.5	gene_id	MSTRG.28874;
5	StringTie	exon	14061205	14061304	.	-	.	transcript_	MSTRG.28874.5	gene_id	MSTRG.28874;
5	StringTie	transcrip	16006972	16008494	.	-	.	transcript_	MSTRG.28946.1	gene_id	MSTRG.28946;
5	StringTie	exon	16006972	16007081	.	-	.	transcript_	MSTRG.28946.1	gene_id	MSTRG.28946;
5	StringTie	exon	16008040	16008253	.	-	.	transcript_	MSTRG.28946.1	gene_id	MSTRG.28946;
5	StringTie	exon	16008406	16008494	.	-	.	transcript_	MSTRG.28946.1	gene_id	MSTRG.28946;
5	StringTie	transcrip	16006972	16016114	.	-	.	transcript_	MSTRG.28946.2	gene_id	MSTRG.28946;
5	StringTie	exon	16006972	16007081	.	-	.	transcript_	MSTRG.28946.2	gene_id	MSTRG.28946;
5	StringTie	exon	16008040	16008253	.	-	.	transcript_	MSTRG.28946.2	gene_id	MSTRG.28946;
5	StringTie	exon	16011981	16012180	.	-	.	transcript_	MSTRG.28946.2	gene_id	MSTRG.28946;
5	StringTie	exon	16016048	16016114	.	-	.	transcript_	MSTRG.28946.2	gene_id	MSTRG.28946;
5	StringTie	transcrip	16012070	16016120	.	-	.	transcript_	MSTRG.28946.3	gene_id	MSTRG.28946;
5	StringTie	exon	16012070	16012180	.	-	.	transcript_	MSTRG.28946.3	gene_id	MSTRG.28946;
5	StringTie	exon	16015953	16016120	.	-	.	transcript_	MSTRG.28946.3	gene_id	MSTRG.28946;
5	StringTie	transcrip	16265090	16321342	.	-	.	transcript_	MSTRG.28962.1	gene_id	MSTRG.28962;
5	StringTie	exon	16265090	16265107	.	-	.	transcript_	MSTRG.28962.1	gene_id	MSTRG.28962;
5	StringTie	exon	16320973	16321342	.	-	.	transcript_	MSTRG.28962.1	gene_id	MSTRG.28962;
5	StringTie	transcrip	19196497	19197119	.	-	.	transcript_	MSTRG.29032.1	gene_id	MSTRG.29032;
5	StringTie	exon	19196497	19196555	.	-	.	transcript_	MSTRG.29032.1	gene_id	MSTRG.29032;
5	StringTie	exon	19196633	19197119	.	-	.	transcript_	MSTRG.29032.1	gene_id	MSTRG.29032;
5	StringTie	transcrip	19211066	19212779	.	-	.	transcript_	MSTRG.29039.1	gene_id	MSTRG.29039;
5	StringTie	exon	19211066	19212053	.	-	.	transcript_	MSTRG.29039.1	gene_id	MSTRG.29039;
5	StringTie	exon	19212489	19212779	.	-	.	transcript_	MSTRG.29039.1	gene_id	MSTRG.29039;
5	StringTie	transcrip	19290184	19293598	.	-	.	transcript_	MSTRG.29043.1	gene_id	MSTRG.29043;
5	StringTie	exon	19290184	19292060	.	-	.	transcript_	MSTRG.29043.1	gene_id	MSTRG.29043;
5	StringTie	exon	19292854	19293598	.	-	.	transcript_	MSTRG.29043.1	gene_id	MSTRG.29043;
5	StringTie	transcrip	21529880	21545040	.	-	.	transcript_	MSTRG.29105.1	gene_id	MSTRG.29105;

5	StringTie	exon	21529880	21530259	.	-	.	transcript_	MSTRG.29105.1	gene_id	MSTRG.29105;
5	StringTie	exon	21536633	21536897	.	-	.	transcript_	MSTRG.29105.1	gene_id	MSTRG.29105;
5	StringTie	exon	21538638	21538759	.	-	.	transcript_	MSTRG.29105.1	gene_id	MSTRG.29105;
5	StringTie	exon	21543057	21543230	.	-	.	transcript_	MSTRG.29105.1	gene_id	MSTRG.29105;
5	StringTie	exon	21544169	21545040	.	-	.	transcript_	MSTRG.29105.1	gene_id	MSTRG.29105;
5	StringTie	transcrip	21529895	21543231	.	-	.	transcript_	MSTRG.29105.2	gene_id	MSTRG.29105;
5	StringTie	exon	21529895	21530259	.	-	.	transcript_	MSTRG.29105.2	gene_id	MSTRG.29105;
5	StringTie	exon	21536633	21538759	.	-	.	transcript_	MSTRG.29105.2	gene_id	MSTRG.29105;
5	StringTie	exon	21543057	21543231	.	-	.	transcript_	MSTRG.29105.2	gene_id	MSTRG.29105;
5	StringTie	transcrip	21532921	21542248	.	-	.	transcript_	MSTRG.29105.3	gene_id	MSTRG.29105;
5	StringTie	exon	21532921	21532994	.	-	.	transcript_	MSTRG.29105.3	gene_id	MSTRG.29105;
5	StringTie	exon	21536633	21542248	.	-	.	transcript_	MSTRG.29105.3	gene_id	MSTRG.29105;
5	StringTie	transcrip	24872428	24874957	.	-	.	transcript_	MSTRG.29281.1	gene_id	MSTRG.29281;
5	StringTie	exon	24872428	24874223	.	-	.	transcript_	MSTRG.29281.1	gene_id	MSTRG.29281;
5	StringTie	exon	24874540	24874957	.	-	.	transcript_	MSTRG.29281.1	gene_id	MSTRG.29281;
5	StringTie	transcrip	34375861	34384546	.	-	.	transcript_	MSTRG.29435.2	gene_id	MSTRG.29435;
5	StringTie	exon	34375861	34376738	.	-	.	transcript_	MSTRG.29435.2	gene_id	MSTRG.29435;
5	StringTie	exon	34384211	34384546	.	-	.	transcript_	MSTRG.29435.2	gene_id	MSTRG.29435;
5	StringTie	transcrip	34423300	34424201	.	-	.	transcript_	MSTRG.29446.1	gene_id	MSTRG.29446;
5	StringTie	exon	34423300	34423805	.	-	.	transcript_	MSTRG.29446.1	gene_id	MSTRG.29446;
5	StringTie	exon	34424093	34424201	.	-	.	transcript_	MSTRG.29446.1	gene_id	MSTRG.29446;
5	StringTie	transcrip	34721915	34730997	.	-	.	transcript_	MSTRG.29454.1	gene_id	MSTRG.29454;
5	StringTie	exon	34721915	34722143	.	-	.	transcript_	MSTRG.29454.1	gene_id	MSTRG.29454;
5	StringTie	exon	34730580	34730997	.	-	.	transcript_	MSTRG.29454.1	gene_id	MSTRG.29454;
5	StringTie	transcrip	47335142	47403245	.	-	.	transcript_	MSTRG.29612.1	gene_id	MSTRG.29612;
5	StringTie	exon	47335142	47336122	.	-	.	transcript_	MSTRG.29612.1	gene_id	MSTRG.29612;
5	StringTie	exon	47403174	47403245	.	-	.	transcript_	MSTRG.29612.1	gene_id	MSTRG.29612;
5	StringTie	transcrip	49156547	49159735	.	-	.	transcript_	MSTRG.29630.1	gene_id	MSTRG.29630;
5	StringTie	exon	49156547	49159247	.	-	.	transcript_	MSTRG.29630.1	gene_id	MSTRG.29630;
5	StringTie	exon	49159586	49159735	.	-	.	transcript_	MSTRG.29630.1	gene_id	MSTRG.29630;
5	StringTie	transcrip	50992737	51040469	.	-	.	transcript_	MSTRG.29729.1	gene_id	MSTRG.29729;
5	StringTie	exon	50992737	50992763	.	-	.	transcript_	MSTRG.29729.1	gene_id	MSTRG.29729;
5	StringTie	exon	51006181	51006215	.	-	.	transcript_	MSTRG.29729.1	gene_id	MSTRG.29729;
5	StringTie	exon	51037127	51037211	.	-	.	transcript_	MSTRG.29729.1	gene_id	MSTRG.29729;
5	StringTie	exon	51040103	51040469	.	-	.	transcript_	MSTRG.29729.1	gene_id	MSTRG.29729;

5	StringTie	transcrip	51670381	51671219	.	-	.	transcript_	MSTRG.29739.1	gene_id	MSTRG.29739;
5	StringTie	exon	51670381	51670706	.	-	.	transcript_	MSTRG.29739.1	gene_id	MSTRG.29739;
5	StringTie	exon	51670754	51671219	.	-	.	transcript_	MSTRG.29739.1	gene_id	MSTRG.29739;
5	StringTie	transcrip	59712262	59727461	.	-	.	transcript_	MSTRG.29879.1	gene_id	MSTRG.29879;
5	StringTie	exon	59712262	59712749	.	-	.	transcript_	MSTRG.29879.1	gene_id	MSTRG.29879;
5	StringTie	exon	59727351	59727461	.	-	.	transcript_	MSTRG.29879.1	gene_id	MSTRG.29879;
5	StringTie	transcrip	61910608	61972716	.	-	.	transcript_	MSTRG.29938.1	gene_id	MSTRG.29938;
5	StringTie	exon	61910608	61910806	.	-	.	transcript_	MSTRG.29938.1	gene_id	MSTRG.29938;
5	StringTie	exon	61913250	61913350	.	-	.	transcript_	MSTRG.29938.1	gene_id	MSTRG.29938;
5	StringTie	exon	61946285	61946340	.	-	.	transcript_	MSTRG.29938.1	gene_id	MSTRG.29938;
5	StringTie	exon	61957083	61957221	.	-	.	transcript_	MSTRG.29938.1	gene_id	MSTRG.29938;
5	StringTie	exon	61966567	61966635	.	-	.	transcript_	MSTRG.29938.1	gene_id	MSTRG.29938;
5	StringTie	exon	61972460	61972716	.	-	.	transcript_	MSTRG.29938.1	gene_id	MSTRG.29938;
5	StringTie	transcrip	61912593	61915133	.	-	.	transcript_	MSTRG.29938.2	gene_id	MSTRG.29938;
5	StringTie	exon	61912593	61912668	.	-	.	transcript_	MSTRG.29938.2	gene_id	MSTRG.29938;
5	StringTie	exon	61913250	61913350	.	-	.	transcript_	MSTRG.29938.2	gene_id	MSTRG.29938;
5	StringTie	exon	61915106	61915133	.	-	.	transcript_	MSTRG.29938.2	gene_id	MSTRG.29938;
5	StringTie	transcrip	61912594	61972630	.	-	.	transcript_	MSTRG.29938.3	gene_id	MSTRG.29938;
5	StringTie	exon	61912594	61912668	.	-	.	transcript_	MSTRG.29938.3	gene_id	MSTRG.29938;
5	StringTie	exon	61913250	61913350	.	-	.	transcript_	MSTRG.29938.3	gene_id	MSTRG.29938;
5	StringTie	exon	61946285	61946340	.	-	.	transcript_	MSTRG.29938.3	gene_id	MSTRG.29938;
5	StringTie	exon	61957083	61957221	.	-	.	transcript_	MSTRG.29938.3	gene_id	MSTRG.29938;
5	StringTie	exon	61966567	61966635	.	-	.	transcript_	MSTRG.29938.3	gene_id	MSTRG.29938;
5	StringTie	exon	61972460	61972630	.	-	.	transcript_	MSTRG.29938.3	gene_id	MSTRG.29938;
5	StringTie	transcrip	61913333	61972559	.	-	.	transcript_	MSTRG.29938.4	gene_id	MSTRG.29938;
5	StringTie	exon	61913333	61913350	.	-	.	transcript_	MSTRG.29938.4	gene_id	MSTRG.29938;
5	StringTie	exon	61946285	61946340	.	-	.	transcript_	MSTRG.29938.4	gene_id	MSTRG.29938;
5	StringTie	exon	61957083	61957221	.	-	.	transcript_	MSTRG.29938.4	gene_id	MSTRG.29938;
5	StringTie	exon	61972460	61972559	.	-	.	transcript_	MSTRG.29938.4	gene_id	MSTRG.29938;
5	StringTie	transcrip	61915236	61972608	.	-	.	transcript_	MSTRG.29938.5	gene_id	MSTRG.29938;
5	StringTie	exon	61915236	61915259	.	-	.	transcript_	MSTRG.29938.5	gene_id	MSTRG.29938;
5	StringTie	exon	61946285	61946340	.	-	.	transcript_	MSTRG.29938.5	gene_id	MSTRG.29938;
5	StringTie	exon	61957083	61957221	.	-	.	transcript_	MSTRG.29938.5	gene_id	MSTRG.29938;
5	StringTie	exon	61966567	61966635	.	-	.	transcript_	MSTRG.29938.5	gene_id	MSTRG.29938;
5	StringTie	exon	61972460	61972608	.	-	.	transcript_	MSTRG.29938.5	gene_id	MSTRG.29938;

5	StringTie	transcrip	61923174	61972749	.	-	.	transcript_	MSTRG.29938.(	gene_id	MSTRG.29938;
5	StringTie	exon	61923174	61925675	.	-	.	transcript_	MSTRG.29938.(	gene_id	MSTRG.29938;
5	StringTie	exon	61928911	61929058	.	-	.	transcript_	MSTRG.29938.(	gene_id	MSTRG.29938;
5	StringTie	exon	61946285	61946340	.	-	.	transcript_	MSTRG.29938.(	gene_id	MSTRG.29938;
5	StringTie	exon	61957083	61957221	.	-	.	transcript_	MSTRG.29938.(	gene_id	MSTRG.29938;
5	StringTie	exon	61966567	61966635	.	-	.	transcript_	MSTRG.29938.(	gene_id	MSTRG.29938;
5	StringTie	exon	61972460	61972749	.	-	.	transcript_	MSTRG.29938.(	gene_id	MSTRG.29938;
5	StringTie	transcrip	61923188	61972738	.	-	.	transcript_	MSTRG.29938.7	gene_id	MSTRG.29938;
5	StringTie	exon	61923188	61925675	.	-	.	transcript_	MSTRG.29938.7	gene_id	MSTRG.29938;
5	StringTie	exon	61946285	61946340	.	-	.	transcript_	MSTRG.29938.7	gene_id	MSTRG.29938;
5	StringTie	exon	61957083	61957221	.	-	.	transcript_	MSTRG.29938.7	gene_id	MSTRG.29938;
5	StringTie	exon	61966567	61966635	.	-	.	transcript_	MSTRG.29938.7	gene_id	MSTRG.29938;
5	StringTie	exon	61972460	61972738	.	-	.	transcript_	MSTRG.29938.7	gene_id	MSTRG.29938;
5	StringTie	transcrip	61923206	61972559	.	-	.	transcript_	MSTRG.29938.8	gene_id	MSTRG.29938;
5	StringTie	exon	61923206	61925675	.	-	.	transcript_	MSTRG.29938.8	gene_id	MSTRG.29938;
5	StringTie	exon	61957083	61957221	.	-	.	transcript_	MSTRG.29938.8	gene_id	MSTRG.29938;
5	StringTie	exon	61966567	61966635	.	-	.	transcript_	MSTRG.29938.8	gene_id	MSTRG.29938;
5	StringTie	exon	61972460	61972559	.	-	.	transcript_	MSTRG.29938.8	gene_id	MSTRG.29938;
5	StringTie	transcrip	61953677	61972716	.	-	.	transcript_	MSTRG.29938.9	gene_id	MSTRG.29938;
5	StringTie	exon	61953677	61955289	.	-	.	transcript_	MSTRG.29938.9	gene_id	MSTRG.29938;
5	StringTie	exon	61957083	61957221	.	-	.	transcript_	MSTRG.29938.9	gene_id	MSTRG.29938;
5	StringTie	exon	61966567	61966635	.	-	.	transcript_	MSTRG.29938.9	gene_id	MSTRG.29938;
5	StringTie	exon	61972460	61972716	.	-	.	transcript_	MSTRG.29938.9	gene_id	MSTRG.29938;
5	StringTie	transcrip	64105293	64106001	.	-	.	transcript_	MSTRG.30019.1	gene_id	MSTRG.30019;
5	StringTie	exon	64105293	64105515	.	-	.	transcript_	MSTRG.30019.1	gene_id	MSTRG.30019;
5	StringTie	exon	64105723	64106001	.	-	.	transcript_	MSTRG.30019.1	gene_id	MSTRG.30019;
5	StringTie	transcrip	70422012	70423323	.	-	.	transcript_	MSTRG.30146.1	gene_id	MSTRG.30146;
5	StringTie	exon	70422012	70422734	.	-	.	transcript_	MSTRG.30146.1	gene_id	MSTRG.30146;
5	StringTie	exon	70422994	70423323	.	-	.	transcript_	MSTRG.30146.1	gene_id	MSTRG.30146;
5	StringTie	transcrip	77933968	77936406	.	-	.	transcript_	MSTRG.30248.1	gene_id	MSTRG.30248;
5	StringTie	exon	77933968	77935747	.	-	.	transcript_	MSTRG.30248.1	gene_id	MSTRG.30248;
5	StringTie	exon	77936261	77936406	.	-	.	transcript_	MSTRG.30248.1	gene_id	MSTRG.30248;
5	StringTie	transcrip	77934000	77936365	.	-	.	transcript_	MSTRG.30248.2	gene_id	MSTRG.30248;
5	StringTie	exon	77934000	77935747	.	-	.	transcript_	MSTRG.30248.2	gene_id	MSTRG.30248;
5	StringTie	exon	77936247	77936365	.	-	.	transcript_	MSTRG.30248.2	gene_id	MSTRG.30248;

5	StringTie	transcrip	77935316	77936345	.	-	.	transcript_	MSTRG.30248.:	gene_id	MSTRG.30248;
5	StringTie	exon	77935316	77936047	.	-	.	transcript_	MSTRG.30248.:	gene_id	MSTRG.30248;
5	StringTie	exon	77936261	77936345	.	-	.	transcript_	MSTRG.30248.:	gene_id	MSTRG.30248;
5	StringTie	transcrip	82751891	82754980	.	-	.	transcript_	MSTRG.30387.	gene_id	MSTRG.30387;
5	StringTie	exon	82751891	82752040	.	-	.	transcript_	MSTRG.30387.	gene_id	MSTRG.30387;
5	StringTie	exon	82754872	82754980	.	-	.	transcript_	MSTRG.30387.	gene_id	MSTRG.30387;
5	StringTie	transcrip	86814194	86814771	.	-	.	transcript_	MSTRG.30425.	gene_id	MSTRG.30425;
5	StringTie	exon	86814194	86814391	.	-	.	transcript_	MSTRG.30425.	gene_id	MSTRG.30425;
5	StringTie	exon	86814640	86814771	.	-	.	transcript_	MSTRG.30425.	gene_id	MSTRG.30425;
5	StringTie	transcrip	89240156	89241476	.	-	.	transcript_	MSTRG.30497.	gene_id	MSTRG.30497;
5	StringTie	exon	89240156	89240380	.	-	.	transcript_	MSTRG.30497.	gene_id	MSTRG.30497;
5	StringTie	exon	89241456	89241476	.	-	.	transcript_	MSTRG.30497.	gene_id	MSTRG.30497;
5	StringTie	transcrip	89285487	89301931	.	-	.	transcript_	MSTRG.30507.	gene_id	MSTRG.30507;
5	StringTie	exon	89285487	89285579	.	-	.	transcript_	MSTRG.30507.	gene_id	MSTRG.30507;
5	StringTie	exon	89286740	89286799	.	-	.	transcript_	MSTRG.30507.	gene_id	MSTRG.30507;
5	StringTie	exon	89295168	89295346	.	-	.	transcript_	MSTRG.30507.	gene_id	MSTRG.30507;
5	StringTie	exon	89301806	89301931	.	-	.	transcript_	MSTRG.30507.	gene_id	MSTRG.30507;
5	StringTie	transcrip	90498102	90516427	.	-	.	transcript_	MSTRG.30565.	gene_id	MSTRG.30565;
5	StringTie	exon	90498102	90498996	.	-	.	transcript_	MSTRG.30565.	gene_id	MSTRG.30565;
5	StringTie	exon	90515733	90516427	.	-	.	transcript_	MSTRG.30565.	gene_id	MSTRG.30565;
5	StringTie	transcrip	91549028	91558714	.	-	.	transcript_	MSTRG.30568.	gene_id	MSTRG.30568;
5	StringTie	exon	91549028	91549127	.	-	.	transcript_	MSTRG.30568.	gene_id	MSTRG.30568;
5	StringTie	exon	91558562	91558714	.	-	.	transcript_	MSTRG.30568.	gene_id	MSTRG.30568;
5	StringTie	transcrip	99210834	99227168	.	-	.	transcript_	MSTRG.30645.	gene_id	MSTRG.30645;
5	StringTie	exon	99210834	99211198	.	-	.	transcript_	MSTRG.30645.	gene_id	MSTRG.30645;
5	StringTie	exon	99226562	99227168	.	-	.	transcript_	MSTRG.30645.	gene_id	MSTRG.30645;
6	StringTie	transcrip	3772143	3773225	.	+	.	transcript_	MSTRG.30790.	gene_id	MSTRG.30790;
6	StringTie	exon	3772143	3772345	.	+	.	transcript_	MSTRG.30790.	gene_id	MSTRG.30790;
6	StringTie	exon	3772829	3773225	.	+	.	transcript_	MSTRG.30790.	gene_id	MSTRG.30790;
6	StringTie	transcrip	10745568	10753095	.	+	.	transcript_	MSTRG.30894.	gene_id	MSTRG.30894;
6	StringTie	exon	10745568	10745702	.	+	.	transcript_	MSTRG.30894.	gene_id	MSTRG.30894;
6	StringTie	exon	10753023	10753095	.	+	.	transcript_	MSTRG.30894.	gene_id	MSTRG.30894;
6	StringTie	transcrip	13674896	13675970	.	+	.	transcript_	MSTRG.30986.'	gene_id	MSTRG.30986;
6	StringTie	exon	13674896	13675150	.	+	.	transcript_	MSTRG.30986.'	gene_id	MSTRG.30986;
6	StringTie	exon	13675814	13675970	.	+	.	transcript_	MSTRG.30986.'	gene_id	MSTRG.30986;

6	StringTie	transcrip	17673287	17681836	.	+	.	transcript_	MSTRG.31030.1	gene_id	MSTRG.31030;
6	StringTie	exon	17673287	17673319	.	+	.	transcript_	MSTRG.31030.1	gene_id	MSTRG.31030;
6	StringTie	exon	17677823	17681836	.	+	.	transcript_	MSTRG.31030.1	gene_id	MSTRG.31030;
6	StringTie	transcrip	18335933	18338738	.	+	.	transcript_	MSTRG.31035.1	gene_id	MSTRG.31035;
6	StringTie	exon	18335933	18336006	.	+	.	transcript_	MSTRG.31035.1	gene_id	MSTRG.31035;
6	StringTie	exon	18336240	18338738	.	+	.	transcript_	MSTRG.31035.1	gene_id	MSTRG.31035;
6	StringTie	transcrip	26233953	26235815	.	+	.	transcript_	MSTRG.31103.1	gene_id	MSTRG.31103;
6	StringTie	exon	26233953	26234142	.	+	.	transcript_	MSTRG.31103.1	gene_id	MSTRG.31103;
6	StringTie	exon	26235704	26235815	.	+	.	transcript_	MSTRG.31103.1	gene_id	MSTRG.31103;
6	StringTie	transcrip	27727638	27733899	.	+	.	transcript_	MSTRG.31140.1	gene_id	MSTRG.31140;
6	StringTie	exon	27727638	27728283	.	+	.	transcript_	MSTRG.31140.1	gene_id	MSTRG.31140;
6	StringTie	exon	27733580	27733899	.	+	.	transcript_	MSTRG.31140.1	gene_id	MSTRG.31140;
6	StringTie	transcrip	30562254	30568110	.	+	.	transcript_	MSTRG.31229.1	gene_id	MSTRG.31229;
6	StringTie	exon	30562254	30563345	.	+	.	transcript_	MSTRG.31229.1	gene_id	MSTRG.31229;
6	StringTie	exon	30563569	30563615	.	+	.	transcript_	MSTRG.31229.1	gene_id	MSTRG.31229;
6	StringTie	exon	30568053	30568110	.	+	.	transcript_	MSTRG.31229.1	gene_id	MSTRG.31229;
6	StringTie	transcrip	30568385	30570979	.	+	.	transcript_	MSTRG.31227.1	gene_id	MSTRG.31227;
6	StringTie	exon	30568385	30568505	.	+	.	transcript_	MSTRG.31227.1	gene_id	MSTRG.31227;
6	StringTie	exon	30570645	30570979	.	+	.	transcript_	MSTRG.31227.1	gene_id	MSTRG.31227;
6	StringTie	transcrip	43085883	43087500	.	+	.	transcript_	MSTRG.31354.2	gene_id	MSTRG.31354;
6	StringTie	exon	43085883	43086398	.	+	.	transcript_	MSTRG.31354.2	gene_id	MSTRG.31354;
6	StringTie	exon	43086869	43087500	.	+	.	transcript_	MSTRG.31354.2	gene_id	MSTRG.31354;
6	StringTie	transcrip	43085883	43087531	.	+	.	transcript_	MSTRG.31354.1	gene_id	MSTRG.31354;
6	StringTie	exon	43085883	43086215	.	+	.	transcript_	MSTRG.31354.1	gene_id	MSTRG.31354;
6	StringTie	exon	43086869	43087531	.	+	.	transcript_	MSTRG.31354.1	gene_id	MSTRG.31354;
6	StringTie	transcrip	43087646	43090630	.	+	.	transcript_	MSTRG.31355.1	gene_id	MSTRG.31355;
6	StringTie	exon	43087646	43087837	.	+	.	transcript_	MSTRG.31355.1	gene_id	MSTRG.31355;
6	StringTie	exon	43090482	43090630	.	+	.	transcript_	MSTRG.31355.1	gene_id	MSTRG.31355;
6	StringTie	transcrip	48231448	48239613	.	+	.	transcript_	MSTRG.31613.2	gene_id	MSTRG.31613;
6	StringTie	exon	48231448	48232278	.	+	.	transcript_	MSTRG.31613.2	gene_id	MSTRG.31613;
6	StringTie	exon	48232617	48232824	.	+	.	transcript_	MSTRG.31613.2	gene_id	MSTRG.31613;
6	StringTie	exon	48236268	48239613	.	+	.	transcript_	MSTRG.31613.2	gene_id	MSTRG.31613;
6	StringTie	transcrip	48713347	48722357	.	+	.	transcript_	MSTRG.31634.1	gene_id	MSTRG.31634;
6	StringTie	exon	48713347	48713452	.	+	.	transcript_	MSTRG.31634.1	gene_id	MSTRG.31634;
6	StringTie	exon	48719984	48720178	.	+	.	transcript_	MSTRG.31634.1	gene_id	MSTRG.31634;

6	StringTie	exon	48721541	48722357	.	+	.	transcript_	MSTRG.31634.1	gene_id	MSTRG.31634;
6	StringTie	transcrip	49063715	49083320	.	+	.	transcript_	MSTRG.31663.3	gene_id	MSTRG.31663;
6	StringTie	exon	49063715	49063947	.	+	.	transcript_	MSTRG.31663.3	gene_id	MSTRG.31663;
6	StringTie	exon	49065666	49065786	.	+	.	transcript_	MSTRG.31663.3	gene_id	MSTRG.31663;
6	StringTie	exon	49083254	49083320	.	+	.	transcript_	MSTRG.31663.3	gene_id	MSTRG.31663;
6	StringTie	transcrip	50569762	50571747	.	+	.	transcript_	MSTRG.31742.1	gene_id	MSTRG.31742;
6	StringTie	exon	50569762	50569853	.	+	.	transcript_	MSTRG.31742.1	gene_id	MSTRG.31742;
6	StringTie	exon	50571620	50571747	.	+	.	transcript_	MSTRG.31742.1	gene_id	MSTRG.31742;
6	StringTie	transcrip	54061493	54068338	.	+	.	transcript_	MSTRG.31936.1	gene_id	MSTRG.31936;
6	StringTie	exon	54061493	54061586	.	+	.	transcript_	MSTRG.31936.1	gene_id	MSTRG.31936;
6	StringTie	exon	54068223	54068338	.	+	.	transcript_	MSTRG.31936.1	gene_id	MSTRG.31936;
6	StringTie	transcrip	57085514	57093420	.	+	.	transcript_	MSTRG.32090.1	gene_id	MSTRG.32090;
6	StringTie	exon	57085514	57086794	.	+	.	transcript_	MSTRG.32090.1	gene_id	MSTRG.32090;
6	StringTie	exon	57092334	57092579	.	+	.	transcript_	MSTRG.32090.1	gene_id	MSTRG.32090;
6	StringTie	exon	57093248	57093420	.	+	.	transcript_	MSTRG.32090.1	gene_id	MSTRG.32090;
6	StringTie	transcrip	57085778	57093345	.	+	.	transcript_	MSTRG.32090.2	gene_id	MSTRG.32090;
6	StringTie	exon	57085778	57086794	.	+	.	transcript_	MSTRG.32090.2	gene_id	MSTRG.32090;
6	StringTie	exon	57093268	57093345	.	+	.	transcript_	MSTRG.32090.2	gene_id	MSTRG.32090;
6	StringTie	transcrip	57091147	57116060	.	+	.	transcript_	MSTRG.32092.1	gene_id	MSTRG.32092;
6	StringTie	exon	57091147	57091251	.	+	.	transcript_	MSTRG.32092.1	gene_id	MSTRG.32092;
6	StringTie	exon	57115955	57116060	.	+	.	transcript_	MSTRG.32092.1	gene_id	MSTRG.32092;
6	StringTie	transcrip	57092705	57118651	.	+	.	transcript_	MSTRG.32093.1	gene_id	MSTRG.32093;
6	StringTie	exon	57092705	57093023	.	+	.	transcript_	MSTRG.32093.1	gene_id	MSTRG.32093;
6	StringTie	exon	57118398	57118651	.	+	.	transcript_	MSTRG.32093.1	gene_id	MSTRG.32093;
6	StringTie	transcrip	57093730	57094674	.	+	.	transcript_	MSTRG.32094.1	gene_id	MSTRG.32094;
6	StringTie	exon	57093730	57093801	.	+	.	transcript_	MSTRG.32094.1	gene_id	MSTRG.32094;
6	StringTie	exon	57094106	57094674	.	+	.	transcript_	MSTRG.32094.1	gene_id	MSTRG.32094;
6	StringTie	transcrip	57262645	57264310	.	+	.	transcript_	MSTRG.32068.2	gene_id	MSTRG.32068;
6	StringTie	exon	57262645	57262714	.	+	.	transcript_	MSTRG.32068.2	gene_id	MSTRG.32068;
6	StringTie	exon	57263576	57264310	.	+	.	transcript_	MSTRG.32068.2	gene_id	MSTRG.32068;
6	StringTie	transcrip	57263401	57264529	.	+	.	transcript_	MSTRG.32068.3	gene_id	MSTRG.32068;
6	StringTie	exon	57263401	57263474	.	+	.	transcript_	MSTRG.32068.3	gene_id	MSTRG.32068;
6	StringTie	exon	57263576	57264529	.	+	.	transcript_	MSTRG.32068.3	gene_id	MSTRG.32068;
6	StringTie	transcrip	57297256	57299675	.	+	.	transcript_	MSTRG.32081.1	gene_id	MSTRG.32081;
6	StringTie	exon	57297256	57297462	.	+	.	transcript_	MSTRG.32081.1	gene_id	MSTRG.32081;

6	StringTie	exon	57299143	57299675	.	+	.	transcript_	MSTRG.32081.1	gene_id	MSTRG.32081;
6	StringTie	transcrip	59593578	59597688	.	+	.	transcript_	MSTRG.32215.1	gene_id	MSTRG.32215;
6	StringTie	exon	59593578	59593661	.	+	.	transcript_	MSTRG.32215.1	gene_id	MSTRG.32215;
6	StringTie	exon	59597382	59597688	.	+	.	transcript_	MSTRG.32215.1	gene_id	MSTRG.32215;
6	StringTie	transcrip	61849276	61852249	.	+	.	transcript_	MSTRG.32322.6	gene_id	MSTRG.32322;
6	StringTie	exon	61849276	61849397	.	+	.	transcript_	MSTRG.32322.6	gene_id	MSTRG.32322;
6	StringTie	exon	61852163	61852249	.	+	.	transcript_	MSTRG.32322.6	gene_id	MSTRG.32322;
6	StringTie	transcrip	62644069	62644950	.	+	.	transcript_	MSTRG.32394.1	gene_id	MSTRG.32394;
6	StringTie	exon	62644069	62644617	.	+	.	transcript_	MSTRG.32394.1	gene_id	MSTRG.32394;
6	StringTie	exon	62644663	62644950	.	+	.	transcript_	MSTRG.32394.1	gene_id	MSTRG.32394;
6	StringTie	transcrip	69396224	69397240	.	+	.	transcript_	MSTRG.32537.1	gene_id	MSTRG.32537;
6	StringTie	exon	69396224	69396455	.	+	.	transcript_	MSTRG.32537.1	gene_id	MSTRG.32537;
6	StringTie	exon	69397194	69397240	.	+	.	transcript_	MSTRG.32537.1	gene_id	MSTRG.32537;
6	StringTie	transcrip	69561989	69563635	.	+	.	transcript_	MSTRG.32543.1	gene_id	MSTRG.32543;
6	StringTie	exon	69561989	69562592	.	+	.	transcript_	MSTRG.32543.1	gene_id	MSTRG.32543;
6	StringTie	exon	69562617	69563635	.	+	.	transcript_	MSTRG.32543.1	gene_id	MSTRG.32543;
6	StringTie	transcrip	69567813	69578970	.	+	.	transcript_	MSTRG.32544.1	gene_id	MSTRG.32544;
6	StringTie	exon	69567813	69568218	.	+	.	transcript_	MSTRG.32544.1	gene_id	MSTRG.32544;
6	StringTie	exon	69577230	69578970	.	+	.	transcript_	MSTRG.32544.1	gene_id	MSTRG.32544;
6	StringTie	transcrip	71235290	71236392	.	+	.	transcript_	MSTRG.32596.1	gene_id	MSTRG.32596;
6	StringTie	exon	71235290	71235381	.	+	.	transcript_	MSTRG.32596.1	gene_id	MSTRG.32596;
6	StringTie	exon	71236197	71236392	.	+	.	transcript_	MSTRG.32596.1	gene_id	MSTRG.32596;
6	StringTie	transcrip	72544519	72559548	.	+	.	transcript_	MSTRG.32628.1	gene_id	MSTRG.32628;
6	StringTie	exon	72544519	72544743	.	+	.	transcript_	MSTRG.32628.1	gene_id	MSTRG.32628;
6	StringTie	exon	72558498	72559548	.	+	.	transcript_	MSTRG.32628.1	gene_id	MSTRG.32628;
6	StringTie	transcrip	72557006	72559515	.	+	.	transcript_	MSTRG.32628.2	gene_id	MSTRG.32628;
6	StringTie	exon	72557006	72558003	.	+	.	transcript_	MSTRG.32628.2	gene_id	MSTRG.32628;
6	StringTie	exon	72558498	72559515	.	+	.	transcript_	MSTRG.32628.2	gene_id	MSTRG.32628;
6	StringTie	transcrip	72560102	72580706	.	+	.	transcript_	MSTRG.32630.1	gene_id	MSTRG.32630;
6	StringTie	exon	72560102	72561802	.	+	.	transcript_	MSTRG.32630.1	gene_id	MSTRG.32630;
6	StringTie	exon	72580648	72580706	.	+	.	transcript_	MSTRG.32630.1	gene_id	MSTRG.32630;
6	StringTie	transcrip	77708215	77729680	.	+	.	transcript_	MSTRG.32738.1	gene_id	MSTRG.32738;
6	StringTie	exon	77708215	77708246	.	+	.	transcript_	MSTRG.32738.1	gene_id	MSTRG.32738;
6	StringTie	exon	77729128	77729680	.	+	.	transcript_	MSTRG.32738.1	gene_id	MSTRG.32738;
6	StringTie	transcrip	79530693	79563182	.	+	.	transcript_	MSTRG.32746.1	gene_id	MSTRG.32746;

6	StringTie	exon	79530693	79531456	.	+	.	transcript_	MSTRG.32746.1	gene_id	MSTRG.32746;
6	StringTie	exon	79553399	79553541	.	+	.	transcript_	MSTRG.32746.1	gene_id	MSTRG.32746;
6	StringTie	exon	79554860	79554957	.	+	.	transcript_	MSTRG.32746.1	gene_id	MSTRG.32746;
6	StringTie	exon	79562593	79563182	.	+	.	transcript_	MSTRG.32746.1	gene_id	MSTRG.32746;
6	StringTie	transcrip	84417880	84430025	.	+	.	transcript_	MSTRG.32906.1	gene_id	MSTRG.32906;
6	StringTie	exon	84417880	84418300	.	+	.	transcript_	MSTRG.32906.1	gene_id	MSTRG.32906;
6	StringTie	exon	84419733	84419865	.	+	.	transcript_	MSTRG.32906.1	gene_id	MSTRG.32906;
6	StringTie	exon	84429348	84430025	.	+	.	transcript_	MSTRG.32906.1	gene_id	MSTRG.32906;
6	StringTie	transcrip	84697900	84707773	.	+	.	transcript_	MSTRG.32913.1	gene_id	MSTRG.32913;
6	StringTie	exon	84697900	84697963	.	+	.	transcript_	MSTRG.32913.1	gene_id	MSTRG.32913;
6	StringTie	exon	84706200	84707773	.	+	.	transcript_	MSTRG.32913.1	gene_id	MSTRG.32913;
6	StringTie	transcrip	85643321	85649266	.	+	.	transcript_	MSTRG.32964.1	gene_id	MSTRG.32964;
6	StringTie	exon	85643321	85643386	.	+	.	transcript_	MSTRG.32964.1	gene_id	MSTRG.32964;
6	StringTie	exon	85649084	85649266	.	+	.	transcript_	MSTRG.32964.1	gene_id	MSTRG.32964;
6	StringTie	transcrip	87951063	87951875	.	+	.	transcript_	MSTRG.32986.1	gene_id	MSTRG.32986;
6	StringTie	exon	87951063	87951189	.	+	.	transcript_	MSTRG.32986.1	gene_id	MSTRG.32986;
6	StringTie	exon	87951492	87951875	.	+	.	transcript_	MSTRG.32986.1	gene_id	MSTRG.32986;
6	StringTie	transcrip	88108595	88121818	.	+	.	transcript_	MSTRG.32993.1	gene_id	MSTRG.32993;
6	StringTie	exon	88108595	88111293	.	+	.	transcript_	MSTRG.32993.1	gene_id	MSTRG.32993;
6	StringTie	exon	88114585	88121818	.	+	.	transcript_	MSTRG.32993.1	gene_id	MSTRG.32993;
6	StringTie	transcrip	89298403	89311500	.	+	.	transcript_	MSTRG.33042.1	gene_id	MSTRG.33042;
6	StringTie	exon	89298403	89298958	.	+	.	transcript_	MSTRG.33042.1	gene_id	MSTRG.33042;
6	StringTie	exon	89310955	89311500	.	+	.	transcript_	MSTRG.33042.1	gene_id	MSTRG.33042;
6	StringTie	transcrip	91822059	91823310	.	+	.	transcript_	MSTRG.33081.1	gene_id	MSTRG.33081;
6	StringTie	exon	91822059	91822483	.	+	.	transcript_	MSTRG.33081.1	gene_id	MSTRG.33081;
6	StringTie	exon	91822781	91823310	.	+	.	transcript_	MSTRG.33081.1	gene_id	MSTRG.33081;
6	StringTie	transcrip	94803175	94838735	.	+	.	transcript_	MSTRG.33150.2	gene_id	MSTRG.33150;
6	StringTie	exon	94803175	94803478	.	+	.	transcript_	MSTRG.33150.2	gene_id	MSTRG.33150;
6	StringTie	exon	94816990	94817067	.	+	.	transcript_	MSTRG.33150.2	gene_id	MSTRG.33150;
6	StringTie	exon	94838586	94838735	.	+	.	transcript_	MSTRG.33150.2	gene_id	MSTRG.33150;
6	StringTie	transcrip	94803175	94838735	.	+	.	transcript_	MSTRG.33150.1	gene_id	MSTRG.33150;
6	StringTie	exon	94803175	94803478	.	+	.	transcript_	MSTRG.33150.1	gene_id	MSTRG.33150;
6	StringTie	exon	94838586	94838735	.	+	.	transcript_	MSTRG.33150.1	gene_id	MSTRG.33150;
6	StringTie	transcrip	94803254	94838697	.	+	.	transcript_	MSTRG.33150.3	gene_id	MSTRG.33150;
6	StringTie	exon	94803254	94803478	.	+	.	transcript_	MSTRG.33150.3	gene_id	MSTRG.33150;

6	StringTie	exon	94816535	94816676	.	+	.	transcript_	MSTRG.33150.3	gene_id	MSTRG.33150;
6	StringTie	exon	94816990	94817067	.	+	.	transcript_	MSTRG.33150.3	gene_id	MSTRG.33150;
6	StringTie	exon	94838586	94838697	.	+	.	transcript_	MSTRG.33150.3	gene_id	MSTRG.33150;
6	StringTie	transcrip	94814539	94838735	.	+	.	transcript_	MSTRG.33150.5	gene_id	MSTRG.33150;
6	StringTie	exon	94814539	94814808	.	+	.	transcript_	MSTRG.33150.5	gene_id	MSTRG.33150;
6	StringTie	exon	94816990	94817067	.	+	.	transcript_	MSTRG.33150.5	gene_id	MSTRG.33150;
6	StringTie	exon	94838586	94838735	.	+	.	transcript_	MSTRG.33150.5	gene_id	MSTRG.33150;
6	StringTie	transcrip	94814539	94838884	.	+	.	transcript_	MSTRG.33150.4	gene_id	MSTRG.33150;
6	StringTie	exon	94814539	94814808	.	+	.	transcript_	MSTRG.33150.4	gene_id	MSTRG.33150;
6	StringTie	exon	94816535	94816676	.	+	.	transcript_	MSTRG.33150.4	gene_id	MSTRG.33150;
6	StringTie	exon	94816990	94817067	.	+	.	transcript_	MSTRG.33150.4	gene_id	MSTRG.33150;
6	StringTie	exon	94838586	94838884	.	+	.	transcript_	MSTRG.33150.4	gene_id	MSTRG.33150;
6	StringTie	transcrip	94814571	94820467	.	+	.	transcript_	MSTRG.33150.6	gene_id	MSTRG.33150;
6	StringTie	exon	94814571	94814808	.	+	.	transcript_	MSTRG.33150.6	gene_id	MSTRG.33150;
6	StringTie	exon	94816535	94816676	.	+	.	transcript_	MSTRG.33150.6	gene_id	MSTRG.33150;
6	StringTie	exon	94816990	94817067	.	+	.	transcript_	MSTRG.33150.6	gene_id	MSTRG.33150;
6	StringTie	exon	94819363	94820467	.	+	.	transcript_	MSTRG.33150.6	gene_id	MSTRG.33150;
6	StringTie	transcrip	94814571	94838697	.	+	.	transcript_	MSTRG.33150.7	gene_id	MSTRG.33150;
6	StringTie	exon	94814571	94814808	.	+	.	transcript_	MSTRG.33150.7	gene_id	MSTRG.33150;
6	StringTie	exon	94816535	94816676	.	+	.	transcript_	MSTRG.33150.7	gene_id	MSTRG.33150;
6	StringTie	exon	94816990	94817067	.	+	.	transcript_	MSTRG.33150.7	gene_id	MSTRG.33150;
6	StringTie	exon	94838647	94838697	.	+	.	transcript_	MSTRG.33150.7	gene_id	MSTRG.33150;
6	StringTie	transcrip	99673790	99735724	.	+	.	transcript_	MSTRG.33279.1	gene_id	MSTRG.33279;
6	StringTie	exon	99673790	99673837	.	+	.	transcript_	MSTRG.33279.1	gene_id	MSTRG.33279;
6	StringTie	exon	99734556	99735724	.	+	.	transcript_	MSTRG.33279.1	gene_id	MSTRG.33279;
6	StringTie	transcrip	100093687	100095729	.	+	.	transcript_	MSTRG.33272.1	gene_id	MSTRG.33272;
6	StringTie	exon	100093687	100093739	.	+	.	transcript_	MSTRG.33272.1	gene_id	MSTRG.33272;
6	StringTie	exon	100095341	100095729	.	+	.	transcript_	MSTRG.33272.1	gene_id	MSTRG.33272;
6	StringTie	transcrip	101795369	101798808	.	+	.	transcript_	MSTRG.33295.1	gene_id	MSTRG.33295;
6	StringTie	exon	101795369	101795586	.	+	.	transcript_	MSTRG.33295.1	gene_id	MSTRG.33295;
6	StringTie	exon	101798736	101798808	.	+	.	transcript_	MSTRG.33295.1	gene_id	MSTRG.33295;
6	StringTie	transcrip	103391661	103414915	.	+	.	transcript_	MSTRG.33307.1	gene_id	MSTRG.33307;
6	StringTie	exon	103391661	103391832	.	+	.	transcript_	MSTRG.33307.1	gene_id	MSTRG.33307;
6	StringTie	exon	103414590	103414915	.	+	.	transcript_	MSTRG.33307.1	gene_id	MSTRG.33307;
6	StringTie	transcrip	103391722	103414770	.	+	.	transcript_	MSTRG.33307.2	gene_id	MSTRG.33307;

6	StringTie	exon	103391722	103391905	.	+	.	transcript_	MSTRG.33307.2	gene_id	MSTRG.33307;
6	StringTie	exon	103414663	103414770	.	+	.	transcript_	MSTRG.33307.2	gene_id	MSTRG.33307;
6	StringTie	transcrip	106314743	106347679	.	+	.	transcript_	MSTRG.33364.1	gene_id	MSTRG.33364;
6	StringTie	exon	106314743	106314771	.	+	.	transcript_	MSTRG.33364.1	gene_id	MSTRG.33364;
6	StringTie	exon	106346412	106347679	.	+	.	transcript_	MSTRG.33364.1	gene_id	MSTRG.33364;
6	StringTie	transcrip	106347716	106349490	.	+	.	transcript_	MSTRG.33367.1	gene_id	MSTRG.33367;
6	StringTie	exon	106347716	106348274	.	+	.	transcript_	MSTRG.33367.1	gene_id	MSTRG.33367;
6	StringTie	exon	106348390	106349490	.	+	.	transcript_	MSTRG.33367.1	gene_id	MSTRG.33367;
6	StringTie	transcrip	109110534	109126639	.	+	.	transcript_	MSTRG.33430.1	gene_id	MSTRG.33430;
6	StringTie	exon	109110534	109110767	.	+	.	transcript_	MSTRG.33430.1	gene_id	MSTRG.33430;
6	StringTie	exon	109111769	109111853	.	+	.	transcript_	MSTRG.33430.1	gene_id	MSTRG.33430;
6	StringTie	exon	109125216	109126639	.	+	.	transcript_	MSTRG.33430.1	gene_id	MSTRG.33430;
6	StringTie	transcrip	111477180	111480657	.	+	.	transcript_	MSTRG.33459.1	gene_id	MSTRG.33459;
6	StringTie	exon	111477180	111477326	.	+	.	transcript_	MSTRG.33459.1	gene_id	MSTRG.33459;
6	StringTie	exon	111479482	111480657	.	+	.	transcript_	MSTRG.33459.1	gene_id	MSTRG.33459;
6	StringTie	transcrip	135367993	135376194	.	+	.	transcript_	MSTRG.33632.2	gene_id	MSTRG.33632;
6	StringTie	exon	135367993	135372124	.	+	.	transcript_	MSTRG.33632.2	gene_id	MSTRG.33632;
6	StringTie	exon	135375059	135376194	.	+	.	transcript_	MSTRG.33632.2	gene_id	MSTRG.33632;
6	StringTie	transcrip	142588990	142592375	.	+	.	transcript_	MSTRG.33708.1	gene_id	MSTRG.33708;
6	StringTie	exon	142588990	142589008	.	+	.	transcript_	MSTRG.33708.1	gene_id	MSTRG.33708;
6	StringTie	exon	142590997	142592375	.	+	.	transcript_	MSTRG.33708.1	gene_id	MSTRG.33708;
6	StringTie	transcrip	146052103	146086714	.	+	.	transcript_	MSTRG.33748.1	gene_id	MSTRG.33748;
6	StringTie	exon	146052103	146052402	.	+	.	transcript_	MSTRG.33748.1	gene_id	MSTRG.33748;
6	StringTie	exon	146085381	146085474	.	+	.	transcript_	MSTRG.33748.1	gene_id	MSTRG.33748;
6	StringTie	exon	146086689	146086714	.	+	.	transcript_	MSTRG.33748.1	gene_id	MSTRG.33748;
6	StringTie	transcrip	146142447	146157227	.	+	.	transcript_	MSTRG.33783.1	gene_id	MSTRG.33783;
6	StringTie	exon	146142447	146143109	.	+	.	transcript_	MSTRG.33783.1	gene_id	MSTRG.33783;
6	StringTie	exon	146150171	146150281	.	+	.	transcript_	MSTRG.33783.1	gene_id	MSTRG.33783;
6	StringTie	exon	146157101	146157227	.	+	.	transcript_	MSTRG.33783.1	gene_id	MSTRG.33783;
6	StringTie	transcrip	165869239	165870714	.	+	.	transcript_	MSTRG.34114.1	gene_id	MSTRG.34114;
6	StringTie	exon	165869239	165869381	.	+	.	transcript_	MSTRG.34114.1	gene_id	MSTRG.34114;
6	StringTie	exon	165870449	165870714	.	+	.	transcript_	MSTRG.34114.1	gene_id	MSTRG.34114;
6	StringTie	transcrip	168637454	168649429	.	+	.	transcript_	MSTRG.34195.1	gene_id	MSTRG.34195;
6	StringTie	exon	168637454	168637641	.	+	.	transcript_	MSTRG.34195.1	gene_id	MSTRG.34195;
6	StringTie	exon	168642529	168642628	.	+	.	transcript_	MSTRG.34195.1	gene_id	MSTRG.34195;

6	StringTie	exon	168647465	168647645	.	+	.	transcript_	MSTRG.34195.:	gene_id	MSTRG.34195;
6	StringTie	exon	168648369	168649429	.	+	.	transcript_	MSTRG.34195.:	gene_id	MSTRG.34195;
6	StringTie	transcrip	168642285	168678887	.	+	.	transcript_	MSTRG.34197.:	gene_id	MSTRG.34197;
6	StringTie	exon	168642285	168642317	.	+	.	transcript_	MSTRG.34197.:	gene_id	MSTRG.34197;
6	StringTie	exon	168677970	168678887	.	+	.	transcript_	MSTRG.34197.:	gene_id	MSTRG.34197;
6	StringTie	transcrip	170485847	170492167	.	+	.	transcript_	MSTRG.34238.:	gene_id	MSTRG.34238;
6	StringTie	exon	170485847	170485879	.	+	.	transcript_	MSTRG.34238.:	gene_id	MSTRG.34238;
6	StringTie	exon	170491913	170492167	.	+	.	transcript_	MSTRG.34238.:	gene_id	MSTRG.34238;
6	StringTie	transcrip	170486282	170492188	.	+	.	transcript_	MSTRG.34238.:	gene_id	MSTRG.34238;
6	StringTie	exon	170486282	170486568	.	+	.	transcript_	MSTRG.34238.:	gene_id	MSTRG.34238;
6	StringTie	exon	170491913	170492188	.	+	.	transcript_	MSTRG.34238.:	gene_id	MSTRG.34238;
6	StringTie	transcrip	170615301	170637300	.	+	.	transcript_	MSTRG.34245.:	gene_id	MSTRG.34245;
6	StringTie	exon	170615301	170615446	.	+	.	transcript_	MSTRG.34245.:	gene_id	MSTRG.34245;
6	StringTie	exon	170620189	170620312	.	+	.	transcript_	MSTRG.34245.:	gene_id	MSTRG.34245;
6	StringTie	exon	170637004	170637300	.	+	.	transcript_	MSTRG.34245.:	gene_id	MSTRG.34245;
6	StringTie	transcrip	248014	253049	.	-	.	transcript_	MSTRG.30708.:	gene_id	MSTRG.30708;
6	StringTie	exon	248014	248320	.	-	.	transcript_	MSTRG.30708.:	gene_id	MSTRG.30708;
6	StringTie	exon	252560	253049	.	-	.	transcript_	MSTRG.30708.:	gene_id	MSTRG.30708;
6	StringTie	transcrip	3780080	3786258	.	-	.	transcript_	MSTRG.30789.(	gene_id	MSTRG.30789;
6	StringTie	exon	3780080	3780413	.	-	.	transcript_	MSTRG.30789.(	gene_id	MSTRG.30789;
6	StringTie	exon	3783793	3784066	.	-	.	transcript_	MSTRG.30789.(	gene_id	MSTRG.30789;
6	StringTie	exon	3785767	3786258	.	-	.	transcript_	MSTRG.30789.(	gene_id	MSTRG.30789;
6	StringTie	transcrip	3780184	3786266	.	-	.	transcript_	MSTRG.30789.'	gene_id	MSTRG.30789;
6	StringTie	exon	3780184	3784066	.	-	.	transcript_	MSTRG.30789.'	gene_id	MSTRG.30789;
6	StringTie	exon	3785767	3786266	.	-	.	transcript_	MSTRG.30789.'	gene_id	MSTRG.30789;
6	StringTie	transcrip	8463710	8466681	.	-	.	transcript_	MSTRG.30882.:	gene_id	MSTRG.30882;
6	StringTie	exon	8463710	8466179	.	-	.	transcript_	MSTRG.30882.:	gene_id	MSTRG.30882;
6	StringTie	exon	8466369	8466681	.	-	.	transcript_	MSTRG.30882.:	gene_id	MSTRG.30882;
6	StringTie	transcrip	12092860	12098236	.	-	.	transcript_	MSTRG.30925.:	gene_id	MSTRG.30925;
6	StringTie	exon	12092860	12092893	.	-	.	transcript_	MSTRG.30925.:	gene_id	MSTRG.30925;
6	StringTie	exon	12097527	12098236	.	-	.	transcript_	MSTRG.30925.:	gene_id	MSTRG.30925;
6	StringTie	transcrip	12096289	12102756	.	-	.	transcript_	MSTRG.30926.:	gene_id	MSTRG.30926;
6	StringTie	exon	12096289	12097190	.	-	.	transcript_	MSTRG.30926.:	gene_id	MSTRG.30926;
6	StringTie	exon	12102610	12102756	.	-	.	transcript_	MSTRG.30926.:	gene_id	MSTRG.30926;
6	StringTie	transcrip	12623499	12634090	.	-	.	transcript_	MSTRG.30942.:	gene_id	MSTRG.30942;

6	StringTie	exon	12623499	12623531	.	-	.	transcript_	MSTRG.30942.1	gene_id	MSTRG.30942;
6	StringTie	exon	12633828	12634090	.	-	.	transcript_	MSTRG.30942.1	gene_id	MSTRG.30942;
6	StringTie	transcrip	28746508	28799258	.	-	.	transcript_	MSTRG.31199.1	gene_id	MSTRG.31199;
6	StringTie	exon	28746508	28746862	.	-	.	transcript_	MSTRG.31199.1	gene_id	MSTRG.31199;
6	StringTie	exon	28798898	28799258	.	-	.	transcript_	MSTRG.31199.1	gene_id	MSTRG.31199;
6	StringTie	transcrip	30149327	30156048	.	-	.	transcript_	MSTRG.31220.1	gene_id	MSTRG.31220;
6	StringTie	exon	30149327	30150289	.	-	.	transcript_	MSTRG.31220.1	gene_id	MSTRG.31220;
6	StringTie	exon	30155851	30156048	.	-	.	transcript_	MSTRG.31220.1	gene_id	MSTRG.31220;
6	StringTie	transcrip	36293563	36300823	.	-	.	transcript_	MSTRG.31266.1	gene_id	MSTRG.31266;
6	StringTie	exon	36293563	36294940	.	-	.	transcript_	MSTRG.31266.1	gene_id	MSTRG.31266;
6	StringTie	exon	36300634	36300823	.	-	.	transcript_	MSTRG.31266.1	gene_id	MSTRG.31266;
6	StringTie	transcrip	36293563	36300845	.	-	.	transcript_	MSTRG.31266.2	gene_id	MSTRG.31266;
6	StringTie	exon	36293563	36294935	.	-	.	transcript_	MSTRG.31266.2	gene_id	MSTRG.31266;
6	StringTie	exon	36300634	36300845	.	-	.	transcript_	MSTRG.31266.2	gene_id	MSTRG.31266;
6	StringTie	transcrip	36294511	36300845	.	-	.	transcript_	MSTRG.31266.3	gene_id	MSTRG.31266;
6	StringTie	exon	36294511	36294935	.	-	.	transcript_	MSTRG.31266.3	gene_id	MSTRG.31266;
6	StringTie	exon	36297478	36297758	.	-	.	transcript_	MSTRG.31266.3	gene_id	MSTRG.31266;
6	StringTie	exon	36300634	36300845	.	-	.	transcript_	MSTRG.31266.3	gene_id	MSTRG.31266;
6	StringTie	transcrip	36294699	36300818	.	-	.	transcript_	MSTRG.31266.4	gene_id	MSTRG.31266;
6	StringTie	exon	36294699	36294940	.	-	.	transcript_	MSTRG.31266.4	gene_id	MSTRG.31266;
6	StringTie	exon	36297478	36297758	.	-	.	transcript_	MSTRG.31266.4	gene_id	MSTRG.31266;
6	StringTie	exon	36300634	36300818	.	-	.	transcript_	MSTRG.31266.4	gene_id	MSTRG.31266;
6	StringTie	transcrip	36299795	36300818	.	-	.	transcript_	MSTRG.31266.5	gene_id	MSTRG.31266;
6	StringTie	exon	36299795	36300183	.	-	.	transcript_	MSTRG.31266.5	gene_id	MSTRG.31266;
6	StringTie	exon	36300634	36300818	.	-	.	transcript_	MSTRG.31266.5	gene_id	MSTRG.31266;
6	StringTie	transcrip	43585470	43587302	.	-	.	transcript_	MSTRG.31372.1	gene_id	MSTRG.31372;
6	StringTie	exon	43585470	43585957	.	-	.	transcript_	MSTRG.31372.1	gene_id	MSTRG.31372;
6	StringTie	exon	43586859	43587302	.	-	.	transcript_	MSTRG.31372.1	gene_id	MSTRG.31372;
6	StringTie	transcrip	44127403	44128094	.	-	.	transcript_	MSTRG.31400.1	gene_id	MSTRG.31400;
6	StringTie	exon	44127403	44127742	.	-	.	transcript_	MSTRG.31400.1	gene_id	MSTRG.31400;
6	StringTie	exon	44128047	44128094	.	-	.	transcript_	MSTRG.31400.1	gene_id	MSTRG.31400;
6	StringTie	transcrip	44559414	44573768	.	-	.	transcript_	MSTRG.31415.1	gene_id	MSTRG.31415;
6	StringTie	exon	44559414	44560935	.	-	.	transcript_	MSTRG.31415.1	gene_id	MSTRG.31415;
6	StringTie	exon	44565370	44565452	.	-	.	transcript_	MSTRG.31415.1	gene_id	MSTRG.31415;
6	StringTie	exon	44572769	44573768	.	-	.	transcript_	MSTRG.31415.1	gene_id	MSTRG.31415;

6	StringTie	transcrip	45557940	45688288	.	-	.	transcript_	MSTRG.31466.1	gene_id	MSTRG.31466;
6	StringTie	exon	45557940	45558209	.	-	.	transcript_	MSTRG.31466.1	gene_id	MSTRG.31466;
6	StringTie	exon	45688210	45688288	.	-	.	transcript_	MSTRG.31466.1	gene_id	MSTRG.31466;
6	StringTie	transcrip	47215788	47217285	.	-	.	transcript_	MSTRG.31551.1	gene_id	MSTRG.31551;
6	StringTie	exon	47215788	47215806	.	-	.	transcript_	MSTRG.31551.1	gene_id	MSTRG.31551;
6	StringTie	exon	47216897	47217285	.	-	.	transcript_	MSTRG.31551.1	gene_id	MSTRG.31551;
6	StringTie	transcrip	47668839	47670903	.	-	.	transcript_	MSTRG.31579.1	gene_id	MSTRG.31579;
6	StringTie	exon	47668839	47669850	.	-	.	transcript_	MSTRG.31579.1	gene_id	MSTRG.31579;
6	StringTie	exon	47670157	47670903	.	-	.	transcript_	MSTRG.31579.1	gene_id	MSTRG.31579;
6	StringTie	transcrip	47671349	47672113	.	-	.	transcript_	MSTRG.31580.1	gene_id	MSTRG.31580;
6	StringTie	exon	47671349	47671788	.	-	.	transcript_	MSTRG.31580.1	gene_id	MSTRG.31580;
6	StringTie	exon	47672044	47672113	.	-	.	transcript_	MSTRG.31580.1	gene_id	MSTRG.31580;
6	StringTie	transcrip	49559105	49559480	.	-	.	transcript_	MSTRG.31722.1	gene_id	MSTRG.31722;
6	StringTie	exon	49559105	49559187	.	-	.	transcript_	MSTRG.31722.1	gene_id	MSTRG.31722;
6	StringTie	exon	49559208	49559480	.	-	.	transcript_	MSTRG.31722.1	gene_id	MSTRG.31722;
6	StringTie	transcrip	51178666	51180874	.	-	.	transcript_	MSTRG.31773.1	gene_id	MSTRG.31773;
6	StringTie	exon	51178666	51178758	.	-	.	transcript_	MSTRG.31773.1	gene_id	MSTRG.31773;
6	StringTie	exon	51179363	51179488	.	-	.	transcript_	MSTRG.31773.1	gene_id	MSTRG.31773;
6	StringTie	exon	51180782	51180874	.	-	.	transcript_	MSTRG.31773.1	gene_id	MSTRG.31773;
6	StringTie	transcrip	51309390	51309827	.	-	.	transcript_	MSTRG.31781.1	gene_id	MSTRG.31781;
6	StringTie	exon	51309390	51309500	.	-	.	transcript_	MSTRG.31781.1	gene_id	MSTRG.31781;
6	StringTie	exon	51309572	51309682	.	-	.	transcript_	MSTRG.31781.1	gene_id	MSTRG.31781;
6	StringTie	exon	51309717	51309827	.	-	.	transcript_	MSTRG.31781.1	gene_id	MSTRG.31781;
6	StringTie	transcrip	51310113	51310401	.	-	.	transcript_	MSTRG.31782.1	gene_id	MSTRG.31782;
6	StringTie	exon	51310113	51310222	.	-	.	transcript_	MSTRG.31782.1	gene_id	MSTRG.31782;
6	StringTie	exon	51310291	51310401	.	-	.	transcript_	MSTRG.31782.1	gene_id	MSTRG.31782;
6	StringTie	transcrip	54039916	54041637	.	-	.	transcript_	MSTRG.31933.1	gene_id	MSTRG.31933;
6	StringTie	exon	54039916	54040608	.	-	.	transcript_	MSTRG.31933.1	gene_id	MSTRG.31933;
6	StringTie	exon	54041208	54041637	.	-	.	transcript_	MSTRG.31933.1	gene_id	MSTRG.31933;
6	StringTie	transcrip	54677318	54678162	.	-	.	transcript_	MSTRG.31972.1	gene_id	MSTRG.31972;
6	StringTie	exon	54677318	54677804	.	-	.	transcript_	MSTRG.31972.1	gene_id	MSTRG.31972;
6	StringTie	exon	54677913	54678162	.	-	.	transcript_	MSTRG.31972.1	gene_id	MSTRG.31972;
6	StringTie	transcrip	58237382	58242308	.	-	.	transcript_	MSTRG.32144.1	gene_id	MSTRG.32144;
6	StringTie	exon	58237382	58238473	.	-	.	transcript_	MSTRG.32144.1	gene_id	MSTRG.32144;
6	StringTie	exon	58242245	58242308	.	-	.	transcript_	MSTRG.32144.1	gene_id	MSTRG.32144;

6	StringTie	transcrip	58237404	58242284	.	-	.	transcript_	MSTRG.32144.2	gene_id	MSTRG.32144;
6	StringTie	exon	58237404	58238473	.	-	.	transcript_	MSTRG.32144.2	gene_id	MSTRG.32144;
6	StringTie	exon	58239305	58239562	.	-	.	transcript_	MSTRG.32144.2	gene_id	MSTRG.32144;
6	StringTie	exon	58241958	58242284	.	-	.	transcript_	MSTRG.32144.2	gene_id	MSTRG.32144;
6	StringTie	transcrip	58237882	58242308	.	-	.	transcript_	MSTRG.32144.3	gene_id	MSTRG.32144;
6	StringTie	exon	58237882	58238473	.	-	.	transcript_	MSTRG.32144.3	gene_id	MSTRG.32144;
6	StringTie	exon	58239305	58239562	.	-	.	transcript_	MSTRG.32144.3	gene_id	MSTRG.32144;
6	StringTie	exon	58242245	58242308	.	-	.	transcript_	MSTRG.32144.3	gene_id	MSTRG.32144;
6	StringTie	transcrip	62639677	62667578	.	-	.	transcript_	MSTRG.32395.1	gene_id	MSTRG.32395;
6	StringTie	exon	62639677	62639738	.	-	.	transcript_	MSTRG.32395.1	gene_id	MSTRG.32395;
6	StringTie	exon	62656598	62656817	.	-	.	transcript_	MSTRG.32395.1	gene_id	MSTRG.32395;
6	StringTie	exon	62657966	62658084	.	-	.	transcript_	MSTRG.32395.1	gene_id	MSTRG.32395;
6	StringTie	exon	62660016	62660163	.	-	.	transcript_	MSTRG.32395.1	gene_id	MSTRG.32395;
6	StringTie	exon	62667347	62667578	.	-	.	transcript_	MSTRG.32395.1	gene_id	MSTRG.32395;
6	StringTie	transcrip	63686247	63687882	.	-	.	transcript_	MSTRG.32356.1	gene_id	MSTRG.32356;
6	StringTie	exon	63686247	63686749	.	-	.	transcript_	MSTRG.32356.1	gene_id	MSTRG.32356;
6	StringTie	exon	63687820	63687882	.	-	.	transcript_	MSTRG.32356.1	gene_id	MSTRG.32356;
6	StringTie	transcrip	71153018	71161066	.	-	.	transcript_	MSTRG.32586.2	gene_id	MSTRG.32586;
6	StringTie	exon	71153018	71157539	.	-	.	transcript_	MSTRG.32586.2	gene_id	MSTRG.32586;
6	StringTie	exon	71160571	71161066	.	-	.	transcript_	MSTRG.32586.2	gene_id	MSTRG.32586;
6	StringTie	transcrip	71155565	71160819	.	-	.	transcript_	MSTRG.32586.3	gene_id	MSTRG.32586;
6	StringTie	exon	71155565	71156023	.	-	.	transcript_	MSTRG.32586.3	gene_id	MSTRG.32586;
6	StringTie	exon	71156113	71157539	.	-	.	transcript_	MSTRG.32586.3	gene_id	MSTRG.32586;
6	StringTie	exon	71160571	71160819	.	-	.	transcript_	MSTRG.32586.3	gene_id	MSTRG.32586;
6	StringTie	transcrip	80912669	80978876	.	-	.	transcript_	MSTRG.32774.1	gene_id	MSTRG.32774;
6	StringTie	exon	80912669	80912721	.	-	.	transcript_	MSTRG.32774.1	gene_id	MSTRG.32774;
6	StringTie	exon	80978094	80978876	.	-	.	transcript_	MSTRG.32774.1	gene_id	MSTRG.32774;
6	StringTie	transcrip	81192692	81194285	.	-	.	transcript_	MSTRG.32772.1	gene_id	MSTRG.32772;
6	StringTie	exon	81192692	81192783	.	-	.	transcript_	MSTRG.32772.1	gene_id	MSTRG.32772;
6	StringTie	exon	81193846	81194285	.	-	.	transcript_	MSTRG.32772.1	gene_id	MSTRG.32772;
6	StringTie	transcrip	81772067	81798959	.	-	.	transcript_	MSTRG.32817.1	gene_id	MSTRG.32817;
6	StringTie	exon	81772067	81772354	.	-	.	transcript_	MSTRG.32817.1	gene_id	MSTRG.32817;
6	StringTie	exon	81798701	81798959	.	-	.	transcript_	MSTRG.32817.1	gene_id	MSTRG.32817;
6	StringTie	transcrip	81796223	81798974	.	-	.	transcript_	MSTRG.32817.2	gene_id	MSTRG.32817;
6	StringTie	exon	81796223	81797174	.	-	.	transcript_	MSTRG.32817.2	gene_id	MSTRG.32817;

6	StringTie	exon	81798701	81798974	.	-	.	transcript_	MSTRG.32817.2	gene_id	MSTRG.32817;
6	StringTie	transcrip	82858753	82861565	.	-	.	transcript_	MSTRG.32849.6	gene_id	MSTRG.32849;
6	StringTie	exon	82858753	82859597	.	-	.	transcript_	MSTRG.32849.6	gene_id	MSTRG.32849;
6	StringTie	exon	82860654	82860856	.	-	.	transcript_	MSTRG.32849.6	gene_id	MSTRG.32849;
6	StringTie	exon	82861441	82861565	.	-	.	transcript_	MSTRG.32849.6	gene_id	MSTRG.32849;
6	StringTie	transcrip	85593754	85597213	.	-	.	transcript_	MSTRG.32955.1	gene_id	MSTRG.32955;
6	StringTie	exon	85593754	85593864	.	-	.	transcript_	MSTRG.32955.1	gene_id	MSTRG.32955;
6	StringTie	exon	85594326	85594377	.	-	.	transcript_	MSTRG.32955.1	gene_id	MSTRG.32955;
6	StringTie	exon	85594728	85594814	.	-	.	transcript_	MSTRG.32955.1	gene_id	MSTRG.32955;
6	StringTie	exon	85595882	85595979	.	-	.	transcript_	MSTRG.32955.1	gene_id	MSTRG.32955;
6	StringTie	exon	85596470	85596517	.	-	.	transcript_	MSTRG.32955.1	gene_id	MSTRG.32955;
6	StringTie	exon	85596926	85597213	.	-	.	transcript_	MSTRG.32955.1	gene_id	MSTRG.32955;
6	StringTie	transcrip	89320251	89328552	.	-	.	transcript_	MSTRG.33040.1	gene_id	MSTRG.33040;
6	StringTie	exon	89320251	89320277	.	-	.	transcript_	MSTRG.33040.1	gene_id	MSTRG.33040;
6	StringTie	exon	89327769	89328132	.	-	.	transcript_	MSTRG.33040.1	gene_id	MSTRG.33040;
6	StringTie	exon	89328394	89328552	.	-	.	transcript_	MSTRG.33040.1	gene_id	MSTRG.33040;
6	StringTie	transcrip	95939214	95941487	.	-	.	transcript_	MSTRG.33176.1	gene_id	MSTRG.33176;
6	StringTie	exon	95939214	95940869	.	-	.	transcript_	MSTRG.33176.1	gene_id	MSTRG.33176;
6	StringTie	exon	95941282	95941487	.	-	.	transcript_	MSTRG.33176.1	gene_id	MSTRG.33176;
6	StringTie	transcrip	100190463	100204085	.	-	.	transcript_	MSTRG.33274.1	gene_id	MSTRG.33274;
6	StringTie	exon	100190463	100190885	.	-	.	transcript_	MSTRG.33274.1	gene_id	MSTRG.33274;
6	StringTie	exon	100201926	100202074	.	-	.	transcript_	MSTRG.33274.1	gene_id	MSTRG.33274;
6	StringTie	exon	100202561	100202696	.	-	.	transcript_	MSTRG.33274.1	gene_id	MSTRG.33274;
6	StringTie	exon	100203977	100204085	.	-	.	transcript_	MSTRG.33274.1	gene_id	MSTRG.33274;
6	StringTie	transcrip	106635947	106650737	.	-	.	transcript_	MSTRG.33356.1	gene_id	MSTRG.33356;
6	StringTie	exon	106635947	106636344	.	-	.	transcript_	MSTRG.33356.1	gene_id	MSTRG.33356;
6	StringTie	exon	106650661	106650737	.	-	.	transcript_	MSTRG.33356.1	gene_id	MSTRG.33356;
6	StringTie	transcrip	115834057	115857831	.	-	.	transcript_	MSTRG.33479.3	gene_id	MSTRG.33479;
6	StringTie	exon	115834057	115834283	.	-	.	transcript_	MSTRG.33479.3	gene_id	MSTRG.33479;
6	StringTie	exon	115834768	115834824	.	-	.	transcript_	MSTRG.33479.3	gene_id	MSTRG.33479;
6	StringTie	exon	115834969	115835074	.	-	.	transcript_	MSTRG.33479.3	gene_id	MSTRG.33479;
6	StringTie	exon	115838276	115838498	.	-	.	transcript_	MSTRG.33479.3	gene_id	MSTRG.33479;
6	StringTie	exon	115857749	115857831	.	-	.	transcript_	MSTRG.33479.3	gene_id	MSTRG.33479;
6	StringTie	transcrip	142163295	142371113	.	-	.	transcript_	MSTRG.33691.1	gene_id	MSTRG.33691;
6	StringTie	exon	142163295	142163468	.	-	.	transcript_	MSTRG.33691.1	gene_id	MSTRG.33691;

6	StringTie	exon	142271334	142271425	.	-	.	transcript_	MSTRG.33691.:	gene_id	MSTRG.33691;
6	StringTie	exon	142371079	142371113	.	-	.	transcript_	MSTRG.33691.:	gene_id	MSTRG.33691;
6	StringTie	transcrip	146101209	146142373	.	-	.	transcript_	MSTRG.33772.:	gene_id	MSTRG.33772;
6	StringTie	exon	146101209	146101223	.	-	.	transcript_	MSTRG.33772.:	gene_id	MSTRG.33772;
6	StringTie	exon	146142135	146142373	.	-	.	transcript_	MSTRG.33772.:	gene_id	MSTRG.33772;
6	StringTie	transcrip	156608053	156611358	.	-	.	transcript_	MSTRG.33951.:	gene_id	MSTRG.33951;
6	StringTie	exon	156608053	156608633	.	-	.	transcript_	MSTRG.33951.:	gene_id	MSTRG.33951;
6	StringTie	exon	156611258	156611358	.	-	.	transcript_	MSTRG.33951.:	gene_id	MSTRG.33951;
6	StringTie	transcrip	156983173	157197843	.	-	.	transcript_	MSTRG.33969.:	gene_id	MSTRG.33969;
6	StringTie	exon	156983173	156983218	.	-	.	transcript_	MSTRG.33969.:	gene_id	MSTRG.33969;
6	StringTie	exon	157101587	157101639	.	-	.	transcript_	MSTRG.33969.:	gene_id	MSTRG.33969;
6	StringTie	exon	157108634	157108736	.	-	.	transcript_	MSTRG.33969.:	gene_id	MSTRG.33969;
6	StringTie	exon	157197784	157197843	.	-	.	transcript_	MSTRG.33969.:	gene_id	MSTRG.33969;
6	StringTie	transcrip	165197130	165201719	.	-	.	transcript_	MSTRG.34095.:	gene_id	MSTRG.34095;
6	StringTie	exon	165197130	165197493	.	-	.	transcript_	MSTRG.34095.:	gene_id	MSTRG.34095;
6	StringTie	exon	165200905	165201719	.	-	.	transcript_	MSTRG.34095.:	gene_id	MSTRG.34095;
6	StringTie	transcrip	169216241	169221297	.	-	.	transcript_	MSTRG.34205.:	gene_id	MSTRG.34205;
6	StringTie	exon	169216241	169216681	.	-	.	transcript_	MSTRG.34205.:	gene_id	MSTRG.34205;
6	StringTie	exon	169220489	169221297	.	-	.	transcript_	MSTRG.34205.:	gene_id	MSTRG.34205;
7	StringTie	transcrip	951585	984061	.	+	.	transcript_	MSTRG.34259.:	gene_id	MSTRG.34259;
7	StringTie	exon	951585	951617	.	+	.	transcript_	MSTRG.34259.:	gene_id	MSTRG.34259;
7	StringTie	exon	952110	953440	.	+	.	transcript_	MSTRG.34259.:	gene_id	MSTRG.34259;
7	StringTie	exon	981411	984061	.	+	.	transcript_	MSTRG.34259.:	gene_id	MSTRG.34259;
7	StringTie	transcrip	1344223	1347684	.	+	.	transcript_	MSTRG.34252.:	gene_id	MSTRG.34252;
7	StringTie	exon	1344223	1344435	.	+	.	transcript_	MSTRG.34252.:	gene_id	MSTRG.34252;
7	StringTie	exon	1345121	1347684	.	+	.	transcript_	MSTRG.34252.:	gene_id	MSTRG.34252;
7	StringTie	transcrip	1431327	1432314	.	+	.	transcript_	MSTRG.34263.:	gene_id	MSTRG.34263;
7	StringTie	exon	1431327	1431552	.	+	.	transcript_	MSTRG.34263.:	gene_id	MSTRG.34263;
7	StringTie	exon	1432261	1432314	.	+	.	transcript_	MSTRG.34263.:	gene_id	MSTRG.34263;
7	StringTie	transcrip	2469276	2481600	.	+	.	transcript_	MSTRG.34281.:	gene_id	MSTRG.34281;
7	StringTie	exon	2469276	2469361	.	+	.	transcript_	MSTRG.34281.:	gene_id	MSTRG.34281;
7	StringTie	exon	2480912	2481600	.	+	.	transcript_	MSTRG.34281.:	gene_id	MSTRG.34281;
7	StringTie	transcrip	5853741	5868768	.	+	.	transcript_	MSTRG.34330.:	gene_id	MSTRG.34330;
7	StringTie	exon	5853741	5853975	.	+	.	transcript_	MSTRG.34330.:	gene_id	MSTRG.34330;
7	StringTie	exon	5868745	5868768	.	+	.	transcript_	MSTRG.34330.:	gene_id	MSTRG.34330;

7	StringTie	transcrip	5877361	5882073	.	+	.	transcript_	MSTRG.34335.1	gene_id	MSTRG.34335;
7	StringTie	exon	5877361	5877977	.	+	.	transcript_	MSTRG.34335.1	gene_id	MSTRG.34335;
7	StringTie	exon	5882017	5882073	.	+	.	transcript_	MSTRG.34335.1	gene_id	MSTRG.34335;
7	StringTie	transcrip	10925109	10930946	.	+	.	transcript_	MSTRG.34426.1	gene_id	MSTRG.34426;
7	StringTie	exon	10925109	10925293	.	+	.	transcript_	MSTRG.34426.1	gene_id	MSTRG.34426;
7	StringTie	exon	10927381	10930946	.	+	.	transcript_	MSTRG.34426.1	gene_id	MSTRG.34426;
7	StringTie	transcrip	11245041	11250429	.	+	.	transcript_	MSTRG.34428.1	gene_id	MSTRG.34428;
7	StringTie	exon	11245041	11245103	.	+	.	transcript_	MSTRG.34428.1	gene_id	MSTRG.34428;
7	StringTie	exon	11249283	11249397	.	+	.	transcript_	MSTRG.34428.1	gene_id	MSTRG.34428;
7	StringTie	exon	11250094	11250429	.	+	.	transcript_	MSTRG.34428.1	gene_id	MSTRG.34428;
7	StringTie	transcrip	12733109	12757663	.	+	.	transcript_	MSTRG.34455.1	gene_id	MSTRG.34455;
7	StringTie	exon	12733109	12733153	.	+	.	transcript_	MSTRG.34455.1	gene_id	MSTRG.34455;
7	StringTie	exon	12733664	12733978	.	+	.	transcript_	MSTRG.34455.1	gene_id	MSTRG.34455;
7	StringTie	exon	12743552	12743662	.	+	.	transcript_	MSTRG.34455.1	gene_id	MSTRG.34455;
7	StringTie	exon	12743786	12743867	.	+	.	transcript_	MSTRG.34455.1	gene_id	MSTRG.34455;
7	StringTie	exon	12746055	12746189	.	+	.	transcript_	MSTRG.34455.1	gene_id	MSTRG.34455;
7	StringTie	exon	12757311	12757471	.	+	.	transcript_	MSTRG.34455.1	gene_id	MSTRG.34455;
7	StringTie	exon	12757641	12757663	.	+	.	transcript_	MSTRG.34455.1	gene_id	MSTRG.34455;
7	StringTie	transcrip	12733858	12735290	.	+	.	transcript_	MSTRG.34455.2	gene_id	MSTRG.34455;
7	StringTie	exon	12733858	12733978	.	+	.	transcript_	MSTRG.34455.2	gene_id	MSTRG.34455;
7	StringTie	exon	12734804	12735290	.	+	.	transcript_	MSTRG.34455.2	gene_id	MSTRG.34455;
7	StringTie	transcrip	13971357	13971699	.	+	.	transcript_	MSTRG.34499.1	gene_id	MSTRG.34499;
7	StringTie	exon	13971357	13971395	.	+	.	transcript_	MSTRG.34499.1	gene_id	MSTRG.34499;
7	StringTie	exon	13971487	13971699	.	+	.	transcript_	MSTRG.34499.1	gene_id	MSTRG.34499;
7	StringTie	transcrip	21559159	21562817	.	+	.	transcript_	MSTRG.34746.1	gene_id	MSTRG.34746;
7	StringTie	exon	21559159	21559347	.	+	.	transcript_	MSTRG.34746.1	gene_id	MSTRG.34746;
7	StringTie	exon	21561413	21561513	.	+	.	transcript_	MSTRG.34746.1	gene_id	MSTRG.34746;
7	StringTie	exon	21562768	21562817	.	+	.	transcript_	MSTRG.34746.1	gene_id	MSTRG.34746;
7	StringTie	transcrip	23234183	23234977	.	+	.	transcript_	MSTRG.34812.1	gene_id	MSTRG.34812;
7	StringTie	exon	23234183	23234289	.	+	.	transcript_	MSTRG.34812.1	gene_id	MSTRG.34812;
7	StringTie	exon	23234872	23234977	.	+	.	transcript_	MSTRG.34812.1	gene_id	MSTRG.34812;
7	StringTie	transcrip	24097492	24098545	.	+	.	transcript_	MSTRG.34932.1	gene_id	MSTRG.34932;
7	StringTie	exon	24097492	24097534	.	+	.	transcript_	MSTRG.34932.1	gene_id	MSTRG.34932;
7	StringTie	exon	24098287	24098545	.	+	.	transcript_	MSTRG.34932.1	gene_id	MSTRG.34932;
7	StringTie	transcrip	24101949	24102935	.	+	.	transcript_	MSTRG.34933.1	gene_id	MSTRG.34933;

7	StringTie	exon	24101949	24101990	.	+	.	transcript_	MSTRG.34933.1	gene_id	MSTRG.34933;
7	StringTie	exon	24102014	24102072	.	+	.	transcript_	MSTRG.34933.1	gene_id	MSTRG.34933;
7	StringTie	exon	24102826	24102935	.	+	.	transcript_	MSTRG.34933.1	gene_id	MSTRG.34933;
7	StringTie	transcrip	24888270	24907578	.	+	.	transcript_	MSTRG.34916.1	gene_id	MSTRG.34916;
7	StringTie	exon	24888270	24888293	.	+	.	transcript_	MSTRG.34916.1	gene_id	MSTRG.34916;
7	StringTie	exon	24907307	24907578	.	+	.	transcript_	MSTRG.34916.1	gene_id	MSTRG.34916;
7	StringTie	transcrip	26461728	26477596	.	+	.	transcript_	MSTRG.34959.1	gene_id	MSTRG.34959;
7	StringTie	exon	26461728	26462166	.	+	.	transcript_	MSTRG.34959.1	gene_id	MSTRG.34959;
7	StringTie	exon	26477116	26477596	.	+	.	transcript_	MSTRG.34959.1	gene_id	MSTRG.34959;
7	StringTie	transcrip	26600994	26601450	.	+	.	transcript_	MSTRG.34971.1	gene_id	MSTRG.34971;
7	StringTie	exon	26600994	26601257	.	+	.	transcript_	MSTRG.34971.1	gene_id	MSTRG.34971;
7	StringTie	exon	26601292	26601450	.	+	.	transcript_	MSTRG.34971.1	gene_id	MSTRG.34971;
7	StringTie	transcrip	30666965	30668334	.	+	.	transcript_	MSTRG.35020.1	gene_id	MSTRG.35020;
7	StringTie	exon	30666965	30667075	.	+	.	transcript_	MSTRG.35020.1	gene_id	MSTRG.35020;
7	StringTie	exon	30667442	30668334	.	+	.	transcript_	MSTRG.35020.1	gene_id	MSTRG.35020;
7	StringTie	transcrip	31353928	31357305	.	+	.	transcript_	MSTRG.35051.1	gene_id	MSTRG.35051;
7	StringTie	exon	31353928	31354093	.	+	.	transcript_	MSTRG.35051.1	gene_id	MSTRG.35051;
7	StringTie	exon	31357058	31357305	.	+	.	transcript_	MSTRG.35051.1	gene_id	MSTRG.35051;
7	StringTie	transcrip	32738838	32756093	.	+	.	transcript_	MSTRG.35093.1	gene_id	MSTRG.35093;
7	StringTie	exon	32738838	32739028	.	+	.	transcript_	MSTRG.35093.1	gene_id	MSTRG.35093;
7	StringTie	exon	32748002	32748103	.	+	.	transcript_	MSTRG.35093.1	gene_id	MSTRG.35093;
7	StringTie	exon	32750281	32750388	.	+	.	transcript_	MSTRG.35093.1	gene_id	MSTRG.35093;
7	StringTie	exon	32752277	32752399	.	+	.	transcript_	MSTRG.35093.1	gene_id	MSTRG.35093;
7	StringTie	exon	32755102	32756093	.	+	.	transcript_	MSTRG.35093.1	gene_id	MSTRG.35093;
7	StringTie	transcrip	32738852	32756539	.	+	.	transcript_	MSTRG.35093.2	gene_id	MSTRG.35093;
7	StringTie	exon	32738852	32739028	.	+	.	transcript_	MSTRG.35093.2	gene_id	MSTRG.35093;
7	StringTie	exon	32750281	32750388	.	+	.	transcript_	MSTRG.35093.2	gene_id	MSTRG.35093;
7	StringTie	exon	32752277	32752399	.	+	.	transcript_	MSTRG.35093.2	gene_id	MSTRG.35093;
7	StringTie	exon	32755102	32756539	.	+	.	transcript_	MSTRG.35093.2	gene_id	MSTRG.35093;
7	StringTie	transcrip	34123306	34134001	.	+	.	transcript_	MSTRG.35117.1	gene_id	MSTRG.35117;
7	StringTie	exon	34123306	34123476	.	+	.	transcript_	MSTRG.35117.1	gene_id	MSTRG.35117;
7	StringTie	exon	34125200	34125310	.	+	.	transcript_	MSTRG.35117.1	gene_id	MSTRG.35117;
7	StringTie	exon	34126262	34126336	.	+	.	transcript_	MSTRG.35117.1	gene_id	MSTRG.35117;
7	StringTie	exon	34129058	34129198	.	+	.	transcript_	MSTRG.35117.1	gene_id	MSTRG.35117;
7	StringTie	exon	34130521	34134001	.	+	.	transcript_	MSTRG.35117.1	gene_id	MSTRG.35117;

7	StringTie	transcrip	34123384	34130768	.	+	.	transcript_	MSTRG.35117.2	gene_id	MSTRG.35117;
7	StringTie	exon	34123384	34123476	.	+	.	transcript_	MSTRG.35117.2	gene_id	MSTRG.35117;
7	StringTie	exon	34125200	34125310	.	+	.	transcript_	MSTRG.35117.2	gene_id	MSTRG.35117;
7	StringTie	exon	34126262	34126336	.	+	.	transcript_	MSTRG.35117.2	gene_id	MSTRG.35117;
7	StringTie	exon	34129058	34129198	.	+	.	transcript_	MSTRG.35117.2	gene_id	MSTRG.35117;
7	StringTie	exon	34130517	34130768	.	+	.	transcript_	MSTRG.35117.2	gene_id	MSTRG.35117;
7	StringTie	transcrip	34555354	34596028	.	+	.	transcript_	MSTRG.35111.1	gene_id	MSTRG.35111;
7	StringTie	exon	34555354	34555398	.	+	.	transcript_	MSTRG.35111.1	gene_id	MSTRG.35111;
7	StringTie	exon	34575271	34575436	.	+	.	transcript_	MSTRG.35111.1	gene_id	MSTRG.35111;
7	StringTie	exon	34595626	34596028	.	+	.	transcript_	MSTRG.35111.1	gene_id	MSTRG.35111;
7	StringTie	transcrip	34709396	34721387	.	+	.	transcript_	MSTRG.35125.1	gene_id	MSTRG.35125;
7	StringTie	exon	34709396	34710732	.	+	.	transcript_	MSTRG.35125.1	gene_id	MSTRG.35125;
7	StringTie	exon	34720729	34721387	.	+	.	transcript_	MSTRG.35125.1	gene_id	MSTRG.35125;
7	StringTie	transcrip	34710239	34714909	.	+	.	transcript_	MSTRG.35125.2	gene_id	MSTRG.35125;
7	StringTie	exon	34710239	34710732	.	+	.	transcript_	MSTRG.35125.2	gene_id	MSTRG.35125;
7	StringTie	exon	34714282	34714909	.	+	.	transcript_	MSTRG.35125.2	gene_id	MSTRG.35125;
7	StringTie	transcrip	37884230	37886935	.	+	.	transcript_	MSTRG.35208.1	gene_id	MSTRG.35208;
7	StringTie	exon	37884230	37885407	.	+	.	transcript_	MSTRG.35208.1	gene_id	MSTRG.35208;
7	StringTie	exon	37885430	37886935	.	+	.	transcript_	MSTRG.35208.1	gene_id	MSTRG.35208;
7	StringTie	transcrip	37887270	37891460	.	+	.	transcript_	MSTRG.35209.1	gene_id	MSTRG.35209;
7	StringTie	exon	37887270	37887607	.	+	.	transcript_	MSTRG.35209.1	gene_id	MSTRG.35209;
7	StringTie	exon	37891367	37891460	.	+	.	transcript_	MSTRG.35209.1	gene_id	MSTRG.35209;
7	StringTie	transcrip	38739175	38740329	.	+	.	transcript_	MSTRG.35229.1	gene_id	MSTRG.35229;
7	StringTie	exon	38739175	38739375	.	+	.	transcript_	MSTRG.35229.1	gene_id	MSTRG.35229;
7	StringTie	exon	38739998	38740329	.	+	.	transcript_	MSTRG.35229.1	gene_id	MSTRG.35229;
7	StringTie	transcrip	45967095	45976289	.	+	.	transcript_	MSTRG.35326.2	gene_id	MSTRG.35326;
7	StringTie	exon	45967095	45967928	.	+	.	transcript_	MSTRG.35326.2	gene_id	MSTRG.35326;
7	StringTie	exon	45974123	45976289	.	+	.	transcript_	MSTRG.35326.2	gene_id	MSTRG.35326;
7	StringTie	transcrip	52202996	52206437	.	+	.	transcript_	MSTRG.35465.1	gene_id	MSTRG.35465;
7	StringTie	exon	52202996	52203203	.	+	.	transcript_	MSTRG.35465.1	gene_id	MSTRG.35465;
7	StringTie	exon	52203308	52203368	.	+	.	transcript_	MSTRG.35465.1	gene_id	MSTRG.35465;
7	StringTie	exon	52206298	52206437	.	+	.	transcript_	MSTRG.35465.1	gene_id	MSTRG.35465;
7	StringTie	transcrip	53859216	53861370	.	+	.	transcript_	MSTRG.35531.1	gene_id	MSTRG.35531;
7	StringTie	exon	53859216	53859278	.	+	.	transcript_	MSTRG.35531.1	gene_id	MSTRG.35531;
7	StringTie	exon	53861190	53861370	.	+	.	transcript_	MSTRG.35531.1	gene_id	MSTRG.35531;

7	StringTie	transcrip	56378612	56381253	.	+	.	transcript_	MSTRG.35628.8	gene_id	MSTRG.35628;
7	StringTie	exon	56378612	56378667	.	+	.	transcript_	MSTRG.35628.8	gene_id	MSTRG.35628;
7	StringTie	exon	56380920	56381253	.	+	.	transcript_	MSTRG.35628.8	gene_id	MSTRG.35628;
7	StringTie	transcrip	58268997	58278781	.	+	.	transcript_	MSTRG.35670.1	gene_id	MSTRG.35670;
7	StringTie	exon	58268997	58269103	.	+	.	transcript_	MSTRG.35670.1	gene_id	MSTRG.35670;
7	StringTie	exon	58269747	58269869	.	+	.	transcript_	MSTRG.35670.1	gene_id	MSTRG.35670;
7	StringTie	exon	58278351	58278575	.	+	.	transcript_	MSTRG.35670.1	gene_id	MSTRG.35670;
7	StringTie	exon	58278707	58278781	.	+	.	transcript_	MSTRG.35670.1	gene_id	MSTRG.35670;
7	StringTie	transcrip	58269007	58278817	.	+	.	transcript_	MSTRG.35670.2	gene_id	MSTRG.35670;
7	StringTie	exon	58269007	58269103	.	+	.	transcript_	MSTRG.35670.2	gene_id	MSTRG.35670;
7	StringTie	exon	58269747	58269869	.	+	.	transcript_	MSTRG.35670.2	gene_id	MSTRG.35670;
7	StringTie	exon	58278351	58278562	.	+	.	transcript_	MSTRG.35670.2	gene_id	MSTRG.35670;
7	StringTie	exon	58278707	58278817	.	+	.	transcript_	MSTRG.35670.2	gene_id	MSTRG.35670;
7	StringTie	transcrip	58279025	58279649	.	+	.	transcript_	MSTRG.35672.1	gene_id	MSTRG.35672;
7	StringTie	exon	58279025	58279070	.	+	.	transcript_	MSTRG.35672.1	gene_id	MSTRG.35672;
7	StringTie	exon	58279386	58279649	.	+	.	transcript_	MSTRG.35672.1	gene_id	MSTRG.35672;
7	StringTie	transcrip	59643260	59644955	.	+	.	transcript_	MSTRG.35710.1	gene_id	MSTRG.35710;
7	StringTie	exon	59643260	59643678	.	+	.	transcript_	MSTRG.35710.1	gene_id	MSTRG.35710;
7	StringTie	exon	59644698	59644955	.	+	.	transcript_	MSTRG.35710.1	gene_id	MSTRG.35710;
7	StringTie	transcrip	68726185	68747586	.	+	.	transcript_	MSTRG.35868.1	gene_id	MSTRG.35868;
7	StringTie	exon	68726185	68726624	.	+	.	transcript_	MSTRG.35868.1	gene_id	MSTRG.35868;
7	StringTie	exon	68746997	68747586	.	+	.	transcript_	MSTRG.35868.1	gene_id	MSTRG.35868;
7	StringTie	transcrip	73163587	73727616	.	+	.	transcript_	MSTRG.35902.1	gene_id	MSTRG.35902;
7	StringTie	exon	73163587	73163844	.	+	.	transcript_	MSTRG.35902.1	gene_id	MSTRG.35902;
7	StringTie	exon	73540139	73540264	.	+	.	transcript_	MSTRG.35902.1	gene_id	MSTRG.35902;
7	StringTie	exon	73724595	73724689	.	+	.	transcript_	MSTRG.35902.1	gene_id	MSTRG.35902;
7	StringTie	exon	73727551	73727616	.	+	.	transcript_	MSTRG.35902.1	gene_id	MSTRG.35902;
7	StringTie	transcrip	73727770	73729049	.	+	.	transcript_	MSTRG.35905.1	gene_id	MSTRG.35905;
7	StringTie	exon	73727770	73727988	.	+	.	transcript_	MSTRG.35905.1	gene_id	MSTRG.35905;
7	StringTie	exon	73728985	73729049	.	+	.	transcript_	MSTRG.35905.1	gene_id	MSTRG.35905;
7	StringTie	transcrip	80413482	80417358	.	+	.	transcript_	MSTRG.36127.1	gene_id	MSTRG.36127;
7	StringTie	exon	80413482	80413801	.	+	.	transcript_	MSTRG.36127.1	gene_id	MSTRG.36127;
7	StringTie	exon	80415099	80417358	.	+	.	transcript_	MSTRG.36127.1	gene_id	MSTRG.36127;
7	StringTie	transcrip	83126875	83128864	.	+	.	transcript_	MSTRG.36124.1	gene_id	MSTRG.36124;
7	StringTie	exon	83126875	83128406	.	+	.	transcript_	MSTRG.36124.1	gene_id	MSTRG.36124;

7	StringTie	exon	83128774	83128864	.	+	.	transcript_	MSTRG.36124.1	gene_id	MSTRG.36124;
7	StringTie	transcrip	83550072	83570760	.	+	.	transcript_	MSTRG.36149.1	gene_id	MSTRG.36149;
7	StringTie	exon	83550072	83550147	.	+	.	transcript_	MSTRG.36149.1	gene_id	MSTRG.36149;
7	StringTie	exon	83569279	83570760	.	+	.	transcript_	MSTRG.36149.1	gene_id	MSTRG.36149;
7	StringTie	transcrip	86924134	86925569	.	+	.	transcript_	MSTRG.36205.1	gene_id	MSTRG.36205;
7	StringTie	exon	86924134	86924299	.	+	.	transcript_	MSTRG.36205.1	gene_id	MSTRG.36205;
7	StringTie	exon	86925256	86925569	.	+	.	transcript_	MSTRG.36205.1	gene_id	MSTRG.36205;
7	StringTie	transcrip	91097333	91097632	.	+	.	transcript_	MSTRG.36254.1	gene_id	MSTRG.36254;
7	StringTie	exon	91097333	91097545	.	+	.	transcript_	MSTRG.36254.1	gene_id	MSTRG.36254;
7	StringTie	exon	91097577	91097632	.	+	.	transcript_	MSTRG.36254.1	gene_id	MSTRG.36254;
7	StringTie	transcrip	103320056	103341576	.	+	.	transcript_	MSTRG.36567.1	gene_id	MSTRG.36567;
7	StringTie	exon	103320056	103320289	.	+	.	transcript_	MSTRG.36567.1	gene_id	MSTRG.36567;
7	StringTie	exon	103325974	103326167	.	+	.	transcript_	MSTRG.36567.1	gene_id	MSTRG.36567;
7	StringTie	exon	103329494	103329661	.	+	.	transcript_	MSTRG.36567.1	gene_id	MSTRG.36567;
7	StringTie	exon	103341353	103341576	.	+	.	transcript_	MSTRG.36567.1	gene_id	MSTRG.36567;
7	StringTie	transcrip	103320056	103341685	.	+	.	transcript_	MSTRG.36567.2	gene_id	MSTRG.36567;
7	StringTie	exon	103320056	103320289	.	+	.	transcript_	MSTRG.36567.2	gene_id	MSTRG.36567;
7	StringTie	exon	103329494	103329661	.	+	.	transcript_	MSTRG.36567.2	gene_id	MSTRG.36567;
7	StringTie	exon	103341353	103341685	.	+	.	transcript_	MSTRG.36567.2	gene_id	MSTRG.36567;
7	StringTie	transcrip	103320072	103341579	.	+	.	transcript_	MSTRG.36567.3	gene_id	MSTRG.36567;
7	StringTie	exon	103320072	103320289	.	+	.	transcript_	MSTRG.36567.3	gene_id	MSTRG.36567;
7	StringTie	exon	103329494	103329661	.	+	.	transcript_	MSTRG.36567.3	gene_id	MSTRG.36567;
7	StringTie	exon	103329832	103329969	.	+	.	transcript_	MSTRG.36567.3	gene_id	MSTRG.36567;
7	StringTie	exon	103331407	103331482	.	+	.	transcript_	MSTRG.36567.3	gene_id	MSTRG.36567;
7	StringTie	exon	103341353	103341579	.	+	.	transcript_	MSTRG.36567.3	gene_id	MSTRG.36567;
7	StringTie	transcrip	103320088	103326554	.	+	.	transcript_	MSTRG.36567.4	gene_id	MSTRG.36567;
7	StringTie	exon	103320088	103320266	.	+	.	transcript_	MSTRG.36567.4	gene_id	MSTRG.36567;
7	StringTie	exon	103325974	103326554	.	+	.	transcript_	MSTRG.36567.4	gene_id	MSTRG.36567;
7	StringTie	transcrip	103320228	103341637	.	+	.	transcript_	MSTRG.36567.5	gene_id	MSTRG.36567;
7	StringTie	exon	103320228	103320289	.	+	.	transcript_	MSTRG.36567.5	gene_id	MSTRG.36567;
7	StringTie	exon	103329494	103329661	.	+	.	transcript_	MSTRG.36567.5	gene_id	MSTRG.36567;
7	StringTie	exon	103331407	103331482	.	+	.	transcript_	MSTRG.36567.5	gene_id	MSTRG.36567;
7	StringTie	exon	103341353	103341637	.	+	.	transcript_	MSTRG.36567.5	gene_id	MSTRG.36567;
7	StringTie	transcrip	104009369	104012312	.	+	.	transcript_	MSTRG.36585.2	gene_id	MSTRG.36585;
7	StringTie	exon	104009369	104009596	.	+	.	transcript_	MSTRG.36585.2	gene_id	MSTRG.36585;

7	StringTie	exon	104010410	104012312	.	+	.	transcript_	MSTRG.36585.[illegible]	gene_id	MSTRG.36585;
7	StringTie	transcrip	104009369	104012590	.	+	.	transcript_	MSTRG.36585.[illegible]	gene_id	MSTRG.36585;
7	StringTie	exon	104009369	104009801	.	+	.	transcript_	MSTRG.36585.[illegible]	gene_id	MSTRG.36585;
7	StringTie	exon	104010410	104012590	.	+	.	transcript_	MSTRG.36585.[illegible]	gene_id	MSTRG.36585;
7	StringTie	transcrip	104023341	104041684	.	+	.	transcript_	MSTRG.36590.[illegible]	gene_id	MSTRG.36590;
7	StringTie	exon	104023341	104023962	.	+	.	transcript_	MSTRG.36590.[illegible]	gene_id	MSTRG.36590;
7	StringTie	exon	104026400	104026585	.	+	.	transcript_	MSTRG.36590.[illegible]	gene_id	MSTRG.36590;
7	StringTie	exon	104041600	104041684	.	+	.	transcript_	MSTRG.36590.[illegible]	gene_id	MSTRG.36590;
7	StringTie	transcrip	104032103	104041672	.	+	.	transcript_	MSTRG.36590.[illegible]	gene_id	MSTRG.36590;
7	StringTie	exon	104032103	104039274	.	+	.	transcript_	MSTRG.36590.[illegible]	gene_id	MSTRG.36590;
7	StringTie	exon	104041600	104041672	.	+	.	transcript_	MSTRG.36590.[illegible]	gene_id	MSTRG.36590;
7	StringTie	transcrip	111279260	111281765	.	+	.	transcript_	MSTRG.36622.[illegible]	gene_id	MSTRG.36622;
7	StringTie	exon	111279260	111279284	.	+	.	transcript_	MSTRG.36622.[illegible]	gene_id	MSTRG.36622;
7	StringTie	exon	111280079	111280264	.	+	.	transcript_	MSTRG.36622.[illegible]	gene_id	MSTRG.36622;
7	StringTie	exon	111281372	111281557	.	+	.	transcript_	MSTRG.36622.[illegible]	gene_id	MSTRG.36622;
7	StringTie	exon	111281674	111281765	.	+	.	transcript_	MSTRG.36622.[illegible]	gene_id	MSTRG.36622;
7	StringTie	transcrip	112935132	112936695	.	+	.	transcript_	MSTRG.36655.[illegible]	gene_id	MSTRG.36655;
7	StringTie	exon	112935132	112935299	.	+	.	transcript_	MSTRG.36655.[illegible]	gene_id	MSTRG.36655;
7	StringTie	exon	112936153	112936695	.	+	.	transcript_	MSTRG.36655.[illegible]	gene_id	MSTRG.36655;
7	StringTie	transcrip	113046444	113118367	.	+	.	transcript_	MSTRG.36661.[illegible]	gene_id	MSTRG.36661;
7	StringTie	exon	113046444	113046658	.	+	.	transcript_	MSTRG.36661.[illegible]	gene_id	MSTRG.36661;
7	StringTie	exon	113118298	113118367	.	+	.	transcript_	MSTRG.36661.[illegible]	gene_id	MSTRG.36661;
7	StringTie	transcrip	116436470	116439600	.	+	.	transcript_	MSTRG.36728.[illegible]	gene_id	MSTRG.36728;
7	StringTie	exon	116436470	116437197	.	+	.	transcript_	MSTRG.36728.[illegible]	gene_id	MSTRG.36728;
7	StringTie	exon	116438487	116439600	.	+	.	transcript_	MSTRG.36728.[illegible]	gene_id	MSTRG.36728;
7	StringTie	transcrip	116436535	116439371	.	+	.	transcript_	MSTRG.36728.[illegible]	gene_id	MSTRG.36728;
7	StringTie	exon	116436535	116437197	.	+	.	transcript_	MSTRG.36728.[illegible]	gene_id	MSTRG.36728;
7	StringTie	exon	116438487	116438583	.	+	.	transcript_	MSTRG.36728.[illegible]	gene_id	MSTRG.36728;
7	StringTie	exon	116438784	116439371	.	+	.	transcript_	MSTRG.36728.[illegible]	gene_id	MSTRG.36728;
7	StringTie	transcrip	116574114	116581328	.	+	.	transcript_	MSTRG.36718.[illegible]	gene_id	MSTRG.36718;
7	StringTie	exon	116574114	116574512	.	+	.	transcript_	MSTRG.36718.[illegible]	gene_id	MSTRG.36718;
7	StringTie	exon	116581007	116581328	.	+	.	transcript_	MSTRG.36718.[illegible]	gene_id	MSTRG.36718;
7	StringTie	transcrip	121666129	121683783	.	+	.	transcript_	MSTRG.36773.[illegible]	gene_id	MSTRG.36773;
7	StringTie	exon	121666129	121666209	.	+	.	transcript_	MSTRG.36773.[illegible]	gene_id	MSTRG.36773;
7	StringTie	exon	121674888	121674962	.	+	.	transcript_	MSTRG.36773.[illegible]	gene_id	MSTRG.36773;

7	StringTie	exon	121683616	121683783	.	+	.	transcript_	MSTRG.36773.2	gene_id	MSTRG.36773;
7	StringTie	transcrip	19618	42910	.	-	.	transcript_	MSTRG.34248.1	gene_id	MSTRG.34248;
7	StringTie	exon	19618	21492	.	-	.	transcript_	MSTRG.34248.1	gene_id	MSTRG.34248;
7	StringTie	exon	35216	35323	.	-	.	transcript_	MSTRG.34248.1	gene_id	MSTRG.34248;
7	StringTie	exon	39549	39796	.	-	.	transcript_	MSTRG.34248.1	gene_id	MSTRG.34248;
7	StringTie	exon	42806	42910	.	-	.	transcript_	MSTRG.34248.1	gene_id	MSTRG.34248;
7	StringTie	transcrip	19622	42910	.	-	.	transcript_	MSTRG.34248.2	gene_id	MSTRG.34248;
7	StringTie	exon	19622	21492	.	-	.	transcript_	MSTRG.34248.2	gene_id	MSTRG.34248;
7	StringTie	exon	35216	35323	.	-	.	transcript_	MSTRG.34248.2	gene_id	MSTRG.34248;
7	StringTie	exon	42806	42910	.	-	.	transcript_	MSTRG.34248.2	gene_id	MSTRG.34248;
7	StringTie	transcrip	19657	29139	.	-	.	transcript_	MSTRG.34248.4	gene_id	MSTRG.34248;
7	StringTie	exon	19657	21492	.	-	.	transcript_	MSTRG.34248.4	gene_id	MSTRG.34248;
7	StringTie	exon	28792	29139	.	-	.	transcript_	MSTRG.34248.4	gene_id	MSTRG.34248;
7	StringTie	transcrip	19657	44825	.	-	.	transcript_	MSTRG.34248.3	gene_id	MSTRG.34248;
7	StringTie	exon	19657	21492	.	-	.	transcript_	MSTRG.34248.3	gene_id	MSTRG.34248;
7	StringTie	exon	35216	35323	.	-	.	transcript_	MSTRG.34248.3	gene_id	MSTRG.34248;
7	StringTie	exon	39549	39796	.	-	.	transcript_	MSTRG.34248.3	gene_id	MSTRG.34248;
7	StringTie	exon	44137	44825	.	-	.	transcript_	MSTRG.34248.3	gene_id	MSTRG.34248;
7	StringTie	transcrip	20155	23848	.	-	.	transcript_	MSTRG.34248.5	gene_id	MSTRG.34248;
7	StringTie	exon	20155	21492	.	-	.	transcript_	MSTRG.34248.5	gene_id	MSTRG.34248;
7	StringTie	exon	23791	23848	.	-	.	transcript_	MSTRG.34248.5	gene_id	MSTRG.34248;
7	StringTie	transcrip	1653270	1656262	.	-	.	transcript_	MSTRG.34265.1	gene_id	MSTRG.34265;
7	StringTie	exon	1653270	1654182	.	-	.	transcript_	MSTRG.34265.1	gene_id	MSTRG.34265;
7	StringTie	exon	1656016	1656262	.	-	.	transcript_	MSTRG.34265.1	gene_id	MSTRG.34265;
7	StringTie	transcrip	2282920	2379157	.	-	.	transcript_	MSTRG.34285.1	gene_id	MSTRG.34285;
7	StringTie	exon	2282920	2283002	.	-	.	transcript_	MSTRG.34285.1	gene_id	MSTRG.34285;
7	StringTie	exon	2368145	2368264	.	-	.	transcript_	MSTRG.34285.1	gene_id	MSTRG.34285;
7	StringTie	exon	2378968	2379157	.	-	.	transcript_	MSTRG.34285.1	gene_id	MSTRG.34285;
7	StringTie	transcrip	2354358	2379002	.	-	.	transcript_	MSTRG.34285.2	gene_id	MSTRG.34285;
7	StringTie	exon	2354358	2354635	.	-	.	transcript_	MSTRG.34285.2	gene_id	MSTRG.34285;
7	StringTie	exon	2368145	2368264	.	-	.	transcript_	MSTRG.34285.2	gene_id	MSTRG.34285;
7	StringTie	exon	2378968	2379002	.	-	.	transcript_	MSTRG.34285.2	gene_id	MSTRG.34285;
7	StringTie	transcrip	2373511	2379152	.	-	.	transcript_	MSTRG.34285.3	gene_id	MSTRG.34285;
7	StringTie	exon	2373511	2373676	.	-	.	transcript_	MSTRG.34285.3	gene_id	MSTRG.34285;
7	StringTie	exon	2378968	2379152	.	-	.	transcript_	MSTRG.34285.3	gene_id	MSTRG.34285;

7	StringTie	transcrip	11242089	11245658	.	-	.	transcript_	MSTRG.34427.[illegible]	gene_id	MSTRG.34427;
7	StringTie	exon	11242089	11244290	.	-	.	transcript_	MSTRG.34427.[illegible]	gene_id	MSTRG.34427;
7	StringTie	exon	11245146	11245658	.	-	.	transcript_	MSTRG.34427.[illegible]	gene_id	MSTRG.34427;
7	StringTie	transcrip	16727932	16729640	.	-	.	transcript_	MSTRG.34542.[illegible]	gene_id	MSTRG.34542;
7	StringTie	exon	16727932	16728136	.	-	.	transcript_	MSTRG.34542.[illegible]	gene_id	MSTRG.34542;
7	StringTie	exon	16729581	16729640	.	-	.	transcript_	MSTRG.34542.[illegible]	gene_id	MSTRG.34542;
7	StringTie	transcrip	18235066	18453073	.	-	.	transcript_	MSTRG.34621.[illegible]	gene_id	MSTRG.34621;
7	StringTie	exon	18235066	18235207	.	-	.	transcript_	MSTRG.34621.[illegible]	gene_id	MSTRG.34621;
7	StringTie	exon	18312212	18312261	.	-	.	transcript_	MSTRG.34621.[illegible]	gene_id	MSTRG.34621;
7	StringTie	exon	18313332	18313378	.	-	.	transcript_	MSTRG.34621.[illegible]	gene_id	MSTRG.34621;
7	StringTie	exon	18369820	18369964	.	-	.	transcript_	MSTRG.34621.[illegible]	gene_id	MSTRG.34621;
7	StringTie	exon	18372056	18372154	.	-	.	transcript_	MSTRG.34621.[illegible]	gene_id	MSTRG.34621;
7	StringTie	exon	18452922	18453073	.	-	.	transcript_	MSTRG.34621.[illegible]	gene_id	MSTRG.34621;
7	StringTie	transcrip	19531443	19585725	.	-	.	transcript_	MSTRG.34644.[illegible]	gene_id	MSTRG.34644;
7	StringTie	exon	19531443	19531660	.	-	.	transcript_	MSTRG.34644.[illegible]	gene_id	MSTRG.34644;
7	StringTie	exon	19584879	19584920	.	-	.	transcript_	MSTRG.34644.[illegible]	gene_id	MSTRG.34644;
7	StringTie	exon	19585003	19585725	.	-	.	transcript_	MSTRG.34644.[illegible]	gene_id	MSTRG.34644;
7	StringTie	transcrip	20000837	20005284	.	-	.	transcript_	MSTRG.34696.[illegible]	gene_id	MSTRG.34696;
7	StringTie	exon	20000837	20002812	.	-	.	transcript_	MSTRG.34696.[illegible]	gene_id	MSTRG.34696;
7	StringTie	exon	20005215	20005284	.	-	.	transcript_	MSTRG.34696.[illegible]	gene_id	MSTRG.34696;
7	StringTie	transcrip	20064389	20151820	.	-	.	transcript_	MSTRG.34696.[illegible]	gene_id	MSTRG.34696;
7	StringTie	exon	20064389	20064436	.	-	.	transcript_	MSTRG.34696.[illegible]	gene_id	MSTRG.34696;
7	StringTie	exon	20071851	20079087	.	-	.	transcript_	MSTRG.34696.[illegible]	gene_id	MSTRG.34696;
7	StringTie	exon	20122371	20122506	.	-	.	transcript_	MSTRG.34696.[illegible]	gene_id	MSTRG.34696;
7	StringTie	exon	20151713	20151820	.	-	.	transcript_	MSTRG.34696.[illegible]	gene_id	MSTRG.34696;
7	StringTie	transcrip	20779961	20783670	.	-	.	transcript_	MSTRG.34677.[illegible]	gene_id	MSTRG.34677;
7	StringTie	exon	20779961	20780744	.	-	.	transcript_	MSTRG.34677.[illegible]	gene_id	MSTRG.34677;
7	StringTie	exon	20783504	20783670	.	-	.	transcript_	MSTRG.34677.[illegible]	gene_id	MSTRG.34677;
7	StringTie	transcrip	21236160	21245446	.	-	.	transcript_	MSTRG.34711.[illegible]	gene_id	MSTRG.34711;
7	StringTie	exon	21236160	21236483	.	-	.	transcript_	MSTRG.34711.[illegible]	gene_id	MSTRG.34711;
7	StringTie	exon	21244638	21245446	.	-	.	transcript_	MSTRG.34711.[illegible]	gene_id	MSTRG.34711;
7	StringTie	transcrip	22825077	22957124	.	-	.	transcript_	MSTRG.34836.[illegible]	gene_id	MSTRG.34836;
7	StringTie	exon	22825077	22825427	.	-	.	transcript_	MSTRG.34836.[illegible]	gene_id	MSTRG.34836;
7	StringTie	exon	22957014	22957124	.	-	.	transcript_	MSTRG.34836.[illegible]	gene_id	MSTRG.34836;
7	StringTie	transcrip	22825077	22957170	.	-	.	transcript_	MSTRG.34836.[illegible]	gene_id	MSTRG.34836;

7	StringTie	exon	22825077	22825427	.	-	.	transcript_	MSTRG.34836.2	gene_id	MSTRG.34836;
7	StringTie	exon	22889898	22889915	.	-	.	transcript_	MSTRG.34836.2	gene_id	MSTRG.34836;
7	StringTie	exon	22957032	22957170	.	-	.	transcript_	MSTRG.34836.2	gene_id	MSTRG.34836;
7	StringTie	transcrip	22825309	22890013	.	-	.	transcript_	MSTRG.34836.5	gene_id	MSTRG.34836;
7	StringTie	exon	22825309	22825445	.	-	.	transcript_	MSTRG.34836.5	gene_id	MSTRG.34836;
7	StringTie	exon	22889916	22890013	.	-	.	transcript_	MSTRG.34836.5	gene_id	MSTRG.34836;
7	StringTie	transcrip	22825309	22939429	.	-	.	transcript_	MSTRG.34836.7	gene_id	MSTRG.34836;
7	StringTie	exon	22825309	22825408	.	-	.	transcript_	MSTRG.34836.7	gene_id	MSTRG.34836;
7	StringTie	exon	22917936	22917972	.	-	.	transcript_	MSTRG.34836.7	gene_id	MSTRG.34836;
7	StringTie	exon	22939312	22939429	.	-	.	transcript_	MSTRG.34836.7	gene_id	MSTRG.34836;
7	StringTie	transcrip	22825309	22957108	.	-	.	transcript_	MSTRG.34836.6	gene_id	MSTRG.34836;
7	StringTie	exon	22825309	22825445	.	-	.	transcript_	MSTRG.34836.6	gene_id	MSTRG.34836;
7	StringTie	exon	22957032	22957108	.	-	.	transcript_	MSTRG.34836.6	gene_id	MSTRG.34836;
7	StringTie	transcrip	22825309	22957185	.	-	.	transcript_	MSTRG.34836.4	gene_id	MSTRG.34836;
7	StringTie	exon	22825309	22825408	.	-	.	transcript_	MSTRG.34836.4	gene_id	MSTRG.34836;
7	StringTie	exon	22917936	22917972	.	-	.	transcript_	MSTRG.34836.4	gene_id	MSTRG.34836;
7	StringTie	exon	22957032	22957185	.	-	.	transcript_	MSTRG.34836.4	gene_id	MSTRG.34836;
7	StringTie	transcrip	22825309	22957185	.	-	.	transcript_	MSTRG.34836.3	gene_id	MSTRG.34836;
7	StringTie	exon	22825309	22825427	.	-	.	transcript_	MSTRG.34836.3	gene_id	MSTRG.34836;
7	StringTie	exon	22939294	22939401	.	-	.	transcript_	MSTRG.34836.3	gene_id	MSTRG.34836;
7	StringTie	exon	22957122	22957185	.	-	.	transcript_	MSTRG.34836.3	gene_id	MSTRG.34836;
7	StringTie	transcrip	22826153	22919041	.	-	.	transcript_	MSTRG.34836.1	gene_id	MSTRG.34836;
7	StringTie	exon	22826153	22826266	.	-	.	transcript_	MSTRG.34836.1	gene_id	MSTRG.34836;
7	StringTie	exon	22890738	22890857	.	-	.	transcript_	MSTRG.34836.1	gene_id	MSTRG.34836;
7	StringTie	exon	22918918	22919041	.	-	.	transcript_	MSTRG.34836.1	gene_id	MSTRG.34836;
7	StringTie	transcrip	22890483	22976567	.	-	.	transcript_	MSTRG.34836.1	gene_id	MSTRG.34836;
7	StringTie	exon	22890483	22890513	.	-	.	transcript_	MSTRG.34836.1	gene_id	MSTRG.34836;
7	StringTie	exon	22939916	22940106	.	-	.	transcript_	MSTRG.34836.1	gene_id	MSTRG.34836;
7	StringTie	exon	22957830	22957888	.	-	.	transcript_	MSTRG.34836.1	gene_id	MSTRG.34836;
7	StringTie	exon	22976457	22976567	.	-	.	transcript_	MSTRG.34836.1	gene_id	MSTRG.34836;
7	StringTie	transcrip	22918712	22976567	.	-	.	transcript_	MSTRG.34836.2	gene_id	MSTRG.34836;
7	StringTie	exon	22918712	22918787	.	-	.	transcript_	MSTRG.34836.2	gene_id	MSTRG.34836;
7	StringTie	exon	22940107	22940165	.	-	.	transcript_	MSTRG.34836.2	gene_id	MSTRG.34836;
7	StringTie	exon	22976457	22976567	.	-	.	transcript_	MSTRG.34836.2	gene_id	MSTRG.34836;
7	StringTie	transcrip	22826802	22941673	.	-	.	transcript_	MSTRG.34836.1	gene_id	MSTRG.34836;

7	StringTie	exon	22826802	22827728	.	-	.	transcript_	MSTRG.34836.1	gene_id	MSTRG.34836;
7	StringTie	exon	22941563	22941673	.	-	.	transcript_	MSTRG.34836.1	gene_id	MSTRG.34836;
7	StringTie	transcrip	22826802	22959380	.	-	.	transcript_	MSTRG.34836.1	gene_id	MSTRG.34836;
7	StringTie	exon	22826802	22827728	.	-	.	transcript_	MSTRG.34836.1	gene_id	MSTRG.34836;
7	StringTie	exon	22959270	22959380	.	-	.	transcript_	MSTRG.34836.1	gene_id	MSTRG.34836;
7	StringTie	transcrip	22827542	22855176	.	-	.	transcript_	MSTRG.34836.1	gene_id	MSTRG.34836;
7	StringTie	exon	22827542	22827728	.	-	.	transcript_	MSTRG.34836.1	gene_id	MSTRG.34836;
7	StringTie	exon	22855125	22855176	.	-	.	transcript_	MSTRG.34836.1	gene_id	MSTRG.34836;
7	StringTie	transcrip	22827546	22892285	.	-	.	transcript_	MSTRG.34836.1	gene_id	MSTRG.34836;
7	StringTie	exon	22827546	22827728	.	-	.	transcript_	MSTRG.34836.1	gene_id	MSTRG.34836;
7	StringTie	exon	22892175	22892285	.	-	.	transcript_	MSTRG.34836.1	gene_id	MSTRG.34836;
7	StringTie	transcrip	22871227	22941673	.	-	.	transcript_	MSTRG.34836.1	gene_id	MSTRG.34836;
7	StringTie	exon	22871227	22871326	.	-	.	transcript_	MSTRG.34836.1	gene_id	MSTRG.34836;
7	StringTie	exon	22941563	22941673	.	-	.	transcript_	MSTRG.34836.1	gene_id	MSTRG.34836;
7	StringTie	transcrip	22941459	22959380	.	-	.	transcript_	MSTRG.34836.2	gene_id	MSTRG.34836;
7	StringTie	exon	22941459	22941562	.	-	.	transcript_	MSTRG.34836.2	gene_id	MSTRG.34836;
7	StringTie	exon	22959270	22959380	.	-	.	transcript_	MSTRG.34836.2	gene_id	MSTRG.34836;
7	StringTie	transcrip	22889087	22956473	.	-	.	transcript_	MSTRG.34838.1	gene_id	MSTRG.34838;
7	StringTie	exon	22889087	22889188	.	-	.	transcript_	MSTRG.34838.1	gene_id	MSTRG.34838;
7	StringTie	exon	22938585	22938643	.	-	.	transcript_	MSTRG.34838.1	gene_id	MSTRG.34838;
7	StringTie	exon	22956363	22956473	.	-	.	transcript_	MSTRG.34838.1	gene_id	MSTRG.34838;
7	StringTie	transcrip	22889424	22939114	.	-	.	transcript_	MSTRG.34839.1	gene_id	MSTRG.34839;
7	StringTie	exon	22889424	22889534	.	-	.	transcript_	MSTRG.34839.1	gene_id	MSTRG.34839;
7	StringTie	exon	22938931	22939114	.	-	.	transcript_	MSTRG.34839.1	gene_id	MSTRG.34839;
7	StringTie	transcrip	31095624	31160767	.	-	.	transcript_	MSTRG.35039.3	gene_id	MSTRG.35039;
7	StringTie	exon	31095624	31096592	.	-	.	transcript_	MSTRG.35039.3	gene_id	MSTRG.35039;
7	StringTie	exon	31114614	31114734	.	-	.	transcript_	MSTRG.35039.3	gene_id	MSTRG.35039;
7	StringTie	exon	31146135	31146236	.	-	.	transcript_	MSTRG.35039.3	gene_id	MSTRG.35039;
7	StringTie	exon	31160686	31160767	.	-	.	transcript_	MSTRG.35039.3	gene_id	MSTRG.35039;
7	StringTie	transcrip	31095753	31160796	.	-	.	transcript_	MSTRG.35039.4	gene_id	MSTRG.35039;
7	StringTie	exon	31095753	31096998	.	-	.	transcript_	MSTRG.35039.4	gene_id	MSTRG.35039;
7	StringTie	exon	31114614	31114734	.	-	.	transcript_	MSTRG.35039.4	gene_id	MSTRG.35039;
7	StringTie	exon	31146135	31146236	.	-	.	transcript_	MSTRG.35039.4	gene_id	MSTRG.35039;
7	StringTie	exon	31160686	31160796	.	-	.	transcript_	MSTRG.35039.4	gene_id	MSTRG.35039;
7	StringTie	transcrip	31126645	31160793	.	-	.	transcript_	MSTRG.35039.6	gene_id	MSTRG.35039;

7	StringTie	exon	31126645	31128482	.	-	.	transcript_	MSTRG.35039.(	gene_id	MSTRG.35039;
7	StringTie	exon	31146135	31146236	.	-	.	transcript_	MSTRG.35039.(	gene_id	MSTRG.35039;
7	StringTie	exon	31160686	31160793	.	-	.	transcript_	MSTRG.35039.(	gene_id	MSTRG.35039;
7	StringTie	transcrip	35208640	35209196	.	-	.	transcript_	MSTRG.35136.:	gene_id	MSTRG.35136;
7	StringTie	exon	35208640	35208954	.	-	.	transcript_	MSTRG.35136.:	gene_id	MSTRG.35136;
7	StringTie	exon	35209119	35209196	.	-	.	transcript_	MSTRG.35136.:	gene_id	MSTRG.35136;
7	StringTie	transcrip	35208699	35210480	.	-	.	transcript_	MSTRG.35136.:	gene_id	MSTRG.35136;
7	StringTie	exon	35208699	35208954	.	-	.	transcript_	MSTRG.35136.:	gene_id	MSTRG.35136;
7	StringTie	exon	35210291	35210480	.	-	.	transcript_	MSTRG.35136.:	gene_id	MSTRG.35136;
7	StringTie	transcrip	38343868	38344741	.	-	.	transcript_	MSTRG.35222.:	gene_id	MSTRG.35222;
7	StringTie	exon	38343868	38344129	.	-	.	transcript_	MSTRG.35222.:	gene_id	MSTRG.35222;
7	StringTie	exon	38344686	38344741	.	-	.	transcript_	MSTRG.35222.:	gene_id	MSTRG.35222;
7	StringTie	transcrip	43702320	43703924	.	-	.	transcript_	MSTRG.35322.:	gene_id	MSTRG.35322;
7	StringTie	exon	43702320	43702611	.	-	.	transcript_	MSTRG.35322.:	gene_id	MSTRG.35322;
7	StringTie	exon	43703888	43703924	.	-	.	transcript_	MSTRG.35322.:	gene_id	MSTRG.35322;
7	StringTie	transcrip	46487044	46507597	.	-	.	transcript_	MSTRG.35347.:	gene_id	MSTRG.35347;
7	StringTie	exon	46487044	46487501	.	-	.	transcript_	MSTRG.35347.:	gene_id	MSTRG.35347;
7	StringTie	exon	46507444	46507597	.	-	.	transcript_	MSTRG.35347.:	gene_id	MSTRG.35347;
7	StringTie	transcrip	53997484	54006747	.	-	.	transcript_	MSTRG.35541.:	gene_id	MSTRG.35541;
7	StringTie	exon	53997484	53997766	.	-	.	transcript_	MSTRG.35541.:	gene_id	MSTRG.35541;
7	StringTie	exon	53997915	53998035	.	-	.	transcript_	MSTRG.35541.:	gene_id	MSTRG.35541;
7	StringTie	exon	53998882	53998927	.	-	.	transcript_	MSTRG.35541.:	gene_id	MSTRG.35541;
7	StringTie	exon	54000083	54000248	.	-	.	transcript_	MSTRG.35541.:	gene_id	MSTRG.35541;
7	StringTie	exon	54006538	54006747	.	-	.	transcript_	MSTRG.35541.:	gene_id	MSTRG.35541;
7	StringTie	transcrip	55296599	55320344	.	-	.	transcript_	MSTRG.35582.:	gene_id	MSTRG.35582;
7	StringTie	exon	55296599	55296664	.	-	.	transcript_	MSTRG.35582.:	gene_id	MSTRG.35582;
7	StringTie	exon	55320038	55320344	.	-	.	transcript_	MSTRG.35582.:	gene_id	MSTRG.35582;
7	StringTie	transcrip	56183683	56185537	.	-	.	transcript_	MSTRG.35625.:	gene_id	MSTRG.35625;
7	StringTie	exon	56183683	56183721	.	-	.	transcript_	MSTRG.35625.:	gene_id	MSTRG.35625;
7	StringTie	exon	56183783	56185537	.	-	.	transcript_	MSTRG.35625.:	gene_id	MSTRG.35625;
7	StringTie	transcrip	68081123	68106455	.	-	.	transcript_	MSTRG.35858.:	gene_id	MSTRG.35858;
7	StringTie	exon	68081123	68083966	.	-	.	transcript_	MSTRG.35858.:	gene_id	MSTRG.35858;
7	StringTie	exon	68085781	68085843	.	-	.	transcript_	MSTRG.35858.:	gene_id	MSTRG.35858;
7	StringTie	exon	68088137	68088357	.	-	.	transcript_	MSTRG.35858.:	gene_id	MSTRG.35858;
7	StringTie	exon	68106411	68106455	.	-	.	transcript_	MSTRG.35858.:	gene_id	MSTRG.35858;

7	StringTie	transcrip	68148804	68181655	.	-	.	transcript_	MSTRG.35864.1	gene_id	MSTRG.35864;
7	StringTie	exon	68148804	68148862	.	-	.	transcript_	MSTRG.35864.1	gene_id	MSTRG.35864;
7	StringTie	exon	68181192	68181655	.	-	.	transcript_	MSTRG.35864.1	gene_id	MSTRG.35864;
7	StringTie	transcrip	72784874	72804659	.	-	.	transcript_	MSTRG.35899.1	gene_id	MSTRG.35899;
7	StringTie	exon	72784874	72785028	.	-	.	transcript_	MSTRG.35899.1	gene_id	MSTRG.35899;
7	StringTie	exon	72788765	72788840	.	-	.	transcript_	MSTRG.35899.1	gene_id	MSTRG.35899;
7	StringTie	exon	72804556	72804659	.	-	.	transcript_	MSTRG.35899.1	gene_id	MSTRG.35899;
7	StringTie	transcrip	74508881	74511647	.	-	.	transcript_	MSTRG.35911.1	gene_id	MSTRG.35911;
7	StringTie	exon	74508881	74509058	.	-	.	transcript_	MSTRG.35911.1	gene_id	MSTRG.35911;
7	StringTie	exon	74509905	74511647	.	-	.	transcript_	MSTRG.35911.1	gene_id	MSTRG.35911;
7	StringTie	transcrip	74790883	74847480	.	-	.	transcript_	MSTRG.35914.2	gene_id	MSTRG.35914;
7	StringTie	exon	74790883	74791007	.	-	.	transcript_	MSTRG.35914.2	gene_id	MSTRG.35914;
7	StringTie	exon	74847260	74847480	.	-	.	transcript_	MSTRG.35914.2	gene_id	MSTRG.35914;
7	StringTie	transcrip	74790972	74847533	.	-	.	transcript_	MSTRG.35914.3	gene_id	MSTRG.35914;
7	StringTie	exon	74790972	74791007	.	-	.	transcript_	MSTRG.35914.3	gene_id	MSTRG.35914;
7	StringTie	exon	74795052	74795107	.	-	.	transcript_	MSTRG.35914.3	gene_id	MSTRG.35914;
7	StringTie	exon	74847260	74847533	.	-	.	transcript_	MSTRG.35914.3	gene_id	MSTRG.35914;
7	StringTie	transcrip	74981851	74982634	.	-	.	transcript_	MSTRG.35928.1	gene_id	MSTRG.35928;
7	StringTie	exon	74981851	74982160	.	-	.	transcript_	MSTRG.35928.1	gene_id	MSTRG.35928;
7	StringTie	exon	74982498	74982634	.	-	.	transcript_	MSTRG.35928.1	gene_id	MSTRG.35928;
7	StringTie	transcrip	86109845	86124197	.	-	.	transcript_	MSTRG.36180.1	gene_id	MSTRG.36180;
7	StringTie	exon	86109845	86110331	.	-	.	transcript_	MSTRG.36180.1	gene_id	MSTRG.36180;
7	StringTie	exon	86115841	86115893	.	-	.	transcript_	MSTRG.36180.1	gene_id	MSTRG.36180;
7	StringTie	exon	86121038	86121122	.	-	.	transcript_	MSTRG.36180.1	gene_id	MSTRG.36180;
7	StringTie	exon	86121693	86121761	.	-	.	transcript_	MSTRG.36180.1	gene_id	MSTRG.36180;
7	StringTie	exon	86124071	86124197	.	-	.	transcript_	MSTRG.36180.1	gene_id	MSTRG.36180;
7	StringTie	transcrip	86109965	86123864	.	-	.	transcript_	MSTRG.36180.2	gene_id	MSTRG.36180;
7	StringTie	exon	86109965	86110331	.	-	.	transcript_	MSTRG.36180.2	gene_id	MSTRG.36180;
7	StringTie	exon	86115841	86115893	.	-	.	transcript_	MSTRG.36180.2	gene_id	MSTRG.36180;
7	StringTie	exon	86121038	86121122	.	-	.	transcript_	MSTRG.36180.2	gene_id	MSTRG.36180;
7	StringTie	exon	86121693	86121761	.	-	.	transcript_	MSTRG.36180.2	gene_id	MSTRG.36180;
7	StringTie	exon	86123750	86123864	.	-	.	transcript_	MSTRG.36180.2	gene_id	MSTRG.36180;
7	StringTie	transcrip	86109973	86123774	.	-	.	transcript_	MSTRG.36180.3	gene_id	MSTRG.36180;
7	StringTie	exon	86109973	86110331	.	-	.	transcript_	MSTRG.36180.3	gene_id	MSTRG.36180;
7	StringTie	exon	86115565	86115893	.	-	.	transcript_	MSTRG.36180.3	gene_id	MSTRG.36180;

7	StringTie	exon	86121038	86121122	.	-	.	transcript_	MSTRG.36180.3	gene_id	MSTRG.36180;
7	StringTie	exon	86121693	86121761	.	-	.	transcript_	MSTRG.36180.3	gene_id	MSTRG.36180;
7	StringTie	exon	86123504	86123774	.	-	.	transcript_	MSTRG.36180.3	gene_id	MSTRG.36180;
7	StringTie	transcrip	86110027	86124187	.	-	.	transcript_	MSTRG.36180.4	gene_id	MSTRG.36180;
7	StringTie	exon	86110027	86110331	.	-	.	transcript_	MSTRG.36180.4	gene_id	MSTRG.36180;
7	StringTie	exon	86115565	86115631	.	-	.	transcript_	MSTRG.36180.4	gene_id	MSTRG.36180;
7	StringTie	exon	86115841	86115893	.	-	.	transcript_	MSTRG.36180.4	gene_id	MSTRG.36180;
7	StringTie	exon	86121038	86121122	.	-	.	transcript_	MSTRG.36180.4	gene_id	MSTRG.36180;
7	StringTie	exon	86121693	86121761	.	-	.	transcript_	MSTRG.36180.4	gene_id	MSTRG.36180;
7	StringTie	exon	86124071	86124187	.	-	.	transcript_	MSTRG.36180.4	gene_id	MSTRG.36180;
7	StringTie	transcrip	89075192	89088317	.	-	.	transcript_	MSTRG.36236.1	gene_id	MSTRG.36236;
7	StringTie	exon	89075192	89075218	.	-	.	transcript_	MSTRG.36236.1	gene_id	MSTRG.36236;
7	StringTie	exon	89087874	89088317	.	-	.	transcript_	MSTRG.36236.1	gene_id	MSTRG.36236;
7	StringTie	transcrip	89393376	89395254	.	-	.	transcript_	MSTRG.36242.1	gene_id	MSTRG.36242;
7	StringTie	exon	89393376	89393588	.	-	.	transcript_	MSTRG.36242.1	gene_id	MSTRG.36242;
7	StringTie	exon	89395171	89395254	.	-	.	transcript_	MSTRG.36242.1	gene_id	MSTRG.36242;
7	StringTie	transcrip	92516548	92517863	.	-	.	transcript_	MSTRG.36280.1	gene_id	MSTRG.36280;
7	StringTie	exon	92516548	92517268	.	-	.	transcript_	MSTRG.36280.1	gene_id	MSTRG.36280;
7	StringTie	exon	92517650	92517863	.	-	.	transcript_	MSTRG.36280.1	gene_id	MSTRG.36280;
7	StringTie	transcrip	96563474	96569077	.	-	.	transcript_	MSTRG.36418.1	gene_id	MSTRG.36418;
7	StringTie	exon	96563474	96563557	.	-	.	transcript_	MSTRG.36418.1	gene_id	MSTRG.36418;
7	StringTie	exon	96568407	96569077	.	-	.	transcript_	MSTRG.36418.1	gene_id	MSTRG.36418;
7	StringTie	transcrip	96906048	96910879	.	-	.	transcript_	MSTRG.36430.1	gene_id	MSTRG.36430;
7	StringTie	exon	96906048	96906428	.	-	.	transcript_	MSTRG.36430.1	gene_id	MSTRG.36430;
7	StringTie	exon	96910669	96910879	.	-	.	transcript_	MSTRG.36430.1	gene_id	MSTRG.36430;
7	StringTie	transcrip	98771174	98810283	.	-	.	transcript_	MSTRG.36481.1	gene_id	MSTRG.36481;
7	StringTie	exon	98771174	98771272	.	-	.	transcript_	MSTRG.36481.1	gene_id	MSTRG.36481;
7	StringTie	exon	98809790	98810283	.	-	.	transcript_	MSTRG.36481.1	gene_id	MSTRG.36481;
7	StringTie	transcrip	98771300	98810283	.	-	.	transcript_	MSTRG.36481.2	gene_id	MSTRG.36481;
7	StringTie	exon	98771300	98771410	.	-	.	transcript_	MSTRG.36481.2	gene_id	MSTRG.36481;
7	StringTie	exon	98809940	98810283	.	-	.	transcript_	MSTRG.36481.2	gene_id	MSTRG.36481;
7	StringTie	transcrip	98862257	98865063	.	-	.	transcript_	MSTRG.36487.1	gene_id	MSTRG.36487;
7	StringTie	exon	98862257	98864342	.	-	.	transcript_	MSTRG.36487.1	gene_id	MSTRG.36487;
7	StringTie	exon	98864720	98865063	.	-	.	transcript_	MSTRG.36487.1	gene_id	MSTRG.36487;
7	StringTie	transcrip	112318632	112321512	.	-	.	transcript_	MSTRG.36646.1	gene_id	MSTRG.36646;

7	StringTie	exon	112318632	112319573	.	-	.	transcript_	MSTRG.36646.1	gene_id	MSTRG.36646;
7	StringTie	exon	112319718	112321512	.	-	.	transcript_	MSTRG.36646.1	gene_id	MSTRG.36646;
7	StringTie	transcrip	114233408	114234332	.	-	.	transcript_	MSTRG.36690.1	gene_id	MSTRG.36690;
7	StringTie	exon	114233408	114233687	.	-	.	transcript_	MSTRG.36690.1	gene_id	MSTRG.36690;
7	StringTie	exon	114234225	114234332	.	-	.	transcript_	MSTRG.36690.1	gene_id	MSTRG.36690;
7	StringTie	transcrip	115261158	115262016	.	-	.	transcript_	MSTRG.36699.1	gene_id	MSTRG.36699;
7	StringTie	exon	115261158	115261219	.	-	.	transcript_	MSTRG.36699.1	gene_id	MSTRG.36699;
7	StringTie	exon	115261838	115262016	.	-	.	transcript_	MSTRG.36699.1	gene_id	MSTRG.36699;
7	StringTie	transcrip	118053311	118055856	.	-	.	transcript_	MSTRG.36741.4	gene_id	MSTRG.36741;
7	StringTie	exon	118053311	118053570	.	-	.	transcript_	MSTRG.36741.4	gene_id	MSTRG.36741;
7	StringTie	exon	118055210	118055396	.	-	.	transcript_	MSTRG.36741.4	gene_id	MSTRG.36741;
7	StringTie	exon	118055559	118055856	.	-	.	transcript_	MSTRG.36741.4	gene_id	MSTRG.36741;
7	StringTie	transcrip	118053311	118055856	.	-	.	transcript_	MSTRG.36741.3	gene_id	MSTRG.36741;
7	StringTie	exon	118053311	118053570	.	-	.	transcript_	MSTRG.36741.3	gene_id	MSTRG.36741;
7	StringTie	exon	118055210	118055451	.	-	.	transcript_	MSTRG.36741.3	gene_id	MSTRG.36741;
7	StringTie	exon	118055559	118055856	.	-	.	transcript_	MSTRG.36741.3	gene_id	MSTRG.36741;
7	StringTie	transcrip	118053311	118060169	.	-	.	transcript_	MSTRG.36741.2	gene_id	MSTRG.36741;
7	StringTie	exon	118053311	118053570	.	-	.	transcript_	MSTRG.36741.2	gene_id	MSTRG.36741;
7	StringTie	exon	118059705	118060169	.	-	.	transcript_	MSTRG.36741.2	gene_id	MSTRG.36741;
7	StringTie	transcrip	118053311	118060169	.	-	.	transcript_	MSTRG.36741.1	gene_id	MSTRG.36741;
7	StringTie	exon	118053311	118053570	.	-	.	transcript_	MSTRG.36741.1	gene_id	MSTRG.36741;
7	StringTie	exon	118055210	118055396	.	-	.	transcript_	MSTRG.36741.1	gene_id	MSTRG.36741;
7	StringTie	exon	118059705	118060169	.	-	.	transcript_	MSTRG.36741.1	gene_id	MSTRG.36741;
7	StringTie	transcrip	118053315	118055745	.	-	.	transcript_	MSTRG.36741.5	gene_id	MSTRG.36741;
7	StringTie	exon	118053315	118053570	.	-	.	transcript_	MSTRG.36741.5	gene_id	MSTRG.36741;
7	StringTie	exon	118055559	118055745	.	-	.	transcript_	MSTRG.36741.5	gene_id	MSTRG.36741;
7	StringTie	transcrip	118053316	118059825	.	-	.	transcript_	MSTRG.36741.6	gene_id	MSTRG.36741;
7	StringTie	exon	118053316	118053570	.	-	.	transcript_	MSTRG.36741.6	gene_id	MSTRG.36741;
7	StringTie	exon	118055210	118055451	.	-	.	transcript_	MSTRG.36741.6	gene_id	MSTRG.36741;
7	StringTie	exon	118059705	118059825	.	-	.	transcript_	MSTRG.36741.6	gene_id	MSTRG.36741;
8	StringTie	transcrip	383135	384704	.	+	.	transcript_	MSTRG.36786.1	gene_id	MSTRG.36786;
8	StringTie	exon	383135	383643	.	+	.	transcript_	MSTRG.36786.1	gene_id	MSTRG.36786;
8	StringTie	exon	383824	384704	.	+	.	transcript_	MSTRG.36786.1	gene_id	MSTRG.36786;
8	StringTie	transcrip	6423575	6425251	.	+	.	transcript_	MSTRG.36904.1	gene_id	MSTRG.36904;
8	StringTie	exon	6423575	6423685	.	+	.	transcript_	MSTRG.36904.1	gene_id	MSTRG.36904;

8	StringTie	exon	6425157	6425251	.	+	.	transcript_	MSTRG.36904.[illegible]	gene_id	MSTRG.36904;
8	StringTie	transcrip	12702127	12712701	.	+	.	transcript_	MSTRG.36980.[illegible]	gene_id	MSTRG.36980;
8	StringTie	exon	12702127	12702157	.	+	.	transcript_	MSTRG.36980.[illegible]	gene_id	MSTRG.36980;
8	StringTie	exon	12712271	12712701	.	+	.	transcript_	MSTRG.36980.[illegible]	gene_id	MSTRG.36980;
8	StringTie	transcrip	18334669	18346652	.	+	.	transcript_	MSTRG.37007.[illegible]	gene_id	MSTRG.37007;
8	StringTie	exon	18334669	18334730	.	+	.	transcript_	MSTRG.37007.[illegible]	gene_id	MSTRG.37007;
8	StringTie	exon	18345971	18346652	.	+	.	transcript_	MSTRG.37007.[illegible]	gene_id	MSTRG.37007;
8	StringTie	transcrip	44462502	44478675	.	+	.	transcript_	MSTRG.37365.[illegible]	gene_id	MSTRG.37365;
8	StringTie	exon	44462502	44463138	.	+	.	transcript_	MSTRG.37365.[illegible]	gene_id	MSTRG.37365;
8	StringTie	exon	44463875	44463924	.	+	.	transcript_	MSTRG.37365.[illegible]	gene_id	MSTRG.37365;
8	StringTie	exon	44478046	44478675	.	+	.	transcript_	MSTRG.37365.[illegible]	gene_id	MSTRG.37365;
8	StringTie	transcrip	47007218	47010075	.	+	.	transcript_	MSTRG.37379.[illegible]	gene_id	MSTRG.37379;
8	StringTie	exon	47007218	47007349	.	+	.	transcript_	MSTRG.37379.[illegible]	gene_id	MSTRG.37379;
8	StringTie	exon	47008014	47008127	.	+	.	transcript_	MSTRG.37379.[illegible]	gene_id	MSTRG.37379;
8	StringTie	exon	47009920	47010075	.	+	.	transcript_	MSTRG.37379.[illegible]	gene_id	MSTRG.37379;
8	StringTie	transcrip	47007252	47010176	.	+	.	transcript_	MSTRG.37379.[illegible]	gene_id	MSTRG.37379;
8	StringTie	exon	47007252	47007349	.	+	.	transcript_	MSTRG.37379.[illegible]	gene_id	MSTRG.37379;
8	StringTie	exon	47008014	47008127	.	+	.	transcript_	MSTRG.37379.[illegible]	gene_id	MSTRG.37379;
8	StringTie	exon	47009955	47010176	.	+	.	transcript_	MSTRG.37379.[illegible]	gene_id	MSTRG.37379;
8	StringTie	transcrip	47098463	47113139	.	+	.	transcript_	MSTRG.37387.[illegible]	gene_id	MSTRG.37387;
8	StringTie	exon	47098463	47098632	.	+	.	transcript_	MSTRG.37387.[illegible]	gene_id	MSTRG.37387;
8	StringTie	exon	47111487	47111728	.	+	.	transcript_	MSTRG.37387.[illegible]	gene_id	MSTRG.37387;
8	StringTie	exon	47112088	47113139	.	+	.	transcript_	MSTRG.37387.[illegible]	gene_id	MSTRG.37387;
8	StringTie	transcrip	66129244	66132994	.	+	.	transcript_	MSTRG.37594.[illegible]	gene_id	MSTRG.37594;
8	StringTie	exon	66129244	66129631	.	+	.	transcript_	MSTRG.37594.[illegible]	gene_id	MSTRG.37594;
8	StringTie	exon	66132583	66132994	.	+	.	transcript_	MSTRG.37594.[illegible]	gene_id	MSTRG.37594;
8	StringTie	transcrip	66561939	66602263	.	+	.	transcript_	MSTRG.37579.[illegible]	gene_id	MSTRG.37579;
8	StringTie	exon	66561939	66562053	.	+	.	transcript_	MSTRG.37579.[illegible]	gene_id	MSTRG.37579;
8	StringTie	exon	66601475	66602263	.	+	.	transcript_	MSTRG.37579.[illegible]	gene_id	MSTRG.37579;
8	StringTie	transcrip	67609057	67612680	.	+	.	transcript_	MSTRG.37634.[illegible]	gene_id	MSTRG.37634;
8	StringTie	exon	67609057	67609342	.	+	.	transcript_	MSTRG.37634.[illegible]	gene_id	MSTRG.37634;
8	StringTie	exon	67612599	67612680	.	+	.	transcript_	MSTRG.37634.[illegible]	gene_id	MSTRG.37634;
8	StringTie	transcrip	80889057	80898675	.	+	.	transcript_	MSTRG.37826.[illegible]	gene_id	MSTRG.37826;
8	StringTie	exon	80889057	80889292	.	+	.	transcript_	MSTRG.37826.[illegible]	gene_id	MSTRG.37826;
8	StringTie	exon	80897238	80897367	.	+	.	transcript_	MSTRG.37826.[illegible]	gene_id	MSTRG.37826;

8	StringTie	exon	80897843	80898675	.	+	.	transcript_	MSTRG.37826.1	gene_id	MSTRG.37826;
8	StringTie	transcrip	85637467	85686020	.	+	.	transcript_	MSTRG.37922.1	gene_id	MSTRG.37922;
8	StringTie	exon	85637467	85637495	.	+	.	transcript_	MSTRG.37922.1	gene_id	MSTRG.37922;
8	StringTie	exon	85679499	85686020	.	+	.	transcript_	MSTRG.37922.1	gene_id	MSTRG.37922;
8	StringTie	transcrip	87570303	87586268	.	+	.	transcript_	MSTRG.37976.1	gene_id	MSTRG.37976;
8	StringTie	exon	87570303	87570375	.	+	.	transcript_	MSTRG.37976.1	gene_id	MSTRG.37976;
8	StringTie	exon	87570618	87570820	.	+	.	transcript_	MSTRG.37976.1	gene_id	MSTRG.37976;
8	StringTie	exon	87571470	87571641	.	+	.	transcript_	MSTRG.37976.1	gene_id	MSTRG.37976;
8	StringTie	exon	87584914	87586268	.	+	.	transcript_	MSTRG.37976.1	gene_id	MSTRG.37976;
8	StringTie	transcrip	87570303	87608869	.	+	.	transcript_	MSTRG.37976.2	gene_id	MSTRG.37976;
8	StringTie	exon	87570303	87570375	.	+	.	transcript_	MSTRG.37976.2	gene_id	MSTRG.37976;
8	StringTie	exon	87570618	87570820	.	+	.	transcript_	MSTRG.37976.2	gene_id	MSTRG.37976;
8	StringTie	exon	87571470	87571641	.	+	.	transcript_	MSTRG.37976.2	gene_id	MSTRG.37976;
8	StringTie	exon	87608550	87608667	.	+	.	transcript_	MSTRG.37976.2	gene_id	MSTRG.37976;
8	StringTie	exon	87608769	87608869	.	+	.	transcript_	MSTRG.37976.2	gene_id	MSTRG.37976;
8	StringTie	transcrip	96143487	96143930	.	+	.	transcript_	MSTRG.38030.1	gene_id	MSTRG.38030;
8	StringTie	exon	96143487	96143710	.	+	.	transcript_	MSTRG.38030.1	gene_id	MSTRG.38030;
8	StringTie	exon	96143771	96143930	.	+	.	transcript_	MSTRG.38030.1	gene_id	MSTRG.38030;
8	StringTie	transcrip	97039024	97040520	.	+	.	transcript_	MSTRG.38052.1	gene_id	MSTRG.38052;
8	StringTie	exon	97039024	97039059	.	+	.	transcript_	MSTRG.38052.1	gene_id	MSTRG.38052;
8	StringTie	exon	97040307	97040520	.	+	.	transcript_	MSTRG.38052.1	gene_id	MSTRG.38052;
8	StringTie	transcrip	101284412	101297302	.	+	.	transcript_	MSTRG.38105.5	gene_id	MSTRG.38105;
8	StringTie	exon	101284412	101284490	.	+	.	transcript_	MSTRG.38105.5	gene_id	MSTRG.38105;
8	StringTie	exon	101284699	101284798	.	+	.	transcript_	MSTRG.38105.5	gene_id	MSTRG.38105;
8	StringTie	exon	101287910	101287993	.	+	.	transcript_	MSTRG.38105.5	gene_id	MSTRG.38105;
8	StringTie	exon	101290169	101290347	.	+	.	transcript_	MSTRG.38105.5	gene_id	MSTRG.38105;
8	StringTie	exon	101291324	101291424	.	+	.	transcript_	MSTRG.38105.5	gene_id	MSTRG.38105;
8	StringTie	exon	101295075	101297302	.	+	.	transcript_	MSTRG.38105.5	gene_id	MSTRG.38105;
8	StringTie	transcrip	110006401	110006972	.	+	.	transcript_	MSTRG.38201.1	gene_id	MSTRG.38201;
8	StringTie	exon	110006401	110006456	.	+	.	transcript_	MSTRG.38201.1	gene_id	MSTRG.38201;
8	StringTie	exon	110006731	110006972	.	+	.	transcript_	MSTRG.38201.1	gene_id	MSTRG.38201;
8	StringTie	transcrip	110461832	110470144	.	+	.	transcript_	MSTRG.38246.1	gene_id	MSTRG.38246;
8	StringTie	exon	110461832	110462370	.	+	.	transcript_	MSTRG.38246.1	gene_id	MSTRG.38246;
8	StringTie	exon	110463506	110470144	.	+	.	transcript_	MSTRG.38246.1	gene_id	MSTRG.38246;
8	StringTie	transcrip	110471344	110486374	.	+	.	transcript_	MSTRG.38247.1	gene_id	MSTRG.38247;

8	StringTie	exon	110471344	110473307	.	+	.	transcript_	MSTRG.38247.	gene_id	MSTRG.38247;
8	StringTie	exon	110474400	110486374	.	+	.	transcript_	MSTRG.38247.	gene_id	MSTRG.38247;
8	StringTie	transcrip	110505292	111529703	.	+	.	transcript_	MSTRG.38248.	gene_id	MSTRG.38248;
8	StringTie	exon	110505292	110505397	.	+	.	transcript_	MSTRG.38248.	gene_id	MSTRG.38248;
8	StringTie	exon	110555988	110556063	.	+	.	transcript_	MSTRG.38248.	gene_id	MSTRG.38248;
8	StringTie	exon	110775738	110775865	.	+	.	transcript_	MSTRG.38248.	gene_id	MSTRG.38248;
8	StringTie	exon	110965934	110966000	.	+	.	transcript_	MSTRG.38248.	gene_id	MSTRG.38248;
8	StringTie	exon	111092560	111092630	.	+	.	transcript_	MSTRG.38248.	gene_id	MSTRG.38248;
8	StringTie	exon	111411576	111411717	.	+	.	transcript_	MSTRG.38248.	gene_id	MSTRG.38248;
8	StringTie	exon	111416359	111416443	.	+	.	transcript_	MSTRG.38248.	gene_id	MSTRG.38248;
8	StringTie	exon	111424351	111424432	.	+	.	transcript_	MSTRG.38248.	gene_id	MSTRG.38248;
8	StringTie	exon	111529682	111529703	.	+	.	transcript_	MSTRG.38248.	gene_id	MSTRG.38248;
8	StringTie	transcrip	110529009	110637001	.	+	.	transcript_	MSTRG.38248.	gene_id	MSTRG.38248;
8	StringTie	exon	110529009	110529081	.	+	.	transcript_	MSTRG.38248.	gene_id	MSTRG.38248;
8	StringTie	exon	110538148	110538202	.	+	.	transcript_	MSTRG.38248.	gene_id	MSTRG.38248;
8	StringTie	exon	110555988	110556063	.	+	.	transcript_	MSTRG.38248.	gene_id	MSTRG.38248;
8	StringTie	exon	110636950	110637001	.	+	.	transcript_	MSTRG.38248.	gene_id	MSTRG.38248;
8	StringTie	transcrip	111753849	111953369	.	+	.	transcript_	MSTRG.38257.	gene_id	MSTRG.38257;
8	StringTie	exon	111753849	111753899	.	+	.	transcript_	MSTRG.38257.	gene_id	MSTRG.38257;
8	StringTie	exon	111817010	111817103	.	+	.	transcript_	MSTRG.38257.	gene_id	MSTRG.38257;
8	StringTie	exon	111829191	111829302	.	+	.	transcript_	MSTRG.38257.	gene_id	MSTRG.38257;
8	StringTie	exon	111952613	111953369	.	+	.	transcript_	MSTRG.38257.	gene_id	MSTRG.38257;
8	StringTie	transcrip	111753858	111832456	.	+	.	transcript_	MSTRG.38257.	gene_id	MSTRG.38257;
8	StringTie	exon	111753858	111753899	.	+	.	transcript_	MSTRG.38257.	gene_id	MSTRG.38257;
8	StringTie	exon	111829191	111832456	.	+	.	transcript_	MSTRG.38257.	gene_id	MSTRG.38257;
8	StringTie	transcrip	111817044	111864063	.	+	.	transcript_	MSTRG.38257.	gene_id	MSTRG.38257;
8	StringTie	exon	111817044	111817103	.	+	.	transcript_	MSTRG.38257.	gene_id	MSTRG.38257;
8	StringTie	exon	111829191	111829302	.	+	.	transcript_	MSTRG.38257.	gene_id	MSTRG.38257;
8	StringTie	exon	111862969	111864063	.	+	.	transcript_	MSTRG.38257.	gene_id	MSTRG.38257;
8	StringTie	transcrip	112324568	112338138	.	+	.	transcript_	MSTRG.38291.	gene_id	MSTRG.38291;
8	StringTie	exon	112324568	112324624	.	+	.	transcript_	MSTRG.38291.	gene_id	MSTRG.38291;
8	StringTie	exon	112337924	112338138	.	+	.	transcript_	MSTRG.38291.	gene_id	MSTRG.38291;
8	StringTie	transcrip	114146088	114170135	.	+	.	transcript_	MSTRG.38339.	gene_id	MSTRG.38339;
8	StringTie	exon	114146088	114146144	.	+	.	transcript_	MSTRG.38339.	gene_id	MSTRG.38339;
8	StringTie	exon	114146846	114146935	.	+	.	transcript_	MSTRG.38339.	gene_id	MSTRG.38339;

8	StringTie	exon	114169795	114170135	.	+	.	transcript_	MSTRG.38339.[illegible]	gene_id	MSTRG.38339;
8	StringTie	transcrip	130468075	130471802	.	+	.	transcript_	MSTRG.38617.[illegible]	gene_id	MSTRG.38617;
8	StringTie	exon	130468075	130468278	.	+	.	transcript_	MSTRG.38617.[illegible]	gene_id	MSTRG.38617;
8	StringTie	exon	130470334	130471802	.	+	.	transcript_	MSTRG.38617.[illegible]	gene_id	MSTRG.38617;
8	StringTie	transcrip	130950605	131007824	.	+	.	transcript_	MSTRG.38633.[illegible]	gene_id	MSTRG.38633;
8	StringTie	exon	130950605	130950627	.	+	.	transcript_	MSTRG.38633.[illegible]	gene_id	MSTRG.38633;
8	StringTie	exon	131007251	131007824	.	+	.	transcript_	MSTRG.38633.[illegible]	gene_id	MSTRG.38633;
8	StringTie	transcrip	131473758	131487268	.	+	.	transcript_	MSTRG.38639.[illegible]	gene_id	MSTRG.38639;
8	StringTie	exon	131473758	131473952	.	+	.	transcript_	MSTRG.38639.[illegible]	gene_id	MSTRG.38639;
8	StringTie	exon	131487197	131487268	.	+	.	transcript_	MSTRG.38639.[illegible]	gene_id	MSTRG.38639;
8	StringTie	transcrip	135204061	135206417	.	+	.	transcript_	MSTRG.38723.[illegible]	gene_id	MSTRG.38723;
8	StringTie	exon	135204061	135204168	.	+	.	transcript_	MSTRG.38723.[illegible]	gene_id	MSTRG.38723;
8	StringTie	exon	135206109	135206417	.	+	.	transcript_	MSTRG.38723.[illegible]	gene_id	MSTRG.38723;
8	StringTie	transcrip	135420747	135429670	.	+	.	transcript_	MSTRG.38761.[illegible]	gene_id	MSTRG.38761;
8	StringTie	exon	135420747	135421042	.	+	.	transcript_	MSTRG.38761.[illegible]	gene_id	MSTRG.38761;
8	StringTie	exon	135421180	135421328	.	+	.	transcript_	MSTRG.38761.[illegible]	gene_id	MSTRG.38761;
8	StringTie	exon	135424299	135424367	.	+	.	transcript_	MSTRG.38761.[illegible]	gene_id	MSTRG.38761;
8	StringTie	exon	135426086	135426180	.	+	.	transcript_	MSTRG.38761.[illegible]	gene_id	MSTRG.38761;
8	StringTie	exon	135426403	135426542	.	+	.	transcript_	MSTRG.38761.[illegible]	gene_id	MSTRG.38761;
8	StringTie	exon	135426893	135426969	.	+	.	transcript_	MSTRG.38761.[illegible]	gene_id	MSTRG.38761;
8	StringTie	exon	135428264	135429670	.	+	.	transcript_	MSTRG.38761.[illegible]	gene_id	MSTRG.38761;
8	StringTie	transcrip	356620	367097	.	-	.	transcript_	MSTRG.36784.[illegible]	gene_id	MSTRG.36784;
8	StringTie	exon	356620	357880	.	-	.	transcript_	MSTRG.36784.[illegible]	gene_id	MSTRG.36784;
8	StringTie	exon	363181	363222	.	-	.	transcript_	MSTRG.36784.[illegible]	gene_id	MSTRG.36784;
8	StringTie	exon	366871	367097	.	-	.	transcript_	MSTRG.36784.[illegible]	gene_id	MSTRG.36784;
8	StringTie	transcrip	356893	366712	.	-	.	transcript_	MSTRG.36784.[illegible]	gene_id	MSTRG.36784;
8	StringTie	exon	356893	357880	.	-	.	transcript_	MSTRG.36784.[illegible]	gene_id	MSTRG.36784;
8	StringTie	exon	363181	363222	.	-	.	transcript_	MSTRG.36784.[illegible]	gene_id	MSTRG.36784;
8	StringTie	exon	364467	364557	.	-	.	transcript_	MSTRG.36784.[illegible]	gene_id	MSTRG.36784;
8	StringTie	exon	366031	366123	.	-	.	transcript_	MSTRG.36784.[illegible]	gene_id	MSTRG.36784;
8	StringTie	exon	366459	366712	.	-	.	transcript_	MSTRG.36784.[illegible]	gene_id	MSTRG.36784;
8	StringTie	transcrip	356907	366093	.	-	.	transcript_	MSTRG.36784.[illegible]	gene_id	MSTRG.36784;
8	StringTie	exon	356907	357880	.	-	.	transcript_	MSTRG.36784.[illegible]	gene_id	MSTRG.36784;
8	StringTie	exon	362139	362242	.	-	.	transcript_	MSTRG.36784.[illegible]	gene_id	MSTRG.36784;
8	StringTie	exon	363181	363222	.	-	.	transcript_	MSTRG.36784.[illegible]	gene_id	MSTRG.36784;

8	StringTie	exon	366031	366093	.	-	.	transcript_	MSTRG.36784.4	gene_id	MSTRG.36784;
8	StringTie	transcrip	399242	399928	.	-	.	transcript_	MSTRG.36788.1	gene_id	MSTRG.36788;
8	StringTie	exon	399242	399550	.	-	.	transcript_	MSTRG.36788.1	gene_id	MSTRG.36788;
8	StringTie	exon	399840	399928	.	-	.	transcript_	MSTRG.36788.1	gene_id	MSTRG.36788;
8	StringTie	transcrip	1382347	1387543	.	-	.	transcript_	MSTRG.36831.1	gene_id	MSTRG.36831;
8	StringTie	exon	1382347	1382810	.	-	.	transcript_	MSTRG.36831.1	gene_id	MSTRG.36831;
8	StringTie	exon	1382907	1383122	.	-	.	transcript_	MSTRG.36831.1	gene_id	MSTRG.36831;
8	StringTie	exon	1386949	1387543	.	-	.	transcript_	MSTRG.36831.1	gene_id	MSTRG.36831;
8	StringTie	transcrip	3764933	3768937	.	-	.	transcript_	MSTRG.36865.1	gene_id	MSTRG.36865;
8	StringTie	exon	3764933	3765306	.	-	.	transcript_	MSTRG.36865.1	gene_id	MSTRG.36865;
8	StringTie	exon	3768365	3768435	.	-	.	transcript_	MSTRG.36865.1	gene_id	MSTRG.36865;
8	StringTie	exon	3768815	3768937	.	-	.	transcript_	MSTRG.36865.1	gene_id	MSTRG.36865;
8	StringTie	transcrip	3764945	3768925	.	-	.	transcript_	MSTRG.36865.2	gene_id	MSTRG.36865;
8	StringTie	exon	3764945	3765306	.	-	.	transcript_	MSTRG.36865.2	gene_id	MSTRG.36865;
8	StringTie	exon	3768815	3768925	.	-	.	transcript_	MSTRG.36865.2	gene_id	MSTRG.36865;
8	StringTie	transcrip	10748769	10750464	.	-	.	transcript_	MSTRG.36945.1	gene_id	MSTRG.36945;
8	StringTie	exon	10748769	10749034	.	-	.	transcript_	MSTRG.36945.1	gene_id	MSTRG.36945;
8	StringTie	exon	10749422	10749480	.	-	.	transcript_	MSTRG.36945.1	gene_id	MSTRG.36945;
8	StringTie	exon	10750109	10750464	.	-	.	transcript_	MSTRG.36945.1	gene_id	MSTRG.36945;
8	StringTie	transcrip	12467608	12525499	.	-	.	transcript_	MSTRG.36971.1	gene_id	MSTRG.36971;
8	StringTie	exon	12467608	12467701	.	-	.	transcript_	MSTRG.36971.1	gene_id	MSTRG.36971;
8	StringTie	exon	12494816	12494904	.	-	.	transcript_	MSTRG.36971.1	gene_id	MSTRG.36971;
8	StringTie	exon	12525413	12525499	.	-	.	transcript_	MSTRG.36971.1	gene_id	MSTRG.36971;
8	StringTie	transcrip	19969758	19975904	.	-	.	transcript_	MSTRG.37047.1	gene_id	MSTRG.37047;
8	StringTie	exon	19969758	19970530	.	-	.	transcript_	MSTRG.37047.1	gene_id	MSTRG.37047;
8	StringTie	exon	19974013	19975904	.	-	.	transcript_	MSTRG.37047.1	gene_id	MSTRG.37047;
8	StringTie	transcrip	28306560	28306822	.	-	.	transcript_	MSTRG.37120.2	gene_id	MSTRG.37120;
8	StringTie	exon	28306560	28306657	.	-	.	transcript_	MSTRG.37120.2	gene_id	MSTRG.37120;
8	StringTie	exon	28306685	28306822	.	-	.	transcript_	MSTRG.37120.2	gene_id	MSTRG.37120;
8	StringTie	transcrip	28306560	28306822	.	-	.	transcript_	MSTRG.37120.1	gene_id	MSTRG.37120;
8	StringTie	exon	28306560	28306684	.	-	.	transcript_	MSTRG.37120.1	gene_id	MSTRG.37120;
8	StringTie	exon	28306712	28306822	.	-	.	transcript_	MSTRG.37120.1	gene_id	MSTRG.37120;
8	StringTie	transcrip	33359097	33379503	.	-	.	transcript_	MSTRG.37196.1	gene_id	MSTRG.37196;
8	StringTie	exon	33359097	33359216	.	-	.	transcript_	MSTRG.37196.1	gene_id	MSTRG.37196;
8	StringTie	exon	33378424	33379503	.	-	.	transcript_	MSTRG.37196.1	gene_id	MSTRG.37196;

8	StringTie	transcrip	70082397	70083634	.	-	.	transcript_	MSTRG.37649.1	gene_id	MSTRG.37649;
8	StringTie	exon	70082397	70082546	.	-	.	transcript_	MSTRG.37649.1	gene_id	MSTRG.37649;
8	StringTie	exon	70083500	70083634	.	-	.	transcript_	MSTRG.37649.1	gene_id	MSTRG.37649;
8	StringTie	transcrip	70099227	70100969	.	-	.	transcript_	MSTRG.37650.1	gene_id	MSTRG.37650;
8	StringTie	exon	70099227	70099294	.	-	.	transcript_	MSTRG.37650.1	gene_id	MSTRG.37650;
8	StringTie	exon	70100175	70100308	.	-	.	transcript_	MSTRG.37650.1	gene_id	MSTRG.37650;
8	StringTie	exon	70100844	70100969	.	-	.	transcript_	MSTRG.37650.1	gene_id	MSTRG.37650;
8	StringTie	transcrip	74651533	74655677	.	-	.	transcript_	MSTRG.37750.1	gene_id	MSTRG.37750;
8	StringTie	exon	74651533	74652534	.	-	.	transcript_	MSTRG.37750.1	gene_id	MSTRG.37750;
8	StringTie	exon	74655286	74655677	.	-	.	transcript_	MSTRG.37750.1	gene_id	MSTRG.37750;
8	StringTie	transcrip	81070886	81085666	.	-	.	transcript_	MSTRG.37830.1	gene_id	MSTRG.37830;
8	StringTie	exon	81070886	81070900	.	-	.	transcript_	MSTRG.37830.1	gene_id	MSTRG.37830;
8	StringTie	exon	81085009	81085666	.	-	.	transcript_	MSTRG.37830.1	gene_id	MSTRG.37830;
8	StringTie	transcrip	86070145	86071701	.	-	.	transcript_	MSTRG.37921.1	gene_id	MSTRG.37921;
8	StringTie	exon	86070145	86070304	.	-	.	transcript_	MSTRG.37921.1	gene_id	MSTRG.37921;
8	StringTie	exon	86071519	86071701	.	-	.	transcript_	MSTRG.37921.1	gene_id	MSTRG.37921;
8	StringTie	transcrip	87459092	87461393	.	-	.	transcript_	MSTRG.37941.2	gene_id	MSTRG.37941;
8	StringTie	exon	87459092	87460067	.	-	.	transcript_	MSTRG.37941.2	gene_id	MSTRG.37941;
8	StringTie	exon	87460710	87460856	.	-	.	transcript_	MSTRG.37941.2	gene_id	MSTRG.37941;
8	StringTie	exon	87461001	87461098	.	-	.	transcript_	MSTRG.37941.2	gene_id	MSTRG.37941;
8	StringTie	exon	87461195	87461393	.	-	.	transcript_	MSTRG.37941.2	gene_id	MSTRG.37941;
8	StringTie	transcrip	87459092	87461393	.	-	.	transcript_	MSTRG.37941.1	gene_id	MSTRG.37941;
8	StringTie	exon	87459092	87460067	.	-	.	transcript_	MSTRG.37941.1	gene_id	MSTRG.37941;
8	StringTie	exon	87460710	87460856	.	-	.	transcript_	MSTRG.37941.1	gene_id	MSTRG.37941;
8	StringTie	exon	87461001	87461098	.	-	.	transcript_	MSTRG.37941.1	gene_id	MSTRG.37941;
8	StringTie	exon	87461190	87461393	.	-	.	transcript_	MSTRG.37941.1	gene_id	MSTRG.37941;
8	StringTie	transcrip	87459586	87461342	.	-	.	transcript_	MSTRG.37941.5	gene_id	MSTRG.37941;
8	StringTie	exon	87459586	87460067	.	-	.	transcript_	MSTRG.37941.5	gene_id	MSTRG.37941;
8	StringTie	exon	87461001	87461098	.	-	.	transcript_	MSTRG.37941.5	gene_id	MSTRG.37941;
8	StringTie	exon	87461195	87461342	.	-	.	transcript_	MSTRG.37941.5	gene_id	MSTRG.37941;
8	StringTie	transcrip	87459586	87461342	.	-	.	transcript_	MSTRG.37941.4	gene_id	MSTRG.37941;
8	StringTie	exon	87459586	87460067	.	-	.	transcript_	MSTRG.37941.4	gene_id	MSTRG.37941;
8	StringTie	exon	87461001	87461098	.	-	.	transcript_	MSTRG.37941.4	gene_id	MSTRG.37941;
8	StringTie	exon	87461190	87461342	.	-	.	transcript_	MSTRG.37941.4	gene_id	MSTRG.37941;
8	StringTie	transcrip	87459586	87464714	.	-	.	transcript_	MSTRG.37941.3	gene_id	MSTRG.37941;

8	StringTie	exon	87459586	87460067	.	-	.	transcript_	MSTRG.37941.:	gene_id	MSTRG.37941;
8	StringTie	exon	87461001	87461098	.	-	.	transcript_	MSTRG.37941.:	gene_id	MSTRG.37941;
8	StringTie	exon	87463798	87463858	.	-	.	transcript_	MSTRG.37941.:	gene_id	MSTRG.37941;
8	StringTie	exon	87464283	87464348	.	-	.	transcript_	MSTRG.37941.:	gene_id	MSTRG.37941;
8	StringTie	exon	87464522	87464714	.	-	.	transcript_	MSTRG.37941.:	gene_id	MSTRG.37941;
8	StringTie	transcrip	87459601	87464333	.	-	.	transcript_	MSTRG.37941.(	gene_id	MSTRG.37941;
8	StringTie	exon	87459601	87460067	.	-	.	transcript_	MSTRG.37941.(	gene_id	MSTRG.37941;
8	StringTie	exon	87461001	87461101	.	-	.	transcript_	MSTRG.37941.(	gene_id	MSTRG.37941;
8	StringTie	exon	87463798	87463858	.	-	.	transcript_	MSTRG.37941.(	gene_id	MSTRG.37941;
8	StringTie	exon	87464283	87464333	.	-	.	transcript_	MSTRG.37941.(	gene_id	MSTRG.37941;
8	StringTie	transcrip	96540536	96693882	.	-	.	transcript_	MSTRG.38032.:	gene_id	MSTRG.38032;
8	StringTie	exon	96540536	96540614	.	-	.	transcript_	MSTRG.38032.:	gene_id	MSTRG.38032;
8	StringTie	exon	96648481	96648672	.	-	.	transcript_	MSTRG.38032.:	gene_id	MSTRG.38032;
8	StringTie	exon	96686661	96686766	.	-	.	transcript_	MSTRG.38032.:	gene_id	MSTRG.38032;
8	StringTie	exon	96687384	96687491	.	-	.	transcript_	MSTRG.38032.:	gene_id	MSTRG.38032;
8	StringTie	exon	96693440	96693882	.	-	.	transcript_	MSTRG.38032.:	gene_id	MSTRG.38032;
8	StringTie	transcrip	96665514	96693946	.	-	.	transcript_	MSTRG.38032.:	gene_id	MSTRG.38032;
8	StringTie	exon	96665514	96665584	.	-	.	transcript_	MSTRG.38032.:	gene_id	MSTRG.38032;
8	StringTie	exon	96686661	96686766	.	-	.	transcript_	MSTRG.38032.:	gene_id	MSTRG.38032;
8	StringTie	exon	96687384	96687491	.	-	.	transcript_	MSTRG.38032.:	gene_id	MSTRG.38032;
8	StringTie	exon	96693440	96693946	.	-	.	transcript_	MSTRG.38032.:	gene_id	MSTRG.38032;
8	StringTie	transcrip	96263368	96328921	.	-	.	transcript_	MSTRG.38033.:	gene_id	MSTRG.38033;
8	StringTie	exon	96263368	96263402	.	-	.	transcript_	MSTRG.38033.:	gene_id	MSTRG.38033;
8	StringTie	exon	96328684	96328921	.	-	.	transcript_	MSTRG.38033.:	gene_id	MSTRG.38033;
8	StringTie	transcrip	99776346	100200974	.	-	.	transcript_	MSTRG.38082.:	gene_id	MSTRG.38082;
8	StringTie	exon	99776346	99776728	.	-	.	transcript_	MSTRG.38082.:	gene_id	MSTRG.38082;
8	StringTie	exon	99967575	99967669	.	-	.	transcript_	MSTRG.38082.:	gene_id	MSTRG.38082;
8	StringTie	exon	100071700	100071786	.	-	.	transcript_	MSTRG.38082.:	gene_id	MSTRG.38082;
8	StringTie	exon	100200731	100200974	.	-	.	transcript_	MSTRG.38082.:	gene_id	MSTRG.38082;
8	StringTie	transcrip	99776346	100200974	.	-	.	transcript_	MSTRG.38082.:	gene_id	MSTRG.38082;
8	StringTie	exon	99776346	99776728	.	-	.	transcript_	MSTRG.38082.:	gene_id	MSTRG.38082;
8	StringTie	exon	99899201	99899317	.	-	.	transcript_	MSTRG.38082.:	gene_id	MSTRG.38082;
8	StringTie	exon	99967575	99967669	.	-	.	transcript_	MSTRG.38082.:	gene_id	MSTRG.38082;
8	StringTie	exon	100071700	100071786	.	-	.	transcript_	MSTRG.38082.:	gene_id	MSTRG.38082;
8	StringTie	exon	100200731	100200974	.	-	.	transcript_	MSTRG.38082.:	gene_id	MSTRG.38082;

8	StringTie	transcrip	104403036	104489752	.	-	.	transcript_	MSTRG.38154.1	gene_id	MSTRG.38154;
8	StringTie	exon	104403036	104403062	.	-	.	transcript_	MSTRG.38154.1	gene_id	MSTRG.38154;
8	StringTie	exon	104414579	104414627	.	-	.	transcript_	MSTRG.38154.1	gene_id	MSTRG.38154;
8	StringTie	exon	104417213	104417327	.	-	.	transcript_	MSTRG.38154.1	gene_id	MSTRG.38154;
8	StringTie	exon	104420833	104420922	.	-	.	transcript_	MSTRG.38154.1	gene_id	MSTRG.38154;
8	StringTie	exon	104459229	104459289	.	-	.	transcript_	MSTRG.38154.1	gene_id	MSTRG.38154;
8	StringTie	exon	104489701	104489752	.	-	.	transcript_	MSTRG.38154.1	gene_id	MSTRG.38154;
8	StringTie	transcrip	104412692	104489769	.	-	.	transcript_	MSTRG.38154.2	gene_id	MSTRG.38154;
8	StringTie	exon	104412692	104413029	.	-	.	transcript_	MSTRG.38154.2	gene_id	MSTRG.38154;
8	StringTie	exon	104414579	104414627	.	-	.	transcript_	MSTRG.38154.2	gene_id	MSTRG.38154;
8	StringTie	exon	104417213	104417327	.	-	.	transcript_	MSTRG.38154.2	gene_id	MSTRG.38154;
8	StringTie	exon	104420833	104420922	.	-	.	transcript_	MSTRG.38154.2	gene_id	MSTRG.38154;
8	StringTie	exon	104459229	104459289	.	-	.	transcript_	MSTRG.38154.2	gene_id	MSTRG.38154;
8	StringTie	exon	104489701	104489769	.	-	.	transcript_	MSTRG.38154.2	gene_id	MSTRG.38154;
8	StringTie	transcrip	104412885	104459292	.	-	.	transcript_	MSTRG.38154.3	gene_id	MSTRG.38154;
8	StringTie	exon	104412885	104412969	.	-	.	transcript_	MSTRG.38154.3	gene_id	MSTRG.38154;
8	StringTie	exon	104414579	104414627	.	-	.	transcript_	MSTRG.38154.3	gene_id	MSTRG.38154;
8	StringTie	exon	104417213	104417327	.	-	.	transcript_	MSTRG.38154.3	gene_id	MSTRG.38154;
8	StringTie	exon	104417406	104417499	.	-	.	transcript_	MSTRG.38154.3	gene_id	MSTRG.38154;
8	StringTie	exon	104420829	104420922	.	-	.	transcript_	MSTRG.38154.3	gene_id	MSTRG.38154;
8	StringTie	exon	104459229	104459292	.	-	.	transcript_	MSTRG.38154.3	gene_id	MSTRG.38154;
8	StringTie	transcrip	104412895	104459275	.	-	.	transcript_	MSTRG.38154.4	gene_id	MSTRG.38154;
8	StringTie	exon	104412895	104412969	.	-	.	transcript_	MSTRG.38154.4	gene_id	MSTRG.38154;
8	StringTie	exon	104414579	104414627	.	-	.	transcript_	MSTRG.38154.4	gene_id	MSTRG.38154;
8	StringTie	exon	104417213	104417327	.	-	.	transcript_	MSTRG.38154.4	gene_id	MSTRG.38154;
8	StringTie	exon	104420833	104420922	.	-	.	transcript_	MSTRG.38154.4	gene_id	MSTRG.38154;
8	StringTie	exon	104459229	104459275	.	-	.	transcript_	MSTRG.38154.4	gene_id	MSTRG.38154;
8	StringTie	transcrip	105197395	105198092	.	-	.	transcript_	MSTRG.38184.1	gene_id	MSTRG.38184;
8	StringTie	exon	105197395	105197504	.	-	.	transcript_	MSTRG.38184.1	gene_id	MSTRG.38184;
8	StringTie	exon	105197688	105197731	.	-	.	transcript_	MSTRG.38184.1	gene_id	MSTRG.38184;
8	StringTie	exon	105197982	105198092	.	-	.	transcript_	MSTRG.38184.1	gene_id	MSTRG.38184;
8	StringTie	transcrip	110181202	110181841	.	-	.	transcript_	MSTRG.38238.1	gene_id	MSTRG.38238;
8	StringTie	exon	110181202	110181519	.	-	.	transcript_	MSTRG.38238.1	gene_id	MSTRG.38238;
8	StringTie	exon	110181771	110181841	.	-	.	transcript_	MSTRG.38238.1	gene_id	MSTRG.38238;
8	StringTie	transcrip	110377878	110378293	.	-	.	transcript_	MSTRG.38240.1	gene_id	MSTRG.38240;

8	StringTie	exon	110377878	110378156	.	-	.	transcript_	MSTRG.38240.1	gene_id	MSTRG.38240;
8	StringTie	exon	110378253	110378293	.	-	.	transcript_	MSTRG.38240.1	gene_id	MSTRG.38240;
8	StringTie	transcrip	111258562	111264177	.	-	.	transcript_	MSTRG.38252.1	gene_id	MSTRG.38252;
8	StringTie	exon	111258562	111259567	.	-	.	transcript_	MSTRG.38252.1	gene_id	MSTRG.38252;
8	StringTie	exon	111263855	111264177	.	-	.	transcript_	MSTRG.38252.1	gene_id	MSTRG.38252;
8	StringTie	transcrip	111984934	112028160	.	-	.	transcript_	MSTRG.38284.1	gene_id	MSTRG.38284;
8	StringTie	exon	111984934	111985548	.	-	.	transcript_	MSTRG.38284.1	gene_id	MSTRG.38284;
8	StringTie	exon	112016351	112016435	.	-	.	transcript_	MSTRG.38284.1	gene_id	MSTRG.38284;
8	StringTie	exon	112017025	112017167	.	-	.	transcript_	MSTRG.38284.1	gene_id	MSTRG.38284;
8	StringTie	exon	112028082	112028160	.	-	.	transcript_	MSTRG.38284.1	gene_id	MSTRG.38284;
8	StringTie	transcrip	112012623	112028184	.	-	.	transcript_	MSTRG.38284.6	gene_id	MSTRG.38284;
8	StringTie	exon	112012623	112015021	.	-	.	transcript_	MSTRG.38284.6	gene_id	MSTRG.38284;
8	StringTie	exon	112016351	112016435	.	-	.	transcript_	MSTRG.38284.6	gene_id	MSTRG.38284;
8	StringTie	exon	112017025	112017167	.	-	.	transcript_	MSTRG.38284.6	gene_id	MSTRG.38284;
8	StringTie	exon	112028082	112028184	.	-	.	transcript_	MSTRG.38284.6	gene_id	MSTRG.38284;
8	StringTie	transcrip	112012697	112028184	.	-	.	transcript_	MSTRG.38284.7	gene_id	MSTRG.38284;
8	StringTie	exon	112012697	112015021	.	-	.	transcript_	MSTRG.38284.7	gene_id	MSTRG.38284;
8	StringTie	exon	112016463	112016639	.	-	.	transcript_	MSTRG.38284.7	gene_id	MSTRG.38284;
8	StringTie	exon	112017025	112017167	.	-	.	transcript_	MSTRG.38284.7	gene_id	MSTRG.38284;
8	StringTie	exon	112028082	112028184	.	-	.	transcript_	MSTRG.38284.7	gene_id	MSTRG.38284;
8	StringTie	transcrip	112041648	112046081	.	-	.	transcript_	MSTRG.38255.1	gene_id	MSTRG.38255;
8	StringTie	exon	112041648	112045404	.	-	.	transcript_	MSTRG.38255.1	gene_id	MSTRG.38255;
8	StringTie	exon	112045451	112046081	.	-	.	transcript_	MSTRG.38255.1	gene_id	MSTRG.38255;
8	StringTie	transcrip	112340092	112348098	.	-	.	transcript_	MSTRG.38289.1	gene_id	MSTRG.38289;
8	StringTie	exon	112340092	112340282	.	-	.	transcript_	MSTRG.38289.1	gene_id	MSTRG.38289;
8	StringTie	exon	112347958	112348098	.	-	.	transcript_	MSTRG.38289.1	gene_id	MSTRG.38289;
8	StringTie	transcrip	118197144	118202771	.	-	.	transcript_	MSTRG.38416.1	gene_id	MSTRG.38416;
8	StringTie	exon	118197144	118200840	.	-	.	transcript_	MSTRG.38416.1	gene_id	MSTRG.38416;
8	StringTie	exon	118202631	118202771	.	-	.	transcript_	MSTRG.38416.1	gene_id	MSTRG.38416;
8	StringTie	transcrip	119424800	119430821	.	-	.	transcript_	MSTRG.38428.1	gene_id	MSTRG.38428;
8	StringTie	exon	119424800	119426794	.	-	.	transcript_	MSTRG.38428.1	gene_id	MSTRG.38428;
8	StringTie	exon	119428353	119428493	.	-	.	transcript_	MSTRG.38428.1	gene_id	MSTRG.38428;
8	StringTie	exon	119430798	119430821	.	-	.	transcript_	MSTRG.38428.1	gene_id	MSTRG.38428;
8	StringTie	transcrip	120164418	120168150	.	-	.	transcript_	MSTRG.38433.2	gene_id	MSTRG.38433;
8	StringTie	exon	120164418	120166188	.	-	.	transcript_	MSTRG.38433.2	gene_id	MSTRG.38433;

8	StringTie	exon	120168065	120168150	.	-	.	transcript_	MSTRG.38433.	gene_id	MSTRG.38433;
8	StringTie	transcrip	120164418	120309738	.	-	.	transcript_	MSTRG.38433.	gene_id	MSTRG.38433;
8	StringTie	exon	120164418	120166188	.	-	.	transcript_	MSTRG.38433.	gene_id	MSTRG.38433;
8	StringTie	exon	120167960	120168344	.	-	.	transcript_	MSTRG.38433.	gene_id	MSTRG.38433;
8	StringTie	exon	120309686	120309738	.	-	.	transcript_	MSTRG.38433.	gene_id	MSTRG.38433;
8	StringTie	transcrip	120726925	120804505	.	-	.	transcript_	MSTRG.38441.	gene_id	MSTRG.38441;
8	StringTie	exon	120726925	120726959	.	-	.	transcript_	MSTRG.38441.	gene_id	MSTRG.38441;
8	StringTie	exon	120804031	120804505	.	-	.	transcript_	MSTRG.38441.	gene_id	MSTRG.38441;
8	StringTie	transcrip	127718904	127720410	.	-	.	transcript_	MSTRG.38494.	gene_id	MSTRG.38494;
8	StringTie	exon	127718904	127719417	.	-	.	transcript_	MSTRG.38494.	gene_id	MSTRG.38494;
8	StringTie	exon	127720358	127720410	.	-	.	transcript_	MSTRG.38494.	gene_id	MSTRG.38494;
8	StringTie	transcrip	129869521	129876414	.	-	.	transcript_	MSTRG.38593.	gene_id	MSTRG.38593;
8	StringTie	exon	129869521	129872842	.	-	.	transcript_	MSTRG.38593.	gene_id	MSTRG.38593;
8	StringTie	exon	129872958	129876414	.	-	.	transcript_	MSTRG.38593.	gene_id	MSTRG.38593;
8	StringTie	transcrip	130930522	130984484	.	-	.	transcript_	MSTRG.38632.	gene_id	MSTRG.38632;
8	StringTie	exon	130930522	130930694	.	-	.	transcript_	MSTRG.38632.	gene_id	MSTRG.38632;
8	StringTie	exon	130984070	130984484	.	-	.	transcript_	MSTRG.38632.	gene_id	MSTRG.38632;
8	StringTie	transcrip	130963128	131024073	.	-	.	transcript_	MSTRG.38634.	gene_id	MSTRG.38634;
8	StringTie	exon	130963128	130963158	.	-	.	transcript_	MSTRG.38634.	gene_id	MSTRG.38634;
8	StringTie	exon	131022193	131024073	.	-	.	transcript_	MSTRG.38634.	gene_id	MSTRG.38634;
8	StringTie	transcrip	132893965	132894666	.	-	.	transcript_	MSTRG.38678.	gene_id	MSTRG.38678;
8	StringTie	exon	132893965	132894392	.	-	.	transcript_	MSTRG.38678.	gene_id	MSTRG.38678;
8	StringTie	exon	132894429	132894666	.	-	.	transcript_	MSTRG.38678.	gene_id	MSTRG.38678;
9	StringTie	transcrip	212669	219634	.	+	.	transcript_	MSTRG.38796.	gene_id	MSTRG.38796;
9	StringTie	exon	212669	212694	.	+	.	transcript_	MSTRG.38796.	gene_id	MSTRG.38796;
9	StringTie	exon	219388	219634	.	+	.	transcript_	MSTRG.38796.	gene_id	MSTRG.38796;
9	StringTie	transcrip	3341637	3348481	.	+	.	transcript_	MSTRG.38876.	gene_id	MSTRG.38876;
9	StringTie	exon	3341637	3341840	.	+	.	transcript_	MSTRG.38876.	gene_id	MSTRG.38876;
9	StringTie	exon	3348324	3348481	.	+	.	transcript_	MSTRG.38876.	gene_id	MSTRG.38876;
9	StringTie	transcrip	3341657	3345086	.	+	.	transcript_	MSTRG.38876.	gene_id	MSTRG.38876;
9	StringTie	exon	3341657	3341840	.	+	.	transcript_	MSTRG.38876.	gene_id	MSTRG.38876;
9	StringTie	exon	3344404	3345086	.	+	.	transcript_	MSTRG.38876.	gene_id	MSTRG.38876;
9	StringTie	transcrip	6628148	6636320	.	+	.	transcript_	MSTRG.38938.	gene_id	MSTRG.38938;
9	StringTie	exon	6628148	6628395	.	+	.	transcript_	MSTRG.38938.	gene_id	MSTRG.38938;
9	StringTie	exon	6633757	6636320	.	+	.	transcript_	MSTRG.38938.	gene_id	MSTRG.38938;

9	StringTie	transcrip	6629356	6634805	.	+	.	transcript_	MSTRG.38938.[illegible]	gene_id	MSTRG.38938;
9	StringTie	exon	6629356	6629444	.	+	.	transcript_	MSTRG.38938.[illegible]	gene_id	MSTRG.38938;
9	StringTie	exon	6633757	6634805	.	+	.	transcript_	MSTRG.38938.[illegible]	gene_id	MSTRG.38938;
9	StringTie	transcrip	7207299	7209795	.	+	.	transcript_	MSTRG.38961.[illegible]	gene_id	MSTRG.38961;
9	StringTie	exon	7207299	7207471	.	+	.	transcript_	MSTRG.38961.[illegible]	gene_id	MSTRG.38961;
9	StringTie	exon	7209766	7209795	.	+	.	transcript_	MSTRG.38961.[illegible]	gene_id	MSTRG.38961;
9	StringTie	transcrip	8391585	8403575	.	+	.	transcript_	MSTRG.39006.[illegible]	gene_id	MSTRG.39006;
9	StringTie	exon	8391585	8391994	.	+	.	transcript_	MSTRG.39006.[illegible]	gene_id	MSTRG.39006;
9	StringTie	exon	8393073	8393675	.	+	.	transcript_	MSTRG.39006.[illegible]	gene_id	MSTRG.39006;
9	StringTie	exon	8395149	8395299	.	+	.	transcript_	MSTRG.39006.[illegible]	gene_id	MSTRG.39006;
9	StringTie	exon	8395839	8396088	.	+	.	transcript_	MSTRG.39006.[illegible]	gene_id	MSTRG.39006;
9	StringTie	exon	8396439	8396565	.	+	.	transcript_	MSTRG.39006.[illegible]	gene_id	MSTRG.39006;
9	StringTie	exon	8397171	8398694	.	+	.	transcript_	MSTRG.39006.[illegible]	gene_id	MSTRG.39006;
9	StringTie	exon	8399650	8399738	.	+	.	transcript_	MSTRG.39006.[illegible]	gene_id	MSTRG.39006;
9	StringTie	exon	8400194	8400298	.	+	.	transcript_	MSTRG.39006.[illegible]	gene_id	MSTRG.39006;
9	StringTie	exon	8400718	8400939	.	+	.	transcript_	MSTRG.39006.[illegible]	gene_id	MSTRG.39006;
9	StringTie	exon	8402430	8403575	.	+	.	transcript_	MSTRG.39006.[illegible]	gene_id	MSTRG.39006;
9	StringTie	transcrip	8391713	8403183	.	+	.	transcript_	MSTRG.39006.[illegible]	gene_id	MSTRG.39006;
9	StringTie	exon	8391713	8391994	.	+	.	transcript_	MSTRG.39006.[illegible]	gene_id	MSTRG.39006;
9	StringTie	exon	8393073	8393675	.	+	.	transcript_	MSTRG.39006.[illegible]	gene_id	MSTRG.39006;
9	StringTie	exon	8395149	8395299	.	+	.	transcript_	MSTRG.39006.[illegible]	gene_id	MSTRG.39006;
9	StringTie	exon	8395839	8396088	.	+	.	transcript_	MSTRG.39006.[illegible]	gene_id	MSTRG.39006;
9	StringTie	exon	8396439	8396565	.	+	.	transcript_	MSTRG.39006.[illegible]	gene_id	MSTRG.39006;
9	StringTie	exon	8397171	8399738	.	+	.	transcript_	MSTRG.39006.[illegible]	gene_id	MSTRG.39006;
9	StringTie	exon	8400194	8400298	.	+	.	transcript_	MSTRG.39006.[illegible]	gene_id	MSTRG.39006;
9	StringTie	exon	8400718	8400939	.	+	.	transcript_	MSTRG.39006.[illegible]	gene_id	MSTRG.39006;
9	StringTie	exon	8402430	8403183	.	+	.	transcript_	MSTRG.39006.[illegible]	gene_id	MSTRG.39006;
9	StringTie	transcrip	8391854	8403406	.	+	.	transcript_	MSTRG.39006.[illegible]	gene_id	MSTRG.39006;
9	StringTie	exon	8391854	8391994	.	+	.	transcript_	MSTRG.39006.[illegible]	gene_id	MSTRG.39006;
9	StringTie	exon	8393073	8393675	.	+	.	transcript_	MSTRG.39006.[illegible]	gene_id	MSTRG.39006;
9	StringTie	exon	8395149	8395299	.	+	.	transcript_	MSTRG.39006.[illegible]	gene_id	MSTRG.39006;
9	StringTie	exon	8396439	8400298	.	+	.	transcript_	MSTRG.39006.[illegible]	gene_id	MSTRG.39006;
9	StringTie	exon	8400718	8400939	.	+	.	transcript_	MSTRG.39006.[illegible]	gene_id	MSTRG.39006;
9	StringTie	exon	8402430	8403406	.	+	.	transcript_	MSTRG.39006.[illegible]	gene_id	MSTRG.39006;
9	StringTie	transcrip	8392670	8403575	.	+	.	transcript_	MSTRG.39006.[illegible]	gene_id	MSTRG.39006;

9	StringTie	exon	8392670	8393675	.	+	.	transcript_	MSTRG.39006.4	gene_id	MSTRG.39006;
9	StringTie	exon	8395839	8396088	.	+	.	transcript_	MSTRG.39006.4	gene_id	MSTRG.39006;
9	StringTie	exon	8396439	8396565	.	+	.	transcript_	MSTRG.39006.4	gene_id	MSTRG.39006;
9	StringTie	exon	8397171	8399738	.	+	.	transcript_	MSTRG.39006.4	gene_id	MSTRG.39006;
9	StringTie	exon	8400194	8400298	.	+	.	transcript_	MSTRG.39006.4	gene_id	MSTRG.39006;
9	StringTie	exon	8400718	8400939	.	+	.	transcript_	MSTRG.39006.4	gene_id	MSTRG.39006;
9	StringTie	exon	8402430	8403575	.	+	.	transcript_	MSTRG.39006.4	gene_id	MSTRG.39006;
9	StringTie	transcrip	8396367	8403630	.	+	.	transcript_	MSTRG.39006.7	gene_id	MSTRG.39006;
9	StringTie	exon	8396367	8398694	.	+	.	transcript_	MSTRG.39006.7	gene_id	MSTRG.39006;
9	StringTie	exon	8399665	8399738	.	+	.	transcript_	MSTRG.39006.7	gene_id	MSTRG.39006;
9	StringTie	exon	8400194	8400298	.	+	.	transcript_	MSTRG.39006.7	gene_id	MSTRG.39006;
9	StringTie	exon	8400718	8400939	.	+	.	transcript_	MSTRG.39006.7	gene_id	MSTRG.39006;
9	StringTie	exon	8402430	8403630	.	+	.	transcript_	MSTRG.39006.7	gene_id	MSTRG.39006;
9	StringTie	transcrip	8396367	8403677	.	+	.	transcript_	MSTRG.39006.6	gene_id	MSTRG.39006;
9	StringTie	exon	8396367	8397251	.	+	.	transcript_	MSTRG.39006.6	gene_id	MSTRG.39006;
9	StringTie	exon	8397749	8397989	.	+	.	transcript_	MSTRG.39006.6	gene_id	MSTRG.39006;
9	StringTie	exon	8398532	8400298	.	+	.	transcript_	MSTRG.39006.6	gene_id	MSTRG.39006;
9	StringTie	exon	8400718	8400939	.	+	.	transcript_	MSTRG.39006.6	gene_id	MSTRG.39006;
9	StringTie	exon	8402430	8403677	.	+	.	transcript_	MSTRG.39006.6	gene_id	MSTRG.39006;
9	StringTie	transcrip	8396415	8403648	.	+	.	transcript_	MSTRG.39006.9	gene_id	MSTRG.39006;
9	StringTie	exon	8396415	8396565	.	+	.	transcript_	MSTRG.39006.9	gene_id	MSTRG.39006;
9	StringTie	exon	8402156	8402231	.	+	.	transcript_	MSTRG.39006.9	gene_id	MSTRG.39006;
9	StringTie	exon	8402430	8403648	.	+	.	transcript_	MSTRG.39006.9	gene_id	MSTRG.39006;
9	StringTie	transcrip	8396459	8402974	.	+	.	transcript_	MSTRG.39006.1	gene_id	MSTRG.39006;
9	StringTie	exon	8396459	8400298	.	+	.	transcript_	MSTRG.39006.1	gene_id	MSTRG.39006;
9	StringTie	exon	8400718	8400939	.	+	.	transcript_	MSTRG.39006.1	gene_id	MSTRG.39006;
9	StringTie	exon	8402156	8402231	.	+	.	transcript_	MSTRG.39006.1	gene_id	MSTRG.39006;
9	StringTie	exon	8402430	8402974	.	+	.	transcript_	MSTRG.39006.1	gene_id	MSTRG.39006;
9	StringTie	transcrip	8397744	8411609	.	+	.	transcript_	MSTRG.39006.1	gene_id	MSTRG.39006;
9	StringTie	exon	8397744	8398694	.	+	.	transcript_	MSTRG.39006.1	gene_id	MSTRG.39006;
9	StringTie	exon	8410435	8411609	.	+	.	transcript_	MSTRG.39006.1	gene_id	MSTRG.39006;
9	StringTie	transcrip	8796015	8809119	.	+	.	transcript_	MSTRG.38997.1	gene_id	MSTRG.38997;
9	StringTie	exon	8796015	8796145	.	+	.	transcript_	MSTRG.38997.1	gene_id	MSTRG.38997;
9	StringTie	exon	8807690	8809119	.	+	.	transcript_	MSTRG.38997.1	gene_id	MSTRG.38997;
9	StringTie	transcrip	9779068	9780683	.	+	.	transcript_	MSTRG.39026.1	gene_id	MSTRG.39026;

9	StringTie	exon	9779068	9779528	.	+	.	transcript_	MSTRG.39026.1	gene_id	MSTRG.39026;
9	StringTie	exon	9780043	9780683	.	+	.	transcript_	MSTRG.39026.1	gene_id	MSTRG.39026;
9	StringTie	transcrip	9935588	9997293	.	+	.	transcript_	MSTRG.39039.1	gene_id	MSTRG.39039;
9	StringTie	exon	9935588	9935750	.	+	.	transcript_	MSTRG.39039.1	gene_id	MSTRG.39039;
9	StringTie	exon	9996636	9997293	.	+	.	transcript_	MSTRG.39039.1	gene_id	MSTRG.39039;
9	StringTie	transcrip	10965765	10971371	.	+	.	transcript_	MSTRG.39076.6	gene_id	MSTRG.39076;
9	StringTie	exon	10965765	10965964	.	+	.	transcript_	MSTRG.39076.6	gene_id	MSTRG.39076;
9	StringTie	exon	10967857	10967952	.	+	.	transcript_	MSTRG.39076.6	gene_id	MSTRG.39076;
9	StringTie	exon	10968089	10968170	.	+	.	transcript_	MSTRG.39076.6	gene_id	MSTRG.39076;
9	StringTie	exon	10970864	10971371	.	+	.	transcript_	MSTRG.39076.6	gene_id	MSTRG.39076;
9	StringTie	transcrip	10965765	10971371	.	+	.	transcript_	MSTRG.39076.4	gene_id	MSTRG.39076;
9	StringTie	exon	10965765	10965890	.	+	.	transcript_	MSTRG.39076.4	gene_id	MSTRG.39076;
9	StringTie	exon	10967857	10967952	.	+	.	transcript_	MSTRG.39076.4	gene_id	MSTRG.39076;
9	StringTie	exon	10968089	10968170	.	+	.	transcript_	MSTRG.39076.4	gene_id	MSTRG.39076;
9	StringTie	exon	10970864	10971371	.	+	.	transcript_	MSTRG.39076.4	gene_id	MSTRG.39076;
9	StringTie	transcrip	10966061	10968552	.	+	.	transcript_	MSTRG.39076.8	gene_id	MSTRG.39076;
9	StringTie	exon	10966061	10966657	.	+	.	transcript_	MSTRG.39076.8	gene_id	MSTRG.39076;
9	StringTie	exon	10967857	10967952	.	+	.	transcript_	MSTRG.39076.8	gene_id	MSTRG.39076;
9	StringTie	exon	10968089	10968170	.	+	.	transcript_	MSTRG.39076.8	gene_id	MSTRG.39076;
9	StringTie	exon	10968276	10968552	.	+	.	transcript_	MSTRG.39076.8	gene_id	MSTRG.39076;
9	StringTie	transcrip	10969724	10971371	.	+	.	transcript_	MSTRG.39076.9	gene_id	MSTRG.39076;
9	StringTie	exon	10969724	10970621	.	+	.	transcript_	MSTRG.39076.9	gene_id	MSTRG.39076;
9	StringTie	exon	10970650	10971371	.	+	.	transcript_	MSTRG.39076.9	gene_id	MSTRG.39076;
9	StringTie	transcrip	10983184	10991311	.	+	.	transcript_	MSTRG.39076.1	gene_id	MSTRG.39076;
9	StringTie	exon	10983184	10985142	.	+	.	transcript_	MSTRG.39076.1	gene_id	MSTRG.39076;
9	StringTie	exon	10990926	10991311	.	+	.	transcript_	MSTRG.39076.1	gene_id	MSTRG.39076;
9	StringTie	transcrip	20034427	20052525	.	+	.	transcript_	MSTRG.39253.1	gene_id	MSTRG.39253;
9	StringTie	exon	20034427	20034641	.	+	.	transcript_	MSTRG.39253.1	gene_id	MSTRG.39253;
9	StringTie	exon	20039480	20039574	.	+	.	transcript_	MSTRG.39253.1	gene_id	MSTRG.39253;
9	StringTie	exon	20042362	20042472	.	+	.	transcript_	MSTRG.39253.1	gene_id	MSTRG.39253;
9	StringTie	exon	20050168	20052525	.	+	.	transcript_	MSTRG.39253.1	gene_id	MSTRG.39253;
9	StringTie	transcrip	20034431	20036510	.	+	.	transcript_	MSTRG.39253.2	gene_id	MSTRG.39253;
9	StringTie	exon	20034431	20034641	.	+	.	transcript_	MSTRG.39253.2	gene_id	MSTRG.39253;
9	StringTie	exon	20036261	20036510	.	+	.	transcript_	MSTRG.39253.2	gene_id	MSTRG.39253;
9	StringTie	transcrip	21761696	21774950	.	+	.	transcript_	MSTRG.39292.1	gene_id	MSTRG.39292;

9	StringTie	exon	21761696	21761742	.	+	.	transcript_	MSTRG.39292.	gene_id	MSTRG.39292;
9	StringTie	exon	21772973	21773084	.	+	.	transcript_	MSTRG.39292.	gene_id	MSTRG.39292;
9	StringTie	exon	21774621	21774950	.	+	.	transcript_	MSTRG.39292.	gene_id	MSTRG.39292;
9	StringTie	transcrip	21815583	21843550	.	+	.	transcript_	MSTRG.39292.	gene_id	MSTRG.39292;
9	StringTie	exon	21815583	21816072	.	+	.	transcript_	MSTRG.39292.	gene_id	MSTRG.39292;
9	StringTie	exon	21832756	21832965	.	+	.	transcript_	MSTRG.39292.	gene_id	MSTRG.39292;
9	StringTie	exon	21837617	21837743	.	+	.	transcript_	MSTRG.39292.	gene_id	MSTRG.39292;
9	StringTie	exon	21843500	21843550	.	+	.	transcript_	MSTRG.39292.	gene_id	MSTRG.39292;
9	StringTie	transcrip	21921441	21953702	.	+	.	transcript_	MSTRG.39295.	gene_id	MSTRG.39295;
9	StringTie	exon	21921441	21921560	.	+	.	transcript_	MSTRG.39295.	gene_id	MSTRG.39295;
9	StringTie	exon	21926085	21926135	.	+	.	transcript_	MSTRG.39295.	gene_id	MSTRG.39295;
9	StringTie	exon	21942610	21942709	.	+	.	transcript_	MSTRG.39295.	gene_id	MSTRG.39295;
9	StringTie	exon	21952713	21953702	.	+	.	transcript_	MSTRG.39295.	gene_id	MSTRG.39295;
9	StringTie	transcrip	25763804	25776030	.	+	.	transcript_	MSTRG.39340.	gene_id	MSTRG.39340;
9	StringTie	exon	25763804	25763860	.	+	.	transcript_	MSTRG.39340.	gene_id	MSTRG.39340;
9	StringTie	exon	25775733	25776030	.	+	.	transcript_	MSTRG.39340.	gene_id	MSTRG.39340;
9	StringTie	transcrip	26986221	27004832	.	+	.	transcript_	MSTRG.39365.	gene_id	MSTRG.39365;
9	StringTie	exon	26986221	26986355	.	+	.	transcript_	MSTRG.39365.	gene_id	MSTRG.39365;
9	StringTie	exon	26993312	26993444	.	+	.	transcript_	MSTRG.39365.	gene_id	MSTRG.39365;
9	StringTie	exon	26996492	26996597	.	+	.	transcript_	MSTRG.39365.	gene_id	MSTRG.39365;
9	StringTie	exon	27004708	27004832	.	+	.	transcript_	MSTRG.39365.	gene_id	MSTRG.39365;
9	StringTie	transcrip	28251134	28254554	.	+	.	transcript_	MSTRG.39399.	gene_id	MSTRG.39399;
9	StringTie	exon	28251134	28251957	.	+	.	transcript_	MSTRG.39399.	gene_id	MSTRG.39399;
9	StringTie	exon	28254112	28254554	.	+	.	transcript_	MSTRG.39399.	gene_id	MSTRG.39399;
9	StringTie	transcrip	33061580	33061888	.	+	.	transcript_	MSTRG.39452.	gene_id	MSTRG.39452;
9	StringTie	exon	33061580	33061634	.	+	.	transcript_	MSTRG.39452.	gene_id	MSTRG.39452;
9	StringTie	exon	33061656	33061888	.	+	.	transcript_	MSTRG.39452.	gene_id	MSTRG.39452;
9	StringTie	transcrip	34511351	34549380	.	+	.	transcript_	MSTRG.39471.	gene_id	MSTRG.39471;
9	StringTie	exon	34511351	34511542	.	+	.	transcript_	MSTRG.39471.	gene_id	MSTRG.39471;
9	StringTie	exon	34547142	34549380	.	+	.	transcript_	MSTRG.39471.	gene_id	MSTRG.39471;
9	StringTie	transcrip	35856642	35872637	.	+	.	transcript_	MSTRG.39507.	gene_id	MSTRG.39507;
9	StringTie	exon	35856642	35856847	.	+	.	transcript_	MSTRG.39507.	gene_id	MSTRG.39507;
9	StringTie	exon	35858842	35858997	.	+	.	transcript_	MSTRG.39507.	gene_id	MSTRG.39507;
9	StringTie	exon	35872616	35872637	.	+	.	transcript_	MSTRG.39507.	gene_id	MSTRG.39507;
9	StringTie	transcrip	41997946	42027490	.	+	.	transcript_	MSTRG.39694.	gene_id	MSTRG.39694;

9	StringTie	exon	41997946	41999395	.	+	.	transcript_	MSTRG.39694.1	gene_id	MSTRG.39694;
9	StringTie	exon	42003950	42004004	.	+	.	transcript_	MSTRG.39694.1	gene_id	MSTRG.39694;
9	StringTie	exon	42004715	42004779	.	+	.	transcript_	MSTRG.39694.1	gene_id	MSTRG.39694;
9	StringTie	exon	42017972	42018171	.	+	.	transcript_	MSTRG.39694.1	gene_id	MSTRG.39694;
9	StringTie	exon	42021461	42021601	.	+	.	transcript_	MSTRG.39694.1	gene_id	MSTRG.39694;
9	StringTie	exon	42022788	42027490	.	+	.	transcript_	MSTRG.39694.1	gene_id	MSTRG.39694;
9	StringTie	transcrip	42102948	42111713	.	+	.	transcript_	MSTRG.39708.1	gene_id	MSTRG.39708;
9	StringTie	exon	42102948	42103515	.	+	.	transcript_	MSTRG.39708.1	gene_id	MSTRG.39708;
9	StringTie	exon	42103970	42104504	.	+	.	transcript_	MSTRG.39708.1	gene_id	MSTRG.39708;
9	StringTie	exon	42108219	42108332	.	+	.	transcript_	MSTRG.39708.1	gene_id	MSTRG.39708;
9	StringTie	exon	42111652	42111713	.	+	.	transcript_	MSTRG.39708.1	gene_id	MSTRG.39708;
9	StringTie	transcrip	42103433	42147425	.	+	.	transcript_	MSTRG.39708.3	gene_id	MSTRG.39708;
9	StringTie	exon	42103433	42103515	.	+	.	transcript_	MSTRG.39708.3	gene_id	MSTRG.39708;
9	StringTie	exon	42104279	42104504	.	+	.	transcript_	MSTRG.39708.3	gene_id	MSTRG.39708;
9	StringTie	exon	42108219	42108332	.	+	.	transcript_	MSTRG.39708.3	gene_id	MSTRG.39708;
9	StringTie	exon	42111564	42111734	.	+	.	transcript_	MSTRG.39708.3	gene_id	MSTRG.39708;
9	StringTie	exon	42144407	42144703	.	+	.	transcript_	MSTRG.39708.3	gene_id	MSTRG.39708;
9	StringTie	exon	42146520	42147425	.	+	.	transcript_	MSTRG.39708.3	gene_id	MSTRG.39708;
9	StringTie	transcrip	42103464	42125309	.	+	.	transcript_	MSTRG.39708.4	gene_id	MSTRG.39708;
9	StringTie	exon	42103464	42103515	.	+	.	transcript_	MSTRG.39708.4	gene_id	MSTRG.39708;
9	StringTie	exon	42103970	42104504	.	+	.	transcript_	MSTRG.39708.4	gene_id	MSTRG.39708;
9	StringTie	exon	42108219	42108332	.	+	.	transcript_	MSTRG.39708.4	gene_id	MSTRG.39708;
9	StringTie	exon	42111564	42111734	.	+	.	transcript_	MSTRG.39708.4	gene_id	MSTRG.39708;
9	StringTie	exon	42114015	42114189	.	+	.	transcript_	MSTRG.39708.4	gene_id	MSTRG.39708;
9	StringTie	exon	42124297	42125309	.	+	.	transcript_	MSTRG.39708.4	gene_id	MSTRG.39708;
9	StringTie	transcrip	42103464	42151855	.	+	.	transcript_	MSTRG.39708.5	gene_id	MSTRG.39708;
9	StringTie	exon	42103464	42103515	.	+	.	transcript_	MSTRG.39708.5	gene_id	MSTRG.39708;
9	StringTie	exon	42103970	42104504	.	+	.	transcript_	MSTRG.39708.5	gene_id	MSTRG.39708;
9	StringTie	exon	42108219	42108332	.	+	.	transcript_	MSTRG.39708.5	gene_id	MSTRG.39708;
9	StringTie	exon	42111564	42111734	.	+	.	transcript_	MSTRG.39708.5	gene_id	MSTRG.39708;
9	StringTie	exon	42114015	42114189	.	+	.	transcript_	MSTRG.39708.5	gene_id	MSTRG.39708;
9	StringTie	exon	42149189	42149311	.	+	.	transcript_	MSTRG.39708.5	gene_id	MSTRG.39708;
9	StringTie	exon	42150770	42151855	.	+	.	transcript_	MSTRG.39708.5	gene_id	MSTRG.39708;
9	StringTie	transcrip	42144848	42152121	.	+	.	transcript_	MSTRG.39708.6	gene_id	MSTRG.39708;
9	StringTie	exon	42144848	42144967	.	+	.	transcript_	MSTRG.39708.6	gene_id	MSTRG.39708;

9	StringTie	exon	42149189	42149311	.	+	.	transcript_	MSTRG.39708.6	gene_id	MSTRG.39708;
9	StringTie	exon	42150770	42152121	.	+	.	transcript_	MSTRG.39708.6	gene_id	MSTRG.39708;
9	StringTie	transcrip	44150920	44151295	.	+	.	transcript_	MSTRG.39724.2	gene_id	MSTRG.39724;
9	StringTie	exon	44150920	44151030	.	+	.	transcript_	MSTRG.39724.2	gene_id	MSTRG.39724;
9	StringTie	exon	44151070	44151108	.	+	.	transcript_	MSTRG.39724.2	gene_id	MSTRG.39724;
9	StringTie	exon	44151187	44151295	.	+	.	transcript_	MSTRG.39724.2	gene_id	MSTRG.39724;
9	StringTie	transcrip	44150920	44151315	.	+	.	transcript_	MSTRG.39724.1	gene_id	MSTRG.39724;
9	StringTie	exon	44150920	44151030	.	+	.	transcript_	MSTRG.39724.1	gene_id	MSTRG.39724;
9	StringTie	exon	44151070	44151315	.	+	.	transcript_	MSTRG.39724.1	gene_id	MSTRG.39724;
9	StringTie	transcrip	44151037	44151335	.	+	.	transcript_	MSTRG.39724.3	gene_id	MSTRG.39724;
9	StringTie	exon	44151037	44151147	.	+	.	transcript_	MSTRG.39724.3	gene_id	MSTRG.39724;
9	StringTie	exon	44151226	44151335	.	+	.	transcript_	MSTRG.39724.3	gene_id	MSTRG.39724;
9	StringTie	transcrip	46571839	46580000	.	+	.	transcript_	MSTRG.39810.1	gene_id	MSTRG.39810;
9	StringTie	exon	46571839	46575016	.	+	.	transcript_	MSTRG.39810.1	gene_id	MSTRG.39810;
9	StringTie	exon	46575339	46580000	.	+	.	transcript_	MSTRG.39810.1	gene_id	MSTRG.39810;
9	StringTie	transcrip	46891042	46898219	.	+	.	transcript_	MSTRG.39812.1	gene_id	MSTRG.39812;
9	StringTie	exon	46891042	46891102	.	+	.	transcript_	MSTRG.39812.1	gene_id	MSTRG.39812;
9	StringTie	exon	46895577	46895632	.	+	.	transcript_	MSTRG.39812.1	gene_id	MSTRG.39812;
9	StringTie	exon	46896388	46896482	.	+	.	transcript_	MSTRG.39812.1	gene_id	MSTRG.39812;
9	StringTie	exon	46898109	46898219	.	+	.	transcript_	MSTRG.39812.1	gene_id	MSTRG.39812;
9	StringTie	transcrip	52227162	52228973	.	+	.	transcript_	MSTRG.39938.1	gene_id	MSTRG.39938;
9	StringTie	exon	52227162	52227337	.	+	.	transcript_	MSTRG.39938.1	gene_id	MSTRG.39938;
9	StringTie	exon	52228597	52228973	.	+	.	transcript_	MSTRG.39938.1	gene_id	MSTRG.39938;
9	StringTie	transcrip	52227275	52231264	.	+	.	transcript_	MSTRG.39938.2	gene_id	MSTRG.39938;
9	StringTie	exon	52227275	52227337	.	+	.	transcript_	MSTRG.39938.2	gene_id	MSTRG.39938;
9	StringTie	exon	52230084	52231264	.	+	.	transcript_	MSTRG.39938.2	gene_id	MSTRG.39938;
9	StringTie	transcrip	55544321	55554590	.	+	.	transcript_	MSTRG.40002.1	gene_id	MSTRG.40002;
9	StringTie	exon	55544321	55544443	.	+	.	transcript_	MSTRG.40002.1	gene_id	MSTRG.40002;
9	StringTie	exon	55554336	55554590	.	+	.	transcript_	MSTRG.40002.1	gene_id	MSTRG.40002;
9	StringTie	transcrip	60830108	60834130	.	+	.	transcript_	MSTRG.40096.1	gene_id	MSTRG.40096;
9	StringTie	exon	60830108	60831345	.	+	.	transcript_	MSTRG.40096.1	gene_id	MSTRG.40096;
9	StringTie	exon	60831366	60834130	.	+	.	transcript_	MSTRG.40096.1	gene_id	MSTRG.40096;
9	StringTie	transcrip	67430506	67432529	.	+	.	transcript_	MSTRG.40175.1	gene_id	MSTRG.40175;
9	StringTie	exon	67430506	67430662	.	+	.	transcript_	MSTRG.40175.1	gene_id	MSTRG.40175;
9	StringTie	exon	67431198	67431350	.	+	.	transcript_	MSTRG.40175.1	gene_id	MSTRG.40175;

9	StringTie	exon	67432464	67432529	.	+	.	transcript_	MSTRG.40175.1	gene_id	MSTRG.40175;
9	StringTie	transcrip	74963977	74990214	.	+	.	transcript_	MSTRG.40413.1	gene_id	MSTRG.40413;
9	StringTie	exon	74963977	74964007	.	+	.	transcript_	MSTRG.40413.1	gene_id	MSTRG.40413;
9	StringTie	exon	74989966	74990214	.	+	.	transcript_	MSTRG.40413.1	gene_id	MSTRG.40413;
9	StringTie	transcrip	74963986	75048843	.	+	.	transcript_	MSTRG.40413.2	gene_id	MSTRG.40413;
9	StringTie	exon	74963986	74964007	.	+	.	transcript_	MSTRG.40413.2	gene_id	MSTRG.40413;
9	StringTie	exon	75048412	75048843	.	+	.	transcript_	MSTRG.40413.2	gene_id	MSTRG.40413;
9	StringTie	transcrip	75017670	75048196	.	+	.	transcript_	MSTRG.40415.1	gene_id	MSTRG.40415;
9	StringTie	exon	75017670	75017742	.	+	.	transcript_	MSTRG.40415.1	gene_id	MSTRG.40415;
9	StringTie	exon	75046534	75048196	.	+	.	transcript_	MSTRG.40415.1	gene_id	MSTRG.40415;
9	StringTie	transcrip	75677287	75679659	.	+	.	transcript_	MSTRG.40446.1	gene_id	MSTRG.40446;
9	StringTie	exon	75677287	75679110	.	+	.	transcript_	MSTRG.40446.1	gene_id	MSTRG.40446;
9	StringTie	exon	75679179	75679659	.	+	.	transcript_	MSTRG.40446.1	gene_id	MSTRG.40446;
9	StringTie	transcrip	78387579	78388539	.	+	.	transcript_	MSTRG.40520.1	gene_id	MSTRG.40520;
9	StringTie	exon	78387579	78388200	.	+	.	transcript_	MSTRG.40520.1	gene_id	MSTRG.40520;
9	StringTie	exon	78388487	78388539	.	+	.	transcript_	MSTRG.40520.1	gene_id	MSTRG.40520;
9	StringTie	transcrip	84239887	84248653	.	+	.	transcript_	MSTRG.40562.1	gene_id	MSTRG.40562;
9	StringTie	exon	84239887	84240196	.	+	.	transcript_	MSTRG.40562.1	gene_id	MSTRG.40562;
9	StringTie	exon	84247109	84247167	.	+	.	transcript_	MSTRG.40562.1	gene_id	MSTRG.40562;
9	StringTie	exon	84248341	84248653	.	+	.	transcript_	MSTRG.40562.1	gene_id	MSTRG.40562;
9	StringTie	transcrip	85732984	85733507	.	+	.	transcript_	MSTRG.40592.1	gene_id	MSTRG.40592;
9	StringTie	exon	85732984	85733132	.	+	.	transcript_	MSTRG.40592.1	gene_id	MSTRG.40592;
9	StringTie	exon	85733434	85733507	.	+	.	transcript_	MSTRG.40592.1	gene_id	MSTRG.40592;
9	StringTie	transcrip	87164452	87201015	.	+	.	transcript_	MSTRG.40614.1	gene_id	MSTRG.40614;
9	StringTie	exon	87164452	87164583	.	+	.	transcript_	MSTRG.40614.1	gene_id	MSTRG.40614;
9	StringTie	exon	87179427	87179631	.	+	.	transcript_	MSTRG.40614.1	gene_id	MSTRG.40614;
9	StringTie	exon	87200902	87201015	.	+	.	transcript_	MSTRG.40614.1	gene_id	MSTRG.40614;
9	StringTie	transcrip	91667533	91741969	.	+	.	transcript_	MSTRG.40694.1	gene_id	MSTRG.40694;
9	StringTie	exon	91667533	91667557	.	+	.	transcript_	MSTRG.40694.1	gene_id	MSTRG.40694;
9	StringTie	exon	91740638	91741969	.	+	.	transcript_	MSTRG.40694.1	gene_id	MSTRG.40694;
9	StringTie	transcrip	100281890	100283453	.	+	.	transcript_	MSTRG.40817.1	gene_id	MSTRG.40817;
9	StringTie	exon	100281890	100281974	.	+	.	transcript_	MSTRG.40817.1	gene_id	MSTRG.40817;
9	StringTie	exon	100282524	100283453	.	+	.	transcript_	MSTRG.40817.1	gene_id	MSTRG.40817;
9	StringTie	transcrip	102534657	102589246	.	+	.	transcript_	MSTRG.40937.1	gene_id	MSTRG.40937;
9	StringTie	exon	102534657	102534761	.	+	.	transcript_	MSTRG.40937.1	gene_id	MSTRG.40937;

9	StringTie	exon	102588372	102589246	.	+	.	transcript_	MSTRG.40937.1	gene_id	MSTRG.40937;
9	StringTie	transcrip	105733491	105777344	.	+	.	transcript_	MSTRG.40999.1	gene_id	MSTRG.40999;
9	StringTie	exon	105733491	105733934	.	+	.	transcript_	MSTRG.40999.1	gene_id	MSTRG.40999;
9	StringTie	exon	105774732	105777344	.	+	.	transcript_	MSTRG.40999.1	gene_id	MSTRG.40999;
9	StringTie	transcrip	108686588	108715479	.	+	.	transcript_	MSTRG.41059.1	gene_id	MSTRG.41059;
9	StringTie	exon	108686588	108686669	.	+	.	transcript_	MSTRG.41059.1	gene_id	MSTRG.41059;
9	StringTie	exon	108715320	108715479	.	+	.	transcript_	MSTRG.41059.1	gene_id	MSTRG.41059;
9	StringTie	transcrip	113719580	113720352	.	+	.	transcript_	MSTRG.41092.1	gene_id	MSTRG.41092;
9	StringTie	exon	113719580	113719812	.	+	.	transcript_	MSTRG.41092.1	gene_id	MSTRG.41092;
9	StringTie	exon	113720220	113720352	.	+	.	transcript_	MSTRG.41092.1	gene_id	MSTRG.41092;
9	StringTie	transcrip	116488246	116489566	.	+	.	transcript_	MSTRG.41204.1	gene_id	MSTRG.41204;
9	StringTie	exon	116488246	116488334	.	+	.	transcript_	MSTRG.41204.1	gene_id	MSTRG.41204;
9	StringTie	exon	116489380	116489566	.	+	.	transcript_	MSTRG.41204.1	gene_id	MSTRG.41204;
9	StringTie	transcrip	124514198	124517263	.	+	.	transcript_	MSTRG.41374.1	gene_id	MSTRG.41374;
9	StringTie	exon	124514198	124514256	.	+	.	transcript_	MSTRG.41374.1	gene_id	MSTRG.41374;
9	StringTie	exon	124514625	124514758	.	+	.	transcript_	MSTRG.41374.1	gene_id	MSTRG.41374;
9	StringTie	exon	124516511	124517263	.	+	.	transcript_	MSTRG.41374.1	gene_id	MSTRG.41374;
9	StringTie	transcrip	125338882	125344013	.	+	.	transcript_	MSTRG.41361.1	gene_id	MSTRG.41361;
9	StringTie	exon	125338882	125338950	.	+	.	transcript_	MSTRG.41361.1	gene_id	MSTRG.41361;
9	StringTie	exon	125343150	125343352	.	+	.	transcript_	MSTRG.41361.1	gene_id	MSTRG.41361;
9	StringTie	exon	125343854	125344013	.	+	.	transcript_	MSTRG.41361.1	gene_id	MSTRG.41361;
9	StringTie	transcrip	126286011	126316440	.	+	.	transcript_	MSTRG.41393.2	gene_id	MSTRG.41393;
9	StringTie	exon	126286011	126288248	.	+	.	transcript_	MSTRG.41393.2	gene_id	MSTRG.41393;
9	StringTie	exon	126315814	126316440	.	+	.	transcript_	MSTRG.41393.2	gene_id	MSTRG.41393;
9	StringTie	transcrip	127830724	127846078	.	+	.	transcript_	MSTRG.41411.1	gene_id	MSTRG.41411;
9	StringTie	exon	127830724	127830938	.	+	.	transcript_	MSTRG.41411.1	gene_id	MSTRG.41411;
9	StringTie	exon	127846015	127846078	.	+	.	transcript_	MSTRG.41411.1	gene_id	MSTRG.41411;
9	StringTie	transcrip	129041180	129042410	.	+	.	transcript_	MSTRG.41447.1	gene_id	MSTRG.41447;
9	StringTie	exon	129041180	129041598	.	+	.	transcript_	MSTRG.41447.1	gene_id	MSTRG.41447;
9	StringTie	exon	129042370	129042410	.	+	.	transcript_	MSTRG.41447.1	gene_id	MSTRG.41447;
9	StringTie	transcrip	131074709	131126387	.	+	.	transcript_	MSTRG.41496.1	gene_id	MSTRG.41496;
9	StringTie	exon	131074709	131075157	.	+	.	transcript_	MSTRG.41496.1	gene_id	MSTRG.41496;
9	StringTie	exon	131124494	131124755	.	+	.	transcript_	MSTRG.41496.1	gene_id	MSTRG.41496;
9	StringTie	exon	131125209	131126387	.	+	.	transcript_	MSTRG.41496.1	gene_id	MSTRG.41496;
9	StringTie	transcrip	131110505	131124788	.	+	.	transcript_	MSTRG.41496.2	gene_id	MSTRG.41496;

9	StringTie	exon	131110505	131110732	.	+	.	transcript_	MSTRG.41496.2	gene_id	MSTRG.41496;
9	StringTie	exon	131124494	131124788	.	+	.	transcript_	MSTRG.41496.2	gene_id	MSTRG.41496;
9	StringTie	transcrip	131891310	131903296	.	+	.	transcript_	MSTRG.41519.1	gene_id	MSTRG.41519;
9	StringTie	exon	131891310	131891746	.	+	.	transcript_	MSTRG.41519.1	gene_id	MSTRG.41519;
9	StringTie	exon	131892075	131892248	.	+	.	transcript_	MSTRG.41519.1	gene_id	MSTRG.41519;
9	StringTie	exon	131894097	131894252	.	+	.	transcript_	MSTRG.41519.1	gene_id	MSTRG.41519;
9	StringTie	exon	131900186	131903296	.	+	.	transcript_	MSTRG.41519.1	gene_id	MSTRG.41519;
9	StringTie	transcrip	131891317	131903989	.	+	.	transcript_	MSTRG.41519.2	gene_id	MSTRG.41519;
9	StringTie	exon	131891317	131892248	.	+	.	transcript_	MSTRG.41519.2	gene_id	MSTRG.41519;
9	StringTie	exon	131894097	131894252	.	+	.	transcript_	MSTRG.41519.2	gene_id	MSTRG.41519;
9	StringTie	exon	131900186	131902031	.	+	.	transcript_	MSTRG.41519.2	gene_id	MSTRG.41519;
9	StringTie	exon	131903894	131903989	.	+	.	transcript_	MSTRG.41519.2	gene_id	MSTRG.41519;
9	StringTie	transcrip	131891361	131902938	.	+	.	transcript_	MSTRG.41519.3	gene_id	MSTRG.41519;
9	StringTie	exon	131891361	131891509	.	+	.	transcript_	MSTRG.41519.3	gene_id	MSTRG.41519;
9	StringTie	exon	131892075	131892248	.	+	.	transcript_	MSTRG.41519.3	gene_id	MSTRG.41519;
9	StringTie	exon	131894097	131894252	.	+	.	transcript_	MSTRG.41519.3	gene_id	MSTRG.41519;
9	StringTie	exon	131900186	131902938	.	+	.	transcript_	MSTRG.41519.3	gene_id	MSTRG.41519;
9	StringTie	transcrip	131892105	131895861	.	+	.	transcript_	MSTRG.41519.4	gene_id	MSTRG.41519;
9	StringTie	exon	131892105	131892248	.	+	.	transcript_	MSTRG.41519.4	gene_id	MSTRG.41519;
9	StringTie	exon	131894097	131894252	.	+	.	transcript_	MSTRG.41519.4	gene_id	MSTRG.41519;
9	StringTie	exon	131895763	131895861	.	+	.	transcript_	MSTRG.41519.4	gene_id	MSTRG.41519;
9	StringTie	transcrip	139169935	139186605	.	+	.	transcript_	MSTRG.41591.1	gene_id	MSTRG.41591;
9	StringTie	exon	139169935	139170244	.	+	.	transcript_	MSTRG.41591.1	gene_id	MSTRG.41591;
9	StringTie	exon	139185907	139186605	.	+	.	transcript_	MSTRG.41591.1	gene_id	MSTRG.41591;
9	StringTie	transcrip	2876972	3063801	.	-	.	transcript_	MSTRG.38842.1	gene_id	MSTRG.38842;
9	StringTie	exon	2876972	2877078	.	-	.	transcript_	MSTRG.38842.1	gene_id	MSTRG.38842;
9	StringTie	exon	2974784	2974814	.	-	.	transcript_	MSTRG.38842.1	gene_id	MSTRG.38842;
9	StringTie	exon	3063705	3063801	.	-	.	transcript_	MSTRG.38842.1	gene_id	MSTRG.38842;
9	StringTie	transcrip	3339665	3340833	.	-	.	transcript_	MSTRG.38879.3	gene_id	MSTRG.38879;
9	StringTie	exon	3339665	3340164	.	-	.	transcript_	MSTRG.38879.3	gene_id	MSTRG.38879;
9	StringTie	exon	3340722	3340833	.	-	.	transcript_	MSTRG.38879.3	gene_id	MSTRG.38879;
9	StringTie	transcrip	3803701	3805933	.	-	.	transcript_	MSTRG.38889.1	gene_id	MSTRG.38889;
9	StringTie	exon	3803701	3803784	.	-	.	transcript_	MSTRG.38889.1	gene_id	MSTRG.38889;
9	StringTie	exon	3805010	3805933	.	-	.	transcript_	MSTRG.38889.1	gene_id	MSTRG.38889;
9	StringTie	transcrip	9932852	9935798	.	-	.	transcript_	MSTRG.39037.1	gene_id	MSTRG.39037;

9	StringTie	exon	9932852	9933061	.	-	.	transcript_	MSTRG.39037.1	gene_id	MSTRG.39037;
9	StringTie	exon	9935641	9935798	.	-	.	transcript_	MSTRG.39037.1	gene_id	MSTRG.39037;
9	StringTie	transcrip	9934250	9990161	.	-	.	transcript_	MSTRG.39038.1	gene_id	MSTRG.39038;
9	StringTie	exon	9934250	9934295	.	-	.	transcript_	MSTRG.39038.1	gene_id	MSTRG.39038;
9	StringTie	exon	9989824	9990161	.	-	.	transcript_	MSTRG.39038.1	gene_id	MSTRG.39038;
9	StringTie	transcrip	9935975	10003157	.	-	.	transcript_	MSTRG.39040.2	gene_id	MSTRG.39040;
9	StringTie	exon	9935975	9936451	.	-	.	transcript_	MSTRG.39040.2	gene_id	MSTRG.39040;
9	StringTie	exon	9997870	9998050	.	-	.	transcript_	MSTRG.39040.2	gene_id	MSTRG.39040;
9	StringTie	exon	10003058	10003157	.	-	.	transcript_	MSTRG.39040.2	gene_id	MSTRG.39040;
9	StringTie	transcrip	9935975	10013550	.	-	.	transcript_	MSTRG.39040.1	gene_id	MSTRG.39040;
9	StringTie	exon	9935975	9936466	.	-	.	transcript_	MSTRG.39040.1	gene_id	MSTRG.39040;
9	StringTie	exon	9997870	9998050	.	-	.	transcript_	MSTRG.39040.1	gene_id	MSTRG.39040;
9	StringTie	exon	10003058	10003175	.	-	.	transcript_	MSTRG.39040.1	gene_id	MSTRG.39040;
9	StringTie	exon	10013470	10013550	.	-	.	transcript_	MSTRG.39040.1	gene_id	MSTRG.39040;
9	StringTie	transcrip	9949537	9950589	.	-	.	transcript_	MSTRG.39040.3	gene_id	MSTRG.39040;
9	StringTie	exon	9949537	9949687	.	-	.	transcript_	MSTRG.39040.3	gene_id	MSTRG.39040;
9	StringTie	exon	9950447	9950589	.	-	.	transcript_	MSTRG.39040.3	gene_id	MSTRG.39040;
9	StringTie	transcrip	9949540	9950591	.	-	.	transcript_	MSTRG.39040.4	gene_id	MSTRG.39040;
9	StringTie	exon	9949540	9949687	.	-	.	transcript_	MSTRG.39040.4	gene_id	MSTRG.39040;
9	StringTie	exon	9950125	9950591	.	-	.	transcript_	MSTRG.39040.4	gene_id	MSTRG.39040;
9	StringTie	transcrip	9949548	10013547	.	-	.	transcript_	MSTRG.39040.5	gene_id	MSTRG.39040;
9	StringTie	exon	9949548	9949687	.	-	.	transcript_	MSTRG.39040.5	gene_id	MSTRG.39040;
9	StringTie	exon	9949767	9950588	.	-	.	transcript_	MSTRG.39040.5	gene_id	MSTRG.39040;
9	StringTie	exon	10009519	10009740	.	-	.	transcript_	MSTRG.39040.5	gene_id	MSTRG.39040;
9	StringTie	exon	10013093	10013547	.	-	.	transcript_	MSTRG.39040.5	gene_id	MSTRG.39040;
9	StringTie	transcrip	9949555	9950591	.	-	.	transcript_	MSTRG.39040.6	gene_id	MSTRG.39040;
9	StringTie	exon	9949555	9949687	.	-	.	transcript_	MSTRG.39040.6	gene_id	MSTRG.39040;
9	StringTie	exon	9950125	9950221	.	-	.	transcript_	MSTRG.39040.6	gene_id	MSTRG.39040;
9	StringTie	exon	9950447	9950591	.	-	.	transcript_	MSTRG.39040.6	gene_id	MSTRG.39040;
9	StringTie	transcrip	9997356	10003157	.	-	.	transcript_	MSTRG.39040.8	gene_id	MSTRG.39040;
9	StringTie	exon	9997356	9997483	.	-	.	transcript_	MSTRG.39040.8	gene_id	MSTRG.39040;
9	StringTie	exon	9997870	9998050	.	-	.	transcript_	MSTRG.39040.8	gene_id	MSTRG.39040;
9	StringTie	exon	10003058	10003157	.	-	.	transcript_	MSTRG.39040.8	gene_id	MSTRG.39040;
9	StringTie	transcrip	10007082	10013547	.	-	.	transcript_	MSTRG.39040.9	gene_id	MSTRG.39040;
9	StringTie	exon	10007082	10008197	.	-	.	transcript_	MSTRG.39040.9	gene_id	MSTRG.39040;

9	StringTie	exon	10009519	10009740	.	-	.	transcript_	MSTRG.39040.9	gene_id	MSTRG.39040;
9	StringTie	exon	10013470	10013547	.	-	.	transcript_	MSTRG.39040.9	gene_id	MSTRG.39040;
9	StringTie	transcrip	10007084	10013550	.	-	.	transcript_	MSTRG.39040.1	gene_id	MSTRG.39040;
9	StringTie	exon	10007084	10007295	.	-	.	transcript_	MSTRG.39040.1	gene_id	MSTRG.39040;
9	StringTie	exon	10007734	10008197	.	-	.	transcript_	MSTRG.39040.1	gene_id	MSTRG.39040;
9	StringTie	exon	10013470	10013550	.	-	.	transcript_	MSTRG.39040.1	gene_id	MSTRG.39040;
9	StringTie	transcrip	10007084	10013569	.	-	.	transcript_	MSTRG.39040.1	gene_id	MSTRG.39040;
9	StringTie	exon	10007084	10007295	.	-	.	transcript_	MSTRG.39040.1	gene_id	MSTRG.39040;
9	StringTie	exon	10007734	10008197	.	-	.	transcript_	MSTRG.39040.1	gene_id	MSTRG.39040;
9	StringTie	exon	10009519	10009740	.	-	.	transcript_	MSTRG.39040.1	gene_id	MSTRG.39040;
9	StringTie	exon	10011020	10011087	.	-	.	transcript_	MSTRG.39040.1	gene_id	MSTRG.39040;
9	StringTie	exon	10013470	10013569	.	-	.	transcript_	MSTRG.39040.1	gene_id	MSTRG.39040;
9	StringTie	transcrip	11145542	11172785	.	-	.	transcript_	MSTRG.39081.1	gene_id	MSTRG.39081;
9	StringTie	exon	11145542	11146918	.	-	.	transcript_	MSTRG.39081.1	gene_id	MSTRG.39081;
9	StringTie	exon	11171741	11172785	.	-	.	transcript_	MSTRG.39081.1	gene_id	MSTRG.39081;
9	StringTie	transcrip	12652827	12656714	.	-	.	transcript_	MSTRG.39119.1	gene_id	MSTRG.39119;
9	StringTie	exon	12652827	12652905	.	-	.	transcript_	MSTRG.39119.1	gene_id	MSTRG.39119;
9	StringTie	exon	12656063	12656714	.	-	.	transcript_	MSTRG.39119.1	gene_id	MSTRG.39119;
9	StringTie	transcrip	20650795	20704693	.	-	.	transcript_	MSTRG.39262.1	gene_id	MSTRG.39262;
9	StringTie	exon	20650795	20650820	.	-	.	transcript_	MSTRG.39262.1	gene_id	MSTRG.39262;
9	StringTie	exon	20702051	20704693	.	-	.	transcript_	MSTRG.39262.1	gene_id	MSTRG.39262;
9	StringTie	transcrip	20931767	20933450	.	-	.	transcript_	MSTRG.39283.1	gene_id	MSTRG.39283;
9	StringTie	exon	20931767	20932882	.	-	.	transcript_	MSTRG.39283.1	gene_id	MSTRG.39283;
9	StringTie	exon	20933165	20933450	.	-	.	transcript_	MSTRG.39283.1	gene_id	MSTRG.39283;
9	StringTie	transcrip	21023789	21029111	.	-	.	transcript_	MSTRG.39284.2	gene_id	MSTRG.39284;
9	StringTie	exon	21023789	21029111	.	-	.	transcript_	MSTRG.39284.2	gene_id	MSTRG.39284;
9	StringTie	transcrip	26882129	26976504	.	-	.	transcript_	MSTRG.39379.1	gene_id	MSTRG.39379;
9	StringTie	exon	26882129	26882321	.	-	.	transcript_	MSTRG.39379.1	gene_id	MSTRG.39379;
9	StringTie	exon	26976404	26976504	.	-	.	transcript_	MSTRG.39379.1	gene_id	MSTRG.39379;
9	StringTie	transcrip	27118484	27122137	.	-	.	transcript_	MSTRG.39371.1	gene_id	MSTRG.39371;
9	StringTie	exon	27118484	27118705	.	-	.	transcript_	MSTRG.39371.1	gene_id	MSTRG.39371;
9	StringTie	exon	27122073	27122137	.	-	.	transcript_	MSTRG.39371.1	gene_id	MSTRG.39371;
9	StringTie	transcrip	35782501	35786587	.	-	.	transcript_	MSTRG.39498.1	gene_id	MSTRG.39498;
9	StringTie	exon	35782501	35782862	.	-	.	transcript_	MSTRG.39498.1	gene_id	MSTRG.39498;
9	StringTie	exon	35786500	35786587	.	-	.	transcript_	MSTRG.39498.1	gene_id	MSTRG.39498;

9	StringTie	transcrip	36849672	36855068	.	-	.	transcript_	MSTRG.39550.1	gene_id	MSTRG.39550;
9	StringTie	exon	36849672	36849965	.	-	.	transcript_	MSTRG.39550.1	gene_id	MSTRG.39550;
9	StringTie	exon	36854910	36855068	.	-	.	transcript_	MSTRG.39550.1	gene_id	MSTRG.39550;
9	StringTie	transcrip	38315783	38318611	.	-	.	transcript_	MSTRG.39578.1	gene_id	MSTRG.39578;
9	StringTie	exon	38315783	38316211	.	-	.	transcript_	MSTRG.39578.1	gene_id	MSTRG.39578;
9	StringTie	exon	38316587	38316778	.	-	.	transcript_	MSTRG.39578.1	gene_id	MSTRG.39578;
9	StringTie	exon	38318461	38318611	.	-	.	transcript_	MSTRG.39578.1	gene_id	MSTRG.39578;
9	StringTie	transcrip	39457004	39464386	.	-	.	transcript_	MSTRG.39628.1	gene_id	MSTRG.39628;
9	StringTie	exon	39457004	39457030	.	-	.	transcript_	MSTRG.39628.1	gene_id	MSTRG.39628;
9	StringTie	exon	39463709	39464386	.	-	.	transcript_	MSTRG.39628.1	gene_id	MSTRG.39628;
9	StringTie	transcrip	39543616	39545797	.	-	.	transcript_	MSTRG.39629.1	gene_id	MSTRG.39629;
9	StringTie	exon	39543616	39543648	.	-	.	transcript_	MSTRG.39629.1	gene_id	MSTRG.39629;
9	StringTie	exon	39545483	39545797	.	-	.	transcript_	MSTRG.39629.1	gene_id	MSTRG.39629;
9	StringTie	transcrip	41625084	41639431	.	-	.	transcript_	MSTRG.39669.1	gene_id	MSTRG.39669;
9	StringTie	exon	41625084	41626120	.	-	.	transcript_	MSTRG.39669.1	gene_id	MSTRG.39669;
9	StringTie	exon	41632309	41632589	.	-	.	transcript_	MSTRG.39669.1	gene_id	MSTRG.39669;
9	StringTie	exon	41636157	41636225	.	-	.	transcript_	MSTRG.39669.1	gene_id	MSTRG.39669;
9	StringTie	exon	41639292	41639431	.	-	.	transcript_	MSTRG.39669.1	gene_id	MSTRG.39669;
9	StringTie	transcrip	41631442	41636212	.	-	.	transcript_	MSTRG.39669.2	gene_id	MSTRG.39669;
9	StringTie	exon	41631442	41631559	.	-	.	transcript_	MSTRG.39669.2	gene_id	MSTRG.39669;
9	StringTie	exon	41632309	41632589	.	-	.	transcript_	MSTRG.39669.2	gene_id	MSTRG.39669;
9	StringTie	exon	41636157	41636212	.	-	.	transcript_	MSTRG.39669.2	gene_id	MSTRG.39669;
9	StringTie	transcrip	41632307	41638798	.	-	.	transcript_	MSTRG.39669.3	gene_id	MSTRG.39669;
9	StringTie	exon	41632307	41633336	.	-	.	transcript_	MSTRG.39669.3	gene_id	MSTRG.39669;
9	StringTie	exon	41636157	41636225	.	-	.	transcript_	MSTRG.39669.3	gene_id	MSTRG.39669;
9	StringTie	exon	41638166	41638798	.	-	.	transcript_	MSTRG.39669.3	gene_id	MSTRG.39669;
9	StringTie	transcrip	41681800	41686100	.	-	.	transcript_	MSTRG.39671.1	gene_id	MSTRG.39671;
9	StringTie	exon	41681800	41682328	.	-	.	transcript_	MSTRG.39671.1	gene_id	MSTRG.39671;
9	StringTie	exon	41685726	41686100	.	-	.	transcript_	MSTRG.39671.1	gene_id	MSTRG.39671;
9	StringTie	transcrip	41807649	41839511	.	-	.	transcript_	MSTRG.39672.1	gene_id	MSTRG.39672;
9	StringTie	exon	41807649	41811027	.	-	.	transcript_	MSTRG.39672.1	gene_id	MSTRG.39672;
9	StringTie	exon	41837740	41839511	.	-	.	transcript_	MSTRG.39672.1	gene_id	MSTRG.39672;
9	StringTie	transcrip	42137524	42141853	.	-	.	transcript_	MSTRG.39710.1	gene_id	MSTRG.39710;
9	StringTie	exon	42137524	42137581	.	-	.	transcript_	MSTRG.39710.1	gene_id	MSTRG.39710;
9	StringTie	exon	42141623	42141853	.	-	.	transcript_	MSTRG.39710.1	gene_id	MSTRG.39710;

9	StringTie	transcrip	42617017	42620121	.	-	.	transcript_	MSTRG.39716.	gene_id	MSTRG.39716;
9	StringTie	exon	42617017	42617913	.	-	.	transcript_	MSTRG.39716.	gene_id	MSTRG.39716;
9	StringTie	exon	42619966	42620121	.	-	.	transcript_	MSTRG.39716.	gene_id	MSTRG.39716;
9	StringTie	transcrip	43220727	43230994	.	-	.	transcript_	MSTRG.39719.	gene_id	MSTRG.39719;
9	StringTie	exon	43220727	43223789	.	-	.	transcript_	MSTRG.39719.	gene_id	MSTRG.39719;
9	StringTie	exon	43230625	43230994	.	-	.	transcript_	MSTRG.39719.	gene_id	MSTRG.39719;
9	StringTie	transcrip	52743919	52758923	.	-	.	transcript_	MSTRG.39949.	gene_id	MSTRG.39949;
9	StringTie	exon	52743919	52744363	.	-	.	transcript_	MSTRG.39949.	gene_id	MSTRG.39949;
9	StringTie	exon	52754869	52758923	.	-	.	transcript_	MSTRG.39949.	gene_id	MSTRG.39949;
9	StringTie	transcrip	57544333	57544769	.	-	.	transcript_	MSTRG.40059.	gene_id	MSTRG.40059;
9	StringTie	exon	57544333	57544527	.	-	.	transcript_	MSTRG.40059.	gene_id	MSTRG.40059;
9	StringTie	exon	57544629	57544769	.	-	.	transcript_	MSTRG.40059.	gene_id	MSTRG.40059;
9	StringTie	transcrip	64863700	64869140	.	-	.	transcript_	MSTRG.40122.	gene_id	MSTRG.40122;
9	StringTie	exon	64863700	64867696	.	-	.	transcript_	MSTRG.40122.	gene_id	MSTRG.40122;
9	StringTie	exon	64868384	64869140	.	-	.	transcript_	MSTRG.40122.	gene_id	MSTRG.40122;
9	StringTie	transcrip	67137460	67156366	.	-	.	transcript_	MSTRG.40166.	gene_id	MSTRG.40166;
9	StringTie	exon	67137460	67137666	.	-	.	transcript_	MSTRG.40166.	gene_id	MSTRG.40166;
9	StringTie	exon	67137871	67137961	.	-	.	transcript_	MSTRG.40166.	gene_id	MSTRG.40166;
9	StringTie	exon	67156194	67156366	.	-	.	transcript_	MSTRG.40166.	gene_id	MSTRG.40166;
9	StringTie	transcrip	71372342	71598824	.	-	.	transcript_	MSTRG.40264.	gene_id	MSTRG.40264;
9	StringTie	exon	71372342	71372452	.	-	.	transcript_	MSTRG.40264.	gene_id	MSTRG.40264;
9	StringTie	exon	71420907	71421085	.	-	.	transcript_	MSTRG.40264.	gene_id	MSTRG.40264;
9	StringTie	exon	71598786	71598824	.	-	.	transcript_	MSTRG.40264.	gene_id	MSTRG.40264;
9	StringTie	transcrip	74547449	74570858	.	-	.	transcript_	MSTRG.40392.	gene_id	MSTRG.40392;
9	StringTie	exon	74547449	74548195	.	-	.	transcript_	MSTRG.40392.	gene_id	MSTRG.40392;
9	StringTie	exon	74557279	74557492	.	-	.	transcript_	MSTRG.40392.	gene_id	MSTRG.40392;
9	StringTie	exon	74570812	74570858	.	-	.	transcript_	MSTRG.40392.	gene_id	MSTRG.40392;
9	StringTie	transcrip	78063970	78219087	.	-	.	transcript_	MSTRG.40505.	gene_id	MSTRG.40505;
9	StringTie	exon	78063970	78064007	.	-	.	transcript_	MSTRG.40505.	gene_id	MSTRG.40505;
9	StringTie	exon	78218733	78219087	.	-	.	transcript_	MSTRG.40505.	gene_id	MSTRG.40505;
9	StringTie	transcrip	81785200	81787325	.	-	.	transcript_	MSTRG.40543.	gene_id	MSTRG.40543;
9	StringTie	exon	81785200	81786165	.	-	.	transcript_	MSTRG.40543.	gene_id	MSTRG.40543;
9	StringTie	exon	81787132	81787325	.	-	.	transcript_	MSTRG.40543.	gene_id	MSTRG.40543;
9	StringTie	transcrip	85311171	85318441	.	-	.	transcript_	MSTRG.40581.	gene_id	MSTRG.40581;
9	StringTie	exon	85311171	85311508	.	-	.	transcript_	MSTRG.40581.	gene_id	MSTRG.40581;

9	StringTie	exon	85318377	85318441	.	-	.	transcript_	MSTRG.40581.1	gene_id	MSTRG.40581;
9	StringTie	transcrip	85377473	85421316	.	-	.	transcript_	MSTRG.40593.2	gene_id	MSTRG.40593;
9	StringTie	exon	85377473	85377848	.	-	.	transcript_	MSTRG.40593.2	gene_id	MSTRG.40593;
9	StringTie	exon	85418943	85421316	.	-	.	transcript_	MSTRG.40593.2	gene_id	MSTRG.40593;
9	StringTie	transcrip	88116184	88279484	.	-	.	transcript_	MSTRG.40657.2	gene_id	MSTRG.40657;
9	StringTie	exon	88116184	88118872	.	-	.	transcript_	MSTRG.40657.2	gene_id	MSTRG.40657;
9	StringTie	exon	88168738	88168835	.	-	.	transcript_	MSTRG.40657.2	gene_id	MSTRG.40657;
9	StringTie	exon	88279181	88279484	.	-	.	transcript_	MSTRG.40657.2	gene_id	MSTRG.40657;
9	StringTie	transcrip	88116590	88279508	.	-	.	transcript_	MSTRG.40657.3	gene_id	MSTRG.40657;
9	StringTie	exon	88116590	88119279	.	-	.	transcript_	MSTRG.40657.3	gene_id	MSTRG.40657;
9	StringTie	exon	88168738	88168835	.	-	.	transcript_	MSTRG.40657.3	gene_id	MSTRG.40657;
9	StringTie	exon	88178970	88179094	.	-	.	transcript_	MSTRG.40657.3	gene_id	MSTRG.40657;
9	StringTie	exon	88279181	88279508	.	-	.	transcript_	MSTRG.40657.3	gene_id	MSTRG.40657;
9	StringTie	transcrip	88118569	88278297	.	-	.	transcript_	MSTRG.40657.5	gene_id	MSTRG.40657;
9	StringTie	exon	88118569	88119279	.	-	.	transcript_	MSTRG.40657.5	gene_id	MSTRG.40657;
9	StringTie	exon	88168738	88168835	.	-	.	transcript_	MSTRG.40657.5	gene_id	MSTRG.40657;
9	StringTie	exon	88178970	88179094	.	-	.	transcript_	MSTRG.40657.5	gene_id	MSTRG.40657;
9	StringTie	exon	88278260	88278297	.	-	.	transcript_	MSTRG.40657.5	gene_id	MSTRG.40657;
9	StringTie	transcrip	93365360	93437518	.	-	.	transcript_	MSTRG.40736.1	gene_id	MSTRG.40736;
9	StringTie	exon	93365360	93365398	.	-	.	transcript_	MSTRG.40736.1	gene_id	MSTRG.40736;
9	StringTie	exon	93433915	93437518	.	-	.	transcript_	MSTRG.40736.1	gene_id	MSTRG.40736;
9	StringTie	transcrip	101312352	101312829	.	-	.	transcript_	MSTRG.40910.1	gene_id	MSTRG.40910;
9	StringTie	exon	101312352	101312456	.	-	.	transcript_	MSTRG.40910.1	gene_id	MSTRG.40910;
9	StringTie	exon	101312493	101312829	.	-	.	transcript_	MSTRG.40910.1	gene_id	MSTRG.40910;
9	StringTie	transcrip	105794287	105907571	.	-	.	transcript_	MSTRG.40985.1	gene_id	MSTRG.40985;
9	StringTie	exon	105794287	105794325	.	-	.	transcript_	MSTRG.40985.1	gene_id	MSTRG.40985;
9	StringTie	exon	105838629	105838741	.	-	.	transcript_	MSTRG.40985.1	gene_id	MSTRG.40985;
9	StringTie	exon	105907230	105907571	.	-	.	transcript_	MSTRG.40985.1	gene_id	MSTRG.40985;
9	StringTie	transcrip	107446488	107456349	.	-	.	transcript_	MSTRG.41024.1	gene_id	MSTRG.41024;
9	StringTie	exon	107446488	107446794	.	-	.	transcript_	MSTRG.41024.1	gene_id	MSTRG.41024;
9	StringTie	exon	107456268	107456349	.	-	.	transcript_	MSTRG.41024.1	gene_id	MSTRG.41024;
9	StringTie	transcrip	116244477	116245065	.	-	.	transcript_	MSTRG.41179.1	gene_id	MSTRG.41179;
9	StringTie	exon	116244477	116244694	.	-	.	transcript_	MSTRG.41179.1	gene_id	MSTRG.41179;
9	StringTie	exon	116245026	116245065	.	-	.	transcript_	MSTRG.41179.1	gene_id	MSTRG.41179;
9	StringTie	transcrip	123591395	123620136	.	-	.	transcript_	MSTRG.41313.1	gene_id	MSTRG.41313;

9	StringTie	exon	123591395	123591451	.	-	.	transcript_	MSTRG.41313.1	gene_id	MSTRG.41313;
9	StringTie	exon	123619560	123619617	.	-	.	transcript_	MSTRG.41313.1	gene_id	MSTRG.41313;
9	StringTie	exon	123619964	123620136	.	-	.	transcript_	MSTRG.41313.1	gene_id	MSTRG.41313;
9	StringTie	transcrip	123620782	123627495	.	-	.	transcript_	MSTRG.41315.1	gene_id	MSTRG.41315;
9	StringTie	exon	123620782	123620927	.	-	.	transcript_	MSTRG.41315.1	gene_id	MSTRG.41315;
9	StringTie	exon	123627252	123627495	.	-	.	transcript_	MSTRG.41315.1	gene_id	MSTRG.41315;
9	StringTie	transcrip	123627793	123630459	.	-	.	transcript_	MSTRG.41307.1	gene_id	MSTRG.41307;
9	StringTie	exon	123627793	123628195	.	-	.	transcript_	MSTRG.41307.1	gene_id	MSTRG.41307;
9	StringTie	exon	123629999	123630459	.	-	.	transcript_	MSTRG.41307.1	gene_id	MSTRG.41307;
9	StringTie	transcrip	124447063	124488317	.	-	.	transcript_	MSTRG.41350.1	gene_id	MSTRG.41350;
9	StringTie	exon	124447063	124447087	.	-	.	transcript_	MSTRG.41350.1	gene_id	MSTRG.41350;
9	StringTie	exon	124486225	124487047	.	-	.	transcript_	MSTRG.41350.1	gene_id	MSTRG.41350;
9	StringTie	exon	124488050	124488317	.	-	.	transcript_	MSTRG.41350.1	gene_id	MSTRG.41350;
9	StringTie	transcrip	124534569	124536903	.	-	.	transcript_	MSTRG.41375.1	gene_id	MSTRG.41375;
9	StringTie	exon	124534569	124534636	.	-	.	transcript_	MSTRG.41375.1	gene_id	MSTRG.41375;
9	StringTie	exon	124535531	124536903	.	-	.	transcript_	MSTRG.41375.1	gene_id	MSTRG.41375;
9	StringTie	transcrip	124534569	124538749	.	-	.	transcript_	MSTRG.41375.2	gene_id	MSTRG.41375;
9	StringTie	exon	124534569	124535762	.	-	.	transcript_	MSTRG.41375.2	gene_id	MSTRG.41375;
9	StringTie	exon	124538688	124538749	.	-	.	transcript_	MSTRG.41375.2	gene_id	MSTRG.41375;
9	StringTie	transcrip	130519236	130541587	.	-	.	transcript_	MSTRG.41457.1	gene_id	MSTRG.41457;
9	StringTie	exon	130519236	130519257	.	-	.	transcript_	MSTRG.41457.1	gene_id	MSTRG.41457;
9	StringTie	exon	130541258	130541587	.	-	.	transcript_	MSTRG.41457.1	gene_id	MSTRG.41457;
AEMK	StringTie	transcrip	63398	89423	.	-	.	transcript_	MSTRG.41613.1	gene_id	MSTRG.41613;
AEMK	StringTie	exon	63398	63415	.	-	.	transcript_	MSTRG.41613.1	gene_id	MSTRG.41613;
AEMK	StringTie	exon	88986	89423	.	-	.	transcript_	MSTRG.41613.1	gene_id	MSTRG.41613;
AEMK	StringTie	transcrip	1106	7745	.	-	.	transcript_	MSTRG.41644.1	gene_id	MSTRG.41644;
AEMK	StringTie	exon	1106	1267	.	-	.	transcript_	MSTRG.41644.1	gene_id	MSTRG.41644;
AEMK	StringTie	exon	7668	7745	.	-	.	transcript_	MSTRG.41644.1	gene_id	MSTRG.41644;
AEMK	StringTie	transcrip	7291	7744	.	-	.	transcript_	MSTRG.41644.2	gene_id	MSTRG.41644;
AEMK	StringTie	exon	7291	7513	.	-	.	transcript_	MSTRG.41644.2	gene_id	MSTRG.41644;
AEMK	StringTie	exon	7668	7744	.	-	.	transcript_	MSTRG.41644.2	gene_id	MSTRG.41644;
AEMK	StringTie	transcrip	15683	17619	.	+	.	transcript_	MSTRG.41654.1	gene_id	MSTRG.41654;
AEMK	StringTie	exon	15683	15851	.	+	.	transcript_	MSTRG.41654.1	gene_id	MSTRG.41654;
AEMK	StringTie	exon	17030	17619	.	+	.	transcript_	MSTRG.41654.1	gene_id	MSTRG.41654;
AEMK	StringTie	transcrip	2339	5679	.	-	.	transcript_	MSTRG.41713.1	gene_id	MSTRG.41713;

AEMK	StringTie	exon	2339	2501	.	-	.	transcript_	MSTRG.41713.1	gene_id	MSTRG.41713;
AEMK	StringTie	exon	4253	4333	.	-	.	transcript_	MSTRG.41713.1	gene_id	MSTRG.41713;
AEMK	StringTie	exon	5620	5679	.	-	.	transcript_	MSTRG.41713.1	gene_id	MSTRG.41713;
AEMK	StringTie	transcrip	15190	16664	.	-	.	transcript_	MSTRG.41732.1	gene_id	MSTRG.41732;
AEMK	StringTie	exon	15190	16004	.	-	.	transcript_	MSTRG.41732.1	gene_id	MSTRG.41732;
AEMK	StringTie	exon	16576	16664	.	-	.	transcript_	MSTRG.41732.1	gene_id	MSTRG.41732;
AEMK	StringTie	transcrip	326889	328962	.	-	.	transcript_	MSTRG.41775.2	gene_id	MSTRG.41775;
AEMK	StringTie	exon	326889	326929	.	-	.	transcript_	MSTRG.41775.2	gene_id	MSTRG.41775;
AEMK	StringTie	exon	327871	328962	.	-	.	transcript_	MSTRG.41775.2	gene_id	MSTRG.41775;
AEMK	StringTie	transcrip	10834	14297	.	-	.	transcript_	MSTRG.41778.1	gene_id	MSTRG.41778;
AEMK	StringTie	exon	10834	13649	.	-	.	transcript_	MSTRG.41778.1	gene_id	MSTRG.41778;
AEMK	StringTie	exon	14238	14297	.	-	.	transcript_	MSTRG.41778.1	gene_id	MSTRG.41778;
AEMK	StringTie	transcrip	20264	27991	.	-	.	transcript_	MSTRG.41820.1	gene_id	MSTRG.41820;
AEMK	StringTie	exon	20264	23729	.	-	.	transcript_	MSTRG.41820.1	gene_id	MSTRG.41820;
AEMK	StringTie	exon	24319	24503	.	-	.	transcript_	MSTRG.41820.1	gene_id	MSTRG.41820;
AEMK	StringTie	exon	27595	27991	.	-	.	transcript_	MSTRG.41820.1	gene_id	MSTRG.41820;
AEMK	StringTie	transcrip	933397	962762	.	+	.	transcript_	MSTRG.41903.1	gene_id	MSTRG.41903;
AEMK	StringTie	exon	933397	933419	.	+	.	transcript_	MSTRG.41903.1	gene_id	MSTRG.41903;
AEMK	StringTie	exon	962416	962762	.	+	.	transcript_	MSTRG.41903.1	gene_id	MSTRG.41903;
AEMK	StringTie	transcrip	3361474	3361988	.	+	.	transcript_	MSTRG.41992.1	gene_id	MSTRG.41992;
AEMK	StringTie	exon	3361474	3361570	.	+	.	transcript_	MSTRG.41992.1	gene_id	MSTRG.41992;
AEMK	StringTie	exon	3361728	3361988	.	+	.	transcript_	MSTRG.41992.1	gene_id	MSTRG.41992;
AEMK	StringTie	transcrip	3615213	3676789	.	+	.	transcript_	MSTRG.42045.1	gene_id	MSTRG.42045;
AEMK	StringTie	exon	3615213	3615323	.	+	.	transcript_	MSTRG.42045.1	gene_id	MSTRG.42045;
AEMK	StringTie	exon	3676699	3676789	.	+	.	transcript_	MSTRG.42045.1	gene_id	MSTRG.42045;
AEMK	StringTie	transcrip	3658497	3676803	.	+	.	transcript_	MSTRG.42045.2	gene_id	MSTRG.42045;
AEMK	StringTie	exon	3658497	3658605	.	+	.	transcript_	MSTRG.42045.2	gene_id	MSTRG.42045;
AEMK	StringTie	exon	3676699	3676803	.	+	.	transcript_	MSTRG.42045.2	gene_id	MSTRG.42045;
AEMK	StringTie	transcrip	3634441	3659579	.	+	.	transcript_	MSTRG.42046.1	gene_id	MSTRG.42046;
AEMK	StringTie	exon	3634441	3634546	.	+	.	transcript_	MSTRG.42046.1	gene_id	MSTRG.42046;
AEMK	StringTie	exon	3659472	3659579	.	+	.	transcript_	MSTRG.42046.1	gene_id	MSTRG.42046;
AEMK	StringTie	transcrip	3659354	3699937	.	+	.	transcript_	MSTRG.42047.1	gene_id	MSTRG.42047;
AEMK	StringTie	exon	3659354	3659435	.	+	.	transcript_	MSTRG.42047.1	gene_id	MSTRG.42047;
AEMK	StringTie	exon	3677529	3677564	.	+	.	transcript_	MSTRG.42047.1	gene_id	MSTRG.42047;
AEMK	StringTie	exon	3699829	3699937	.	+	.	transcript_	MSTRG.42047.1	gene_id	MSTRG.42047;

AEMK	StringTie	transcrip	3760564	3768547	.	+	.	transcript_	MSTRG.42049.1	gene_id	MSTRG.42049;
AEMK	StringTie	exon	3760564	3760720	.	+	.	transcript_	MSTRG.42049.1	gene_id	MSTRG.42049;
AEMK	StringTie	exon	3768438	3768547	.	+	.	transcript_	MSTRG.42049.1	gene_id	MSTRG.42049;
AEMK	StringTie	transcrip	775344	782495	.	+	.	transcript_	MSTRG.42120.3	gene_id	MSTRG.42120;
AEMK	StringTie	exon	775344	775681	.	+	.	transcript_	MSTRG.42120.3	gene_id	MSTRG.42120;
AEMK	StringTie	exon	775853	776016	.	+	.	transcript_	MSTRG.42120.3	gene_id	MSTRG.42120;
AEMK	StringTie	exon	777768	777888	.	+	.	transcript_	MSTRG.42120.3	gene_id	MSTRG.42120;
AEMK	StringTie	exon	782391	782495	.	+	.	transcript_	MSTRG.42120.3	gene_id	MSTRG.42120;
AEMK	StringTie	transcrip	775344	782495	.	+	.	transcript_	MSTRG.42120.4	gene_id	MSTRG.42120;
AEMK	StringTie	exon	775344	775626	.	+	.	transcript_	MSTRG.42120.4	gene_id	MSTRG.42120;
AEMK	StringTie	exon	775853	776016	.	+	.	transcript_	MSTRG.42120.4	gene_id	MSTRG.42120;
AEMK	StringTie	exon	777768	777888	.	+	.	transcript_	MSTRG.42120.4	gene_id	MSTRG.42120;
AEMK	StringTie	exon	782391	782495	.	+	.	transcript_	MSTRG.42120.4	gene_id	MSTRG.42120;
AEMK	StringTie	transcrip	775344	782495	.	+	.	transcript_	MSTRG.42120.2	gene_id	MSTRG.42120;
AEMK	StringTie	exon	775344	775681	.	+	.	transcript_	MSTRG.42120.2	gene_id	MSTRG.42120;
AEMK	StringTie	exon	775853	776016	.	+	.	transcript_	MSTRG.42120.2	gene_id	MSTRG.42120;
AEMK	StringTie	exon	777768	777864	.	+	.	transcript_	MSTRG.42120.2	gene_id	MSTRG.42120;
AEMK	StringTie	exon	782391	782495	.	+	.	transcript_	MSTRG.42120.2	gene_id	MSTRG.42120;
AEMK	StringTie	transcrip	775344	782495	.	+	.	transcript_	MSTRG.42120.1	gene_id	MSTRG.42120;
AEMK	StringTie	exon	775344	775683	.	+	.	transcript_	MSTRG.42120.1	gene_id	MSTRG.42120;
AEMK	StringTie	exon	775853	776016	.	+	.	transcript_	MSTRG.42120.1	gene_id	MSTRG.42120;
AEMK	StringTie	exon	777768	777864	.	+	.	transcript_	MSTRG.42120.1	gene_id	MSTRG.42120;
AEMK	StringTie	exon	782391	782495	.	+	.	transcript_	MSTRG.42120.1	gene_id	MSTRG.42120;
AEMK	StringTie	transcrip	775406	782495	.	+	.	transcript_	MSTRG.42120.6	gene_id	MSTRG.42120;
AEMK	StringTie	exon	775406	775705	.	+	.	transcript_	MSTRG.42120.6	gene_id	MSTRG.42120;
AEMK	StringTie	exon	775853	776016	.	+	.	transcript_	MSTRG.42120.6	gene_id	MSTRG.42120;
AEMK	StringTie	exon	777768	777888	.	+	.	transcript_	MSTRG.42120.6	gene_id	MSTRG.42120;
AEMK	StringTie	exon	782391	782495	.	+	.	transcript_	MSTRG.42120.6	gene_id	MSTRG.42120;
AEMK	StringTie	transcrip	775406	782495	.	+	.	transcript_	MSTRG.42120.5	gene_id	MSTRG.42120;
AEMK	StringTie	exon	775406	775683	.	+	.	transcript_	MSTRG.42120.5	gene_id	MSTRG.42120;
AEMK	StringTie	exon	775853	776016	.	+	.	transcript_	MSTRG.42120.5	gene_id	MSTRG.42120;
AEMK	StringTie	exon	777768	777888	.	+	.	transcript_	MSTRG.42120.5	gene_id	MSTRG.42120;
AEMK	StringTie	exon	782391	782495	.	+	.	transcript_	MSTRG.42120.5	gene_id	MSTRG.42120;
AEMK	StringTie	transcrip	775446	782495	.	+	.	transcript_	MSTRG.42120.7	gene_id	MSTRG.42120;
AEMK	StringTie	exon	775446	775705	.	+	.	transcript_	MSTRG.42120.7	gene_id	MSTRG.42120;

AEMK	StringTie	exon	775853	776016	.	+	.	transcript_	MSTRG.42120.7	gene_id	MSTRG.42120;
AEMK	StringTie	exon	777768	777864	.	+	.	transcript_	MSTRG.42120.7	gene_id	MSTRG.42120;
AEMK	StringTie	exon	782391	782495	.	+	.	transcript_	MSTRG.42120.7	gene_id	MSTRG.42120;
AEMK	StringTie	transcrip	775766	784478	.	+	.	transcript_	MSTRG.42120.8	gene_id	MSTRG.42120;
AEMK	StringTie	exon	775766	776016	.	+	.	transcript_	MSTRG.42120.8	gene_id	MSTRG.42120;
AEMK	StringTie	exon	784110	784478	.	+	.	transcript_	MSTRG.42120.8	gene_id	MSTRG.42120;
AEMK	StringTie	transcrip	775806	782495	.	+	.	transcript_	MSTRG.42120.9	gene_id	MSTRG.42120;
AEMK	StringTie	exon	775806	776016	.	+	.	transcript_	MSTRG.42120.9	gene_id	MSTRG.42120;
AEMK	StringTie	exon	782391	782495	.	+	.	transcript_	MSTRG.42120.9	gene_id	MSTRG.42120;
AEMK	StringTie	transcrip	8486	61488	.	-	.	transcript_	MSTRG.42165.1	gene_id	MSTRG.42165;
AEMK	StringTie	exon	8486	9044	.	-	.	transcript_	MSTRG.42165.1	gene_id	MSTRG.42165;
AEMK	StringTie	exon	61183	61488	.	-	.	transcript_	MSTRG.42165.1	gene_id	MSTRG.42165;
AEMK	StringTie	transcrip	61024	117317	.	-	.	transcript_	MSTRG.42167.1	gene_id	MSTRG.42167;
AEMK	StringTie	exon	61024	61182	.	-	.	transcript_	MSTRG.42167.1	gene_id	MSTRG.42167;
AEMK	StringTie	exon	117273	117317	.	-	.	transcript_	MSTRG.42167.1	gene_id	MSTRG.42167;
AEMK	StringTie	transcrip	4306	10737	.	+	.	transcript_	MSTRG.42145.1	gene_id	MSTRG.42145;
AEMK	StringTie	exon	4306	4526	.	+	.	transcript_	MSTRG.42145.1	gene_id	MSTRG.42145;
AEMK	StringTie	exon	10562	10737	.	+	.	transcript_	MSTRG.42145.1	gene_id	MSTRG.42145;
AEMK	StringTie	transcrip	4767	12149	.	+	.	transcript_	MSTRG.42146.1	gene_id	MSTRG.42146;
AEMK	StringTie	exon	4767	5335	.	+	.	transcript_	MSTRG.42146.1	gene_id	MSTRG.42146;
AEMK	StringTie	exon	11371	12149	.	+	.	transcript_	MSTRG.42146.1	gene_id	MSTRG.42146;
AEMK	StringTie	transcrip	16881	23557	.	+	.	transcript_	MSTRG.42147.1	gene_id	MSTRG.42147;
AEMK	StringTie	exon	16881	17405	.	+	.	transcript_	MSTRG.42147.1	gene_id	MSTRG.42147;
AEMK	StringTie	exon	23443	23557	.	+	.	transcript_	MSTRG.42147.1	gene_id	MSTRG.42147;
AEMK	StringTie	transcrip	103826	138067	.	+	.	transcript_	MSTRG.42158.1	gene_id	MSTRG.42158;
AEMK	StringTie	exon	103826	104214	.	+	.	transcript_	MSTRG.42158.1	gene_id	MSTRG.42158;
AEMK	StringTie	exon	133823	134007	.	+	.	transcript_	MSTRG.42158.1	gene_id	MSTRG.42158;
AEMK	StringTie	exon	134593	138067	.	+	.	transcript_	MSTRG.42158.1	gene_id	MSTRG.42158;
AEMK	StringTie	transcrip	125217	138059	.	+	.	transcript_	MSTRG.42158.2	gene_id	MSTRG.42158;
AEMK	StringTie	exon	125217	125242	.	+	.	transcript_	MSTRG.42158.2	gene_id	MSTRG.42158;
AEMK	StringTie	exon	130561	130732	.	+	.	transcript_	MSTRG.42158.2	gene_id	MSTRG.42158;
AEMK	StringTie	exon	133823	134007	.	+	.	transcript_	MSTRG.42158.2	gene_id	MSTRG.42158;
AEMK	StringTie	exon	134593	138059	.	+	.	transcript_	MSTRG.42158.2	gene_id	MSTRG.42158;
AEMK	StringTie	transcrip	88792	93778	.	-	.	transcript_	MSTRG.42193.1	gene_id	MSTRG.42193;
AEMK	StringTie	exon	88792	89826	.	-	.	transcript_	MSTRG.42193.1	gene_id	MSTRG.42193;

AEMK	StringTie	exon	91604	91751	.	-	.	transcript_	MSTRG.42193.1	gene_id	MSTRG.42193;
AEMK	StringTie	exon	92295	92446	.	-	.	transcript_	MSTRG.42193.1	gene_id	MSTRG.42193;
AEMK	StringTie	exon	92895	93778	.	-	.	transcript_	MSTRG.42193.1	gene_id	MSTRG.42193;
AEMK	StringTie	transcrip	24329	24855	.	+	.	transcript_	MSTRG.42180.1	gene_id	MSTRG.42180;
AEMK	StringTie	exon	24329	24706	.	+	.	transcript_	MSTRG.42180.1	gene_id	MSTRG.42180;
AEMK	StringTie	exon	24737	24855	.	+	.	transcript_	MSTRG.42180.1	gene_id	MSTRG.42180;
AEMK	StringTie	transcrip	2133	25073	.	-	.	transcript_	MSTRG.42191.1	gene_id	MSTRG.42191;
AEMK	StringTie	exon	2133	2219	.	-	.	transcript_	MSTRG.42191.1	gene_id	MSTRG.42191;
AEMK	StringTie	exon	3682	3794	.	-	.	transcript_	MSTRG.42191.1	gene_id	MSTRG.42191;
AEMK	StringTie	exon	24862	25073	.	-	.	transcript_	MSTRG.42191.1	gene_id	MSTRG.42191;
AEMK	StringTie	transcrip	1058466	1063776	.	+	.	transcript_	MSTRG.42274.1	gene_id	MSTRG.42274;
AEMK	StringTie	exon	1058466	1058660	.	+	.	transcript_	MSTRG.42274.1	gene_id	MSTRG.42274;
AEMK	StringTie	exon	1059177	1059217	.	+	.	transcript_	MSTRG.42274.1	gene_id	MSTRG.42274;
AEMK	StringTie	exon	1060568	1060633	.	+	.	transcript_	MSTRG.42274.1	gene_id	MSTRG.42274;
AEMK	StringTie	exon	1061420	1061494	.	+	.	transcript_	MSTRG.42274.1	gene_id	MSTRG.42274;
AEMK	StringTie	exon	1063373	1063776	.	+	.	transcript_	MSTRG.42274.1	gene_id	MSTRG.42274;
AEMK	StringTie	transcrip	1058528	1063776	.	+	.	transcript_	MSTRG.42274.2	gene_id	MSTRG.42274;
AEMK	StringTie	exon	1058528	1058709	.	+	.	transcript_	MSTRG.42274.2	gene_id	MSTRG.42274;
AEMK	StringTie	exon	1059177	1059217	.	+	.	transcript_	MSTRG.42274.2	gene_id	MSTRG.42274;
AEMK	StringTie	exon	1060568	1060633	.	+	.	transcript_	MSTRG.42274.2	gene_id	MSTRG.42274;
AEMK	StringTie	exon	1061420	1061494	.	+	.	transcript_	MSTRG.42274.2	gene_id	MSTRG.42274;
AEMK	StringTie	exon	1063373	1063776	.	+	.	transcript_	MSTRG.42274.2	gene_id	MSTRG.42274;
AEMK	StringTie	transcrip	402934	408296	.	-	.	transcript_	MSTRG.42223.1	gene_id	MSTRG.42223;
AEMK	StringTie	exon	402934	402997	.	-	.	transcript_	MSTRG.42223.1	gene_id	MSTRG.42223;
AEMK	StringTie	exon	408094	408296	.	-	.	transcript_	MSTRG.42223.1	gene_id	MSTRG.42223;
AEMK	StringTie	transcrip	839454	840881	.	-	.	transcript_	MSTRG.42256.1	gene_id	MSTRG.42256;
AEMK	StringTie	exon	839454	840683	.	-	.	transcript_	MSTRG.42256.1	gene_id	MSTRG.42256;
AEMK	StringTie	exon	840771	840881	.	-	.	transcript_	MSTRG.42256.1	gene_id	MSTRG.42256;
AEMK	StringTie	transcrip	1556164	1556993	.	-	.	transcript_	MSTRG.42294.1	gene_id	MSTRG.42294;
AEMK	StringTie	exon	1556164	1556230	.	-	.	transcript_	MSTRG.42294.1	gene_id	MSTRG.42294;
AEMK	StringTie	exon	1556359	1556993	.	-	.	transcript_	MSTRG.42294.1	gene_id	MSTRG.42294;
AEMK	StringTie	transcrip	10139	45216	.	-	.	transcript_	MSTRG.42362.1	gene_id	MSTRG.42362;
AEMK	StringTie	exon	10139	13625	.	-	.	transcript_	MSTRG.42362.1	gene_id	MSTRG.42362;
AEMK	StringTie	exon	14212	14396	.	-	.	transcript_	MSTRG.42362.1	gene_id	MSTRG.42362;
AEMK	StringTie	exon	45094	45216	.	-	.	transcript_	MSTRG.42362.1	gene_id	MSTRG.42362;

AEMK	StringTie	transcrip	10144	45210	.	-	.	transcript_	MSTRG.42362.2	gene_id	MSTRG.42362;
AEMK	StringTie	exon	10144	13625	.	-	.	transcript_	MSTRG.42362.2	gene_id	MSTRG.42362;
AEMK	StringTie	exon	14212	14396	.	-	.	transcript_	MSTRG.42362.2	gene_id	MSTRG.42362;
AEMK	StringTie	exon	45154	45210	.	-	.	transcript_	MSTRG.42362.2	gene_id	MSTRG.42362;
MT	StringTie	transcrip	1	9630	.	-	.	transcript_	MSTRG.42387.1	gene_id	MSTRG.42387;
MT	StringTie	exon	1	144	.	-	.	transcript_	MSTRG.42387.1	gene_id	MSTRG.42387;
MT	StringTie	exon	2994	3149	.	-	.	transcript_	MSTRG.42387.1	gene_id	MSTRG.42387;
MT	StringTie	exon	9520	9630	.	-	.	transcript_	MSTRG.42387.1	gene_id	MSTRG.42387;
MT	StringTie	transcrip	2567	9630	.	-	.	transcript_	MSTRG.42387.2	gene_id	MSTRG.42387;
MT	StringTie	exon	2567	2677	.	-	.	transcript_	MSTRG.42387.2	gene_id	MSTRG.42387;
MT	StringTie	exon	4413	4518	.	-	.	transcript_	MSTRG.42387.2	gene_id	MSTRG.42387;
MT	StringTie	exon	4577	4731	.	-	.	transcript_	MSTRG.42387.2	gene_id	MSTRG.42387;
MT	StringTie	exon	9172	9319	.	-	.	transcript_	MSTRG.42387.2	gene_id	MSTRG.42387;
MT	StringTie	exon	9423	9630	.	-	.	transcript_	MSTRG.42387.2	gene_id	MSTRG.42387;
MT	StringTie	transcrip	2567	9639	.	-	.	transcript_	MSTRG.42387.3	gene_id	MSTRG.42387;
MT	StringTie	exon	2567	2677	.	-	.	transcript_	MSTRG.42387.3	gene_id	MSTRG.42387;
MT	StringTie	exon	9098	9319	.	-	.	transcript_	MSTRG.42387.3	gene_id	MSTRG.42387;
MT	StringTie	exon	9423	9639	.	-	.	transcript_	MSTRG.42387.3	gene_id	MSTRG.42387;
MT	StringTie	transcrip	2738	12567	.	-	.	transcript_	MSTRG.42387.5	gene_id	MSTRG.42387;
MT	StringTie	exon	2738	2862	.	-	.	transcript_	MSTRG.42387.5	gene_id	MSTRG.42387;
MT	StringTie	exon	12457	12567	.	-	.	transcript_	MSTRG.42387.5	gene_id	MSTRG.42387;
MT	StringTie	transcrip	2738	12726	.	-	.	transcript_	MSTRG.42387.4	gene_id	MSTRG.42387;
MT	StringTie	exon	2738	2848	.	-	.	transcript_	MSTRG.42387.4	gene_id	MSTRG.42387;
MT	StringTie	exon	12112	12387	.	-	.	transcript_	MSTRG.42387.4	gene_id	MSTRG.42387;
MT	StringTie	exon	12457	12726	.	-	.	transcript_	MSTRG.42387.4	gene_id	MSTRG.42387;
MT	StringTie	transcrip	3024	9603	.	-	.	transcript_	MSTRG.42387.6	gene_id	MSTRG.42387;
MT	StringTie	exon	3024	3119	.	-	.	transcript_	MSTRG.42387.6	gene_id	MSTRG.42387;
MT	StringTie	exon	9493	9603	.	-	.	transcript_	MSTRG.42387.6	gene_id	MSTRG.42387;
MT	StringTie	transcrip	5432	5809	.	-	.	transcript_	MSTRG.42387.1	gene_id	MSTRG.42387;
MT	StringTie	exon	5432	5542	.	-	.	transcript_	MSTRG.42387.1	gene_id	MSTRG.42387;
MT	StringTie	exon	5699	5809	.	-	.	transcript_	MSTRG.42387.1	gene_id	MSTRG.42387;
MT	StringTie	transcrip	5432	16248	.	-	.	transcript_	MSTRG.42387.1	gene_id	MSTRG.42387;
MT	StringTie	exon	5432	5542	.	-	.	transcript_	MSTRG.42387.1	gene_id	MSTRG.42387;
MT	StringTie	exon	5605	5751	.	-	.	transcript_	MSTRG.42387.1	gene_id	MSTRG.42387;
MT	StringTie	exon	16112	16248	.	-	.	transcript_	MSTRG.42387.1	gene_id	MSTRG.42387;

MT	StringTie	transcrip	7093	9603	.	-	.	transcript_	MSTRG.42387.1	gene_id	MSTRG.42387;
MT	StringTie	exon	7093	7163	.	-	.	transcript_	MSTRG.42387.1	gene_id	MSTRG.42387;
MT	StringTie	exon	9412	9603	.	-	.	transcript_	MSTRG.42387.1	gene_id	MSTRG.42387;
MT	StringTie	transcrip	7418	12016	.	-	.	transcript_	MSTRG.42387.1	gene_id	MSTRG.42387;
MT	StringTie	exon	7418	7528	.	-	.	transcript_	MSTRG.42387.1	gene_id	MSTRG.42387;
MT	StringTie	exon	7869	7983	.	-	.	transcript_	MSTRG.42387.1	gene_id	MSTRG.42387;
MT	StringTie	exon	9320	9563	.	-	.	transcript_	MSTRG.42387.1	gene_id	MSTRG.42387;
MT	StringTie	exon	9624	9750	.	-	.	transcript_	MSTRG.42387.1	gene_id	MSTRG.42387;
MT	StringTie	exon	11911	12016	.	-	.	transcript_	MSTRG.42387.1	gene_id	MSTRG.42387;
MT	StringTie	transcrip	7730	10384	.	-	.	transcript_	MSTRG.42387.1	gene_id	MSTRG.42387;
MT	StringTie	exon	7730	7840	.	-	.	transcript_	MSTRG.42387.1	gene_id	MSTRG.42387;
MT	StringTie	exon	10234	10384	.	-	.	transcript_	MSTRG.42387.1	gene_id	MSTRG.42387;
MT	StringTie	transcrip	8479	12568	.	-	.	transcript_	MSTRG.42387.1	gene_id	MSTRG.42387;
MT	StringTie	exon	8479	8590	.	-	.	transcript_	MSTRG.42387.1	gene_id	MSTRG.42387;
MT	StringTie	exon	12457	12568	.	-	.	transcript_	MSTRG.42387.1	gene_id	MSTRG.42387;
MT	StringTie	transcrip	8984	12567	.	-	.	transcript_	MSTRG.42387.1	gene_id	MSTRG.42387;
MT	StringTie	exon	8984	9206	.	-	.	transcript_	MSTRG.42387.1	gene_id	MSTRG.42387;
MT	StringTie	exon	12457	12567	.	-	.	transcript_	MSTRG.42387.1	gene_id	MSTRG.42387;
MT	StringTie	transcrip	10004	13143	.	-	.	transcript_	MSTRG.42387.2	gene_id	MSTRG.42387;
MT	StringTie	exon	10004	10114	.	-	.	transcript_	MSTRG.42387.2	gene_id	MSTRG.42387;
MT	StringTie	exon	12457	12701	.	-	.	transcript_	MSTRG.42387.2	gene_id	MSTRG.42387;
MT	StringTie	exon	13025	13143	.	-	.	transcript_	MSTRG.42387.2	gene_id	MSTRG.42387;
MT	StringTie	transcrip	10064	10456	.	-	.	transcript_	MSTRG.42387.2	gene_id	MSTRG.42387;
MT	StringTie	exon	10064	10200	.	-	.	transcript_	MSTRG.42387.2	gene_id	MSTRG.42387;
MT	StringTie	exon	10274	10456	.	-	.	transcript_	MSTRG.42387.2	gene_id	MSTRG.42387;
MT	StringTie	transcrip	10079	10427	.	-	.	transcript_	MSTRG.42387.2	gene_id	MSTRG.42387;
MT	StringTie	exon	10079	10233	.	-	.	transcript_	MSTRG.42387.2	gene_id	MSTRG.42387;
MT	StringTie	exon	10297	10427	.	-	.	transcript_	MSTRG.42387.2	gene_id	MSTRG.42387;
MT	StringTie	transcrip	15708	16250	.	-	.	transcript_	MSTRG.42387.2	gene_id	MSTRG.42387;
MT	StringTie	exon	15708	15880	.	-	.	transcript_	MSTRG.42387.2	gene_id	MSTRG.42387;
MT	StringTie	exon	16037	16085	.	-	.	transcript_	MSTRG.42387.2	gene_id	MSTRG.42387;
MT	StringTie	exon	16122	16250	.	-	.	transcript_	MSTRG.42387.2	gene_id	MSTRG.42387;
MT	StringTie	transcrip	15834	16232	.	-	.	transcript_	MSTRG.42387.3	gene_id	MSTRG.42387;
MT	StringTie	exon	15834	15963	.	-	.	transcript_	MSTRG.42387.3	gene_id	MSTRG.42387;
MT	StringTie	exon	16122	16232	.	-	.	transcript_	MSTRG.42387.3	gene_id	MSTRG.42387;

MT	StringTie	transcrip	15945	16250	.	-	.	transcript_	MSTRG.42387.3	gene_id	MSTRG.42387;
MT	StringTie	exon	15945	16085	.	-	.	transcript_	MSTRG.42387.3	gene_id	MSTRG.42387;
MT	StringTie	exon	16138	16250	.	-	.	transcript_	MSTRG.42387.3	gene_id	MSTRG.42387;
MT	StringTie	transcrip	11274	11614	.	-	.	transcript_	MSTRG.42387.2	gene_id	MSTRG.42387;
MT	StringTie	exon	11274	11350	.	-	.	transcript_	MSTRG.42387.2	gene_id	MSTRG.42387;
MT	StringTie	exon	11417	11614	.	-	.	transcript_	MSTRG.42387.2	gene_id	MSTRG.42387;
X	StringTie	transcrip	123389	124088	.	+	.	transcript_	MSTRG.42379.1	gene_id	MSTRG.42379;
X	StringTie	exon	123389	123599	.	+	.	transcript_	MSTRG.42379.1	gene_id	MSTRG.42379;
X	StringTie	exon	124038	124088	.	+	.	transcript_	MSTRG.42379.1	gene_id	MSTRG.42379;
X	StringTie	transcrip	17954049	18000839	.	+	.	transcript_	MSTRG.42563.1	gene_id	MSTRG.42563;
X	StringTie	exon	17954049	17954090	.	+	.	transcript_	MSTRG.42563.1	gene_id	MSTRG.42563;
X	StringTie	exon	17997505	17997575	.	+	.	transcript_	MSTRG.42563.1	gene_id	MSTRG.42563;
X	StringTie	exon	18000686	18000839	.	+	.	transcript_	MSTRG.42563.1	gene_id	MSTRG.42563;
X	StringTie	transcrip	38902375	38908896	.	+	.	transcript_	MSTRG.42745.1	gene_id	MSTRG.42745;
X	StringTie	exon	38902375	38902507	.	+	.	transcript_	MSTRG.42745.1	gene_id	MSTRG.42745;
X	StringTie	exon	38903448	38903844	.	+	.	transcript_	MSTRG.42745.1	gene_id	MSTRG.42745;
X	StringTie	exon	38908790	38908896	.	+	.	transcript_	MSTRG.42745.1	gene_id	MSTRG.42745;
X	StringTie	transcrip	41916338	41933353	.	+	.	transcript_	MSTRG.42790.1	gene_id	MSTRG.42790;
X	StringTie	exon	41916338	41916354	.	+	.	transcript_	MSTRG.42790.1	gene_id	MSTRG.42790;
X	StringTie	exon	41932961	41933353	.	+	.	transcript_	MSTRG.42790.1	gene_id	MSTRG.42790;
X	StringTie	transcrip	43193282	43201542	.	+	.	transcript_	MSTRG.42838.1	gene_id	MSTRG.42838;
X	StringTie	exon	43193282	43193352	.	+	.	transcript_	MSTRG.42838.1	gene_id	MSTRG.42838;
X	StringTie	exon	43200984	43201542	.	+	.	transcript_	MSTRG.42838.1	gene_id	MSTRG.42838;
X	StringTie	transcrip	45856403	45858832	.	+	.	transcript_	MSTRG.42891.1	gene_id	MSTRG.42891;
X	StringTie	exon	45856403	45856660	.	+	.	transcript_	MSTRG.42891.1	gene_id	MSTRG.42891;
X	StringTie	exon	45858784	45858832	.	+	.	transcript_	MSTRG.42891.1	gene_id	MSTRG.42891;
X	StringTie	transcrip	47703572	47780522	.	+	.	transcript_	MSTRG.42949.2	gene_id	MSTRG.42949;
X	StringTie	exon	47703572	47703873	.	+	.	transcript_	MSTRG.42949.2	gene_id	MSTRG.42949;
X	StringTie	exon	47780418	47780522	.	+	.	transcript_	MSTRG.42949.2	gene_id	MSTRG.42949;
X	StringTie	transcrip	47704400	47781299	.	+	.	transcript_	MSTRG.42951.1	gene_id	MSTRG.42951;
X	StringTie	exon	47704400	47704658	.	+	.	transcript_	MSTRG.42951.1	gene_id	MSTRG.42951;
X	StringTie	exon	47781201	47781299	.	+	.	transcript_	MSTRG.42951.1	gene_id	MSTRG.42951;
X	StringTie	transcrip	48953580	48986602	.	+	.	transcript_	MSTRG.42980.1	gene_id	MSTRG.42980;
X	StringTie	exon	48953580	48953648	.	+	.	transcript_	MSTRG.42980.1	gene_id	MSTRG.42980;
X	StringTie	exon	48986400	48986602	.	+	.	transcript_	MSTRG.42980.1	gene_id	MSTRG.42980;

X	StringTie	transcrip	56463836	56465669	.	+	.	transcript_	MSTRG.43156.[illegible]	gene_id	MSTRG.43156;
X	StringTie	exon	56463836	56464252	.	+	.	transcript_	MSTRG.43156.[illegible]	gene_id	MSTRG.43156;
X	StringTie	exon	56464792	56465669	.	+	.	transcript_	MSTRG.43156.[illegible]	gene_id	MSTRG.43156;
X	StringTie	transcrip	57483652	57488565	.	+	.	transcript_	MSTRG.43179.[illegible]	gene_id	MSTRG.43179;
X	StringTie	exon	57483652	57483762	.	+	.	transcript_	MSTRG.43179.[illegible]	gene_id	MSTRG.43179;
X	StringTie	exon	57488423	57488565	.	+	.	transcript_	MSTRG.43179.[illegible]	gene_id	MSTRG.43179;
X	StringTie	transcrip	59490596	59557323	.	+	.	transcript_	MSTRG.43296.[illegible]	gene_id	MSTRG.43296;
X	StringTie	exon	59490596	59490909	.	+	.	transcript_	MSTRG.43296.[illegible]	gene_id	MSTRG.43296;
X	StringTie	exon	59555996	59557323	.	+	.	transcript_	MSTRG.43296.[illegible]	gene_id	MSTRG.43296;
X	StringTie	transcrip	59511286	59557197	.	+	.	transcript_	MSTRG.43296.[illegible]	gene_id	MSTRG.43296;
X	StringTie	exon	59511286	59511652	.	+	.	transcript_	MSTRG.43296.[illegible]	gene_id	MSTRG.43296;
X	StringTie	exon	59513367	59513455	.	+	.	transcript_	MSTRG.43296.[illegible]	gene_id	MSTRG.43296;
X	StringTie	exon	59555996	59557197	.	+	.	transcript_	MSTRG.43296.[illegible]	gene_id	MSTRG.43296;
X	StringTie	transcrip	60626650	60627001	.	+	.	transcript_	MSTRG.43340.[illegible]	gene_id	MSTRG.43340;
X	StringTie	exon	60626650	60626758	.	+	.	transcript_	MSTRG.43340.[illegible]	gene_id	MSTRG.43340;
X	StringTie	exon	60626892	60627001	.	+	.	transcript_	MSTRG.43340.[illegible]	gene_id	MSTRG.43340;
X	StringTie	transcrip	83223821	83224690	.	+	.	transcript_	MSTRG.43611.[illegible]	gene_id	MSTRG.43611;
X	StringTie	exon	83223821	83223920	.	+	.	transcript_	MSTRG.43611.[illegible]	gene_id	MSTRG.43611;
X	StringTie	exon	83224417	83224690	.	+	.	transcript_	MSTRG.43611.[illegible]	gene_id	MSTRG.43611;
X	StringTie	transcrip	84922666	84924863	.	+	.	transcript_	MSTRG.43678.[illegible]	gene_id	MSTRG.43678;
X	StringTie	exon	84922666	84922705	.	+	.	transcript_	MSTRG.43678.[illegible]	gene_id	MSTRG.43678;
X	StringTie	exon	84924656	84924863	.	+	.	transcript_	MSTRG.43678.[illegible]	gene_id	MSTRG.43678;
X	StringTie	transcrip	94983320	94986601	.	+	.	transcript_	MSTRG.43766.[illegible]	gene_id	MSTRG.43766;
X	StringTie	exon	94983320	94983449	.	+	.	transcript_	MSTRG.43766.[illegible]	gene_id	MSTRG.43766;
X	StringTie	exon	94986508	94986601	.	+	.	transcript_	MSTRG.43766.[illegible]	gene_id	MSTRG.43766;
X	StringTie	transcrip	98758640	98821328	.	+	.	transcript_	MSTRG.43846.[illegible]	gene_id	MSTRG.43846;
X	StringTie	exon	98758640	98758879	.	+	.	transcript_	MSTRG.43846.[illegible]	gene_id	MSTRG.43846;
X	StringTie	exon	98771798	98771922	.	+	.	transcript_	MSTRG.43846.[illegible]	gene_id	MSTRG.43846;
X	StringTie	exon	98821106	98821328	.	+	.	transcript_	MSTRG.43846.[illegible]	gene_id	MSTRG.43846;
X	StringTie	transcrip	98812630	98821328	.	+	.	transcript_	MSTRG.43846.[illegible]	gene_id	MSTRG.43846;
X	StringTie	exon	98812630	98812697	.	+	.	transcript_	MSTRG.43846.[illegible]	gene_id	MSTRG.43846;
X	StringTie	exon	98821106	98821328	.	+	.	transcript_	MSTRG.43846.[illegible]	gene_id	MSTRG.43846;
X	StringTie	transcrip	109538090	109541377	.	+	.	transcript_	MSTRG.43921.[illegible]	gene_id	MSTRG.43921;
X	StringTie	exon	109538090	109538220	.	+	.	transcript_	MSTRG.43921.[illegible]	gene_id	MSTRG.43921;
X	StringTie	exon	109541255	109541377	.	+	.	transcript_	MSTRG.43921.[illegible]	gene_id	MSTRG.43921;

X	StringTie	transcrip	110810159	110810779	.	+	.	transcript_	MSTRG.43950.1	gene_id	MSTRG.43950;
X	StringTie	exon	110810159	110810779	.	+	.	transcript_	MSTRG.43950.1	gene_id	MSTRG.43950;
X	StringTie	transcrip	110810174	110834078	.	+	.	transcript_	MSTRG.43950.2	gene_id	MSTRG.43950;
X	StringTie	exon	110810174	110810354	.	+	.	transcript_	MSTRG.43950.2	gene_id	MSTRG.43950;
X	StringTie	exon	110810392	110810447	.	+	.	transcript_	MSTRG.43950.2	gene_id	MSTRG.43950;
X	StringTie	exon	110833318	110834078	.	+	.	transcript_	MSTRG.43950.2	gene_id	MSTRG.43950;
X	StringTie	transcrip	110960325	110962267	.	+	.	transcript_	MSTRG.43965.1	gene_id	MSTRG.43965;
X	StringTie	exon	110960325	110960421	.	+	.	transcript_	MSTRG.43965.1	gene_id	MSTRG.43965;
X	StringTie	exon	110961195	110961322	.	+	.	transcript_	MSTRG.43965.1	gene_id	MSTRG.43965;
X	StringTie	exon	110961935	110962267	.	+	.	transcript_	MSTRG.43965.1	gene_id	MSTRG.43965;
X	StringTie	transcrip	114575759	115016866	.	+	.	transcript_	MSTRG.43999.1	gene_id	MSTRG.43999;
X	StringTie	exon	114575759	114576101	.	+	.	transcript_	MSTRG.43999.1	gene_id	MSTRG.43999;
X	StringTie	exon	114868858	114868937	.	+	.	transcript_	MSTRG.43999.1	gene_id	MSTRG.43999;
X	StringTie	exon	115016586	115016866	.	+	.	transcript_	MSTRG.43999.1	gene_id	MSTRG.43999;
X	StringTie	transcrip	122517978	122521952	.	+	.	transcript_	MSTRG.44070.1	gene_id	MSTRG.44070;
X	StringTie	exon	122517978	122518220	.	+	.	transcript_	MSTRG.44070.1	gene_id	MSTRG.44070;
X	StringTie	exon	122521848	122521952	.	+	.	transcript_	MSTRG.44070.1	gene_id	MSTRG.44070;
X	StringTie	transcrip	106463	107581	.	-	.	transcript_	MSTRG.42372.1	gene_id	MSTRG.42372;
X	StringTie	exon	106463	107312	.	-	.	transcript_	MSTRG.42372.1	gene_id	MSTRG.42372;
X	StringTie	exon	107359	107581	.	-	.	transcript_	MSTRG.42372.1	gene_id	MSTRG.42372;
X	StringTie	transcrip	113393	115663	.	-	.	transcript_	MSTRG.42378.1	gene_id	MSTRG.42378;
X	StringTie	exon	113393	113467	.	-	.	transcript_	MSTRG.42378.1	gene_id	MSTRG.42378;
X	StringTie	exon	113618	115663	.	-	.	transcript_	MSTRG.42378.1	gene_id	MSTRG.42378;
X	StringTie	transcrip	6929828	6930070	.	-	.	transcript_	MSTRG.42424.1	gene_id	MSTRG.42424;
X	StringTie	exon	6929828	6929925	.	-	.	transcript_	MSTRG.42424.1	gene_id	MSTRG.42424;
X	StringTie	exon	6929963	6930070	.	-	.	transcript_	MSTRG.42424.1	gene_id	MSTRG.42424;
X	StringTie	transcrip	17793666	17829024	.	-	.	transcript_	MSTRG.42569.1	gene_id	MSTRG.42569;
X	StringTie	exon	17793666	17793693	.	-	.	transcript_	MSTRG.42569.1	gene_id	MSTRG.42569;
X	StringTie	exon	17794077	17794472	.	-	.	transcript_	MSTRG.42569.1	gene_id	MSTRG.42569;
X	StringTie	exon	17828753	17828888	.	-	.	transcript_	MSTRG.42569.1	gene_id	MSTRG.42569;
X	StringTie	exon	17828998	17829024	.	-	.	transcript_	MSTRG.42569.1	gene_id	MSTRG.42569;
X	StringTie	transcrip	17845536	17862340	.	-	.	transcript_	MSTRG.42571.1	gene_id	MSTRG.42571;
X	StringTie	exon	17845536	17846263	.	-	.	transcript_	MSTRG.42571.1	gene_id	MSTRG.42571;
X	StringTie	exon	17862290	17862340	.	-	.	transcript_	MSTRG.42571.1	gene_id	MSTRG.42571;
X	StringTie	transcrip	22068981	22069290	.	-	.	transcript_	MSTRG.42621.1	gene_id	MSTRG.42621;

X	StringTie	exon	22068981	22069092	.	-	.	transcript_	MSTRG.42621.1	gene_id	MSTRG.42621;
X	StringTie	exon	22069177	22069290	.	-	.	transcript_	MSTRG.42621.1	gene_id	MSTRG.42621;
X	StringTie	transcrip	33051723	33058725	.	-	.	transcript_	MSTRG.42706.1	gene_id	MSTRG.42706;
X	StringTie	exon	33051723	33052505	.	-	.	transcript_	MSTRG.42706.1	gene_id	MSTRG.42706;
X	StringTie	exon	33058611	33058725	.	-	.	transcript_	MSTRG.42706.1	gene_id	MSTRG.42706;
X	StringTie	transcrip	33052193	33058725	.	-	.	transcript_	MSTRG.42706.2	gene_id	MSTRG.42706;
X	StringTie	exon	33052193	33052505	.	-	.	transcript_	MSTRG.42706.2	gene_id	MSTRG.42706;
X	StringTie	exon	33055312	33055460	.	-	.	transcript_	MSTRG.42706.2	gene_id	MSTRG.42706;
X	StringTie	exon	33058611	33058725	.	-	.	transcript_	MSTRG.42706.2	gene_id	MSTRG.42706;
X	StringTie	transcrip	34317938	34516499	.	-	.	transcript_	MSTRG.42728.1	gene_id	MSTRG.42728;
X	StringTie	exon	34317938	34318077	.	-	.	transcript_	MSTRG.42728.1	gene_id	MSTRG.42728;
X	StringTie	exon	34516411	34516499	.	-	.	transcript_	MSTRG.42728.1	gene_id	MSTRG.42728;
X	StringTie	transcrip	35099720	35108595	.	-	.	transcript_	MSTRG.42726.1	gene_id	MSTRG.42726;
X	StringTie	exon	35099720	35099903	.	-	.	transcript_	MSTRG.42726.1	gene_id	MSTRG.42726;
X	StringTie	exon	35108572	35108595	.	-	.	transcript_	MSTRG.42726.1	gene_id	MSTRG.42726;
X	StringTie	transcrip	36919670	36929089	.	-	.	transcript_	MSTRG.42739.1	gene_id	MSTRG.42739;
X	StringTie	exon	36919670	36919826	.	-	.	transcript_	MSTRG.42739.1	gene_id	MSTRG.42739;
X	StringTie	exon	36926805	36926886	.	-	.	transcript_	MSTRG.42739.1	gene_id	MSTRG.42739;
X	StringTie	exon	36928945	36929089	.	-	.	transcript_	MSTRG.42739.1	gene_id	MSTRG.42739;
X	StringTie	transcrip	38253314	38273269	.	-	.	transcript_	MSTRG.42746.1	gene_id	MSTRG.42746;
X	StringTie	exon	38253314	38260108	.	-	.	transcript_	MSTRG.42746.1	gene_id	MSTRG.42746;
X	StringTie	exon	38273038	38273269	.	-	.	transcript_	MSTRG.42746.1	gene_id	MSTRG.42746;
X	StringTie	transcrip	38255989	38273249	.	-	.	transcript_	MSTRG.42746.2	gene_id	MSTRG.42746;
X	StringTie	exon	38255989	38260108	.	-	.	transcript_	MSTRG.42746.2	gene_id	MSTRG.42746;
X	StringTie	exon	38262964	38263046	.	-	.	transcript_	MSTRG.42746.2	gene_id	MSTRG.42746;
X	StringTie	exon	38273038	38273249	.	-	.	transcript_	MSTRG.42746.2	gene_id	MSTRG.42746;
X	StringTie	transcrip	38256951	38266306	.	-	.	transcript_	MSTRG.42746.3	gene_id	MSTRG.42746;
X	StringTie	exon	38256951	38260108	.	-	.	transcript_	MSTRG.42746.3	gene_id	MSTRG.42746;
X	StringTie	exon	38262964	38263046	.	-	.	transcript_	MSTRG.42746.3	gene_id	MSTRG.42746;
X	StringTie	exon	38265995	38266306	.	-	.	transcript_	MSTRG.42746.3	gene_id	MSTRG.42746;
X	StringTie	transcrip	45721669	45757912	.	-	.	transcript_	MSTRG.42888.1	gene_id	MSTRG.42888;
X	StringTie	exon	45721669	45721695	.	-	.	transcript_	MSTRG.42888.1	gene_id	MSTRG.42888;
X	StringTie	exon	45755450	45755527	.	-	.	transcript_	MSTRG.42888.1	gene_id	MSTRG.42888;
X	StringTie	exon	45757655	45757912	.	-	.	transcript_	MSTRG.42888.1	gene_id	MSTRG.42888;
X	StringTie	transcrip	59326048	59368990	.	-	.	transcript_	MSTRG.43295.1	gene_id	MSTRG.43295;

X	StringTie	exon	59326048	59326066	.	-	.	transcript_	MSTRG.43295.1	gene_id	MSTRG.43295;
X	StringTie	exon	59368329	59368990	.	-	.	transcript_	MSTRG.43295.1	gene_id	MSTRG.43295;
X	StringTie	transcrip	59475708	59484479	.	-	.	transcript_	MSTRG.43297.1	gene_id	MSTRG.43297;
X	StringTie	exon	59475708	59475782	.	-	.	transcript_	MSTRG.43297.1	gene_id	MSTRG.43297;
X	StringTie	exon	59476476	59476545	.	-	.	transcript_	MSTRG.43297.1	gene_id	MSTRG.43297;
X	StringTie	exon	59484252	59484479	.	-	.	transcript_	MSTRG.43297.1	gene_id	MSTRG.43297;
X	StringTie	transcrip	64700247	64701792	.	-	.	transcript_	MSTRG.43413.1	gene_id	MSTRG.43413;
X	StringTie	exon	64700247	64700329	.	-	.	transcript_	MSTRG.43413.1	gene_id	MSTRG.43413;
X	StringTie	exon	64700892	64701792	.	-	.	transcript_	MSTRG.43413.1	gene_id	MSTRG.43413;
X	StringTie	transcrip	78360516	78539976	.	-	.	transcript_	MSTRG.43545.1	gene_id	MSTRG.43545;
X	StringTie	exon	78360516	78360916	.	-	.	transcript_	MSTRG.43545.1	gene_id	MSTRG.43545;
X	StringTie	exon	78539834	78539976	.	-	.	transcript_	MSTRG.43545.1	gene_id	MSTRG.43545;
X	StringTie	transcrip	84903304	84922540	.	-	.	transcript_	MSTRG.43677.4	gene_id	MSTRG.43677;
X	StringTie	exon	84903304	84903590	.	-	.	transcript_	MSTRG.43677.4	gene_id	MSTRG.43677;
X	StringTie	exon	84909691	84909912	.	-	.	transcript_	MSTRG.43677.4	gene_id	MSTRG.43677;
X	StringTie	exon	84922436	84922540	.	-	.	transcript_	MSTRG.43677.4	gene_id	MSTRG.43677;
X	StringTie	transcrip	84903304	84922540	.	-	.	transcript_	MSTRG.43677.2	gene_id	MSTRG.43677;
X	StringTie	exon	84903304	84903590	.	-	.	transcript_	MSTRG.43677.2	gene_id	MSTRG.43677;
X	StringTie	exon	84918739	84918910	.	-	.	transcript_	MSTRG.43677.2	gene_id	MSTRG.43677;
X	StringTie	exon	84922390	84922540	.	-	.	transcript_	MSTRG.43677.2	gene_id	MSTRG.43677;
X	StringTie	transcrip	84903304	84922586	.	-	.	transcript_	MSTRG.43677.3	gene_id	MSTRG.43677;
X	StringTie	exon	84903304	84903590	.	-	.	transcript_	MSTRG.43677.3	gene_id	MSTRG.43677;
X	StringTie	exon	84922436	84922586	.	-	.	transcript_	MSTRG.43677.3	gene_id	MSTRG.43677;
X	StringTie	transcrip	84903304	84922586	.	-	.	transcript_	MSTRG.43677.1	gene_id	MSTRG.43677;
X	StringTie	exon	84903304	84903590	.	-	.	transcript_	MSTRG.43677.1	gene_id	MSTRG.43677;
X	StringTie	exon	84922390	84922586	.	-	.	transcript_	MSTRG.43677.1	gene_id	MSTRG.43677;
X	StringTie	transcrip	84903305	84922540	.	-	.	transcript_	MSTRG.43677.6	gene_id	MSTRG.43677;
X	StringTie	exon	84903305	84903590	.	-	.	transcript_	MSTRG.43677.6	gene_id	MSTRG.43677;
X	StringTie	exon	84911734	84911797	.	-	.	transcript_	MSTRG.43677.6	gene_id	MSTRG.43677;
X	StringTie	exon	84922390	84922540	.	-	.	transcript_	MSTRG.43677.6	gene_id	MSTRG.43677;
X	StringTie	transcrip	84903305	84922694	.	-	.	transcript_	MSTRG.43677.5	gene_id	MSTRG.43677;
X	StringTie	exon	84903305	84903590	.	-	.	transcript_	MSTRG.43677.5	gene_id	MSTRG.43677;
X	StringTie	exon	84909691	84909912	.	-	.	transcript_	MSTRG.43677.5	gene_id	MSTRG.43677;
X	StringTie	exon	84922390	84922694	.	-	.	transcript_	MSTRG.43677.5	gene_id	MSTRG.43677;
X	StringTie	transcrip	84903306	84922538	.	-	.	transcript_	MSTRG.43677.7	gene_id	MSTRG.43677;

X	StringTie	exon	84903306	84903590	.	-	.	transcript_	MSTRG.43677.7	gene_id	MSTRG.43677;
X	StringTie	exon	84909691	84909912	.	-	.	transcript_	MSTRG.43677.7	gene_id	MSTRG.43677;
X	StringTie	exon	84911734	84911797	.	-	.	transcript_	MSTRG.43677.7	gene_id	MSTRG.43677;
X	StringTie	exon	84918739	84918910	.	-	.	transcript_	MSTRG.43677.7	gene_id	MSTRG.43677;
X	StringTie	exon	84922390	84922538	.	-	.	transcript_	MSTRG.43677.7	gene_id	MSTRG.43677;
X	StringTie	transcrip	84903306	84922544	.	-	.	transcript_	MSTRG.43677.8	gene_id	MSTRG.43677;
X	StringTie	exon	84903306	84903590	.	-	.	transcript_	MSTRG.43677.8	gene_id	MSTRG.43677;
X	StringTie	exon	84918739	84918910	.	-	.	transcript_	MSTRG.43677.8	gene_id	MSTRG.43677;
X	StringTie	exon	84922436	84922544	.	-	.	transcript_	MSTRG.43677.8	gene_id	MSTRG.43677;
X	StringTie	transcrip	84903316	84922536	.	-	.	transcript_	MSTRG.43677.9	gene_id	MSTRG.43677;
X	StringTie	exon	84903316	84903590	.	-	.	transcript_	MSTRG.43677.9	gene_id	MSTRG.43677;
X	StringTie	exon	84911734	84911797	.	-	.	transcript_	MSTRG.43677.9	gene_id	MSTRG.43677;
X	StringTie	exon	84918739	84918910	.	-	.	transcript_	MSTRG.43677.9	gene_id	MSTRG.43677;
X	StringTie	exon	84922390	84922536	.	-	.	transcript_	MSTRG.43677.9	gene_id	MSTRG.43677;
X	StringTie	transcrip	87401251	87401779	.	-	.	transcript_	MSTRG.43699.1	gene_id	MSTRG.43699;
X	StringTie	exon	87401251	87401646	.	-	.	transcript_	MSTRG.43699.1	gene_id	MSTRG.43699;
X	StringTie	exon	87401756	87401779	.	-	.	transcript_	MSTRG.43699.1	gene_id	MSTRG.43699;
X	StringTie	transcrip	94864973	94947055	.	-	.	transcript_	MSTRG.43769.1	gene_id	MSTRG.43769;
X	StringTie	exon	94864973	94864999	.	-	.	transcript_	MSTRG.43769.1	gene_id	MSTRG.43769;
X	StringTie	exon	94946663	94947055	.	-	.	transcript_	MSTRG.43769.1	gene_id	MSTRG.43769;
X	StringTie	transcrip	98390553	98397208	.	-	.	transcript_	MSTRG.43819.1	gene_id	MSTRG.43819;
X	StringTie	exon	98390553	98390784	.	-	.	transcript_	MSTRG.43819.1	gene_id	MSTRG.43819;
X	StringTie	exon	98393822	98393919	.	-	.	transcript_	MSTRG.43819.1	gene_id	MSTRG.43819;
X	StringTie	exon	98396759	98397208	.	-	.	transcript_	MSTRG.43819.1	gene_id	MSTRG.43819;
X	StringTie	transcrip	111367233	111367765	.	-	.	transcript_	MSTRG.43971.1	gene_id	MSTRG.43971;
X	StringTie	exon	111367233	111367343	.	-	.	transcript_	MSTRG.43971.1	gene_id	MSTRG.43971;
X	StringTie	exon	111367364	111367505	.	-	.	transcript_	MSTRG.43971.1	gene_id	MSTRG.43971;
X	StringTie	exon	111367680	111367765	.	-	.	transcript_	MSTRG.43971.1	gene_id	MSTRG.43971;
X	StringTie	transcrip	124949459	124952307	.	-	.	transcript_	MSTRG.44122.1	gene_id	MSTRG.44122;
X	StringTie	exon	124949459	124950075	.	-	.	transcript_	MSTRG.44122.1	gene_id	MSTRG.44122;
X	StringTie	exon	124951979	124952307	.	-	.	transcript_	MSTRG.44122.1	gene_id	MSTRG.44122;
Y	StringTie	transcrip	3370143	3383785	.	+	.	transcript_	MSTRG.44187.1	gene_id	MSTRG.44187;
Y	StringTie	exon	3370143	3370621	.	+	.	transcript_	MSTRG.44187.1	gene_id	MSTRG.44187;
Y	StringTie	exon	3383705	3383785	.	+	.	transcript_	MSTRG.44187.1	gene_id	MSTRG.44187;
Y	StringTie	transcrip	24407834	24408619	.	+	.	transcript_	MSTRG.44262.1	gene_id	MSTRG.44262;

Y	StringTie	exon	24407834	24408156	.	+	.	transcript_	MSTRG.44262.1	gene_id	MSTRG.44262;
Y	StringTie	exon	24408539	24408619	.	+	.	transcript_	MSTRG.44262.1	gene_id	MSTRG.44262;
Y	StringTie	transcrip	10380556	10385519	.	-	.	transcript_	MSTRG.44275.1	gene_id	MSTRG.44275;
Y	StringTie	exon	10380556	10380851	.	-	.	transcript_	MSTRG.44275.1	gene_id	MSTRG.44275;
Y	StringTie	exon	10381060	10381212	.	-	.	transcript_	MSTRG.44275.1	gene_id	MSTRG.44275;
Y	StringTie	exon	10383056	10383144	.	-	.	transcript_	MSTRG.44275.1	gene_id	MSTRG.44275;
Y	StringTie	exon	10385415	10385519	.	-	.	transcript_	MSTRG.44275.1	gene_id	MSTRG.44275;
Y	StringTie	transcrip	10380601	10385508	.	-	.	transcript_	MSTRG.44275.2	gene_id	MSTRG.44275;
Y	StringTie	exon	10380601	10380846	.	-	.	transcript_	MSTRG.44275.2	gene_id	MSTRG.44275;
Y	StringTie	exon	10381060	10381212	.	-	.	transcript_	MSTRG.44275.2	gene_id	MSTRG.44275;
Y	StringTie	exon	10383056	10383144	.	-	.	transcript_	MSTRG.44275.2	gene_id	MSTRG.44275;
Y	StringTie	exon	10385415	10385508	.	-	.	transcript_	MSTRG.44275.2	gene_id	MSTRG.44275;
Y	StringTie	transcrip	25247074	25262224	.	-	.	transcript_	MSTRG.44267.1	gene_id	MSTRG.44267;
Y	StringTie	exon	25247074	25247274	.	-	.	transcript_	MSTRG.44267.1	gene_id	MSTRG.44267;
Y	StringTie	exon	25262118	25262224	.	-	.	transcript_	MSTRG.44267.1	gene_id	MSTRG.44267;

Table S3. The list of differentially expressed lncRNAs between Tibetan and Yorkshire group.

Row.names	baseMean	log2FoldChange	pvalue	padj	TA249	TA251	TA253	YA273	YA302	YA338
MSTRG.42621	42945.17246	9.054355962	4.6E-171	5.7E-168	81719.24	80252.22	95215.76	131.7094	212.9192	139.1747
MSTRG.6486	599.3038534	5.009726177	1.32E-22	8.27E-20	849.8736	638.7772	1998.881	46.91022	42.76583	18.61572
MSTRG.35541	163.3573401	6.905038888	4.42E-22	1.84E-19	176.5438	386.3823	409.0833	6.314837	1.819822	0
MSTRG.7083	112.8347174	7.222282327	1.02E-18	3.2E-16	299.7139	143.3354	229.433	0	3.639645	0.886463
MSTRG.22266	419.3128747	-3.912765599	1.15E-16	2.87E-14	39.00386	29.08254	88.74294	541.2717	811.6408	1006.135
MSTRG.29167	237.8656796	3.414259064	1.65E-12	3.43E-10	579.9258	185.9205	538.951	36.9869	46.40547	39.00437
MSTRG.31613	734.7886528	2.072215317	1.29E-11	2.3E-09	1268.652	1061.513	1231.579	317.5461	283.8923	245.5502
MSTRG.34932	433.293014	2.838509343	1.47E-10	2.3E-08	1094.161	475.7072	711.0258	144.3391	64.6037	109.9214
MSTRG.26887	49.92186349	-3.984085754	1.92E-10	2.66E-08	4.105669	9.347959	4.328924	92.91831	103.7299	85.10044
MSTRG.44267	50.61604049	9.257506019	2.44E-10	3.05E-08	176.5438	116.3302	10.82231	0	0	0
MSTRG.14578	220.6957944	3.756557799	3.99E-10	4.25E-08	537.8427	277.3228	417.7412	8.119076	61.87396	21.27511
MSTRG.26349	33.86186153	-8.45550637	4.09E-10	4.25E-08	0	0	0	41.4975	81.89201	79.78166
MSTRG.20146	30.79061575	-8.318181086	7.39E-10	7.1E-08	0	0	0	65.85472	72.7929	46.09607
MSTRG.13220	199.5567015	-2.650004437	2.39E-09	2.13E-07	41.05669	74.78367	48.70039	227.3341	331.2077	474.2576
MSTRG.37120	114.4454127	4.941691919	2.57E-09	2.14E-07	468.0463	154.7606	42.20701	15.33603	4.549556	1.772926
MSTRG.34839	388.2575829	-3.493351942	1.53E-08	1.19E-06	17.44909	63.35839	109.3053	687.4151	1169.236	282.7817
MSTRG.42120	228.5895817	2.637706599	1.39E-07	1.02E-05	453.6765	163.0699	564.9246	45.10598	79.16228	65.59825
MSTRG.41778	29.11435743	-5.775785121	1.53E-07	1.06E-05	2.052835	1.038662	0	43.30174	93.72086	34.57205
MSTRG.26210	373.6143697	3.45017405	2.48E-07	1.63E-05	914.5378	858.9735	280.2978	9.923315	60.05414	117.8996
MSTRG.36741	296.6356346	3.964892869	2.91E-07	1.82E-05	333.5856	118.4075	1220.757	28.86782	7.27929	70.91703
MSTRG.20839	205.4386972	2.1184202	3.12E-07	1.86E-05	415.699	293.9414	292.2024	58.63777	110.0993	62.0524
MSTRG.9698	19.81434496	7.903055922	6.4E-07	3.63E-05	16.42268	8.309296	94.1541	0	0	0
MSTRG.7015	53.18766218	3.1879084	7.56E-07	4.11E-05	161.1475	57.12641	69.26278	10.82543	14.55858	6.20524
MSTRG.36786	31.5698236	5.352640508	1.19E-06	6.21E-05	47.2152	20.77324	116.8809	0	4.549556	0
MSTRG.18750	15.61813073	-7.338925796	1.38E-06	6.89E-05	0	0	0	11.72755	50.95503	31.0262
MSTRG.27122	19.56135232	6.913390526	1.84E-06	8.82E-05	42.08311	58.16508	16.23346	0	0	0.886463
MSTRG.24516	47.08883713	4.789588625	2.21E-06	0.000102	176.5438	4.154648	91.98963	2.706359	1.819822	5.318777
MSTRG.3404	38.58398736	2.977338571	2.56E-06	0.000114	71.84921	40.50782	93.07186	9.021195	8.189201	8.864629
MSTRG.18486	72.17159823	2.241740467	3.67E-06	0.000158	94.4304	101.7889	161.2524	27.96571	23.65769	23.9345
MSTRG.22154	181.2614254	2.0519654	4.64E-06	0.000193	232.9967	454.934	188.3082	61.34413	75.52263	74.46288
MSTRG.8034	121.759659	3.365675584	5E-06	0.000201	29.7661	183.8432	452.3726	16.23815	18.19822	30.13974
MSTRG.34838	477.9972341	-2.383949155	6.03E-06	0.000235	157.0419	128.7941	175.3214	416.7792	410.37	1579.677
MSTRG.40446	68.83445952	-2.307587407	7.62E-06	0.000285	12.31701	31.15986	25.97354	124.4925	107.3695	111.6943

MSTRG.42745	11.77361858	7.152633754	7.76E-06	0.000285	28.73969	7.270634	34.63139	0	0	0
MSTRG.49	24.9495356	4.743336113	8.25E-06	0.000294	16.42268	98.6729	29.22024	3.608478	0	1.772926
MSTRG.14097	44619.70867	1.810883234	8.7E-06	0.000302	90868.73	45201.53	72268.14	13751.91	29580.3	16047.64
MSTRG.42256	322.8327769	2.276057766	1.51E-05	0.000511	666.1449	180.7272	758.6439	77.58228	151.9552	101.9432
MSTRG.23445	10265.9626	1.601740364	1.6E-05	0.000524	15663.13	9043.631	21624.06	5037.435	4797.052	5430.472
MSTRG.10082	321.7594389	-2.473008681	1.78E-05	0.000571	112.9059	105.9435	75.75617	140.7306	988.1636	507.0568
MSTRG.7741	9.794036737	6.887417593	2.65E-05	0.000828	8.211339	33.23719	17.3157	0	0	0
MSTRG.23649	65.31461186	2.512278061	2.74E-05	0.000834	188.8608	88.28628	56.27601	13.53179	23.65769	21.27511
MSTRG.23416	2127.711921	-1.302586966	3.45E-05	0.001027	1218.357	1057.358	1406.9	2540.369	2832.554	3710.734
MSTRG.39076	297.0799804	-2.691315244	3.68E-05	0.001068	130.355	16.61859	91.98963	890.392	439.4871	213.6376
MSTRG.3410	104.3445503	-1.970812141	4.16E-05	0.001154	43.10953	60.2424	23.80908	198.4663	169.2435	131.1965
MSTRG.42049	52.26769529	2.327868076	4.15E-05	0.001154	125.2229	52.97177	83.33179	18.04239	13.64867	20.38865
MSTRG.12355	24.45076857	5.317398009	4.29E-05	0.001164	108.8002	33.23719	1.082231	0.90212	0.909911	1.772926
MSTRG.42165	54.56877017	2.316192717	5.47E-05	0.001453	71.84921	140.2194	60.60494	18.04239	13.64867	23.04803
MSTRG.42167	20.10805223	6.039735909	8.89E-05	0.002265	55.42654	62.31972	1.082231	0	1.819822	0
MSTRG.42706	49.4418572	2.693390577	8.75E-05	0.002265	139.5928	67.51303	49.78263	7.216956	24.5676	7.978166
MSTRG.15651	53.15523755	-2.663097764	9.16E-05	0.002289	10.26417	5.19331	28.13801	105.548	52.77485	117.0131
MSTRG.7074	1558.711708	1.951722061	0.000108	0.002647	3393.336	745.7594	3292.147	520.523	787.0732	613.4323
MSTRG.5473	7066.494479	2.043846249	0.000164	0.003938	12135.33	2929.027	19059.17	3041.045	1885.336	3349.057
MSTRG.5836	14.36328735	-4.292065597	0.000175	0.004116	2.052835	1.038662	1.082231	48.71445	19.10814	14.18341
MSTRG.37750	28.8116675	2.880937297	0.000253	0.005859	18.47551	88.28628	45.4537	4.510598	7.27929	8.864629
MSTRG.30986	105.8511399	1.412262679	0.000361	0.008194	157.0419	142.2967	162.3346	54.12717	54.59467	64.71179
MSTRG.12543	170.8781112	1.397262218	0.000375	0.008362	264.8157	207.7324	270.5577	91.11407	121.0182	70.03057
MSTRG.1016	11.25608335	-4.958563304	0.000393	0.008507	0	1.038662	1.082231	13.53179	18.19822	33.68559
MSTRG.23889	30.23506866	2.351866647	0.000395	0.008507	29.7661	60.2424	61.68717	11.72755	10.00902	7.978166
MSTRG.13970	49.06057159	2.361597498	0.000412	0.008718	74.92847	92.44092	79.00286	13.53179	30.02707	4.432314
MSTRG.1179	163.7790885	-1.311066103	0.000436	0.009067	74.92847	113.2142	94.1541	228.2362	227.4778	244.6638
MSTRG.18342	28.14583876	2.840551348	0.000452	0.009245	69.79638	14.54127	63.85163	3.608478	9.099112	7.978166
MSTRG.30640	33.54505142	-2.268861428	0.000464	0.009342	10.26417	13.50261	10.82231	52.32293	34.57663	79.78166
MSTRG.25441	27.450802	2.470397098	0.000491	0.009736	59.53221	54.01043	25.97354	11.72755	6.369379	7.091703

Table S4 The functional enrichment results of up-regulated DEGs using Metascape.

GroupID	Category	Term	Description	LogP	Log(q-value)	InTerm_InList	Symbols
1_Summary	GO Biological Processes	GO:0002274	myeloid leukocyte activation	-22.4552	-18.243	53/649	TSH,CTSS,CTSZ,CYBA,CYBB,FCER1G,GRN,HK3,HMOX1,ITGAX,ITGB2,LGALS3,MAPT,MIF,MMP9,CFP,PLAUR,RELB,S100A8,S100A9,S100A12,SLC2A5,SLC11A1,SLPI,SPI1,SYK,TYROBP,LAT2,CD84,GMFG,CKAP4,PADI2,COTL1,ARHGAP45,CLEC5A,TLR8,ADA2,DOK3,DCSTAMP,PRAM1,NFAM1,ADGRG3,A2M,LGALS3BP,PLEK,STX3,TIMP1,TIMP3,NCS1,FERMT3,SCIN,SCAMP5,ACE,LCP1,MFNG,SEMA4A
2_Summary	GO Biological Processes	GO:0002573	myeloid leukocyte differentiation	-13.8337	-10.7354	24/203	C1QC,CA2,RUNX1,CD74,CCR1,CSF1,CSF1R,FASN,FCER1G,HCLS1,INPP5D,MMP9,RELB,SPI1,TYROBP,MAFB,TREM2,FAM20C,LRRK1,DCSTAMP,UBASH3B,GAB3,IL34,NRROS,ADAM8,CR2,EGR3,IL7R,LGALS3,MFNG,SYK,VAV1,CD83,SEMA4A,NFAM1,ADGRG3,ALAS2,CSF3R,IRF8,CLEC5A
3_Summary	GO Biological Processes	GO:0050900	leukocyte migration	-10.4123	-7.40421	31/486	ADAM8,ATP1B1,CD48,CD74,CCR1,CSF1,CSF3R,DDT,FCER1G,HMOX1,INPP5D,ITGAX,ITGB2,L1CAM,LGALS3,MIF,MMP9,LGMN,S100A8,S100A9,S100A12,CCL2,CCL3L1,SYK,VAV1,CD84,DOK2,PADI2,SMPD3,P2RY12,CD200R1,CSF1R,EGR2,EGR3,HSPB1,PLAUR,RAP1GAP,ROBO1,STX3,FOSL1,GPNMB,SEMA4A,UNC5A,UNC5D,CYBA,KLKB1
4_Summary	KEGG Pathway	hsa04610	Complement and coagulation cascades	-9.57241	-6.66127	13/79	A2M,BDKRB2,C1QA,C1QB,C1QC,C2,CLU,CR2,ITGAX,ITGB2,KLKB1,PLAUR,MASP1,ADAM8,CPM,CTSH,CTSS,CTSZ,ACE,ENO1,CFP,LGMN,RCE1,ACP4,WFS1,DDT,FCER1G,MMP9,NT5E,S100A8,S100A9,S100A12,CCL3L1,SOCS3,CD200R1,METRNL,CD22,CD74,HK1,HMOX1,IL7R,LGALS3,MIF,SYK,VAV1,CD84,PRAM1,HLA-DRA,GNAO1,IRAK1,CCL2,PTGES,SLC11A1,SLPI,CD83,TREM2,CD48,ADGRE1,INPP5D,RELB,TAP1,LAT2,TLR8,SEMA4A,UNC93B1,IRF8
5_Summary	GO Biological Processes	GO:0001816	cytokine production	-9.34644	-6.4565	37/737	ADAM8,RUNX1,CD74,CLU,CSF1R,CYBA,CYBB,DDT,FCER1G,HK1,HMOX1,HSPB1,IRF8,INPP5D,IRAK1,MIF,RELB,S100A8,S100A9,S100A12,SLC11A1,SYK,CD84,SPHK1,CD83,HOMER2,LITAF,GPNMB,CLEC5A,TLR8,CD200R1,CCM2L,NFAM1,SCAMP5,TIGIT,RAB7B,NRROS
6_Summary	GO Biological Processes	GO:0002253	activation of immune response	-8.30657	-5.50938	33/662	A2M,ACTG1,C1QA,C1QB,C1QC,C2,RUNX1,CD22,CLU,CR2,CTSS,CYBA,FCER1G,HLA-DRA,INPP5D,IRAK1,ITGB2,LGALS3,CFP,MASP1,RELB,S100A8,S100A9,SYK,VAV1,LAT2,LPXN,TLR8,CLEC7A,UNC93B1,PRAM1,NFAM1,RAB7B,CTSH,CD200R1

7_Summary	GO Biological Processes	GO:0019221	cytokine-mediated signaling pathway	-8.21169	-5.44581	35/739	RUNX1, CD74, CCR1, CSF1, CSF1R, CSF2RB, CSF3R, FCER1G, IFI6, HLA-DRA, HMOX1, IRF8, IL2RB, IL7R, IL10RA, INPP5D, IRAK1, ITGAX, ITGB2, LCP1, MIF, MMP9, ROBO1, CCL2, CCL3L1, STX3, SYK, TIMP1, VAV1, SPHK1, SOCS3, IFI30, PADI2, TREM2, IL34
8_Summary	GO Biological Processes	GO:0030225	macrophage differentiation	-7.95823	-5.20847	9/41	C1QC, CSF1, CSF1R, HCLS1, MMP9, SPI1, GAB3, IL34, NRROS, CD74, FASN, INPP5D, DCSTAMP
9_Summary	GO Biological Processes	GO:0044283	small molecule biosynthetic process	-7.86148	-5.15447	35/763	ACACA, ACLY, AMPD3, ATF3, BCAT1, CD74, DHCR7, ENO1, FASN, HK1, HK3, FADS3, LSS, MIF, NT5E, PFKFB1, PLEK, PSPH, PYCR1, SCD, SREBF2, SYK, SPHK1, PTGES, DHRS9, EBP, SMPDL3A, HAAO, ADA2, ACSS2, MID1IP1, ACSS1, NUDT7, RIMKLA, NAT8L, ALAS2, CYBA, CYBB, GSS, ME1, PDHB, SLC25A39, AHCY, FOLR1, HMOX1, ETHE1, ALDH4A1, ME3, PNPLA3, CYGB, PLIN5, ATP1B1, CKB, CLU, COMT, CTSH, CYP4B1, ITGB2, FAM20C, CHCHD10, TST, CHST1, SPOCK2, ENTPD6, CDS1, TXNRD1, INPP5D, SMPD3
9_Member	GO Biological Processes	GO:0044283	small molecule biosynthetic process	-7.86148	-5.15447	35/763	ACACA, ACLY, AMPD3, ATF3, BCAT1, CD74, DHCR7, ENO1, FASN, HK1, HK3, FADS3, LSS, MIF, NT5E, PFKFB1, PLEK, PSPH, PYCR1, SCD, SREBF2, SYK, SPHK1, PTGES, DHRS9, EBP, SMPDL3A, HAAO, ADA2, ACSS2, MID1IP1, ACSS1, NUDT7, RIMKLA, NAT8L
9_Member	GO Biological Processes	GO:0046394	carboxylic acid biosynthetic process	-6.74904	-4.19009	23/415	ACACA, ACLY, BCAT1, CD74, ENO1, FASN, HK1, HK3, FADS3, MIF, PFKFB1, PSPH, PYCR1, SCD, SYK, PTGES, DHRS9, HAAO, ACSS2, MID1IP1, ACSS1, RIMKLA, NAT8L
9_Member	GO Biological Processes	GO:0016053	organic acid biosynthetic process	-6.73049	-4.18108	23/416	ACACA, ACLY, BCAT1, CD74, ENO1, FASN, HK1, HK3, FADS3, MIF, PFKFB1, PSPH, PYCR1, SCD, SYK, PTGES, DHRS9, HAAO, ACSS2, MID1IP1, ACSS1, RIMKLA, NAT8L
9_Member	GO Biological Processes	GO:0051188	cofactor biosynthetic process	-6.21432	-3.71816	18/285	ACACA, ACLY, ALAS2, CYBA, CYBB, ENO1, FASN, GSS, HK1, HK3, ME1, PDHB, PFKFB1, SCD, HAAO, SLC25A39, ACSS2, ACSS1
9_Member	GO Biological Processes	GO:0006084	acetyl-CoA metabolic process	-5.84102	-3.4282	7/37	ACACA, ACLY, FASN, PDHB, ACSS2, ACSS1, NUDT7
9_Member	GO Biological Processes	GO:0051186	cofactor metabolic process	-5.56504	-3.17895	25/559	ACACA, ACLY, AHCY, ALAS2, CYBA, CYBB, ENO1, FASN, FOLR1, GSS, HK1, HK3, HMOX1, ME1, NT5E, PDHB, PFKFB1, SCD, PTGES, ETHE1, HAAO, SLC25A39, ACSS2, ACSS1, NUDT7
9_Member	GO Biological Processes	GO:0032787	monocarboxylic acid metabolic process	-5.10687	-2.82852	25/594	ACACA, ACLY, CD74, ENO1, FASN, HK1, HK3, FADS3, ME1, MIF, PDHB, PFKFB1, SCD, ALDH4A1, PTGES, DHRS9, ME3, HAAO, ACSS2, MID1IP1, PNPLA3, ACSS1, CYGB, NUDT7, PLIN5
9_Member	GO Biological Processes	GO:0035384	thioester biosynthetic process	-4.87133	-2.63229	7/51	ACACA, ACLY, FASN, PDHB, SCD, ACSS2, ACSS1
9_Member	GO Biological Processes	GO:0071616	acyl-CoA biosynthetic process	-4.87133	-2.63229	7/51	ACACA, ACLY, FASN, PDHB, SCD, ACSS2, ACSS1

9_Member	GO Biological Processes	GO:0072330	monocarboxylic acid biosynthetic process	-4.75514	-2.52975	16/295	ACACA,ACLY,CD74,ENO1,FASN,HK1,HK3,FADS3,MIF,PFKFB1,SCD,PTGES,DHRS9,ACSS2,MID1IP1,ACSS1
9_Member	GO Biological Processes	GO:0017144	drug metabolic process	-4.71388	-2.49295	29/789	ACLY,AHCY,AMPD3,ATP1B1,CKB,CLU,COMT,CTSH,CYBA,CYBB,CYP4B1,ENO1,FOLR1,HK1,HK3,ITGB2,NT5E,PDHB,PFKFB1,PYCR1,DHRS9,ME3,ETHE1,HAAO,ADA2,ACSS2,FAM20C,ACSS1,CHCHD10
9_Member	KEGG Pathway	hsa00620	Pyruvate metabolism	-4.53919	-2.35835	6/39	ACACA,ME1,PDHB,ME3,ACSS2,ACSS1
9_Member	GO Biological Processes	GO:0006633	fatty acid biosynthetic process	-4.43134	-2.28736	11/157	ACACA,ACLY,CD74,FASN,FADS3,MIF,SCD,PTGES,ACSS2,MID1IP1,ACSS1
9_Member	GO Biological Processes	GO:0009108	coenzyme biosynthetic process	-4.32907	-2.20681	13/221	ACACA,ACLY,ENO1,FASN,HK1,HK3,ME1,PDHB,PFKFB1,SCD,HAAO,ACSS2,ACSS1
9_Member	GO Biological Processes	GO:0006732	coenzyme metabolic process	-4.23784	-2.13289	17/361	ACACA,ACLY,AHCY,ENO1,FASN,FOLR1,HK1,HK3,ME1,NT5E,PDHB,PFKFB1,SCD,HAAO,ACSS2,ACSS1,NUDT7
9_Member	GO Biological Processes	GO:0033866	nucleoside bisphosphate biosynthetic process	-4.13034	-2.0487	7/66	ACACA,ACLY,FASN,PDHB,SCD,ACSS2,ACSS1
9_Member	GO Biological Processes	GO:0034030	ribonucleoside bisphosphate biosynthetic process	-4.13034	-2.0487	7/66	ACACA,ACLY,FASN,PDHB,SCD,ACSS2,ACSS1
9_Member	GO Biological Processes	GO:0034033	purine nucleoside bisphosphate biosynthetic process	-4.13034	-2.0487	7/66	ACACA,ACLY,FASN,PDHB,SCD,ACSS2,ACSS1
9_Member	GO Biological Processes	GO:0072522	purine-containing compound biosynthetic process	-4.02433	-1.97353	14/270	ACACA,ACLY,AMPD3,ENO1,FASN,HK1,HK3,NT5E,PDHB,PFKFB1,SCD,ADA2,ACSS2,ACSS1
9_Member	GO Biological Processes	GO:0006637	acyl-CoA metabolic process	-3.67726	-1.72517	8/103	ACACA,ACLY,FASN,PDHB,SCD,ACSS2,ACSS1,NUDT7
9_Member	GO Biological Processes	GO:0035383	thioester metabolic process	-3.67726	-1.72517	8/103	ACACA,ACLY,FASN,PDHB,SCD,ACSS2,ACSS1,NUDT7
9_Member	GO Biological Processes	GO:0006790	sulfur compound metabolic process	-3.65354	-1.70855	16/366	ACACA,ACLY,AHCY,COMT,FASN,GSS,PDHB,SCD,TST,CHST1,PTGES,SPOCK2,ETHE1,ACSS2,ACSS1,NUDT7
9_Member	GO Biological Processes	GO:0055086	nucleobase-containing small molecule metabolic process	-3.55667	-1.63893	25/742	ACACA,ACLY,AHCY,AMPD3,ATP1B1,ENTPD6,CDS1,ENO1,FASN,HK1,HK3,ME1,NT5E,PDHB,PFKFB1,SCD,TXNRD1,SMPDL3A,HAAO,ADA2,ACSS2,FAM20C,ACSS1,NUDT7,CHCHD10

9_Member	GO Biological Processes	GO:0006085	acetyl-CoA biosynthetic process	-3.55224	-1.63893	4/21	ACLY, PDHB, ACSS2, ACSS1
9_Member	GO Biological Processes	GO:0009165	nucleotide biosynthetic process	-3.43537	-1.55689	15/345	ACACA, ACLY, AMPD3, CDS1, ENO1, FASN, HK1, HK3, ME1, PDHB, PFKFB1, SCD, HAAO, ACSS2, ACSS1
9_Member	GO Biological Processes	GO:0009152	purine ribonucleotide biosynthetic process	-3.41983	-1.54811	12/238	ACACA, ACLY, AMPD3, ENO1, FASN, HK1, HK3, PDHB, PFKFB1, SCD, ACSS2, ACSS1
9_Member	GO Biological Processes	GO:1901293	nucleoside phosphate biosynthetic process	-3.39597	-1.53281	15/348	ACACA, ACLY, AMPD3, CDS1, ENO1, FASN, HK1, HK3, ME1, PDHB, PFKFB1, SCD, HAAO, ACSS2, ACSS1
9_Member	GO Biological Processes	GO:0009260	ribonucleotide biosynthetic process	-3.19889	-1.40003	12/252	ACACA, ACLY, AMPD3, ENO1, FASN, HK1, HK3, PDHB, PFKFB1, SCD, ACSS2, ACSS1
9_Member	GO Biological Processes	GO:0006164	purine nucleotide biosynthetic process	-3.12414	-1.34655	12/257	ACACA, ACLY, AMPD3, ENO1, FASN, HK1, HK3, PDHB, PFKFB1, SCD, ACSS2, ACSS1
9_Member	GO Biological Processes	GO:0006631	fatty acid metabolic process	-3.12179	-1.34579	15/370	ACACA, ACLY, CD74, FASN, FADS3, MIF, SCD, PTGES, ACSS2, MID1IP1, PNPLA3, ACSS1, CYGB, NUDT7, PLIN5
9_Member	GO Biological Processes	GO:0046390	ribose phosphate biosynthetic process	-3.10944	-1.33976	12/258	ACACA, ACLY, AMPD3, ENO1, FASN, HK1, HK3, PDHB, PFKFB1, SCD, ACSS2, ACSS1
9_Member	GO Biological Processes	GO:0072521	purine-containing compound metabolic process	-2.87655	-1.17761	19/559	ACACA, ACLY, AHCY, AMPD3, ATP1B1, ENO1, FASN, HK1, HK3, NT5E, PDHB, PFKFB1, SCD, ADA2, ACSS2, FAM20C, ACSS1, NUDT7, CHCHD10
9_Member	GO Biological Processes	GO:0033865	nucleoside bisphosphate metabolic process	-2.83324	-1.15137	8/138	ACACA, ACLY, FASN, PDHB, SCD, ACSS2, ACSS1, NUDT7
9_Member	GO Biological Processes	GO:0033875	ribonucleoside bisphosphate metabolic process	-2.83324	-1.15137	8/138	ACACA, ACLY, FASN, PDHB, SCD, ACSS2, ACSS1, NUDT7
9_Member	GO Biological Processes	GO:0034032	purine nucleoside bisphosphate metabolic process	-2.83324	-1.15137	8/138	ACACA, ACLY, FASN, PDHB, SCD, ACSS2, ACSS1, NUDT7
9_Member	GO Biological Processes	GO:0006753	nucleoside phosphate metabolic process	-2.76833	-1.10148	21/660	ACACA, ACLY, AMPD3, ATP1B1, CDS1, ENO1, FASN, HK1, HK3, ME1, NT5E, PDHB, PFKFB1, SCD, SMPDL3A, HAAO, ACSS2, FAM20C, ACSS1, NUDT7, CHCHD10
9_Member	GO Biological Processes	GO:0009150	purine ribonucleotide metabolic process	-2.73211	-1.07505	17/489	ACACA, ACLY, AMPD3, ATP1B1, ENO1, FASN, HK1, HK3, NT5E, PDHB, PFKFB1, SCD, ACSS2, FAM20C, ACSS1, NUDT7, CHCHD10

9_Member	GO Biological Processes	GO:0008610	lipid biosynthetic process	-2.67142	-1.02746	22/718	ACACA, ACLY, CD74, CDS1, DHCR7, FASN, INPP5D, FADS3, LSS, MIF, SCD, SREBF2, SPHK1, PTGES, DHRS9, EBP, SMPD3, ACSS2, MID1IP1, PNPLA3, ACSS1, PLIN5
9_Member	GO Biological Processes	GO:0009259	ribonucleotide metabolic process	-2.58749	-0.97631	17/505	ACACA, ACLY, AMPD3, ATP1B1, ENO1, FASN, HK1, HK3, NT5E, PDHB, PFKFB1, SCD, ACSS2, FAM20C, ACSS1, NUDT7, CHCHD10
9_Member	GO Biological Processes	GO:0044272	sulfur compound biosynthetic process	-2.53198	-0.93996	9/189	ACACA, ACLY, FASN, GSS, PDHB, SCD, CHST1, ACSS2, ACSS1
9_Member	GO Biological Processes	GO:0009117	nucleotide metabolic process	-2.46722	-0.89554	20/654	ACACA, ACLY, AMPD3, ATP1B1, CDS1, ENO1, FASN, HK1, HK3, ME1, NT5E, PDHB, PFKFB1, SCD, HAAO, ACSS2, FAM20C, ACSS1, NUDT7, CHCHD10
9_Member	GO Biological Processes	GO:0006163	purine nucleotide metabolic process	-2.45044	-0.88468	17/521	ACACA, ACLY, AMPD3, ATP1B1, ENO1, FASN, HK1, HK3, NT5E, PDHB, PFKFB1, SCD, ACSS2, FAM20C, ACSS1, NUDT7, CHCHD10
9_Member	GO Biological Processes	GO:0019693	ribose phosphate metabolic process	-2.42554	-0.86659	17/524	ACACA, ACLY, AMPD3, ATP1B1, ENO1, FASN, HK1, HK3, NT5E, PDHB, PFKFB1, SCD, ACSS2, FAM20C, ACSS1, NUDT7, CHCHD10
10_Summary	GO Biological Processes	GO:0001894	tissue homeostasis	-7.11252	-4.51314	17/221	ABCA4, ACACA, ACTG1, ADAM8, CA2, CSF1, CSF1R, CTSH, HSPB1, INPP5D, SPP1, SYK, HOMER2, WHRN, LRRK1, DCSTAMP, UBASH3B, ADRB3, AMPD3, SCD, SLC11A1, WFS1, MFN2, METRNL, CST3, GPNMB, ACE, TIMP1, CAPN3, NCS
11_Summary	GO Biological Processes	GO:0038156	interleukin-3-mediated signaling pathway	-6.54454	-4.01362	4/5	CSF2RB, CSF3R, FCER1G, SYK, HMOX1, ITGB2, CD84, PRAM1, S100A12, LAT2, SREBF2, NCS1, SMPD3, SCAMP5, CD83
12_Summary	KEGG Pathway	hsa04142	Lysosome	-6.28547	-3.77228	12/123	CD68, CTSB, CTSD, CTSH, CTSS, CTSZ, LGMN, SGSH, SLC11A1, LAPTM5, LITAF, NAGPA, CST3, MMP9, PEPD, ACTG1, CSF2RB, TUBA1C, CYGB
13_Summary	KEGG Pathway	hsa04145	Phagosome	-6.03257	-3.56201	13/154	ACTG1, CTSS, CYBA, CYBB, HLA-DRA, ITGB2, MSR1, NCF2, TAP1, ATP6V1F, CLEC7A, TUBA1C, RAB7B
14_Summary	KEGG Pathway	hsa04380	Osteoclast differentiation	-6.02598	-3.56201	12/130	CSF1, CSF1R, CYBA, CYBB, NCF2, RELB, SPI1, SYK, TYROBP, FOSL1, SOCS3, TREM2, ACTG1, BDKRB2, IFI6, HSPB1, IGFBP6, MMP9, PLAUR, LGMN, ROBO1, VAV1, DOK2, ATP6V1F, ESRP1, FAM20C, RHBDF2, UBASH3B, ACP4, SH
15_Summary	GO Biological Processes	GO:0050865	regulation of cell activation	-5.88145	-3.4282	27/610	ADAM8, CAPN3, RUNX1, CD22, CD74, EGR3, FCER1G, HMOX1, IL7R, INPP5D, ITGB2, LGALS3, MIF, PLEK, RPS6KA1, CCL2, SYK, TYROBP, VAV1, CD84, CD83, GPNMB, PRAM1, UBASH3B, CYGB, NFAM1, TIGIT, CR2, LCP1, MFNG, RELB, SLC11A1, SPI1, LAT2, MAFB, SEMA4A, CLEC7A, ADGRG3, IRAK1, NT5E, S100A8, S100A9, FERMT3, CELSR2, CCM2L, ARHGDIA, CSF1, STX3, LPX
16_Summary	GO Biological Processes	GO:0043269	regulation of ion transport	-5.62103	-3.22178	28/668	ATP1B1, CA2, CAPN3, CCR1, CTSS, CYBA, CYBB, GNAO1, KCNQ1, LGALS3, MIF, MMP9, FXYD1, RGS2, RYR2, CCL2, SNCG, SYK, WFS1, CD84, HOMER2, RAMP3, NEDD4L, KCNH3, KCNK13, P2RY12, UBASH3B, CHCHD10, ALAS2, ATP2A3, BDKRB2, CKB, COMT, HMOX1, S100A8, S100A9, SLC11A1, TACR1, NCS1, FAM20A, TRPV5, TTC7A, SLC4A11, HK1, HK3, PIM3, IFI6, ADRB3, CTSZ, ACE, RCAN1, SLC2A5, CRYAB, ENO1, SPHK1

17_Summary	GO Biological Processes	GO:0002683	negative regulation of immune system process	-5.57711	-3.18449	22/450	A2M,C1QC,RUNX1,CD22,CD74,DDT,FCER1G,HMOX1,IL7R,INPP5D,LGALS3,MIF,CCL2,CD84,LPXN,MAFB,GPNMB,PADI2,UBASH3B,CD200R1,TIGIT,RAB7B,CYGB,RELB,SLC11A1,CD83,HOMER2,TLR8
18_Summary	GO Biological Processes	GO:0022617	extracellular matrix disassembly	-5.55723	-3.17758	9/76	A2M,ADAM8,CAPG,CST3,CTSS,KLKB1,LCP1,MMP9,TIMP1,PLK3,PLEK,SREBF2,CCNB2,MFN2,PADI2,MID1IP1,MRPL11,SCIN,CHCHD10,ITGAX,ITGB2,SPP1,SPOCK2
19_Summary	GO Biological Processes	GO:0007229	integrin-mediated signaling pathway	-5.39247	-3.04954	10/101	FCER1G,ITGAX,ITGB2,PLEK,SYK,TIMP1,TYROBP,VAV1,FERMT3,PRAM1,A2M,ACTG1,RHOC,CAPN3,CCR1,EVPL,FOLR1,HMOX1,HSPB1,KLKB1,LCP1,PLAUR,PPL,S100A8,SLC11A1,SPP1,MFN2,PIK3R5,P2RY12,UBASH3B,CCM2L,CLU,LGALS3BP,TIMP3,CYBA,WFS1,TRPV5
20_Summary	GO Biological Processes	GO:0019882	antigen processing and presentation	-5.38312	-3.04602	13/176	CD74,CTSD,CTSH,CTSS,ACE,FCER1G,HLA-DRA,LGMN,RELB,SLC11A1,TAP1,IFI30,TREM2,PLK3,IL10RA,IRAK1,ITGAX,ITGB2,SYK,SPHK1,CLEC7A,CTSB

Table S5 The functional enrichment results of down-regulated DEGs.

GroupID	Category	Term	Description	LogP	Log(q-value)	InTerm_InList	Symbols
1_Summary	GO Biological Processes	GO:0072073	kidney epithelium development	-5.6967934	-1.67515	9/139	EYA1,FGF2,FGFR2,VEGFA,PROM1,SLIT2,ADIPOQ,GREM1,IRX3,ACTA2,PLAG1,MET,ACTG2,COCH,TBX1,TGFB3,TPM1,MMRN2,KIT,CXCR5,CHGA,NGFR
2_Summary	GO Biological Processes	GO:0048705	skeletal system morphogenesis	-5.4145828	-1.67515	11/237	BMPR1B,COCH,EYA1,FGFR2,HOXC8,MGP,MMP16,TBX1,TGFB3,GREM1,PAPPA2,COMP,ASPN,FGF2,KIT,PENK,KAZALD1,IGSF10,ACTA2
2_Member	GO Biological Processes	GO:0048705	skeletal system morphogenesis	-5.4145828	-1.67515	11/237	BMPR1B,COCH,EYA1,FGFR2,HOXC8,MGP,MMP16,TBX1,TGFB3,GREM1,PAPPA2
2_Member	GO Biological Processes	GO:0030282	bone mineralization	-4.5251726	-1.3544	7/109	BMPR1B,COMP,FGFR2,MGP,TGFB3,GREM1,ASPN
2_Member	GO Biological Processes	GO:0031214	biomineral tissue development	-4.4082789	-1.2753	8/155	BMPR1B,COMP,FGFR2,MGP,TBX1,TGFB3,GREM1,ASPN
2_Member	GO Biological Processes	GO:0001501	skeletal system development	-4.0886672	-1.17445	14/515	BMPR1B,COMP,COCH,EYA1,FGF2,FGFR2,HOXC8,KIT,MGP,MMP16,TBX1,TGFB3,GREM1,PAPPA2
2_Member	GO Biological Processes	GO:0070167	regulation of biomineral tissue development	-4.0685629	-1.17445	6/89	BMPR1B,COMP,MGP,TGFB3,GREM1,ASPN
2_Member	GO Biological Processes	GO:0060349	bone morphogenesis	-3.5627633	-1.01336	6/110	BMPR1B,COCH,FGFR2,MMP16,TGFB3,PAPPA2
2_Member	GO Biological Processes	GO:0030500	regulation of bone mineralization	-3.5316519	-1.00996	5/72	BMPR1B,COMP,MGP,TGFB3,GREM1

2_Member	GO Biological Processes	GO:0001503	ossification	-3.5240856	-1.00996	11/383	BMPR1B, COMP, FGFR2, MGP, MMP16, PENK, TGFB3, GREM1, ASPN, KAZALD1, IGSF10
2_Member	GO Biological Processes	GO:0060348	bone development	-3.5092121	-1.00462	8/209	BMPR1B, COCH, FGFR2, KIT, MMP16, TGFB3, GREM1, PAPPA2
2_Member	GO Biological Processes	GO:0035988	chondrocyte proliferation	-3.4893368	-1.00431	3/17	BMPR1B, COMP, MMP16
2_Member	GO Biological Processes	GO:0030278	regulation of ossification	-2.2992083	-0.32509	6/194	BMPR1B, COMP, FGFR2, MGP, TGFB3, GREM1
2_Member	GO Biological Processes	GO:0061448	connective tissue development	-2.212237	-0.29673	7/267	ACTA2, BMPR1B, COMP, COCH, FGF2, MGP, GREM1
2_Member	GO Biological Processes	GO:0051216	cartilage development	-2.165121	-0.26943	6/207	BMPR1B, COMP, COCH, FGF2, MGP, GREM1
2_Member	GO Biological Processes	GO:0002063	chondrocyte development	-2.1635302	-0.26943	3/48	BMPR1B, COMP, COCH
3_Summary	GO Biological Processes	GO:0014706	striated muscle tissue development	-5.4101893	-1.67515	14/391	EYA1, FGF2, FGFR2, MYH11, SGCG, TBX1, TPM1, VEGFA, COPS2, NEBL, NUPR1, GREM1, FHOD3, DIPK2A, CPE, TGFB3, SLIT2, COMP, DES, KRT8, MET, CAP2, RHOBTB3, ANKRD53, STMN4, ESPN, KIT, CHODL, CNN1, SCN3B
4_Summary	GO Biological Processes	GO:0070848	response to growth factor	-5.0687594	-1.55557	19/734	BMPR1B, COMP, FGF2, FGFR2, GTF2F2, HSPA5, NGFR, PENK, TBX1, TGFB3, VEGFA, HAP1, SLIT2, ACAP2, GREM1, ASPN, FNDC4, MMRN2, SHISA2
4_Member	GO Biological Processes	GO:0070848	response to growth factor	-5.0687594	-1.55557	19/734	BMPR1B, COMP, FGF2, FGFR2, GTF2F2, HSPA5, NGFR, PENK, TBX1, TGFB3, VEGFA, HAP1, SLIT2, ACAP2, GREM1, ASPN, FNDC4, MMRN2, SHISA2
4_Member	GO Biological Processes	GO:0071363	cellular response to growth factor stimulus	-4.7640378	-1.39697	18/703	BMPR1B, COMP, FGF2, FGFR2, GTF2F2, HSPA5, NGFR, PENK, TBX1, TGFB3, VEGFA, HAP1, SLIT2, ACAP2, GREM1, ASPN, MMRN2, SHISA2

4_Member	GO Biological Processes	GO:0090287	regulation of cellular response to growth factor stimulus	-3.9183197	-1.17445	10/287	FGF2,FGFR2,HSPA5,TGFB3,VEGFA,SLIT2,GREM1,ASPN,MMRN2,SHISA2
4_Member	GO Biological Processes	GO:0090288	negative regulation of cellular response to growth factor stimulus	-3.4157609	-0.96703	7/164	HSPA5,TGFB3,SLIT2,GREM1,ASPN,MMRN2,SHISA2
5_Summary	GO Biological Processes	GO:0032787	monocarboxylic acid metabolic process	-4.5518707	-1.3544	16/594	ACAA1,ALOX12B,FAAH,HAL,KIT,LDHB,PDK4,PLP1,PRKAR2B,ACSM3,HACD1,ADIPOQ,LPGAT1,PHGDH,OGDHL,AMDHD1,SUCLA2,FGF2,LEPR,PLCH2
5_Member	GO Biological Processes	GO:0032787	monocarboxylic acid metabolic process	-4.5518707	-1.3544	16/594	ACAA1,ALOX12B,FAAH,HAL,KIT,LDHB,PDK4,PLP1,PRKAR2B,ACSM3,HACD1,ADIPOQ,LPGAT1,PHGDH,OGDHL,AMDHD1
5_Member	GO Biological Processes	GO:0006633	fatty acid biosynthetic process	-3.5304068	-1.00996	7/157	ALOX12B,PDK4,PLP1,ACSM3,HACD1,ADIPOQ,LPGAT1
5_Member	GO Biological Processes	GO:0006631	fatty acid metabolic process	-3.0488649	-0.75052	10/370	ACAA1,ALOX12B,FAAH,PDK4,PLP1,PRKAR2B,ACSM3,HACD1,ADIPOQ,LPGAT1
5_Member	GO Biological Processes	GO:0046394	carboxylic acid biosynthetic process	-2.6793188	-0.5284	10/415	ALOX12B,PDK4,PLP1,ACSM3,SUCLA2,HACD1,ADIPOQ,LPGAT1,PHGDH,OGDHL
5_Member	GO Biological Processes	GO:0016053	organic acid biosynthetic process	-2.6717419	-0.5284	10/416	ALOX12B,PDK4,PLP1,ACSM3,SUCLA2,HACD1,ADIPOQ,LPGAT1,PHGDH,OGDHL
5_Member	GO Biological Processes	GO:0072330	monocarboxylic acid biosynthetic process	-2.5484095	-0.48854	8/295	ALOX12B,PDK4,PLP1,ACSM3,HACD1,ADIPOQ,LPGAT1,OGDHL
5_Member	GO Biological Processes	GO:0042304	regulation of fatty acid biosynthetic process	-2.1143239	-0.24667	3/50	PDK4,ADIPOQ,LPGAT1
5_Member	GO Biological Processes	GO:0044283	small molecule biosynthetic process	-2.0316749	-0.19058	13/763	ALOX12B,FGF2,LEPR,PDK4,PLP1,ACSM3,SUCLA2,HACD1,ADIPOQ,PLCH2,LPGAT1,PHGDH,OGDHL

6_Summary	GO Biological Processes	GO:0031346	positive regulation of cell projection organization	-4.2720789	-1.17445	12/372	HSPA5, KIT, NGFR, RELN, TGFB3, VEGFA, HAP1, SLIT2, KCTD17, ESPN, CHODL, NEGR1, PLAG1, MMD, GJC2, IRX3, ADIPOQ, CAMK2A, EYA1, SLC6A4, IGSF10
7_Summary	KEGG Pathway	hsa04060	Cytokine-cytokine receptor interaction	-4.1367061	-1.17445	10/270	CXCR5, BMPR1B, IL2RA, KIT, LEPR, MET, NGFR, TGFB3, VEGFA, IL20RB
8_Summary	GO Biological Processes	GO:0009636	response to toxic substance	-4.0532137	-1.17445	14/519	ACAA1, FGFR2, GSTM3, MET, MT1A, ND6, PENK, SLC6A4, ADIPOQ, ZNF277, NUPR1, GJC2, PPP1R1B, GRIN3A, HSPA5, PRKAR2B, PHGDH
9_Summary	GO Biological Processes	GO:0002551	mast cell chemotaxis	-3.9664904	-1.17445	3/12	CHGA, KIT, VEGFA, TGFB3, COMP, FGFR2, COCH, TPM1, RHOBTB3
10_Summary	GO Biological Processes	GO:0008585	female gonad development	-3.9111142	-1.17445	6/95	BMPR1B, HSPA5, KIT, VEGFA, SLIT2, NUPR1, TGFB3, RHOBTB3, NGFR, MAPK10, SLC6A4, ADIPOQ, FGFR2, KRT8, PLAG1, PENK, ASPN, FNDC4
11_Summary	GO Biological Processes	GO:0006936	muscle contraction	-3.8178483	-1.14976	11/354	ACTA2, ACTG2, CHGA, CNN1, COMP, DES, KIT, MYH11, SCN7A, TPM1, SCN3B, ACSM3, SGCG, SLC6A4, SLIT2, ADIPOQ
12_Summary	KEGG Pathway	M00011	Citrate cycle, second carbon oxidation, 2-oxoglutarate =>	-3.7529876	-1.13189	3/14	SUCLG2, SUCLA2, OGDHL, PDK4, ACSM3, HACD1, LDHB, PHGDH, TKTL2, ND1, LEPR, ATP8, ND2, ADIPOQ, STEAP4, AK5, HAL, AMDHD1
13_Summary	GO Biological Processes	GO:2001028	positive regulation of endothelial cell chemotaxis	-3.6586641	-1.06242	3/15	FGF2, MET, VEGFA, COL4A5, COMP, FGFR2, IL2RA, KIT, NGFR, RELN, CISH, DGKB, GTF2F2, PDK4, HAP1, ADIPOQ, GREM1, MMRN2, SHISA2, CXCR5, TGFB3, ACAA1, ANKRD53, MAPK10
14_Summary	GO Biological Processes	GO:1904706	negative regulation of vascular smooth muscle cell proliferation	-3.4812993	-1.00431	4/41	CNN1, TGFB3, TPM1, ADIPOQ, FGF2, FGFR2, SLIT2, DIPK2A, DES, SGCG, BMPR1B, IL2RA, NGFR, SLC6A4, NUDT6, NUPR1, GREM1, IL20RB
15_Summary	GO Biological Processes	GO:0001101	response to acid chemical	-3.3507991	-0.93103	10/338	FGFR2, KRT8, PDK4, MAPK10, PTGFR, SLC6A4, TBX1, VEGFA, ADIPOQ, RRAGD, MMP16, TGFB3, EYA1, CPE, TPM1, SLIT2, GREM1, ASPN, CRISPLD1, COMP, IRX3, FGF2, GTF2F2, SHISA2, SGCG, COPS2, NUPR1, CHODL
16_Summary	GO Biological Processes	GO:0048871	multicellular organismal homeostasis	-3.2854559	-0.89284	12/476	ALOX12B, ESRRG, LEPR, MET, PDK4, PRKAR2B, VEGFA, PROM1, ADIPOQ, PLAC8, IL20RB, IRX3

17_Summary	GO Biological Processes	GO:0032102	negative regulation of response to external stimulus	-3.2718594	-0.88739	10/346	FGF2, IL2RA, TNFAIP6, SLIT2, ADIPOQ, GREM1, IL20RB, RNF125, FNDC4, GRIN3A
18_Summary	GO Biological Processes	GO:0035235	ionotropic glutamate receptor signaling pathway	-2.9807592	-0.71662	3/25	GRIA3, GRID1, GRIN3A, CAMK2A, PPP1R1B, MAPK10, HCAR1, RELN
19_Summary	GO Biological Processes	GO:0035265	organ growth	-2.9326485	-0.69106	7/198	COCH, FGF2, FGFR2, LEPR, PLAG1, SLC6A4, DIPK2A, BMPR1B, COMP, VEGFA, SLIT2, PLAC8
19_Member	GO Biological Processes	GO:0035265	organ growth	-2.9326485	-0.69106	7/198	COCH, FGF2, FGFR2, LEPR, PLAG1, SLC6A4, DIPK2A
19_Member	GO Biological Processes	GO:0098868	bone growth	-2.2418296	-0.30151	3/45	COCH, FGFR2, LEPR
19_Member	GO Biological Processes	GO:0048589	developmental growth	-2.1453139	-0.26357	12/655	BMPR1B, COMP, COCH, FGF2, FGFR2, LEPR, PLAG1, SLC6A4, VEGFA, SLIT2, PLAC8, DIPK2A
20_Summary	GO Biological Processes	GO:0120034	positive regulation of plasma membrane bounded cell projection assembly	-2.879718	-0.64528	5/100	KIT, TGFB3, HAP1, KCTD17, ESPN, CDH17, CHGA, MET, TPM1, VEGFA, ANKRD53, SLIT2, COCH, EYA1, TBX1

Table S6 The trans-targeting genes prediction of lncRNAs.

LncRNA_id	LncRNA position	Gene_id	Gene symbol	Location	Pearson correlation	adjust-value
MSTRG.42621	X:22068981-22069290	ENSSSCG00000035520	-	X:22068922-22069186	0.995617347	0.011830751
MSTRG.6486	11:23914858-23927125	ENSSSCG00000034192	GNAO1	6:29417601-29592698	0.997464435	0.009773049
MSTRG.7083	12:1562157-1567167	ENSSSCG00000024045	CASTOR2	3:11956300-12039913	0.997587298	0.009773049
MSTRG.7083	12:1562157-1567167	ENSSSCG00000002452	LGMN	7:114175183-114217319	0.99782614	0.008623517
MSTRG.7083	12:1562157-1567167	ENSSSCG00000001633	-	7:37281046-37281420	0.999921998	0.000411091
MSTRG.7083	12:1562157-1567167	ENSSSCG00000032337	CDK20	AEMK02000556.1:20291-25858	0.999370275	0.007563287
MSTRG.7083	12:1562157-1567167	ENSSSCG00000039633	CRLF2	AEMK02000569.1:672382-694311	0.999274366	0.007563287
MSTRG.22266	2:85486691-85513807	ENSSSCG00000000135	KCTD17	5:10707354-10718983	0.998675101	0.007563287
MSTRG.31613	6:48231448-48239613	ENSSSCG00000012459	APOOL	X:68281804-68355006	-0.998830351	0.007563287
MSTRG.31613	6:48231448-48239613	ENSSSCG00000005467	HSDL2	1:253124403-253191760	-0.998365052	0.007563287
MSTRG.31613	6:48231448-48239613	ENSSSCG00000017673	INTS2	12:36435812-36494591	-0.998490461	0.007563287
MSTRG.31613	6:48231448-48239613	ENSSSCG00000024399	EVC2	8:4904743-5023720	-0.998542742	0.007563287
MSTRG.34932	7:24097492-24098545	ENSSSCG00000034866	TACC1	AEMK02000380.1:8513-65498	0.997366215	0.009773049
MSTRG.34932	7:24097492-24098545	ENSSSCG00000000419	RDH16	5:22251753-22293249	0.998218497	0.007563287
MSTRG.26349	4:17192373-17281592	ENSSSCG00000014378	PCDHB4	2:142817677-142820079	0.999054659	0.007563287
MSTRG.42120	AEMK02000569.1:775344-782495	ENSSSCG00000030209	MFNG	5:10319826-10334504	0.998814058	0.007563287
MSTRG.42120	AEMK02000569.1:775344-782495	ENSSSCG00000033262	CD74	2:151399969-151433844	0.99746226	0.009773049
MSTRG.42120	AEMK02000569.1:775344-782495	ENSSSCG00000015830	UNC5D	15:50201405-50460534	0.997935524	0.00838697
MSTRG.42120	AEMK02000569.1:775344-782495	ENSSSCG00000014347	PROB1	2:141282086-141285145	0.998860126	0.007563287

MSTRG.42120	AEMK02000569.1:775344-782495	ENSSSCG00000034036	CCDC187	AEMK02000682.1:1393127-1425625	0.998394407	0.007563287
MSTRG.41778	AEMK02000359.1:10834-14297	ENSSSCG00000015299	STEAP4	9:68544721-68570585	0.99791494	0.00838697
MSTRG.41778	AEMK02000359.1:10834-14297	ENSSSCG00000034356	ERVW-1	6:10405594-10406889	0.998154143	0.00766912
MSTRG.36786	8:383135-384704	ENSSSCG00000032380	C1orf53	10:20732726-20738659	0.997955335	0.00838697
MSTRG.24516	3:41482346-41486942	ENSSSCG00000006588	S100A9	4:96235327-96239285	0.998238385	0.007563287
MSTRG.24516	3:41482346-41486942	ENSSSCG00000034313	SELENOW	6:53472322-53474985	0.997598071	0.009773049
MSTRG.42745	X:38902375-38908896	ENSSSCG00000033262	CD74	2:151399969-151433844	0.998959813	0.007563287
MSTRG.42745	X:38902375-38908896	ENSSSCG00000015830	UNC5D	15:50201405-50460534	0.997953664	0.00838697
MSTRG.42745	X:38902375-38908896	ENSSSCG00000034036	CCDC187	AEMK02000682.1:1393127-1425625	0.998201858	0.007563287
MSTRG.42745	X:38902375-38908896	ENSSSCG00000017106	MED10	16:75517023-75524760	0.99884127	0.007563287
MSTRG.42745	X:38902375-38908896	ENSSSCG00000016577	ATP6V1F	18:19753759-19756658	0.998197747	0.007563287
MSTRG.49	1:824024-825885	ENSSSCG00000029281	PCDHAC2	2:142472588-142688089	0.997560054	0.009773049
MSTRG.14097	14:111470803-111477982	ENSSSCG00000010554	SCD	14:111461157-111478031	0.998610472	0.007563287
MSTRG.14097	14:111470803-111477982	ENSSSCG00000013492	APBA3	2:74929342-74945290	0.998381096	0.007563287
MSTRG.42256	AEMK02000682.1:839454-840881	ENSSSCG00000034566	NPDC1	AEMK02000682.1:836624-843893	0.99822849	0.007563287
MSTRG.42256	AEMK02000682.1:839454-840881	ENSSSCG00000022998	PKIG	17:47007780-47044047	0.99736946	0.009773049
MSTRG.42256	AEMK02000682.1:839454-840881	ENSSSCG00000022029	RAP1GAP	6:79662137-79735361	0.997864072	0.008556559
MSTRG.42256	AEMK02000682.1:839454-840881	ENSSSCG00000024736	ACP4	6:55534864-55541862	0.999499407	0.007563287

MSTRG.42049	AEMK02000452.1:3760564-3768547	ENSSSCG00000039798	SLC11A1	15:120433736-120447490	0.99887915	0.007563287
MSTRG.42049	AEMK02000452.1:3760564-3768547	ENSSSCG00000035032	-	7:7608189-7635391	0.997363926	0.009773049
MSTRG.7074	12:1190077-1192134	ENSSSCG00000013046	COX8A	2:8101611-8103285	0.998722893	0.007563287
MSTRG.7074	12:1190077-1192134	ENSSSCG00000002907	COX6B1	6:45091285-45102669	0.998298402	0.007563287
MSTRG.7074	12:1190077-1192134	ENSSSCG00000023527	NRROS	13:133470645-133496264	0.997774157	0.008802805
MSTRG.30986	6:13674896-13675970	ENSSSCG00000020706	CBR1	13:199600314-199719628	-0.998288946	0.007563287
MSTRG.1179	1:66413113-66467841	ENSSSCG00000024399	EVC2	8:4904743-5023720	0.998423454	0.007563287
MSTRG.18342	17:27099733-27123696	ENSSSCG00000013046	COX8A	2:8101611-8103285	0.997979084	0.00838697
MSTRG.18342	17:27099733-27123696	ENSSSCG00000002907	COX6B1	6:45091285-45102669	0.997489711	0.009773049
MSTRG.18342	17:27099733-27123696	ENSSSCG00000034058	ARHGDIA	12:1093195-1121126	0.998833901	0.007563287
MSTRG.18342	17:27099733-27123696	ENSSSCG00000023527	NRROS	13:133470645-133496264	0.998742862	0.007563287
MSTRG.25441	3:94069841-94073841	ENSSSCG00000013556	ADGRE1	2:72217039-72306013	0.997416341	0.009773049

Table S7 Functional enrichment analysis of the trans-targeting genes of lncRNAs.

GroupID	Category	Term	Description	LogP	Log(q-value)	InTerm_InList	Symbols
1_Summary	GO Biological Processes	GO:0006636	unsaturated fatty acid biosynthetic process	-4.19381	0	3/48	CBR1,CD74,SCD
2_Summary	GO Biological Processes	GO:0006826	iron ion transport	-3.86082	0	3/62	SLC11A1,ATP6V1F,STEAP4,S100A9,COX6B1,COX8A
3_Summary	GO Biological Processes	GO:0022900	electron transport chain	-3.69881	0	4/180	COX6B1,COX8A,RDH16,STEAP4,ATP6V1F
4_Summary	GO Biological Processes	GO:0048002	antigen processing and presentation of peptide antigen	-2.8254	0	3/140	CD74,LGMN,SLC11A1,RAP1GAP,S100A9,UNC5D,CRLF2,NRROS
5_Summary	GO Biological Processes	GO:0031345	negative regulation of cell projection organization	-2.51518	0	3/180	ARHGDIA,RAP1GAP,ACP4,S100A9,KCTD17,GNAO1,LGMN
6_Summary	GO Biological Processes	GO:0098742	cell-cell adhesion via plasma-membrane adhesion molecules	-2.0019	0	3/276	PCDHB4,PCDHAC2,UNC5D

2.38 1.4

3.64

#NUM!

0.3
